# Co-Catalytic Coupling of Alkyl Halides and Alkenes: the Curious Role of Lutidine

**DOI:** 10.1021/jacs.4c15812

**Published:** 2025-02-03

**Authors:** Roshini Hanumanthu, Parul Sharma, Avery Ethridge, Jimmie D. Weaver

**Affiliations:** Department of Chemistry, Oklahoma State University, Stillwater, Oklahoma 74078, United States

## Abstract

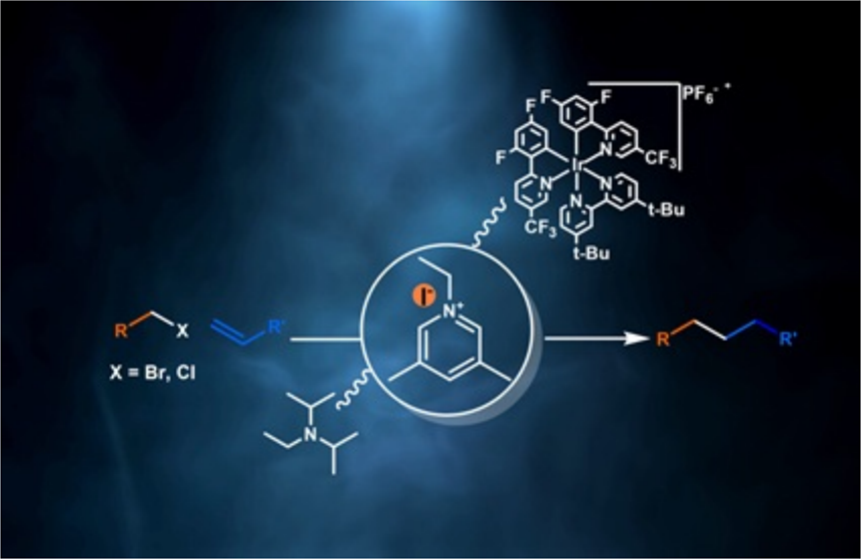

Continuous pressure to shorten synthetic sequences along with the concomitant expansion of scope makes the use of alkyl bromides, chlorides, and oxygen based leaving groups- which are abundant and readily available feedstocks, highly attractive for C–C bond synthesis. However, selective activation of these bonds to generate radical intermediates remains challenging and is generally unfeasible using traditional activation strategies. Herein, we report a dual catalytic activation strategy to access primary, secondary, and tertiary alkyl radicals from respective alkyl chlorides and bromides, as well as primary tosylates and trifluoroacetates. While the method relies on visible light and a photocatalyst to facilitate electron transfer, based on reduction potentials, the substrates are not expected to be reduceable, and yet they are reduced in the presence of lutidine. Ultimately, our investigation revealed that lutidine was a precatalyst and ultimately led to the use of lutidinium iodide salt which served as a critical cocatalyst that resulted in improved reaction profiles. Our studies revealed two critical roles that lutidinium iodide salts play which made it possible to engage otherwise unreactive substrates: nucleophilic exchange and halogen atom transfer by the lutidinium radical. In short, this work converts unactivated alkyl chlorides, bromides, tosylates, and trifluoroacetates to radicals that can be used for C–C bond formation without the need for preactivation—effectively expediting synthesis

## Introduction

**Scheme 1:**
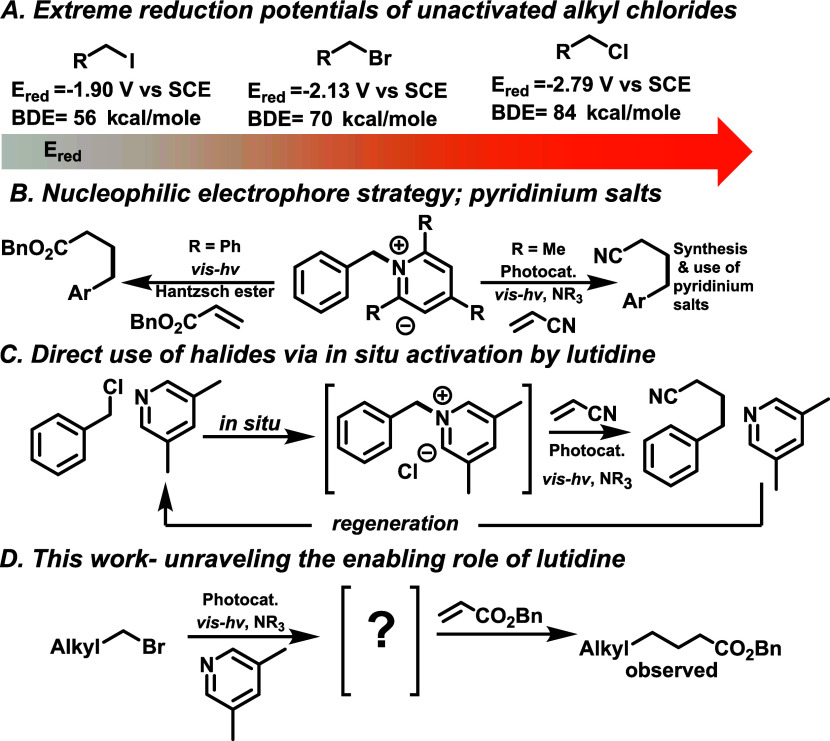
Activation Strategies for Alkyl Halides

**Scheme 2:**
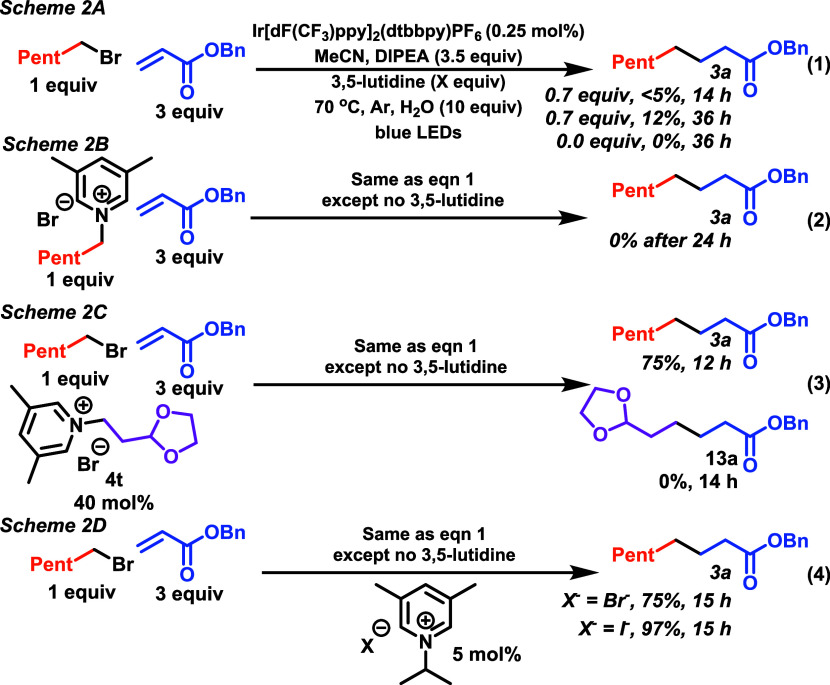
Initial Identification and Inspection

Chemical reactions that activate and elaborate feedstock chemicals are ideal for rapid enhancement of structural complexity while increasing the overall sustainability of synthesis. In this vein, the use of commercially available alkyl halides, especially chlorides, which are the most commercially abundant halides, is ideal.^1−5^ Alkyl chlorides are, however, rather recalcitrant,^6^ with relatively few viable strategies for selective and catalytic activation of such bonds.^7−11^ For some time, our group has been interested in methods that can activate C–X bonds and has developed several photocatalytic reactions that utilize light-induced single electron transfer (SET) to selectively fragment C–X bonds.^12^ We and many others have studied the activation of aryl–halide bonds.^13,14^ However, reduction of aryl halides typically relies on the π*-orbital to first capture the electron, which then populates the σ*-orbital and fragmentation of the halide. However, for alkyl halides, which do not possess the relevant orbitals, this strategy fails. Instead, a direct SET into the C–X σ*-orbital would be required,^15^ which is quite challenging, as reflected in their extreme- and functional group limiting, reduction potentials (Scheme 1A). Thus, we investigated the use of nucleophilic pyridines that could facilitate the transformation by exploiting the inherent electrophilicity of the halide to deliver a nucleophilic pyridine that, once added, could aid in electron capture. We first studied benzylic collidinium salts (*E*_1/2_ −1.46 V vs SCE)^16,17^—which proved capable of leveling the reduction potential across benzyl halides with a wide range of reduction potentials and functional groups (Scheme 1B). A drawback to this approach was that collidine was not nucleophilic enough to displace the halide *in situ*, and its corresponding salt had to be synthesized separately at higher temperatures. Kozlowski, Rosenthal, and Watson groups recently performed a study on related Katritzky salts (*E*_1/2_ −0.92 V vs SCE^18^).^19^ They concluded that the rate of fragmentation of the reduced pyridinium radical was a function of the C–N bond strength and the bulk around the C–N bond. Hence, the ortho-flanking aryl groups of the Katritzky salt were important to achieve fragmentation. However, recently, we showed that 3,5-lutidine (3,5-dimethylpyridine) could accomplish both the *in situ* displacement and the activation when used in catalytic amounts (Scheme 1C).^20^ Based on Kozlowski, Rosenthal, and Watson’s report, we anticipated this approach would be limited to the benzylic halides and not extend to aliphatic halides, as they make a stronger C–N bond. However, at longer reaction times, we observed the formation of a product consistent with a successful coupling of a simple alkyl bromide and an alkene. This came as a surprise, as we did not expect to form a primary alkyl radical under these conditions (eq 1, Scheme 2).

We then set out to investigate how this was feasible and whether we could extend the scope beyond primary alkyl bromides (Scheme 1D).

## Results and Discussion

We started our investigation by subjecting hexyl bromide and benzyl acrylate to various reaction conditions to form the Giese product. Initially, after 14 h of irradiation, only trace amounts of Giese product (**3a**, Scheme 2) were observed with 70 mol % lutidine, but no product was observed in the absence of lutidine- suggesting a key role served by lutidine. Furthermore, after 36 h, the yield increased to 12% (Scheme 2A). This reaction profile was suggestive of an induction period which might indicate the formation of a critical intermediate. We suspected the intermediate to be the lutidinium salt- which has been shown to be capable of generating benzyl radicals upon reduction and fragmentation of the C–N bond.^16,17,20^ So, we started the reaction directly with the suspected intermediate salt. Surprisingly, it yielded no desired product (Scheme 2B). This revealed a different operative mechanism than previously observed^16,20^ for the case of alkyl halides. Next, a crossover experiment was carried out using hexyl bromide and a substoichiometric amount of *N*-ethyl dioxolane lutidinium bromide (Scheme 2C). We observed that the yield further increased to 75% in just 12 h of irradiation, and no alkylation product resulting from the dioxolane salt was observed. These results indicate that there was no fragmentation of the C–N bond of the salt- as the salt stayed intact throughout the reaction and that the induction period could be avoided by the inclusion of catalytic lutidinium salt. Finally, we further reduced the salt loading and looked at the effect of the counterion on the yield of the desired alkylation (Scheme 2D). Notably, using the catalytic amount of iodide salt was significantly higher yielding than the substoichiometric bromide salt (Scheme 2C).

This indicated that the iodide counterion played a significant role in the reaction which was reminiscent of the Finkelstein reaction—in which iodide salts are used to facilitate nucleophilic substitution by displacing less easily substituted halides (*i.e.*, chlorides) to yield a transient iodide that is ultimately substituted more readily by another nucleophile.^21^ If operative, this would be a simple way to engage other, less reactive electrophiles, such as chlorides and sulfonates, and would be a major advance. Several attempts to use more traditional iodide salts failed, *vide infra*. Thus, we set out to screen different pyridinium salts targeting unactivated alkyl bromides and chlorides.

**Scheme 3:**
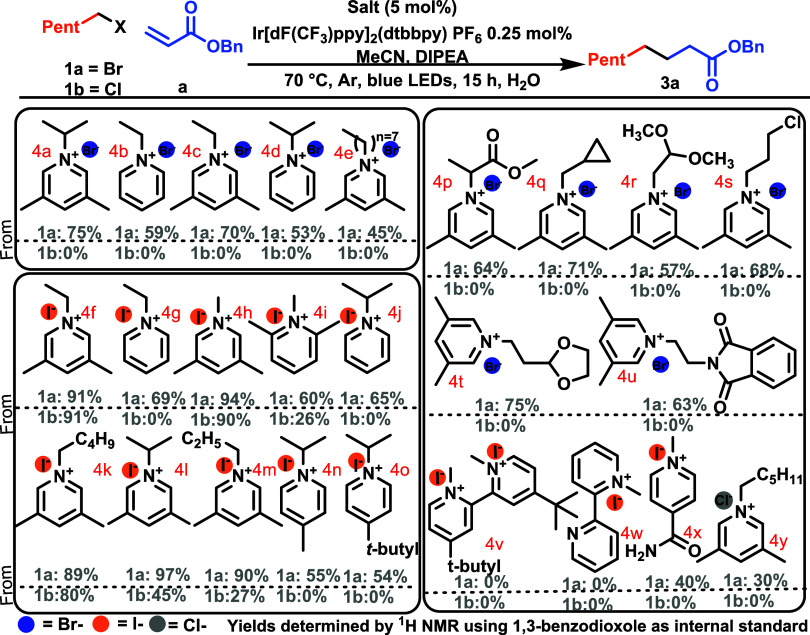
Exploring the Pyridinium Core

First, we performed an optimization for the Giese coupling involving primary, secondary, and tertiary alkyl bromides and chlorides with benzyl acrylate as the alkene (see Supporting Information (SI)). We found optimal conditions involved use of 5 mol % *N*-alkyl lutidinium salt, 0.25 mol % photocatalyst Ir[dF(CF_3_)ppy]_2_(dtbbpy)PF_6_,^20^ with variable equivalents of diisopropylethylamine (DIPEA) which served as a reductant, and water, under blue LED irradiation, with heating at 70 °C. After identifying the optimized conditions, we then built a library of *N*-alkylpyridinium and bipyridinum salts that varied in (1) *N*-alkyl substituent, (2) substitution pattern, and (3) the counterion and then studied the effect of the salt on two parallel reactions using hexyl bromide and chloride (**1a** and **1b**, Scheme 3). Immediately, it became clear that hexyl chloride (**1b**) required an iodide counterion for the desired alkylation to occur (**4a**–**4e** vs **4f**). We then evaluated the effect of the substitution pattern on the pyridinium ring. A primary concern here was that nucleophilic radicals might undergo addition to the pyridinium core.^22^

Indeed, we consistently observed that unsubstituted pyridinium salts (**4g**) performed worse for bromide **1a** and completely failed in the case of chloride **1b**. An assessment of the substitution pattern around the pyridinium core (**4f**–**4o**) revealed that 3,5-lutidinium resulted in a significantly superior yield compared to 2,6-lutidinium (**4h** vs **4i**). Finally, we examined the effect of the *N*-substituent for hexyl bromide (**1a, 4a**–**4e**). While we observed some differences in the performance of *N*-alkyl-3,5-lutidinium bromide salts (75–45%, **4a**–**4e**), there was little difference among the different iodide salts (89–97%, **4f**, **4h**, **4i**, **4k**, **4l**, **4m**). Whereas for hexyl chloride (**1b**), a more prominent difference was observed (91–27%, **4f**, **4h**, **4i**, **4k**, **4l**, **4m**), suggesting a link between the *N*-substituent and the ability of the iodide to substitute the chloride. Bipyridinium di- and monosalts (**4v**, **4w**), which could possibly be generated in a number of processes in which bipyridines are used as ligands,^23^ both failed to generate product from either hexyl-chloride or -bromide (**1a** and **1b**). The major takeaways from this study were that iodide salts are superior to bromides for alkyl bromides and absolutely essential in alkyl chlorides, that 3,5-substitution is superior to other patterns on the pyridine core, and hexyl chloride was sensitive to the nature of the *N*-substituent.

Presuming that iodide must substitute the halogen- at least in the case of chlorides, it seemed likely that this would be highly sensitive to the structure of the halide, so, we screened 1°–, 2°–, and 3°-bromides and chlorides using the top five *N*-alkyl lutidinium iodide salts from Scheme 3 (Table 1). Consistently for alkyl bromides, *N*-isopropyl lutidinium iodide gave the highest yield. Whereas for chlorides, smaller *N*-alkyl chains were optimal. Finally, optimization and control experiments underscored the critical nature of DIPEA, salt, blue LEDs, and photocatalysts (see SI). The absence of DIPEA left the starting materials intact. Omitting the salt resulted in a 0% yield for product **3a**, and the acrylate reacted with the amine to form an adduct^24^ (see SI for more details). The reaction failed without blue LEDs or the photocatalyst leaving the alkene unreacted at the end of the reaction.

**Table 1:**
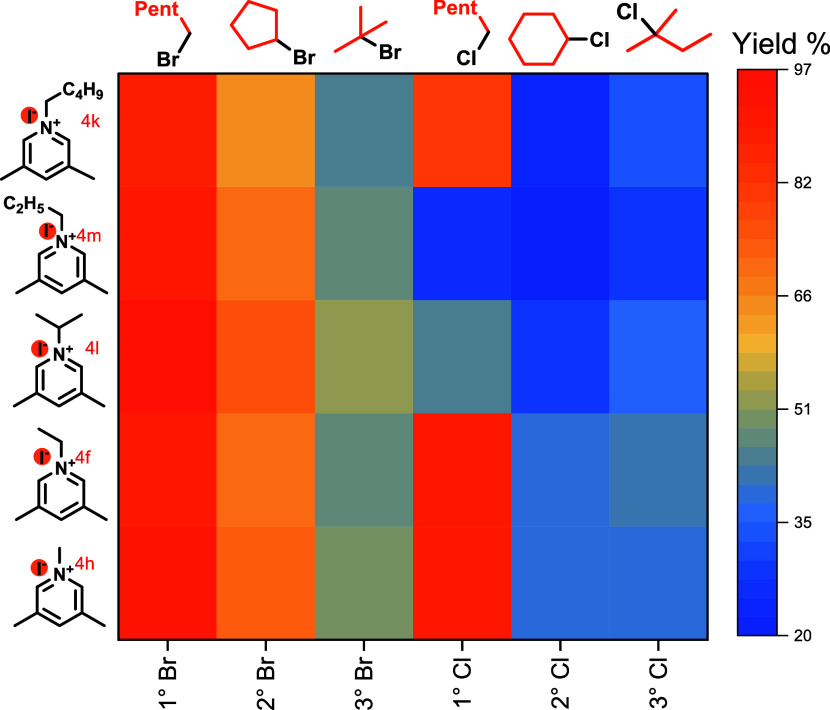
Heat Map of Lutidinium Iodides vs Different Classes of Alkyl Halides

### Mechanism

To better understand the reaction mechanism, several experiments were conducted. First, to confirm the substitution of hexyl chloride by iodide, we looked for the corresponding hexyl iodide via EI-GCMS (see SI for details) in the reaction media at an intermediate reaction time (6 h) which confirmed its presence.

**Scheme 4:**
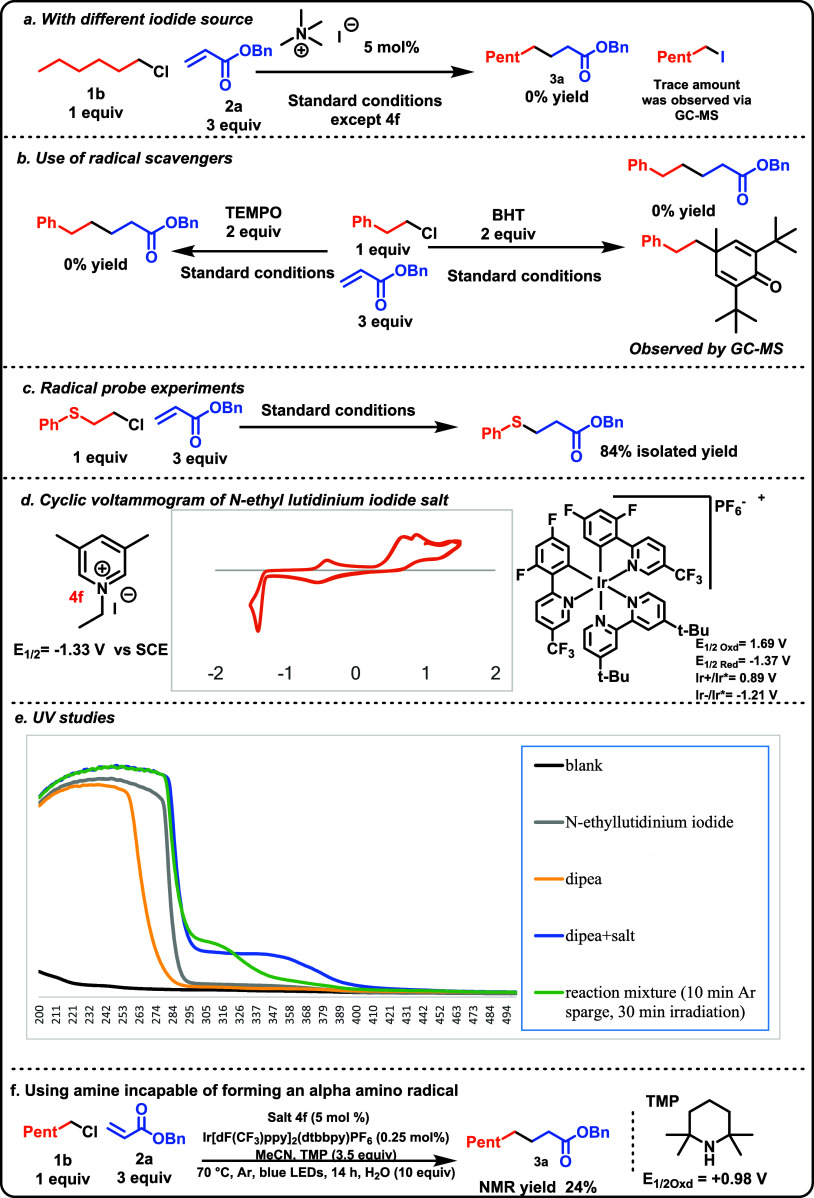
Mechanistic Insight

Next, to address whether lutidinium serves merely as an iodide counterion in the substitution step or plays a more critical role in the XAT (halogen atom transfer) process, further investigations were carried out. Reactions were performed using alternative iodide sources, such as tetramethylammonium iodide and sodium iodide, in place of lutidinium iodide (Scheme 4a). Despite the complete homogeneity of the reaction mixture and successful generation of the alkyl iodide intermediate- detected via GCMS, these reactions failed to produce the desired product. This indicates that at the least, the lutidinium salt is a superior iodide source and may have a greater role in the XAT step, but this was still unclear.

Tertiary amines like DIPEA are known to generate α-amino radicals, which Leonori has shown facilitate XAT.^25,26^ However, it remains unclear whether XAT in this reaction is facilitated by the lutidinium radical or the DIPEA-derived α-amino radical. To investigate this, reactions were carried out using alternative reductants, such as DABCO and tetramethylpiperidine (TMP), which can supply an electron but do not produce α-amino radicals. Notably, replacing DIPEA with TMP as the reductant yielded 24% of the desired product, demonstrating that the lutidinium radical plays a critical role in facilitating XAT in this system. Jui has shown that DIPEA derived α amino radicals are readily trapped by electron deficient alkenes.^27^ So, this result, coupled with aforementioned control study that highlighted the role of the lutidinium salt in the prevention of the DIPEA-acrylate adduct, indicates that lutidinium is key to XAT.

When radical scavengers like (2,2,6,6-tetramethylpiperidine-1-yl)oxyl (TEMPO) and butylated hydroxytoluene (BHT)^28^ were added to the reaction mixture (Scheme 4b), normal reactivity was completely suppressed, and a BHT adduct was detected by GCMS, supporting the radical mechanism. Furthermore, in the case of a substrate that contained a β-sulfur relative to the chloride (**33**, Scheme 4c), the sulfur-addition product (**33a**) was isolated and none of the ethylene inserted product was observed—suggesting both the formation of a primary radical from the chloride and that C–C formation is slower than unimolecular fragmentation. These results directly support the presence of discrete radical intermediates in the reaction.^29^

We performed cyclic voltammetry on *N*-ethyl lutidinium iodide salt (Scheme 4d), which showed an oxidation event at +0.6 V vs SCE, which is likely due to the oxidation of the iodide anion.^30−32^ On the negative sweep, a reversible peak was observed at −1.33 V vs SCE, which likely corresponds to the reduction of lutidinium to its radical form. Additionally, the photocatalyst shows a reduction potential of −1.37 V vs SCE.^33^ This indicates that a reduced photocatalyst (reduced by the amine, measured *E*_1/2_ = 0.61 V vs SCE)^20^ will transfer an electron to the lutidinium ion, resulting in the formation of the lutidinium radical. Finally, a series of UV–vis absorption studies were conducted to probe the possibility of an EDA complex that could play a key role in the reaction (Scheme 4e). Indeed, we do observe a new absorption band when DIPEA is added to the salt, with maximum absorption at λ 350 nm which tails into the visible region. However, we do not think that this EDA complex is productive, as the control studies indicated the necessity of the photocatalyst.^34^

**Scheme 5:**
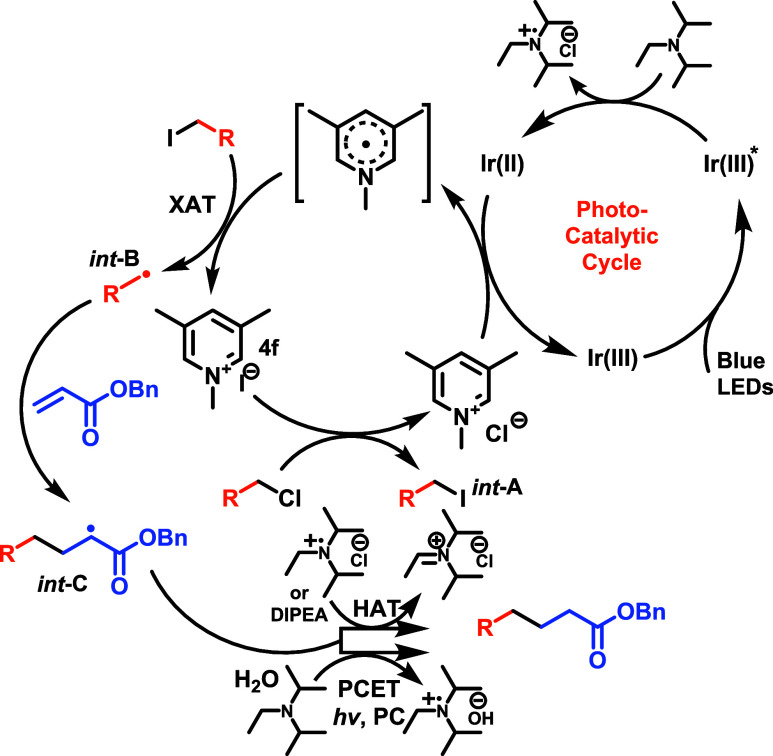
Plausible Mechanism

With this information in mind, we propose a plausible mechanism (Scheme 5). The first step is the absorption of a blue photon by the photocatalyst to produce strongly oxidizing Ir(III)*(Ir*(III)/Ir(II) = +1.42 V vs SCE in MeCN),^35^ which undergoes reductive quenching by the amine^16^ (measured *E*_1/2_ = 0.61 V vs SCE) to give an Ir(II) species. This is supported by Stern–Volmer analysis which revealed quenching of the photocatalyst by both the iodide of the lutidinium iodide and DIPEA (see SI for details). Simultaneously, the anion of the lutidinium salt **4f** performs a substitution, generating alkyl iodide (***int*****-A**, detected by GCMS, *vide supra*) and lutidinium chloride. Next, the reduced photocatalyst, (Ir(II/III) = −1.37 V vs SCE,^33^ in 0.1 M TBAH/MeCN), undergoes SET to the *N*-methyl lutidinium (measured *E*_1/2_ = −1.33 V vs SCE in MeCN, Scheme 4d), generating a lutidinium radical and returning the photocatalyst to its initial state. Next, we propose the alkyl radical (***int*****-*****B***) is generated through an XAT by the lutidinium radical^25,26,36^ which then immediately eliminates iodide to rearomatize and close the lutidinium cycle. The alternative to this would be XAT via the α amino radical formed from DIPEA. This proposed role of lutidinium radical in XAT is supported by the following, (1) we have shown that in the absence of lutidinium salt this radical is readily and efficiently trapped by the acrylate (see SI for details)—which Jui has demonstrated stems from α amino radical and electron deficient alkene, (2) the use of TMP which is incapable of forming an α amino radical still works indicating the reaction still works even in the absence of α amino radicals, and (3) a close inspection of Leonori’s system^25^ reveals generally lower yields for aliphatic bromides than what we observe using our suboptimal lutidinium bromides (**4a** and **4c**) which are not capable of *in situ* formation of the iodide—suggesting that lutidinium radical is at least on par with, if not superior to, α amino radicals at XAT in the challenging case of bromides. Importantly, the use of lutidinium radical for XAT allows us to avoid the complications associated with α amino radicals—namely the trapping by the alkene and undesired reduction of the halide which we do not observe in substantial quantities under optimal conditions.

Our mechanism fails to elucidate an explanation for the exceptional substitution capability of the lutidinium iodide that we have observed (*i.e.*, primary, secondary, and tertiary chlorides).^21^ It seems feasible that reduction of the lutidinium counterion maybe occurring prior to nucleophilic substitution which serves to enhance the nucleophilicity of the iodide by loss of its counterion. Currently, we do not have conclusive evidence one way or the other. In either case, ***int-B*** then adds to the electron-deficient alkenes to create a new C-centered radical ***int-C***, which then yields the product after hydrogen atom transfer from the amine radical cation or directly from the amine.^37−39^ However, there is some evidence that a minor pathway for acrylates is proton-coupled electron transfer (PCET) which completes the reaction.^16,20^

### Substrate Scope

With these conditions in hand, we next turned our attention to evaluating the scope of this reaction (Table 2). First, using a diverse set of primary alkyl bromides and chlorides, we observed excellent functional group compatibility with substrates containing alcohols (Table 2, **8a**), esters (**6a**, **19a**, **20a**), ethers (**7a** and **11a**), trifluoromethyl containing substrates, which would likely be challenging ions to utilize—highlight the advantage of radical formation (**10a**, **16a**), as well as longer chain alkanes (**12a**) which provide many options for intramolecular C–H abstraction. The reaction was tolerant of acid-sensitive acetals (**13a**).

**Table 2:**
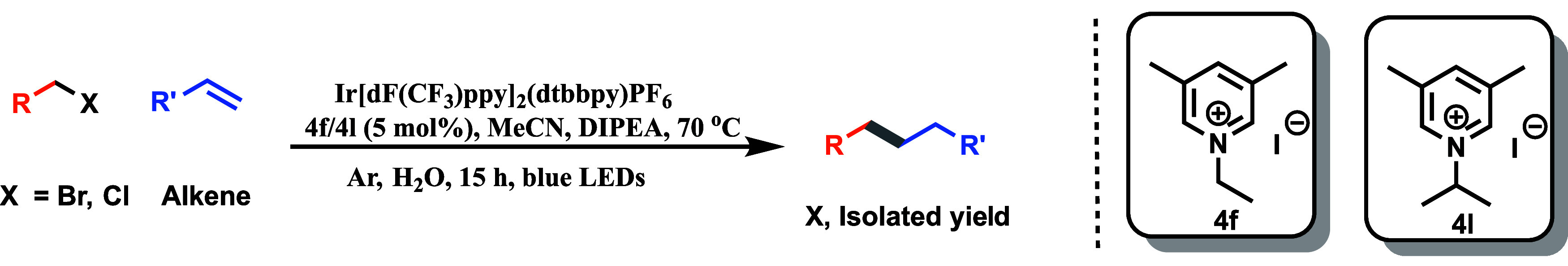

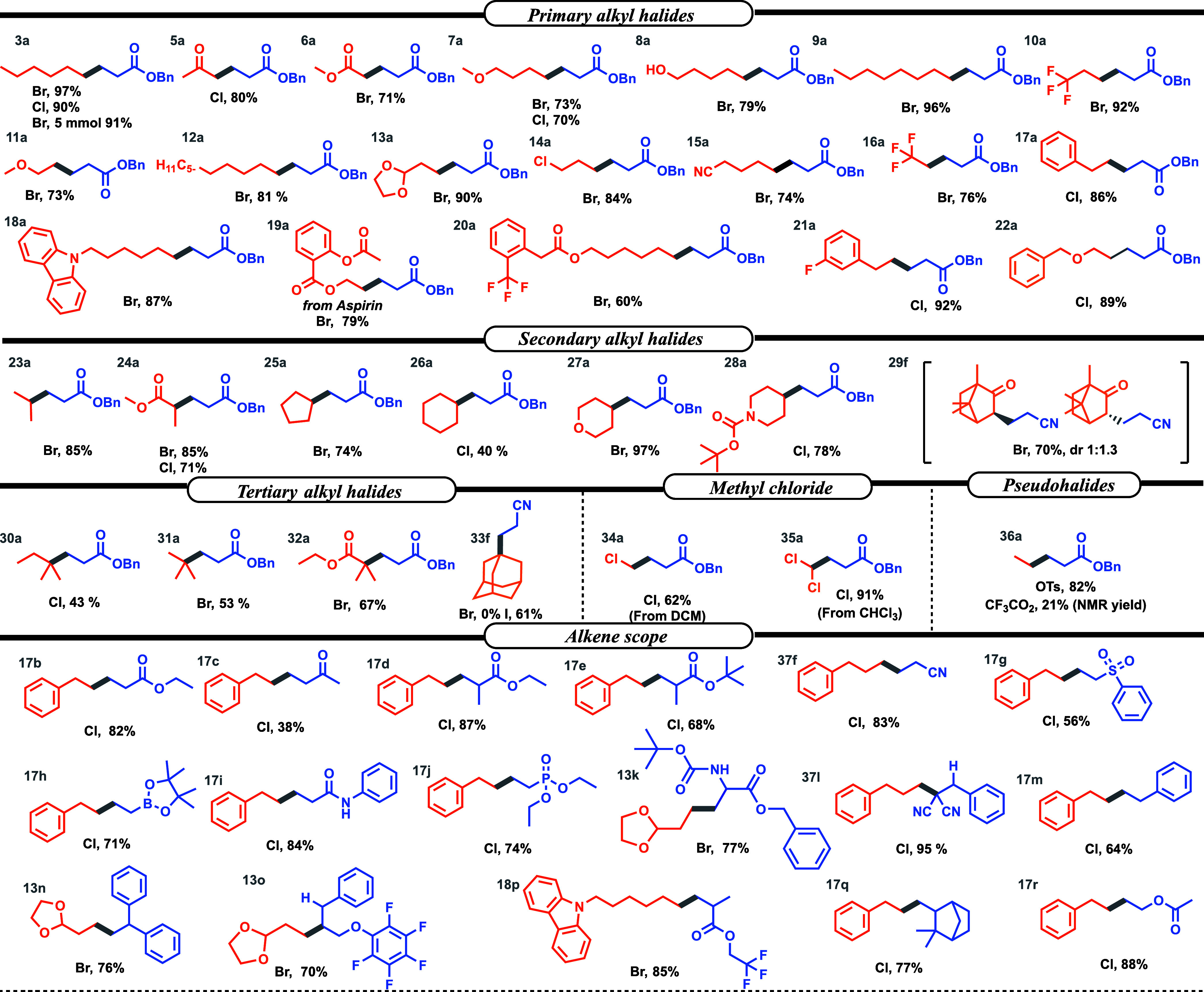
Substrate Scope^a^

a Yields are isolated. For alkyl bromides **4l** salt was used and for the rest **4f** was used.

When 1,3-bromochloropropane was used, perfect selectivity for the bromide was observed (**14a**). Not surprisingly, nitriles (**15a**) were well tolerated, as well as arenes (**17a**, **20a**, **21a**, **22a**)—including one derived from aspirin tablets (**19a**). A direct comparison of the chloride and bromide (**3a**) showed that both gave similar yields and that scaling to 5 mmol resulted in only a moderate reduction of yield.

Secondary bromides worked well (Table 2, **23a**, **24a**, **25a**, **27a, 29f**, 70–97% yield), while secondary chlorides gave comparably lower yields (**24a**, **26a**, **28a**, 40–78% yield)—presumably owing to the more challenging substitution.^40^ Cyclic alkyl bromides and chlorides were converted to alkylation products in moderate to excellent yields (**25a**–**29f**, 40–97% yield).

The MacMillan group also recently developed a method that generated alkyl radicals from the same chlorides.^4^ Their method required stoichiometric amounts of an expensive ($85,546/mol) supersilane that facilitated XAT to generate the radical. Significantly lowered operating costs is another enabling aspect of lutidinium cocatalysis in generating useful radical intermediates.^41^ Saturated heterocycles, such as tetrahydropyrans (**27a**) and piperidines (**28a**), which are prevalent in medicinal agents,^42^ was also readily employed. The radical formed from the bridging bicycloalkyl bromide derived from D-camphor produced diastereomers in a 1:1.3 ratio (**29f**). Tertiary alkyl bromides and chlorides were successfully reacted, albeit with lower yields (**30a**–**33f**) compared to secondary and primary halides. We attributed this to a much more challenging substitution to the corresponding iodide—which has been reported to be slow.^43^ To probe this question, we examined bromo- and iodo-adamantane^44^ (**33f**), which, due to its bridging bicyclic nature, was not expected to be easily substituted via either the S_N_1 or S_N_2 mechanism.^45^ In the case of bromoadamantane, we were unable to detect the product at all, but when iodoadamantane was used, the product was obtained in a 61% yield—indicating that a difficult substitution will have a detrimental impact on the yield.

Next, we wanted to investigate C1 radicals. Interestingly, Zhang and Doyle recently studied the dehalogenation of Cl halogens (primarily iodides and bromides) by XAT using tertiary amines and CuI,^46^ but did not show the use of DCM and chloroform as substrates, suggesting that it might be difficult by their method. C1 radicals are expected to be the least stable radicals formed- based on corresponding bond disassociation energies.^46,47^ On the other hand, they should be readily substituted by lutidinium iodide. Indeed, when dichloromethane and chloroform were used as source of methyl radicals and made to react under these conditions, the benzyl chlorobutanoate (**34a**) and benzyl 4,4-dichlorobutanoate (**35a**) were formed in a great (62 and 91%) yield. The fact that the product, which itself is a primary chloride, was not consumed maybe due to the enhanced rate of substitution of dichloromethane. Attempts using methyl chloride and methyl tosylate were unsuccessful, resulting in unintended and poorly characterized chemical reactions. We believe that this is due to the nature of the methyl radical rather than its ability to form.

Next, we extended our investigation to pseudohalides to demonstrate the versatility of this chemistry beyond halides. We replaced the alkyl halide with ethyl tosylate and ethyl trifluoroacetate without otherwise altering the conditions, indeed, both gave benzyl pentanoate in excellent (82%, **36a**, Table 2) to modest (21%) for the tosylates and trifluoroacetate, respectively. This result underscores the broader applicability of this methodology. Effectively, it allows polar alcohol derivatives—with strong C–O bonds which are not typically effective radical sources to serve as C-centered radical by in situ activation. This substantially extends the utility of lutidinium iodide catalysis.

Finally, we evaluated the scope of the alkene using phenethyl chloride, phenpropyl chloride a masked propionaldehyde bromide, and a carbazole-substituted bromide. A series of electron-deficient olefins were subjected to the standard conditions (Table 2), including acrylates (**17b**), vinyl ketones (**17c**), methacrylates (**17d**, **17e, 17r**), acrylonitrile (**37f**), vinyl sulfone (**17g**), vinyl boronic ester (**17h**), acrylamide (**17i**), vinyl phosphonate (**17j**) and dehydroalanine (**13k**)–all worked as expected to give the desired product in moderate to excellent yield. The ability to rapidly build up molecules from small, readily available fragments that include diverse functional groups increases synthetic access to more complex molecules- and is one reason the Giese reaction has remained so relevant. Of note, when benzene alkylidene malononitrile was used, we observed a complete switch in the regioselectivity- with formation of the benzylic radical rather than the α-malononitrile radical (**37l**).^48^ Supporting the latter idea, some styrene derivatives were investigated (**17m**, **13n**, **13o**, **18p, 17q**). All showed the same regioselectivity and gave good yields. Importantly, these substrates highlight the reaction’s preference to engage in single electron transfer rather than energy transfer catalysis- another well-established quenching mode for these types of catalysts.^48,49^

## Conclusions

We have developed a novel method for generating alkyl radicals from unactivated alkyl chlorides, bromides, tosylates, and even trifluoroacetates using catalytic amounts of *N*-alkyl lutidinium salts- which are readily produced on the decagram scale from inexpensive material (lutidine $23/mol). Furthermore, the method exhibited excellent functional group compatibility and efficiency from the alkene component and worked well for electron-poor alkenes as well as styrene-derived alkenes. Importantly, the method successfully converts primary, secondary, and even tertiary alkyl chlorides and bromides, albeit with decreased efficiency with increased substitution. Even so, we believe this to be a remarkable accomplishment that should enable chemists to readily use reagents from bottles to rapidly enhance synthetic complexity. Finally, in terms of conceptual advances, this reaction highlights exciting new roles for lutidinium salts in facilitating halogen atom transfer and radical formation.

## Supporting Information Available

Experimental procedures, tabulated experimental data, and compound characterization data, and FAIR data including the primary NMR FID files for compounds **4a**–**4y**, **3a**–**36a**, **17b**–**17r** (PDF)
Lutidinium project spectra (ZIP)
