# Supplementary material for: Co-Catalytic Coupling of Alkyl Halides and Alkenes: the Curious Role of Lutidine
Source: J Am Chem Soc. 2025 Feb 3;147(6):5238–46. doi: 10.1021/jacs.4c15812 (PMC11827002; doi:10.1021/jacs.4c15812)

Co-catalytic Coupling of Alkyl Halides and Alkenes; the Curious Role of Lutidine

Roshini Hanumanthu,※ Parul Sharma,※ Avery Ethridge, and Jimmie D. Weaver III*

Supporting Information

Corresponding author: jimmie.weaver@okstate.edu

Table of Contents

A. General Information S3
B. Photocatalytic Reaction Setup S4
C. Experimental Section S5
- C1: General Procedure A S5
- C2: Salts Preparation S5

D. Redox Active Salt Testing S11
E. Reaction Optimization S13
- E1 Optimization Tables of Alkyl Halides S13
- E3 Photocatalyst Screening S22
- E4 Solvent Screening S23
- E5 Control Studies S24

F. Mechanism Studies S25
- F1: Isotopic Labeling Experiment S25
- F2: Radical Probe Experiment S27
- F3: Electrochemical Measurements S29
- F4: Radical Scavenger Experiment S32
- F5: UV Studies S33
- F6: Generation of alkyl iodide S34
- F7: Stern-Volmer S36
- F8: Different amine than DIPEA S40
- F9:Reaction with Aryl-X S41
- F10:Reaction with Aryl-X and Alkyl-X S41

G. Synthesis of Substrates S43
H. Characterization of Products S44
- G1: Alkyl Halides Substrate Characterization S44
  - G1.1: Primary Halides S44
  - G1.2: Secondary Halides S51
  - G1.3: Tertiary Halides S54
  - G1.4: Methyl Radical S55
  - G1.4: Pseudohalides S56
- G2: Alkene Substrate Characterization S57

I. Photocatalytic reaction in large-scale S63
J. References S64

H.NMR Spectra S66

A. General Information

All chemicals were procured from commercial sources (Sigma-Aldrich, Oakwood Chemicals, Alfa Aesar, or Santa Cruz Biotechnology) and used without further purification unless specified otherwise. Acetonitrile (CH_3CN) underwent drying over molecular sieves, while Diisopropylethylamine (DIPEA) was distilled and stored over KOH pellets. The photocatalyst [4,4′-Bis(1,1-dimethylethyl)-2,2′-bipyridine-N1,N1′]bis[3,5-difluoro-2-[5-(trifluoromethyl)-2-pyridinyl-N]phenyl-C]Iridium(III) hexafluorophosphate, also known as Ir[dF(CF_3)ppy]$_2$(dtbbpy)PF_6, was synthesized following a literature procedure.[1]

Reactions were monitored through 1H NMR and GC-MS (Electron Impact, QP 2010S Shimadzu, equipped with an autosampler), GC-MS (Chemical Ionization, QP 2020NX Shimadzu, equipped with an autosampler). NMR spectra were acquired using either a 400 MHz Bruker Avance III spectrometer or a Bruker Neo 800 MHz spectrometer equipped with a TCI cryo-probe. Chemical shifts for ^{1}H and ^{13}C NMR are reported in ppm relative to the residual solvent peak.

Purifications were conducted using a Teledyne Isco Combiflash Rf 200i flash chromatograph with Sorbtech Rf normal-phase silica columns (4 g, 12 g, or 24 g) and product detection at 254 and 288 nm.

Substrate synthesis reactions were monitored via thin-layer chromatography (TLC) using Sorbent Technology silica XHL TLC plates with UV254, glass-backed 250 µm, and visualization with a handheld ultraviolet lamp. Absorption spectra were recorded on a Shimadzu UV-2600 UV-vis spectrometer. High-resolution mass spectrometry (HRMS) data were obtained with a Thermo Scientific Orbitrap Fusion Tribrid Mass Spectrometer, incorporating the quadrupole mass analyzer. Structural assignments were validated with additional information from gCOSY experiments.

B. Photocatalytic Reaction Setup

Photocatalytic reactions were carried out in a light bath which consist of high intensity blue LEDs (λ max emission ~ 450 nm) as described below. The blue LED strips (200 LEDs) were wrapped around the walls of a Pyrex crystallizing dish (capacity 1,200 mL, O.D. × H ~150 mm × 75 mm). A lid that rested on the top was fashioned from cardboard and holes were made such that NMR tubes were held firmly in the cardboard lid which was placed on the top of the bath. Water was added to the bath such that the tubes were submerged in the water, maintained at 70 ºC with a thermostat-controlled heating mantle. Lights are bought from Solid Apollo Blue Waterproof 5050 72 W Blue LED Strip Light. SKU: SA-LS-BL-5050-300-IP67-24V.

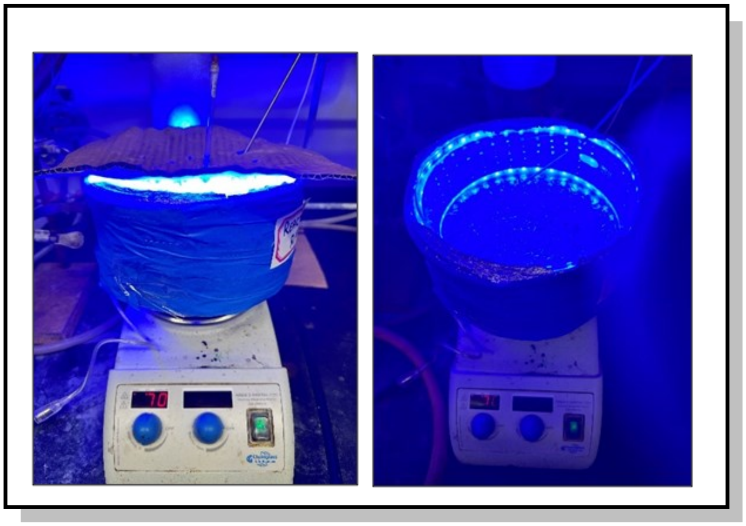

Figure S1. Reaction set up in the light bath.

C. Experimental Section

C1: General Procedure A. Drying Acetonitrile

Acetonitrile (CH_3CN) was initially dried over activated molecular sieves. The procedure reported by Lawton was followed to further dry the solvent.[2] Initially, a preliminary drying was performed with activated potassium carbonate. The mixture was gently stirred for 24 h, then it was decanted onto phosphorus pentoxide and stirred at reflux for 2 h. Further, a fractional distillation was done. The first fraction at a distillation temperature of 80-82 °C gave the dry acetonitrile which was then stored on activated molecular sieves.

C2: Salts Preparation

General Procedure B.

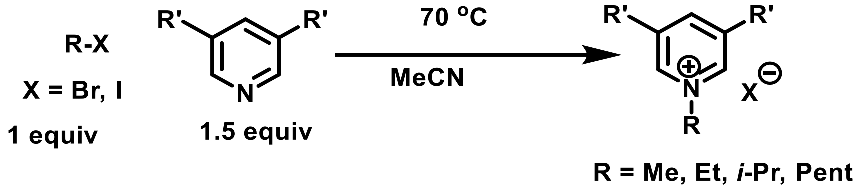

The commercially available pyridine or lutidine (1.5 equiv, 15 mmol) was added to the 25 mL round-bottomed flask along with alkyl bromide or iodide (1 equiv, 10 mmol). The mixture was gently stirred at 60 or 70 °C. After consumption of the starting material, the reaction mixture was cooled in the ice bath, washed twice with hexane, and filtered. The salts obtained were subjected to a high vacuum to remove any volatile components and their purity confirmed using ^{1}H and ^{13}C NMR. The salts were stored in a screw top vial at 4 °C . They were further used in the reaction without any additional purification.

[4a] isopropyl-3,5-dimethylpyridin-1-ium bromide

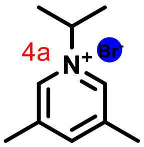

General procedure B was followed using 3,5-lutidine (1.5 equiv, 15 mmol, 1607 mg, 1711 µL) and 2-bromopropyl (1.0 equiv, 10 mmol, 1229 mg, 938 µL) for 2 h to afford isopropyl-3,5-dimethylpyridin-1-ium bromide. Yield: 2.1 g, 93%.

^{1}H NMR: (400 MHz, $CDCl_3$) δ 9.32 (d, J = 1.8 Hz, 2H), 7.97 (s, 1H), 5.40 (hept, J = 6.7 Hz, 1H), 2.58 (s, 6H), 1.72 (d, J = 6.8 Hz, 6H).

^{13}C NMR: (201 MHz, $CDCl_3$) δ 146.4, 140.1, 139.2, 64.7, 23.4, 18.6.

.

[4b] 1-ethyl pyridinium bromide

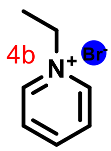

General procedure B was followed, using pyridine (1.5 equiv, 15 mmol, 1186 mg, 1219 µL) and 1-bromoethane (1.0 equiv, 10 mmol, 1089 mg, 746 µL) for 1 h to afford 1-ethyl pyridinium bromide. Yield: 1.7 g, 95%.

^{1}H NMR: (800 MHz, $CDCl_3$) δ 9.66 – 9.63 (m, 2H), 8.54 (tt, J = 7.8, 1.4 Hz, 1H), 8.16 (t, J = 7.0 Hz, 2H), 5.12 (q, J = 7.4 Hz, 2H), 1.75 (t, J = 7.4 Hz, 3H).

^{13}C NMR: (201 MHz, $CDCl_3$) δ 145.1, 145.0, 128.5, 57.5, 17.4.

[4c] 1-ethyl lutidinium bromide

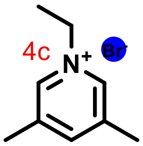

General procedure B was followed using 3,5-lutidine (1.5 equiv, 15 mmol, 1607 mg, 1711 μL) and 1-bromoethane (1.0 equiv, 10 mmol, 1089 mg, 746 μL) for 1 h to afford 1-ethyl lutidinium bromide. Yield: 1.9 g, 93%.

^{1}H NMR: (800 MHz, $CDCl_3$) δ 9.28 (s, 2H), 8.00 (s, 1H), 5.00 (q, *J* = 7.4 Hz, 2H), 2.62 (s, 6H), 1.73 (t, *J* = 7.4 Hz, 3H).

^{13}C NMR: (201 MHz, $CDCl_3$) δ 146.5, 141.4, 139.0, 57.0, 18.6, 17.3.

[4d] 1-isopropylpyridi-1-ium bromide

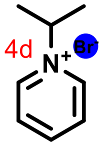

General procedure B was followed using pyridine (1.5 equiv, 15 mmol, 1186 mg, 1219 μL) and 2-bromopropane (1.0 equiv, 10 mmol, 1229 mg, 938 μL) for 2 h to afford 1-isopropylpyridi-1-ium bromide. Yield: 1.6 g, 80%.

^{1}H NMR: (800 MHz, D_2O) δ 8.96 (s, 2H), 8.56 (s, 1H), 8.10 (s, 2H), 5.03 (m, 1H), 1.71 (s, 6H).

^{13}C NMR: (201 MHz, D_2O) δ 145.7, 142.4, 128.3, 65.2, 22.2.

[4e] 3,5-dimethyl-1-nonylpyridin-1-ium bromide

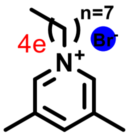

General procedure B was followed using 3,5-lutidine (1.5 equiv, 15 mmol, 1607 mg, 1711 μL) and 1-bromononane (1.0 equiv, 10 mmol, 2071 mg, 1910 μL) for 2 h to afford 3,5-dimethyl-1-nonylpyridin-1-ium bromide. Yield: 2.6 g, 84%.

^{1}H NMR: (800 MHz, $CDCl_3$) δ 9.13 – 9.05 (m, 2H), 7.96 – 7.88 (m, 1H), 4.74 – 4.65 (m, 2H), 2.45 (dd, *J* = 13.6, 4.8 Hz, 6H), 1.89 (dt, *J* = 15.7, 7.2 Hz, 2H), 1.27 – 1.00 (m, 10H), 0.73 – 0.63 (m, 3H).

^{13}C NMR: (201 MHz, $CDCl_3$) δ 146.2, 141.7, 138.8, 61.4, 31.8, 31.7, 31.6, 28.9, 28.9, 26.0, 22.4, 18.4, 14.0.

[4f] 1-ethyl lutidinium iodide

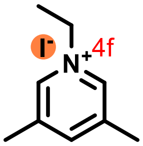

General procedure B was followed using 3,5-lutidine (1.5 equiv, 15 mmol, 1607 mg, 1711 μL) and 1-iodoethane (1.0 equiv, 10 mmol, 1559 mg, 800 μL) for 1 h to afford 1-ethyl lutidinium iodide. Yield: 2.3 g, 89 %.

^{1}H NMR: (800 MHz, $CDCl_3$) δ 9.07 (d, *J* = 17.1 Hz, 2H), 8.04 – 8.00 (m, 1H), 4.94 (q, *J* = 7.4 Hz, 2H), 2.63 (s, 6H), 1.76 – 1.73 (m, 3H).

^{13}C NMR: (201 MHz, $CDCl_3$) δ 146.5, 141.4, 139.0, 57.0, 18.6, 17.3.

HRMS (ESI) m/z: [M] + calcd for $C_9H_{14}IN$ 263.0171; found 263.0172.

[4g] 1-ethyl pyridinium iodide

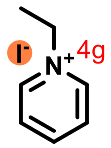

General procedure B was followed using pyridine (1.5 equiv, 15 mmol, 1186 mg, 1219 µL) and 1-iodoethane (1.0 equiv, 10 mmol, 1559 mg, 800 µL) for 1 h to afford 1-ethyl pyridinium iodide. Yield: 2.1 g, 90 %.

^{1}H NMR: (800 MHz, $CDCl_3$) δ 9.44 (d, *J* = 5.9 Hz, 2H), 8.55 (td, *J* = 7.7, 1.4 Hz, 1H), 8.16 (t, *J* = 7.0 Hz, 2H), 5.08 (q, *J* = 7.4 Hz, 2H), 1.78 (t, *J* = 7.4 Hz, 3H).

^{13}C NMR: (201 MHz, $CDCl_3$) δ 145.3, 144.7, 128.6, 57.9, 17.2.

[4h] methyl-3,5-dimethylpyridin-1-ium iodide

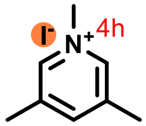

General procedure B was followed using 3,5-lutidine (1.5 equiv, 15 mmol, 1607 mg, 1711 µL) and methyl iodide (1.0 equiv, 10 mmol, 1419 mg, 622 µL) for 2 h to afford methyl-3,5-dimethylpyridin-1-ium iodide. Yield: 2.3 g, 94%.

^{1}H NMR: (800 MHz, $CDCl_3$) δ 9.02 (d, *J* = 1.8 Hz, 2H), 8.04 (s, 1H), 4.54 (s, 3H), 2.54 (s, 6H).

^{13}C NMR: (201 MHz, $CDCl_3$) δ 146.5, 142.4, 138.7, 48.7, 18.5.

HRMS (ESI) m/z: [M] + calcd for $C_8H_{12}IN$ 249.0014; found 249.0016.

[4i] methyl lutidinium iodide

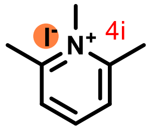

General procedure B was followed using 2,6-lutidine (1.5 equiv, 15 mmol, 1607 mg, 1711 µL) and methyl iodide (1.0 equiv, 10 mmol, 1419 mg, 622 µL) for 2 h to afford methyl lutidinium iodide. Yield: 2.2 g, 89%.

^{1}H NMR: (400 MHz, D_2O) δ 8.11 (t, *J* = 7.9 Hz, 1H), 7.65 (d, *J* = 8.0 Hz, 2H), 4.00 (s, 3H), 2.72 (s, 6H).

^{13}C NMR: (101 MHz, D_2O) δ 155.9, 143.9, 127.1, 39.9, 21.1.

[4j] 1-isopropyl pyridinium iodide

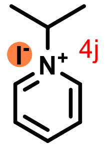

General procedure B was followed using pyridine (1.5 equiv, 15 mmol, 1186 mg, 1219 µL) and 2-iodopropane (1.0 equiv, 10 mmol, 1699 mg, 998 µL) for 1 h to afford 1-isopropyl pyridinium iodide. Yield: 2.0 g, 83%.

^{1}H NMR: (800 MHz, $CDCl_3$) δ 9.55 (dt, *J* = 6.7, 2.2 Hz, 2H), 8.60 (ddt, *J* = 9.6, 7.8, 1.7 Hz, 1H), 8.23 (t, *J* = 6.9 Hz, 2H), 5.57 – 5.51 (m, 1H), 1.83 (dd, *J* = 6.9, 1.8 Hz, 6H).

^{13}C NMR: (201 MHz, $CDCl_3$) δ 145.6, 143.3, 128.9, 65.3, 23.6.

[4k] 1-pentyl lutidinium iodide

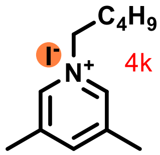

General procedure B was followed using 3,5-lutidine (1.5 equiv, 15 mmol, 1607 mg, 1711 µL) and 1-iodopentane (1.0 equiv, 10 mmol, 1980 mg, 1305 µL) for 2 h to afford 1-pentyl lutidinium iodide. Yield: 2.7 g, 90%.

^{1}H NMR: (400 MHz, $CDCl_3$) δ 9.13 (d, *J* = 3.2 Hz, 2H), 8.05 (s, 1H), 4.83 (t, *J* = 7.5 Hz, 2H), 2.60 (d, *J* = 3.0 Hz, 6H), 2.06 (tq, *J* = 11.4, 5.7 Hz, 2H), 1.44 – 1.32 (m, 4H), 0.89 (ddt, *J* = 7.7, 5.7, 2.3 Hz, 3H).

^{13}C NMR: (101 MHz, $CDCl_3$) δ 146.5, 141.6, 138.9, 61.5, 31.5, 28.1, 22.1, 18.6, 13.9.

HRMS (ESI) m/z: [M] + calcd for $C_{12}H_{20}IN$ 305.0640; found 305.0641.

[4l] 1-isopropyl-lutidinium iodide

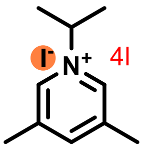

General procedure B was followed, using 3,5-lutidine (1.5 equiv, 15 mmol, 1607 mg, 1711 µL) and 2-iodopropane (1.0 equiv, 10 mmol, 1699 mg, 998 µL) for 1 h to afford pyridinium iodide. Yield: 2.6 g, 97 %.

^{1}H NMR: (800 MHz, $CDCl_3$) δ 9.16 – 9.12 (m, 2H), 8.03 (d, *J* = 5.3 Hz, 1H), 5.46 (m, 1H), 2.66 (s, 6H), 1.81 (d, *J* = 6.8 Hz, 6H).

^{13}C NMR: (201 MHz, $CDCl_3$) δ 146.7, 139.6, 139.3, 64.7, 23.5, 18.8.

HRMS (ESI) m/z: [M] + calcd for $C_{10}H_{16}IN$ 277.0327; found 277.0329.

[4m] propyl-3,5-dimethylpyridin-1-ium iodide

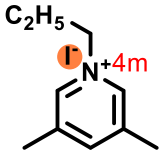

General procedure B was followed using 3,5-lutidine (1.5 equiv, 15 mmol, 1607 mg, 1711 µL) and propyl iodide (1.0 equiv, 10 mmol, 1699 mg, 998 µL) for 2 h to afford propyl-3,5-dimethylpyridin-1-ium iodide. Yield: 2.4 g, 89%.

^{1}H NMR: (800 MHz, $CDCl_3$) δ 9.17 (d, *J* = 1.8 Hz, 2H), 8.06 (s, 1H), 4.79 (t, *J* = 7.5 Hz, 2H), 2.59 (s, 6H), 2.10 (h, *J* = 7.4 Hz, 2H), 1.01 (t, *J* = 7.4 Hz, 3H).

^{13}C NMR: (201 MHz, $CDCl_3$) δ 146.6, 141.6, 138.8, 62.5, 25.2, 18.5, 10.6.

HRMS (ESI) m/z: [M] + calcd for $C_{10}H_{16}IN$ 277.0327.; found 277.0328.

[4n] 1-isopropyl-4-methyl pyridinium iodide

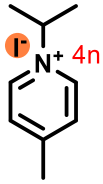

General procedure B was followed using 4-methylpyridine (1.5 equiv, 15 mmol, 1396 mg, 1459 µL) and 2-iodopropane (1.0 equiv, 10 mmol, 1699 mg, 998 µL) for 2 h to afford 1-isopropyl-4-methyl pyridinium iodide. Yield: 2.3 g, 90%.

^{1}H NMR: (400 MHz, $CDCl_3$) δ 9.31 – 9.23 (m, 2H), 7.88 (4 Hz, 2H), 5.36 (hept, *J* = 6.7 Hz, 1H), 2.61 (s, 3H), 1.71 (d, *J* = 6.7 Hz, 6H).

^{13}C NMR: (101 MHz, $CDCl_3$) δ 159.2, 142.3, 129.3, 64.3, 23.5, 22.3.

[4o] 1-isopropyl-4-(tert-butyl) pyridinium iodide

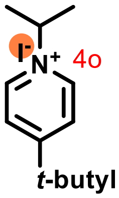

General procedure B was followed using 4-(tert-butyl) pyridine (1.5 equiv, 15 mmol, 2028 mg, 2197 µL) and 2-iodopropane (1.0 equiv, 10 mmol, 1699 mg, 998 µL) for 3 h to afford 1-isopropyl-4-(tert-butyl) pyridinium iodide. Yield: 2.9 g, 96%.

^{1}H NMR: (800 MHz, $CDCl_3$) δ 9.49 – 9.46 (m, 2H), 8.10 – 8.06 (m, 2H), 5.50 (hept, *J* = 6.8 Hz, 1H), 1.80 (d, *J* = 6.8 Hz, 6H), 1.44 (s, 9H).

^{13}C NMR: (201 MHz, $CDCl_3$) δ 171.2, 142.6, 125.7, 64.1, 36.7, 30.1, 23.5.

[4p] 1-(1-methoxy-1-oxopropan-2-yl)-3,5-dimethylpyridin-1-ium bromide

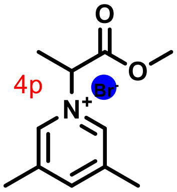
4p

General procedure B was followed using 3,5-lutidine (1.5 equiv, 15 mmol, 1607 mg, 1711 µL) and methyl 2-bromopropanoate (1.0 equiv, 10 mmol, 1670 mg, 1115 µL) for 2 h to afford 1-(1-methoxy-1-oxopropan-2-yl)-3,5-dimethylpyridin-1-ium bromide. Yield: 2.0 g, 75%.

^{1}H NMR: (800 MHz, D_2O) δ 8.52 (s, 2H), 8.22 (d, *J* = 2.0 Hz, 1H), 5.59 (qd, *J* = 7.4, 1.5 Hz, 1H), 3.75 (d, *J* = 1.1 Hz, 3H), 2.45 (s, 6H), 1.89 (dd, *J* = 7.3, 1.3 Hz, 3H).

^{13}C NMR: (201 MHz, D_2O) δ 169.9, 148.1, 140.9, 139.2, 67.9, 54.0, 17.5, 17.0.

[4q] 1-methylcyclopropane lutidinium bromide

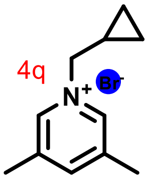
4q

General procedure B was followed using 3,5-lutidine (1.5 equiv, 15 mmol, 1607 mg, 1711 µL) and (bromomethyl)cyclopropane (1.0 equiv, 10 mmol, 1350 mg, 969 µL) for 2 h to afford 1-methylcyclopropane lutidinium bromide. Yield: 2.0 g, 85%.

^{1}H NMR: (800 MHz, $CDCl_3$) δ 9.25 (d, *J* = 1.9 Hz, 2H), 8.01 (s, 1H), 4.76 (d, *J* = 7.7 Hz, 2H), 2.59 (s, 6H), 1.53 (tt, *J* = 7.8, 4.8 Hz, 1H), 0.75 (dt, *J* = 6.4, 4.6 Hz, 2H), 0.72 – 0.67 (m, 2H).

^{13}C NMR: (201 MHz, $CDCl_3$) δ 146.2, 141.6, 138.8, 65.2, 18.6, 12.5, 4.8.

[4r] 1-(2,2-dimethoxyethyl)-3,5-dimethylpyridin-1-ium bromide

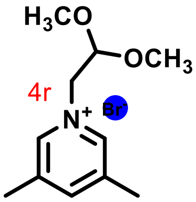
4r

General procedure B was followed using 3,5-lutidine (1.5 equiv, 15 mmol, 1607 mg, 1711 µL) and 2-bromo-1,1-dimethoxyethane (1.0 equiv, 10 mmol, 1690 mg, 1181 µL) for 2 h to afford 1-(2,2-dimethoxyethyl)-3,5-dimethylpyridin-1-ium bromide. Yield: 1.6 g, 60%.

^{1}H NMR: (400 MHz, $CDCl_3$) δ 9.01 (s, 2H), 8.07 (s, 1H), 5.00 (d, *J* = 3.8 Hz, 2H), 4.83 (t, *J* = 4.0 Hz, 1H), 3.45 (s, 6H), 2.53 (d, *J* = 2.5 Hz, 6H).

^{13}C NMR: (101 MHz, $CDCl_3$) δ 146.6, 143.1, 137.9, 101.6, 61.6, 56.4, 18.4.

[4s] 1-(3-chloropropyl)-3,5-dimethylpyridin-1-ium bromide

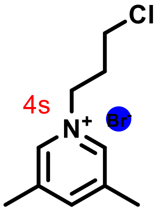
4s

General procedure B was followed using 3,5-lutidine (1.5 equiv, 15 mmol, 1607 mg, 1711 µL) and 1-bromo-2-chloroethane (1.0 equiv, 10 mmol, 1434 g, 832 µL) for 2 h to afford 1-(3-chloropropyl)-3,5-dimethylpyridin-1-ium bromide. Yield: 2.0 g, 77%.

^{1}H NMR: (400 MHz, D_2O) δ 8.58 – 8.48 (m, 2H), 8.18 (d, *J* = 7.3 Hz, 1H), 4.65 (q, *J* = 6.6 Hz, 2H), 3.59 (dt, *J* = 9.2, 4.2 Hz, 1H), 2.85 – 2.26 (m, 9H).

^{13}C NMR: (101 MHz, $CDCl_3$) δ 146.6, 141.9, 139.1, 58.5, 41.0, 33.9, 18.4 (one extra C signal at 57).

[4t] 1-(2-(1,3-dioxolan-2-yl) ethyl)-3,5-dimethylpyridin-1-ium bromide

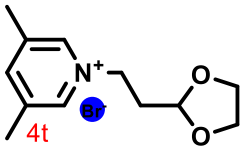
4t

General procedure B was followed using 3,5-lutidine (1.5 equiv, 15 mmol, 1607 mg, 1711 µL) and 2-(2-bromoethyl)-1,3-dioxolane (1.0 equiv, 10 mmol, 1810 mg, 1173 µL) for 2 h to afford 1-(2-(1,3-dioxolan-2-yl) ethyl)-3,5-dimethylpyridin-1-ium bromide. Yield: 2.3 g, 83%.

^{1}H NMR: (800 MHz, D_2O) δ 8.47 (s, 2H), 8.13 (s, 1H), 5.01 – 4.97 (m, 1H), 4.59 (td, *J* = 6.9, 1.9 Hz, 2H), 3.93 – 3.86 (m, 2H), 3.86 – 3.81 (m, 2H), 2.42 (d, *J* = 1.9 Hz, 6H), 2.37 (td, *J* = 6.7, 3.9 Hz, 2H).

^{13}C NMR: (201 MHz, D_2O) δ 146.8, 141.2, 138.9, 101.1, 64.8, 56.1, 33.1, 17.4.

[4u] 1-(2-(1,3-dioxoisoindolin-2-yl) ethyl)-3,5-dimethylpyridin-1-ium bromide

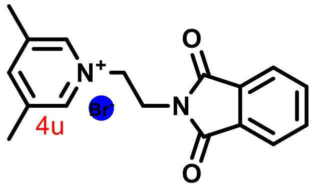

General procedure B was followed using 3,5-lutidine (1.5 equiv, 15 mmol, 1607 mg, 1711 µL) and 2-(2-bromoethyl) isoindoline-1,3-dione (1.0 equiv, 10 mmol, 2540 mg) for 2 h to afford 1-(2-(1,3-dioxoisoindolin-2-yl) ethyl)-3,5-dimethylpyridin-1-ium bromide. Yield: 3.2 g, 89%.

^{1}H NMR: (800 MHz, D_2O) δ 8.49 (s, 2H), 8.15 (s, 1H), 7.75 (tdd, *J* = 8.9, 4.5, 2.2 Hz, 4H), 4.72 (d, *J* = 5.6 Hz, 2H), 4.19 (t, *J* = 5.6 Hz, 2H), 2.34 (s, 6H).

^{13}C NMR: (201 MHz, D_2O) δ 169.6, 147.3, 141.3, 139.5, 135.2, 130.6, 123.7, 59.5, 38.2, 17.2.

[4v] 4,4'-di-tert-butyl-1,1'-dimethyl-[2,2'-bipyridine]-1,1'-diium iodide

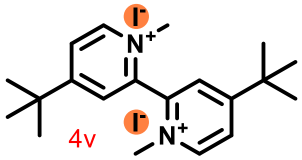

General procedure B was followed using 4,4′-Di-tert-butyl-2,2′-dipyridyl (1.5 equiv, 15 mmol, 2684 mg) and methyl iodide (1.0 equiv, 10 mmol, 1419 mg, 622 µL) for 2 h to afford 4,4'-di-tert-butyl-1,1'-dimethyl-[2,2'-bipyridine]-1,1'-diium iodide. Yield: 4.8 g, 88%.

^{1}H NMR: (400 MHz, DMSO) δ 9.30 (d, *J* = 6.6 Hz, 2H), 8.64 (d, *J* = 2.9 Hz, 2H), 8.50 (dd, *J* = 6.7, 2.4 Hz, 2H), 4.11 (s, 6H), 1.41 (s, 18H).

^{13}C NMR: (101 MHz, DMSO) δ 171.4, 148.4, 143.3, 128.3, 127.1, 47.1, 37.2, 30.0.

[4w] methyl 2,2'-bipyridine iodide

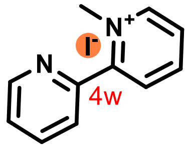

General procedure B was followed using 2,2'-bipyridine (1.5 equiv, 15 mmol, 1560 mg) and methyl iodide (1.0 equiv, 10 mmol, 1419 mg, 622 µL) for 2 h to afford methyl 2,2'-bipyridine iodide. Yield: 2.7 g, 93%.

^{1}H NMR: (800 MHz, $CDCl_3$) δ 9.61 (dd, *J* = 6.8, 3.4 Hz, 1H), 8.82 (dq, *J* = 4.4, 1.4 Hz, 1H), 8.65 (qd, *J* = 7.6, 4.9 Hz, 1H), 8.26 (tdt, *J* = 6.6, 3.3, 1.4 Hz, 1H), 8.16 (dt, *J* = 7.8, 1.1 Hz, 1H), 8.10 (ddt, *J* = 8.3, 4.0, 1.8 Hz, 1H), 8.06 (td, *J* = 7.8, 1.8 Hz, 1H), 7.59 (ddt, *J* = 7.5, 4.9, 1.2 Hz, 1H), 4.55 (s, 3H).

^{13}C NMR: (201 MHz, $CDCl_3$) δ 153.1, 150.0, 149.7, 147.9, 145.7, 138.2, 129.9, 128.0, 126.8, 125.9, 48.6.

[4x] 4-carbamoyl-1-methylpyridin-1-ium iodide

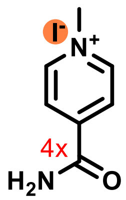

General procedure B was followed using isonicotinamide (1.5 equiv, 15 mmol, 1831 mg) and methyl iodide (1.0 equiv, 10 mmol, 1419 mg, 622 µL) for 2 h to afford 4-carbamoyl-1-methylpyridin-1-ium iodide. Yield: 2.0 g, 79%.

^{1}H NMR: (800 MHz, D_2O) δ 9.00 (d, *J* = 6.3 Hz, 2H), 8.37 (d, *J* = 6.3 Hz, 2H), 4.49 (s, 3H).

^{13}C NMR: (201 MHz, D_2O) δ 166.9, 149.6, 148.2, 146.3, 126.1, 48.5.

[4y] 1-pentyl lutidinium chloride

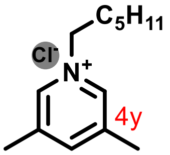

General procedure B was followed using 3,5-lutidine (1.5 equiv, 15 mmol, 1607 mg, 1711 µL) and 1-chlorohexane (1.0 equiv, 10 mmol, 1206 mg, 1370 µL) for 2 h to afford 1-pentyl lutidinium iodide. Yield: 2.4 g, 89%.

^{1}H NMR: ^{1}H NMR (400 MHz, $CDCl_3$) δ 9.22 (d, J = 4.3 Hz, 1H), 7.89 (d, J = 8.6 Hz, 1H), 7.78 (d, J = 6.9 Hz, 1H), 4.60 (q, J = 8.4 Hz, 2H), 2.50 – 2.20 (m, 6H), 2.10 – 1.94 (m, 3H), 1.76 (h, J = 10.2 Hz, 2H), 1.11 – 1.02 (m, 2H), 1.02 – 0.88 (m, 2H), 0.68 – 0.51 (m, 2H), 0.51 – 0.46 (m, 1H).

^{13}C NMR: ^{13}C NMR (101 MHz, $CDCl_3$) δ 146.75, 145.78, 141.95, 138.51, 132.41, 77.83, 77.51, 77.18, 61.02, 31.70, 30.90, 25.45, 22.06, 18.18, 13.68.

D. Redox Active Salt Testing

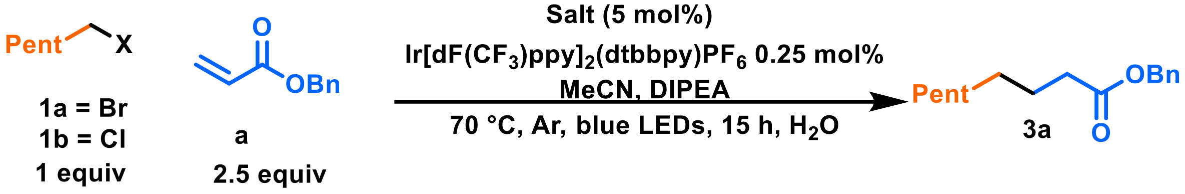

A diverse library of bromide and iodide salts were prepared using substituted pyridines following general procedure B. Then, the salt library was screened using the reactions shown above involving the hexyl bromide and chloride. After 15 h, the reaction mixture was cooled and the lights turned off. The yield was measured in the crude reaction mixture, using NMR using 1,3-benzodioxole as an internal standard.

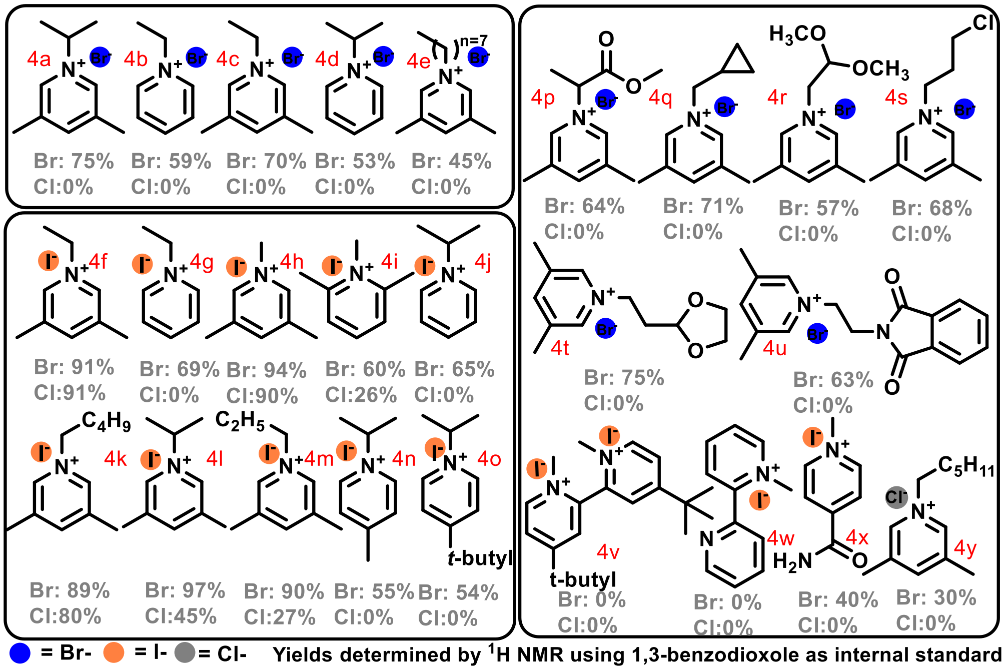

The impact of salt loading on yield was determined via GCMS of the reaction mixture after workup. The values reflect the integration of the TIC. The highest yield was observed using 5 mol% salt, we have optimized the concentration of salt using GCMS.

Table S1: Salt loading

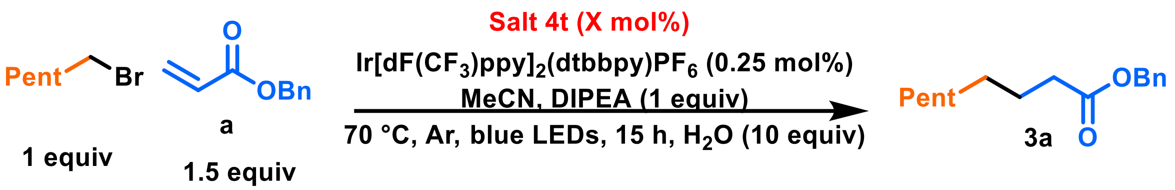

Entry	4t salt mol%	a, TIC 162	3a, TIC 248 (Pdt)	TIC 292 (DIPEA-2a adduct)
**1**	5	13	79	6.89
**2**	10	54	41	4.39
**3**	20	61	38	0.5
**4**	30	68	29	ND
**5**	70	84	15	ND

E. Reaction Optimization

During the course of our optimization experiments, three side products, 4, 5 and 6 were observed. These products were isolated and characterized. We assessed the impact of reaction conditions on their formation.

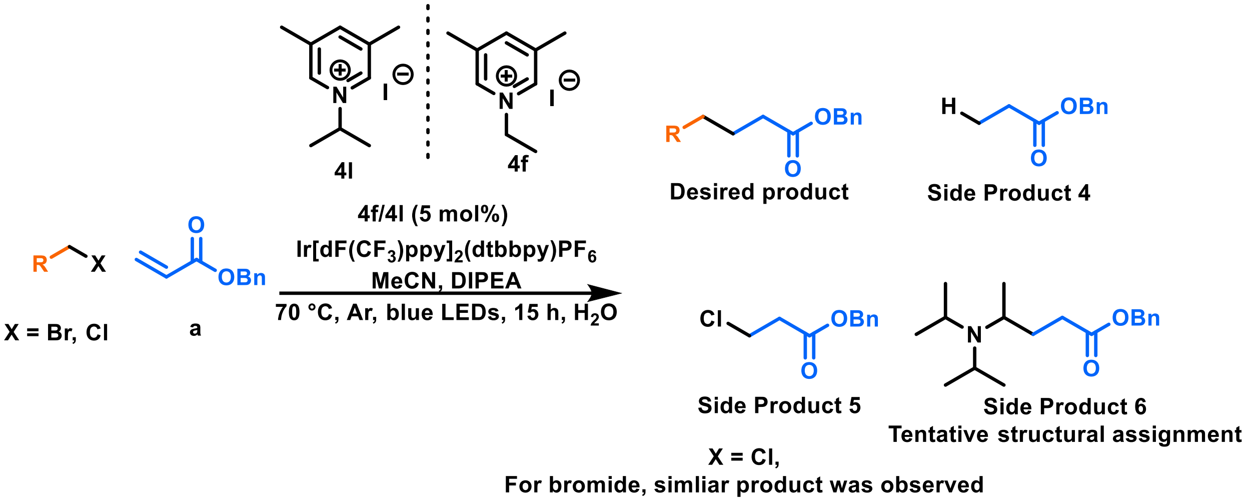

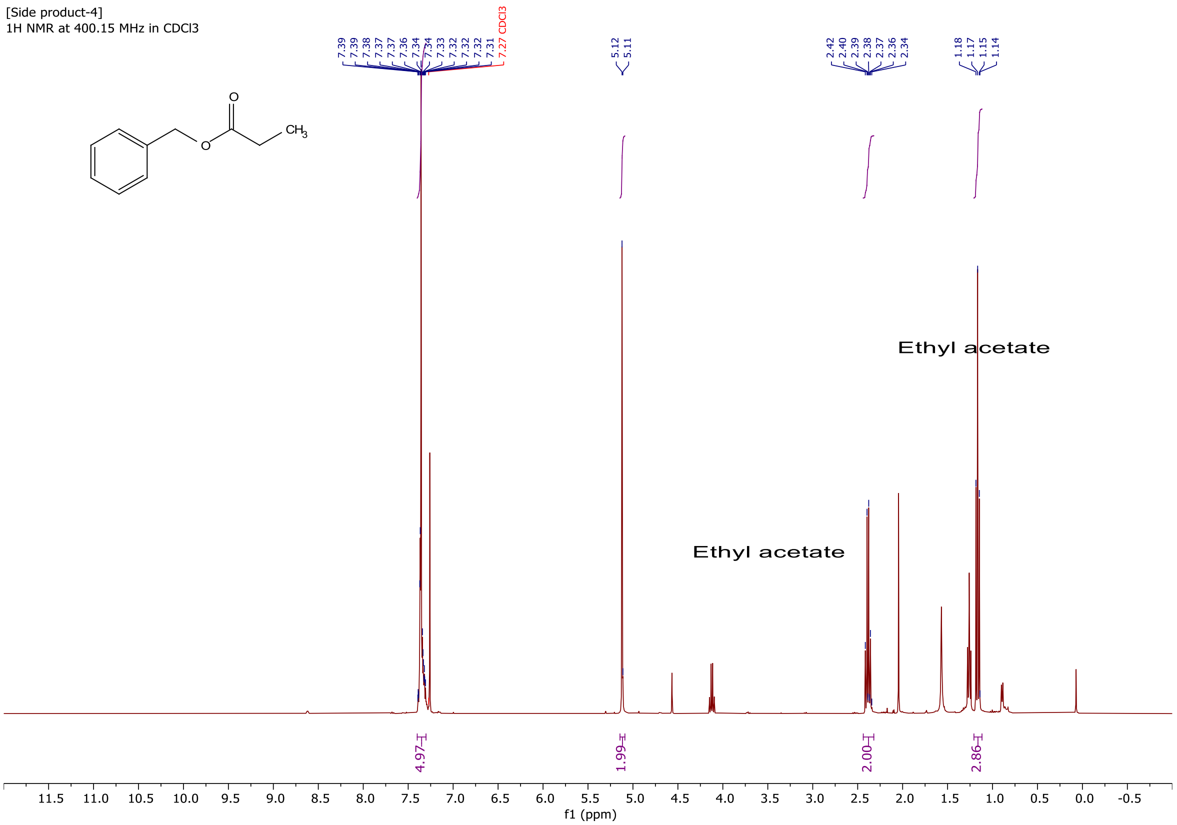

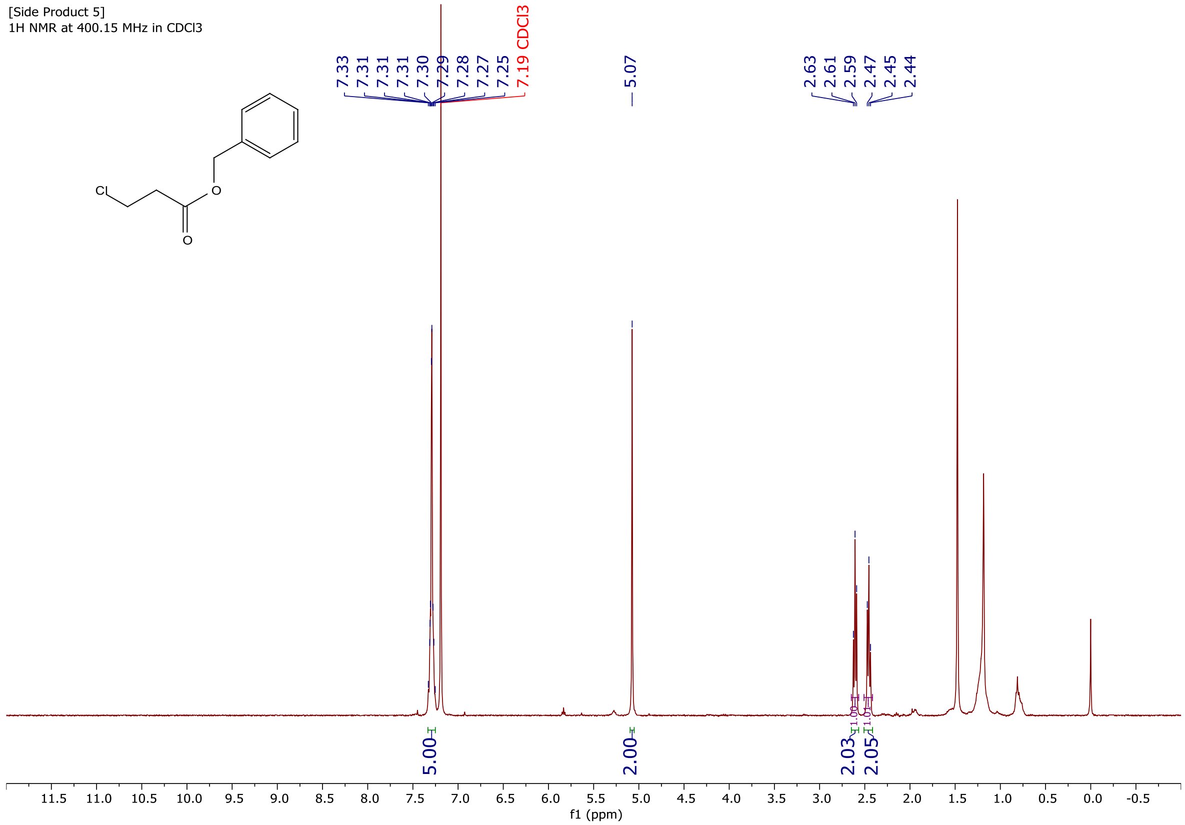

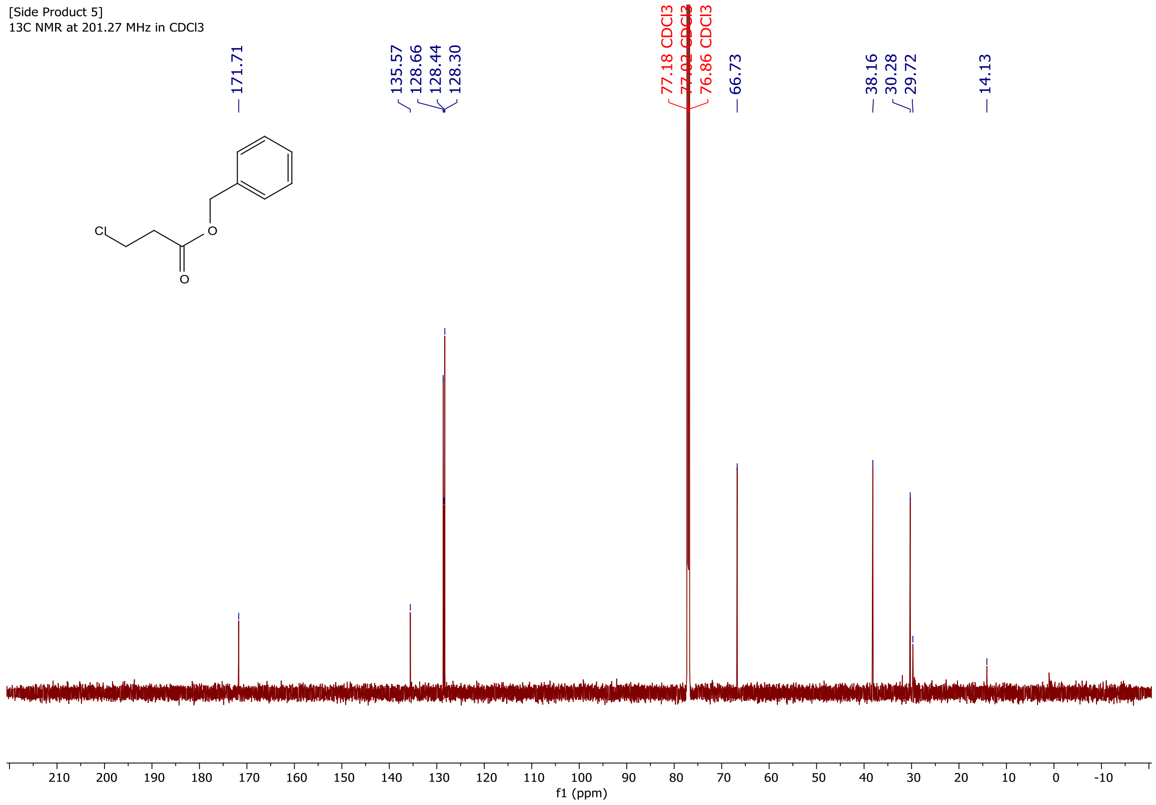

NMR chemical shifts match with the literature value.[3]

GCMS (CI) of side product 6

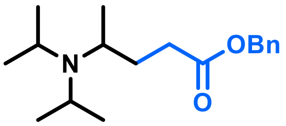

Exact Mass: 291.2198

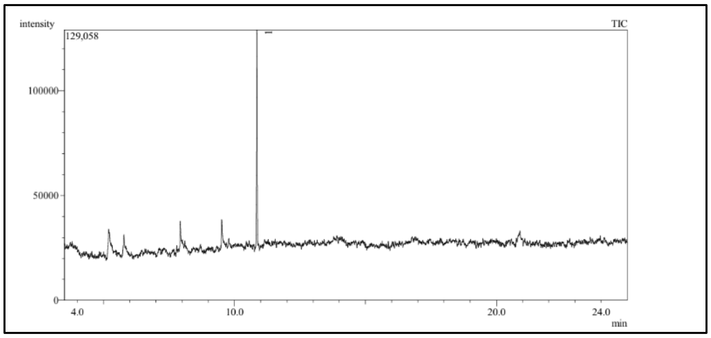

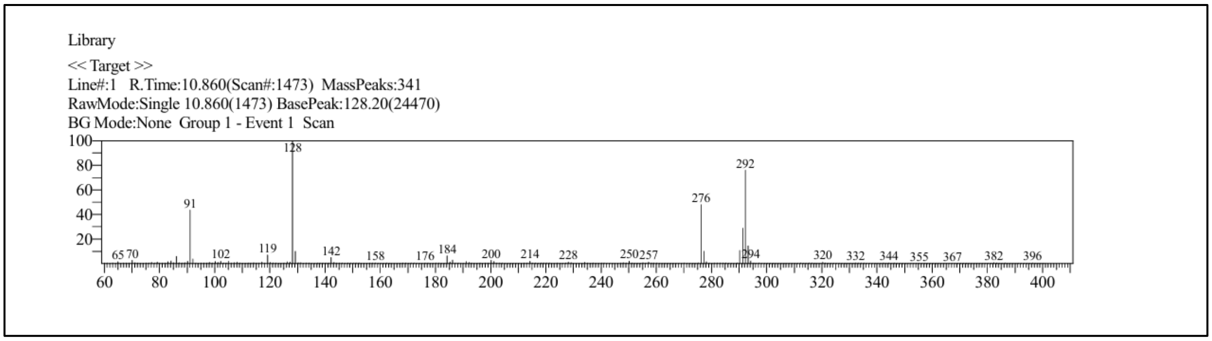

E1: Optimization Tables of alkyl halides

Table S2. Optimization of reaction conditions for primary bromide

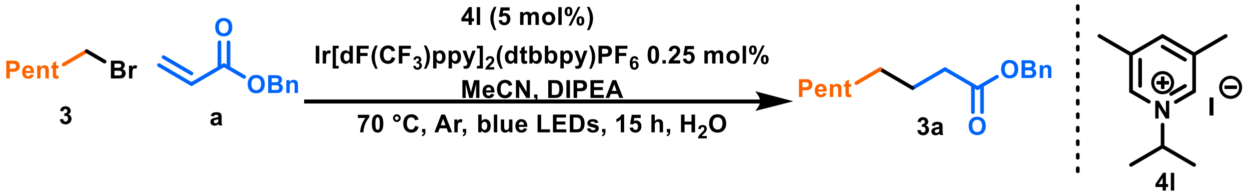

Entry	1 (equiv)	2 (equiv)	Salt (4l) (mol%)	DIPEA (equiv)	H_2O (equiv)	Time (h)	Yield of 3a%[a]
1	1.0	0.5	5.0	1.0	10	15	20
2	1.0	1.0	5.0	1.0	10	15	25
3	1.0	1.5	5.0	1.0	10	15	50

4	1.0	2.0	5.0	1.0	10	15	41
5	1.0	3.0	5.0	1.0	10	15	39
6	1.0	4.0	5.0	1.0	10	15	47
7	1.0	2.0	5.0	1.0	10	15	50
8	1.0	2.0	5.0	1.5	10	15	45
9	1.0	2.0	5.0	2.0	10	15	28
10	1.0	2.0	5.0	2.5	10	15	68
11	1.0	2.0	5.0	3.0	10	15	71
12	1.0	2.0	5.0	3.5	10	15	63
13	1.0	2.0	5.0	4.0	10	15	60
14	1.0	2.0	5.0	3.0	0	15	53
15	1.0	2.0	5.0	3.0	10	15	71
16	1.0	2.0	5.0	3.0	50	15	74
17	1.0	2.0	5.0	3.0	100	15	97
18	1.0	2.0	5.0	3.0	150	15	40
19	1.0	2.0	5.0	3.0	200	15	66
20	1.0	2.0	5.0	3.0	250	15	38
21	1.0	2.0	5.0	3.0	300	15	ND

[a]Yields determined by ^{1}H NMR using 1,3-benzodioxole as an external standard. All reactions were irradiated using blue LEDs, and the reaction temperatures were maintained at ~ 70 °C. The reactor setup had a capacity for up to 6 reactions running in parallel.

The optimization of reaction conditions relied on NMR yield, which was determined using an internal standard that was added at the end of the reaction. The peak at 5.94 ppm (singlet, 2H) of 1,3-benzodioxole (internal standard) was utilized for comparison with our product peak at 2.3 ppm (triplet, 3H). The integration of the internal standard peak was standardized to 1, corresponding to 2H, and subsequently compared with the triplet signal. Additionally, GC-MS analysis was conducted alongside NMR to further confirm the presence of product.

The NMR yield of primary bromide was evaluated following a standard procedure (C). Benzyl nonanoate was synthesized utilizing 1-bromohexane (1 equiv), benzyl acrylate (0.5 equiv), DIPEA (1.0 equiv), DI water (10 equiv), 2-isopropyl-3,5-lutidinium iodide (0.05 equiv, 5 mol%, 1.5 mg), and 0.5 mL of a stock solution of $[Ir(2',4'\text{-dF-5-CF}_3\text{-ppy})_2(4,4'\text{-dtbbpy})]PF_6$ (0.25 mM) in MeCN. Comparing data under these conditions yielded the NMR yield of 24% (Table 3).

Spectra for entry **1** in **Table S2** is provided for reference. They demonstrate how optimization was carried out across different classes and how yields were determined.

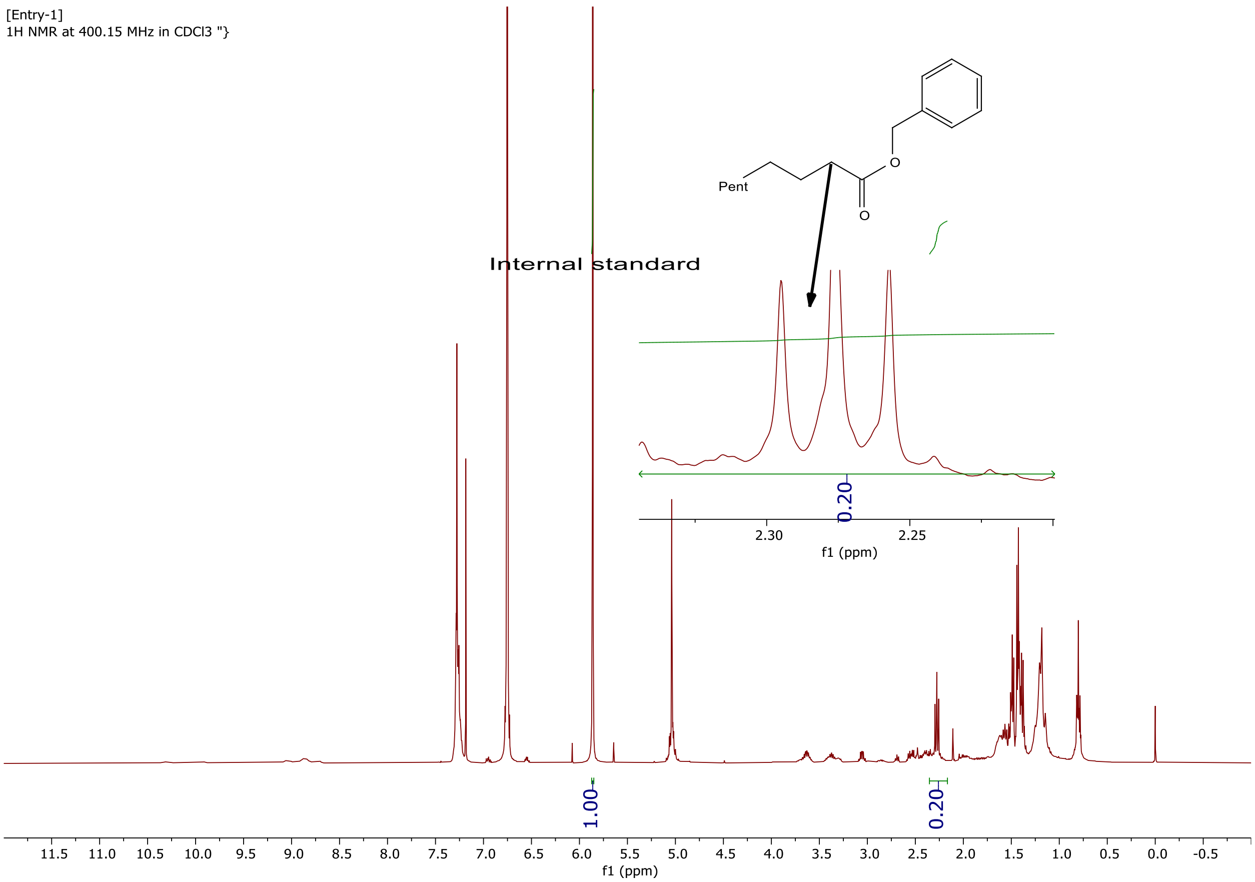

The GC-MS taken for each entry was compared with the isolated compound GC-MS to confirm the product formation at retention time 9.8 showing m/z 248.

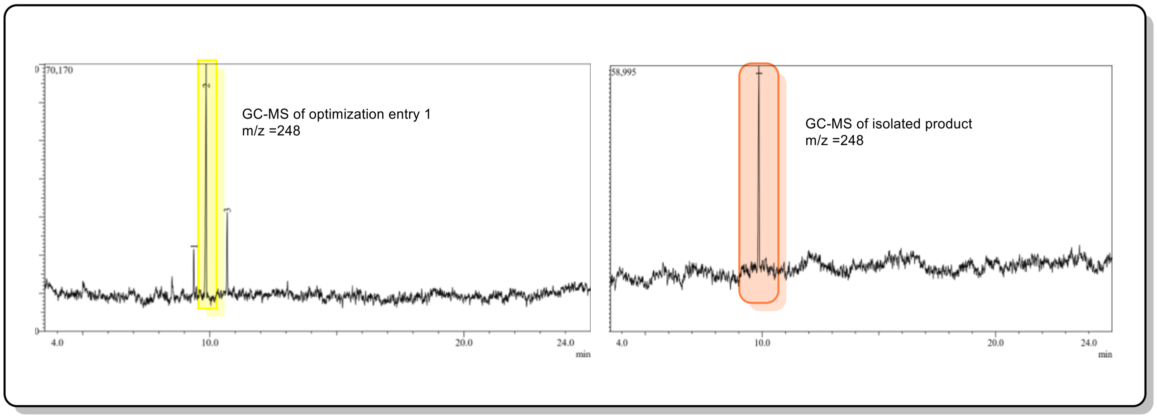

Table S3: Optimization of reaction conditions for primary chloride

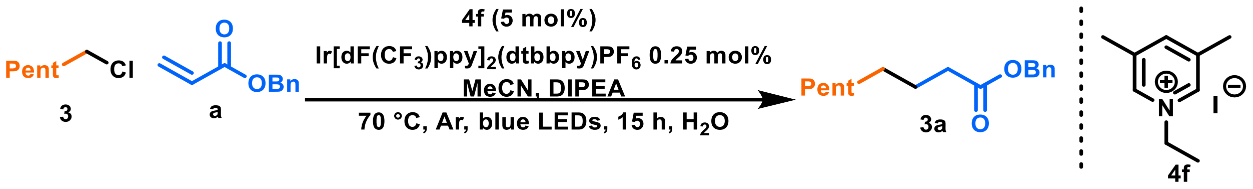

Entry	1 (equiv)	2 (equiv)	Salt (4f) (mol%)	DIPEA (equiv)	H_2O (equiv)	Time (h)	Yield of 3a%[a]
1	1.0	0.5	5.0	1.0	10	15	9
2	1.0	1.0	5.0	1.0	10	15	16
3	1.0	1.5	5.0	1.0	10	15	39
4	1.0	2.0	5.0	1.0	10	15	29
5	1.0	3.0	5.0	1.0	10	15	44
6	1.0	4.0	5.0	1.0	10	15	32
7	1.0	3.0	5.0	1.0	10	15	40
8	1.0	3.0	5.0	1.5	10	15	45
9	1.0	3.0	5.0	2.0	10	15	47
10	1.0	3.0	5.0	2.5	10	15	50
11	1.0	3.0	5.0	3.0	10	15	55
12	1.0	3.0	5.0	3.5	10	15	90
13	1.0	3.0	5.0	3.5	0	15	76
14	1.0	3.0	5.0	4.0	10	15	59
15	1.0	3.0	5.0	3.5	10	15	90
16	1.0	3.0	5.0	3.5	50	15	74
17	1.0	3.0	5.0	3.5	100	15	71
18	1.0	3.0	5.0	3.5	150	15	43
19	1.0	3.0	5.0	3.5	200	15	42
20	1.0	3.0	5.0	3.5	250	15	ND
21	1.0	3.0	5.0	3.5	300	15	ND

[a]Yields determined by ^1H NMR using 1,3-benzodioxole as an external standard. All reactions were irradiated using blue LEDs, and the reaction temperatures were maintained at ~ 70 °C. The reactor setup had a capacity for up to 6 reactions running in parallel.

Table S4. Optimization of reaction conditions for secondary chlorides

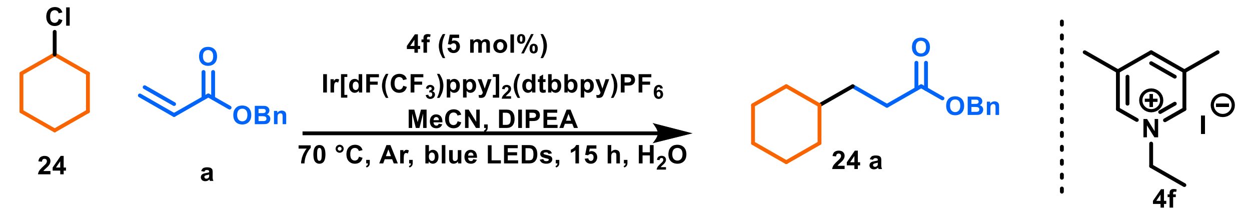

Entry	1 (equiv)	2 (equiv)	Salt (4f) (mol%)	DIPEA (equiv)	H_2O (equiv)	Time (h)	Yield of 24a%[a]
1	1.0	0.5	5.0	1.0	10	15	19
2	1.0	1.0	5.0	1.0	10	15	22
3	1.0	1.5	5.0	1.0	10	15	23
4	1.0	2.0	5.0	1.0	10	15	30
5	1.0	3.0	5.0	1.0	10	15	27
6	1.0	4.0	5.0	1.0	10	15	24
7	1.0	2.0	5.0	1.0	10	15	30
8	1.0	2.0	5.0	1.5	10	15	34
9	1.0	2.0	5.0	2.0	10	15	37
10	1.0	2.0	5.0	2.5	10	15	40
11	1.0	2.0	5.0	3.0	10	15	35
12	1.0	2.0	5.0	3.5	10	15	33
13	1.0	2.0	5.0	4.0	10	15	31
14	1.0	2.0	5.0	4.0	0	15	ND
15	1.0	2.0	5.0	2.5	10	15	40
16	1.0	2.0	5.0	2.5	50	15	35
17	1.0	2.0	5.0	2.5	100	15	30
18	1.0	2.0	5.0	2.5	150	15	24
19	1.0	2.0	5.0	2.5	200	15	ND
20	1.0	2.0	5.0	2.5	250	15	ND
21	1.0	2.0	5.0	2.5	300	15	ND

[a]Yields determined by ^1H NMR using 1,3-benzodioxole as an external standard. All reactions were irradiated using blue LEDs, and the reaction temperatures were maintained at ~ 70 °C. The reactor setup had a capacity for up to 6 reactions running in parallel.

Table S5. Optimization of reaction conditions for secondary bromide

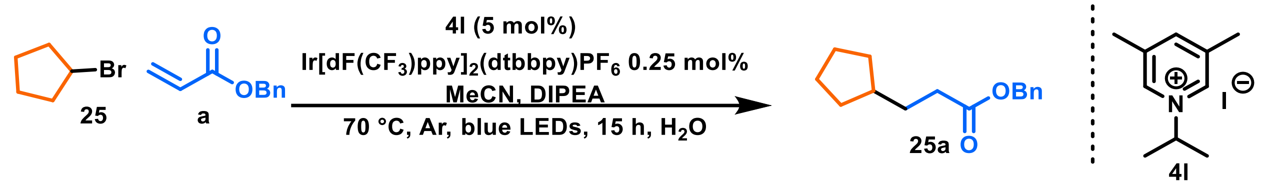

Entry	1 (equiv)	2 (equiv)	Salt (4l) (mol%)	DIPEA (equiv)	H_2O (equiv)	Time (h)	Yield of 23a%[a]
1	1.0	0.5	5.0	1.0	10	15	12
2	1.0	1.0	5.0	1.0	10	15	32
3	1.0	1.5	5.0	1.0	10	15	42
4	1.0	2.0	5.0	1.0	10	15	57
5	1.0	3.0	5.0	1.0	10	15	53
6	1.0	4.0	5.0	1.0	10	15	ND
7	1.0	2.0	5.0	1.0	10	15	57
8	1.0	2.0	5.0	1.5	10	15	59
9	1.0	2.0	5.0	2.0	10	15	62
10	1.0	2.0	5.0	2.5	10	15	63
11	1.0	2.0	5.0	3.0	10	15	69
12	1.0	2.0	5.0	3.5	10	15	53
13	1.0	2.0	5.0	4.0	10	15	53
14	1.0	2.0	5.0	3.0	0	15	57
15	1.0	2.0	5.0	3.0	10	15	69
16	1.0	2.0	5.0	3.0	50	15	72
17	1.0	2.0	5.0	3.0	100	15	71
18	1.0	2.0	5.0	3.0	150	15	60
19	1.0	2.0	5.0	3.0	200	15	52
20	1.0	2.0	5.0	3.0	250	15	42
21	1.0	2.0	5.0	3.0	300	15	20

[a] Yields determined by ^{1}H NMR using 1,3-benzodioxole as an external standard. All reactions were irradiated using blue LEDs, and the reaction temperatures were maintained at ~ 70 °C. The reactor setup had a capacity for up to 6 reactions running in parallel.

Table S6. Optimization of reaction conditions for tertiary chloride

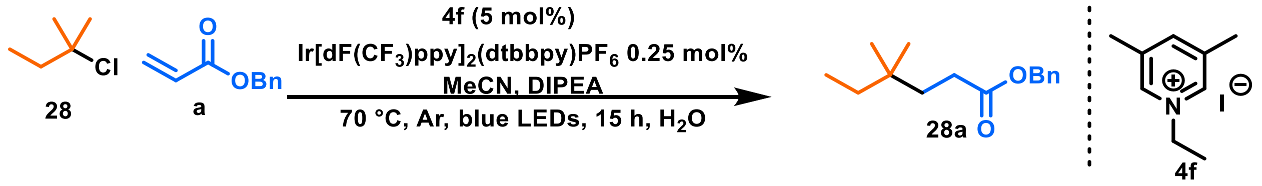

Entry	1 (equiv)	2 (equiv)	Salt (4f) (mol%)	DIPEA (equiv)	H_2O (equiv)	Time (h)	Yield of 28a%[a]

1	1.0	0.5	5.0	1.0	10	15	5
2	1.0	1.0	5.0	1.0	10	15	9
3	1.0	1.5	5.0	1.0	10	15	14
4	1.0	2.0	5.0	1.0	10	15	24
5	1.0	2.5	5.0	1.0	10	15	41
6	1.0	3.0	5.0	1.0	10	15	36
7	1.0	4.0	5.0	1.0	10	15	31
8	1.0	2.5	5.0	1.0	10	15	30
9	1.0	2.5	5.0	1.5	10	15	35
10	1.0	2.5	5.0	2.0	10	15	56
11	1.0	2.5	5.0	2.5	10	15	40
12	1.0	2.5	5.0	3.0	10	15	31
13	1.0	2.5	5.0	3.5	10	15	20
14	1.0	2.5	5.0	4.0	10	15	22
15	1.0	2.5	5.0	4.0	0	15	10
16	1.0	2.5	5.0	2.0	10	15	57
17	1.0	2.5	5.0	2.0	50	15	55
18	1.0	2.5	5.0	2.0	100	15	51
19	1.0	2.5	5.0	2.0	150	15	43
20	1.0	2.5	5.0	2.0	200	15	ND
21	1.0	2.5	5.0	2.0	250	15	ND

[a]Yields determined by ^1H NMR using 1,3-benzodioxole as an external standard. All reactions were irradiated using blue LEDs, and the reaction temperatures were maintained at ~ 70 °C. The reactor setup had a capacity for up to 6 reactions running in parallel.

Table S7. Optimization of reaction conditions for tertiary bromide

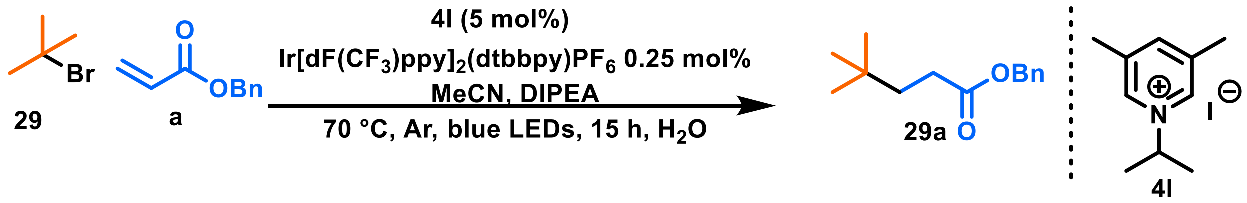

Entry	1 (equiv)	2 (equiv)	Salt (4l) (mol%)	DIPEA (equiv)	H_2O (equiv)	Time (h)	Yield of 29a%[a]
1	1.0	0.5	5.0	1.0	10	15	10
2	1.0	1.0	5.0	1.0	10	15	17.8
3	1.0	1.5	5.0	1.0	10	15	20
4	1.0	2.0	5.0	1.0	10	15	23

5	1.0	2.5	5.0	1.0	10	15	25
6	1.0	3.0	5.0	1.0	10	15	21
7	1.0	4.0	5.0	1.0	10	15	18
8	1.0	2.5	5.0	1.0	10	15	24
9	1.0	2.5	5.0	1.5	10	15	ND
10	1.0	2.5	5.0	2.0	10	15	ND
11	1.0	2.5	5.0	2.5	10	15	25
12	1.0	2.5	5.0	3.0	10	15	30
13	1.0	2.5	5.0	3.5	10	15	31
14	1.0	2.5	5.0	4.0	10	15	54
15	1.0	2.5	5.0	4.0	0	15	25
16	1.0	2.5	5.0	4.0	10	15	54
17	1.0	2.5	5.0	4.0	50	15	50
18	1.0	2.5	5.0	4.0	100	15	47
19	1.0	2.5	5.0	4.0	150	15	42
20	1.0	2.5	5.0	4.0	200	15	ND
21	1.0	2.5	5.0	4.0	250	15	ND

[a]Yields determined by ^{1}H NMR using 1,3-benzodioxole as an external standard. All reactions were irradiated using blue LEDs, and the reaction temperatures were maintained at ~ 70 °C. The reactor setup had a capacity for up to 6 reactions running in parallel.

Table S8. Photocatalyst screening

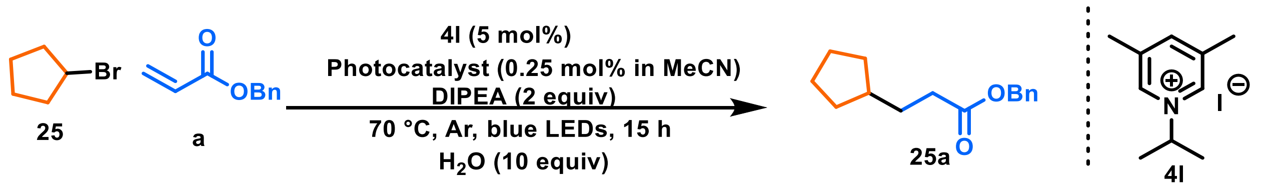

Entry	Photocatalyst	NMR Yield (%) [a]
**1**	Ir(dFppy)$_3$	51
**2**	Ir(Fppy)$_3$	39
**3**	Ir[p-F(t-Bu)-ppy]$_3$	38
**4**	Ir(dtbbpy)(dtbppy)$_2$PF$_6$	68
**5**	[Ru(dmbpy)$_3$]PF$_6$	No product formation
**6**	Ru(bpy)$_3$(PF$_6$)$_2$	13
**7**	(Ir[Me(Me)ppy]$_2$(dtbpy))PF$_6$	54
**8**	Ir[dF(CF$_3$)ppy]$_2$ (dtbbpy) PF$_6$	72
**9**	Ir(ppy)$_3$	No product formation

[a] Yields determined by ^{1}H NMR using 1,3-benzodioxole as an external standard. All reactions were irradiated using blue LEDs, and the reaction temperatures were maintained at ~ 70 °C. The reactor setup had a capacity for up to 6 reactions running in parallel.

Screened Photocatalysts

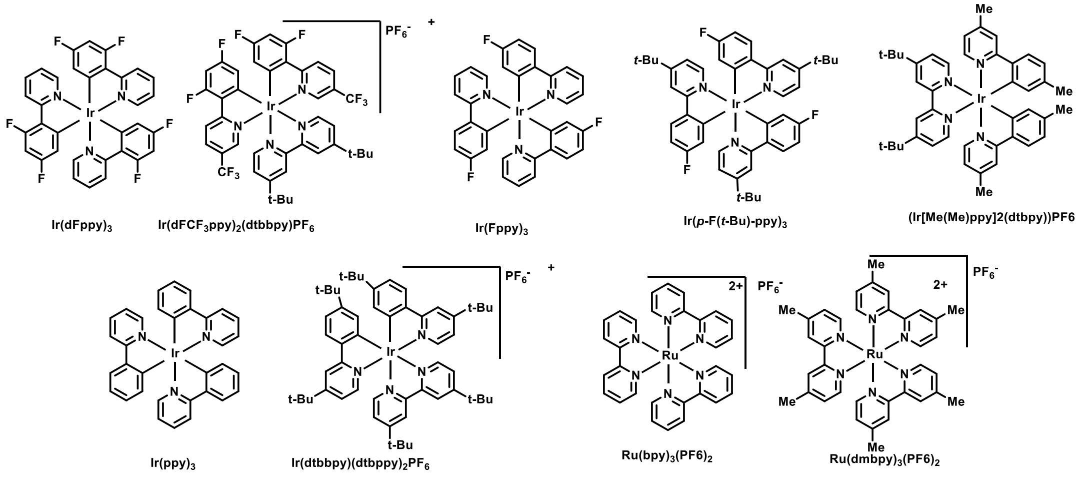

Table S9. Solvent Screening

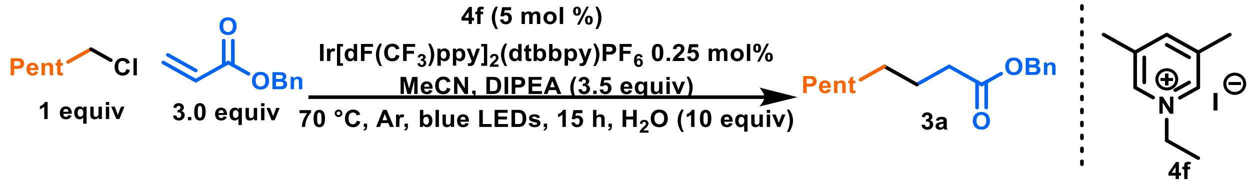

Entry	Photocatalyst	NMR Yield (%) [3a]
1	Acetonitrile	91
2	DMSO	84
3	DMF	76
4	Toluene	Trace amount of product observed
5	Ethyl Acetate	No product formation

[a] Yields determined by ^{1}H NMR using 1,3-benzodioxole as an external standard. All reactions were irradiated using blue LEDs, and the reaction temperatures were maintained at ~ 70 °C. The reactor setup had a capacity for up to 6 reactions running in parallel.

Table S10: Control studies for various parameters

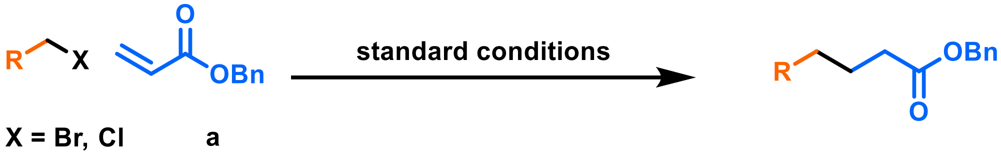

Variation from standard conditions	1° Br (3a)	2° Br (23a)	3° Br (29a)	1° Cl (3a)	2° Cl (24a)	3° Cl (28a)
**Standard conditions reference**	**Table S2**	**Table S5**	**Table S7**	**Table S3**	**Table S4**	**Table S6**
**None**	97%	72%	54%	90%	40%	57%
**No blue LEDs**	0	0	0	0	0	0
**rt, 24 h**	0	0	0	0	0	0
**DIPEA (0 equiv)**	0	0	0	0	0	0
**Salt (0 equiv)**	0	0	0	0	0	0
**4c salt (5 mol%)**	70%	56%	32%	0	0	0

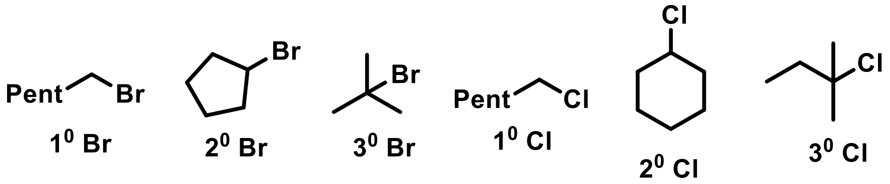

Table S11: Control studies for EDA Complex detection

An EDA complex involving lutidinium salt and DIPEA was observed absorb light in the UV region and tail into the visible region. As a result, we ran control experiments to probe whether this EDA complex might be responsible for SET. While trace amounts of product (**3a**) were detected in the absence of photocatalyst, the efficiency of this process was so low that it could not be responsible for the product formation.

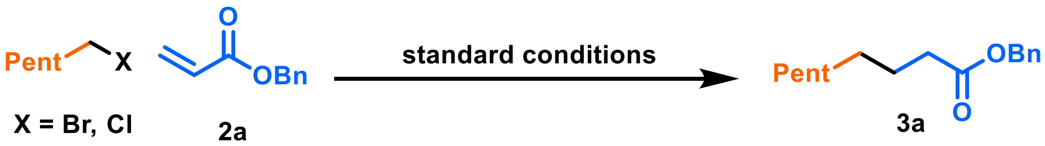

Variation from standard conditions	Reactor	1° Br (3a)	1° Cl (3a)

Standard conditions reference		Table S2	Table S3
**None**	Blue LEDs	97%	90%
**No photocatalyst**	Blue LEDs	No product formation	A trace amount of product observed
**No photocatalyst**	UV	A trace amount of product observed	A trace amount of product observed

F. Mechanistic Studies

F1: Isotopic labeling experiment

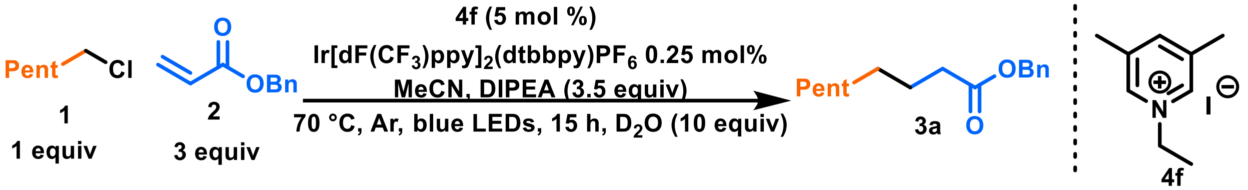

An isotopic labeling experiment was carried out by replacing the H_2O with D_2O in otherwise standard reaction conditions (see X). Reactions were set up using 1-chlorohexane (0.12 mmol, 14.5 mg, 16.4 µL, 1 equiv), benzyl acrylate (0.36 mmol, 58 mg, 3.0 equiv, 54.7 µL), DIPEA (0.42 mmol, 54.3 mg, 73.2 µL, 3.5 equiv), D_2O (1.2 mmol, 21.6 mg, 21.6 µL, 10 equiv), ethyl-3,5-lutidinium iodide (6.1 µmol, 1.6 mg, 0.05 equiv) and 0.5 mL of stock solution of [Ir(2′,4′-dF-5-CF_3-ppy)$_2$(4,4′-dtbbpy)]PF_6 (0.25 mol%) in MeCN. After the completion of the reaction 15 h, the crude was purified via automated flash chromatography using EtOAc in hexanes (0% to 100%) with product eluting at 1.0% on a 4 g silica column to isolate it. The 1H NMR spectrum was recorded and shows the incorporation of deuterium in the carbon alpha to the carbonyl group of the benzyl acrylate product. The deuterium incorporation is 12%. This mid-level amount of deuterium incorporations is consistent with past findings in related Giese couplings[4] which have also observed intermediate D-incorporation. The simplest interpretation involves a reaction in which HAT from a DIPEA would give the di-protio product **3a** while PCET would yield **3a-D**. However, others have shown that upon irradiation almost immediately, isotopic scrambling into the amine starts to occur which further complicates interpretation.[5]

[3a]
1H NMR at 800.34 MHz in CDCl3

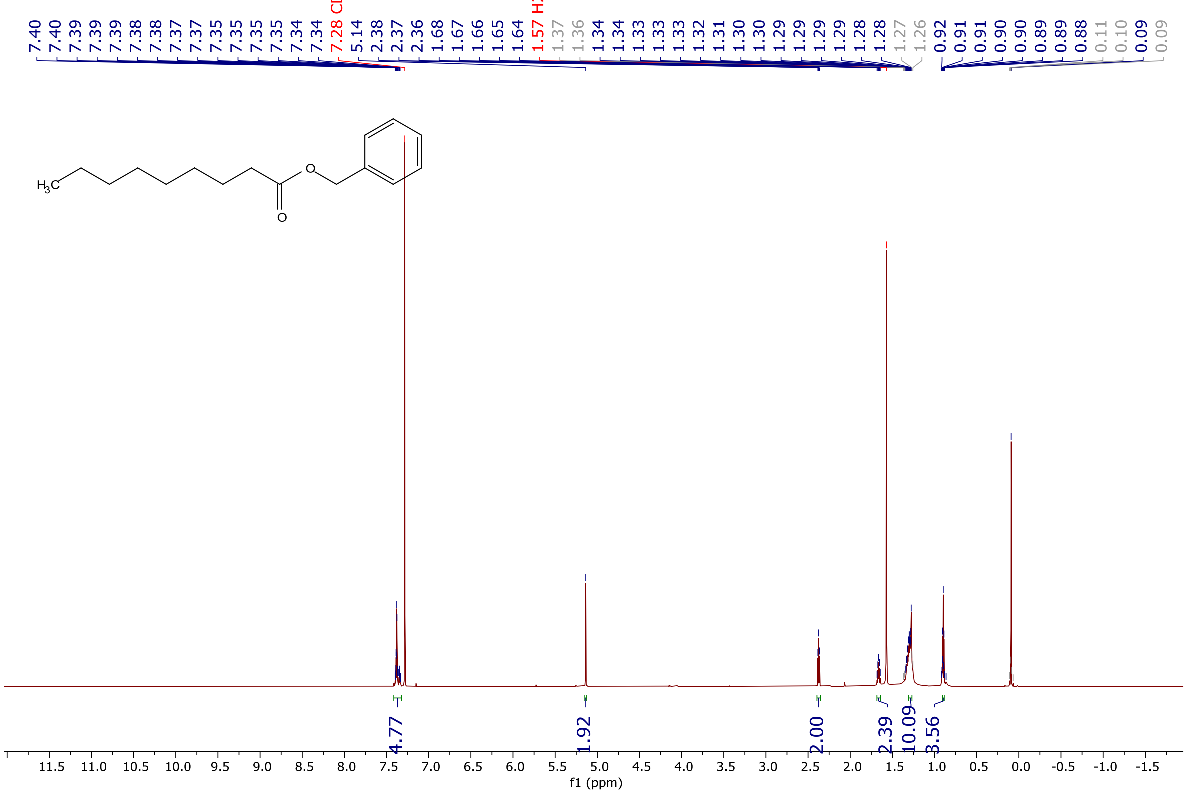

[3a-D]
1H NMR at 800.34 MHz in CDCl3

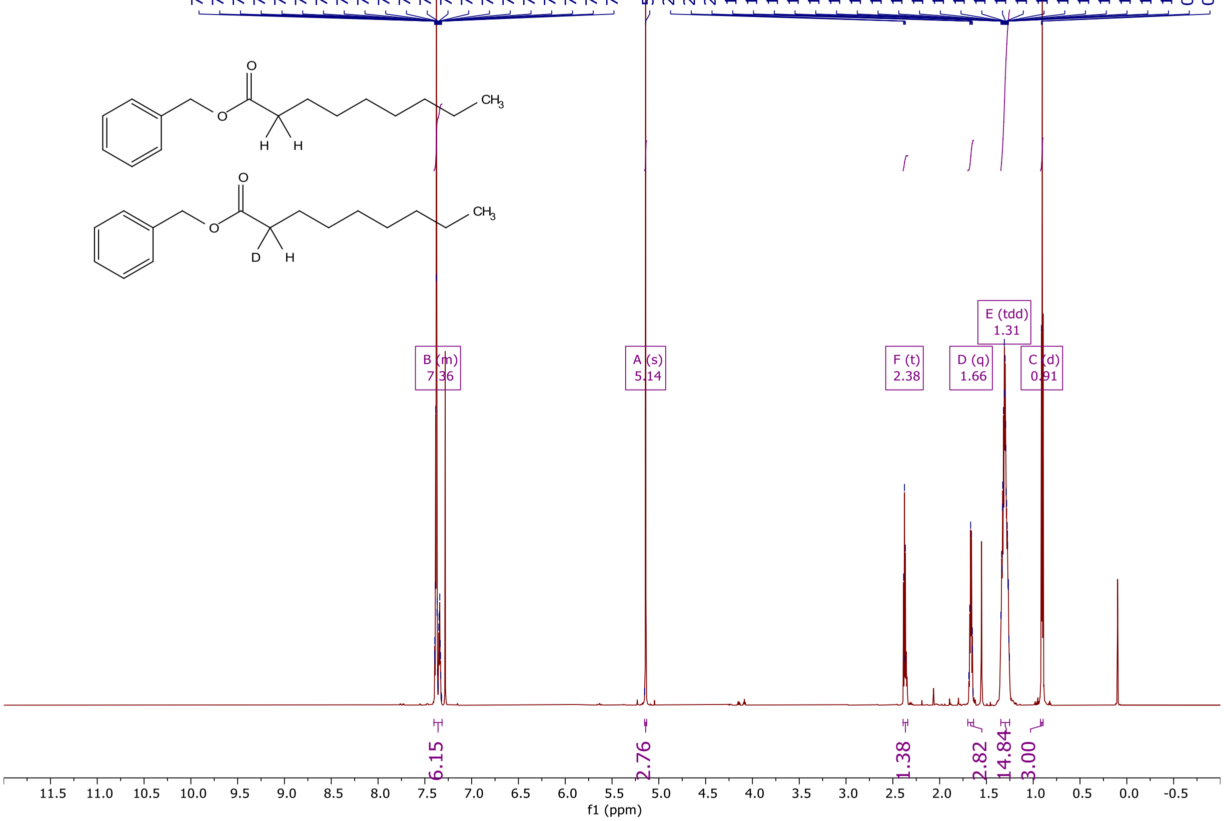

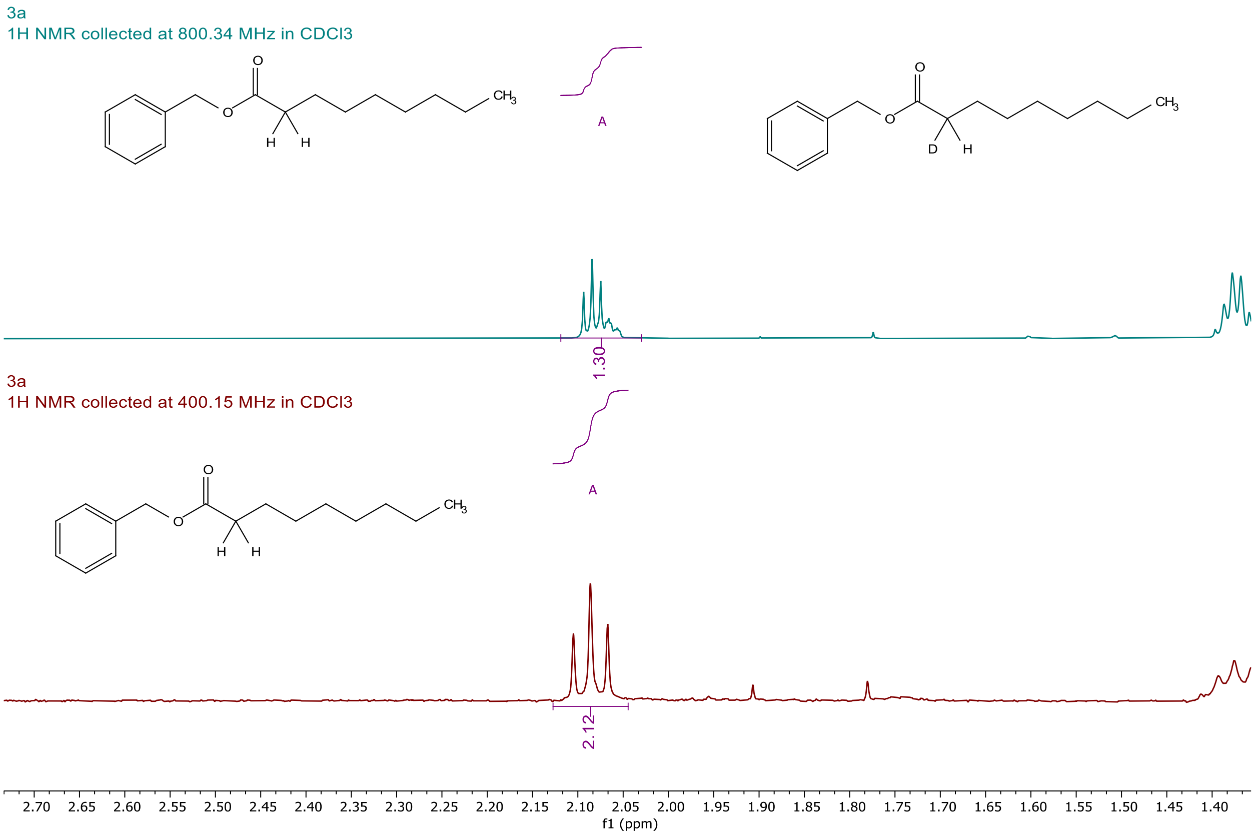

The stacked proton NMR spectrum is presented here, highlighting a tt signal at 2.3 ppm in the ^{1}H NMR. This signal is attributed to the incorporation of deuterium at the alpha-carbon position of the product. The shift and splitting pattern observed are consistent with the expected chemical environment around the alpha-carbon, further validating the incorporation of deuterium into the molecule.

F2: Radical Probe Experiment

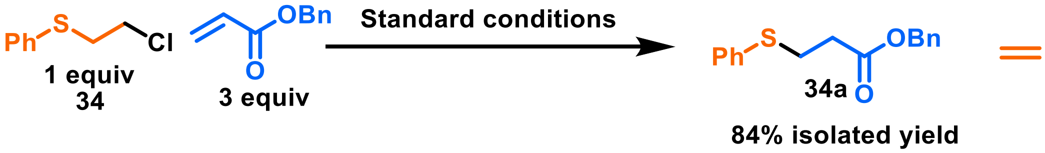

A radical probe experiment was carried out using 2-chloroethyl phenyl sulfide (0.12 mmol, 20.7 mg, 17.7 µL, 1 equiv), benzyl acrylate (0.36 mmol, 58 mg, 3.0 equiv, 54.7 µL), DIPEA (0.42 mmol, 54.3 mg, 73.2 µL, 3.5 equiv), DI water (1.2 mmol, 21.6 mg, 21.6 µL, 10 equiv), ethyl-3,5-lutidinium iodide (6.1 µmol, 1.6 mg, 0.05 equiv) and 0.5 mL of stock solution of [Ir(2′,4′-dF-5-CF_3-ppy)$_2$(4,4′-dtbbpy)]PF_6 (0.25 mol%) in MeCN (0.1 M). After the completion of the reaction 15 h, the crude was purified via automated flash

chromatography using EtOAc in hexanes (0% to 100%) with product benzyl 3-(phenylthio)propanoate eluting at 5.0% on a 4 g silica column to afford it in 84% yield (27.4 mg, 0.10 mmol) as an oil. NMR chemical shifts match with the literature value.[6] The experiment is consistent with the formation of a primary alkyl radical that undergoes a rapid beta-scission to eliminate a phenyl thiyl radical followed by reaction with the acrylate.

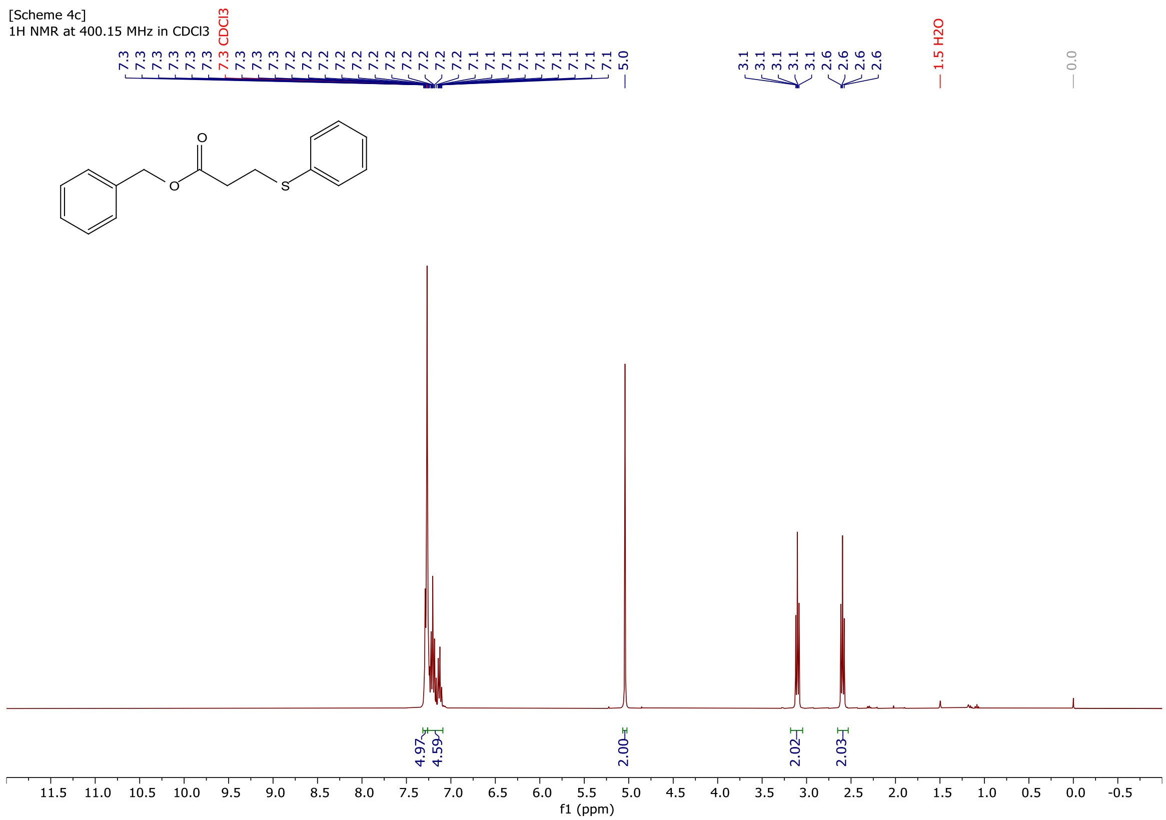

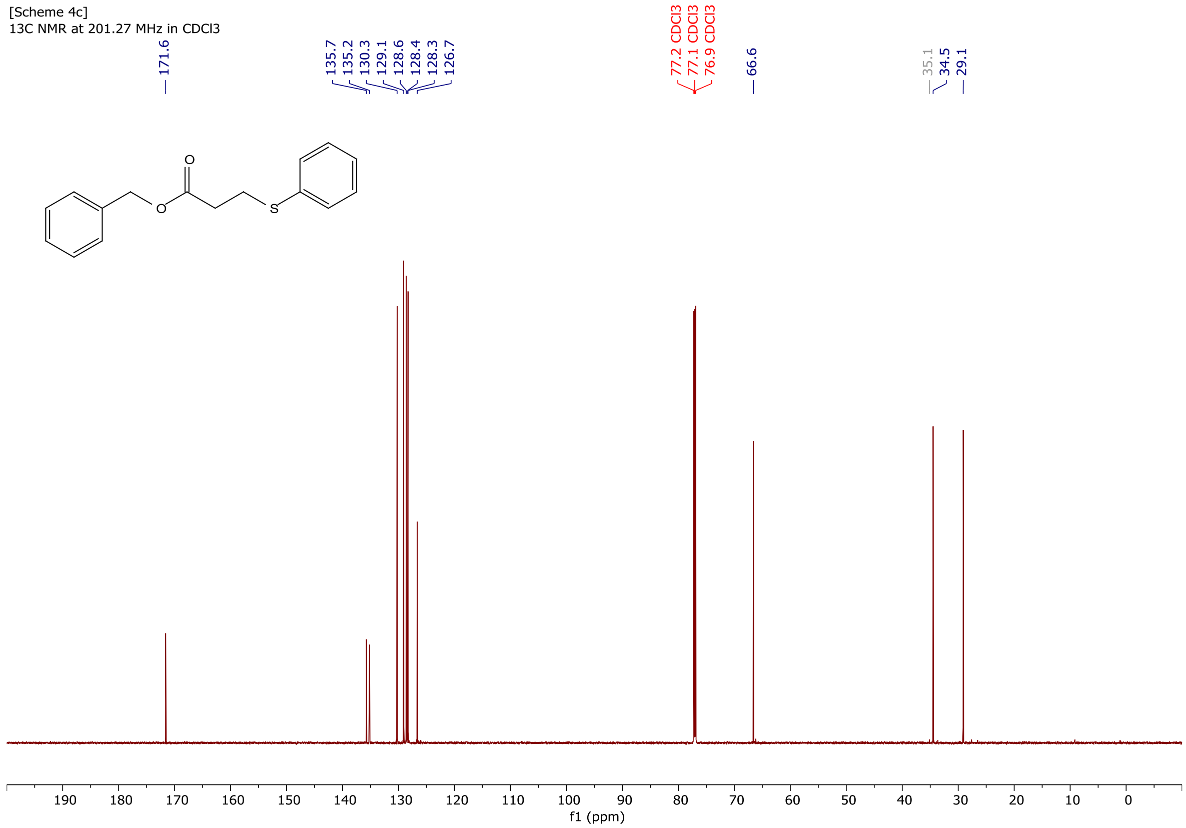

F3: Electrochemical measurements

The lutidinium salts were investigated using cyclic voltammetry. Cyclic voltammograms and electrochemical potentials were obtained with a Pine WaveNow Potentiostat. All the samples were prepared with 0.3 mmol of substrate in 30 mL of 0.1 M tetra-n-butylammonium hexafluorophosphate in dry, degassed acetonitrile. Measurements were taken using a glassy carbon working electrode (3 mm diameter, 0.07 cm^2), a platinum wire counter electrode, and a 4.0 M KCl Ag/AgCl reference electrode. Before use, the working electrode was polished using a 0.05 µm alumina slurry on the micro cloth. All measurements were performed at room temperature. Data was analyzed using AFTERMATH software by identifying the maximum current (Cp) and determining the potential (Ep/2) at half of the maximum current (Cp/2). The obtained value was referenced to Ag/AgCl and converted to SCE by subtracting 0.03 V. The initial potential was set to 1000 mV, the direction of the initial scan (oxidative), and the scan rate was 100 mV/s.

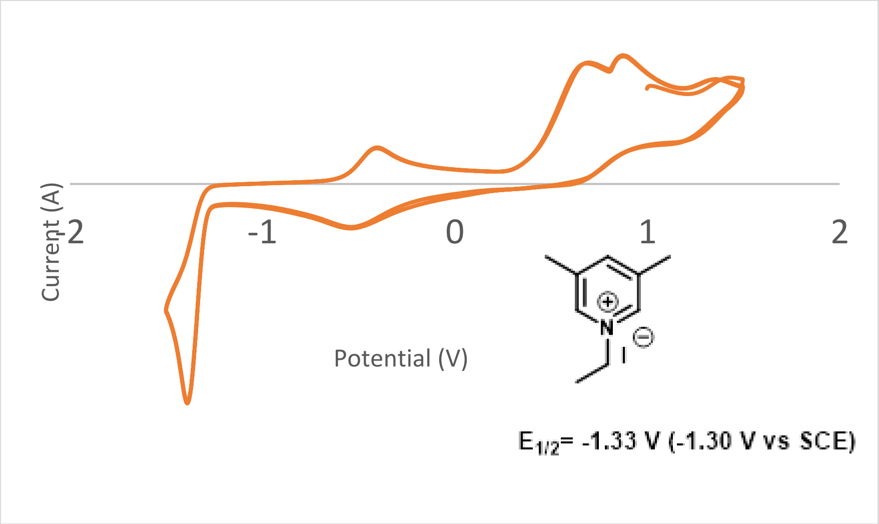

Figure 1: Cyclic voltammogram of *N*-ethyl lutidinium iodide using the IUPAC plotting convention. Starting point: +1.0 V, oxidative scan. Working electrode - glassy carbon, a platinum wire counter electrode, and a 4.0 M KCl Ag/AgCl reference electrode. The sample was prepared with 0.3 mmol of substrate in 30 mL of 0.1 M tetra-n butylammonium hexafluorophosphate in dry, degassed acetonitrile.

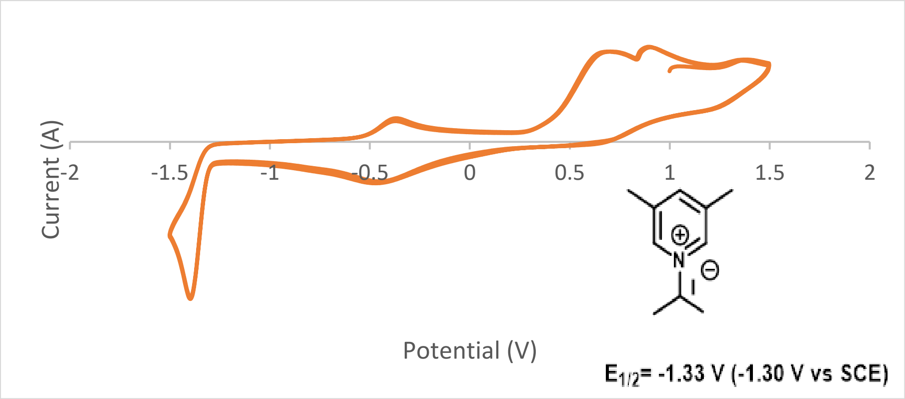

Figure 2: Cyclic voltammogram of *N*- isopropyl lutidinium iodide using the IUPAC plotting convention. Starting point: +1.0 V, oxidative scan. Working electrode - glassy carbon, a platinum wire counter electrode, and a 4.0 M KCl Ag/AgCl reference electrode. The sample was prepared with 0.3 mmol of substrate in 30 mL of 0.1 M tetra-n butylammonium hexafluorophosphate in dry, degassed acetonitrile.

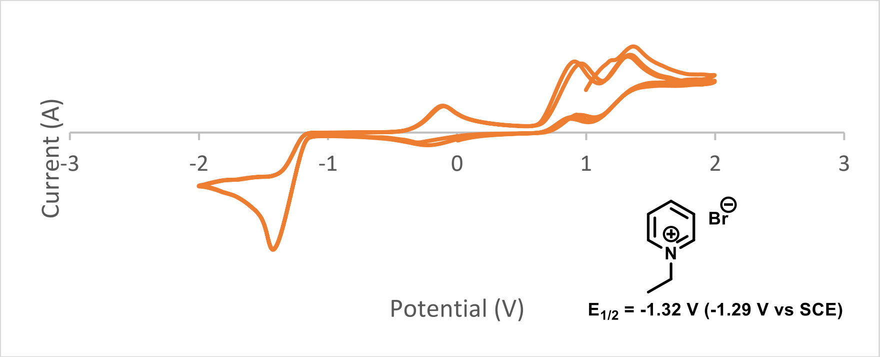

Figure 3: Cyclic voltammogram of *N*- ethyl pyridinium bromide using the IUPAC plotting convention. Starting point: +1.0 V, oxidative scan. Working electrode - glassy carbon, a platinum wire counter electrode, and a 4.0 M KCl Ag/AgCl reference electrode. The sample was prepared with 0.3 mmol of substrate in 30 mL of 0.1 M tetra-n butylammonium hexafluorophosphate in dry, degassed acetonitrile.

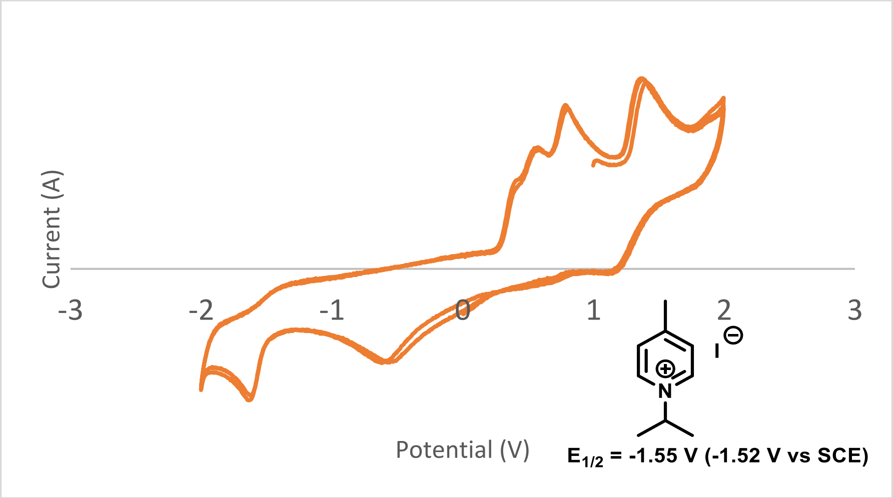

Figure 4: Cyclic voltammogram of 2-methylpyridinium isopropyl iodide using the IUPAC plotting convention. Starting point: +1.0 V, oxidative scan. Working electrode - glassy carbon, a platinum wire counter electrode, and a 4.0 M KCl Ag/AgCl reference electrode. The sample was prepared with 0.3 mmol of substrate in 30 mL of 0.1 M tetra-n butylammonium hexafluorophosphate in dry, degassed acetonitrile.

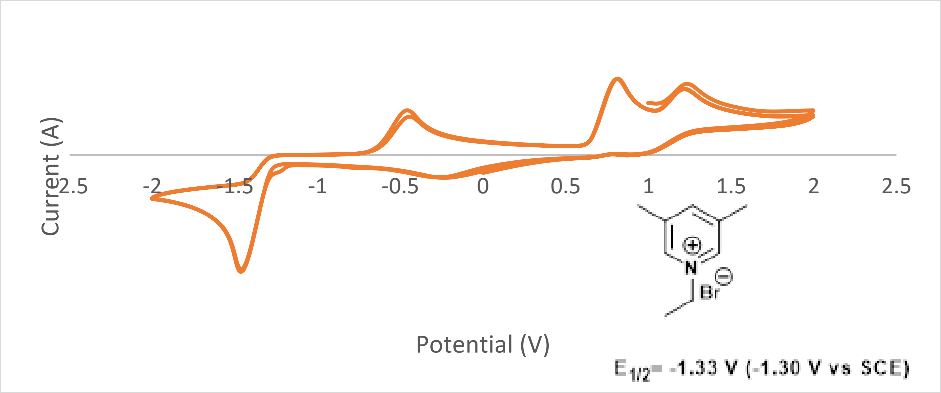

Figure 5: Cyclic voltammogram of *N*-ethyl lutidinium bromide using the IUPAC plotting convention. Starting point: +1.0 V, oxidative scan. Working electrode - glassy carbon, a platinum wire counter electrode, and a 4.0 M KCl Ag/AgCl reference electrode. The sample was prepared with 0.3 mmol of substrate in 30 mL of 0.1 M tetra-n butylammonium hexafluorophosphate in dry, degassed acetonitrile.

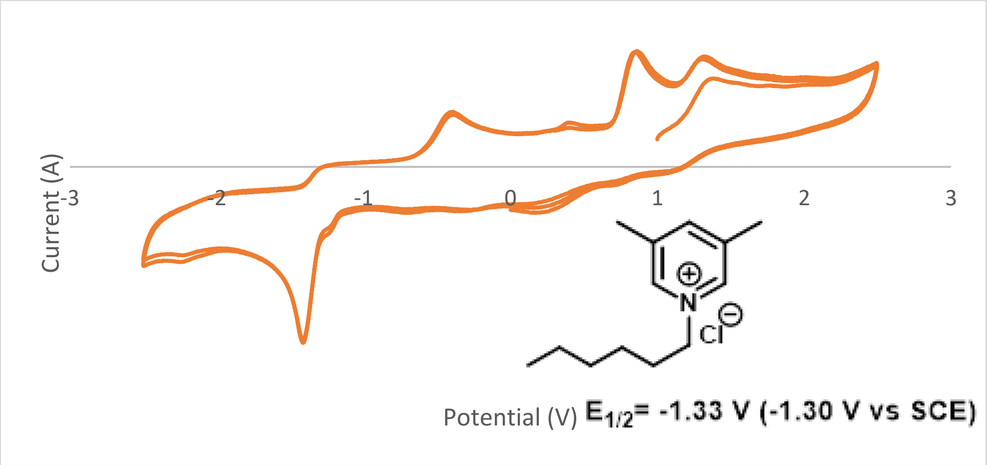

Figure 6: Cyclic voltammogram of lutidinium hexyl chloride using the IUPAC plotting convention. Starting point: +1.0 V, oxidative scan. Working electrode - glassy carbon, a platinum wire counter electrode, and a 4.0 M KCl Ag/AgCl reference electrode. The sample was prepared with 0.3 mmol of substrate in 30 mL of 0.1 M tetra-n butylammonium hexafluorophosphate in dry, degassed acetonitrile.

F4: Radical Scavenger Experiment

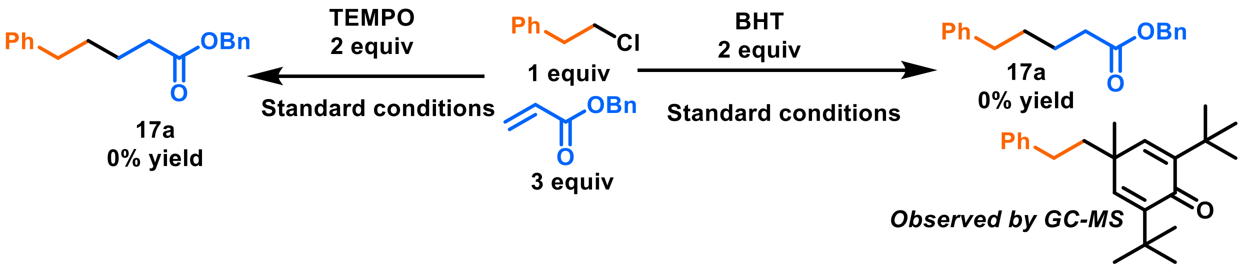

F4.1: Radical inhibition experiment was using (2-chloroethyl)benzene (0.12 mmol, 16 mg, 15.7 µL, 1 equiv), benzyl acrylate (0.36 mmol, 58 mg, 3.0 equiv, 54.7 µL), TEMPO (0.24 mmol, 37.5 mg, 2 equiv) DIPEA (0.42 mmol, 54.3 mg, 73.2 µL, 3.5 equiv), DI water (1.2 mmol, 21.6 mg, 21.6 µL, 10 equiv), ethyl-3,5-lutidinium iodide (6.1 µmol, 1.6 mg, 0.05 equiv) and 0.5 mL of stock solution of [Ir(2′,4′-dF-5-CF_3-ppy)$_2$(4,4′-dtbbpy)]PF_6 (0.25 mol%) in MeCN. After 15 hours of irradiation, the crude material was analyzed using GC-MS. The analysis revealed the starting material was still present and the absence of product (**17a**), indicating radical inhibition.

F4.2: Radical trap experiment was performed using (2-chloroethyl)benzene (0.12 mmol, 16 mg, 15.7 µL, 1 equiv), benzyl acrylate (0.36 mmol, 58 mg, 3.0 equiv, 54.7 µL), BHT (0.24 mmol, 52.8 mg, 2 equiv) DIPEA (0.42 mmol, 54.3 mg, 73.2 µL, 3.5 equiv), DI water (1.2 mmol, 21.6 mg, 21.6 µL, 10 equiv), ethyl-3,5-lutidinium iodide (6.1 µmol, 1.6 mg, 0.05 equiv) and 0.5 mL of stock solution of [Ir(2′,4′-dF-5-CF_3-ppy)$_2$(4,4′-dtbbpy)]PF_6 (0.25 mol%) in MeCN. After completing the reaction for 15 hours, the crude material was analyzed using GC-MS. The analysis did not reveal the presence of product (**17a**), and the GC-MS also indicated the m/z of 225 which is consistent with the formation of a phenyl ethyl-BHT adduct.

F5: UV Studies

Using biodetecters, a colored complex appeared to be forming which could be responsible for SET. A systematic UV-Vis study was conducted. All the solutions were transferred to 3.5 mL macro fluorescence cuvettes, with acetonitrile serving as the solvent. The samples were scanned over the wavelength range of 300–700 nm using a Shimadzu UV-2600 UV-Vis spectrometer to capture the absorption profiles. The concentrations of the components were prepared according to standard experimental conditions: lutidinium ethyl iodide (5 mol%), DIPEA (3.5 equiv), and the reaction mixture corresponding to the scheme outlined below. To ensure accurate and consistent measurements, all components were diluted by adding 3 mL of acetonitrile prior to transfer to the cuvettes. This step allowed for proper mixing and ensured the solutions were within the optimal concentration range for UV-Vis analysis.

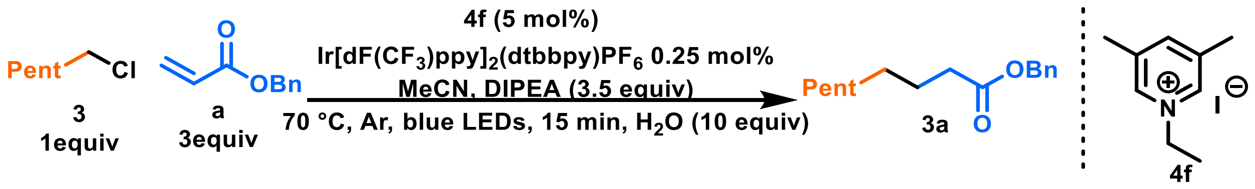

e. UV studies

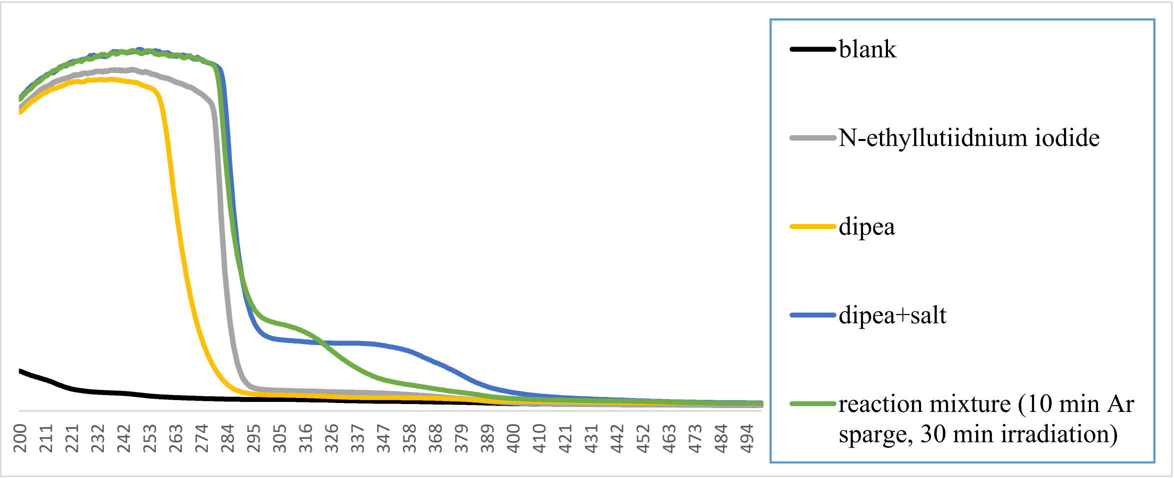

Figure 7: UV-vis Absorbance Studies

F6: Generation of alkyl iodide

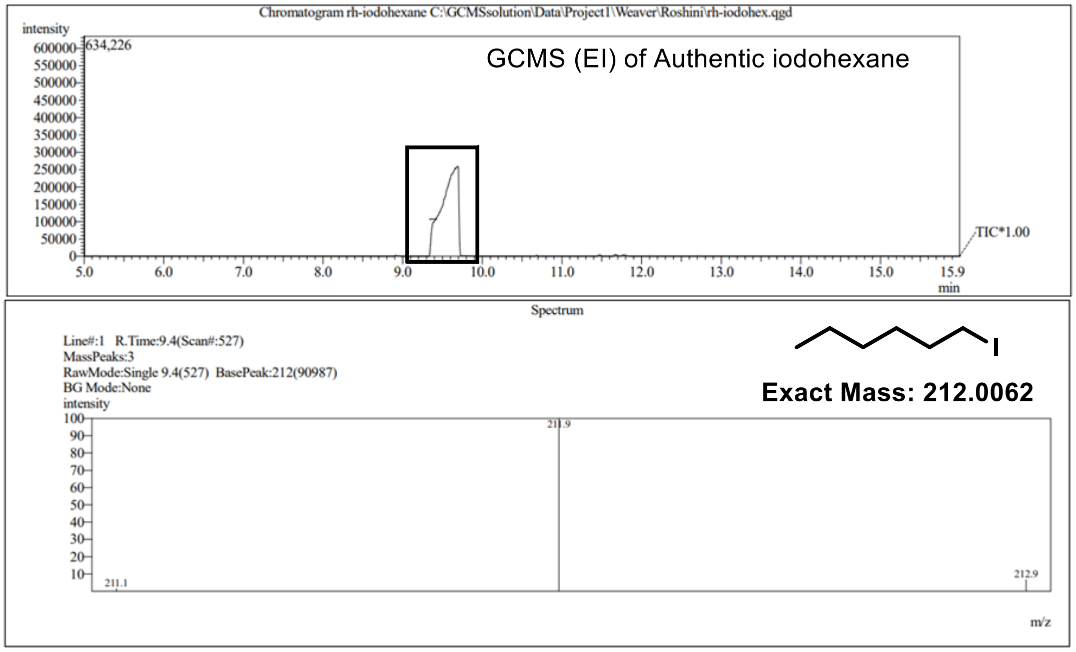

Figure 8: GCMS (EI) of Authentic iodohexane

a. **Detection of alkyl iodide intermediate at 6 h through GCMS of reaction mixture.**

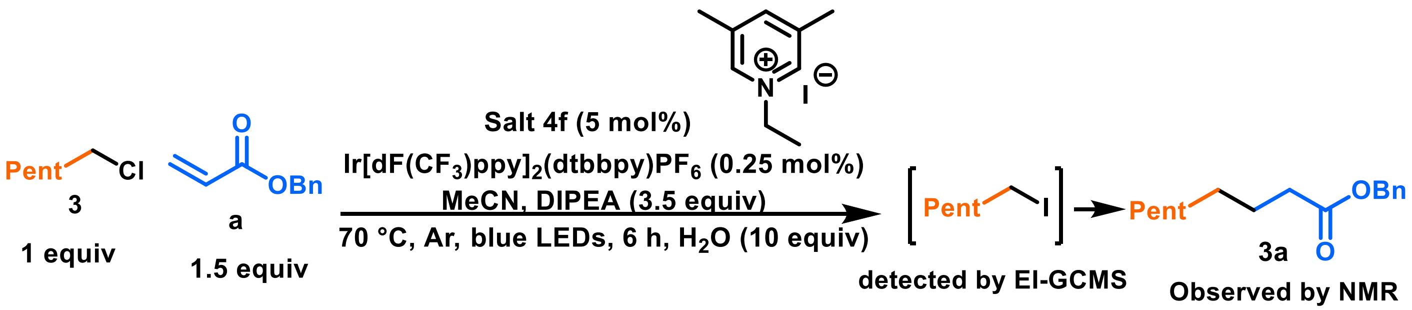

The standard time of the reaction is 15 h.

The peak observed at a retention time (RT) of 9.4 minutes and m/z = 212 indicates the formation of hexyl iodide. This identification is supported by its m/z value of 212.0, which aligns precisely with the standard GC-MS data recorded for hexyl iodide. The consistency between experimental and standard data confirms the successful detection and characterization of hexyl iodide through GC-MS analysis.

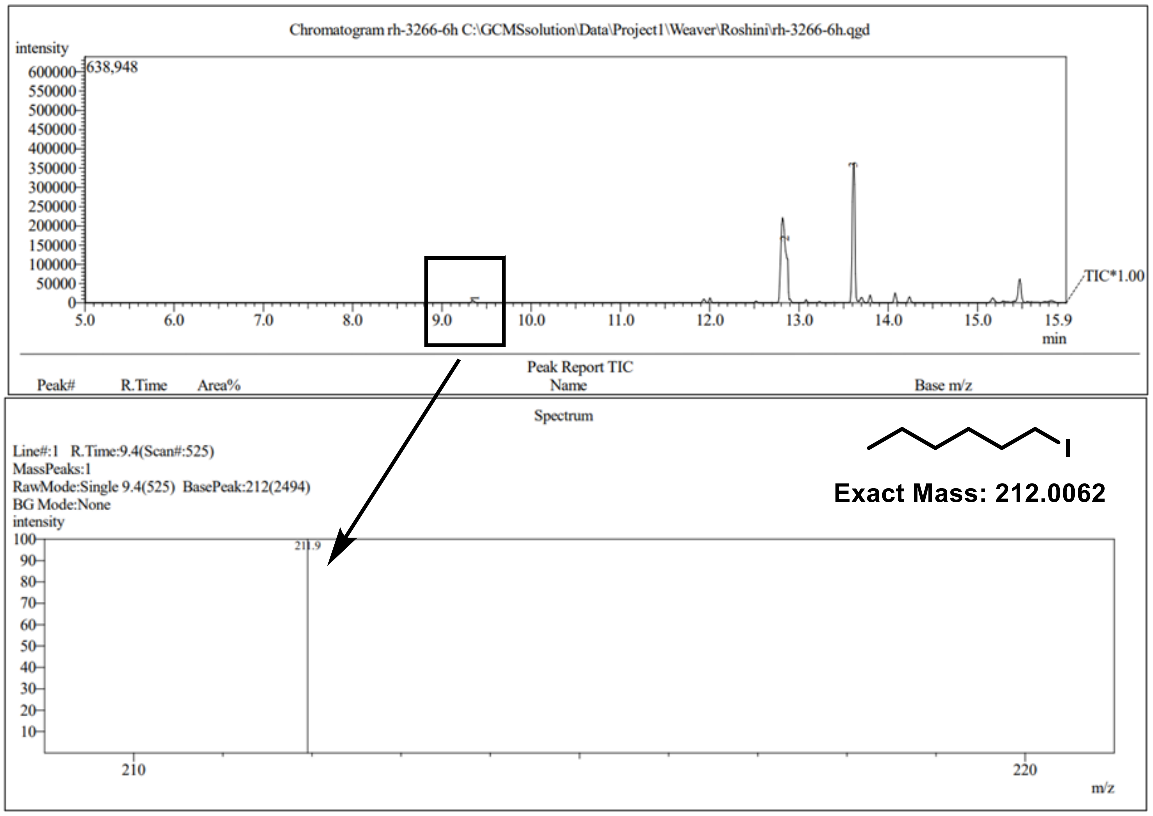

Figure 9: GCMS (EI) of crude material of F6.a

b. Detection of alkyl iodide intermediate at 6 h through GCMS of reaction mixture with tetramethylammonium iodide salt.

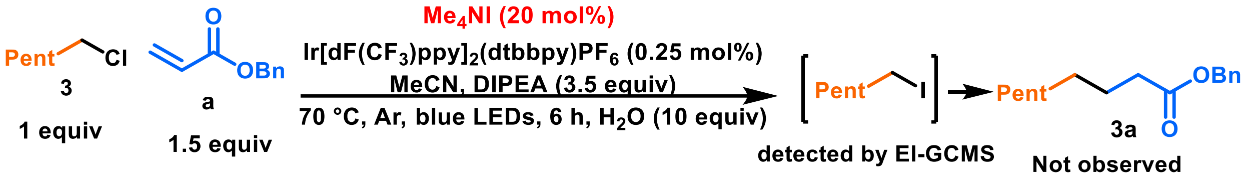

The peak observed at a retention time (RT) of 9.4 minutes confirms the formation of hexyl iodide, as evidenced by its m/z value of 212.0, which matches the standard GC-MS data for hexyl iodide. However, under experimental conditions, the formation of the **3a** product was not detected.

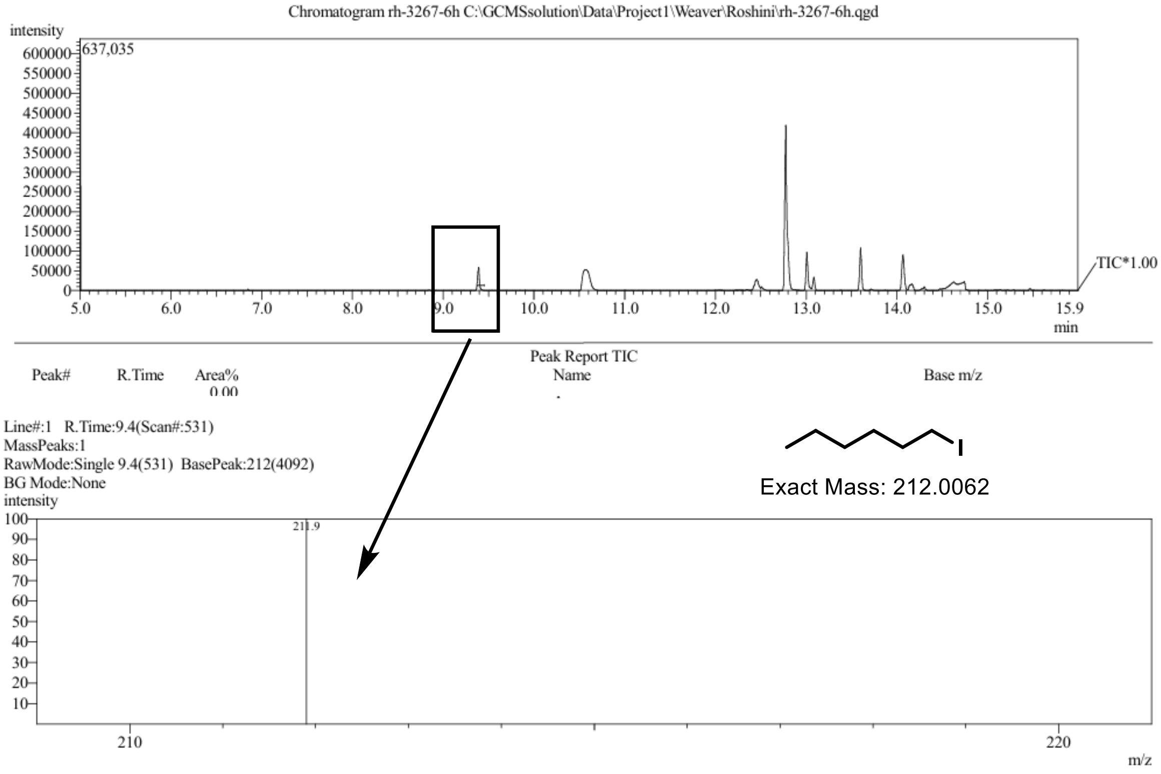

Figure 10: GCMS (EI) of crude material of F6.b

F7: Stern-Volmer experiments

Quenching study on lutidinium alkyl salts and DIPEA with catalyst [Ir(2′,4′-dF-5-CF_3-ppy)$_2$(4,4′-dtbbpy)]PF_6

2.5 µM solutions of catalyst [Ir(2′,4′-dF-5-CF_3-ppy)$_2$(4,4′-dtbbpy)]PF_6 was prepared in acetonitrile. 0.1M stock solutions of DIPEA, lutidinium ethyl iodide, lutidinium ethyl bromide and lutidinium hexyl chloride were prepared in acetonitrile. 0.5 mLstock solution of the photocatalyst was mixed with 1.5 mL of acetonitrile and transferred to a vial. Subsequently, 2 µL of a stock solution of DIPEA was added to the vial. This was followed by the sequential addition of three 10 µL aliquots of solution. The same dilution was carried out with lutidinium ethyl iodide. For the remaining two salts, a 50 mM stock solution of the photocatalyst was prepared and transferred to a vial. Following this, 5 µL of a DIPEA stock solution was added. Three sequential aliquots of 10 µL each were then introduced into the vial. The solution was mixed properly, and fluorescence was measured. The catalyst was excited at 380 nm. The emission was observed

at 468 nm for the catalyst. Fluorescence quenching experimental spectra recorded in MeCN in a 1 cm path quartz cuvettes at 25 ºC.

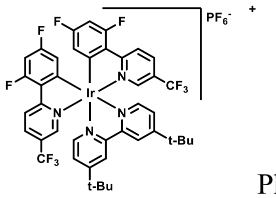

Photocatalyst being studied.

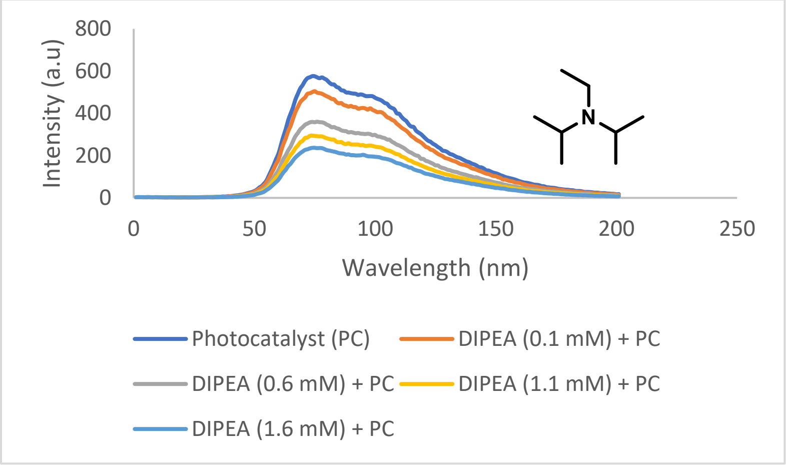

Figure 11: Quenching with DIPEA

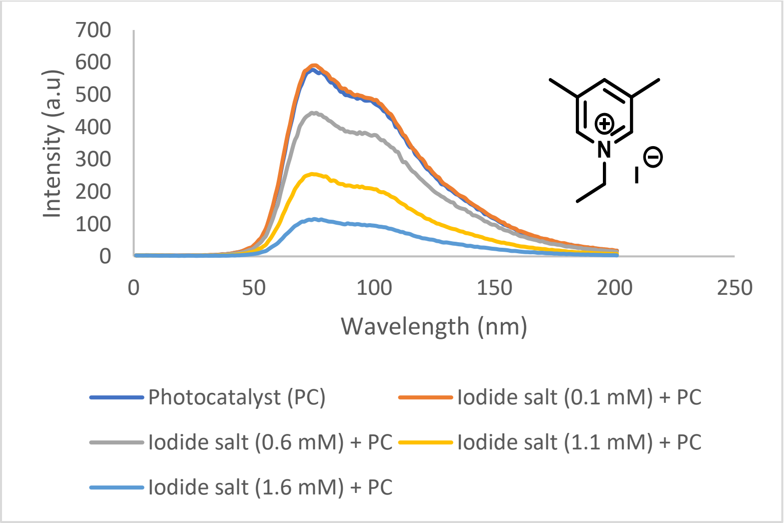

Figure 12: Quenching with lutidinium ethyl iodide

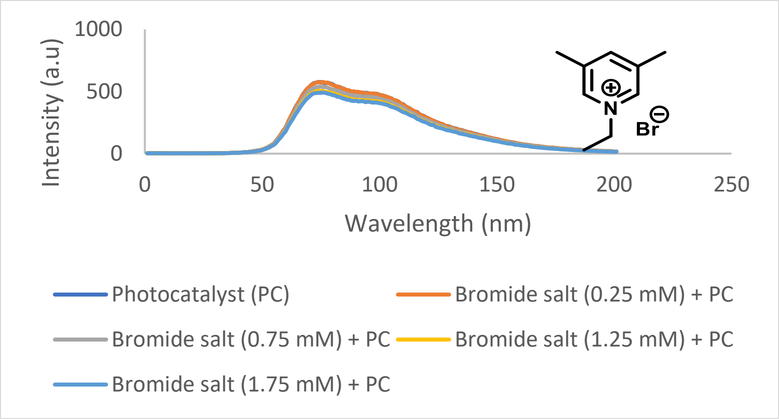

Figure 13: Quenching with lutidinium ethyl bromide

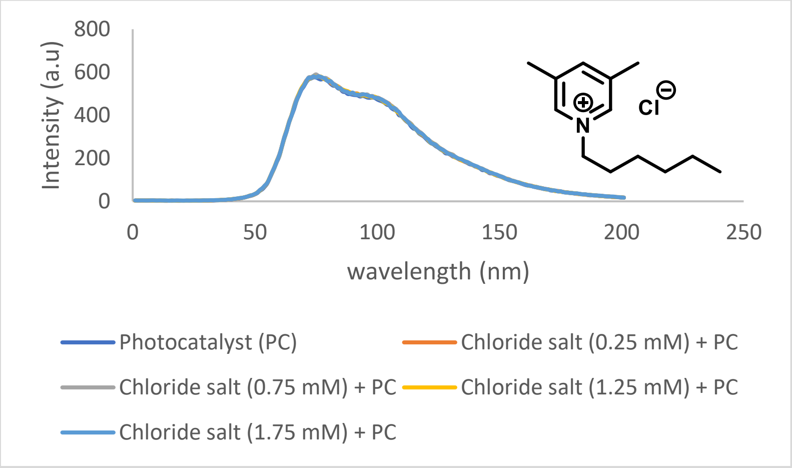

Figure 14: Quenching with lutidinium hexyl chloride

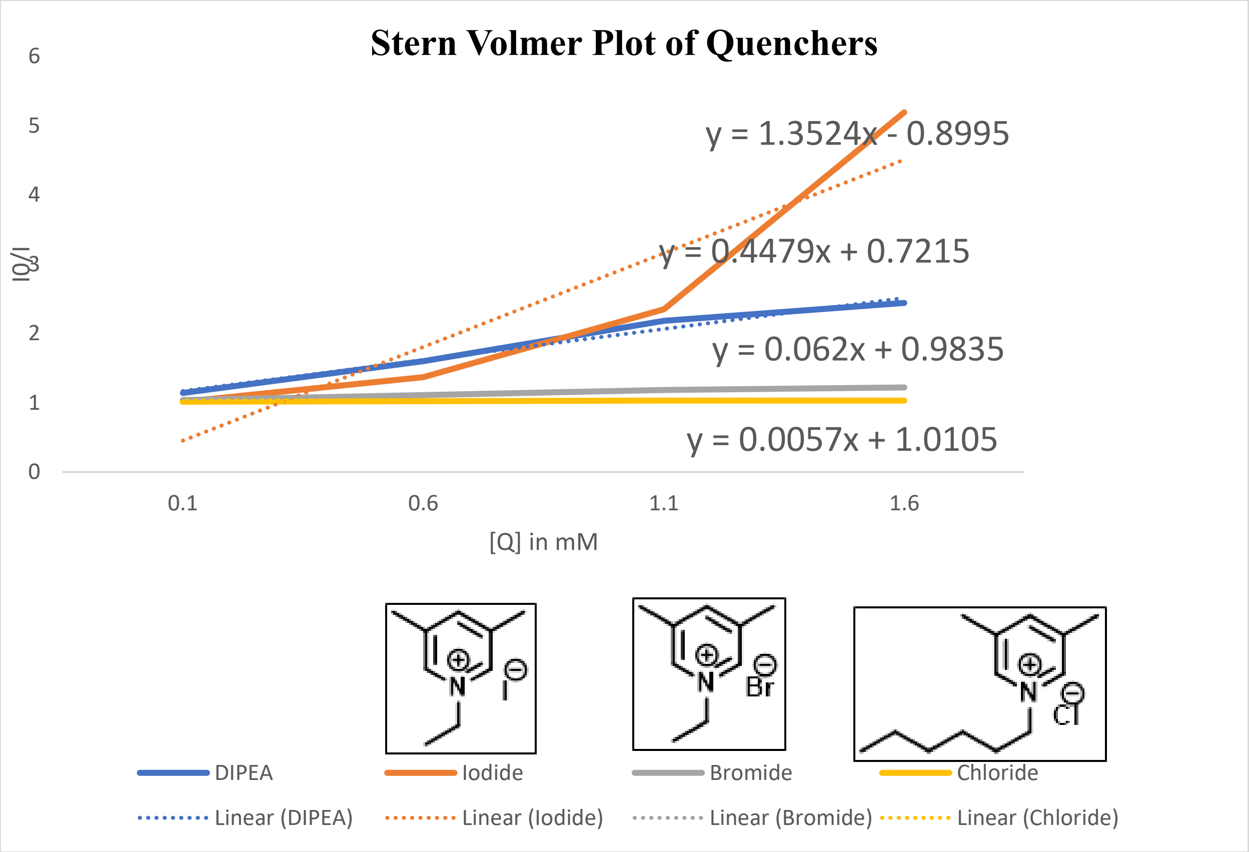

	DIPEA	Iodide	Bromide	Chloride
K_{sv} [in mM^{-1}]	0.4479	1.3524	0.062	0.0057

Figure 15: Plot and linear regression of I0/I for each quencher vs concentration

We have observed significant quenching of the photocatalyst with both DIPEA and lutidinium ethyl iodide.

F8: Different amines than DIPEA

A) Reaction with tetramethylpiperidine

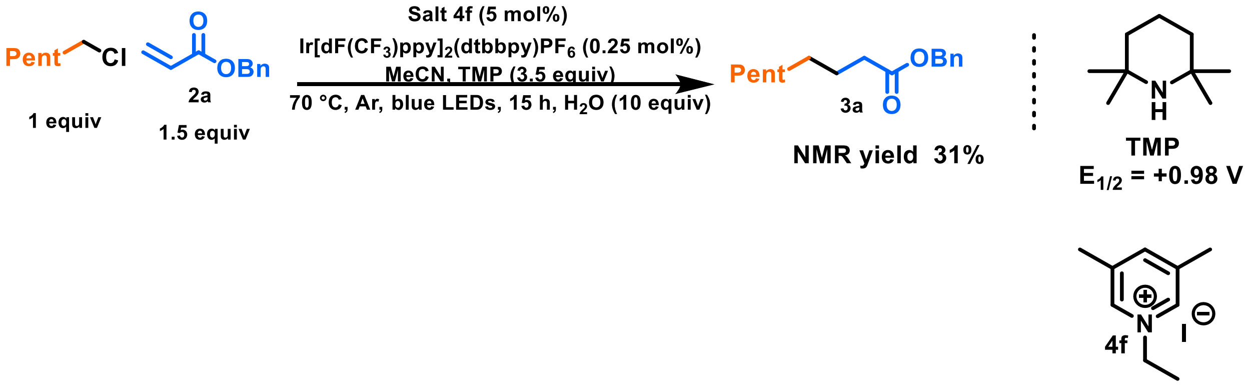

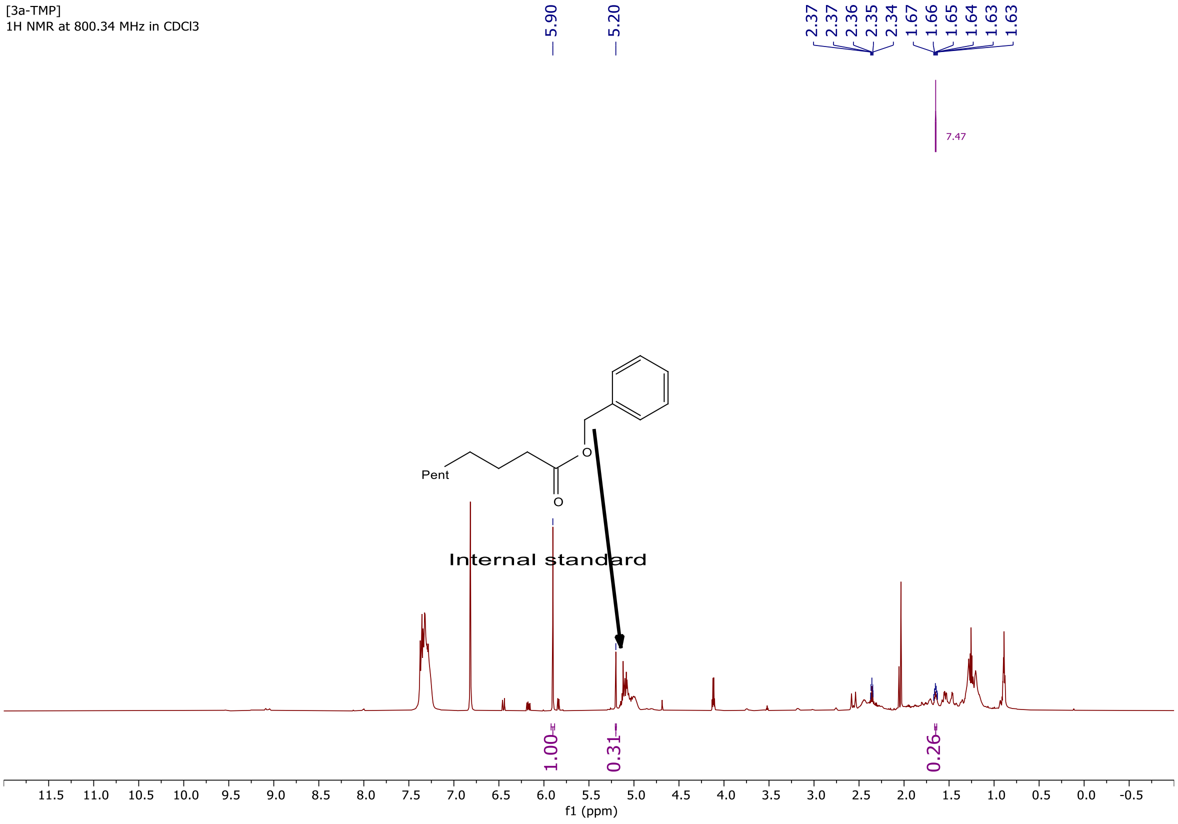

1,3-benzodioxole is used as an internal standard

B) Reaction with DABCO

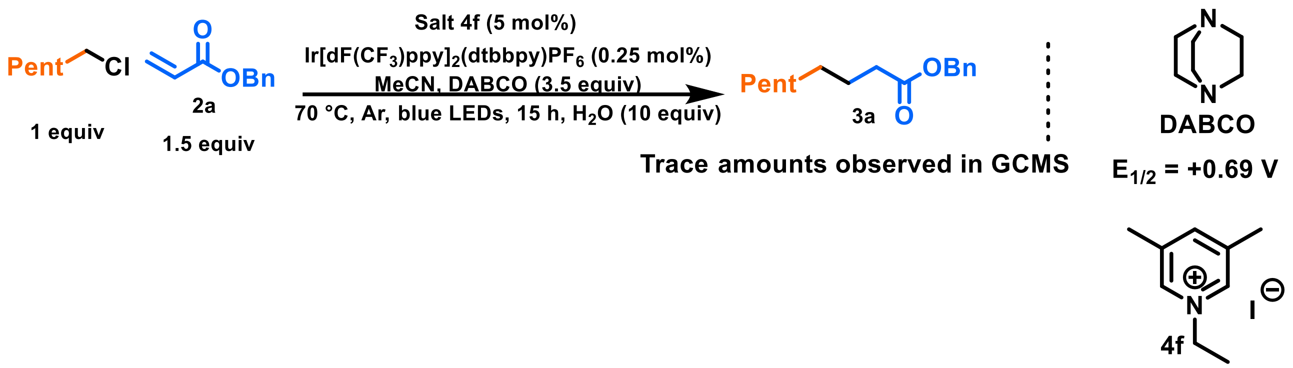

F9:Reaction with Aryl-X

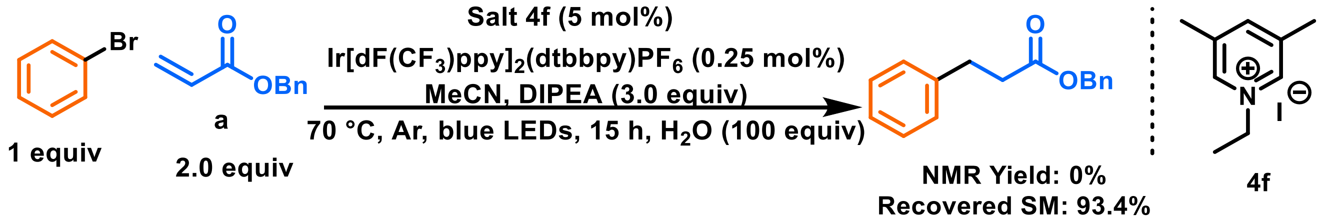

F10:Reaction with Aryl-X and alkyl chloride

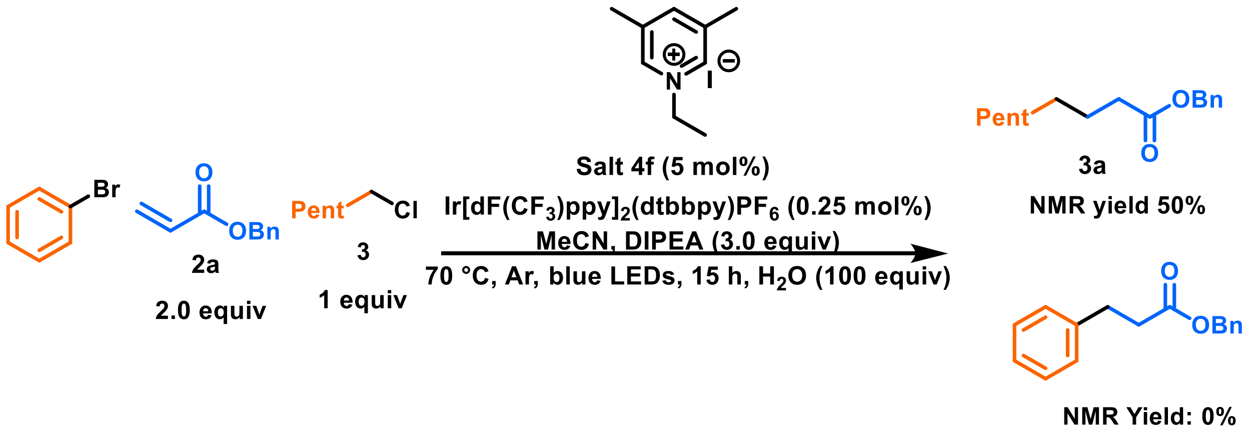

When the standard reaction employing chlorohexane was conducted in the presence of an aryl bromide, the yield of the desired product was reduced to 50%, and no Giese-type product formation involving the aryl bromide was observed.

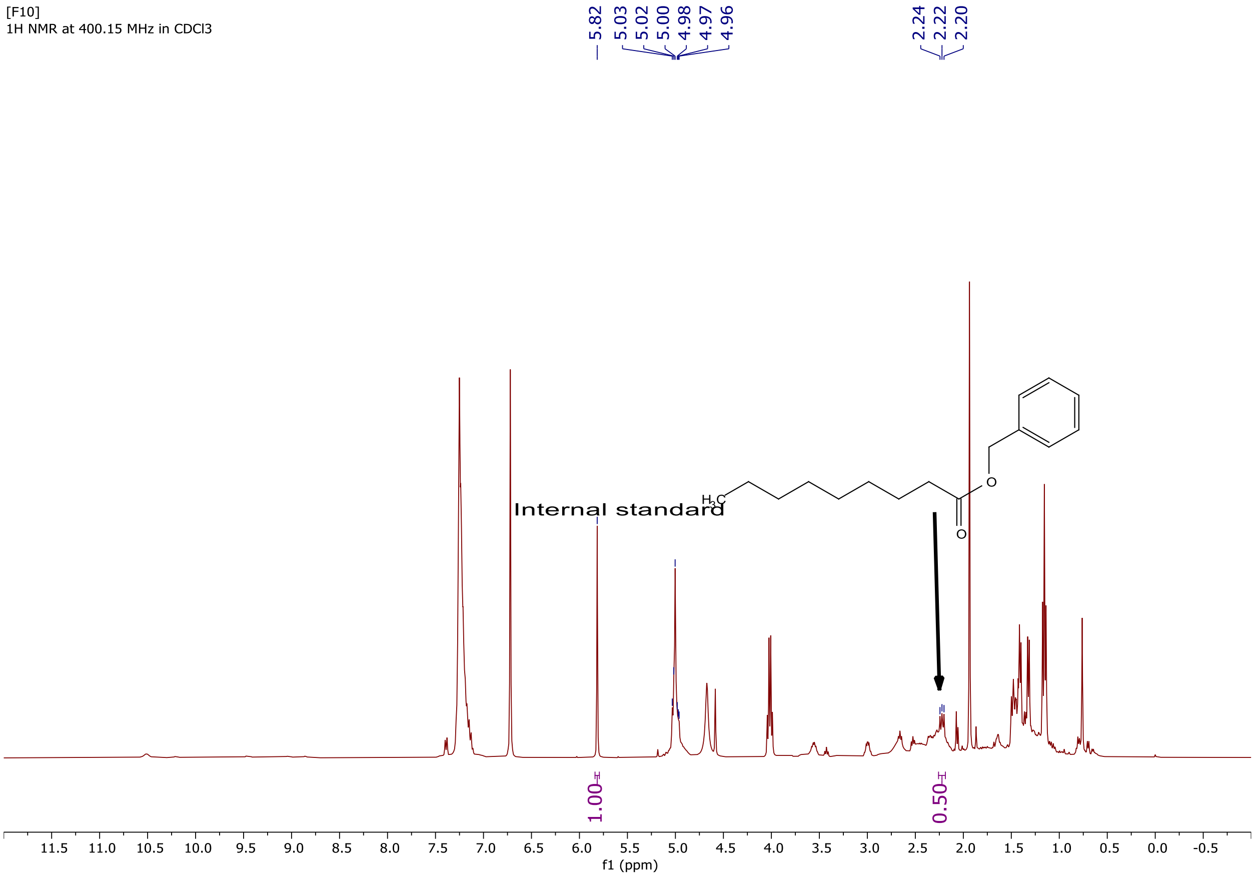
[F10]
1H NMR at 400.15 MHz in CDCl3
5.82
5.03
5.02
5.00
4.98
4.97
4.96
2.24
2.22
2.20
Internal standard
H3C
O
O
1.00
0.50
11.5 11.0 10.5 10.0 9.5 9.0 8.5 8.0 7.5 7.0 6.5 6.0 5.5 5.0 4.5 4.0 3.5 3.0 2.5 2.0 1.5 1.0 0.5 0.0 -0.5
f1 (ppm)

G: Synthesis of Substrates

19: 2-bromoethyl 2-acetoxybenzoate

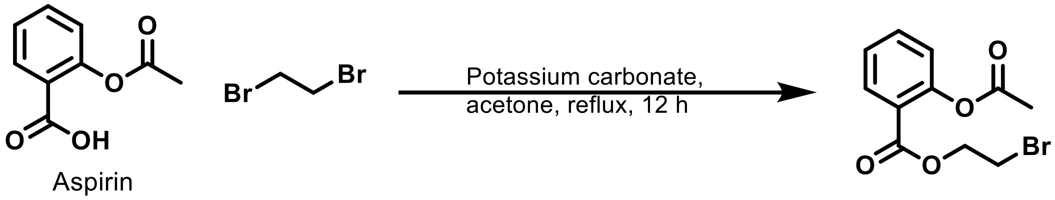

To prepare 2-bromoethyl 2-acetoxybenzoate, an aspirin tablet (Equate brand, one tablet, 325 mg) was reacted with dibromoethane (0.15 mmol, 28.2 mg, 1.3 equiv, 13.0 µL), potassium carbonate (0.15 mmol, 21.5 mg, 1.3 equiv), and acetone (15 mL). The reaction mixture was refluxed at 70 °C for 24 hours and refluxed overnight at 75 °C. After the reaction period, the mixture was allowed to cool to room temperature and was concentrated to dryness using a rotary evaporator. The resulting solid was dissolved in ethyl acetate and washed sequentially with water and a saturated sodium chloride solution. The organic phase was then dried over anhydrous magnesium sulfate ($MgSO_4$), filtered, and concentrated under reduced pressure. The oily residue obtained was purified via automated flash chromatography, using a gradient of ethyl acetate in hexanes (0% to 100%). The product, 2-bromoethyl 2-acetoxybenzoate, was eluted at 3.0% ethyl acetate on a 4 g silica column, yielding the product as an oil (150 mg, 0.5 mmol). **^{1}H NMR** (800 MHz, $CDCl_3$) δ 8.06 (ddt, *J* = 7.9, 3.3, 1.6 Hz, 1H), 7.60 – 7.55 (m, 1H), 7.35 – 7.29 (m, 1H), 7.12 (ddt, *J* = 8.1, 2.8, 1.1 Hz, 1H), 4.57 (qd, *J* = 6.1, 1.5 Hz, 2H), 3.63 – 3.58 (m, 2H), 2.37 – 2.35 (m, 3H). **^{13}C NMR** (201 MHz, $CDCl_3$) δ 169.7, 163.8, 150.9, 134.3, 131.9, 126.1, 123.9, 122.7, 64.4, 28.5, 21.1.

36: Ethyl-4-methylbenzenesulfonate

To synthesize ethyl-4-methylbenzenesulfonate, dissolve 4-toluenesulfonyl chloride (10.36 g, 0.054 mol) in 150 mL of dichloromethane. Separately, prepare a solution of ethanol (2.48 g, 0.054 mol) and triethylamine (15 mL, 0.108 mol) in 20 mL of dichloromethane. Under a nitrogen atmosphere, add the ethanol/triethylamine solution dropwise to the 4-toluenesulfonyl chloride solution at 0°C, noting the evolution of a white gas during the process. Stir the reaction mixture at room temperature overnight. Upon completion, transfer the reaction mixture to a separatory funnel and add 50 mL of water. Extract the aqueous layer with dichloromethane, combine the organic layers, and wash them sequentially with 50 mL of 3M HCl, 50 mL of sodium bicarbonate solution, and 50 mL of water. Dry the organic phase over magnesium sulfate, then remove the solvent under reduced pressure to obtain the product, ethyl-4-methylbenzenesulfonate in a quantitative yield. **^{1}H NMR** (400 MHz, $CDCl_3$) δ 7.80 (d, *J* = 8.0 Hz, 2H), 7.34 (d, *J* = 8.0 Hz, 2H), 4.11 (q, *J* = 7.1 Hz, 2H), 2.45 (s, 3H), 1.30 (t, *J* = 7.1 Hz, 3H). NMR chemical shifts match with the literature value.[7]

Characterization of Products

H1: Alkyl Halides Substrate Characterization

H1.1: Primary Halides

General procedure C

Three NMR tubes fitted with a rubber septum were charged with a solution of [Ir(2′,4′-dF-5-CF_3-ppy)$_2$(4,4′-dtbbpy)]PF_6 (0.25 mM, 0.5mL in MeCN), alkyl bromide (0.12 mmol, 1 equiv) or alkyl chloride (0.12 mmol, 1 equiv), lutidinium iodide salt (**4l**/**4f**) (5 mol%), benzyl acrylate (2 or 3 equiv), DIPEA (3 or 3.5 equiv), DI water (100 or 50 equiv). Then, the reaction mixture was degassed via Ar bubbling for 15 min and then left under positive Ar pressure by removing the exit needle. The tubes were placed in a light bath (description above) which was maintained at 70 °C. The reaction was monitored by 1H or 19F NMR. After the complete consumption of alkyl halide, the volatiles (MeCN and some DIPEA) were removed via rotovap, and the residue was dissolved in ethyl acetate (12 mL) and washed with 1M aqueous HCl solution (3 x 4 mL) and brine solution (6 mL) thrice. The organic layer was dried over anhydrous $MgSO_4$ and filtered. The crude product was concentrated in vacuo and purified via normal-phase chromatography.

3a: benzyl nonanoate

3a

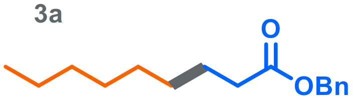

Br, 97%

Following the general procedure C, benzyl nonanoate was isolated using 1-bromohexane (0.12 mmol, 19.8 mg, 16.8 µL, 1 equiv), benzyl acrylate (0.24 mmol, 38.9 mg, 2.0 equiv, 36.5 µL), DIPEA (0.36 mmol, 46.5 mg, 62.7 µL, 3.0 equiv), DI water (12 mmol, 216 mg, 216 µL, 100 equiv), 2-isopropyl-3,5-lutidinium iodide (5.4 µmol, 1.5 mg, 0.05 equiv) and 0.5 mL of stock solution of [Ir(2′,4′-dF-5-CF_3-ppy)$_2$(4,4′-dtbbpy)]PF_6 in MeCN. After the completion of the reaction 15 h, the crude was purified via automated flash chromatography using EtOAc in hexanes (0% to 100%) with product eluting at 1.0% on a 4 g silica column to afford it in 97 % yield (28.8 mg, 0.11 mmol) as an oil. **^{1}H NMR** (800 MHz, $CDCl_3$) δ 7.41 – 7.32 (m, 5H), 5.14 (s, 2H), 2.37 (t, J = 7.6 Hz, 2H), 1.66 (p, J = 7.5 Hz, 2H), 1.31 – 1.27 (m, 10H), 0.90 (t, J = 7.2 Hz, 3H). **^{13}C NMR** (201 MHz, $CDCl_3$) δ 173.7, 136.2, 128.6, 128.2, 128.2, 66.1, 34.4, 31.8, 29.2, 29.1, 25.0, 22.6, 14.1. NMR chemical shifts match with the literature value.[8]

3a: benzyl nonanoate

3a

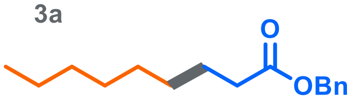

Cl, 90%

Following the general procedure C, benzyl nonanoate was isolated using 1-chlorohexane (0.12 mmol, 14.5 mg, 16.4 µL, 1 equiv), benzyl acrylate (0.36 mmol, 58 mg, 3.0 equiv, 54.7 µL), DIPEA (0.42 mmol, 54.3 mg, 73.2 µL, 3.5 equiv), DI water (1.2 mmol, 21.6 mg, 21.6 µL, 10 equiv), ethyl-3,5-lutidinium iodide (6.1 µmol, 1.6 mg, 0.05 equiv)and 0.5 mL of stock solution of [Ir(2′,4′-dF-5-CF_3-ppy)$_2$(4,4′-dtbbpy)]PF_6 in MeCN. After the completion of the reaction 15 h, the crude was purified via automated flash chromatography using EtOAc in hexanes (0% to 100%) with product eluting at 1.0% on a 4 g silica column to afford it in 90% yield (26.8 mg, 0.10 mmol) as an oil. **^{1}H NMR** (800 MHz, $CDCl_3$) δ 7.41 – 7.32 (m, 5H), 5.14 (s, 2H), 2.37 (t, J = 7.6 Hz, 2H), 1.66 (p, J = 7.5 Hz, 2H), 1.31 – 1.27 (m, 10H), 0.90 (t, J = 7.2 Hz, 3H). **^{13}C NMR** (201 MHz, $CDCl_3$) δ 173.7, 136.2, 128.6, 128.2, 128.2, 66.1, 34.4, 31.8, 29.2, 29.1, 25.0, 22.6, 14.1. NMR chemical shifts match with the literature value.[8]

5a: benzyl 5-oxohexanoate

5a

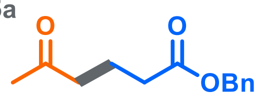

Cl, 80%

Following the general procedure C, benzyl 5-oxohexanoate was isolated using 1-chloropropan-2-one (0.12 mmol, 11.1 mg, 9.5 µL, 1 equiv), benzyl acrylate (0.36 mmol, 58 mg, 3.0 equiv, 54.7 µL), DIPEA (0.42 mmol, 54.3 mg, 73.2 µL, 3.5 equiv), DI water (1.2 mmol, 21.6 mg, 21.6 µL, 10 equiv), ethyl-3,5-lutidinium iodide (6.1 µmol, 1.6 mg, 0.05 equiv)and 0.5 mL of stock solution of [Ir(2′,4′-dF-5-CF_3-ppy)$_2$(4,4′-dtbbpy)]PF_6 in MeCN. After the completion of the reaction 15 h, the crude was purified via automated flash chromatography using EtOAc in hexanes (0% to 100%) with product eluting at 1.0% on a 4 g silica column to afford it in 80% yield (21.1 mg, 0.09 mmol) as an oil. **^{1}H NMR** (800 MHz, $CDCl_3$) δ 7.40 – 7.36 (m, 5H), 5.14 (s, 2H), 2.51 (t, J = 7.2 Hz, 2H), 2.41 (t, J = 7.2 Hz, 2H), 2.14 (s, 3H), 1.93 (p, J = 7.2 Hz, 2H). **^{13}C NMR** (201 MHz, $CDCl_3$) δ 207.96, 172.99, 135.95, 128.59, 128.27, 128.25, 66.26, 42.40, 33.23, 29.94, 18.87. NMR chemical shifts match with the literature value.[6]

6a: benzyl methyl glutarate

6a

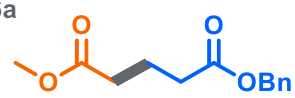

Br, 71%

Following the general procedure C, benzyl methyl glutarate was isolated using 2-bromoethyl methyl ether (0.12 mmol, 16.6 mg, 11.2 µL, 1 equiv), benzyl acrylate (0.24 mmol, 38.9 mg, 2.0 equiv, 36.5 µL), DIPEA (0.36 mmol, 46.5 mg, 62.7 µL, 3.0 equiv), DI water (12 mmol, 216 mg, 216 µL, 100 equiv), 2-isopropyl-3,5-lutidinium iodide (5.4 µmol, 1.5 mg, 0.05 equiv)and 0.5 mL of stock solution of [Ir(2′,4′-dF-5-CF_3-ppy)$_2$(4,4′-dtbbpy)]PF_6 in MeCN. After the completion of the reaction 15 h, the crude was purified via automated flash chromatography using EtOAc in hexanes (0% to 100%) with product eluting at 1.0% on a 4 g silica column to afford it in 71% yield (20.1 mg, 0.08 mmol) as an oil. **^{1}H NMR** (400 MHz, $CDCl_3$) δ 7.42 – 7.29 (m, 5H), 5.12 (s, 2H), 3.67 (s, 3H), 2.41 (dt, J = 19.7, 7.4 Hz, 4H), 1.98 (p, J = 7.3 Hz, 2H). **^{13}C NMR** (101 MHz, $CDCl_3$) δ 173.5, 172.9, 136.1, 128.7, 128.4, 128.4, 66.4, 51.8, 33.4, 33.2, 20.3. NMR chemical shifts match with the literature value.[9]

7a: benzyl 7-methoxyheptanoate

7a

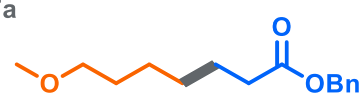

Cl, 70%

Following the general procedure C, benzyl 7-methoxyheptanoate was isolated using methyl 2-chloroacetate (0.12 mmol, 14.7 mg, 1 equiv), benzyl acrylate (0.36 mmol, 58 mg, 3.0 equiv, 54.7 µL), DIPEA (0.42 mmol, 54.3 mg, 73.2 µL, 3.5 equiv), DI water (1.2 mmol, 21.6 mg, 21.6 µL, 10 equiv), ethyl-3,5-lutidinium iodide (6.1 µmol, 1.6 mg, 0.05 equiv)and 0.5 mL of stock solution of [Ir(2′,4′-dF-5-CF_3-ppy)$_2$(4,4′-dtbbpy)]PF_6 in MeCN. After the completion of the reaction 15 h, the crude was purified via automated flash chromatography using EtOAc in hexanes (0% to 100%) with product eluting at 1.0% on a 4 g silica column to afford it in 70% yield (21 mg, 0.08 mmol) as an oil. **^{1}H NMR** (400 MHz, $CDCl_3$) δ 7.34 – 7.21 (m, 5H), 5.04 (s, 2H), 3.31 – 3.23 (m, 5H), 2.29 (t, J = 7.5 Hz, 2H), 1.59 (dd, J = 9.8, 4.8 Hz, 2H), 1.51 – 1.45 (m, 3H), 1.27 (p, J = 3.4 Hz, 3H). **^{13}C NMR** (201 MHz, $CDCl_3$) δ 173.6, 136.1, 128.6, 128.2, 72.8, 66.3, 66.1, 58.6, 34.3, 29.7, 29.5, 25.8, 24.9. HRMS (ESI) m/z: [M+H] + calcd for $C_{15}H_{22}O_3H$ 251.1642; found 251.1642.

7a: benzyl 7-methoxyheptanoate

7a

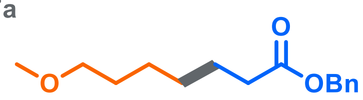

Br, 73%

Following the general procedure C, benzyl 7-methoxyheptanoate was isolated using methyl 2-bromoacetate (0.12 mmol, 18.8 mg, 11.3 µL, 1 equiv), benzyl acrylate (0.24 mmol, 38.9 mg, 2.0 equiv, 36.5 µL), DIPEA (0.36 mmol, 46.5 mg, 62.7 µL, 3.0 equiv), DI water (12 mmol, 216 mg, 216 µL, 100 equiv), 2-isopropyl-3,5-lutidinium iodide (6.1 µmol, 1.5 mg, 0.05 equiv) and 0.5 mL of stock solution of [Ir(2′,4′-dF-5-CF_3-ppy)$_2$(4,4′-dtbbpy)]PF_6 in MeCN. After the completion of the reaction 15 h, the crude was purified via automated flash chromatography using EtOAc in hexanes (0% to 100%) with product eluting at 1.0% on a 4 g silica column to afford it in 73% yield (21.9 mg, 0.08 mmol) as an oil. **^{1}H NMR** (400 MHz, $CDCl_3$) δ 7.34 – 7.21 (m, 5H), 5.04 (s, 2H), 3.31 – 3.23 (m, 5H), 2.29 (t, J = 7.5 Hz, 2H), 1.59 (dd, J = 9.8, 4.8 Hz, 2H), 1.51 – 1.45 (m, 3H), 1.27 (p, J = 3.4 Hz, 3H). **^{13}C NMR** (201 MHz, $CDCl_3$) δ 173.6, 136.1, 128.6, 128.2, 72.8, 66.3, 66.1, 58.6, 34.3, 29.7, 29.5, 25.8, 24.9. HRMS (ESI) m/z: [M+H] + calcd for $C_{15}H_{22}O_3H$ 251.1642; found 251.1642.

8a: benzyl 8-hydroxyoctanoate

8a

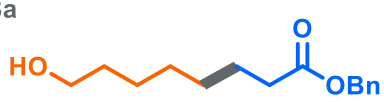

Br, 79%

Following the general procedure C, benzyl 8-hydroxyoctanoate was isolated using 5-bromopentan-1-ol (0.12 mmol, 20.0 mg, 1 equiv), benzyl acrylate (0.24 mmol, 38.9 mg, 2.0 equiv, 36.5 µL), DIPEA (0.36 mmol, 46.5 mg, 62.7 µL, 3.0 equiv), DI water (12 mmol, 216 mg, 216 µL, 100 equiv), 2-isopropyl-3,5-lutidinium iodide (5.4 µmol, 1.5 mg, 0.05 equiv)and 0.5 mL of stock solution of [Ir(2′,4′-dF-5-CF_3-ppy)$_2$(4,4′-dtbbpy)]PF_6 in MeCN. After the completion of the reaction 15 h, the crude was purified via automated flash chromatography using EtOAc in hexanes (0% to 100%) with product eluting at 1.0% on a 4 g silica column to afford it in 79% yield (23.7 mg, 0.09 mmol) as an oil. **^{1}H NMR** (400 MHz, $CDCl_3$) δ 7.37 – 7.31 (m, 5H), 5.11 (s, 2H), 3.63 (t, J = 6.6 Hz, 2H), 2.36 (t, J = 7.5 Hz, 2H), 1.71 – 1.49 (m, 8H).**^{13}C NMR** (201 MHz, $CDCl_3$) δ 173.8, 136.3, 128.7, 128.4, 128.3, 66.2, 63.1, 34.4, 32.8, 29.2, 29. (ESI) m/z: [M+H] + calcd for $C_{15}H_{22}O_3H$ 251.1642; found 251.1685.

9a: benzyl undecanoate

9a

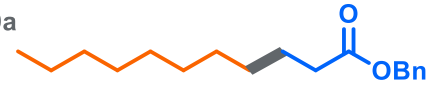

Br, 96%

Following the general procedure C, benzyl undecanoate was isolated using 1-bromooctane (0.12 mmol, 23.1 mg, 20.7 µL, 1 equiv), benzyl acrylate (0.24 mmol, 38.9 mg, 2.0 equiv, 36.5 µL DIPEA (0.36 mmol, 46.5 mg, 62.7 µL, 3.0 equiv), DI water (12 mmol, 216 mg, 216 µL, 100 equiv), 2-isopropyl-3,5-lutidinium iodide (5.4 µmol, 1.5 mg, 0.05 equiv)and 0.5 mL of stock solution of [Ir(2′,4′-dF-5-CF_3-ppy)$_2$(4,4′-dtbbpy)]PF_6 in MeCN. After the completion of the reaction 15 h, the crude was purified via automated flash chromatography using EtOAc in hexanes (0% to 100%) with product eluting at 1.0% on a 4 g silica column to afford it in 96% yield (31.8 mg, 0.11 mmol) as an oil. **^{1}H NMR** (400 MHz, $CDCl_3$) δ 7.34 – 7.20 (m, 5H), 5.04 (s, 2H), 2.28 (t, J = 7.6 Hz, 2H), 1.57 (p, J = 7.4 Hz, 2H), 1.20 (s, 9H), 1.18 (s, 5H), 0.83 – 0.77 (m, 3H). **^{13}C NMR** (201 MHz, $CDCl_3$) δ 173.7, 136.2, 128.6, 128.2, 128.2, 66.1, 34.4, 31.8, 29.2, 29.1, 29.1, 29.1, 29.1, 25.0, 22.6, 14.1.NMR chemical shifts match with the literature value.[10]

10a: benzyl 6,6,6-trifluorohexanoate

10a

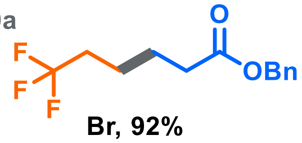

Br, 92%

Following the general procedure C, benzyl 6,6,6-trifluorohexanoate was isolated using 3-bromo-1,1,1-trifluoropropane (0.12 mmol, 21.3 mg, 17.4 µL, 1 equiv), benzyl acrylate (0.24 mmol, 38.9 mg, 2.0 equiv, 36.5 µL DIPEA (0.36 mmol, 46.5 mg, 62.7 µL, 3.0 equiv), DI water (12 mmol, 216 mg, 216 µL, 100 equiv), 2-isopropyl-3,5-lutidinium iodide (5.4 µmol, 1.5 mg, 0.05 equiv) and 0.5 mL of stock solution of [Ir(2′,4′-dF-5-CF_3-ppy)$_2$(4,4′-dtbbpy)]PF_6 in MeCN. After the completion of the reaction 15 h, the crude was purified via automated flash chromatography using EtOAc in hexanes (0% to 100%) with product eluting at 1.0% on a 4 g silica column to afford it in 92% yield (18.6 mg, 0.11 mmol) as an oil.^{1}**H NMR** (400 MHz, $CDCl_3$) δ 7.34 – 7.21 (m, 5H), 5.05 (s, 2H), 2.32 (t, *J* = 7.3 Hz, 2H), 2.05 – 1.94 (m, 2H), 1.65 (p, *J* = 7.3 Hz, 2H), 1.58 – 1.43 (m, 2H). ^{13}**C NMR** (201 MHz, $CDCl_3$) δ 173.0, 136.0, 128.7, 128.4, 128.4, 127.1 (q, *J* = 277.1 Hz), 99.9, 66.5, 33.9, 33.6 (q, *J* = 28.9 Hz), 21.6 (q, *J* = 2.6 Hz).^{19}**F NMR** (376 MHz, $CDCl_3$) δ -66.4 (t, *J* = 10.9 Hz). HRMS (ESI) m/z: [M+H] + calcd for $C_{13}H_{15}O_2F_3H$ 261.1097; found 261.1010.

11a: benzyl 5-methoxypentanoate

11a

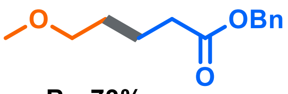

Br, 73%

Following the general procedure C, benzyl 5-methoxypentanoate was isolated using 1-bromo-2-methoxyethane (0.12 mmol, 16.6 mg, 11.2 µL, 1 equiv), benzyl acrylate (0.24 mmol, 38.9 mg, 2.0 equiv, 36.5 µL), DIPEA (0.36 mmol, 46.5 mg, 62.7 µL, 3.0 equiv), DI water (12 mmol, 216 mg, 216 µL, 100 equiv), 2-isopropyl-3,5-lutidinium iodide (5.4 µmol, 1.5 mg, 0.05 equiv) and 0.5 mL of stock solution of [Ir(2′,4′-dF-5-CF_3-ppy)$_2$(4,4′-dtbbpy)]PF_6 in MeCN. After the completion of the reaction 15 h, the crude was purified via automated flash chromatography using EtOAc in hexanes (0% to 100%) with product eluting at 1.0% on a 4 g silica column to afford it in 73% yield (19.4 mg, 0.08 mmol) as an oil. ^{1}**H NMR** (400 MHz, $CDCl_3$) δ 7.43 – 7.28 (m, 5H), 5.11 (s, 2H), 3.37 (t, *J* = 6.3 Hz, 2H), 3.31 (s, 3H), 2.39 (t, *J* = 7.4 Hz, 2H), 1.78 – 1.65 (m, 2H), 1.60 (dp, *J* = 12.8, 6.9 Hz, 2H). ^{13}**C NMR** (101 MHz, $CDCl_3$) δ 136.2, 128.7, 128.3, 72.4, 66.3, 58.7, 34.2, 29.1, 21.8, (missing C between 128). HRMS (ESI) m/z: [M+H] + calcd for $C_{13}H_{18}O_3H$ 223.1329; found 223.1324.

12a: benzyl palmitate

12a

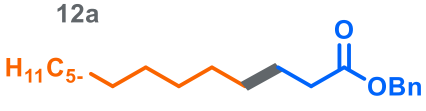

Br, 81 %

Following the general procedure C, benzyl palmitate was isolated using 1-bromotridecane (0.12 mmol, 31.5 mg, 30.6 µL, 1 equiv), benzyl acrylate (0.24 mmol, 38.9 mg, 2.0 equiv, 36.5 µL), DIPEA (0.36 mmol, 46.5 mg, 62.7 µL, 3.0 equiv), DI water (12 mmol, 216 mg, 216 µL, 100 equiv), 2-isopropyl-3,5-lutidinium iodide (5.4 µmol, 1.5 mg, 0.05 equiv) and 0.5 mL of stock solution of [Ir(2′,4′-dF-5-CF_3-ppy)$_2$(4,4′-dtbbpy)]PF_6 in MeCN. After the completion of the reaction 15 h, the crude was purified via automated flash chromatography using EtOAc in hexanes (0% to 100%) with product eluting at 1.0% on a 4 g silica column to afford it in 81% yield (33.6 mg, 0.09 mmol) as an oil. ^{1}**H NMR** ^{1}H NMR (400 MHz, $CDCl_3$) δ 7.34 – 7.20 (m, 5H), 5.04 (s, 2H), 2.28 (t, *J* = 7.5 Hz, 2H), 1.56 (q, *J* = 7.2 Hz, 2H), 1.18 (d, *J* = 2.6 Hz, 19H), 0.81 (t, *J* = 6.7 Hz, 3H). ^{13}**C NMR**[1] ^{13}C NMR (201 MHz, $CDCl_3$) δ 173.7, 136.2, 128.6, 128.2, 128.2, 66.1, 44.8, 34.4, 29.7, 29.7, 29.6, 29.6, 29.5, 29.5, 29.3, 29.1, 25.0, 14.1.NMR chemical shifts match with the literature value.[11]

13a: benzyl 5-(1,3-dioxolan-2-yl) pentanoate

13a

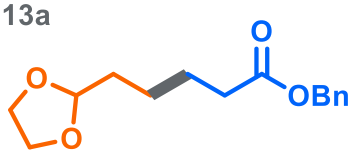

Br, 90%

Following the general procedure C, benzyl 5-(1,3-dioxolan-2-yl)pentanoate was isolated using 2-(2-bromoethyl)-1,3-dioxolane (0.12 mmol, 21.7 mg, 14.8 µL, 1 equiv), benzyl acrylate (0.24 mmol, 38.9 mg, 2.0 equiv, 36.5 µL), DIPEA (0.36 mmol, 46.5 mg, 62.7 µL, 3.0 equiv), DI water (12 mmol, 216 mg, 216 µL, 100 equiv), 2-isopropyl-3,5-lutidinium iodide (5.4 µmol, 1.5 mg, 0.05 equiv) and 0.5 mL of stock solution of [Ir(2′,4′-dF-5-CF_3-ppy)$_2$(4,4′-dtbbpy)]PF_6 in MeCN. After the completion of the reaction 15 h, the crude was purified via automated flash chromatography using EtOAc in hexanes (0% to 100%) with product eluting at 1.0% on a 4 g silica column to afford it in 90% yield (28.5 mg, 0.11 mmol) as an oil. **^{1}H NMR** (800 MHz, $CDCl_3$) δ 7.41 – 7.36 (m, 4H), 7.34 (dddd, *J* = 8.6, 6.2, 5.5, 2.4 Hz, 1H), 5.14 (s, 2H), 4.86 (t, *J* = 4.8 Hz, 1H), 4.00 – 3.94 (m, 2H), 3.89 – 3.83 (m, 2H), 2.40 (t, *J* = 7.5 Hz, 2H), 1.76 – 1.67 (m, 4H), 1.51 – 1.45 (m, 2H). **^{13}C NMR** (201 MHz, $CDCl_3$) δ 173.4, 136.1, 128.6, 128.4, 128.2, 104.3, 66.1, 64.9, 34.2, 33.5, 24.8, 23.6. HRMS (ESI) m/z: [M+2H_2O+H] + calcd for $C_{15}H_{20}O_4$ 301.1646; found 301.1411.

14a: benzyl 6-chlorohexanoate

14a

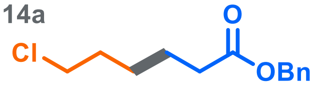

Br, 84%

Following the general procedure C, benzyl 6-chlorohexanoate was isolated using 1-bromo-3-chloropropane (0.12 mmol, 18.8 mg, 11.8 µL, 1 equiv), benzyl acrylate (0.24 mmol, 38.9 mg, 2.0 equiv, 36.5 µL), DIPEA (0.36 mmol, 46.5 mg, 62.7 µL, 3.0 equiv), DI water (12 mmol, 216 mg, 216 µL, 100 equiv), 2-isopropyl-3,5-lutidinium iodide (5.4 µmol, 1.5 mg, 0.05 equiv) and 0.5 mL of stock solution of [Ir(2′,4′-dF-5-CF_3-ppy)$_2$(4,4′-dtbbpy)]PF_6 in MeCN. After the completion of the reaction 15 h, the crude was purified via automated flash chromatography using EtOAc in hexanes (0% to 100%) with product eluting at 1.0% on a 4 g silica column to afford it in 84% yield (24.2 mg, 0.10 mmol) as an oil. **^{1}H NMR** (400 MHz, $CDCl_3$) δ 7.42 – 7.28 (m, 5H), 5.12 (s, 2H), 3.52 (t, *J* = 6.6 Hz, 2H), 2.38 (t, *J* = 7.4 Hz, 2H), 1.82 – 1.73 (m, 2H), 1.68 (p, *J* = 7.4 Hz, 2H), 1.52 – 1.42 (m, 2H). **^{13}C NMR** (201 MHz, $CDCl_3$) δ 173.4, 136.1, 128.7, 128.4, 128.4, 66.3, 44.9, 34.2, 34.2, 32.4, 24.3.NMR chemical shifts match with the literature value.[12]

15a: benzyl 7-cyanoheptanoate

15a

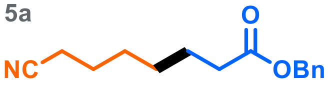

Br, 74%

Following the general procedure C, benzyl 7-cyanoheptanoate was isolated using 5-bromopentanenitrile (0.12 mmol, 19.4 mg, 14.0 µL, 1 equiv), benzyl acrylate (0.24 mmol, 38.9 mg, 2.0 equiv, 36.5 µL), DIPEA (0.36 mmol, 46.5 mg, 62.7 µL, 3.0 equiv), DI water (12 mmol, 216 mg, 216 µL, 100 equiv), 2-isopropyl-3,5-lutidinium iodide (5.4 µmol, 1.5 mg, 0.05 equiv) and 0.5 mL of stock solution of [Ir(2′,4′-dF-5-CF_3-ppy)$_2$(4,4′-dtbbpy)]PF_6 in MeCN. After the completion of the reaction 15 h, the crude was purified via automated flash chromatography using EtOAc in hexanes (0% to 100%) with product eluting at 1.0% on a 4 g silica column to afford it in 74% yield (21.7 mg, 0.08 mmol) as an oil. **^{1}H NMR** (400 MHz, $CDCl_3$) δ 7.30 – 7.26 (m, 5H), 5.05 (s, 2H), 2.30 (t, *J* = 7.4 Hz, 2H), 2.24 (t, *J* = 7.1 Hz, 2H), 1.58 (h, *J* = 7.4 Hz, 4H), 1.44 – 1.34 (m, 2H), 1.33 – 1.24 (m, 2H). **^{13}C NMR** (201 MHz, $CDCl_3$) δ 173.5, 136.2, 128.7, 128.4, 128.3, 119.8, 66.3, 34.2, 28.5, 28.3, 25.3, 24.7, 17.2. HRMS (ESI) m/z: [M+H] + calcd for $C_{15}H_{19}O_2NH$ 246.1489; found 246.1486.

16a: benzyl 5,5,5-trifluoropentanoate

16a

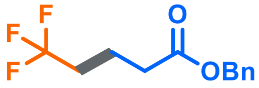

Br, 76%

Following the general procedure C, benzyl 5-(1,3-dioxolan-2-yl)pentanoate was isolated using 2-(2-bromoethyl)-1,3-dioxolane (0.12 mmol, 21.7 mg, 14.8 µL, 1 equiv), benzyl acrylate (0.24 mmol, 38.9 mg, 2.0 equiv, 36.5 µL), DIPEA (0.36 mmol, 46.5 mg, 62.7 µL, 3.0 equiv), DI water (12 mmol, 216 mg, 216 µL, 100 equiv), 2-isopropyl-3,5-lutidinium iodide (5.4 µmol, 1.5 mg, 0.05 equiv) and 0.5 mL of stock solution of [Ir(2′,4′-dF-5-CF_3-ppy)$_2$(4,4′-dtbbpy)]PF_6 in MeCN. After the completion of the reaction 15 h, the crude was purified via automated flash chromatography using EtOAc in hexanes (0% to 100%) with product eluting at 1.0% on a 4 g silica column to afford it in 90% yield (28.5 mg, 0.11 mmol) as an oil. **^{1}H NMR** (800 MHz, $CDCl_3$) δ 7.71 – 7.68 (m, 1H), 7.58 – 7.56 (m, 1H), 7.50 – 7.48 (m, 1H), 7.40 – 7.36 (m, 2H), 5.16 (s, 2H), 2.52 – 2.46 (m, 2H), 2.21 – 2.13 (m, 2H), 1.97 – 1.91 (m, 2H). **^{13}C NMR** (201 MHz, $CDCl_3$) δ 173.0, 136.0, 128.7, 128.4, 127.1 (q, J = 277.0 Hz), 66.5, 33.9, 33.6 (q, J = 28.9 Hz), 24.1, 21.6 (d, J = 2.8 Hz). **^{19}F NMR** (376 MHz, $CDCl_3$) δ -66.3 (t, J = 10.8 Hz). NMR chemical shifts match with the literature value.[12]

17a: benzyl 5-phenylpentanoate

17a

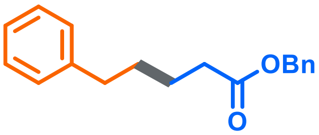

Cl, 86%

Following the general procedure C, benzyl 5-phenylpentanoate was isolated using (2-chloroethyl)benzene (0.12 mmol, 16 mg, 15.7 µL, 1 equiv), benzyl acrylate (0.36 mmol, 58 mg, 3.0 equiv, 54.7 µL), DIPEA (0.42 mmol, 54.3 mg, 73.2 µL, 3.5 equiv), DI water (1.2 mmol, 21.6 mg, 21.6 µL, 10 equiv), ethyl-3,5-lutidinium iodide (6.1 µmol, 1.6 mg, 0.05 equiv) and 0.5 mL of stock solution of [Ir(2′,4′-dF-5-CF_3-ppy)$_2$(4,4′-dtbbpy)]PF_6 in MeCN. After the completion of the reaction 15 h, the crude was purified via automated flash chromatography using EtOAc in hexanes (0% to 100%) with product eluting at 1.0% on a 4 g silica column to afford it in 86% yield (27 mg, 0.10 mmol) as an oil. **^{1}H NMR** (800 MHz, $CDCl_3$) δ 7.40 – 7.33 (m, 5H), 7.30 (d, J = 7.6 Hz, 2H), 7.21 – 7.16 (m, 3H), 5.13 (s, 2H), 2.64 (t, J = 7.5 Hz, 2H), 2.41 (t, J = 7.3 Hz, 2H), 1.75 – 1.64 (m, 4H). **^{13}C NMR** (201 MHz, $CDCl_3$) δ 173.4, 142.1, 136.0, 128.5, 128.5, 128.3, 128.3, 128.2, 125.7, 66.1, 35.5, 34.1, 30.8, 24.6. NMR chemical shifts match with the literature value.[12]

18a: benzyl 9-(9H-carbazol-9-yl)nonanoate

18a

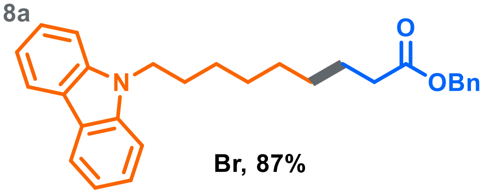

Br, 87%

Following the general procedure C, benzyl 9-(9H-carbazol-9-yl)nonanoate was isolated using 9-(6-bromohexyl)-9H-carbazole (0.12 mmol, 39.6 mg, 1 equiv), benzyl acrylate (0.24 mmol, 38.9 mg, 2.0 equiv, 36.5 µL), DIPEA (0.36 mmol, 46.5 mg, 62.7 µL, 3.0 equiv), DI water (12 mmol, 216 mg, 216 µL, 100 equiv), 2-isopropyl-3,5-lutidinium iodide (5.4 µmol, 1.5 mg, 0.05 equiv) and 0.5 mL of stock solution of [Ir(2′,4′-dF-5-CF_3-ppy)$_2$(4,4′-dtbbpy)]PF_6 in MeCN. After the completion of the reaction 15 h, the crude was purified via automated flash chromatography using EtOAc in hexanes (0% to 100%) with product eluting at 1.0% on a 4 g silica column to afford it in 87% yield (43.1 mg, 0.10 mmol) as an oil. **^{1}H NMR** (800 MHz, $CDCl_3$) δ 8.13 (d, J = 7.7 Hz, 2H), 7.51 – 7.22 (m, 11H), 5.13 (s, 2H), 4.32 (t, J = 7.3 Hz, 2H), 2.35 (t, J = 7.5 Hz, 2H), 1.89 (p, J = 7.4 Hz, 2H), 1.63 (p, J = 7.3 Hz, 2H), 1.42 – 1.36 (m, 2H), 1.36 – 1.32 (m, 2H), 1.32 – 1.26 (m, J = 3.7, 2.9 Hz, 4H). **^{13}C NMR** (201 MHz, $CDCl_3$) δ 173.6, 140.4, 136.1, 128.5, 128.1, 125.5, 122.8, 120.3, 118.7,

108.6, 66.1, 43.1, 34.2, 29.2, 29.1, 29.0, 28.9, 27.3, 24.8 (missing C between 128). HRMS (ESI) m/z: [M+H] + calcd for $C_{28}H_{31}NO_2H$ 414.2428; found 414.2438.

19a: 5-(benzyloxy)-5-oxopentyl 2-acetoxybenzoate

19a

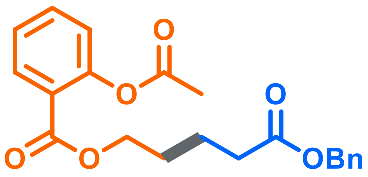

from Aspirin

Br, 79%

Following the general procedure C, 5-(benzyloxy)-5-oxopentyl 2-acetoxybenzoate was isolated using 2-bromoethyl 2-acetoxybenzoate **(19)** (0.12 mmol, 34 mg, 1 equiv), benzyl acrylate (0.24 mmol, 38.9 mg, 2.0 equiv, 36.5 µL), DIPEA (0.36 mmol, 46.5 mg, 62.7 µL, 3.0 equiv), DI water (12 mmol, 216 mg, 216 µL, 100 equiv), 2-isopropyl-3,5-lutidinium iodide (5.4 µmol, 1.5 mg, 0.05 equiv) and 0.5 mL of stock solution of [Ir(2′,4′-dF-5-CF_3-ppy)$_2$(4,4′-dtbbpy)]PF_6 in MeCN. After the completion of the reaction 15 h, the crude was purified via automated flash chromatography using EtOAc in hexanes (0% to 100%) with product eluting at 5.0% on a 4 g silica column to afford it in 79% yield (35.1 mg, 0.09 mmol) as an oil. ^{1}**H NMR** (400 MHz, $CDCl_3$) δ 7.73 (dd, J = 8.0, 1.8 Hz, 1H), 7.35 (ddd, J = 8.7, 7.2, 1.8 Hz, 2H), 7.26 (d, J = 1.6 Hz, 2H), 7.24 – 7.20 (m, 2H), 6.91 – 6.84 (m, 1H), 6.77 (ddd, J = 8.2, 7.3, 1.2 Hz, 1H), 5.04 (s, 3H), 4.25 (td, J = 5.0, 2.0 Hz, 3H), 2.36 (td, J = 5.8, 3.4 Hz, 2H), 2.26 (d, J = 5.5 Hz, 1H), 2.00 (s, 1H), 1.76 – 1.69 (m, 4H).^{13}**C NMR** (201 MHz, $CDCl_3$) δ 173.0, 170.1, 161.7, 135.9, 135.9, 135.7, 129.8, 128.6, 128.3, 128.2, 119.1, 117.6, 112.4, 66.3, 64.8, 33.7, 28.0, 21.5, 21.5. HRMS (ESI) m/z: [M+H] + calcd for $C_{21}H_{22}O_6H$ 371.1489; found 371.1492.

20a: benzyl 9-(2-(2-(trifluoromethyl)phenyl)acetoxy)nonanoate

20a

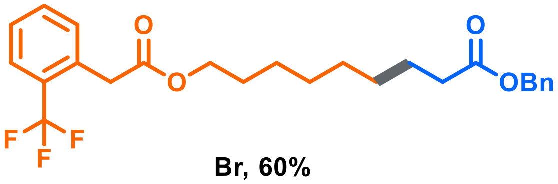

Br, 60%

Following the general procedure C, benzyl 9-(2-(2-(trifluoromethyl)phenyl)acetoxy)nonanoate was isolated using 2-bromoethyl 2-acetoxybenzoate (0.12 mmol, 43.9 mg, 1 equiv), benzyl acrylate (0.24 mmol, 38.9 mg, 2.0 equiv, 36.5 µL DIPEA (0.36 mmol, 46.5 mg, 62.7 µL, 3.0 equiv), DI water (12 mmol, 216 mg, 216 µL, 100 equiv), 2-isopropyl-3,5-lutidinium iodide (5.4 µmol, 1.5 mg, 0.05 equiv) and 0.5 mL of stock solution of [Ir(2′,4′-dF-5-CF_3-ppy)$_2$(4,4′-dtbbpy)]PF_6 in MeCN. After the completion of the reaction 15 h, the crude was purified via automated flash chromatography using EtOAc in hexanes (0% to 100%) with product eluting at 5.0% on a 4 g silica column to afford it in 60% yield (32.4 mg, 0.07 mmol) as an oil. ^{1}**H NMR** (800 MHz, $CDCl_3$) δ 7.65 (d, J = 7.8 Hz, 1H), 7.51 (t, J = 7.6 Hz, 1H), 7.40 – 7.31 (m, 7H), 5.12 (s, 2H), 4.09 (t, J = 6.7 Hz, 2H), 3.84 – 3.79 (m, 2H), 2.35 (t, J = 7.5 Hz, 2H), 1.61 (dt, J = 38.5, 7.0 Hz, 5H), 1.30 – 1.24 (m, 12H). ^{13}**C NMR** (201 MHz, $CDCl_3$) δ 173.7, 170.8, 136.1, 132.6, 132.5, 131.9, 129.0 (q, J = 29.5 Hz), 128.6, 128.2, 127.3, 126.1 (q, J = 5.4 Hz), 126.4 (q, J = 277 Hz), 66.1, 65.2, 38.3, 34.3, 29.1, 29.0, 28.9, 28.4, 25.7, 24.9. ^{19}**F NMR** (753 MHz, $CDCl_3$) δ -60.0. HRMS (ESI) m/z: [M+H] + calcd for $C_{25}H_{29}O_4F_3H$ 451.2091; found 451.2038.

21a: benzyl 5-(3-fluorophenyl)pentanoate

Following the general procedure C, benzyl 5-(3-fluorophenyl)pentanoate was isolated using 1-(2-chloroethyl)-4-fluorobenzene (0.12 mmol, 18.9 mg, 21.5 µL,1 equiv), benzyl acrylate (0.36 mmol, 58 mg,

21a

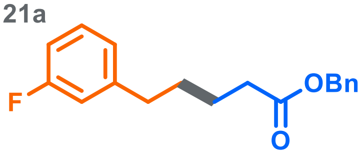

Cl, 92%

3.0 equiv, 54.7 μL), DIPEA (0.42 mmol, 54.3 mg, 73.2 μL, 3.5 equiv), DI water (1.2 mmol, 21.6 mg, 21.6 μL, 10 equiv), ethyl-3,5-lutidinium iodide (6.1 μmol, 1.6 mg, 0.05 equiv)and 0.5 mL of stock solution of [Ir(2′,4′-dF-5-CF_3-ppy)$_2$(4,4′-dtbbpy)]PF_6 in MeCN. After the completion of the reaction 15 h, the crude was purified via automated flash chromatography using EtOAc in hexanes (0% to 100%) with product eluting at 1.0% on a 4 g silica column to afford it in 92% yield (31 mg, 0.11 mmol) as an oil. **^{1}H NMR (400 MHz, $CDCl_3$)** δ 7.32 – 7.19 (m, 5H), 7.09 – 6.92 (m, 2H), 6.90 – 6.81 (m, 2H), 5.03 (s, 2H), 2.50 (t, *J* = 7.3 Hz, 2H), 2.30 (t, *J* = 7.1 Hz, 2H), 1.70 – 1.46 (m, 4H). **^{19}F NMR** (376 MHz, $CDCl_3$) δ -117.84 (ddd, *J* = 14.3, 9.0, 5.4 Hz). **^{13}C NMR** (101 MHz, $CDCl_3$) δ 173.38, 162.46, 160.05, 137.70, 137.67, 136.08, 129.72, 129.64, 128.59, 128.24, 115.14, 114.93, 66.18, 34.74, 34.12, 30.97, 24.48. **CI-MS:** observed [M]+ 285 [M+CH_3] 299.

22a: benzyl 5-phenoxypentanoate

22a

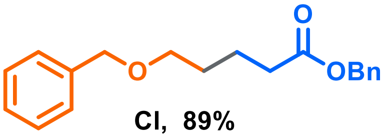

Cl, 89%

Following the general procedure C, benzyl 5-phenoxypentanoate was isolated using (2-chloroethoxy)methyl benzene (0.12 mmol, 20.4 mg, 23.2 μL,1 equiv), benzyl acrylate (0.36 mmol, 58 mg, 3.0 equiv, 54.7 μL), DIPEA (0.42 mmol, 54.3 mg, 73.2 μL, 3.5 equiv), DI water (1.2 mmol, 21.6 mg, 21.6 μL, 10 equiv), ethyl-3,5-lutidinium iodide (6.1 μmol, 1.6 mg, 0.05 equiv)and 0.5 mL of stock solution of [Ir(2′,4′-dF-5-CF_3-ppy)$_2$(4,4′-dtbbpy)]PF_6 in MeCN. After the completion of the reaction 15 h, the crude was purified via automated flash chromatography using EtOAc in hexanes (0% to 100%) with product eluting at 1.0% on a 4 g silica column to afford it in 92% yield (31.8 mg, 0.10 mmol) as an oil. **^{1}H NMR (400 MHz, $CDCl_3$)** δ 7.38 – 7.21 (m, 10H), 5.08 (s, 4H), 2.78 – 2.69 (m, 4H), 2.53 – 2.45 (m, 4H). **^{13}C NMR (101 MHz, $CDCl_3$)** δ 172.06, 135.68, 128.64, 128.40, 128.35, 89.23, 66.69, 44.26, 37.71, 30.94. **CI-MS:** observed [MH]+ 299

H1.2: Secondary Halides

23a: benzyl 4-methylpentanoate

23a

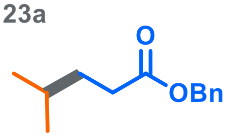

Br, 85%

Following the general procedure D, benzyl 4-methylpentanoate was isolated using 2-bromopropane (0.12 mmol, 14.7 mg, 11.2 μL, 1 equiv), benzyl acrylate (0.24 mmol, 38.9 mg, 2.0 equiv, 36.5 μL), DIPEA (0.36 mmol, 46.5 mg, 62.7 μL, 3.0 equiv), DI water (12 mmol, 216 mg, 216 μL, 100 equiv), 2-isopropyl-3,5-lutidinium iodide (5.4 μmol, 1.5 mg, 0.05 equiv) and 0.5 mL of stock solution of [Ir(2′,4′-dF-5-CF_3-ppy)$_2$(4,4′-dtbbpy)]PF_6 in MeCN. After the completion of the reaction 15 h, the crude was purified via automated flash chromatography using EtOAc in hexanes (0% to 100%) with product eluting at 1.0% on a 4 g silica column to afford it in 85% yield (21.0 mg, 0.10 mmol) as an oil. **^{1}H NMR** (800 MHz, $CDCl_3$) δ 7.40 – 7.33 (m, 5H), 5.13 (s, 2H), 2.41 – 2.36 (m, 2H), 1.59 – 1.56 (m, 3H), 0.92 (d, *J* = 6.3 Hz, 6H). **^{13}C NMR** (201 MHz, $CDCl_3$) δ 173.9, 136.1, 128.5, 128.1, 66.1, 33.7, 32.4, 27.6, 22.2 (missing C signal at 128). NMR chemical shifts match with the literature value.[12]

24a: 5-benzyl 1-methyl 2-methylpentanedioate

24a

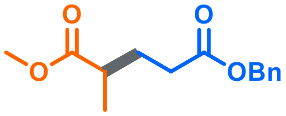

Br, 85%

Following the general procedure D, 5-benzyl 1-methyl 2-methylpentanedioate was isolated using methyl 2-bromopropionate (0.12 mmol, 20 mg, 13.38 µL, 1 equiv), benzyl acrylate (0.24 mmol, 38.9 mg, 2.0 equiv, 36.5 µL), DIPEA (0.36 mmol, 46.5 mg, 62.7 µL, 3.0 equiv), DI water (12 mmol, 216 mg, 216 µL, 100 equiv), 2-isopropyl-3,5-lutidinium iodide (5.4 µmol, 1.5 mg, 0.05 equiv) and 0.5 mL of stock solution of [Ir(2′,4′-dF-5-CF_3-ppy)$_2$(4,4′-dtbbpy)]PF_6 in MeCN. After the completion of the reaction 15 h, the crude was purified via automated flash chromatography using EtOAc in hexanes (0% to 100%) with product eluting at 1.0% on a 4 g silica column to afford it in 85% yield (25.5 mg, 0.10 mmol) as an oil. **^{1}H NMR** (800 MHz, $CDCl_3$) δ 7.43 – 7.37 (m, 3H), 7.37 – 7.33 (m, 2H), 5.14 (s, 2H), 3.68 (s, 3H), 2.53 (dqd, *J* = 8.3, 7.0, 5.8 Hz, 1H), 2.46 – 2.37 (m, 2H), 2.01 (dtd, *J* = 13.9, 8.5, 6.4 Hz, 1H), 1.83 (dddd, *J* = 13.8, 8.9, 6.8, 6.0 Hz, 1H), 1.19 (d, *J* = 7.0 Hz, 3H). **^{13}C NMR** (201 MHz, $CDCl_3$) δ 176.4, 172.8, 135.9, 128.5, 128.5, 128.2, 128.2, 66.1, 51.6, 38.6, 31.9, 28.5, 17.0. NMR chemical shifts match with the literature value.[12]

24a: 5-benzyl 1-methyl 2-methylpentanedioate

24a

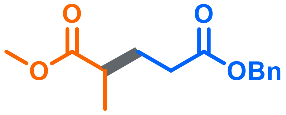

Cl, 71%

Following the general procedure D, 5-benzyl 1-methyl 2-methylpentanedioate was isolated using methyl 2-chloropropionate (0.12 mmol, 14.7 mg, 12.9 µL, 1 equiv), benzyl acrylate (0.24 mmol, 38.9 mg, 2.0 equiv, 36.5 µL), DIPEA (0.3 mmol, 38.7 mg, 52.2 µL, 2.5 equiv), DI water (1.2 mmol, 21.6 mg, 21.6 µL, 10 equiv), ethyl-3,5-lutidinium iodide (6.1 µmol, 1.6 mg, 0.05 equiv) and 0.5 mL of stock solution of [Ir(2′,4′-dF-5-CF_3-ppy)$_2$(4,4′-dtbbpy)]PF_6 in MeCN. After the completion of the reaction 15 h, the crude was purified via automated flash chromatography using EtOAc in hexanes (0% to 100%) with product eluting at 1.0% on a 4 g silica column to afford it in 71% yield (21.3 mg, 0.08 mmol) as an oil. **^{1}H NMR** (800 MHz, $CDCl_3$) δ 7.43 – 7.37 (m, 3H), 7.37 – 7.33 (m, 2H), 5.14 (s, 2H), 3.68 (s, 3H), 2.53 (dqd, *J* = 8.3, 7.0, 5.8 Hz, 1H), 2.46 – 2.37 (m, 2H), 2.01 (dtd, *J* = 13.9, 8.5, 6.4 Hz, 1H), 1.83 (dddd, *J* = 13.8, 8.9, 6.8, 6.0 Hz, 1H), 1.19 (d, *J* = 7.0 Hz, 3H). **^{13}C NMR** (201 MHz, $CDCl_3$) δ 176.4, 172.8, 135.9, 128.5, 128.5, 128.2, 128.2, 66.3, 51.6, 38.6, 31.9, 28.5, 17.0. NMR chemical shifts match with the literature value.[12]

25a: benzyl 3-cyclopentylpropanoate

25a

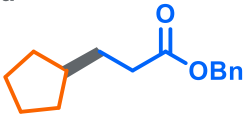

Br, 74%

Following the general procedure D, benzyl 3-cyclopentylpropanoate was isolated using bromocyclopentane (0.12 mmol, 17.8 mg, 12.8 µL, 1 equiv), benzyl acrylate (0.24 mmol, 38.9 mg, 2.0 equiv, 36.5 µL), DIPEA (0.36 mmol, 46.5 mg, 62.7 µL, 3.0 equiv), DI water (12 mmol, 216 mg, 216 µL, 100 equiv), 2-isopropyl-3,5-lutidinium iodide (5.4 µmol, 1.5 mg, 0.05 equiv) and 0.5 mL of stock solution of [Ir(2′,4′-dF-5-CF_3-ppy)$_2$(4,4′-dtbbpy)]PF_6 in MeCN. After the completion of the reaction 15 h, the crude was purified via automated flash chromatography using EtOAc in hexanes (0% to 100%) with product eluting at 1.0% on a 4 g silica column to afford it in 74% yield (20.6 mg, 0.08 mmol) as an oil. **^{1}H NMR** (400 MHz, $CDCl_3$) δ 7.41 – 7.30 (m, 5H), 5.11 (s, 2H), 2.41 – 2.33 (m, 2H), 1.82 – 1.43 (m, 9H), 1.16 – 1.03 (m, 2H). **^{13}C NMR** (101 MHz, $CDCl_3$) δ 174.0, 136.3, 128.7, 128.3, 128.3, 66.2, 39.8, 33.8, 32.5, 31.3, 29.9, 25.3. NMR chemical shifts match with the literature value.[8]

26a: benzyl 3-cyclohexylpropanoate

26a

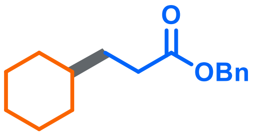

Cl, 40 %

Following the general procedure D, benzyl 3-cyclohexylpropanoate was isolated using chlorocyclohexane (0.12 mmol, 14.2 mg, 14.2 µL, 1 equiv), benzyl acrylate (0.24 mmol, 38.9 mg, 2.0 equiv, 36.5 µL), DIPEA (0.3 mmol, 38.7 mg, 52.2 µL, 2.5 equiv), DI water (1.2 mmol, 21.6 mg, 21.6 µL, 10 equiv), ethyl-3,5-lutidinium iodide (6.1 µmol, 1.6 mg, 0.05 equiv) and 0.5 mL of stock solution of [Ir(2′,4′-dF-5-CF_3-ppy)$_2$(4,4′-dtbbpy)]PF_6 in MeCN. After the completion of the reaction 15 h, the crude was purified via automated flash chromatography using EtOAc in hexanes (0% to 100%) with product eluting at 1.0% on a 4 g silica column to afford it in 40% yield (11.8 mg, 0.04 mmol) as an oil. **^{1}H NMR** (400 MHz, $CDCl_3$) δ 7.41 – 7.31 (m, 5H), 5.12 (s, 2H), 2.42 – 2.31 (m, 2H), 1.73 – 1.66 (m, 3H), 1.59 – 1.51 (m, 2H), 1.43 (d, *J* = 5.7 Hz, 2H), 1.31 – 1.08 (m, 4H), 0.98 – 0.78 (m, 2H). **^{13}C NMR** (201 MHz, $CDCl_3$) δ 174.1, 136.2, 128.6, 128.3, 128.2, 66.2, 37.3, 33.1, 32.4, 32.1, 26.6, 26.3. NMR chemical shifts match with the literature value.[8]

27a: benzyl 3-(tetrahydro-2H-pyran-4-yl) propanoate

27a

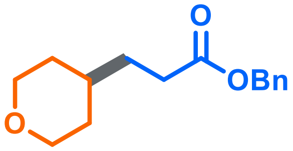

Br, 97%

Following the general procedure D, benzyl 3-(tetrahydro-2H-pyran-4-yl)propanoate was isolated using 4-bromotetrahydro-2H-pyran (0.12 mmol, 19.8 mg, 13.4 µL, 1 equiv), benzyl acrylate (0.24 mmol, 38.9 mg, 2.0 equiv, 36.5 µL), DIPEA (0.36 mmol, 46.5 mg, 62.7 µL, 3.0 equiv), DI water (12 mmol, 216 mg, 216 µL, 100 equiv), 2-isopropyl-3,5-lutidinium (5.4 µmol, 1.5 mg, 0.05 equiv) and 0.5 mL of stock solution of [Ir(2′,4′-dF-5-CF_3-ppy)$_2$(4,4′-dtbbpy)]PF_6 in MeCN. After the completion of the reaction 15 h, the crude was purified via automated flash chromatography using EtOAc in hexanes (0% to 100%) with product eluting at 1.0% on a 4 g silica column to afford it in 97% yield (28.8 mg, 0.11 mmol) as an oil. **^{1}H NMR** (400 MHz, $CDCl_3$) δ 7.39 – 7.30 (m, 5H), 5.12 (s, 2H), 3.93 (ddt, *J* = 11.5, 4.5, 1.2 Hz, 2H), 3.33 (td, *J* = 11.8, 2.1 Hz, 2H), 2.39 (t, *J* = 7.7 Hz, 2H), 1.70 – 1.53 (m, 4H), 1.47 (ddq, *J* = 14.3, 7.0, 3.7 Hz, 1H), 1.34 – 1.18 (m, 2H). **^{13}C NMR** (101 MHz, $CDCl_3$) δ 173.7, 136.1, 128.7, 128.4, 128.3,76.8, 68.0, 66.4, 34.5, 32.8, 31.9, 31.5. HRMS (ESI) m/z: [M+H] + calcd for $C_{15}H_{20}O_3H$ 249.1485; found 249.2326.

28a: tert-butyl 4-(3-(benzyloxy)-3-oxopropyl) piperidine-1-carboxylate

28a

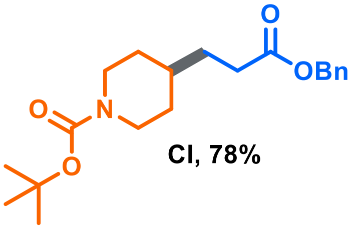

Cl, 78%

Following the general procedure D, tert-butyl 4-(3-(benzyloxy)-3-oxopropyl)piperidine-1-carboxylate was isolated using tert-butyl 4-chloropiperidine-1-carboxylate (0.12 mmol, 26.3 mg, 1 equiv), benzyl acrylate (0.24 mmol, 38.9 mg, 2.0 equiv, 36.5 µL), DIPEA (0.3 mmol, 38.7 mg, 52.2 µL, 2.5 equiv), DI water (1.2 mmol, 21.6 mg, 21.6 µL, 10 equiv), ethyl-3,5-lutidinium iodide (6.1 µmol, 1.6 mg, 0.05 equiv) and 0.5 mL of stock solution of [Ir(2′,4′-dF-5-CF_3-ppy)$_2$(4,4′-dtbbpy)]PF_6 in MeCN. After the completion of the reaction 15 h, the crude was purified via automated flash chromatography using EtOAc in hexanes (0% to 100%) with product eluting at 1.0% on a 4 g silica column to afford it in 78% yield (32.4 mg, 0.09 mmol) as an oil. **^{1}H NMR** (400 MHz, $CDCl_3$) δ 7.34 – 7.21 (m, 5H), 5.05 (s, 2H), 4.13 (tt, *J* = 7.6, 3.7 Hz, 1H), 3.64 (ddd, *J* = 13.6, 7.0, 3.7 Hz, 1H), 3.32 – 3.17 (m, 2H), 2.32 (q, *J* = 7.5 Hz, 2H), 1.95 (ddq, *J* = 15.1, 8.0, 4.0 Hz, 2H), 1.79 – 1.64 (m, 1H), 1.39 (s, 9H), 1.09 (t, *J* = 7.6 Hz, 2H), 0.99 – 0.77 (m, 2H). **^{13}C NMR** (201 MHz, $CDCl_3$) δ 174.4, 136.2, 128.5, 128.2, 128.1, 65.9, 48.2, 43.9, 32.1, 30.9, 23.9, 22.1, 21.0. NMR chemical shifts match with the literature value.[13]

29f: 3-((2S, R)-4,7,7-trimethyl-3-oxobicyclo [2.2.1] heptan-2-yl)propanenitrile

29f

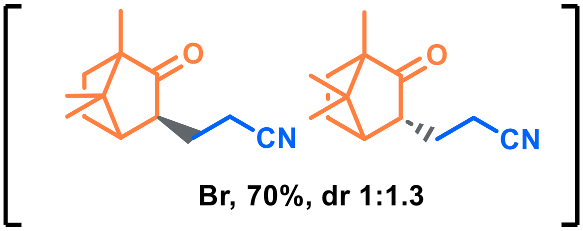

Br, 70%, dr 1:1.3

Following the general procedure D, 3-((2S,R)-4,7,7-trimethyl-3-oxobicyclo[2.2.1]heptan-2-yl)propanenitrile was isolated using (3S)-3-bromo-1,7,7-trimethylbicyclo[2.2.1]heptan-2-one (0.12 mmol, 27.6 mg, 1 equiv), acrylonitrile (0.24 mmol, 12.7 mg, 2.0 equiv, 15.7 µL), DIPEA (0.36 mmol, 46.5 mg, 62.7 µL, 3.0 equiv), DI water (12 mmol, 216 mg, 216 µL, 100 equiv), 2-isopropyl-3,5-lutidinium iodide (5.4 µmol, 1.5 mg, 0.05 equiv) and 0.5 mL of stock solution of [Ir(2′,4′-dF-5-CF_3-ppy)$_2$(4,4′-dtbbpy)]PF_6 in MeCN. After the completion of the reaction 15 h, the crude was purified via automated flash chromatography using EtOAc in hexanes (0% to 100%) with product eluting at 1.0% on a 4 g silica column to afford it in 70% yield (17.2 mg, 0.08 mmol) as an oil. **^{1}H NMR** (800 MHz, $CDCl_3$) δ 2.59 – 2.51 (m, 5H), 2.14 – 2.09 (m, 2H), 2.07 – 1.98 (m, 4H), 1.90 (dd, J = 7.9, 6.4 Hz, 1H), 1.77 – 1.61 (m, 5H), 1.55 – 1.39 (m, 4H), 1.29 (ddd, J = 14.2, 9.3, 5.1 Hz, 1H), 1.01 (s, 3H), 0.95 (s, 4H), 0.91 – 0.89 (m, 10H), 0.84 (s, 4H). **^{13}C NMR** (201 MHz, $CDCl_3$) δ 220.2, 220.1, 119.6, 119.5, 58.9, 57.8, 53.3, 48.2, 47.7, 46.9, 46.5, 46.1, 31.2, 29.3, 29.2, 27.7, 23.8, 21.8, 20.5, 20.4, 19.7, 19.3, 16.2, 9.6, 9.4. HRMS (ESI) m/z: [M+H] + calcd for $C_{13}H_{19}ONH$ 206.1539; found 206.1539.

H1.3: Tertiary Halides

30a: benzyl 4,4-dimethylhexanoate

30a

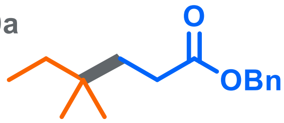

Cl, 43 %

Following the general procedure D, benzyl 4,4-dimethylhexanoate was isolated using 1-chloro-2,2-dimethylbutane (0.12 mmol, 48.6 mg, 1 equiv), benzyl acrylate (0.3 mmol, 48.6 mg, 2.5 equiv, 45.6 µL), DIPEA (0.48 mmol, 62.0 mg, 83.6 µL, 4.0 equiv), DI water (1.2 mmol, 21.6 mg, 21.6 µL, 10 equiv), ethyl-3,5-lutidinium iodide (6.1 µmol, 1.6 mg, 0.05 equiv) and 0.5 mL of stock solution of [Ir(2′,4′-dF-5-CF_3-ppy)$_2$(4,4′-dtbbpy)]PF_6 in MeCN. After the completion of the reaction 15 h, the crude was purified via automated flash chromatography using EtOAc in hexanes (0% to 100%) with product eluting at 1.0% on a 4 g silica column to afford it in 43% yield (12.1 mg, 0.05 mmol) as an oil. **^{1}H NMR** (800 MHz, $CDCl_3$) δ 7.40 – 7.29 (m, 5H), 5.11 (s, 2H), 2.33 – 2.27 (m, 2H), 1.63 – 1.54 (m, 3H), 1.22 (q, J = 7.5 Hz, 2H), 0.83 (s, 5H), 0.81 (t, J = 7.5 Hz, 3H). **^{13}C NMR** (201 MHz, $CDCl_3$) δ 174.5, 136.3, 128.7, 128.3, 128.3, 66.3, 36.1, 34.0, 29.9, 26.4, 14.3, 8.5. HRMS (ESI) m/z: [M+H] + calcd for $C_{15}H_{22}O_2H$ 235.1693; found 235.1612.

31a: benzyl 4,4-dimethylpentanoate

31a

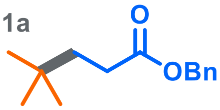

Br, 53 %

Following the general procedure D, benzyl 4,4-dimethylpentanoate was isolated using 2-bromo-2-methylpropane (0.12 mmol, 16.4 mg, 13.4 µL, 1 equiv), benzyl acrylate (0.3 mmol, 48.6 mg, 2.5 equiv, 45.6 µL), DIPEA (0.48 mmol, 62.0 mg, 83.6 µL, 4.0 equiv), DI water (1.2 mmol, 21.6 mg, 21.6 µL, 10 equiv), 2-isopropyl-3,5-lutidinium iodide (5.4 µmol, 1.5 mg, 0.05 equiv) and 0.5 mL of stock solution of [Ir(2′,4′-dF-5-CF_3-ppy)$_2$(4,4′-dtbbpy)]PF_6 in MeCN. After the completion of the reaction 15 h, the crude was purified via automated flash chromatography using EtOAc in hexanes (0% to 100%) with product eluting at 1.0% on a 4 g silica column to afford it in 53% yield (14.0 mg, 0.06 mmol) as an oil. **^{1}H NMR**

(400 MHz, $CDCl_3$) δ 7.39 – 7.31 (m, 5H), 5.11 (s, 2H), 2.39 – 2.30 (m, 2H), 1.63 – 1.54 (m, 2H), 0.89 (s, 9H). **^{13}C NMR** (201 MHz, $CDCl_3$) δ 174.4, 136.2, 128.7, 128.4, 128.3, 66.3, 38.7, 30.3, 30.2, 29.2. NMR chemical shifts match with the literature value.[12]

32a: 5-benzyl 1-ethyl 2,2-dimethylpentanedioate

32a

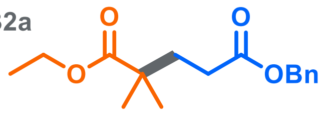

Br, 67%

Following the general procedure D, benzyl 4,4-dimethylpentanoate was isolated using ethyl 2-bromo-2-methylpropanoate (0.12 mmol, 23.4 mg, 17.6 µL, 1 equiv), benzyl acrylate (0.3 mmol, 48.6 mg, 2.5 equiv, 45.6 µL), DIPEA (0.48 mmol, 62.0 mg, 83.6 µL, 4.0 equiv), DI water (1.2 mmol, 21.6 mg, 21.6 µL, 10 equiv), 2-isopropyl-3,5-lutidinium iodide (5.4 µmol, 1.5 mg, 0.05 equiv) and 0.5 mL of stock solution of [Ir(2′,4′-dF-5-CF_3-ppy)$_2$(4,4′-dtbbpy)]PF_6 in MeCN. After the completion of the reaction 15 h, the crude was purified via automated flash chromatography using EtOAc in hexanes (0% to 100%) with product eluting at 1.0% on a 4 g silica column to afford it in 67% yield (22 mg, 0.08 mmol) as an oil. **^{1}H NMR** (400 MHz, $CDCl_3$) δ 7.41 – 7.30 (m, 5H), 5.11 (s, 2H), 4.11 (q, J = 7.1 Hz, 2H), 2.40 – 2.29 (m, 2H), 1.98 – 1.85 (m, 2H), 1.24 (d, J = 7.2 Hz, 3H), 1.18 (s, 6H). **^{13}C NMR** (101 MHz, $CDCl_3$) δ 177.3, 173.4, 136.0, 128.6, 128.3, 66.4, 60.6, 41.7, 35.1, 30.3, 25.1, 14.3 (missing C signal around 128). HRMS (ESI) m/z: [M+H] + calcd for $C_{16}H_{22}O_4H$ 279.1591; found 279.1593.

33f: 3-((3r,5r,7r)-adamantan-1-yl)propanenitrile

33f

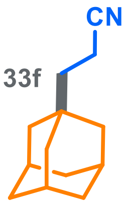

Br, 0% I, 61%

Following the general procedure D, 3-((3r,5r,7r)-adamantan-1-yl)propanenitrile was isolated using 1-iodoadamantane (0.12 mmol, 31.45 mg, 1 equiv), benzyl acrylate (0.3 mmol, 48.6 mg, 2.5 equiv, 45.6 µL), DIPEA (0.48 mmol, 62.0 mg, 83.6 µL, 4.0 equiv), DI water (1.2 mmol, 21.6 mg, 21.6 µL, 10 equiv), 2-isopropyl-3,5-lutidinium iodide (5.4 µmol, 1.5 mg, 0.05 equiv) and 0.5 mL of stock solution of [Ir(2′,4′-dF-5-CF_3-ppy)$_2$(4,4′-dtbbpy)]PF_6 in MeCN. After the completion of the reaction 15 h, the crude was purified via automated flash chromatography using EtOAc in hexanes (0% to 100%) with product eluting at 2.0% on a 4 g silica column to afford it in 61% yield (13.9 mg, 0.07 mmol) as an oil. **^{1}H NMR** (800 MHz, $CDCl_3$) δ 2.32 – 2.27 (m, 2H), 2.03 – 1.99 (m, 3H), 1.75 (s, 3H), 1.66 – 1.63 (m, 3H), 1.53 – 1.48 (m, 8H). **^{13}C NMR** (201 MHz, $CDCl_3$) δ 120.9, 41.6, 39.4, 36.8, 29.7, 28.3, 10.9. NMR chemical shifts match with the literature value.[12]

H1.4: Methyl Radical Substrate

34a: benzyl 4-chlorobutanoate

34a

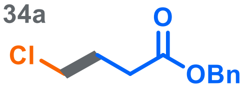

Cl, 62%
(From DCM)

Following the general procedure D, benzyl 4-chlorobutanoate was isolated using dichloromethane (0.12 mmol, 10.2 mg, 7.7 µL, 1 equiv), benzyl acrylate (0.24 mmol, 38.9 mg, 2.0 equiv, 36.5 µL), DIPEA (0.36 mmol, 46.5 mg, 62.7 µL, 3.0 equiv), DI water (12 mmol, 216 mg, 216 µL, 100 equiv), 2-isopropyl-3,5-lutidinium iodide (6.1 µmol, 1.6 mg, 0.05 equiv) and 0.5 mL of stock solution of [Ir(2′,4′-dF-5-CF_3-ppy)$_2$(4,4′-dtbbpy)]PF_6 in MeCN. The reaction was run at 60 °C. After the completion of the reaction 15 h, the crude was purified via automated flash chromatography using EtOAc in hexanes (0% to 100%) with product eluting at 1.0% on a 4 g silica column to afford it in 62% yield (15.7 mg, 0.07 mmol) as an oil. **^{1}H NMR** (400 MHz, $CDCl_3$) δ 7.42 – 7.29 (m, 5H), 5.14 (s, 2H), 3.60 (t, J = 6.3 Hz, 2H), 2.56 (t, J =

7.2 Hz, 2H), 2.12 (p, J = 6.8 Hz, 2H). **^{13}C NMR** (201 MHz, $CDCl_3$) δ 172.6, 135.9, 128.7, 128.4, 128.4, 66.5, 44.2, 31.4, 27.8. NMR chemical shifts match with the literature value.[14]

35a: benzyl 4,4-dichlorobutanoate

35a

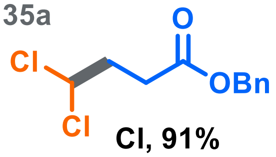

Cl, 91% (From $CHCl_3$)

Following the general procedure D, benzyl 4-chlorobutanoate was isolated using chloroform (0.12 mmol, 14.3 mg, 9.6 µL, 1 equiv), benzyl acrylate (0.24 mmol, 38.9 mg, 2.0 equiv, 36.5 µL), DIPEA (0.36 mmol, 46.5 mg, 62.7 µL, 3.0 equiv), DI water (12 mmol, 216 mg, 216 µL, 100 equiv), 2-isopropyl-3,5-lutidinium iodide (6.1 µmol, 1.6 mg, 0.05 equiv) and 0.5 mL of stock solution of [Ir(2′,4′-dF-5-CF_3-ppy)$_2$(4,4′-dtbbpy)]PF_6 in MeCN. The reaction was run at 60 °C. After the completion of the reaction 15 h, the crude was purified via automated flash chromatography using EtOAc in hexanes (0% to 100%) with product eluting at 5.0% on a 4 g silica column to afford it in 91% yield (27 mg, 0.11 mmol) as an oil. **^{1}H NMR** (800 MHz, $CDCl_3$) δ 7.37 (dtd, J = 17.2, 7.8, 6.4 Hz, 5H), 5.91 (t, J = 5.9 Hz, 1H), 5.15 (s, 2H), 2.68 (t, J = 7.2 Hz, 2H), 2.53 (td, J = 7.3, 5.8 Hz, 2H).**^{13}C NMR** (201 MHz, $CDCl_3$) δ 171.8, 135.7, 128.7, 128.7. **EI-MS:** observed [M]+ 246

H1.5: Pseudohalide Substrate

36a:benzyl pentanoate

36a

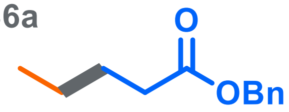

OTs, 82%

Following the general procedure D, benzyl pentanoate was isolated using ethyl 4-methylbenzenesulfonate (0.12 mmol, 24 mg, 1 equiv), benzyl acrylate (0.24 mmol, 38.9 mg, 2.0 equiv, 36.5 µL), DIPEA (0.36 mmol, 46.5 mg, 62.7 µL, 3.0 equiv), DI water (12 mmol, 216 mg, 216 µL, 100 equiv), 2-isopropyl-3,5-lutidinium iodide (6.1 µmol, 1.6 mg, 0.05 equiv) and 0.5 mL of stock solution of [Ir(2′,4′-dF-5-CF_3-ppy)$_2$(4,4′-dtbbpy)]PF_6 in MeCN. After the completion of the reaction 15 h, the crude was purified via automated flash chromatography using EtOAc in hexanes (0% to 100%) with product eluting at 2.5% on a 4 g silica column to afford it in 82% yield (19 mg, 0.09 mmol) as an oil. **^{1}H NMR** (800 MHz, $CDCl_3$) δ 7.40 – 7.31 (m, 5H), 5.13 (s, 2H), 2.37 (t, J = 7.6 Hz, 2H), 1.65 (p, J = 7.6 Hz, 2H), 1.39 – 1.34 (m, 2H), 0.93 (t, J = 7.4 Hz, 3H). **^{13}C NMR** ^{13}C NMR (201 MHz, $CDCl_3$) δ 173.8, 136.3, 128.7, 128.3, 66.2, 34.2, 27.2, 22.4, 13.8. NMR chemical shifts match with the literature value.[15]

36a:benzyl pentanoate

36a

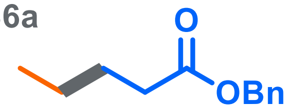

CF_3CO_2, 21%

Following the general procedure D, benzyl pentanoate was isolated using ethyl 2,2,2-trifluoroacetate (0.12 mmol, 17 mg, 1 equiv, 14.5 µL), benzyl acrylate (0.24 mmol, 38.9 mg, 2.0 equiv, 36.5 µL), DIPEA (0.36 mmol, 46.5 mg, 62.7 µL, 3.0 equiv), DI water (12 mmol, 216 mg, 216 µL, 100 equiv), 2-isopropyl-3,5-lutidinium iodide (6.1 µmol, 1.6 mg, 0.05 equiv) and 0.5 mL of stock solution of [Ir(2′,4′-dF-5-CF_3-ppy)$_2$(4,4′-dtbbpy)]PF_6 in MeCN. After the completion of the reaction at 15h, work up was done. NMR yield of 21% was obtained on the crude using 1,3-benzodioxole as internal standard.

H2: Alkene Substrate Characterization

17b: ethyl 5-phenylpentanoate

17b

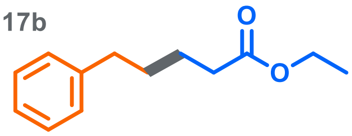

Cl, 82%

Following the general procedure C, ethyl 5-phenylpentanoate was isolated using (2-chloroethyl)benzene (0.12 mmol, 16 mg, 15.7 µL, 1 equiv), ethyl acrylate (0.36 mmol, 36 mg, 3.0 equiv, 39.2 µL), DIPEA (0.42 mmol, 54.3 mg, 73.2 µL, 3.5 equiv), DI water (1.2 mmol, 21.6 mg, 21.6 µL, 10 equiv), ethyl-3,5-lutidinium iodide (6.1 µmol, 1.6 mg, 0.05 equiv) and 0.5 mL of stock solution of [Ir(2′,4′-dF-5-CF_3-ppy)$_2$(4,4′-dtbbpy)]PF_6 in MeCN. After the completion of the reaction 15 h, the crude was purified via automated flash chromatography using EtOAc in hexanes (0% to 100%) with product eluting at 1.0% on a 4 g silica column to afford it in 82% yield (20 mg, 0.09 mmol) as an oil. **^{1}H NMR** (800 MHz, $CDCl_3$) δ 7.30 (dd, J = 8.7, 6.7 Hz, 2H), 7.22 – 7.15 (m, 3H), 4.14 (q, J = 7.1 Hz, 2H), 2.65 (t, J = 7.1 Hz, 2H), 2.34 (t, J = 7.1 Hz, 2H), 1.73 – 1.65 (m, 4H), 1.27 (t, J = 7.1 Hz, 3H). **^{13}C NMR** (201 MHz, $CDCl_3$) δ 173.7, 142.2, 128.4, 128.3, 125.8, 60.2, 35.6, 34.2, 30.9, 24.6, 14.3. NMR chemical shifts match with the literature value.[16]

17c: 6-phenylhexan-2-one

17c

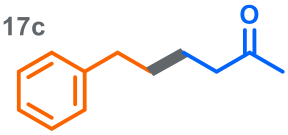

Cl, 38%

Following the general procedure C, 6-phenylhexan-2-one was isolated using (2-chloroethyl)benzene (0.12 mmol, 16 mg, 15.7 µL, 1 equiv), methyl vinyl ketone (0.36 mmol, 25 mg, 3.0 equiv, 29.6 µL), DIPEA (0.42 mmol, 54.3 mg, 73.2 µL, 3.5 equiv), DI water (1.2 mmol, 21.6 mg, 21.6 µL, 10 equiv), ethyl-3,5-lutidinium iodide (6.1 µmol, 1.6 mg, 0.05 equiv) and 0.5 mL of stock solution of [Ir(2′,4′-dF-5-CF_3-ppy)$_2$(4,4′-dtbbpy)]PF_6 in MeCN. After the completion of the reaction 15 h, the crude was purified via automated flash chromatography using EtOAc in hexanes (0% to 100%) with product eluting at 1.0% on a 4 g silica column to afford it in 38% yield (8 mg, 0.04 mmol) as an oil. **^{1}H NMR** (400 MHz, $CDCl_3$) δ 7.26 – 7.21 (m, 2H), 7.17 – 7.06 (m, 3H), 4.21 (t, J = 7.1 Hz, 2H), 2.87 (t, J = 7.1 Hz, 2H), 2.08 – 2.00 (m, 2H), 1.97 (s, 3H), 1.19 (d, J = 4.1 Hz, 1H), 0.95 – 0.89 (m, 1H). **^{13}C NMR** (201 MHz, CDCl3) δ 171.1, 128.9, 128.5, 128.3, 126.6, 91.2, 64.9, 60.4, 35.1, 21.0, 14.2. NMR chemical shifts match with the literature value.[17]

17d: ethyl 2-methyl-5-phenylpentanoate

17d

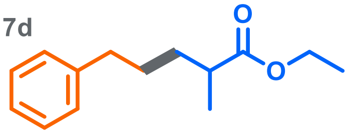

Cl, 87%

Following the general procedure C, ethyl 2-methyl-5-phenylpentanoate was isolated using (2-chloroethyl)benzene (0.12 mmol, 16 mg, 15.7 µL, 1 equiv), ethyl methacrylate (0.36 mmol, 41 mg, 3.0 equiv, 44.8 µL), DIPEA (0.42 mmol, 54.3 mg, 73.2 µL, 3.5 equiv), DI water (1.2 mmol, 21.6 mg, 21.6 µL, 10 equiv), ethyl-3,5-lutidinium iodide (6.1µmol, 1.6 mg, 0.05 equiv) and 0.5 mL of stock solution of [Ir(2′,4′-dF-5-CF_3-ppy)$_2$(4,4′-dtbbpy)]PF_6 in MeCN. After the completion of the reaction 15 h, the crude was purified via automated flash chromatography using EtOAc in hexanes (0% to 100%) with product eluting at 1.0% on a 4 g silica column to afford it in 87% yield (22 mg, 0.10 mmol) as an oil. **^{1}H NMR** (800 MHz, $CDCl_3$) δ 7.30 (dd, J = 8.3, 7.0 Hz, 2H), 7.22 – 7.17 (m, 3H), 4.14 (q, J = 7.1 Hz, 2H), 2.63 (p, J = 7.0 Hz, 2H), 2.46 (h, J = 7.0 Hz, 1H), 1.73 (dq, J = 13.1, 7.8 Hz, 1H), 1.65 (p, J = 7.7 Hz, 2H), 1.48 (ddt, J = 13.1, 8.6, 6.4 Hz, 1H), 1.27 (t, J = 7.1 Hz, 3H), 1.16 (d, J = 7.0 Hz, 3H). **^{13}C NMR** (201 MHz, $CDCl_3$) δ 176.7, 142.2,

128.3, 128.3, 125.7, 60.1, 39.4, 35.7, 33.4, 29.0, 17.1, 14.2. NMR chemical shifts match with the literature value.[18]

17e: tert-butyl 2-methyl-5-phenylpentanoate

17e

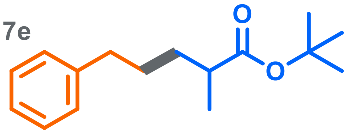

Cl, 68%

Following the general procedure C, tert-butyl 2-methyl-5-phenylpentanoate was isolated using (2-chloroethyl)benzene (0.12 mmol, 16 mg, 15.7 µL, 1 equiv), tert-butyl methacrylate (0.36 mmol, 51 mg, 3.0 equiv, 58 µL), DIPEA (0.42 mmol, 54.3 mg, 73.2 µL, 3.5 equiv), DI water (1.2 mmol, 21.6 mg, 21.6 µL, 10 equiv), ethyl-3,5-lutidinium iodide (6.1 µmol, 1.6 mg, 0.05 equiv) and 0.5 mL of stock solution of [Ir(2′,4′-dF-5-CF_3-ppy)$_2$(4,4′-dtbbpy)]PF_6 in MeCN. After the completion of the reaction 15 h, the crude was purified via automated flash chromatography using EtOAc in hexanes (0% to 100%) with product eluting at 1.0% on a 4 g silica column to afford it in 68% yield (20 mg, 0.08 mmol) as an oil. **^{1}H NMR** (800 MHz, $CDCl_3$) δ 7.31 – 7.27 (m, 2H), 7.22 – 7.14 (m, 3H), 2.64 (t, *J* = 7.5 Hz, 1H), 2.60 (q, *J* = 7.4 Hz, 1H), 2.39 – 2.33 (m, 1H), 1.73 – 1.62 (m, 2H), 1.45 (d, *J* = 13.8 Hz, 11H), 1.13 (dd, *J* = 20.9, 7.0 Hz, 3H). **^{13}C NMR** (201 MHz, $CDCl_3$) δ 176.2, 142.4, 128.4, 128.3, 125.7, 79.8, 60.4, 40.3, 35.8, 33.5, 28.1, 17.2, 14.2. NMR chemical shifts match with the literature value.[19]

37f: 6-phenylhexanenitrile

37f

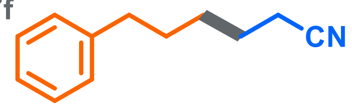

Cl, 83%

Following the general procedure C, 6-phenylhexanenitrile was isolated using (3-chloropropyl)benzene (0.12 mmol, 18 mg, 17.7 µL, 1 equiv), acrylonitrile (0.36 mmol, 19 mg, 3.0 equiv, 23 µL), DIPEA (0.42 mmol, 54.3 mg, 73 µL, 3.5 equiv), DI water (1.2 mmol, 21.6 mg, 21.6 µL, 10 equiv), ethyl-3,5-lutidinium iodide (6.1µmol, 1.6 mg, 0.05 equiv) and 0.5 mL of stock solution of [Ir(2′,4′-dF-5-CF_3-ppy)$_2$(4,4′-dtbbpy)]PF_6 in MeCN. After the completion of the reaction 15 h, the crude was purified via automated flash chromatography using EtOAc in hexanes (0% to 100%) with product eluting at 1.0% on a 4 g alumina column to afford it in 83% yield (17 mg, 0.10 mmol) as an oil. **^{1}H NMR** (400 MHz, $CDCl_3$) δ 7.29 (dd, *J* = 8.0, 6.8 Hz, 2H), 7.22 – 7.14 (m, 3H), 2.63 (t, *J* = 7.6 Hz, 2H), 2.33 (t, *J* = 7.1 Hz, 2H), 1.67 (dq, *J* = 10.0, 7.6 Hz, 4H), 1.54 – 1.45 (m, 2H). **^{13}C NMR** (201 MHz, $CDCl_3$) δ 142.1, 128.5, 128.5, 126.0, 119.9, 35.7, 30.7, 28.4, 25.4, 17.2. NMR chemical shifts match with the literature value.[20]

17g: ((4-phenylbutyl) sulfonyl) benzene

17g

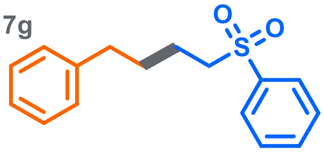

Cl, 56%

Following the general procedure C, ((4-phenylbutyl)sulfonyl)benzene was isolated using (2-chloroethyl)benzene (0.12 mmol, 16 mg, 15.7 µL, 1 equiv), phenyl vinyl sulfone (0.36 mmol, 60 mg, 3.0 equiv), DIPEA (0.42 mmol, 54.3 mg, 73.2 µL, 3.5 equiv), DI water (1.2 mmol, 21.6 mg, 21.6 µL, 10 equiv), ethyl-3,5-lutidinium iodide (6.1 µmol, 1.6 mg, 0.05 equiv) and 0.5 mL of stock solution of [Ir(2′,4′-dF-5-CF_3-ppy)$_2$(4,4′-dtbbpy)]PF_6 in MeCN. After the completion of the reaction 15 h, the crude was purified via automated flash chromatography using EtOAc in hexanes (0% to 100%) with product eluting at 1.0% on a 4 g silica column to afford it in 56% yield (18 mg, 0.06 mmol) as an oil. **^{1}H NMR** (800 MHz, $CDCl_3$) δ 7.92 – 7.84 (m, 2H), 7.66 – 7.63 (m, 1H), 7.56 (dd, *J* = 16.3, 8.5 Hz, 2H), 7.25 (d, *J* = 7.7 Hz, 2H), 7.20 – 7.16 (m, 1H), 7.12 – 7.05 (m, 2H), 3.14 – 3.07 (m, 2H), 2.59 (t, *J* = 7.5 Hz, 2H), 1.76 (tt, *J* = 7.8, 6.0 Hz, 2H), 1.70 (p, *J* = 7.5

Hz, 2H). **^{13}C NMR** (201 MHz, $CDCl_3$) δ 141.4, 139.3, 133.8, 129.4, 128.6, 128.5, 128.2, 126.2, 56.3, 35.4, 30.1, 22.4. NMR chemical shifts match with the literature value.[21]

17h: 4,4,5,5-tetramethyl-2-(4-phenylbutyl)-1,3,2-dioxaborolane

17h

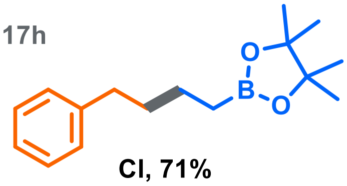

Cl, 71%

Following the general procedure C, 4,4,5,5-tetramethyl-2-(4-phenylbutyl)-1,3,2-dioxaborolane was isolated using (2-chloroethyl)benzene (0.12 mmol, 16 mg, 15.7 µL, 1 equiv), vinylboronic acid pinacol ester (0.36 mmol, 55 mg, 3.0 equiv, 61.0 µL), DIPEA (0.42 mmol, 54.3 mg, 73.2 µL, 3.5 equiv), DI water (1.2 mmol, 21.6 mg, 21.6 µL, 10 equiv), ethyl-3,5-lutidinium iodide (6.1 µmol, 1.6 mg, 0.05 equiv) and 0.5 mL of stock solution of [Ir(2′,4′-dF-5-CF_3-ppy)$_2$(4,4′-dtbbpy)]PF_6 in MeCN. After the completion of the reaction 15 h, the crude was purified via automated flash chromatography using ether in hexanes (0% to 100%) with product eluting at 1.0% on a 4 g alumina column to afford it in 71% yield (22 mg, 0.08 mmol) as an oil. **^{1}H NMR** (800 MHz, $CDCl_3$) δ 7.27 (d, J = 2.1 Hz, 1H), 7.26 – 7.24 (m, 1H), 7.17 (d, J = 7.1 Hz, 3H), 2.62 – 2.59 (m, 2H), 1.65 – 1.61 (m, 2H), 1.50 – 1.45 (m, 2H), 1.24 (s, 12H), 0.81 (t, J = 7.9 Hz, 2H). **^{13}C NMR** (201 MHz, $CDCl_3$) δ 142.9, 128.4, 128.2, 125.5, 82.9, 35.7, 34.2, 29.4, 24.8, 23.7. NMR chemical shifts match with the literature value.[22]

17i: N,5-diphenylpentanamide

17i

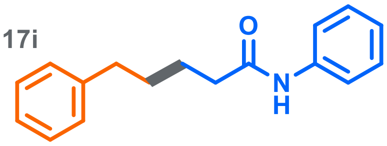

Cl, 84%

Following the general procedure C, N,5-diphenylpentanamide was isolated using (2-chloroethyl)benzene (0.12 mmol, 16 mg, 15.7 µL, 1 equiv), N-phenylacrylamide (0.36 mmol, 52.9 mg, 3.0 equiv), DIPEA (0.42 mmol, 54.3 mg, 73.2 µL, 3.5 equiv), DI water (1.2 mmol, 21.6 mg, 21.6 µL, 10 equiv), ethyl-3,5-lutidinium iodide (6.1 µmol, 1.6 mg, 0.05 equiv) and 0.5 mL of stock solution of [Ir(2′,4′-dF-5-CF_3-ppy)$_2$(4,4′-dtbbpy)]PF_6 in MeCN. After the completion of the reaction 15 h, the crude was purified via automated flash chromatography using acetone in hexanes (0% to 100%) with product eluting at 1.0% on a 4 g silica column to afford it in 84% yield (25 mg, 0.10 mmol) as an oil. **^{1}H NMR** (400 MHz, $CDCl_3$) δ 7.42 (d, J = 7.9 Hz, 2H), 7.27 – 7.18 (m, 4H), 7.14 – 7.08 (m, 2H), 7.02 (t, J = 7.2 Hz, 2H), 2.59 (t, J = 7.3 Hz, 2H), 2.29 (t, J = 7.2 Hz, 2H), 1.68 (ddd, J = 22.9, 14.5, 7.8 Hz, 4H). **^{13}C NMR** (201 MHz, $CDCl_3$) δ 171.0, 142.1, 137.9, 129.0, 128.4, 128.4, 125.8, 124.2, 119.7, 37.7, 35.7, 31.0, 25.2. NMR chemical shifts match with the literature value.[21]

17j: diethyl (4-phenylbutyl)phosphonate

17j

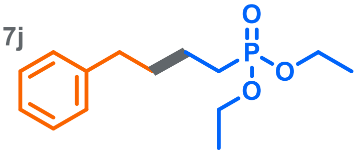

Cl, 74%

Following the general procedure C, 2-benzyl-2-(3-phenylpropyl)malononitrile was isolated using (2-chloroethyl)benzene (0.12 mmol, 16 mg, 15.7 µL, 1 equiv), diethyl vinylphosphonate (0.36 mmol, 59.0 mg, 55.3 µL 3.0 equiv), DIPEA (0.42 mmol, 54.3 mg, 73.2 µL, 3.5 equiv), DI water (1.2 mmol, 21.6 mg, 21.6 µL, 10 equiv), ethyl-3,5-lutidinium iodide (6.1 µmol mmol, 1.6 mg, 0.05 equiv) and 0.5 mL of stock solution of [Ir(2′,4′-dF-5-CF_3-ppy)$_2$(4,4′-dtbbpy)]PF_6 in MeCN. After the completion of the reaction 15 h, the crude was purified via automated flash chromatography using EtOAc in hexanes (0% to 100%) with product eluting at 4.0% on a 4 g silica column to afford it in 74% yield (24 mg, 0.08 mmol) as an oil. **^{1}H NMR** (800 MHz, $CDCl_3$) δ 7.29 (t, J = 7.6 Hz, 2H), 7.21 – 7.17 (m, 3H), 4.14 – 4.04 (m, 4H), 2.64 (t, J = 7.7 Hz, 2H), 1.80 – 1.75 (m, 2H), 1.75 – 1.70 (m, 2H), 1.70 – 1.63 (m, 2H), 1.32 (t, J = 7.1 Hz,

6H).[13]**C NMR** (201 MHz, $CDCl_3$) δ 142.0, 128.4, 128.3, 125.8, 61.4 (d, *J* = 6.2 Hz), 35.4, 32.3 (d, *J* = 16.9 Hz), 25.6 (d, *J* = 140.7 Hz), 22.1 (d, *J* = 5.1 Hz), 16.5 (d, *J* = 6.2 Hz). NMR chemical shifts match with the literature value.[21]

13k: benzyl 2-((tert-butoxycarbonyl)amino)-5-(1,3-dioxolan-2-yl)pentanoate

13k

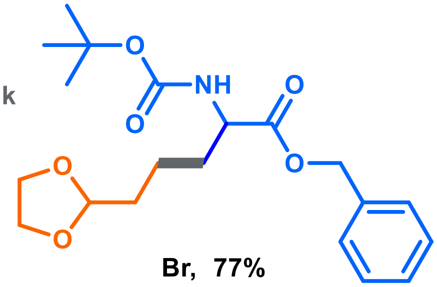

Br, 77%

Following the general procedure C, benzyl 2-((tert-butoxycarbonyl)amino)-5-(1,3-dioxolan-2-yl)pentanoate was isolated using 2-(2-bromoethyl)-1,3-dioxolane (0.12 mmol, 69.9 mg, 90 µL, 1 equiv), benzyl 2-((tert-butoxycarbonyl)(methyl)amino)acrylate (0.24 mmol, 69.9 mg, 2.0 equiv), DIPEA (0.36 mmol, 46.5 mg, 62.7 µL, 3.0 equiv), DI water (12 mmol, 216 mg, 216 µL, 100 equiv), 2-propyl-3,5-lutidinium iodide (5.4 µmol mmol, 1.5 mg, 0.05 equiv) and 0.5 mL of stock solution of $[Ir(2',4'\text{-}dF\text{-}5\text{-}CF_3\text{-}ppy)_2(4,4'\text{-}dtbbpy)]PF_6$ in MeCN. After the completion of the reaction 15 h, the crude was purified via automated flash chromatography using EtOAc in hexanes (0% to 100%) with product eluting at 5.0% on a 4 g alumina column to afford it in 77% yield (35 mg, 0.03 mmol) as an oil. [1]**H NMR** (800 MHz, $CDCl_3$) δ 7.48 – 7.10 (m, 5H), 5.25 – 5.12 (m, 2H), 5.05 (d, *J* = 8.5 Hz, 1H), 4.83 (t, *J* = 4.6 Hz, 1H), 4.37 (dt, *J* = 13.2, 6.1 Hz, 1H), 3.98 – 3.93 (m, 1H), 3.87 – 3.83 (m, 1H), 1.88 (dp, *J* = 16.2, 5.2 Hz, 1H), 1.73 – 1.62 (m, 5H), 1.46 (s, 9H), 1.30 – 1.26 (m, 1H). [13]**C NMR** (201 MHz, $CDCl_3$) δ 172.6, 155.3, 135.4, 128.5, 128.2, 127.0, 104.1, 79.8, 66.9, 65.1, 64.8, 53.5, 33.2, 32.4, 28.3, 19.7. HRMS (ESI) m/z: $[M+2H_2O+H]^+$ calcd for $C_{20}H_{34}NO_8$ 416.2279; found 416.2272.

37l: 2-benzyl-2-(3-phenylpropyl)malononitrile

37l

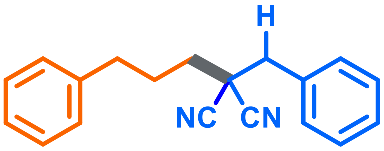

Cl, 95 %

Following the general procedure C, 2-benzyl-2-(3-phenylpropyl)malononitrile was isolated using (2-chloropropyl)benzene (0.12 mmol, 16 mg, 15.7 µL, 1 equiv), 2-benzylidenemalononitrile (0.36 mmol, 55.5 mg, 3.0 equiv), DIPEA (0.42 mmol, 54.3 mg, 73.2 µL, 3.5 equiv), DI water (1.2 mmol, 21.6 mg, 21.6 µL, 10 equiv), ethyl-3,5-lutidinium iodide (6.1 µmol, 1.6 mg, 0.05 equiv) and 0.5 mL of stock solution of $[Ir(2',4'\text{-}dF\text{-}5\text{-}CF_3\text{-}ppy)_2(4,4'\text{-}dtbbpy)]PF_6$ in MeCN. After the completion of the reaction 15 h, the crude was purified via automated flash chromatography using EtOAc in hexanes (0% to 100%) with product eluting at 4.0% on a 4 g silica column to afford it in 95% yield (31.2 mg, 0.11 mmol) as an oil. [1]**H NMR** ^{1}H NMR (400 MHz, $CDCl_3$) δ 7.39 – 6.88 (m, 10H), 3.04 (s, 2H), 2.61 (t, *J* = 7.2 Hz, 2H), 1.98 – 1.90 (m, 2H), 1.87 – 1.81 (m, 2H). [13]**C NMR** (101 MHz, $CDCl_3$) δ 140.2, 132.1, 130.2, 129.0, 128.8, 128.7, 128.4, 126.5, 115.3, 43.2, 39.3, 36.8, 27.2. HRMS (ESI) m/z: $[M+H]^+$ calcd for $C_{19}H_{18}N_2H$ 275.1543; found 275.1545.

17m: 1,4-diphenylbutane

17m

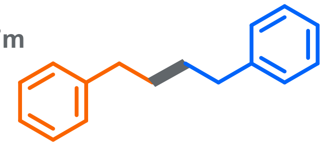

Cl, 64%

Following the general procedure C, 1,4-diphenylbutane was isolated using (2-chloroethyl)benzene (0.12 mmol, 16 mg, 15.7 µL, 1 equiv), styrene (0.36 mmol, 37 mg, 3.0 equiv, 41.3 µL), DIPEA (0.42 mmol, 54.3 mg, 73.2 µL, 3.5 equiv), DI water (1.2 mmol, 21.6 mg, 21.6 µL, 10 equiv), ethyl-3,5-lutidinium iodide (6.1 µmol, 1.6 mg, 0.05 equiv) and 0.5 mL of stock solution of $[Ir(2',4'\text{-}dF\text{-}5\text{-}CF_3\text{-}ppy)_2(4,4'\text{-}dtbbpy)]PF_6$ in MeCN. After the completion of the reaction 15 h, the crude was purified via automated flash chromatography using EtOAc in hexanes (0% to 100%) with product eluting at 1.0% on a 4 g silica column to afford it in 64% yield (16

mg, 0.07 mmol) as an oil. **^{1}H NMR** (800 MHz, $CDCl_3$) δ 7.35 – 7.30 (m, 1H), 7.29 – 7.16 (m, 8H), 7.13 – 7.10 (m, 1H), 2.67 (dq, J = 7.6, 3.9 Hz, 4H), 1.76 – 1.68 (m, 4H). **^{13}C NMR** (201 MHz, $CDCl_3$) δ 142.5, 128.4, 128.3, 125.6, 35.8, 31.1. NMR chemical shifts match with the literature value.[23]

13n: 2-(4,4-diphenylbutyl)-1,3-dioxolane

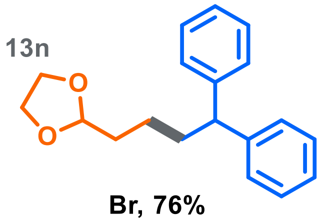

Following the general procedure C, 2-(4,4-diphenylbutyl)-1,3-dioxolane was isolated using 2-(2-bromoethyl)-1,3-dioxolane (0.12 mmol, 21 mg, 63.4 μL, 1 equiv), ethene-1,1-diyldibenzene (0.24 mmol, 64.8 mg, 21.6 μL 2.0 equiv), DIPEA (0.36 mmol, 46.5 mg, 62.7 μL, 3.0 equiv), DI water (12 mmol, 216 mg, 216 μL, 100 equiv), 2-propyl-3,5-lutidinium iodide (5.4 μmol, 1.5 mg, 0.05 equiv) and 0.5 mL of stock solution of [Ir(2′,4′-dF-5-CF_3-ppy)$_2$(4,4′-dtbbpy)]PF_6 in MeCN. After the completion of the reaction 15 h, the crude was purified via automated flash chromatography using EtOAc in hexanes (0% to 100%) with product eluting at 5.0% on a 4 g alumina column to afford it in 76% yield (25.7 mg, 0.09 mmol) as an oil. **^{1}H NMR** (800 MHz, $CDCl_3$) δ 7.34 – 7.27 (m, 5H), 7.26 – 7.17 (m, 5H), 4.82 (t, J = 4.8 Hz, 1H), 4.00 – 3.89 (m, 3H), 3.89 – 3.77 (m, 2H), 2.12 (q, J = 7.9 Hz, 2H), 1.75 – 1.70 (m, 2H), 1.43 (tt, J = 10.4, 6.5 Hz, 2H). **^{13}C NMR** (101 MHz, $CDCl_3$) δ 145.0, 128.4, 128.4, 127.8, 126.0, 104.5, 64.8, 51.4, 35.6, 33.8, 22.7. HRMS (ESI) m/z: [M+2H_2O+H] + calcd for $C_{19}H_{27}O_4$ 319.1904; found 319.1922.

13o: 2-(5-(perfluorophenoxy)-3-phenylpentyl)-1,3-dioxolane

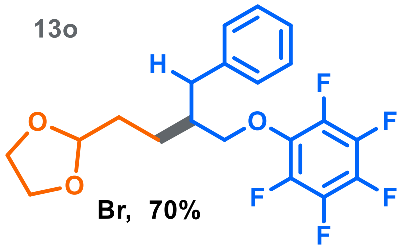

Following the general procedure C, 2-(5-(perfluorophenoxy)-3-phenylpentyl)-1,3-dioxolane was isolated using 2-(2-bromoethyl)-1,3-dioxolane (0.12 mmol, 21 mg, 14 μL, 1 equiv), (1-(cinnamyloxy)-2,3,4,5,6-pentafluorobenzene) (0.24 mmol, 72 mg, 2.0 equiv), DIPEA (0.36 mmol, 46.5 mg, 62.7 μL, 3.0 equiv), DI water (12 mmol, 216 mg, 216 μL, 100 equiv), 2-propyl-3,5-lutidinium iodide (5.4 μmol, 1.5 mg, 0.05 equiv) and 0.5 mL of stock solution of [Ir(2′,4′-dF-5-CF_3-ppy)$_2$(4,4′-dtbbpy)]PF_6 in MeCN. After the completion of the reaction 15 h, the crude was purified via automated flash chromatography using EtOAc in hexanes (0% to 100%) with product eluting at 3.0% on a 4 g alumina column to afford it in 70% yield (33 mg, 0.08 mmol) as an oil. **^{1}H NMR** (800 MHz, $CDCl_3$) δ 7.31 (s, 2H), 7.25 – 7.20 (m, 3H), 4.89 (t, J = 4.6 Hz, 1H), 4.17 – 3.83 (m, 5H), 2.87 – 2.74 (m, 2H), 2.14 – 2.05 (m, 1H), 1.88 – 1.52 (m, 4H), 1.28 (t, J = 7.1 Hz, 1H). **^{19}F NMR** (753 MHz, $CDCl_3$) δ -156.6 (d, J = 5.6 Hz), -156.6, -163.4 – -163.5 (m), -163.8 (t, J = 21.9 Hz). **^{13}C NMR** ^{13}C NMR (201 MHz, $CDCl_3$) δ 139.8, 134.0, 129.9, 129.2, 128.7, 128.4, 128.3, 126.2, 104.5, 76.6, 64.9, 64.9, 40.9, 37.2, 31.2, 25.1.HRMS (ESI) m/z: [M+2H_2O+H] + calcd for $C_{20}H_{24}F_5O_5$ 439.1538; found 439.1571.

18p: 2,2,2-trifluoroethyl 9-(9H-carbazol-9-yl)-2-methylnonanoate

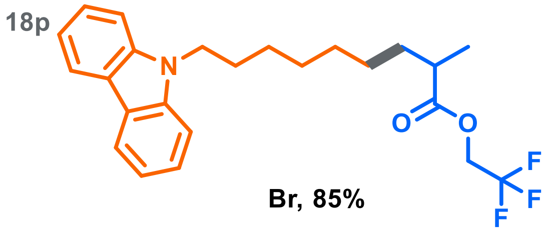

Following the general procedure C, 2,2,2-trifluoroethyl 9-(9H-carbazol-9-yl)-2-methylnonanoate was isolated using 9-(6-bromohexyl)-9H-carbazole (0.12 mmol, 39.6 mg, 1 equiv), 2,2,2-trifluoroethyl methacrylate (0.24 mmol, 40 mg, 34.1 μL, 2.0 equiv), DIPEA (0.36 mmol, 46.5 mg, 62.7 μL, 3.0 equiv), DI water (12 mmol, 216 mg, 216 μL, 100 equiv), 2-propyl-3,5-lutidinium iodide (5.4 μmol, 1.5 mg, 0.05 equiv) and 0.5 mL of stock solution of [Ir(2′,4′-dF-5-CF_3-ppy)$_2$(4,4′-dtbbpy)]PF_6 in MeCN. After the completion of the reaction 15 h, the crude was purified via automated flash chromatography using

EtOAc in hexanes (0% to 100%) with product eluting at 3.0% on a 4 g silica column to afford it in 85% yield (44 mg, 0.10 mmol) as an oil. **^{1}H NMR** (800 MHz, $CDCl_3$) δ 8.13 (d, *J* = 7.8 Hz, 2H), 7.48 (ddd, *J* = 8.2, 7.0, 1.2 Hz, 2H), 7.42 (d, *J* = 8.1 Hz, 2H), 7.24 (d, *J* = 7.4 Hz, 2H), 4.48 (p, *J* = 8.4 Hz, 2H), 4.32 (t, *J* = 7.3 Hz, 2H), 2.54 (h, *J* = 7.0 Hz, 1H), 1.89 (p, *J* = 7.4 Hz, 2H), 1.69 – 1.63 (m, 1H), 1.38 (d, *J* = 7.6 Hz, 1H), 1.37 – 1.32 (m, 3H), 1.30 – 1.26 (m, 5H), 1.19 (d, *J* = 7.0 Hz, 3H). **^{13}C NMR** (201 MHz, $CDCl_3$) δ 175.14, 140.42, 125.57, 122.98 (q, *J* = 277 Hz), 122.81, 120.35, 118.71, 108.63, 60.02 (q, *J* = 36.2 Hz), 43.04, 39.14, 33.44, 29.25, 28.94, 27.20, 26.91, 16.85.**^{19}F NMR** (753 MHz, $CDCl_3$) δ -73.8 (t, *J* = 8.3 Hz). HRMS (ESI) m/z: [M+H] + calcd for $C_{24}H_{28}F_3NO_2H$ 420.2145; found 420.2175.

17q: 2,2-dimethyl-3-(3-phenylpropyl)bicyclo[2.2.1]heptane

17q

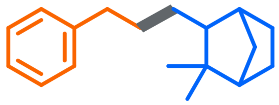

Cl, 77%

Following the general procedure C, 2,2-dimethyl-3-(3-phenylpropyl)bicyclo[2.2.1]heptane was isolated using (2-chloroethyl)benzene (0.12 mmol, 16 mg, 15.7 µL, 1 equiv), camphene (0.36 mmol, 49 mg, 3.0 equiv), DIPEA (0.42 mmol, 54.3 mg, 73.2 µL, 3.5 equiv), DI water (1.2 mmol, 21.6 mg, 21.6 µL, 10 equiv), ethyl-3,5-lutidinium iodide (6.1 µmol, 1.6 mg, 0.05 equiv) and 0.5 mL of stock solution of [Ir(2′,4′-dF-5-CF_3-ppy)$_2$(4,4′-dtbbpy)]PF_6 in MeCN. After the completion of the reaction 15 h, the crude was purified via automated flash chromatography using EtOAc in hexanes (0% to 100%) with product eluting at 1.0% on a 4 g silica column to afford it in 77% yield (22 mg, 0.09 mmol) as an oil. **^{1}H NMR (400 MHz, $CDCl_3$)** δ 7.25 – 7.14 (m, 3H), 7.11 (d, *J* = 7.3 Hz, 2H), 2.53 (dp, *J* = 9.2, 7.2 Hz, 2H), 2.04 (p, *J* = 2.3 Hz, 1H), 1.64 (dd, *J* = 3.4, 1.7 Hz, 1H), 1.54 – 1.49 (m, 2H), 1.42 – 1.34 (m, 1H), 1.29 – 0.99 (m, 9H), 0.85 (s, 3H), 0.71 (s, 2H). **^{13}C NMR (201 MHz, $CDCl_3$)** δ 143.31, 128.68, 128.53, 125.86, 50.55, 49.48, 41.32, 37.25, 36.76, 32.81, 31.53, 26.64, 25.00, 21.66, 20.18. **CI-MS:** observed [MH]+ 243.20

17r: 4-phenylbutyl acetate

17r

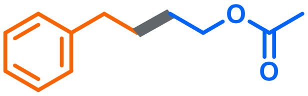

Cl, 88%

Following the general procedure C, 4-phenylbutyl acetate was isolated using (2-chloroethyl)benzene (0.12 mmol, 16 mg, 15.7 µL, 1 equiv), vinyl acetate (0.36 mmol, 31 mg, 3.0 equiv, 26.3 µL), DIPEA (0.42 mmol, 54.3 mg, 73.2 µL, 3.5 equiv), DI water (1.2 mmol, 21.6 mg, 21.6 µL, 10 equiv), ethyl-3,5-lutidinium iodide (6.1 µmol, 1.6 mg, 0.05 equiv) and 0.5 mL of stock solution of [Ir(2′,4′-dF-5-CF_3-ppy)$_2$(4,4′-dtbbpy)]PF_6 in MeCN. After the completion of the reaction 15 h, the crude was purified via automated flash chromatography using EtOAc in hexanes (0% to 100%) with product eluting at 1.0% on a 4 g silica column to afford it in 88% yield (20 mg, 0.10 mmol) as an oil. **^{1}H NMR (800 MHz, $CDCl_3$)** δ 7.37 – 7.31 (m, 2H), 7.29 – 7.24 (m, 3H), 4.33 (t, *J* = 7.2 Hz, 2H), 2.98 (t, *J* = 7.3 Hz, 2H), 2.23 – 1.84 (m, 5H), 1.46 – 0.85 (m, 2H). **^{13}C NMR (201 MHz, $CDCl_3$)** δ 170.94, 137.87, 128.92, 128.54, 126.60, 64.95, 35.12, 29.14, 29.01, 20.93. NMR chemical shifts match with the literature value.[24]

I. Photocatalytic reaction in large scale

3a

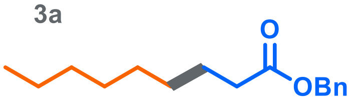

Br, 91%

Following the general procedure C, benzyl nonanoate was isolated using 1-chlorohexane (0.12 mmol, 14.4 mg, 16.4 µL, 1 equiv), benzyl acrylate (0.36 mmol, 58 mg, 3.0 equiv, 54.7 µL), DIPEA (0.42 mmol, 54.3 mg, 73.2 µL, 3.5 equiv), DI water (1.2 mmol, 21.6 mg, 21.6 µL, 10 equiv), ethyl-3,5-lutidinium iodide (0.005 mmol, 1.5 mg, 0.05 equiv) and 0.5 mL of stock solution of [Ir(2′,4′-dF-5-CF_3-ppy)$_2$(4,4′-dtbbpy)]PF_6 in MeCN. The reaction was performed on a 5.0 mmol scale by splitting the stock solution into four 18×150 mm borosilicate tubes, rather than NMR tubes, which were fitted with rubber septa. The tubes were kept equidistant from each other in the light bath and were treated otherwise identically. After reaction completion, the solutions were combined and worked up in accordance with procedure C to yield the desired product. After the completion of the reaction 15 h, the crude was purified via automated flash chromatography using EtOAc in hexanes (0% to 100%) with product eluting at 1.0% on a 24 g silica column to afford it in 91% yield (1.1 g, 4.5 mmol) as an oil.

References

(1) Singh, A.; Teegardin, K.; Kelly, M.; Prasad, K. S.; Krishnan, S.; Weaver, J. D. Facile Synthesis and Complete Characterization of Homoleptic and Heteroleptic Cyclometalated Iridium(III) Complexes for Photocatalysis. *J. Organomet. Chem.* **2015**, *776*, 51–59.

(2) Williams, D. B. G.; Lawton, M. Drying of Organic Solvents: Quantitative Evaluation of the Efficiency of Several Desiccants. *J. Org. Chem.* **2010**, *75*, 8351–8354.

(3) Zhang, Z.; Zhu, Q.; Pyle, D.; Zhou, X.; Dong, G. Methyl Ketones as Alkyl Halide Surrogates: A Deacylative Halogenation Approach for Strategic Functional Group Conversions. *J. Am. Chem. Soc.* **2023**, *145*, 21096–21103.

(4) Rathnayake, M. D.; Weaver, J. D. Coupling Photocatalysis and Substitution Chemistry to Expand and Normalize Redox-Active Halides. *Org. Lett.* **2021**, *23*, 2036–2041.

(5) Loh, Y. Y.; Nagao, K.; Hoover, A. J.; Hesk, D.; Rivera, N. R.; Colletti, S. L.; Davies, I. W.; MacMillan, D. W. C. Photoredox-Catalyzed Deuteration and Tritiation of Pharmaceutical Compounds. *Science* **2017**, *358*, 1182–1187.

(6) Xin, H.; Duan, X.; Liu, L.; Guo, L. Metal-Free, Visible-Light-Induced Selective C−C Bond Cleavage of Cycloalkanones with Molecular Oxygen. *Chemistry A European J* **2020**, *26*, 11690–11694.

(7) Kim, S.; Lee, S. Electrochemical Synthesis of Sulfinic and Sulfonic Esters from Sulfonyl Hydrazides. *Org. Biomol. Chem.* **2024**, *22*, 4436–4444.

(8) Chen, Y.; Wang, W.; Qin, H.; Liu, R.; Zhang, Q. Solvent-Free and under Vacuum *O* -Benzylation of Carboxylic Acids by Using a Rotary Evaporator. *ChemistrySelect* **2023**, *8*, e202304145.

(9) Hanumanthu, R.; Weaver, J. D. Cooperative Catalytic Coupling of Benzyl Chlorides and Bromides with Electron-Deficient Alkenes. *Org. Lett.* **2024**, *26*, 5248–5252.

(10) Kattamuri, P. V.; West, J. G. Hydrogenation of Alkenes via Cooperative Hydrogen Atom Transfer. *J. Am. Chem. Soc.* **2020**, *142*, 19316–19326.

(11) Fieser, M. E.; Schimler, S. D.; Mitchell, L. A.; Wilborn, E. G.; John, A.; Hogan, L. T.; Benson, B.; LaPointe, A. M.; Tolman, W. B. Dual-Catalytic Decarbonylation of Fatty Acid Methyl Esters to Form Olefins. *Chem. Commun.* **2018**, *54*, 7669–7672.

(12) Dong, J.; Wang, X.; Wang, Z.; Song, H.; Liu, Y.; Wang, Q. Visible-Light-Initiated Manganese-Catalyzed Giese Addition of Unactivated Alkyl Iodides to Electron-Poor Olefins. *Chem. Commun.* **2019**, *55*, 11707–11710.

(13) Ma, Y.-Q.; Zhang, M.; Tian, S.-K. Silyl Radical as an Isocyanide Transfer Agent for Giese-Type Reactions Involving Aliphatic Amines. *Org. Lett.* **2024**, *26*, 5172–5176.

(14) Ji, C.-L.; Han, J.; Li, T.; Zhao, C.-G.; Zhu, C.; Xie, J. Photoinduced Gold-Catalyzed Divergent Dechloroalkylation of Gem-Dichloroalkanes. *Nat. Catal.* **2022**, *5*, 1098–1109.

(15) Wang, H.; Long, X.; Chen, J.; Lin, B.; Ding, Y.; Gao, J.; Jiang, H.; Chen, L.; Shen, C.; Chen, L. Photo-Induced Oxidative Esterification via Aldehyde and Alkyl Halides Using Dimethyl Carbonate as Green Solvent. *J. Mol. Struct.* **2024**, *1317*, 139180.

(16) Yoshida, M.; Otaka, H.; Doi, T. An Efficient Partial Reduction of α,β-Unsaturated Esters Using DIBAL-H in Flow. *Eur J Org Chem* **2014**, *2014*, 6010–6016.

(17) Takeda, N.; Furuishi, M.; Nishijima, Y.; Futaki, E.; Ueda, M.; Shinada, T.; Miyata, O. Chiral Isoxazolidine-Mediated Stereoselective Umpolung α-Phenylation of Methyl Ketones. *Org. Biomol. Chem.* **2018**, *16*, 8940–8943.

(18) Mason, J.; Murphree, S. Microwave-Assisted Aqueous Krapcho Decarboxylation. *Synlett* **2013**, *24*, 1391–1394.

(19) Guo, L.; Ma, X.; Fang, H.; Jia, X.; Huang, Z. A General and Mild Catalytic α-Alkylation of Unactivated Esters Using Alcohols. *Angew. Chem. Int. Ed.* **2015**, *54*, 4023–4027.

(20) Bhunia, A.; Bergander, K.; Studer, A. Cooperative Palladium/Lewis Acid-Catalyzed Transfer Hydrocyanation of Alkenes and Alkynes Using 1-Methylcyclohexa-2,5-Diene-1-Carbonitrile. *J. Am. Chem. Soc.* **2018**, *140*, 16353–16359.

(21) Wang, J.-J.; Yu, W. Hydrosulfonylation of Unactivated Alkenes by Visible Light Photoredox Catalysis. *Org. Lett.* **2019**, *21*, 9236–9240.

(22) Wang, H.; Zhao, J.-F.; Zhu, X.-L.; Tian, Q.-Q.; He, W. Photoinduced Borylation of the Inert C(Sp^3)–O Bond of Alkyl Heteroaryl Ethers. *Org. Lett.* **2023**, *25*, 6485–6489.

(23) Messa, F.; Dilauro, G.; Paparella, A. N.; Silvestri, L.; Furlotti, G.; Iacoangeli, T.; Perrone, S.; Salomone, A. Deep Eutectic Solvents Meet Safe, Scalable and Sustainable Hydrogenations Enabled by Aluminum Powder and Pd/C. *Green Chem.* **2022**, *24*, 4388–4394.

(24) Lu, P.; Hou, T.; Gu, X.; Li, P. Visible-Light-Promoted Conversion of Alkyl Benzyl Ether to Alkyl Ester or Alcohol via O-α-Sp^3 C–H Cleavage. *Org. Lett.* **2015**, *17*, 1954–1957.

Salts NMR Spectra

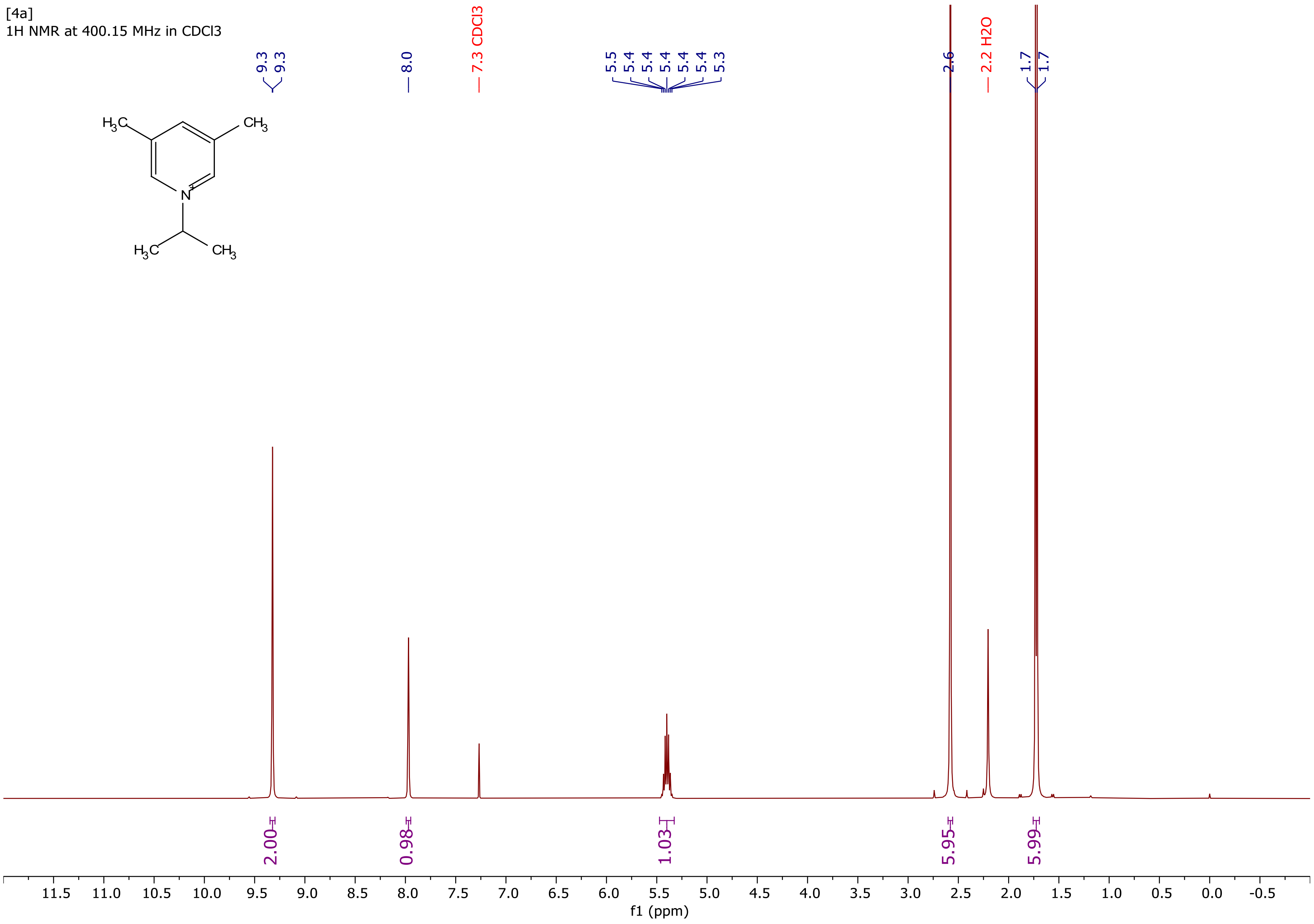

[4a]
13C NMR at 201.27 MHz in CDCl3

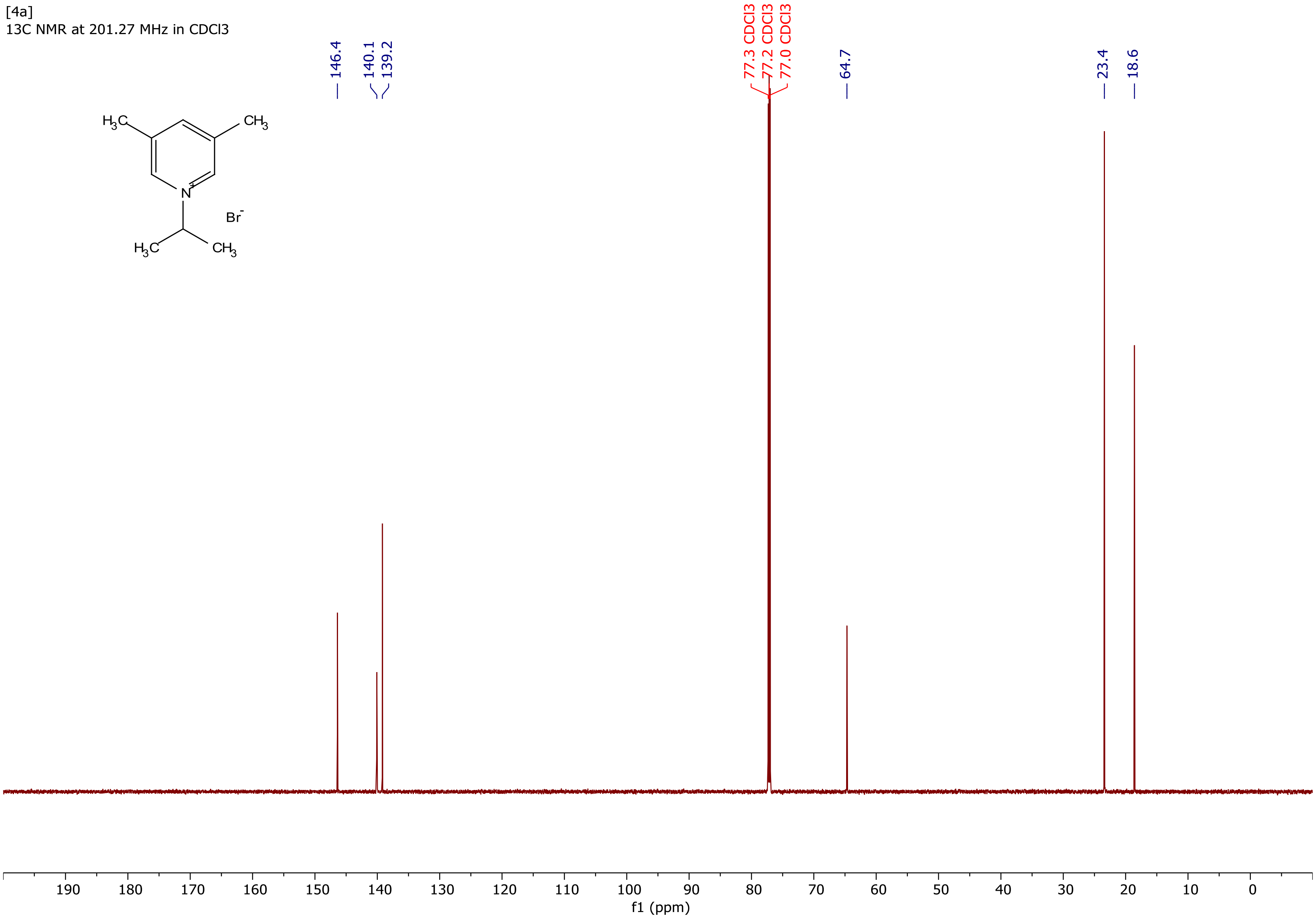

[4b]
1H NMR at 800.34 MHz in CDCl3

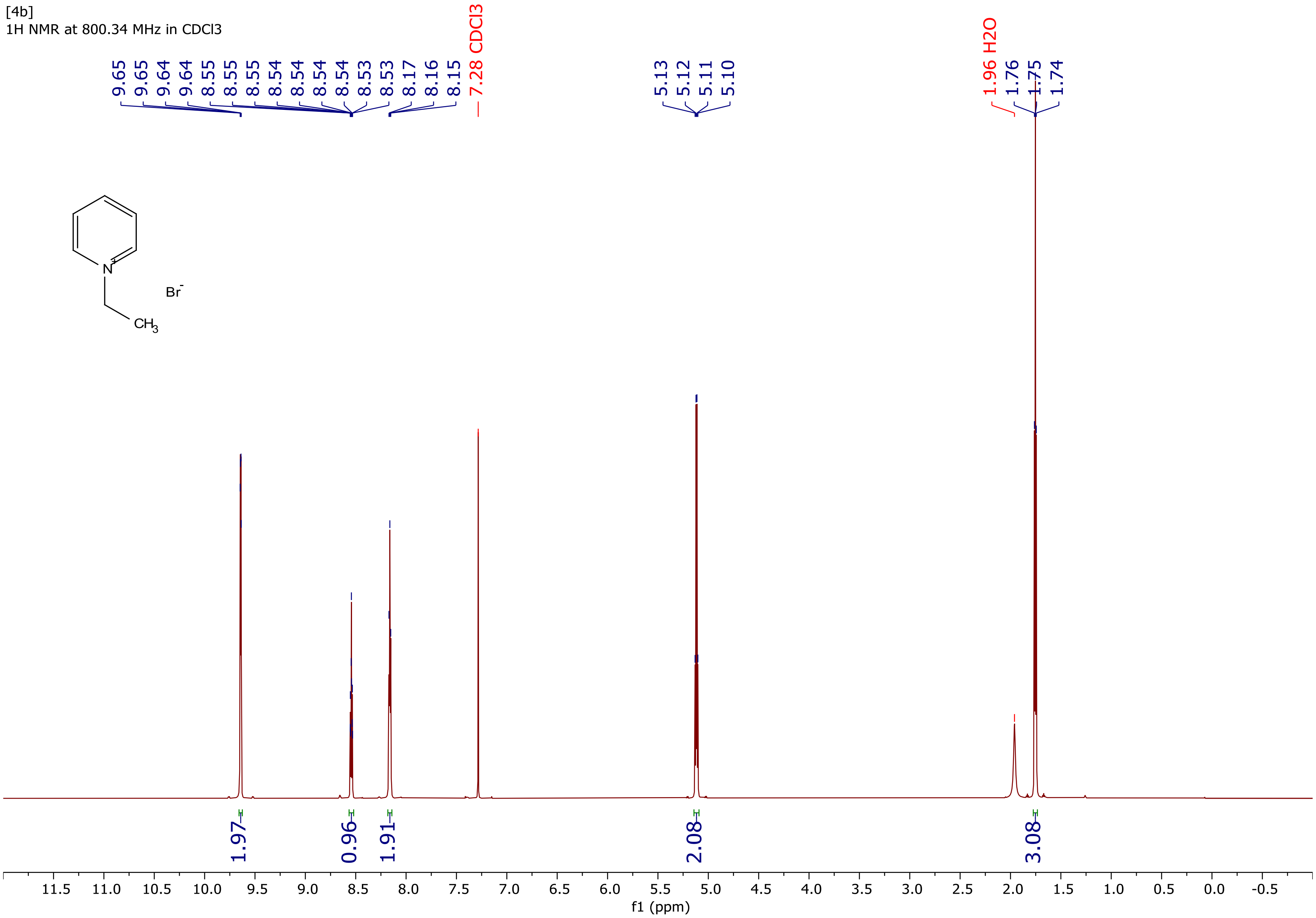

[4b]
13C NMR at 201.27 MHz in CDCl3

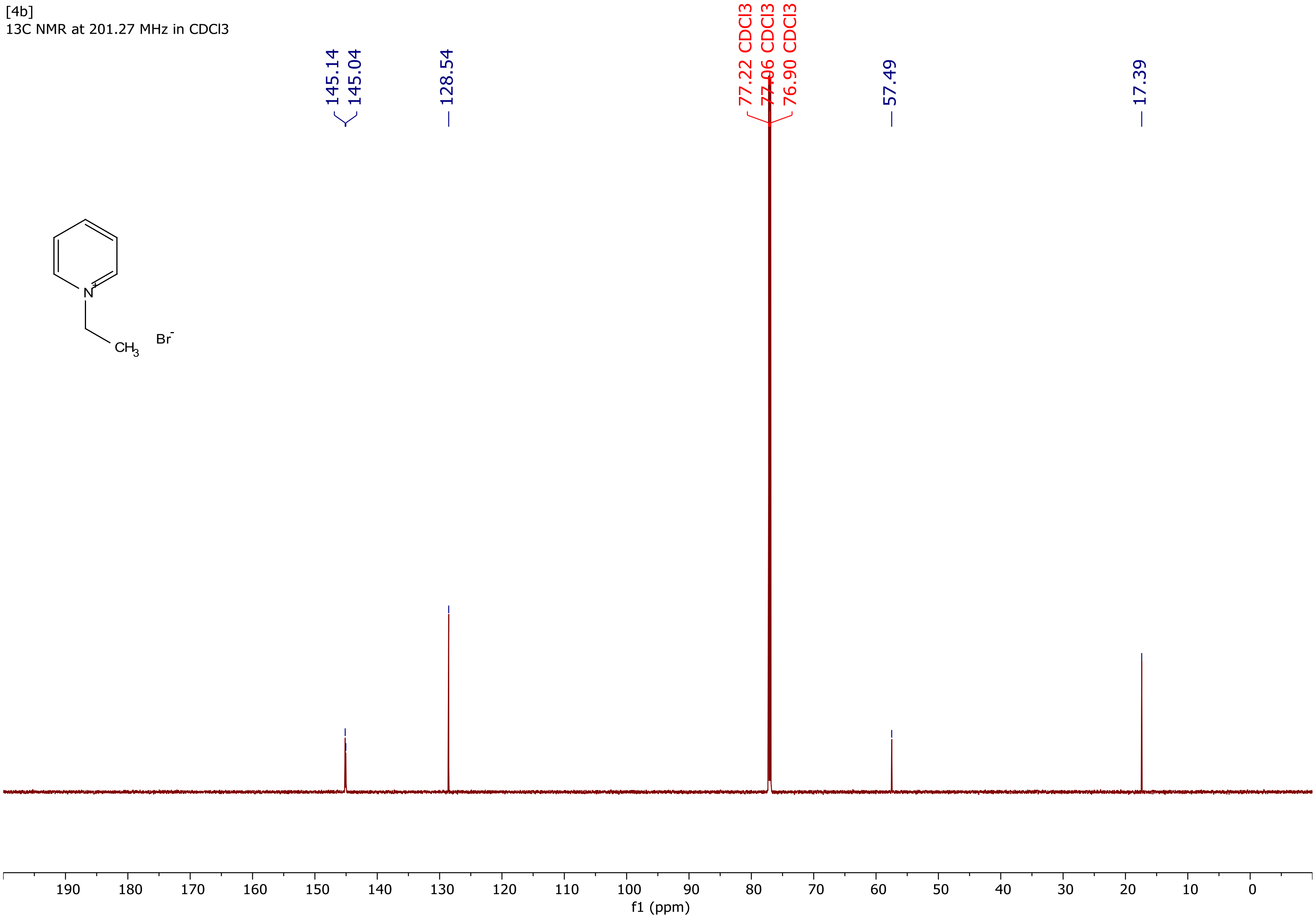

[4c]
1H NMR at 800.34 MHz in CDCl3

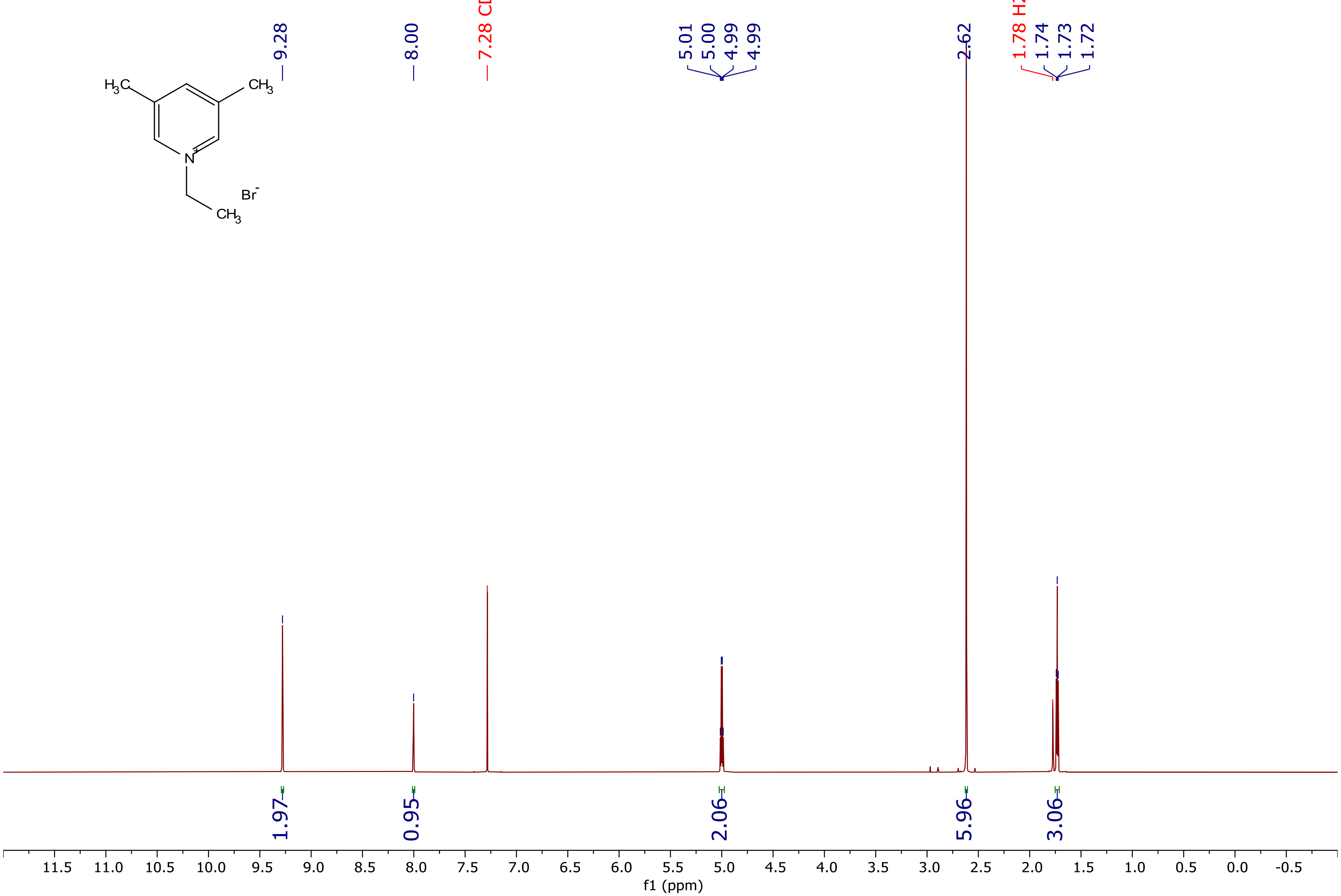

[4c]
13C NMR at 201.27 MHz in CDCl3

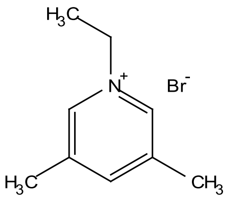

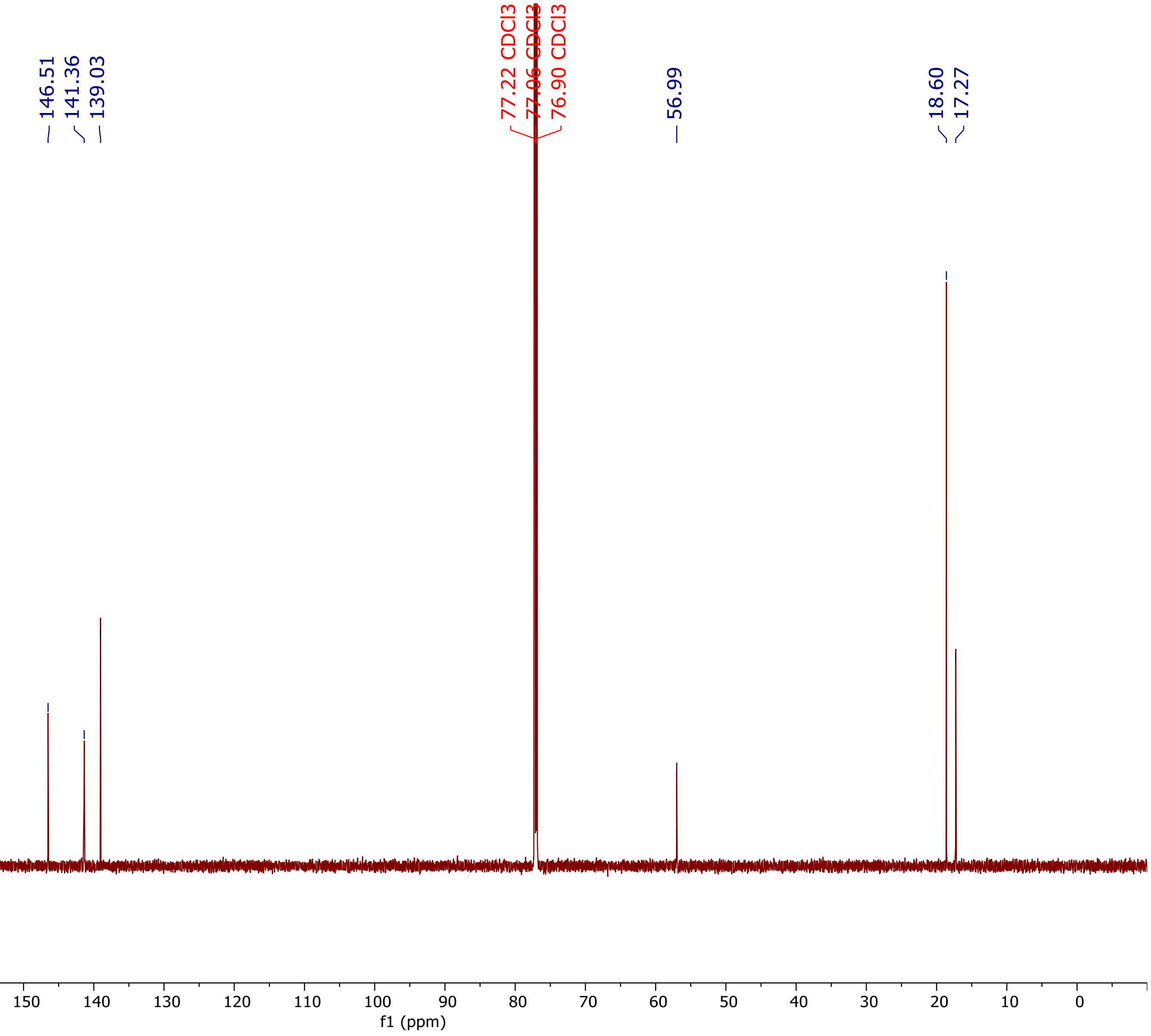

[4d]
1H NMR at 800.34 MHz in D2O

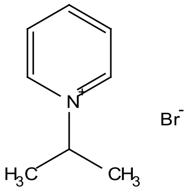

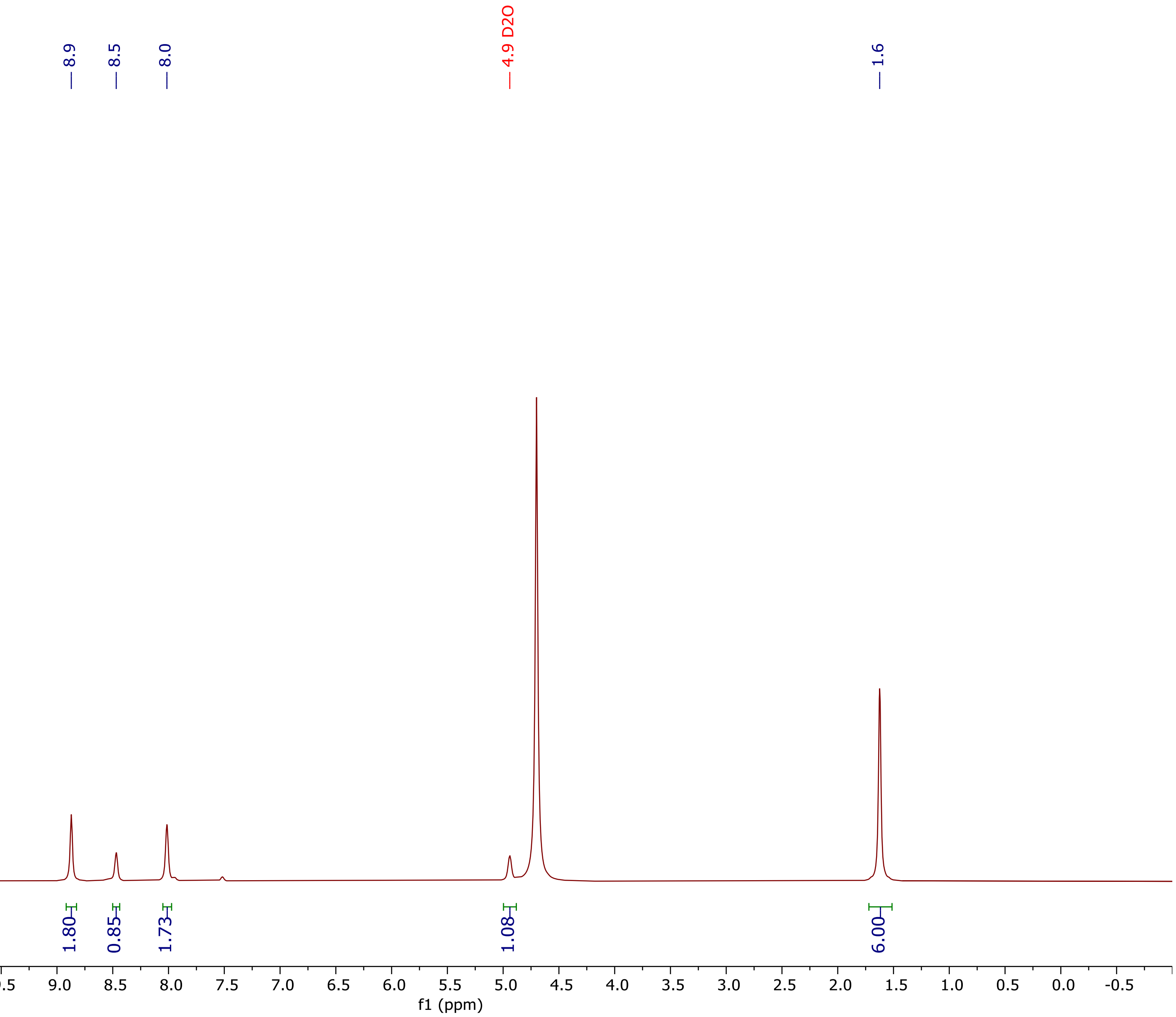

[4d]
13C NMR at 201.27 MHz in D2O

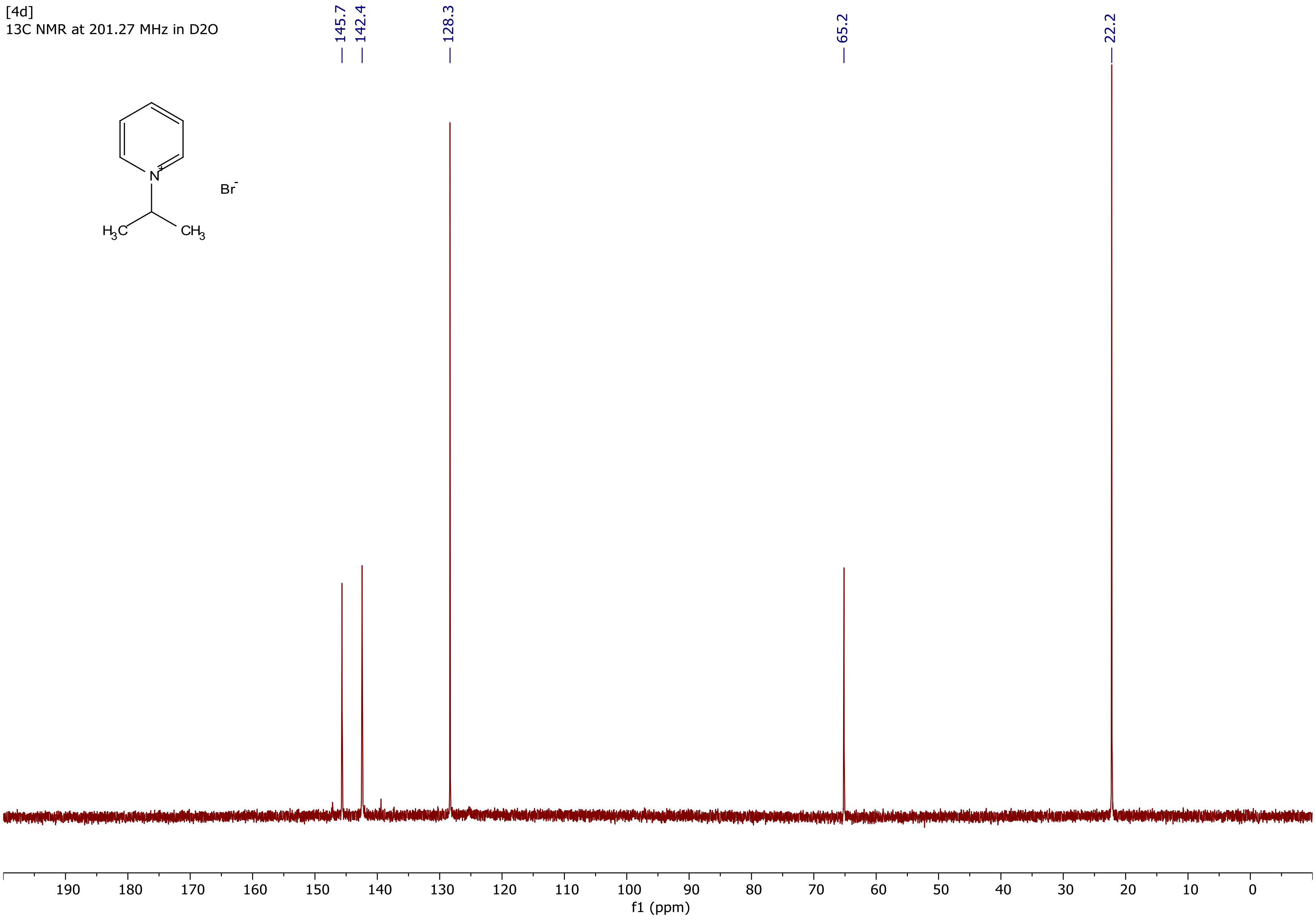

[4e]
1H NMR at 800.34 MHz in CDCl3

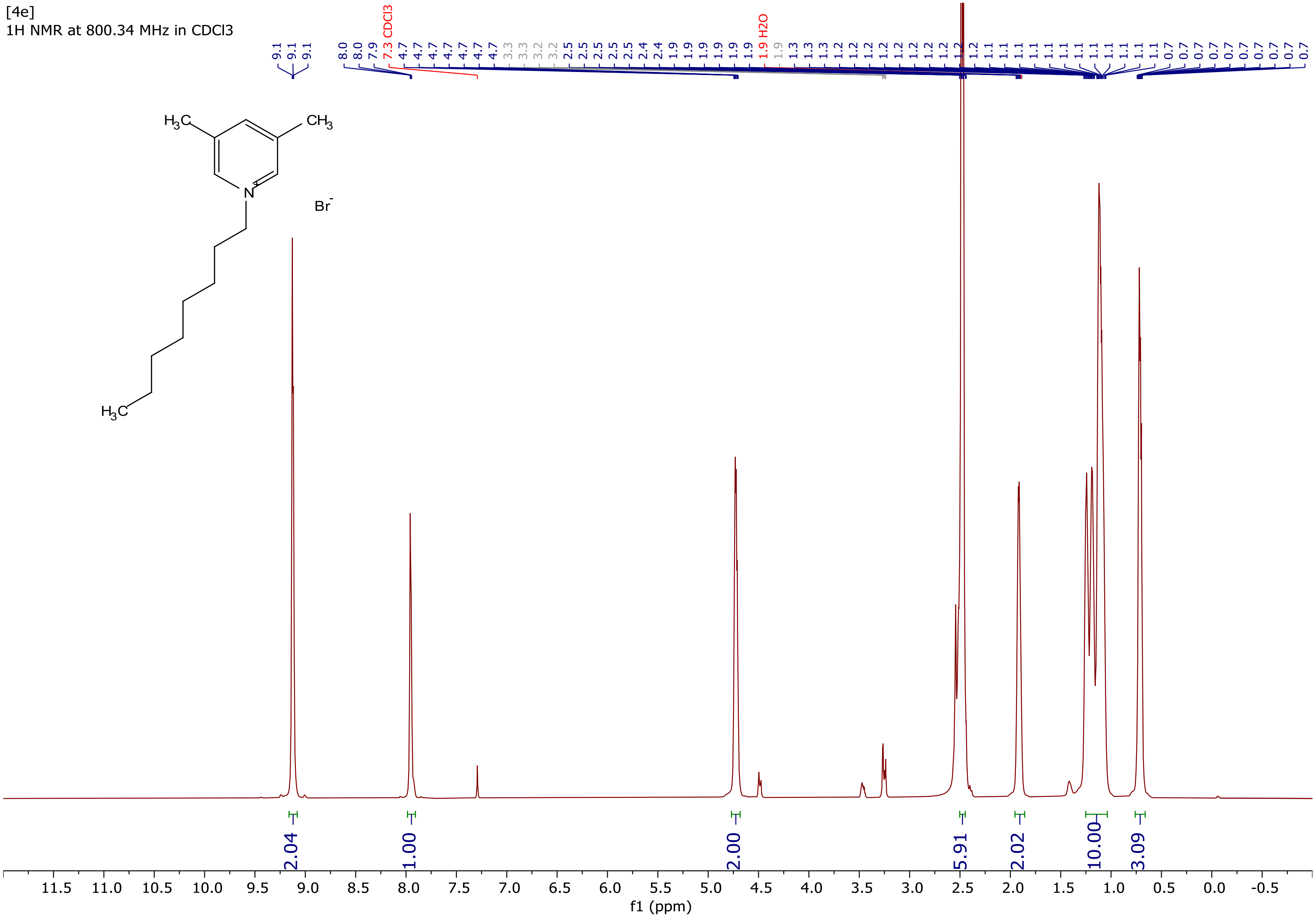

[4e]
13C NMR at 201.27 MHz in CDCl3

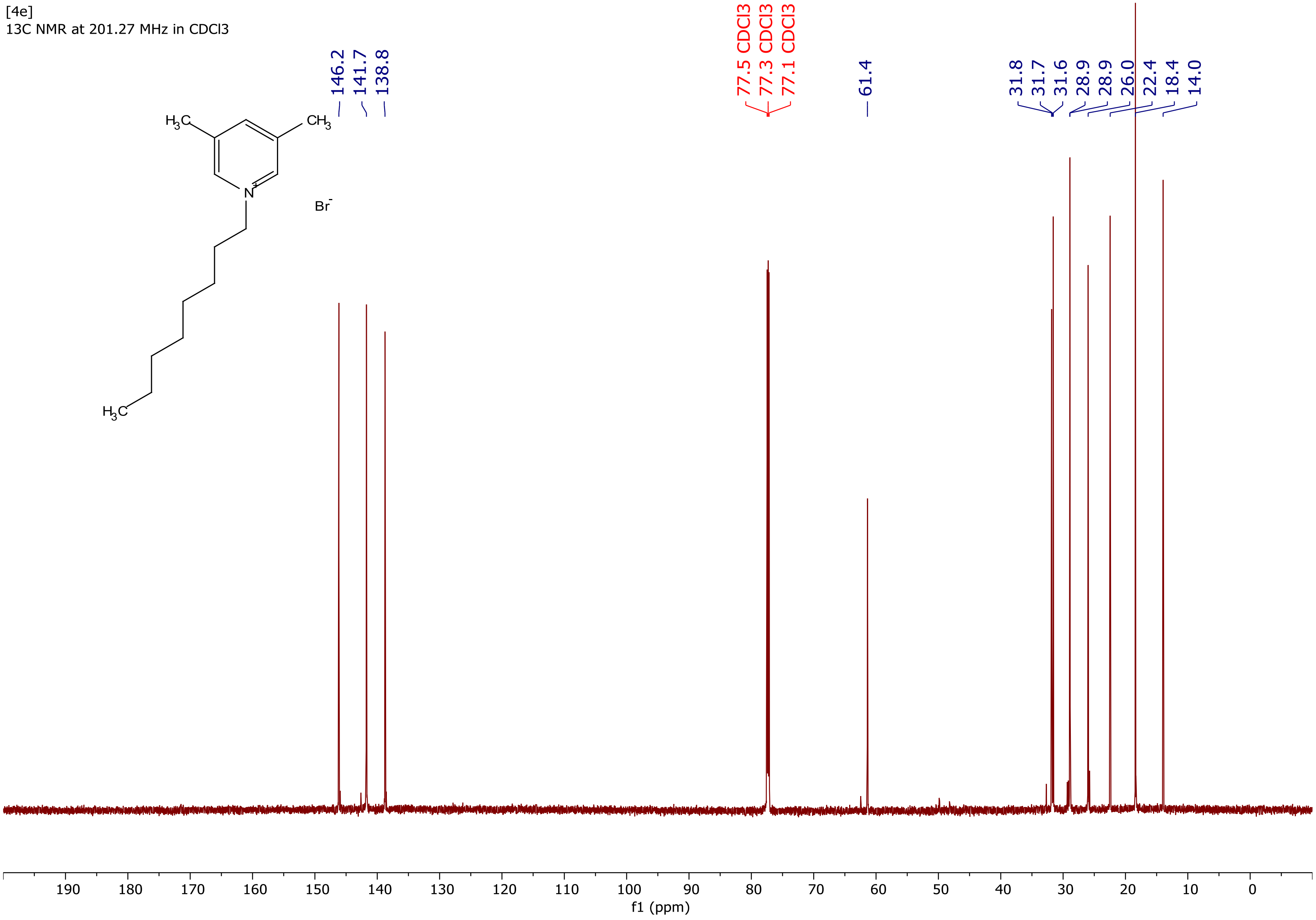

[4f]
1H NMR at 800.34 MHz in CDCl3

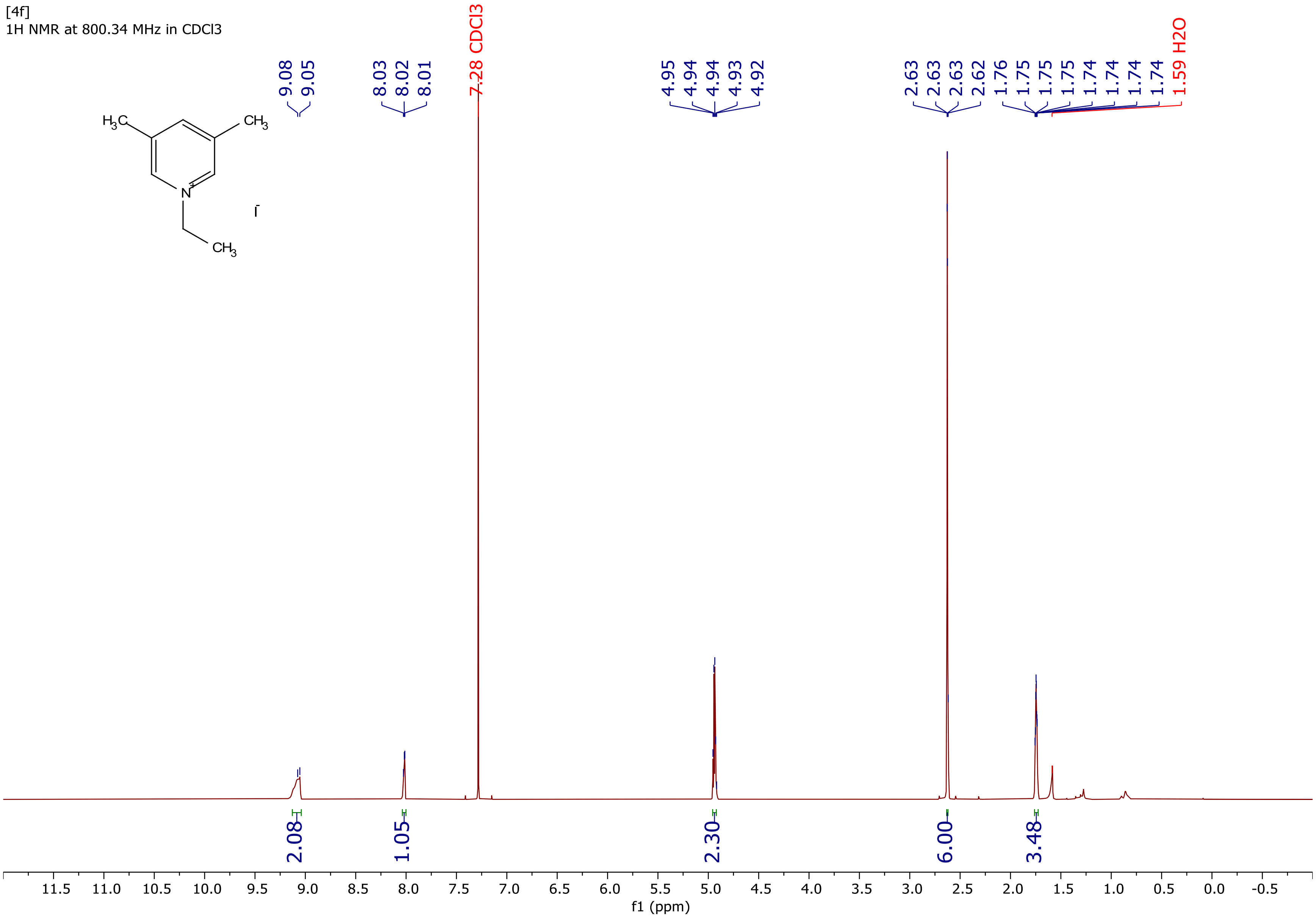

[4f]
13C NMR at 201.27 MHz in CDCl3

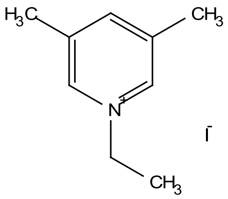

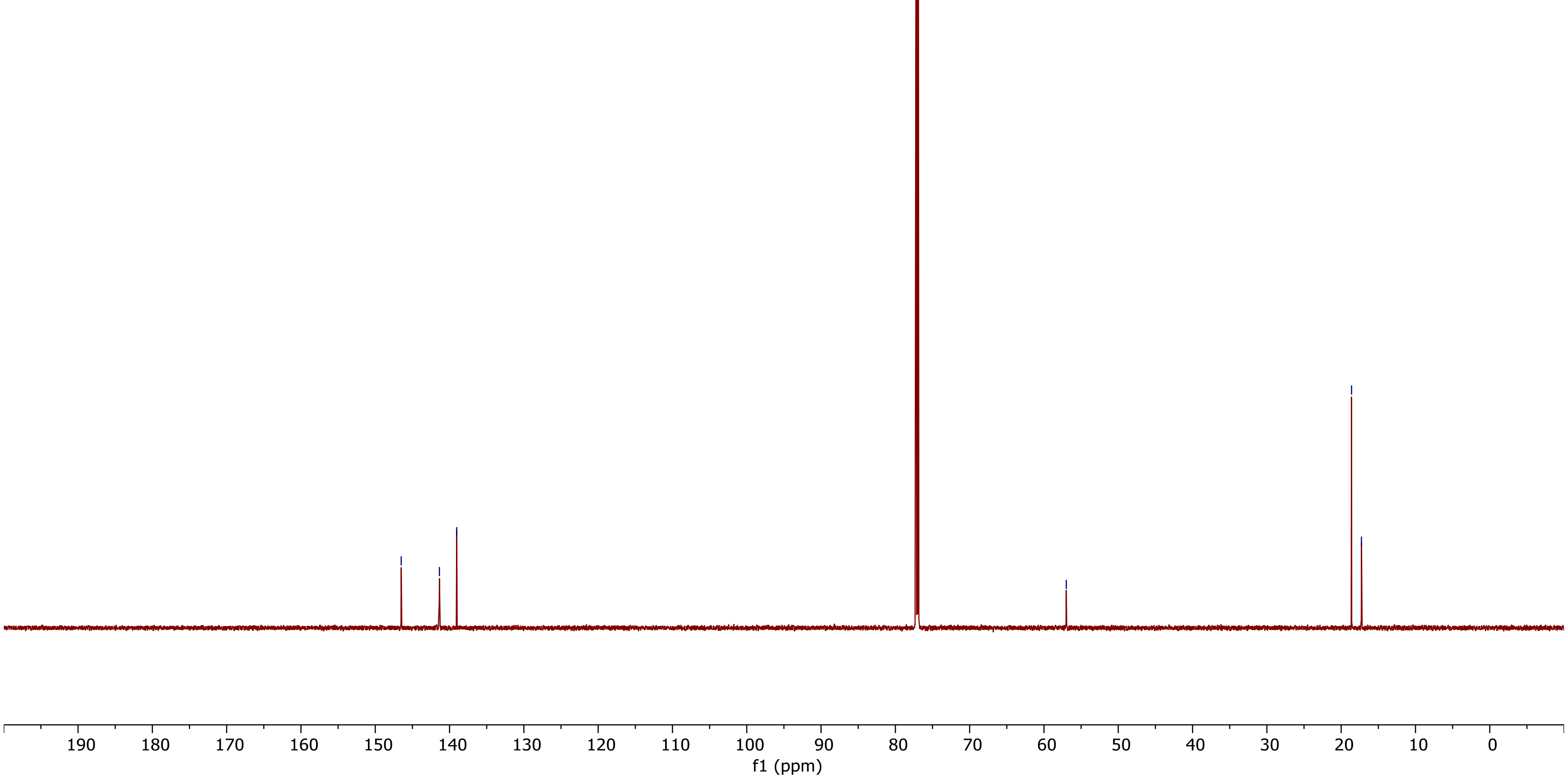

[4g]
1H NMR at 800.34 MHz in CDCl3

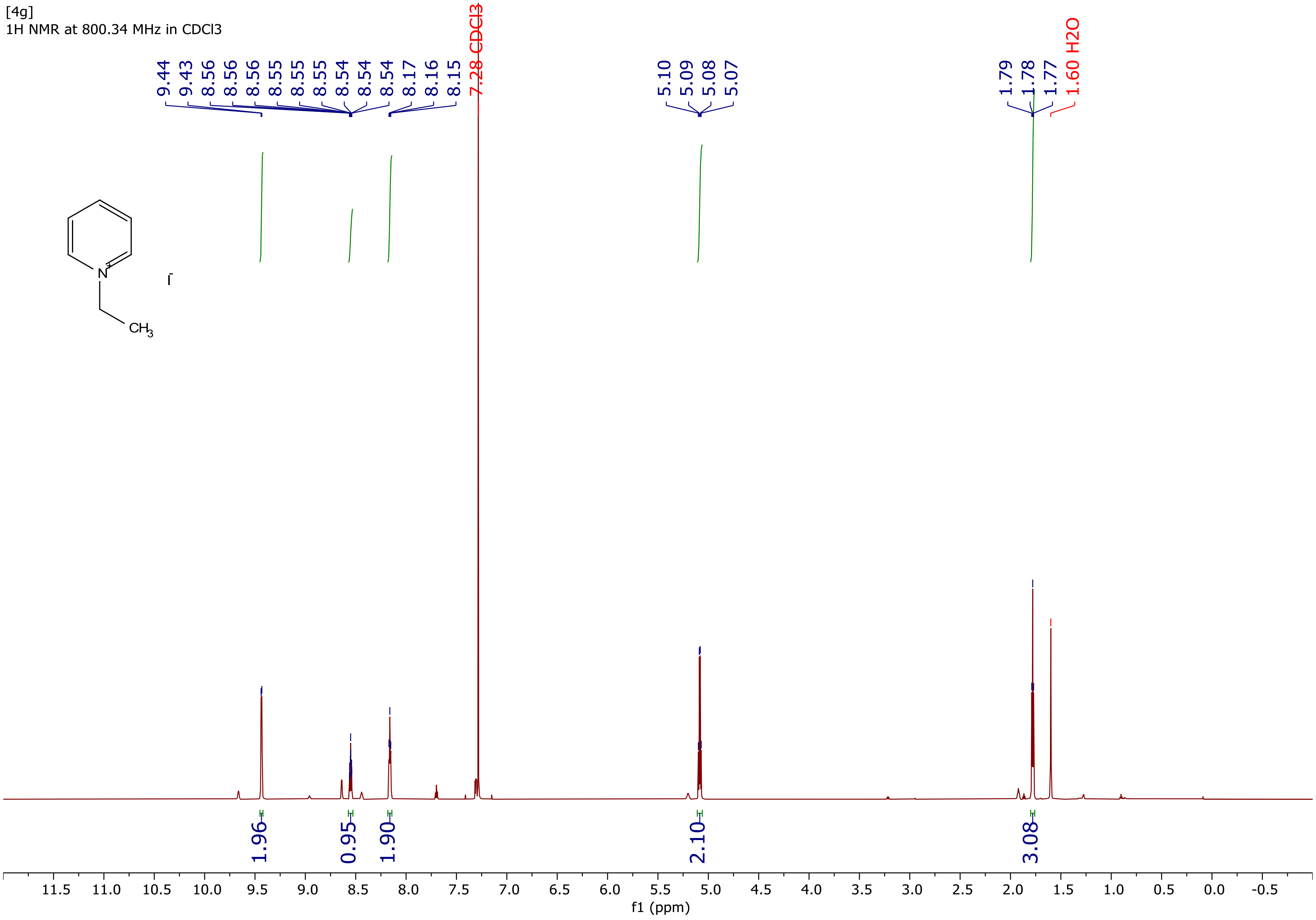

[4g]
13C NMR at 201.27 MHz in CDCl3

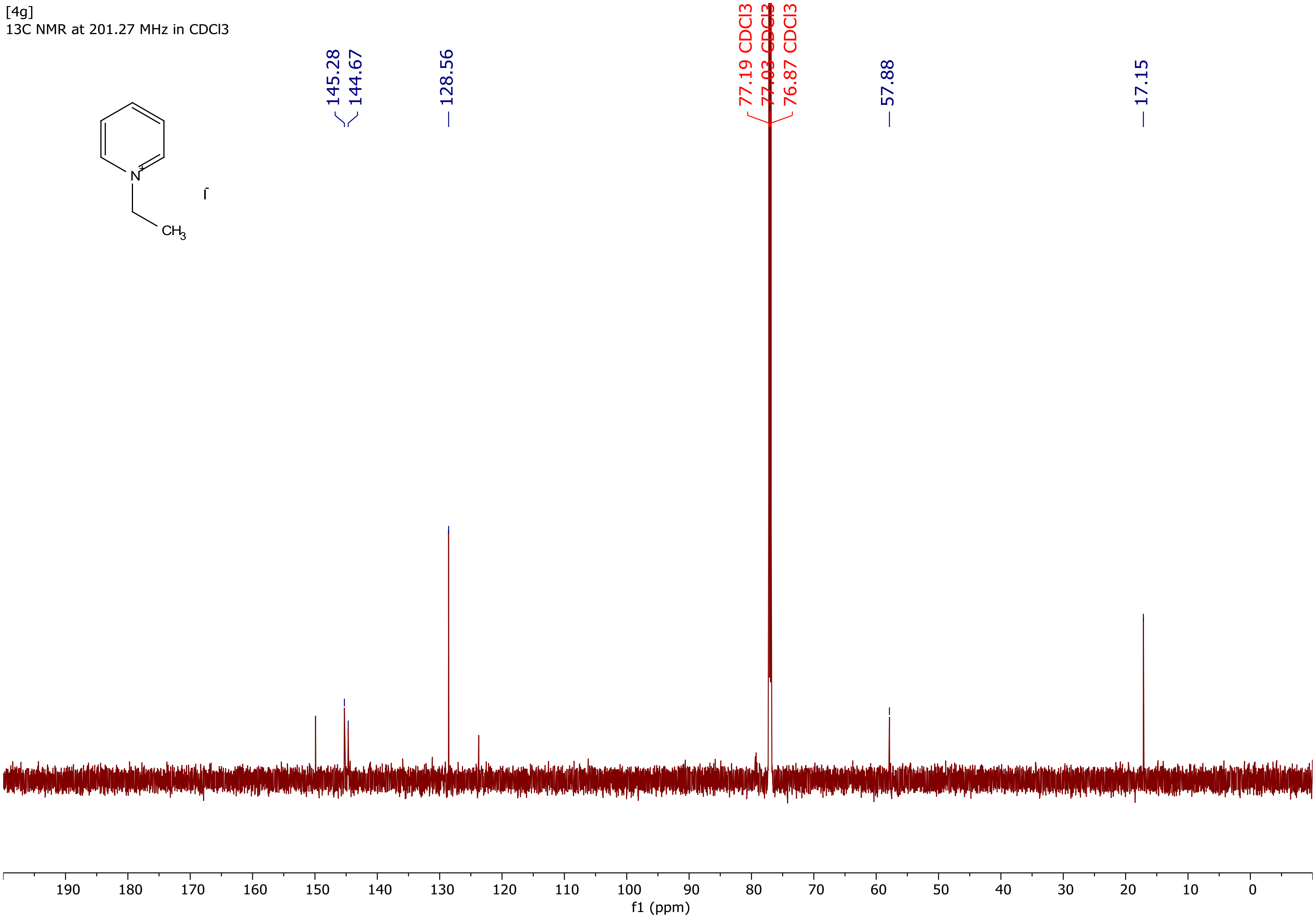

[4h]
1H NMR at 800.34 MHz in CDCl3

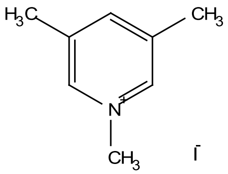

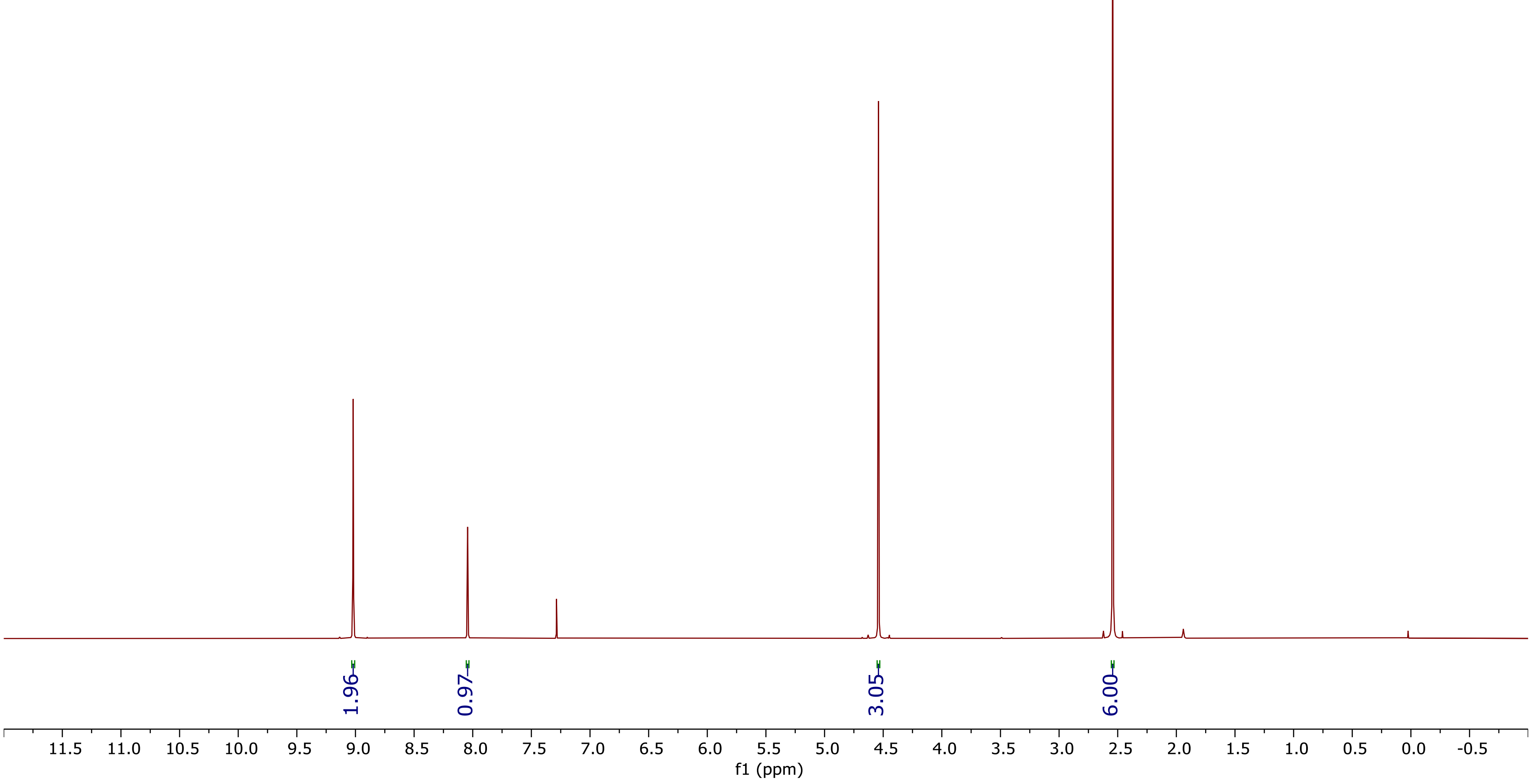

[4h]
13C NMR at 201.27 MHz in CDCl3

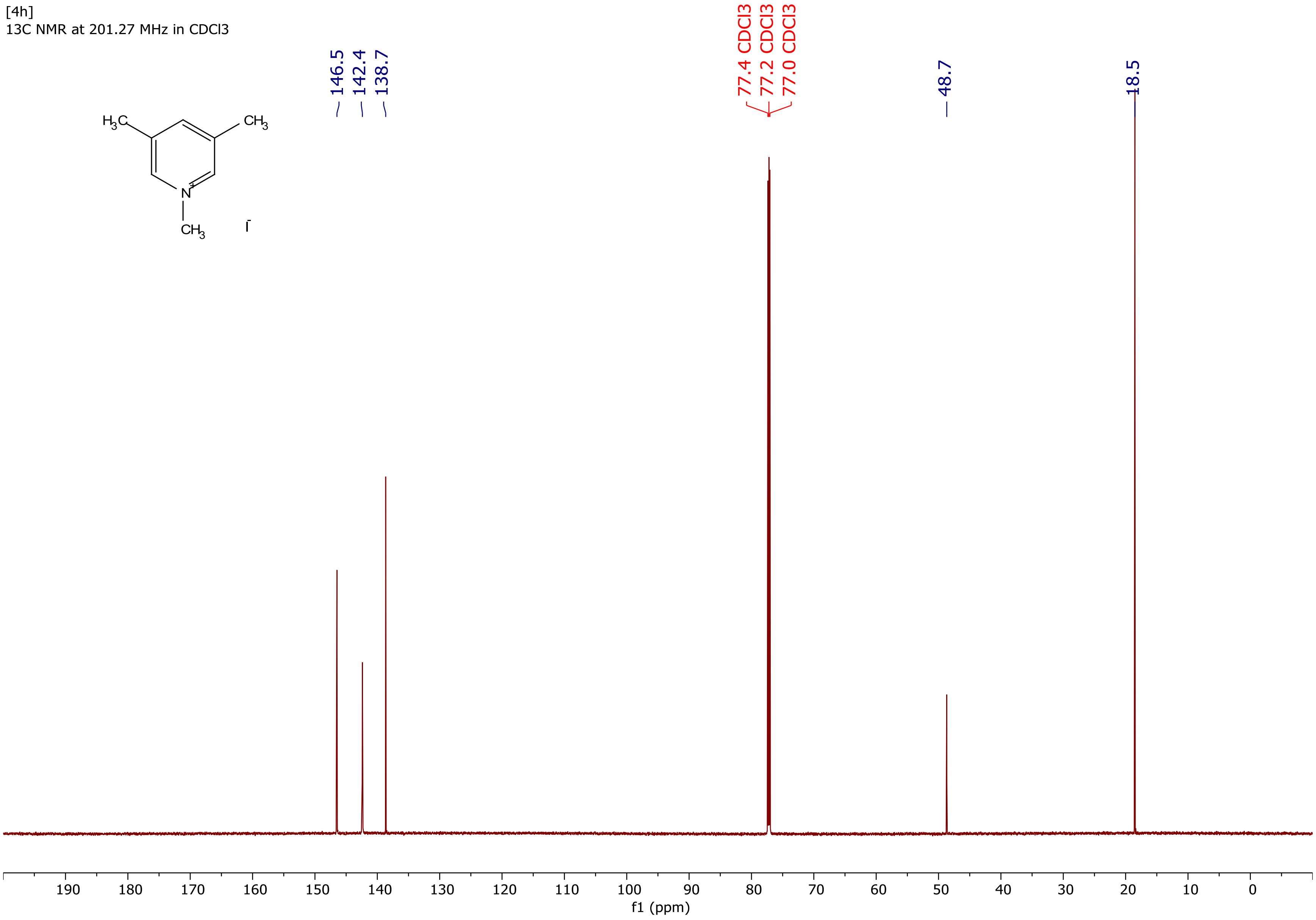

[4i]
1H NMR at 400.15 MHz in D2O

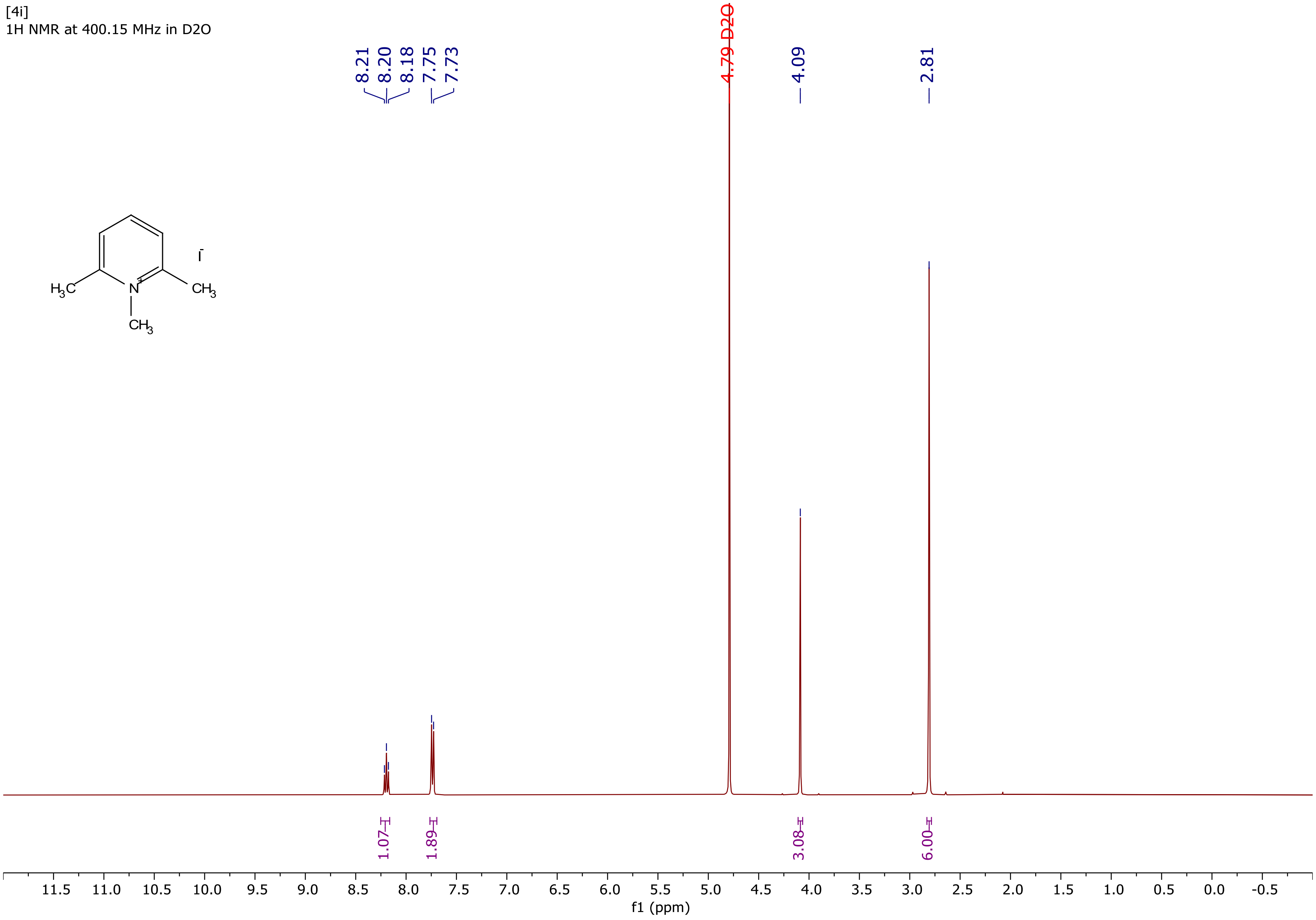

[4i]
13C NMR at 100.63 MHz in D2O

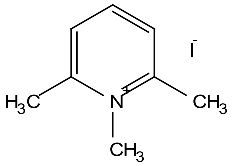

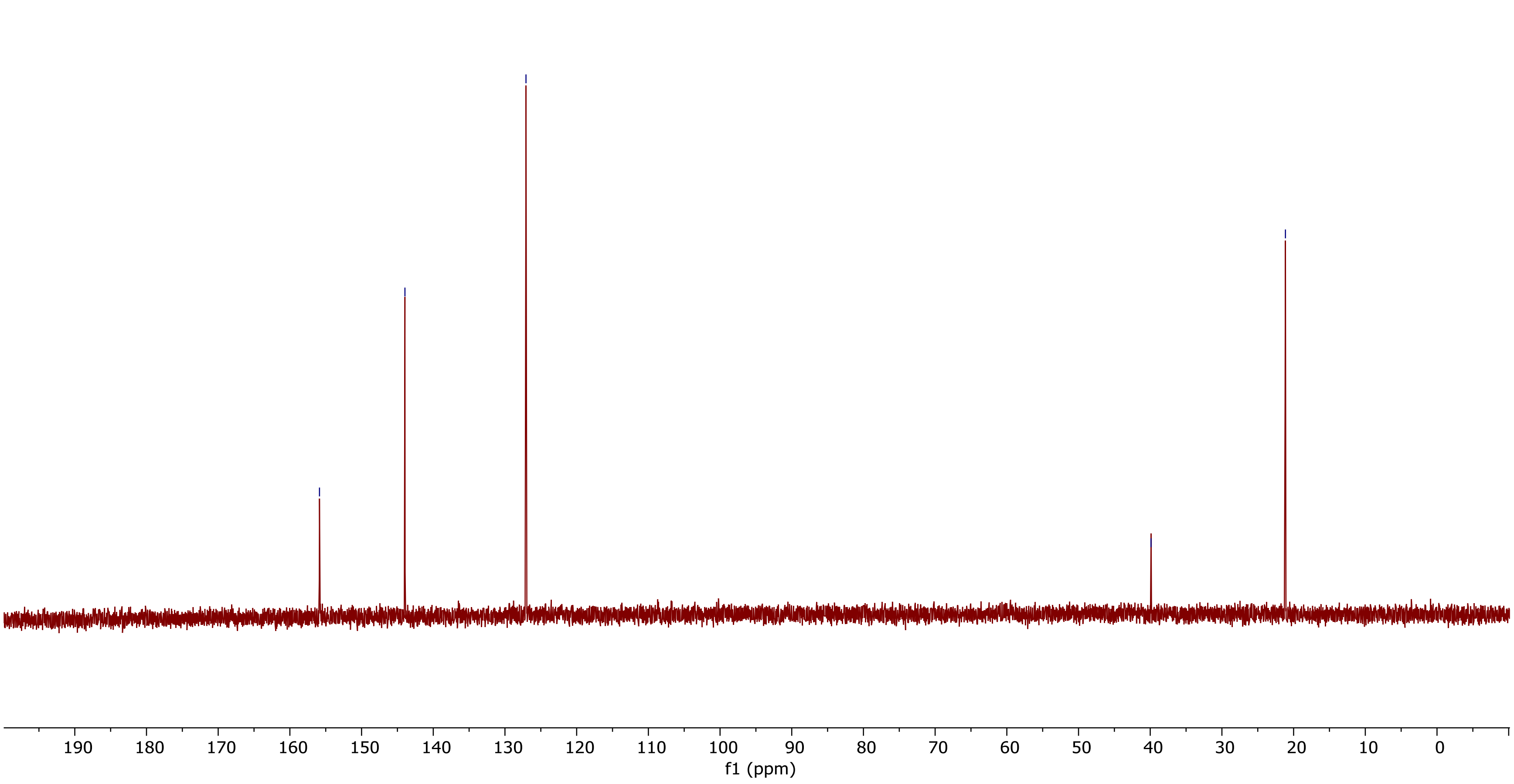

[4j]
1H NMR at 800.34 MHz in CDCl3

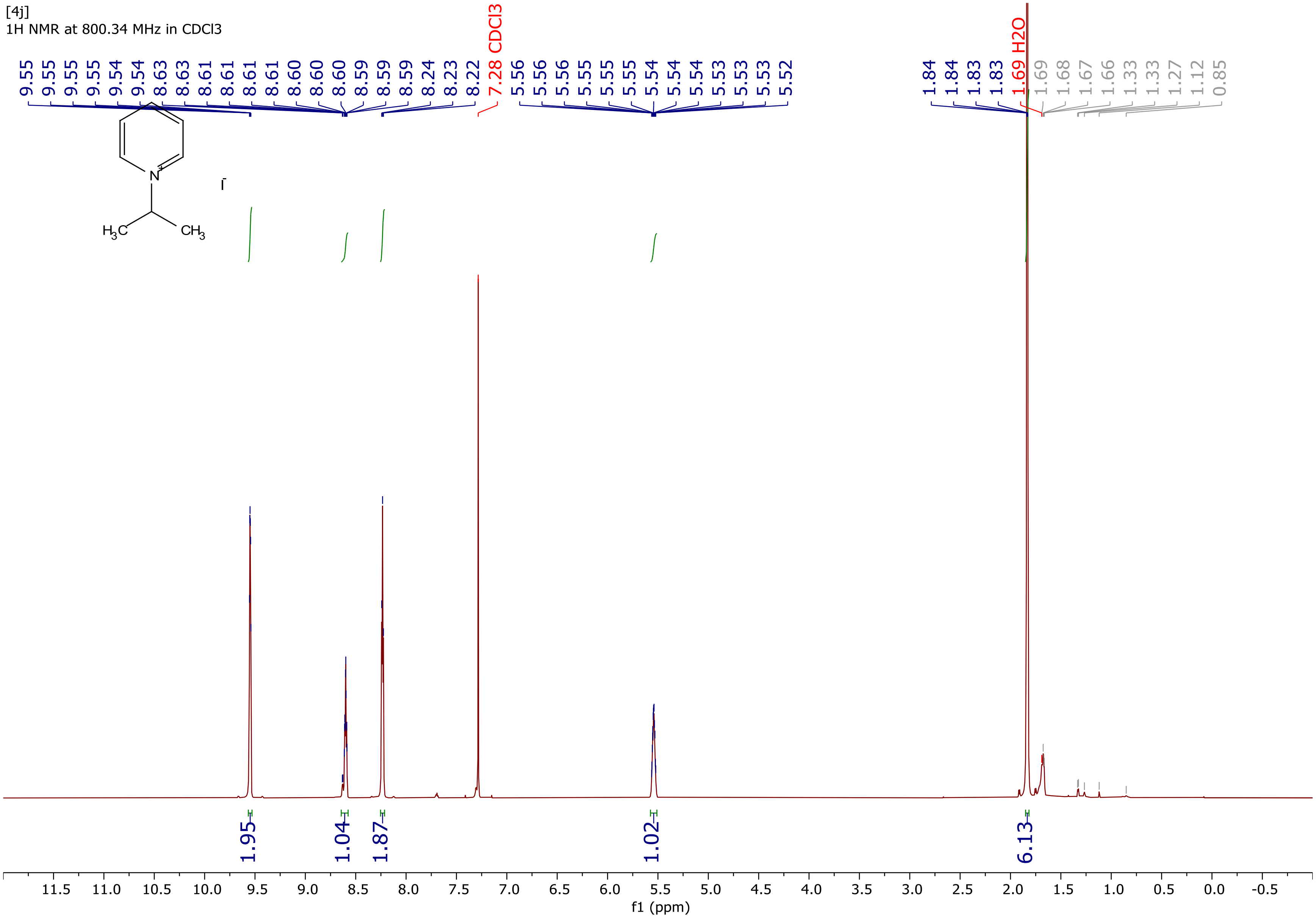

S

[4j]
13C NMR at 201.27 MHz in CDCl3

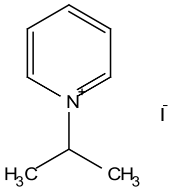

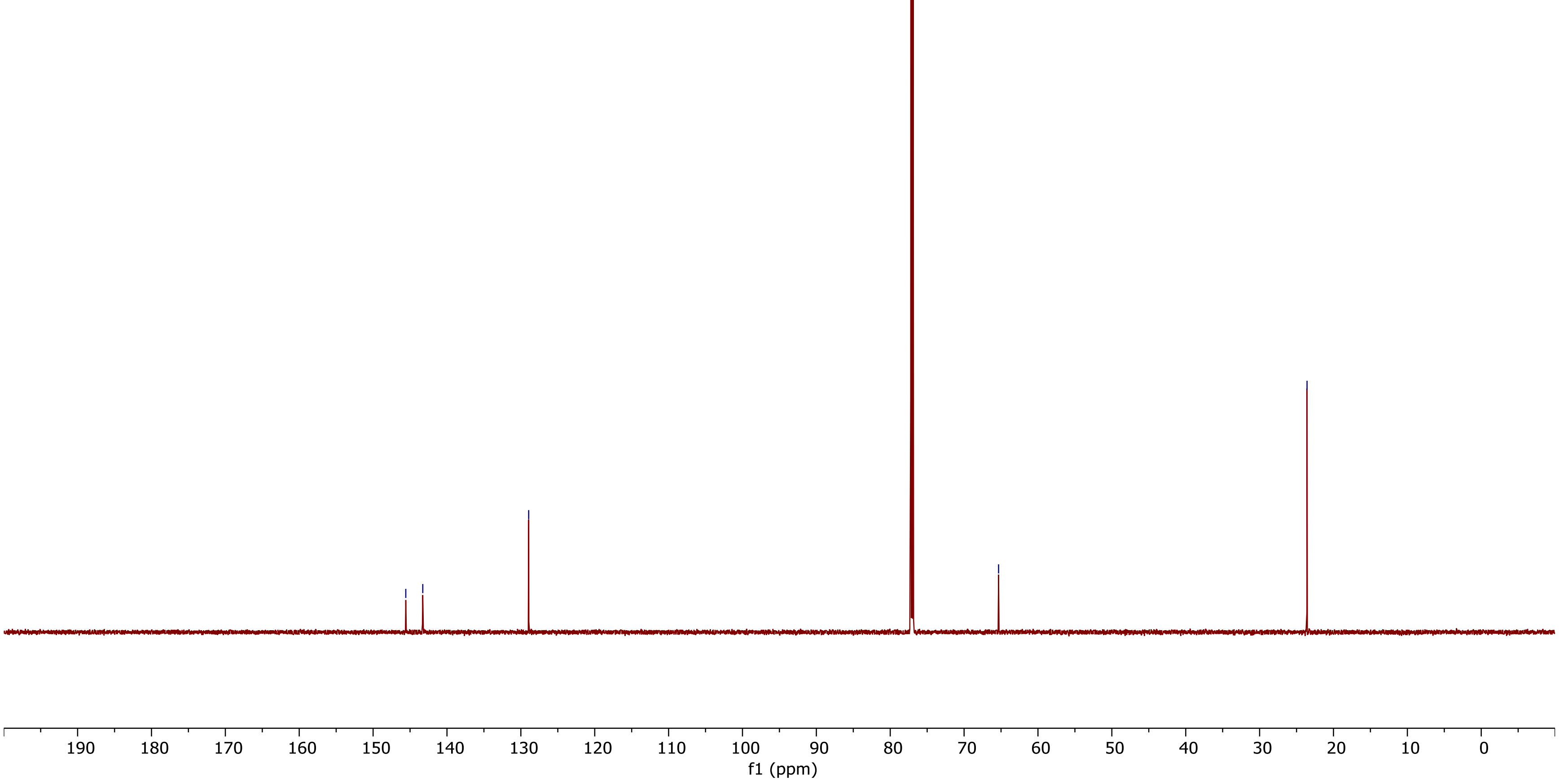

[4k]
1H NMR at 400.15 MHz in CDCl3

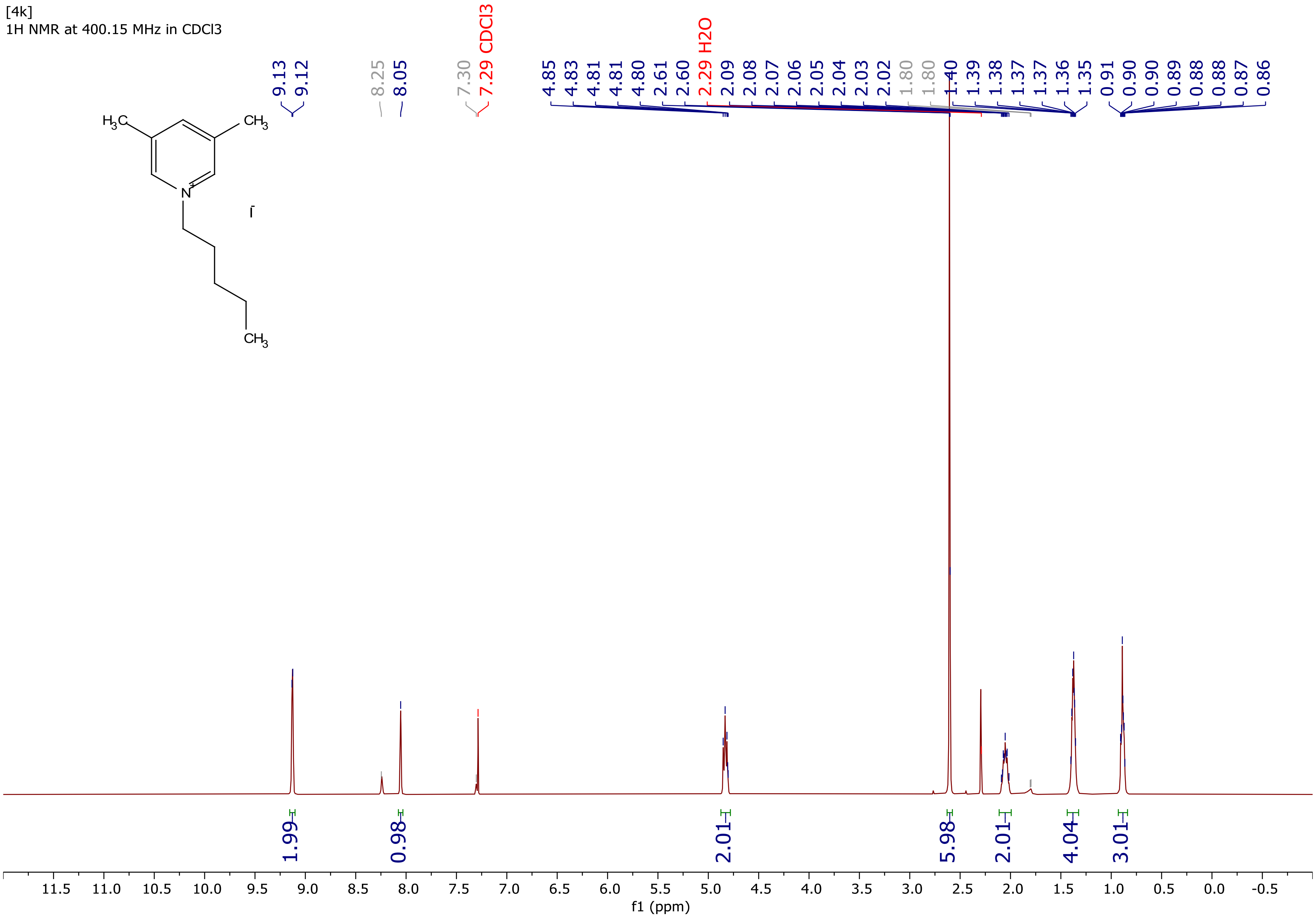

[4k]
13C NMR at 100.63 MHz in CDCl3

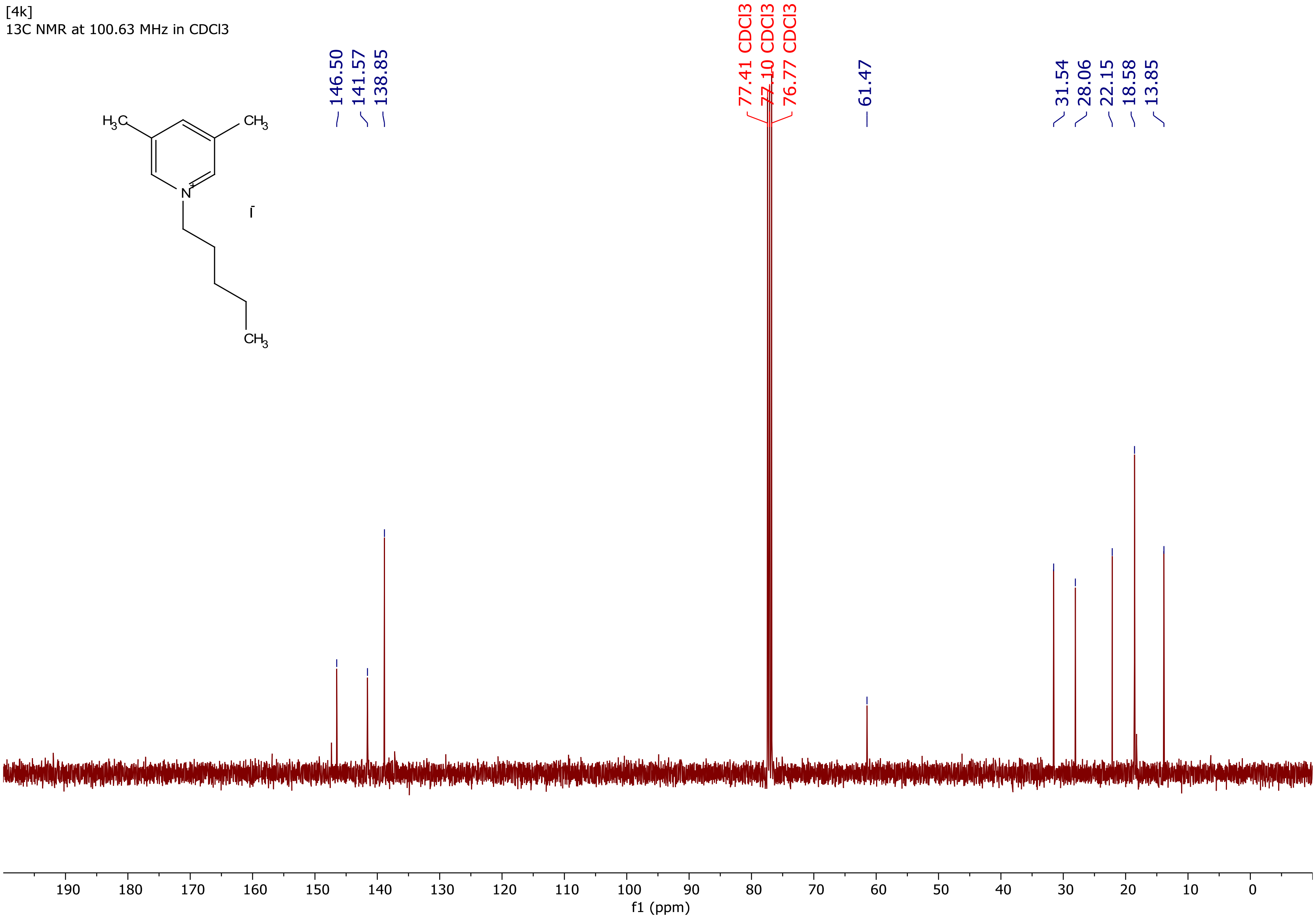

[4l]
1H NMR at 400.15 MHz in CDCl3

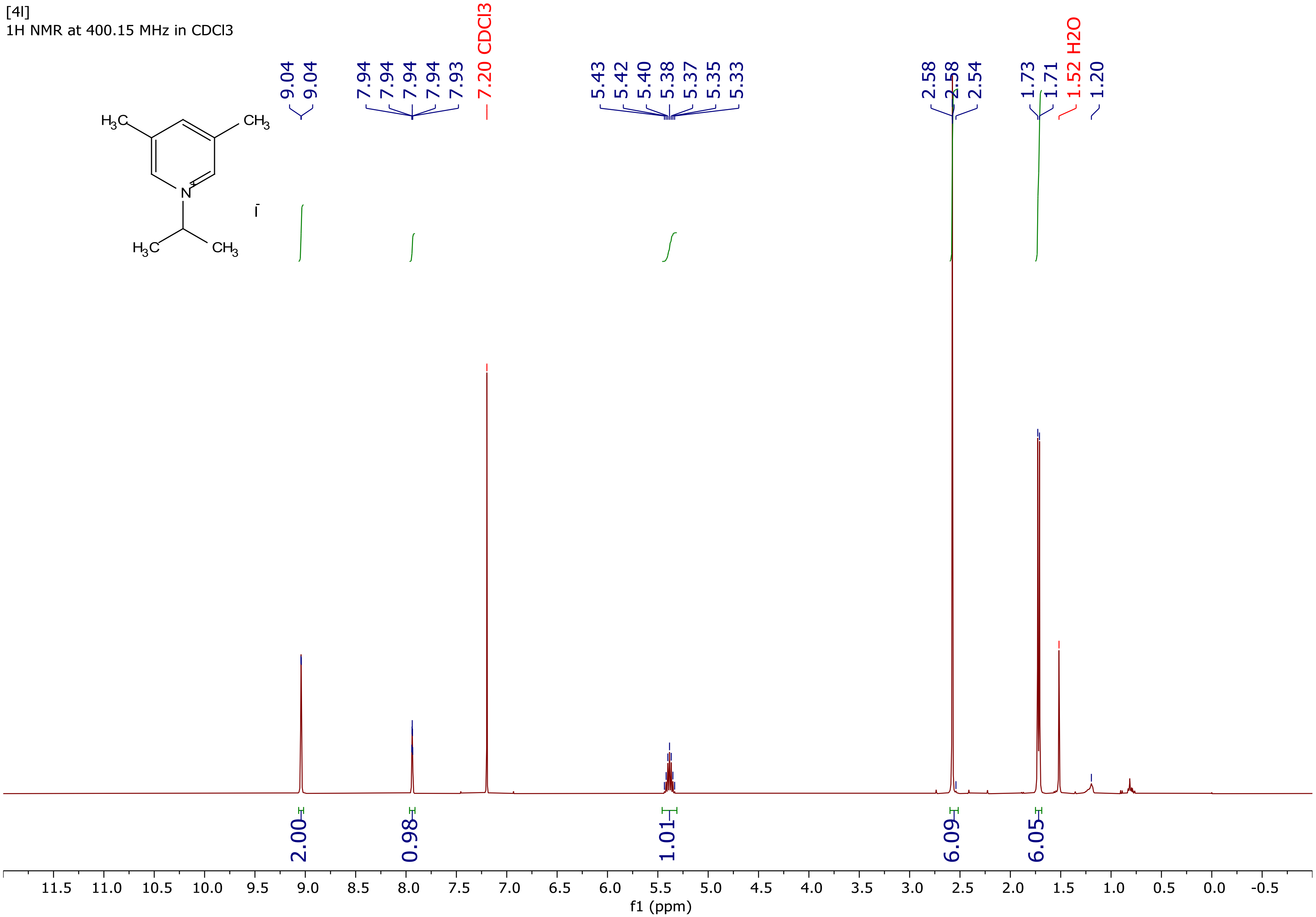

[4l]
13C NMR at 201.27 MHz in CDCl3

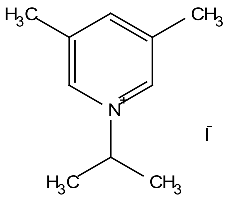

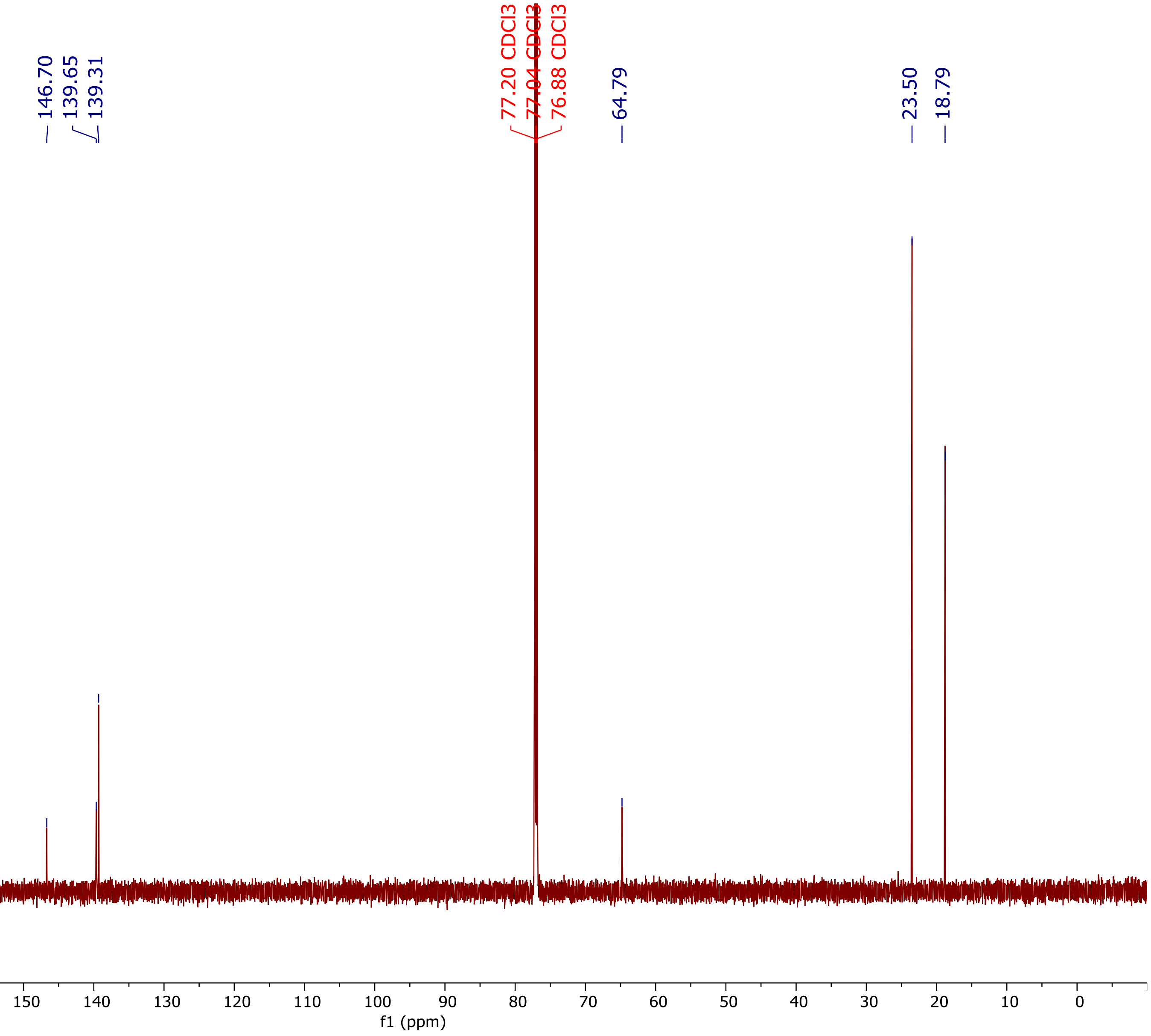

[4m]
1H NMR at 800.34 MHz in CDCl3

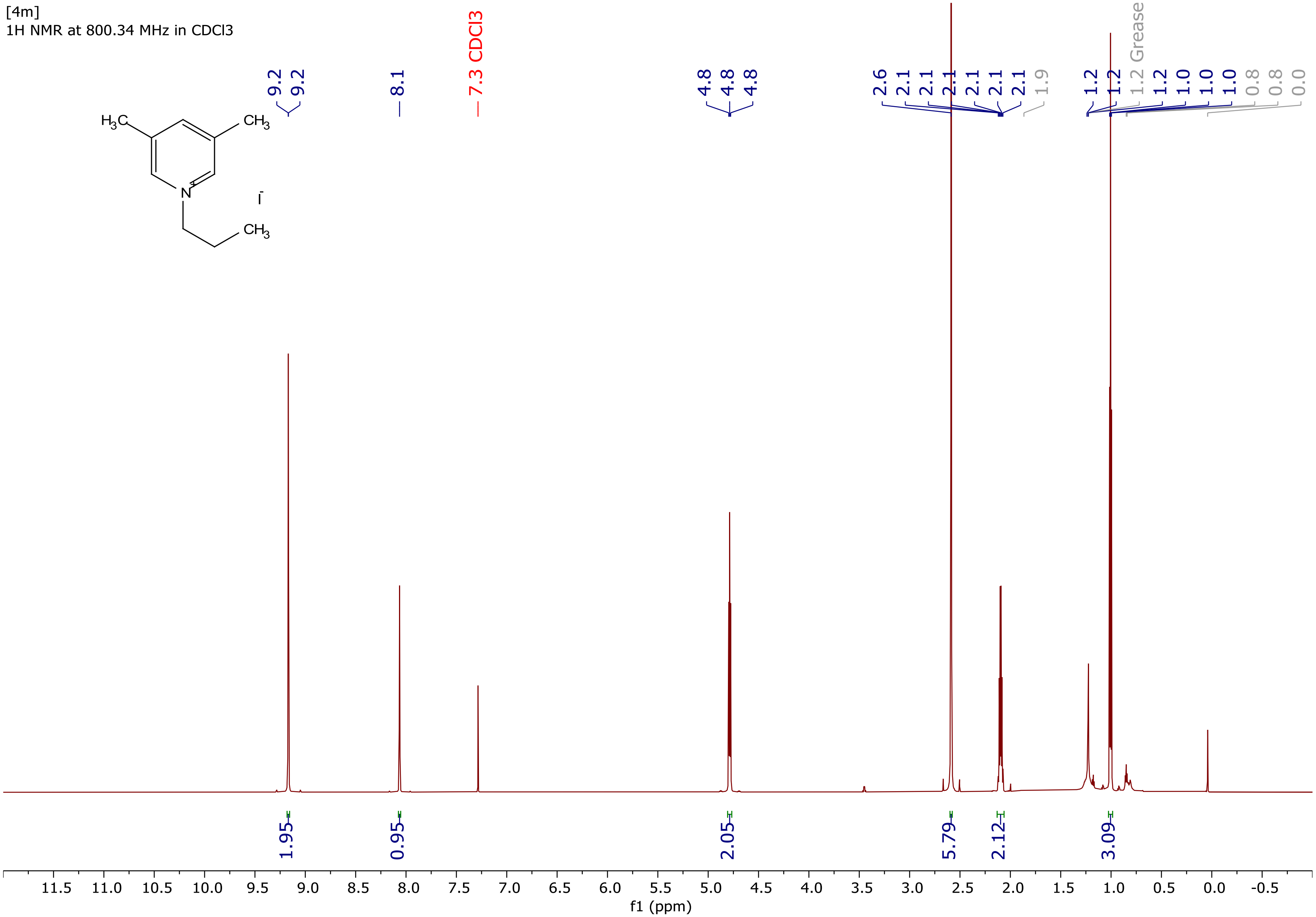

[4m]
13C NMR at 201.27 MHz in CDCl3

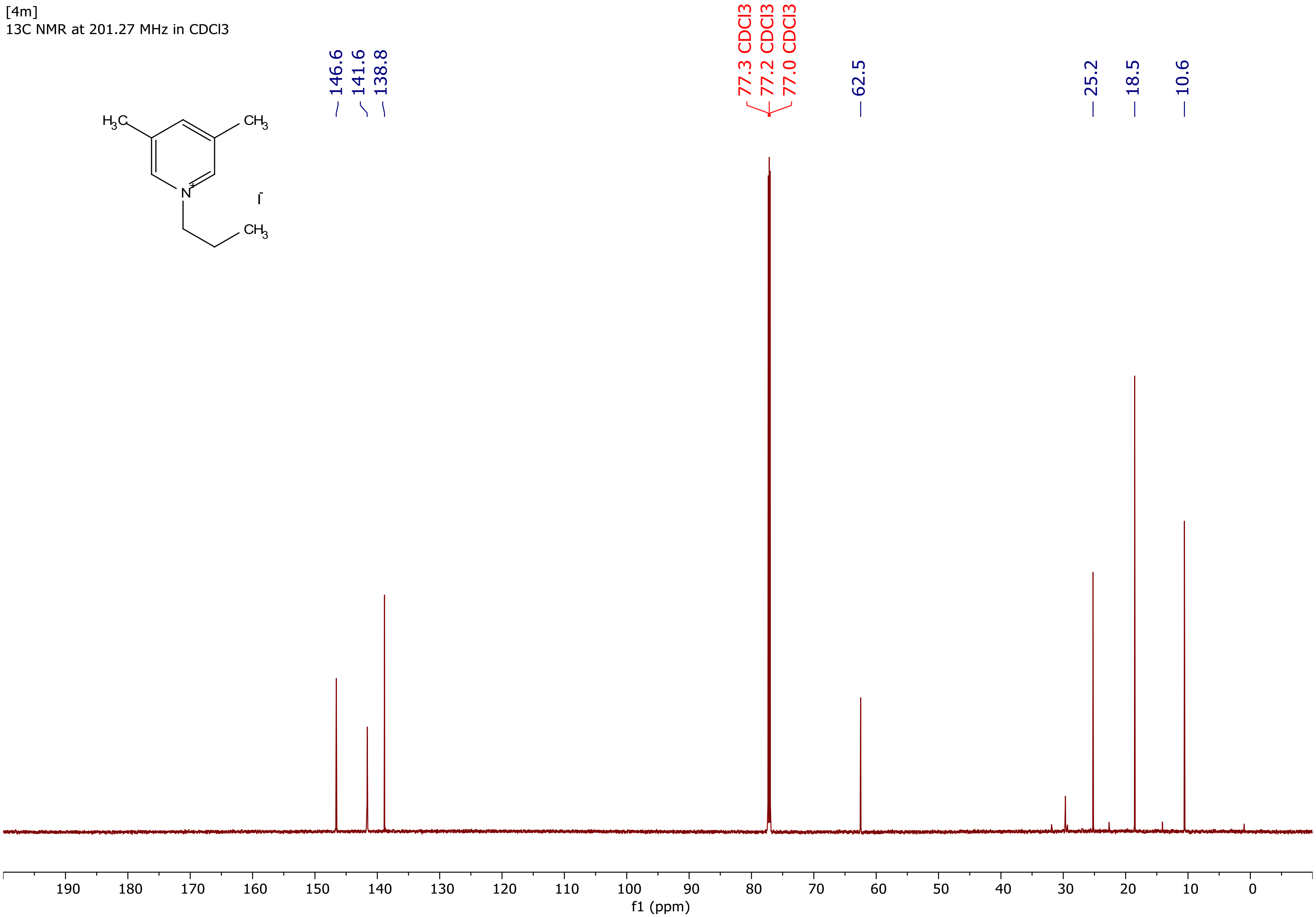

[4n]
1H NMR at 400.15 MHz in CDCl3

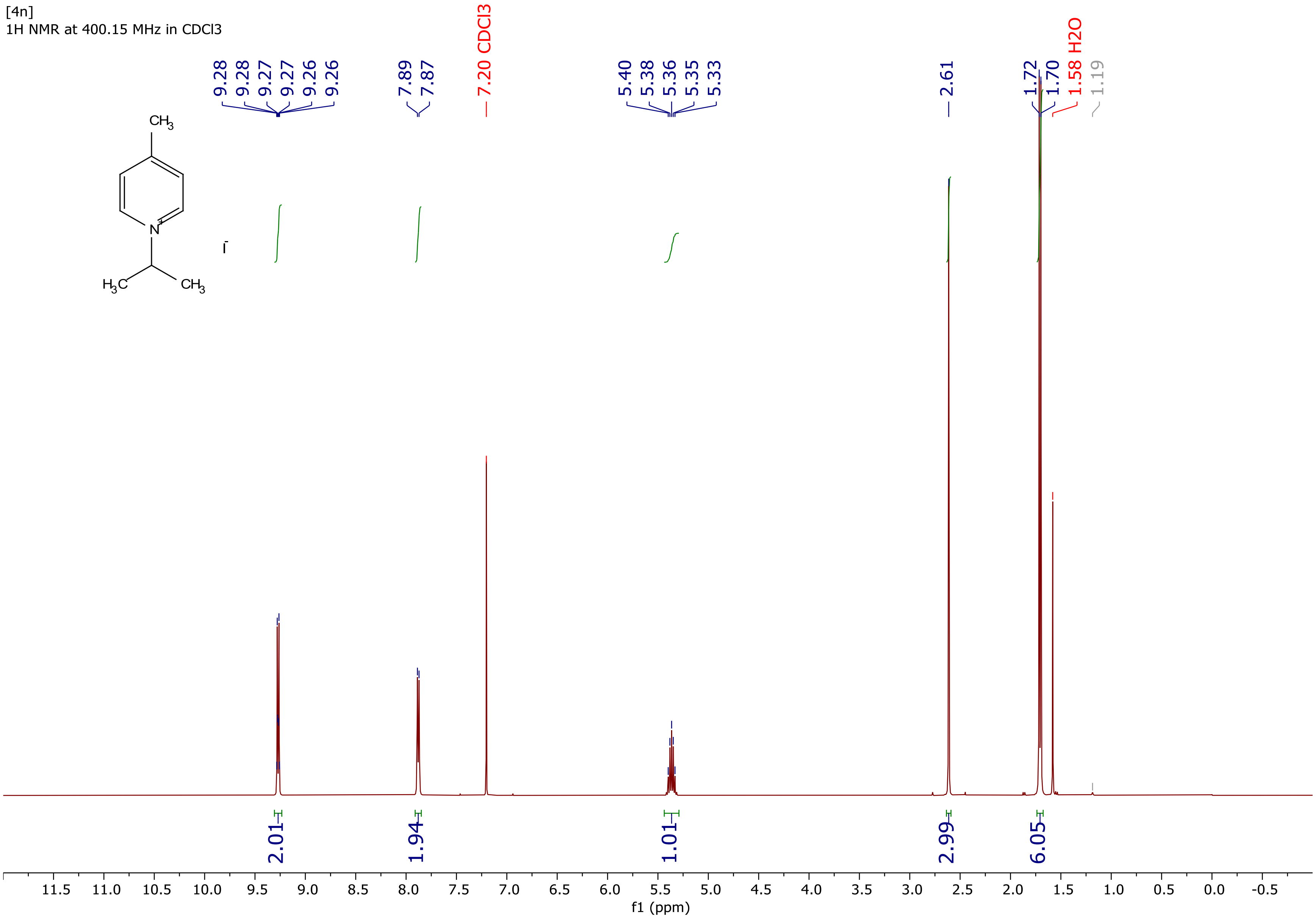

[4n]
13C NMR at 100.63 MHz in CDCl3

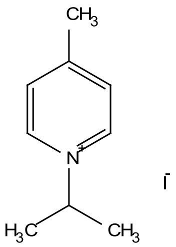

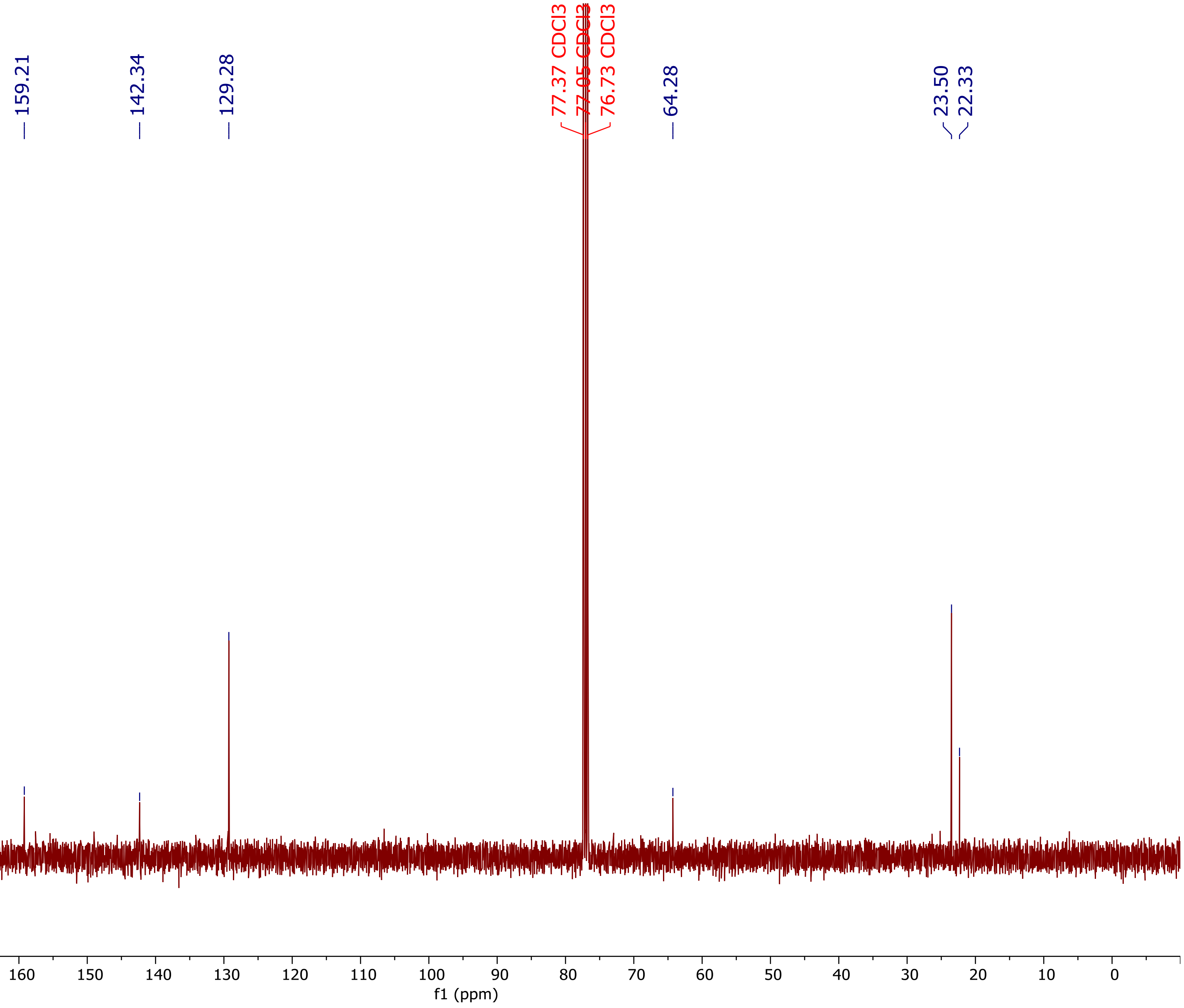

[4o]
1H NMR at 400.15 MHz in CDCl3

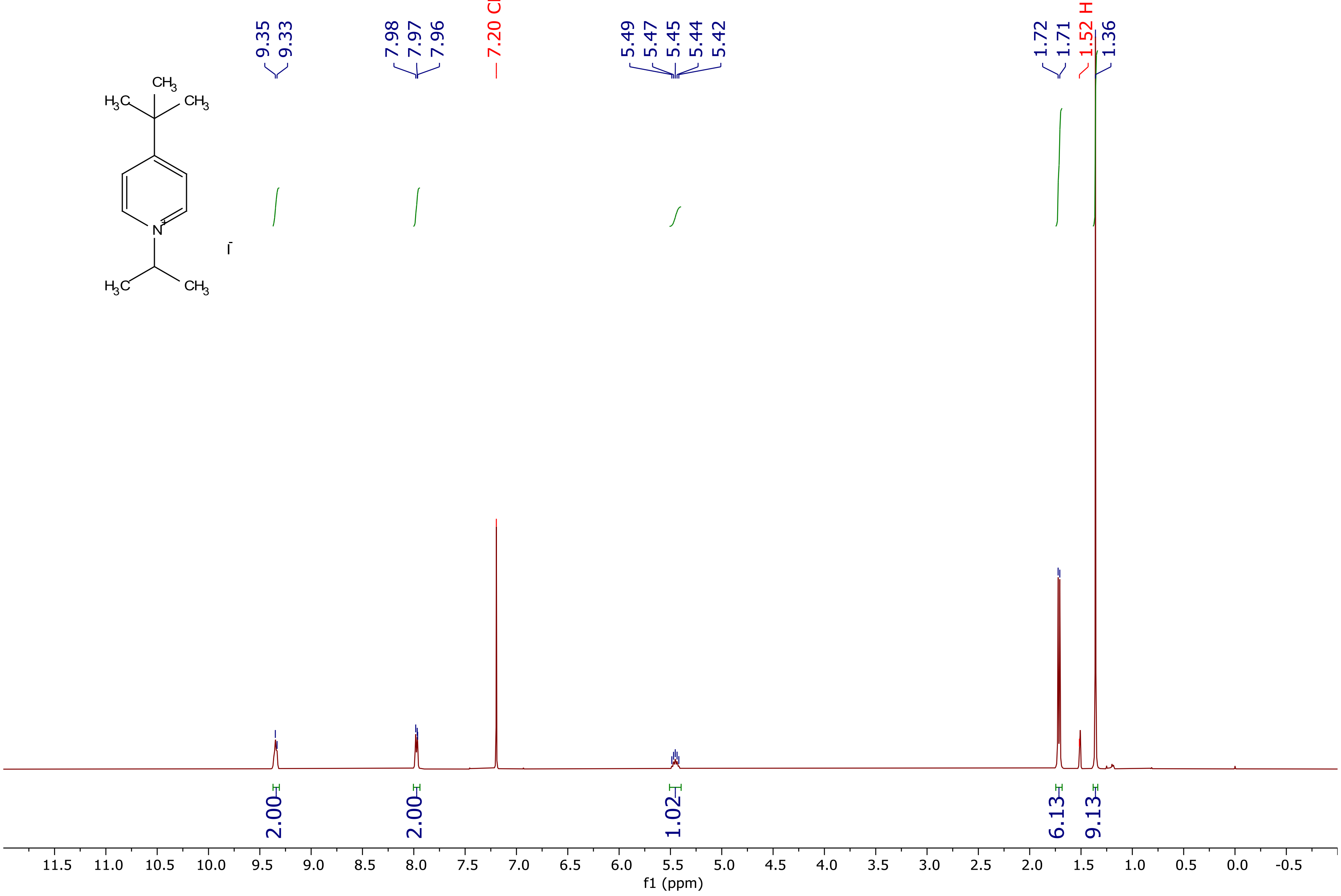

[4o]
13C NMR at 201.27 MHz in CDCl3

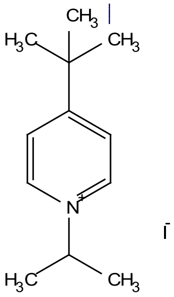

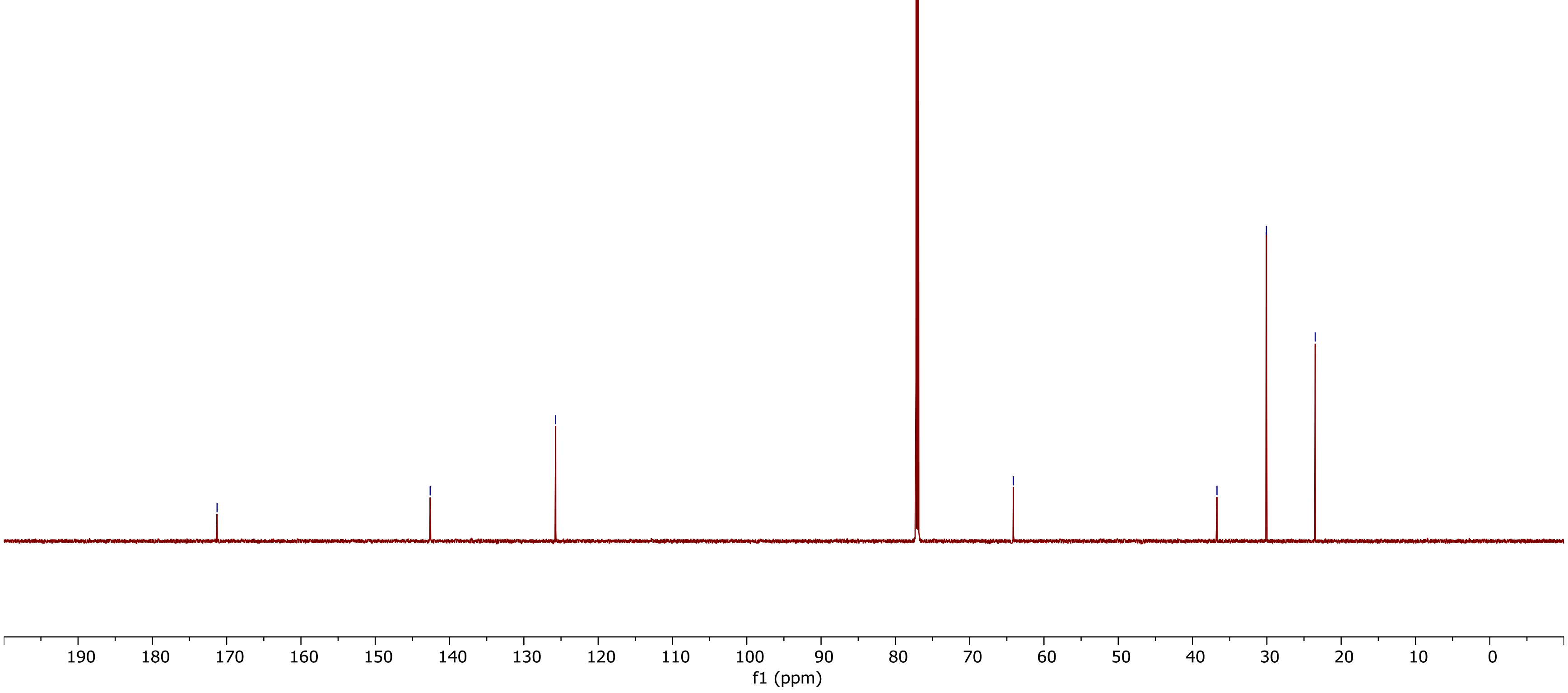

[4p]
1H NMR at 800.34 MHz in D2O

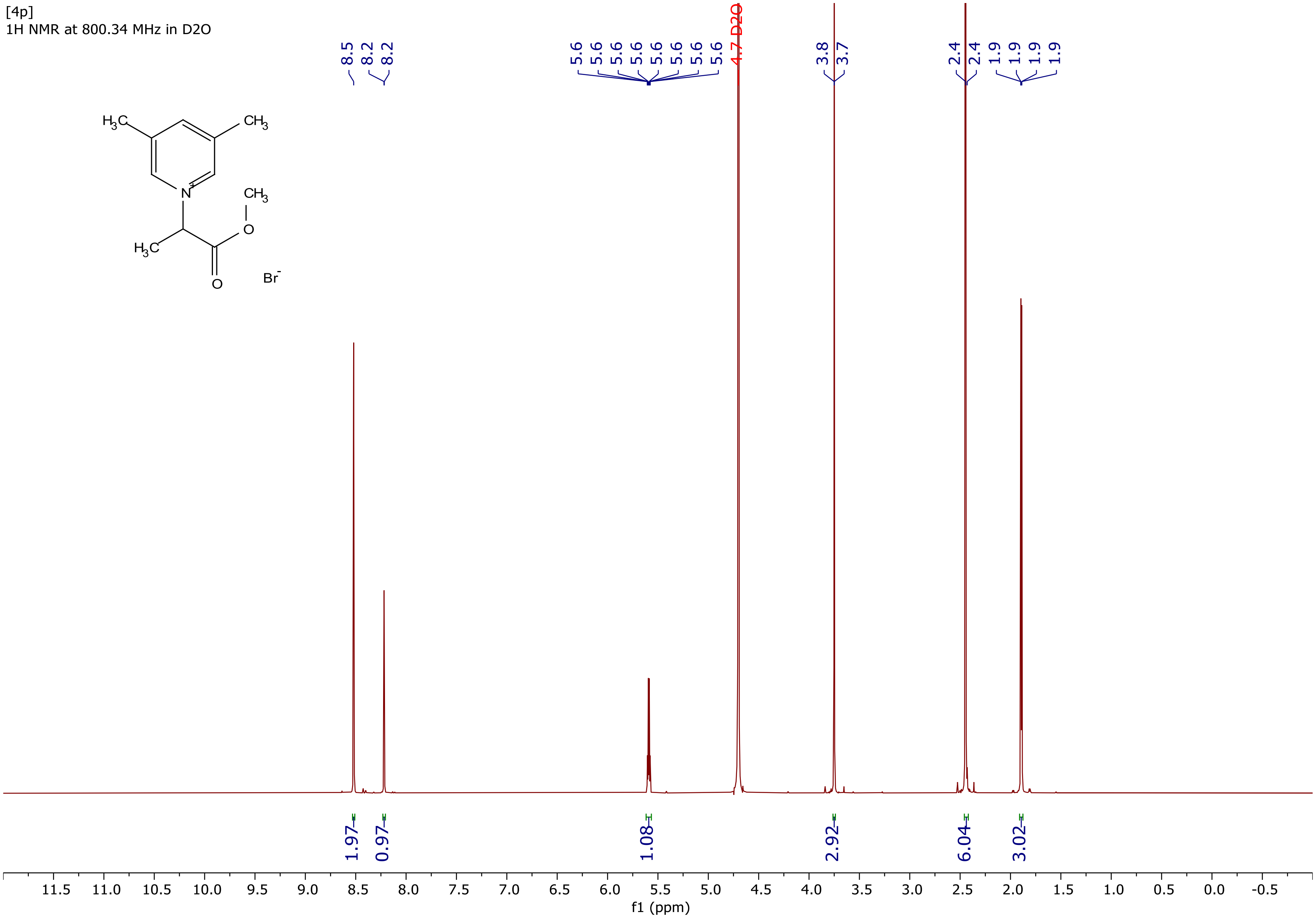

[4p]
13C NMR at 201.27 MHz in D2O

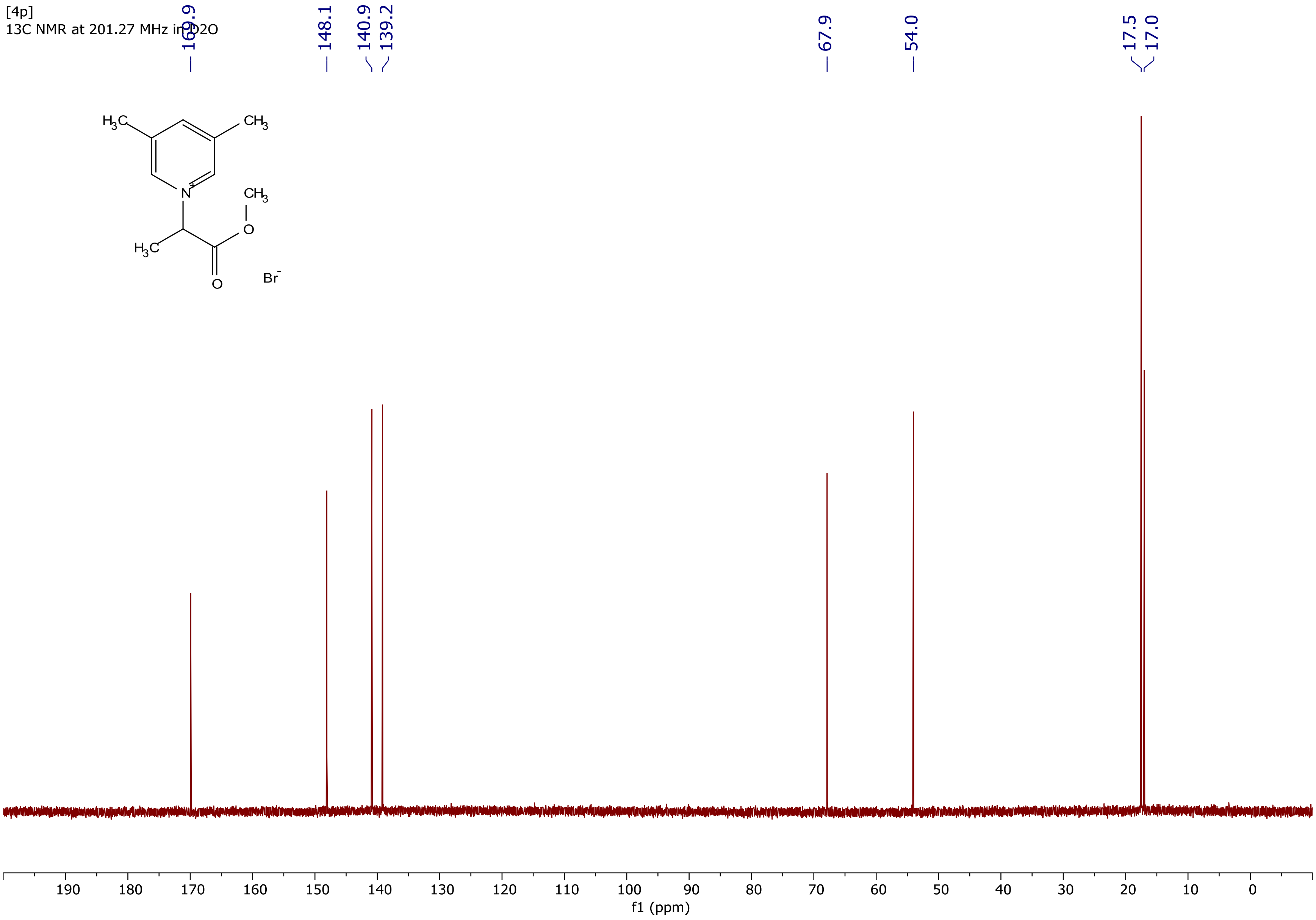

[4q]
1H NMR at 800.34 MHz in CDCl3

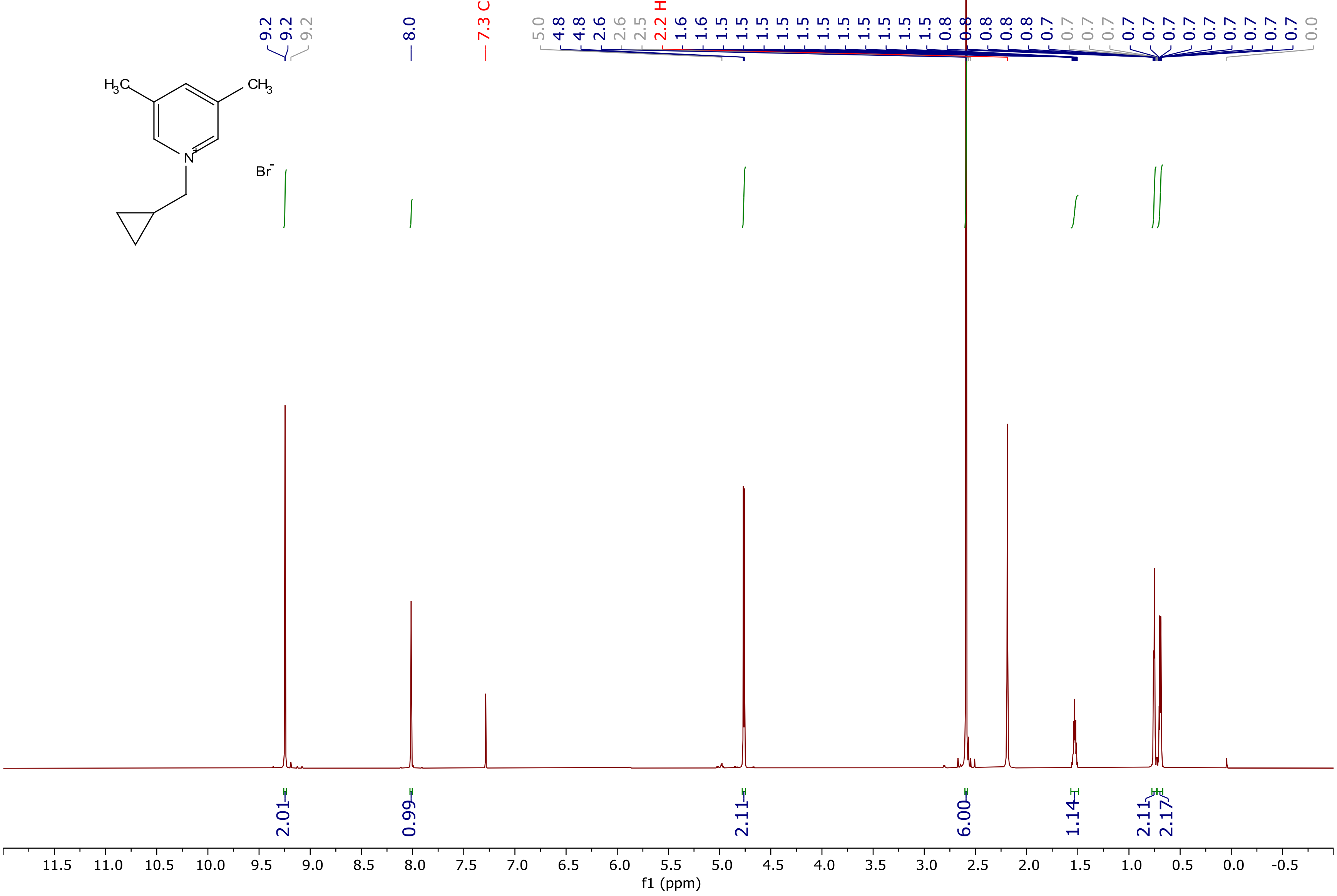

[4q]
13C NMR at 201.27 MHz in CDCl3

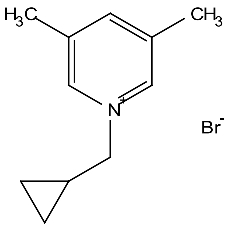

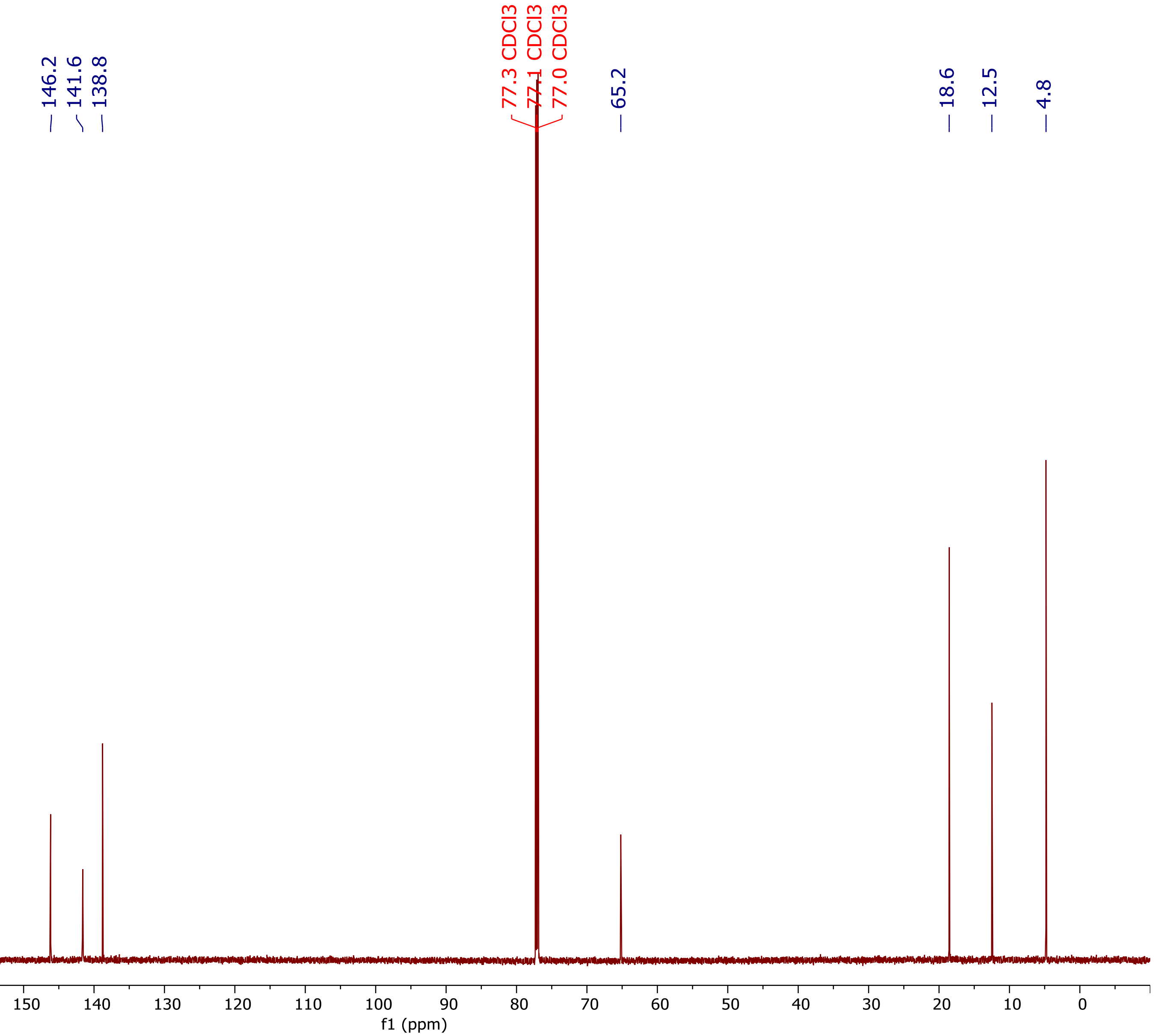

[4r]
1H NMR at 400.15 MHz in CDCl3

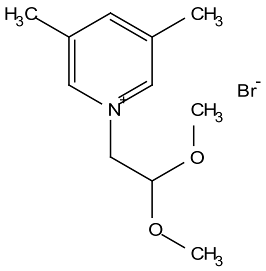

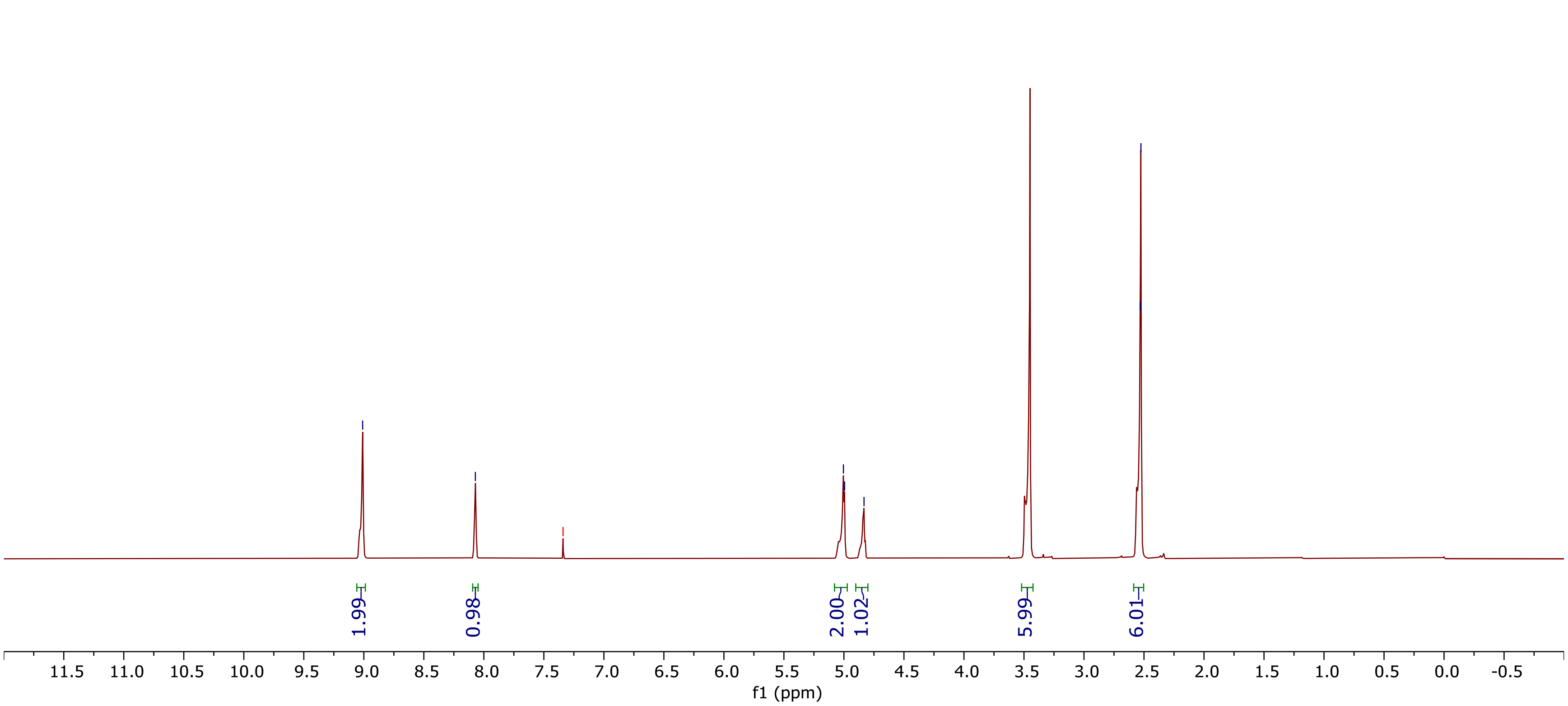

[4r]
13C NMR at 100.63 MHz in CDCl3

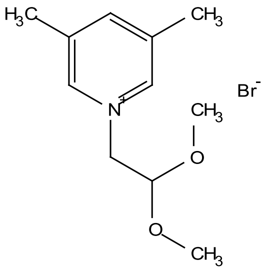

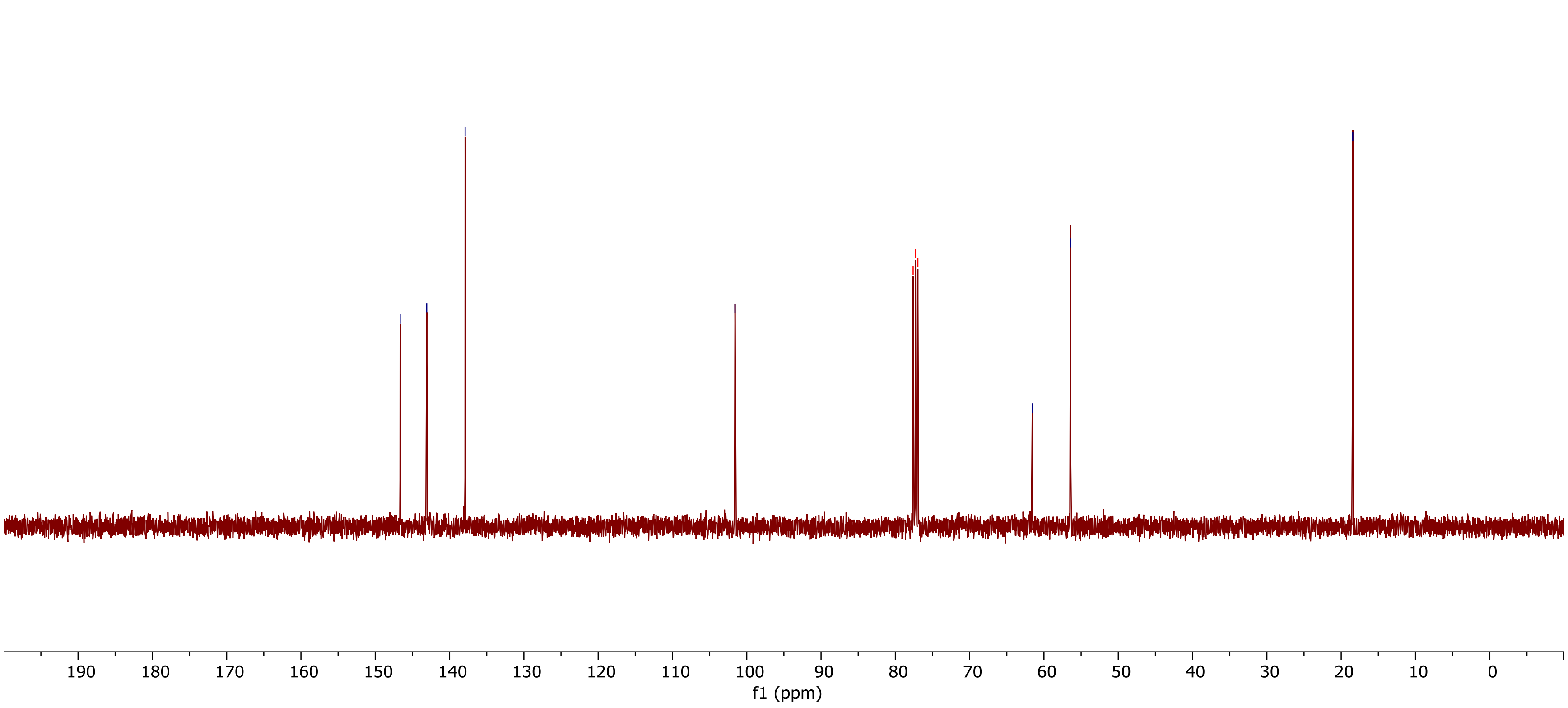

[4s]
1H NMR at 400.15 MHz in D2O

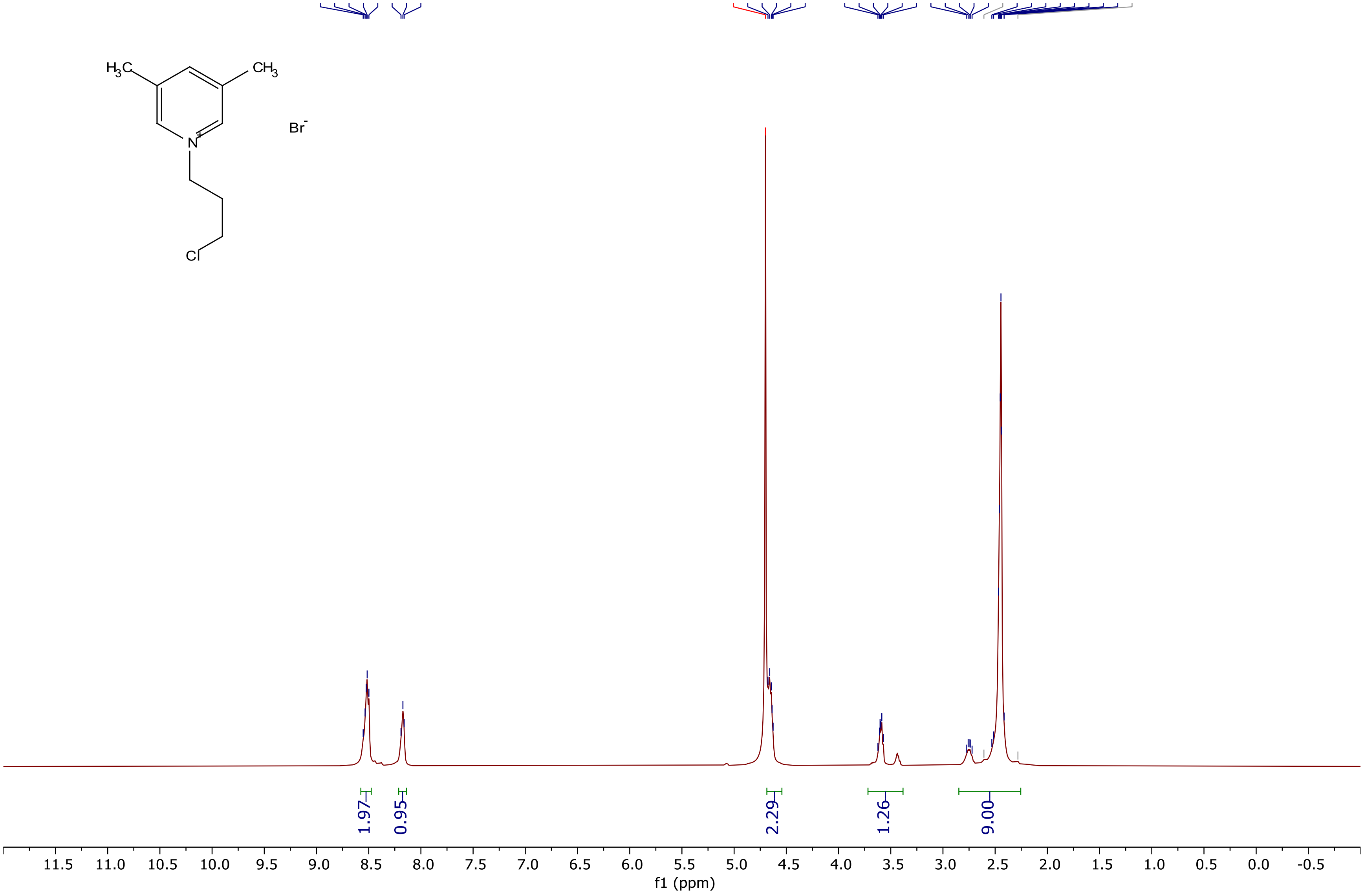

[4s]
13C NMR at 100.63 MHz in CDCl3

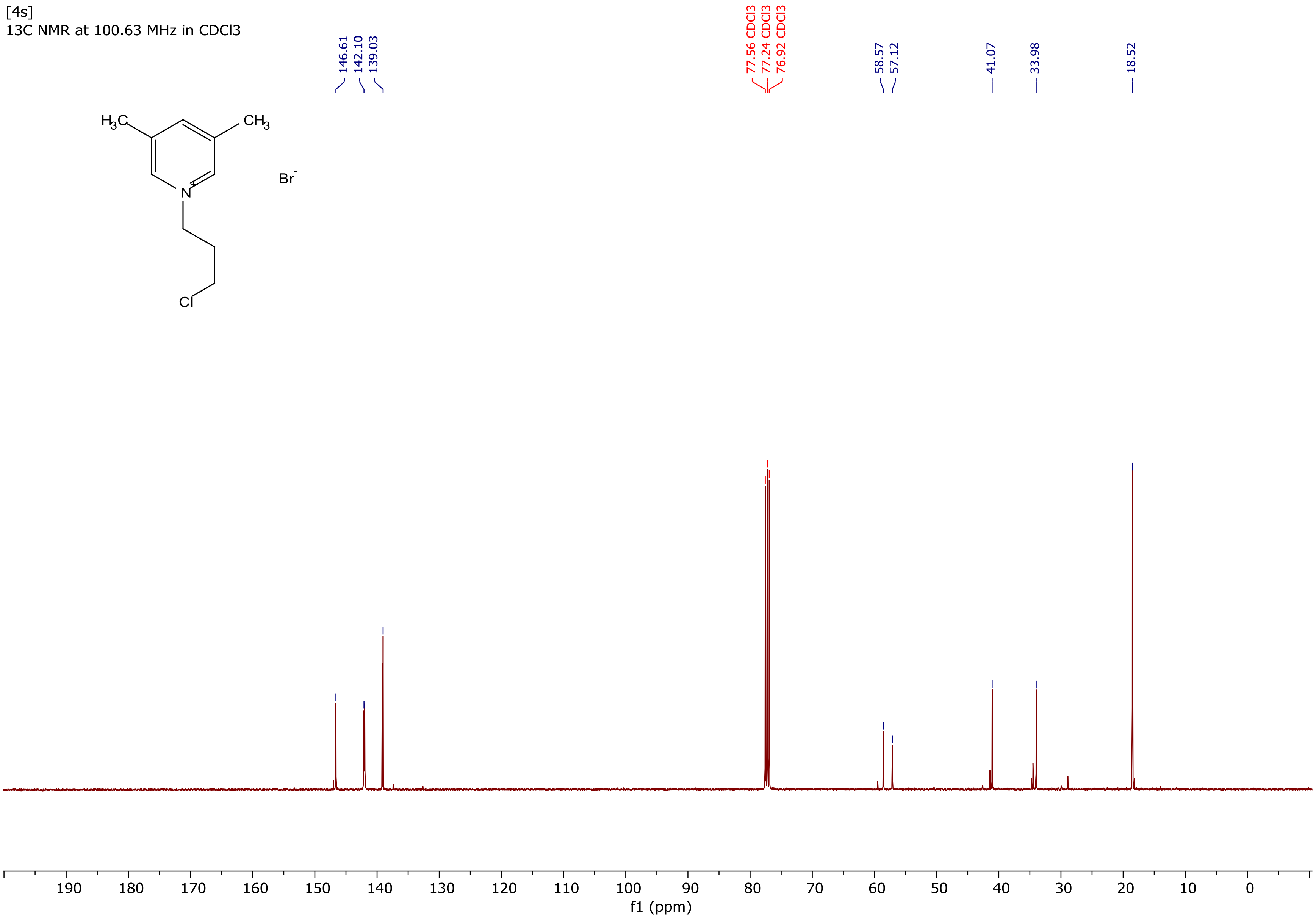

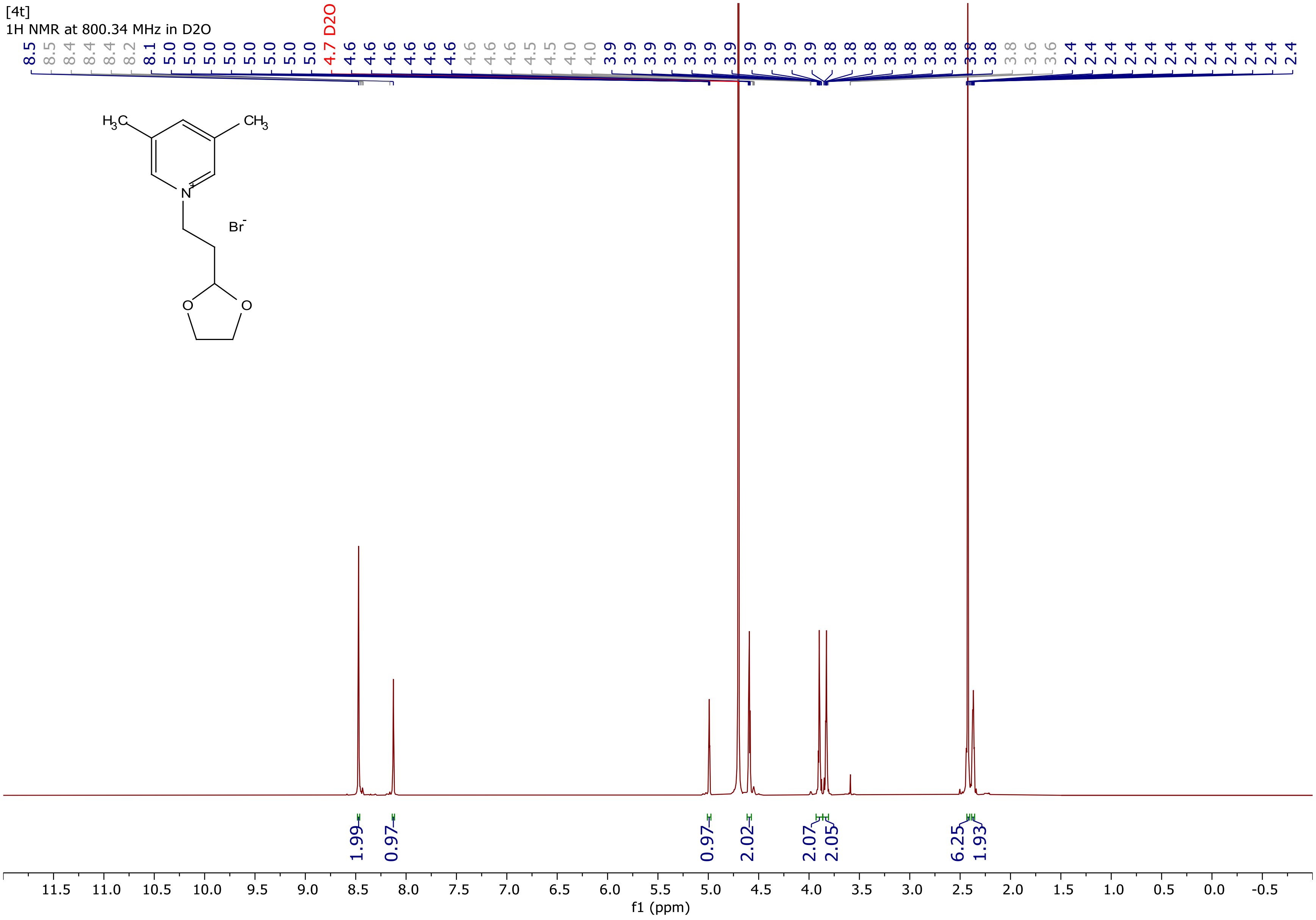
[4t]
1H NMR at 800.34 MHz in D2O
4.7 D2O
H3C
CH3
N+
Br-
O
O
1.99
0.97
0.97
2.02
2.07
2.05
6.25
1.93
f1 (ppm)

[4t]
13C NMR at 201.27 MHz in D2O

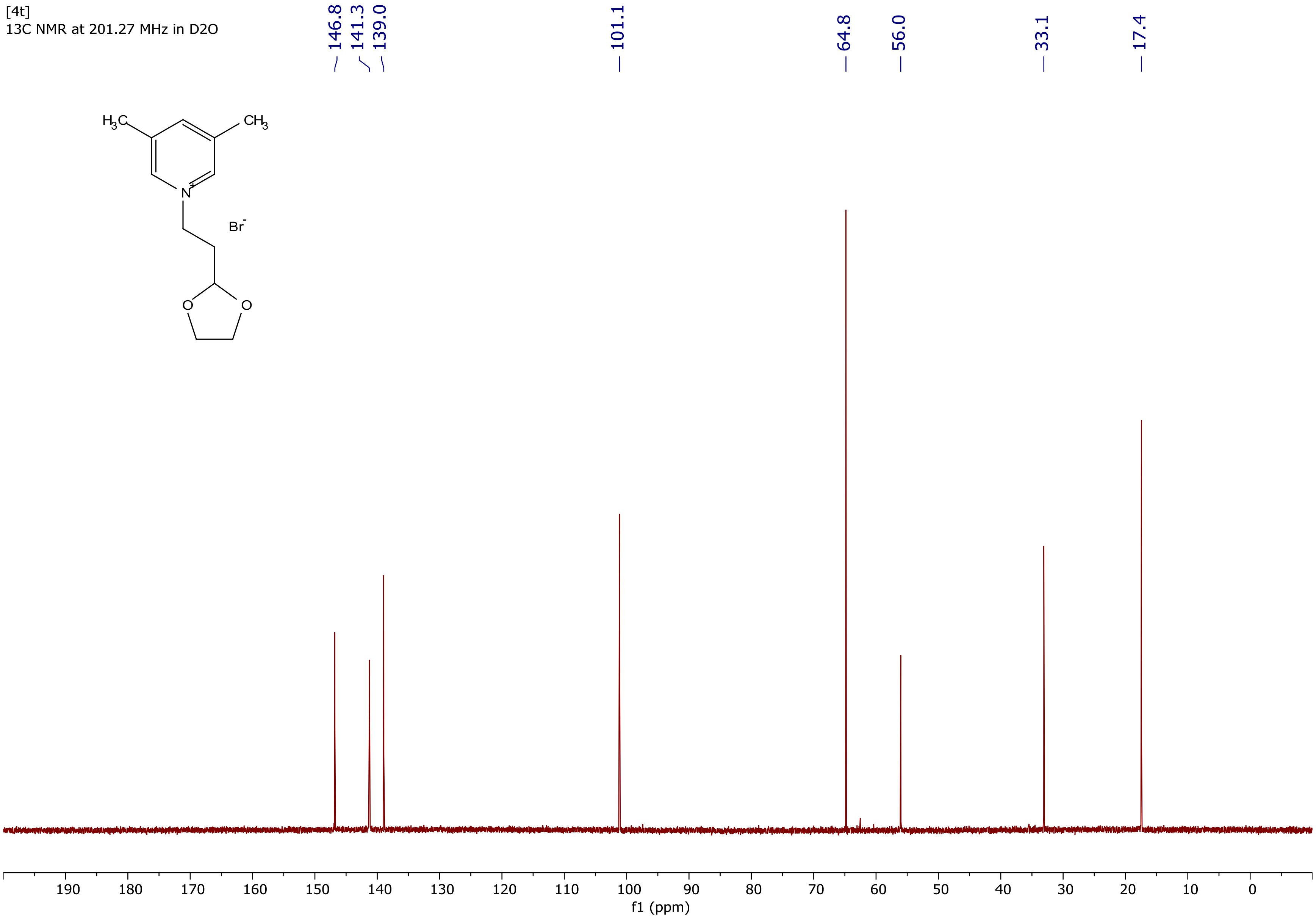

[4u]
1H NMR at 800.34 MHz in D2O

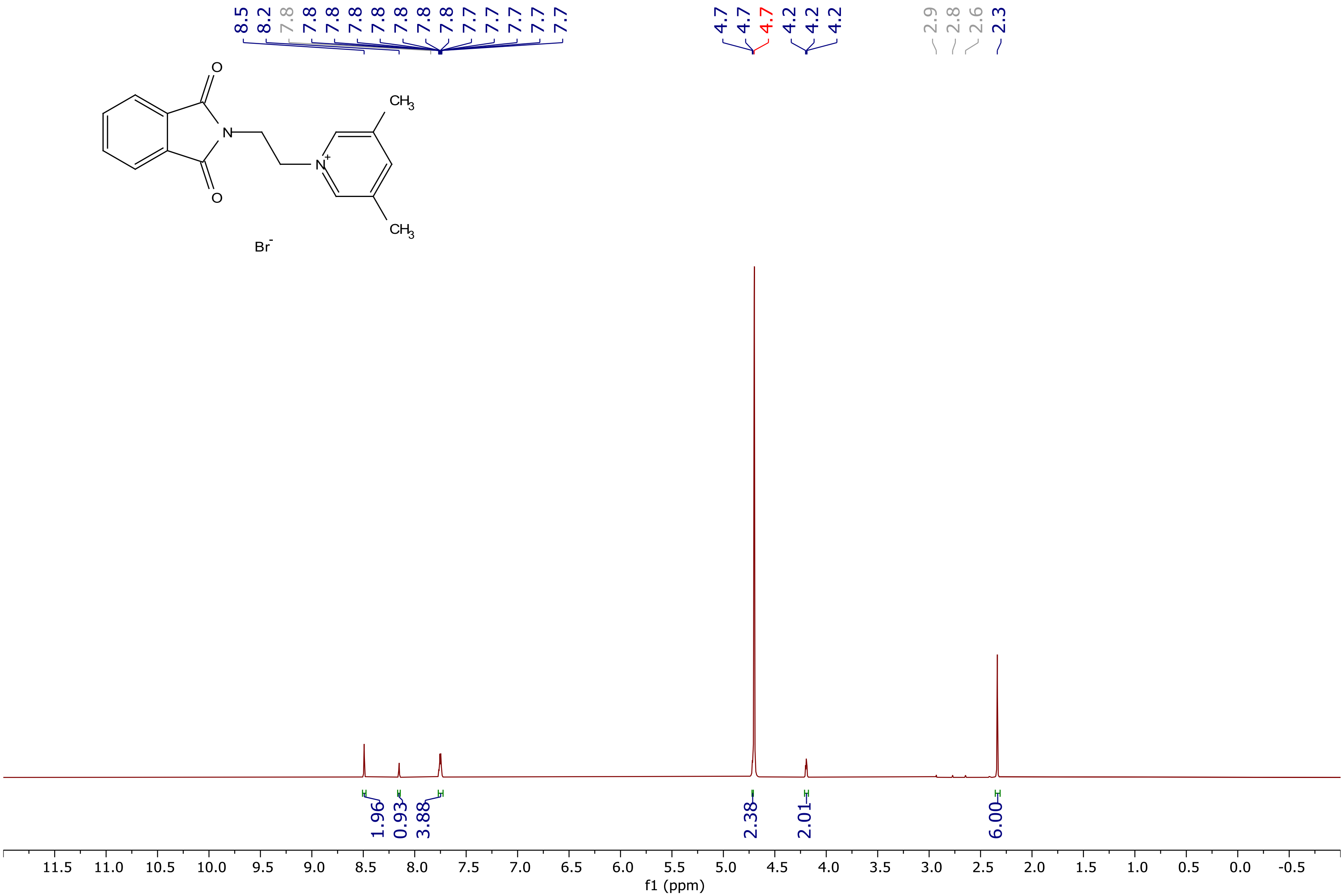

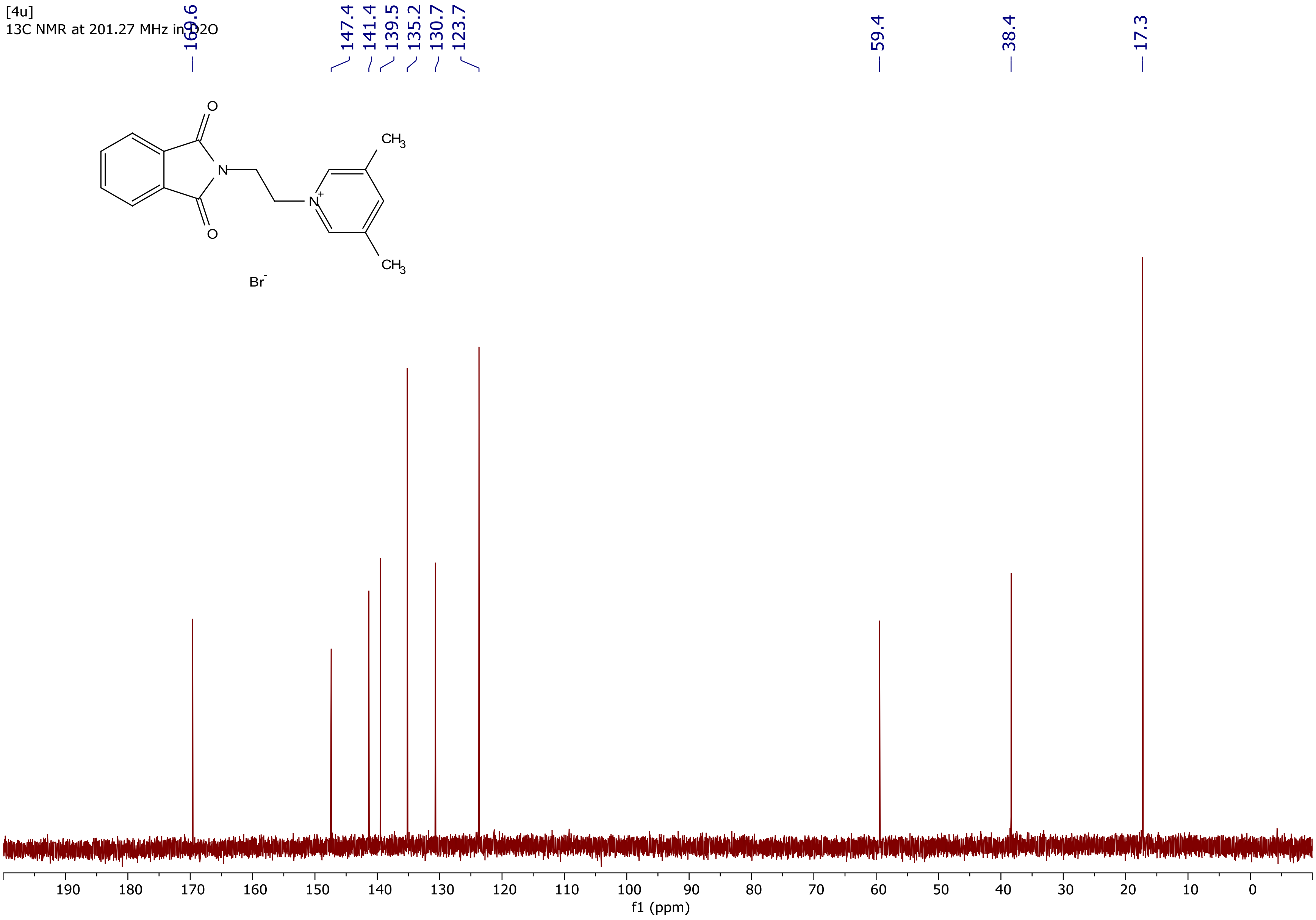
[4u]
13C NMR at 201.27 MHz in D2O
169.6
147.4
141.4
139.5
135.2
130.7
123.7
59.4
38.4
17.3
O
N
CH3
N+
O
CH3
Br-
190 180 170 160 150 140 130 120 110 100 90 80 70 60 50 40 30 20 10 0
f1 (ppm)

[4v]
1H NMR at 400.15 MHz in DMSO

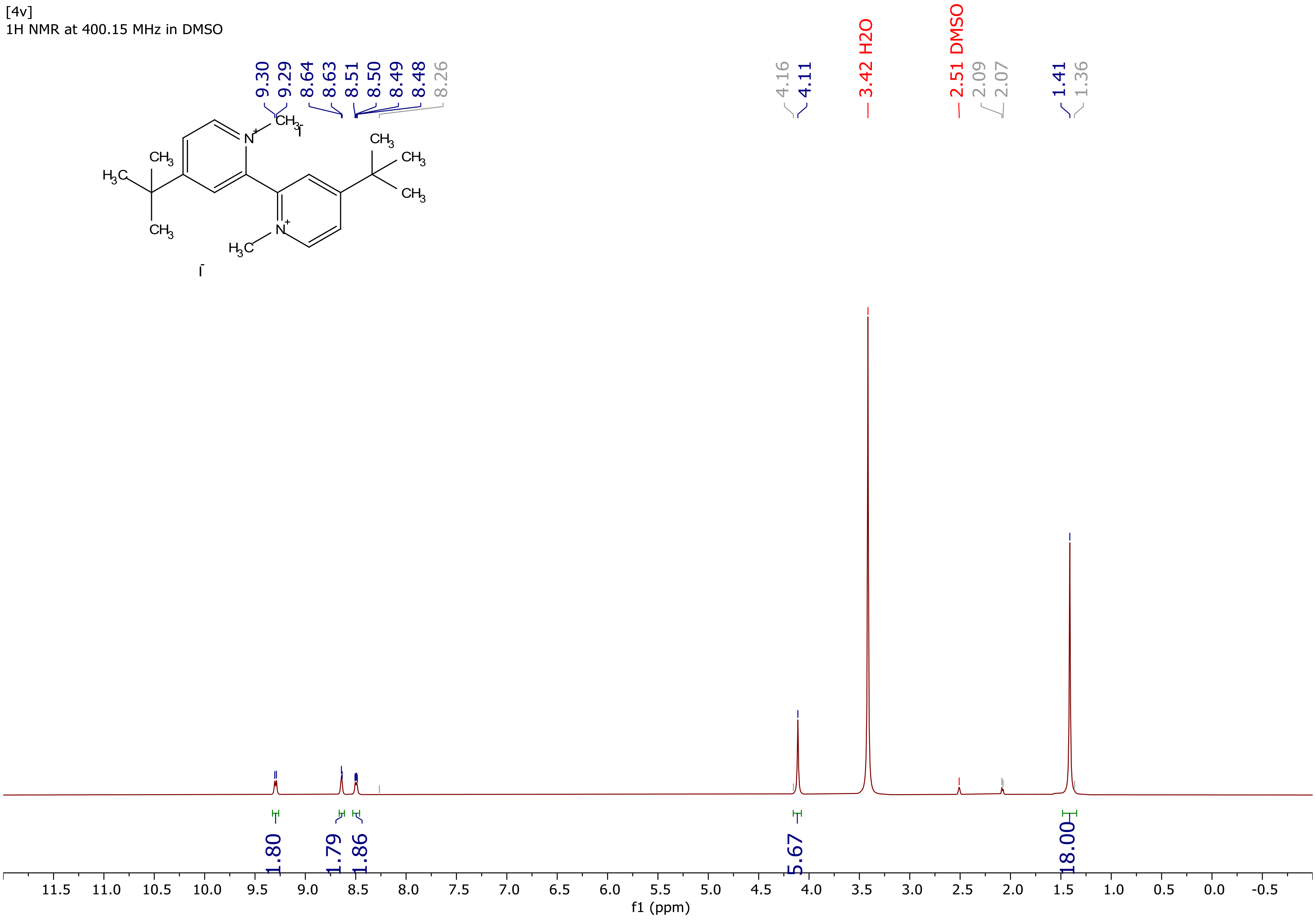

[4v]
13C NMR at 100.63 MHz in DMSO

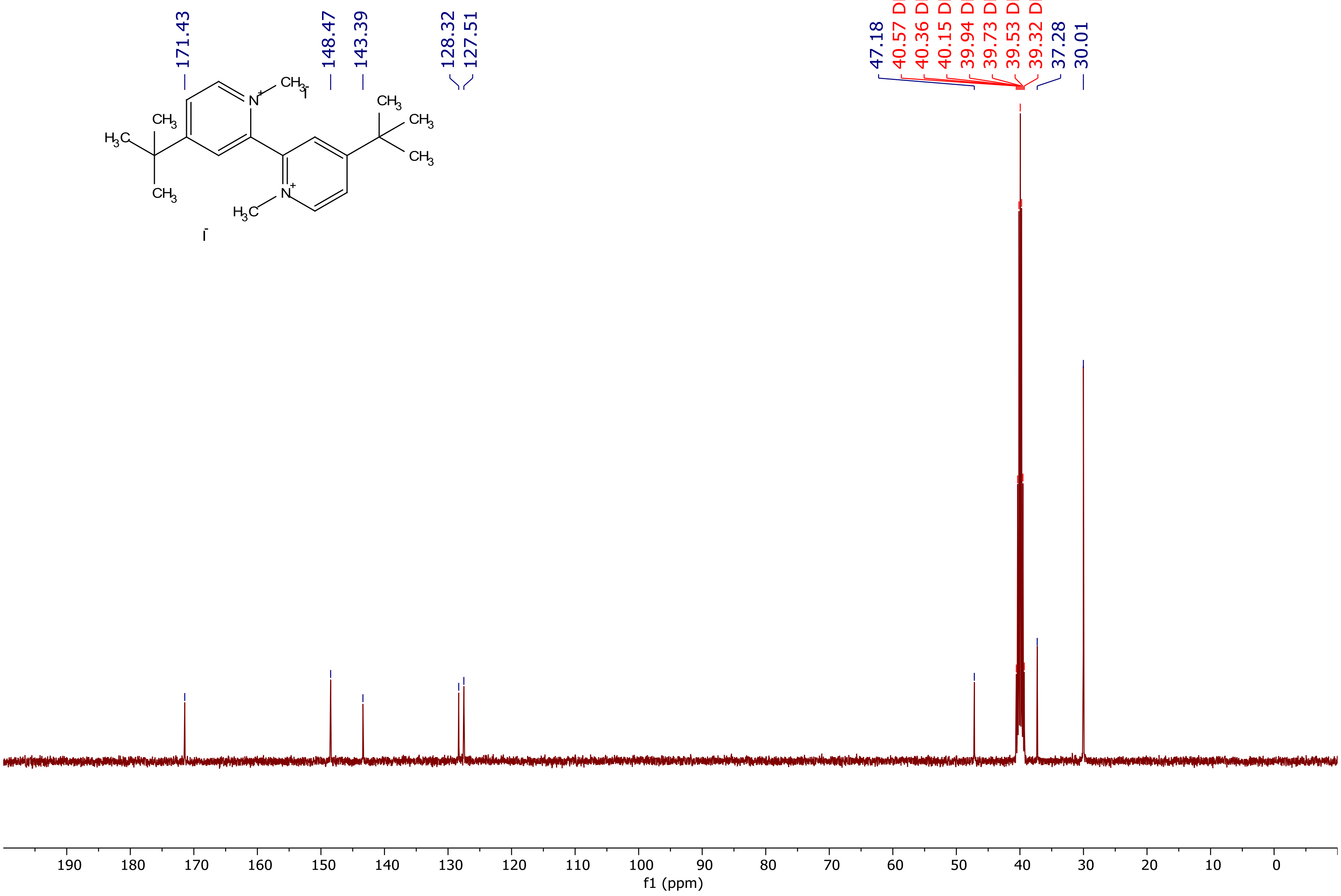

[4w]
1H NMR at 800.34 MHz in CDCl3

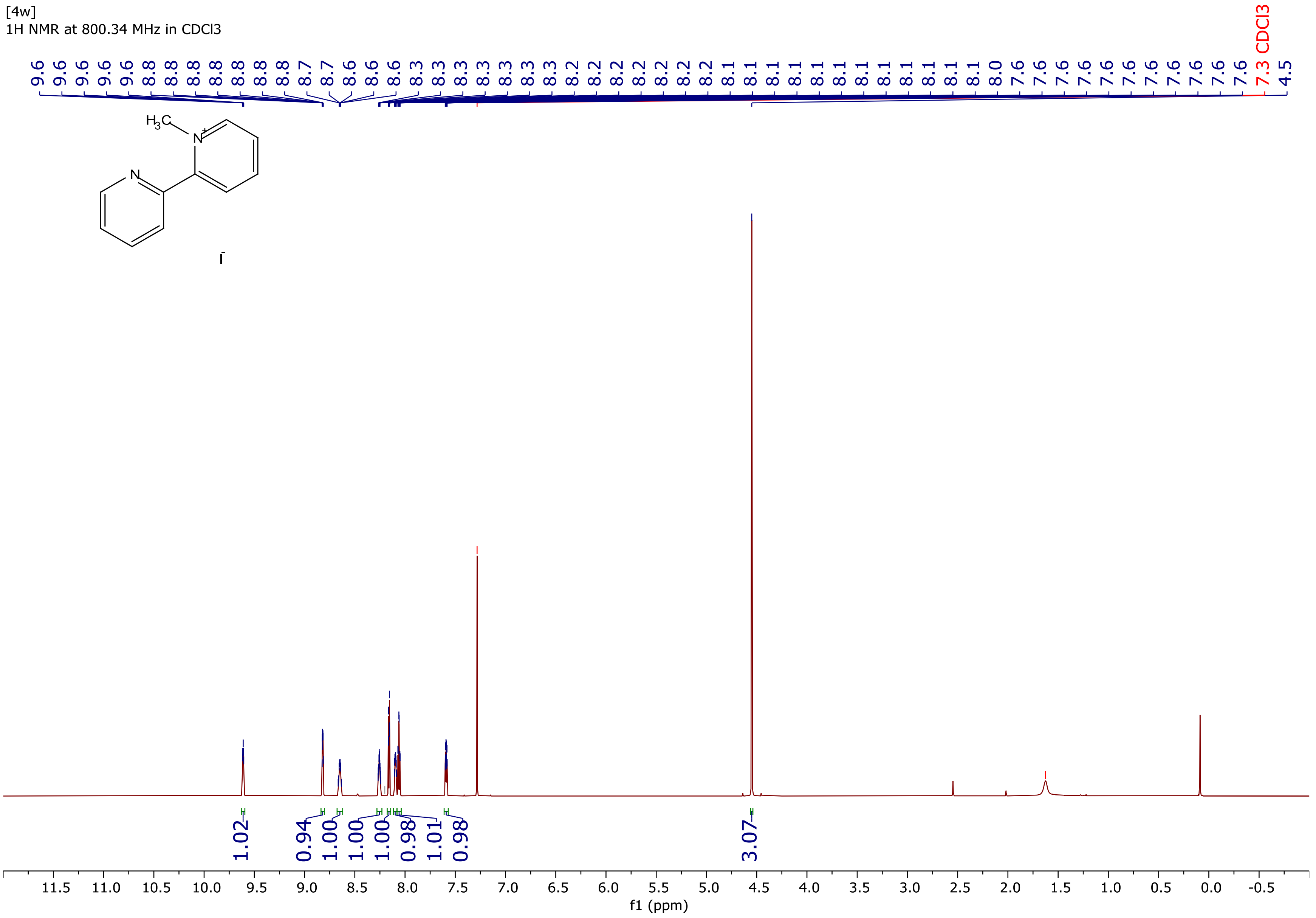

[4w]
13C NMR at 201.27 MHz in CDCl3

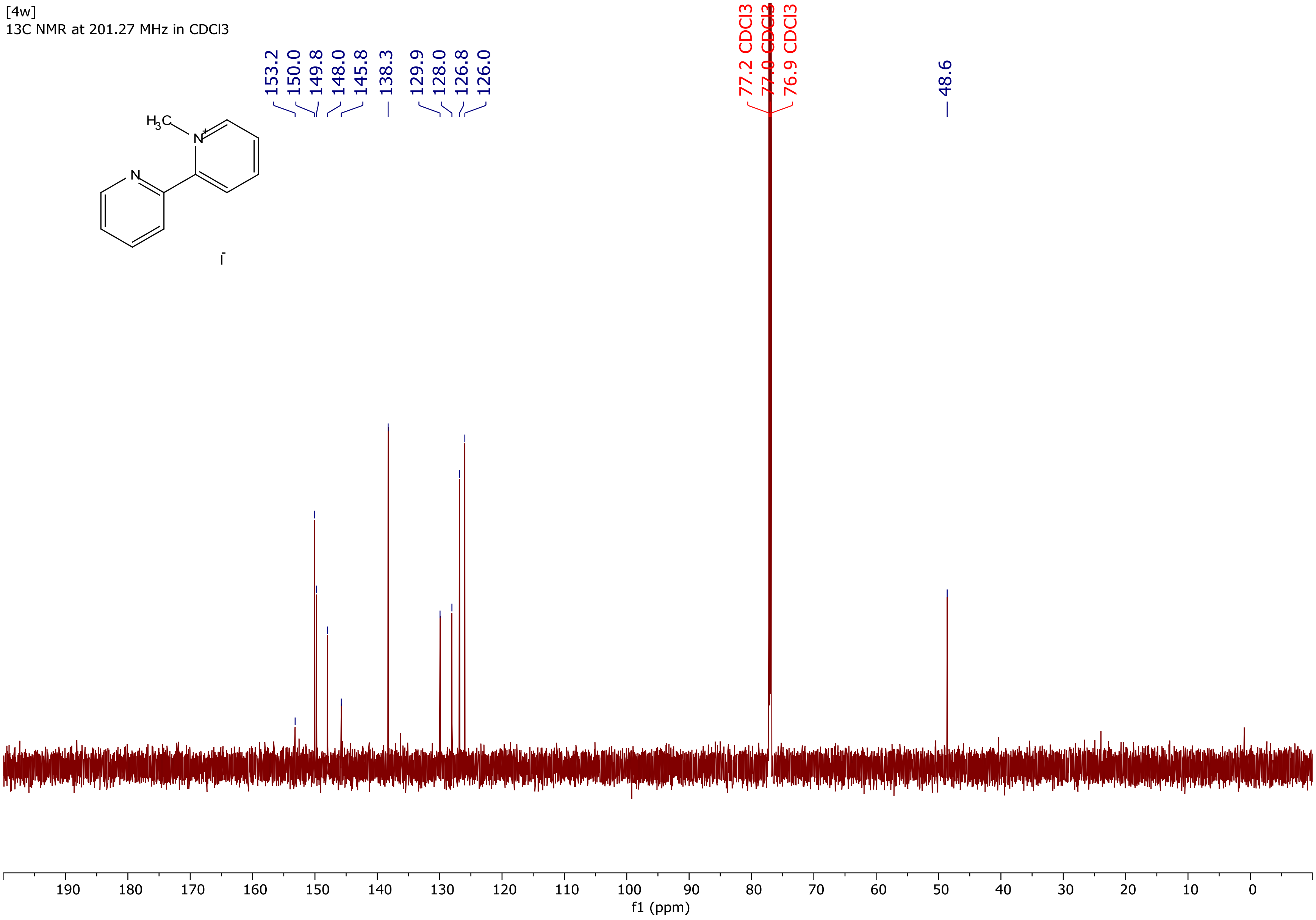

[4x]
1H NMR at 800.34 MHz in D2O

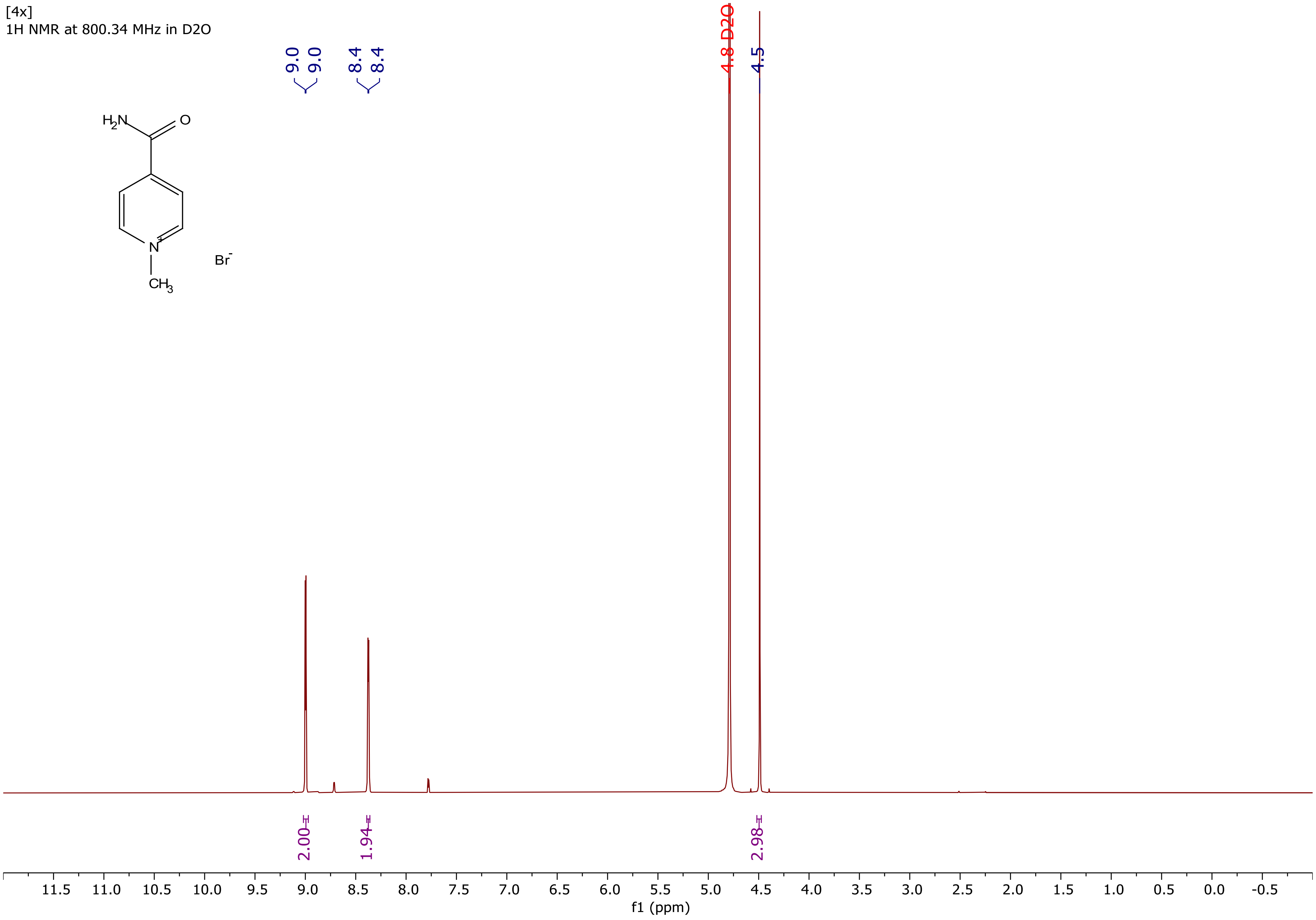

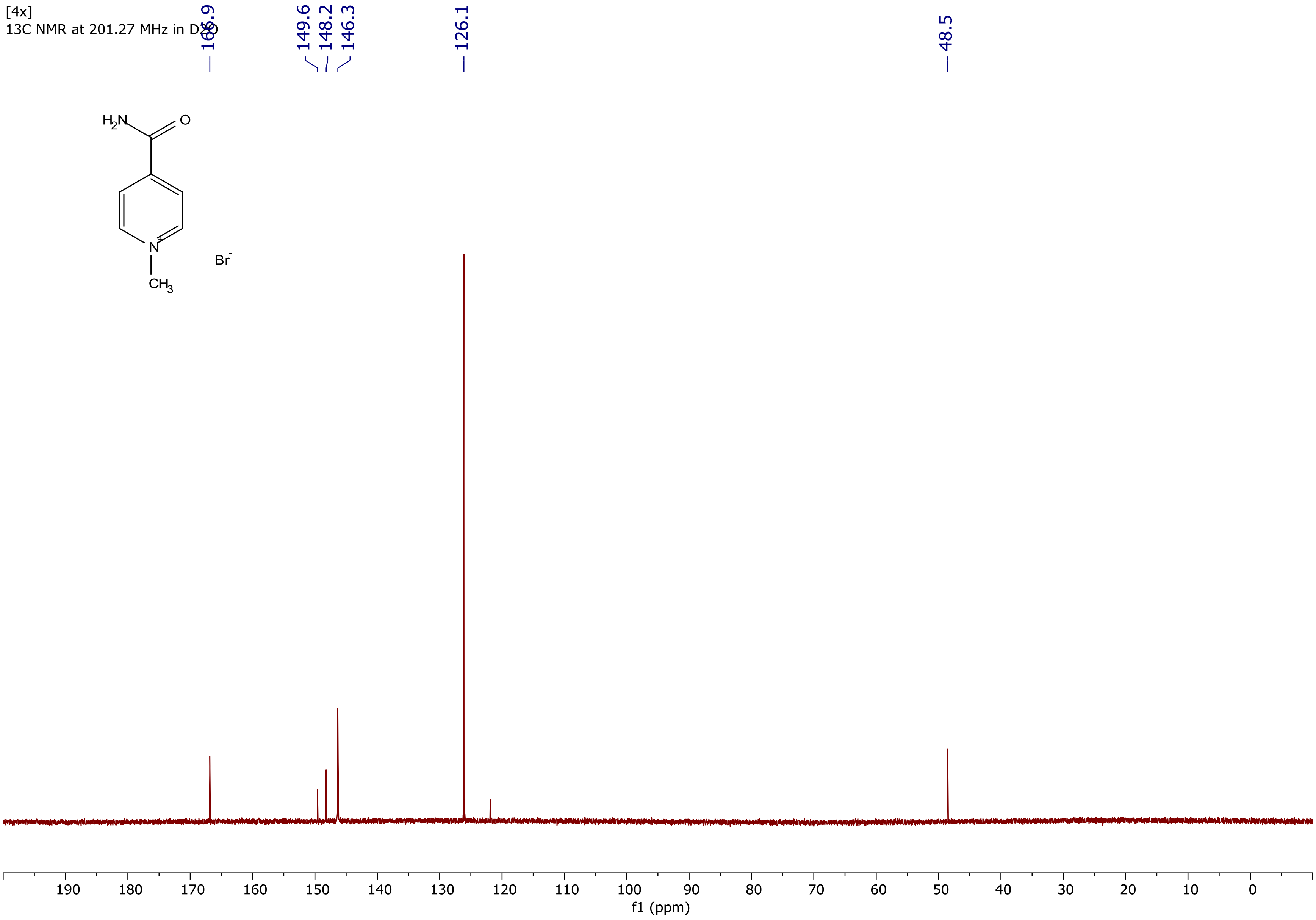
[4x]
13C NMR at 201.27 MHz in D2O
166.9
149.6
148.2
146.3
126.1
48.5
H2N
O
N+
CH3
Br-
190 180 170 160 150 140 130 120 110 100 90 80 70 60 50 40 30 20 10 0
f1 (ppm)

[4y]
1H NMR at 400.15 MHz in CDCl3

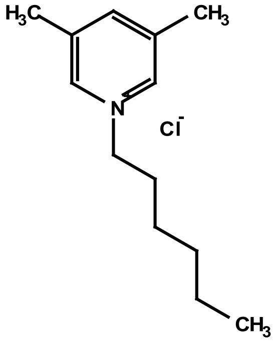

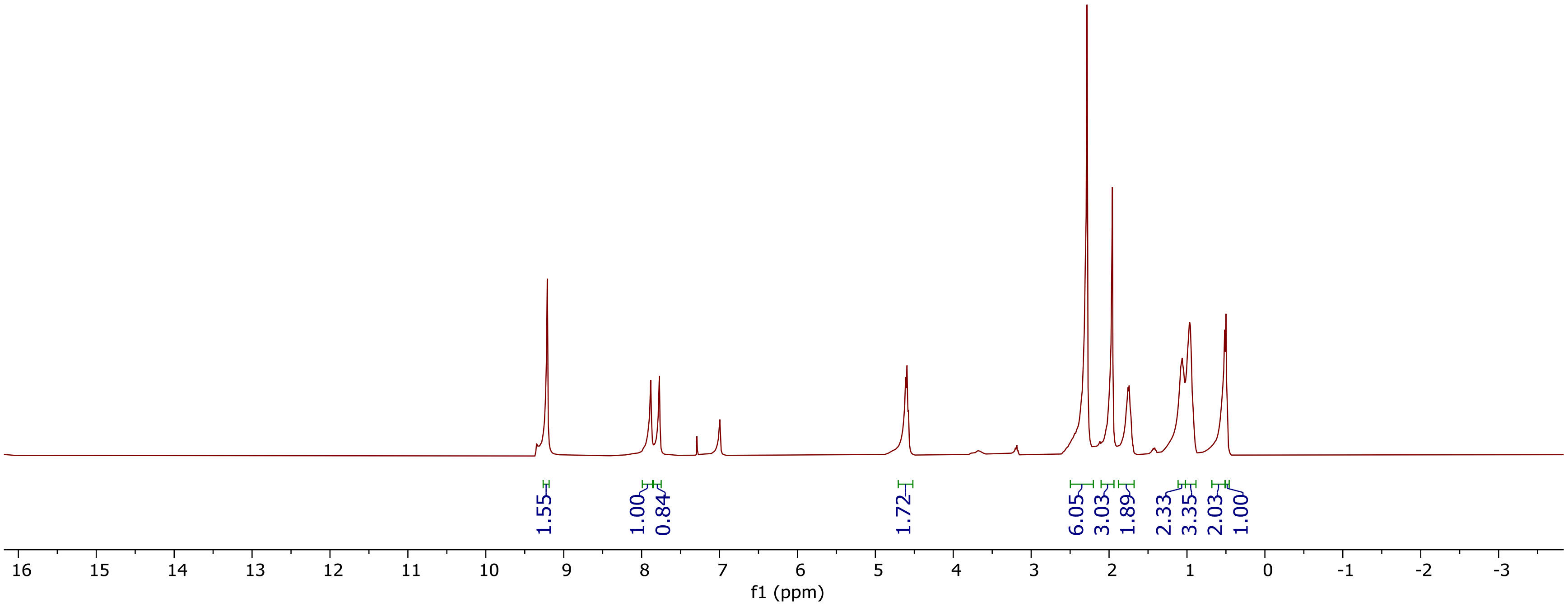

[4y]
13C NMR at 100.63 MHz in CDCl3

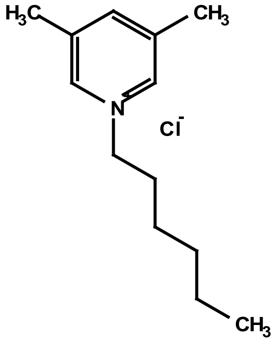

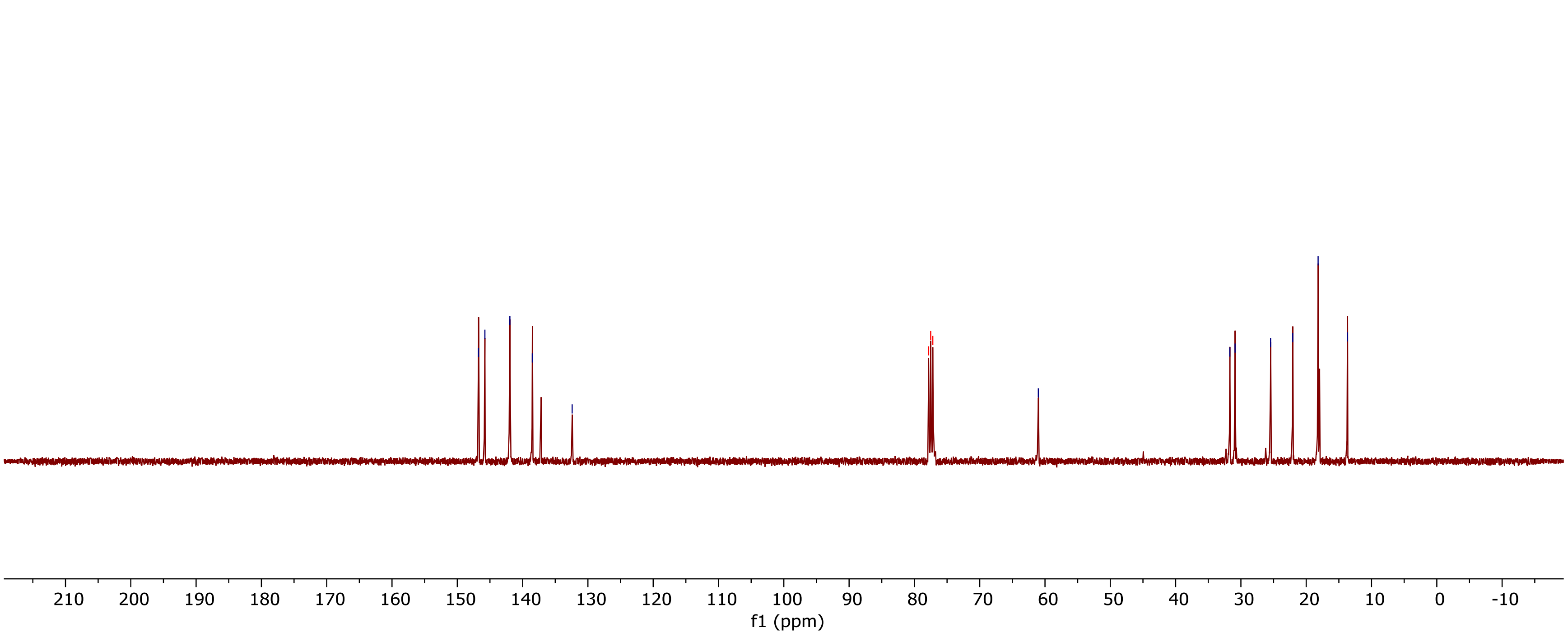

[19]
1H NMR at 800.34 MHz in CDCl3

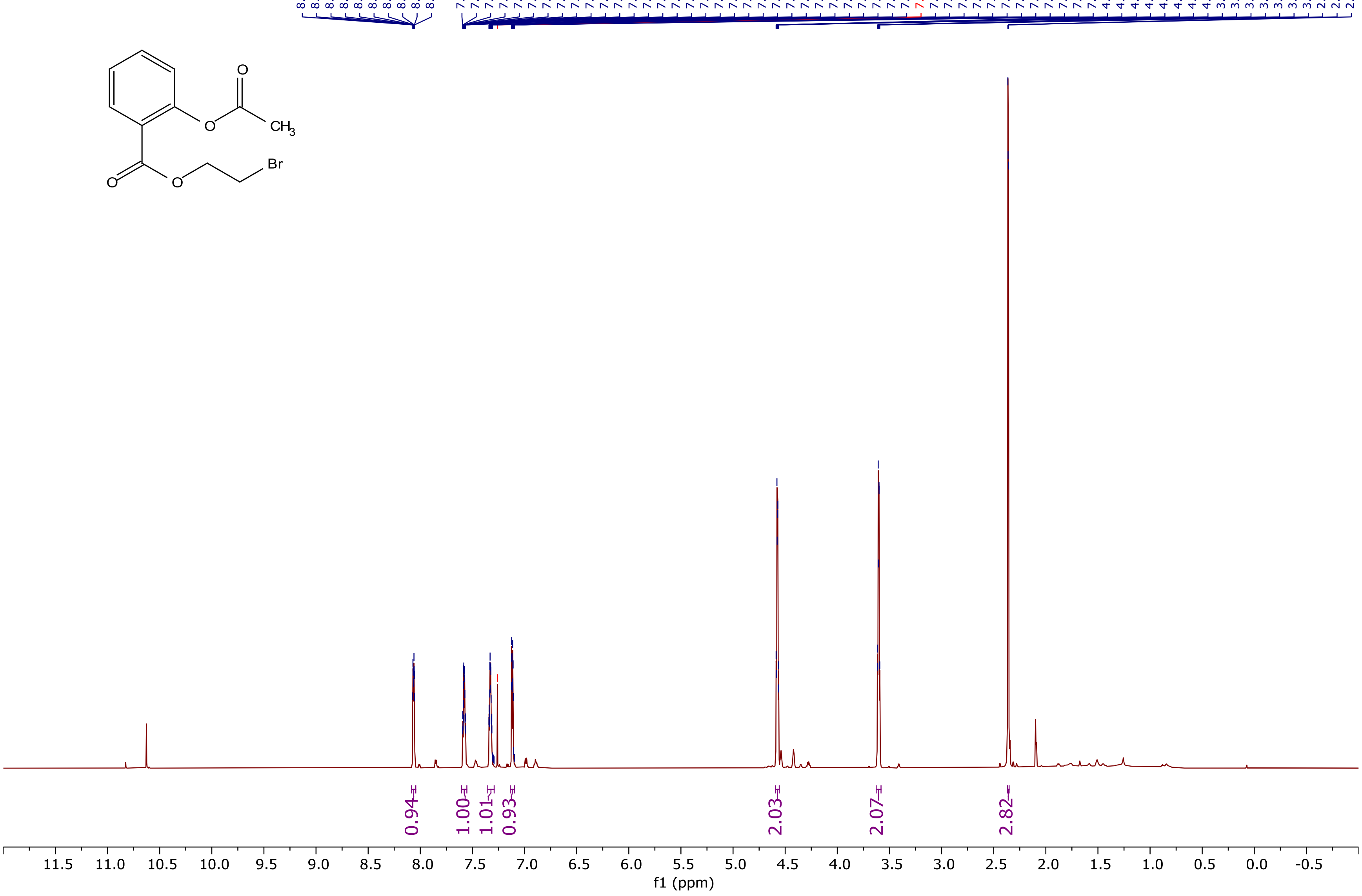

[19]
13C NMR at 201.27 MHz in CDCl3

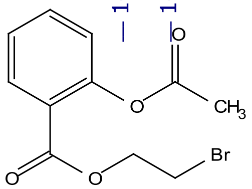

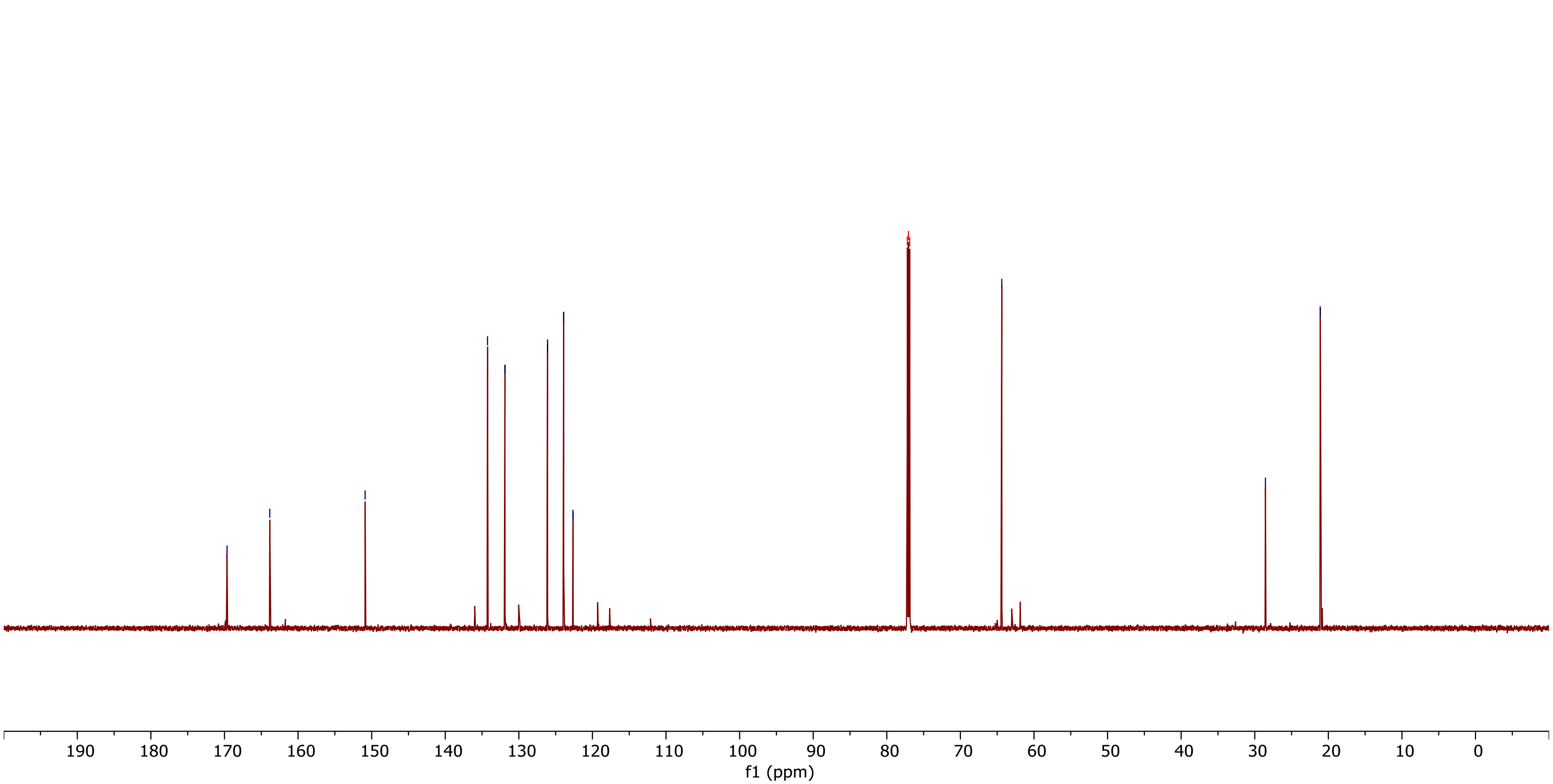

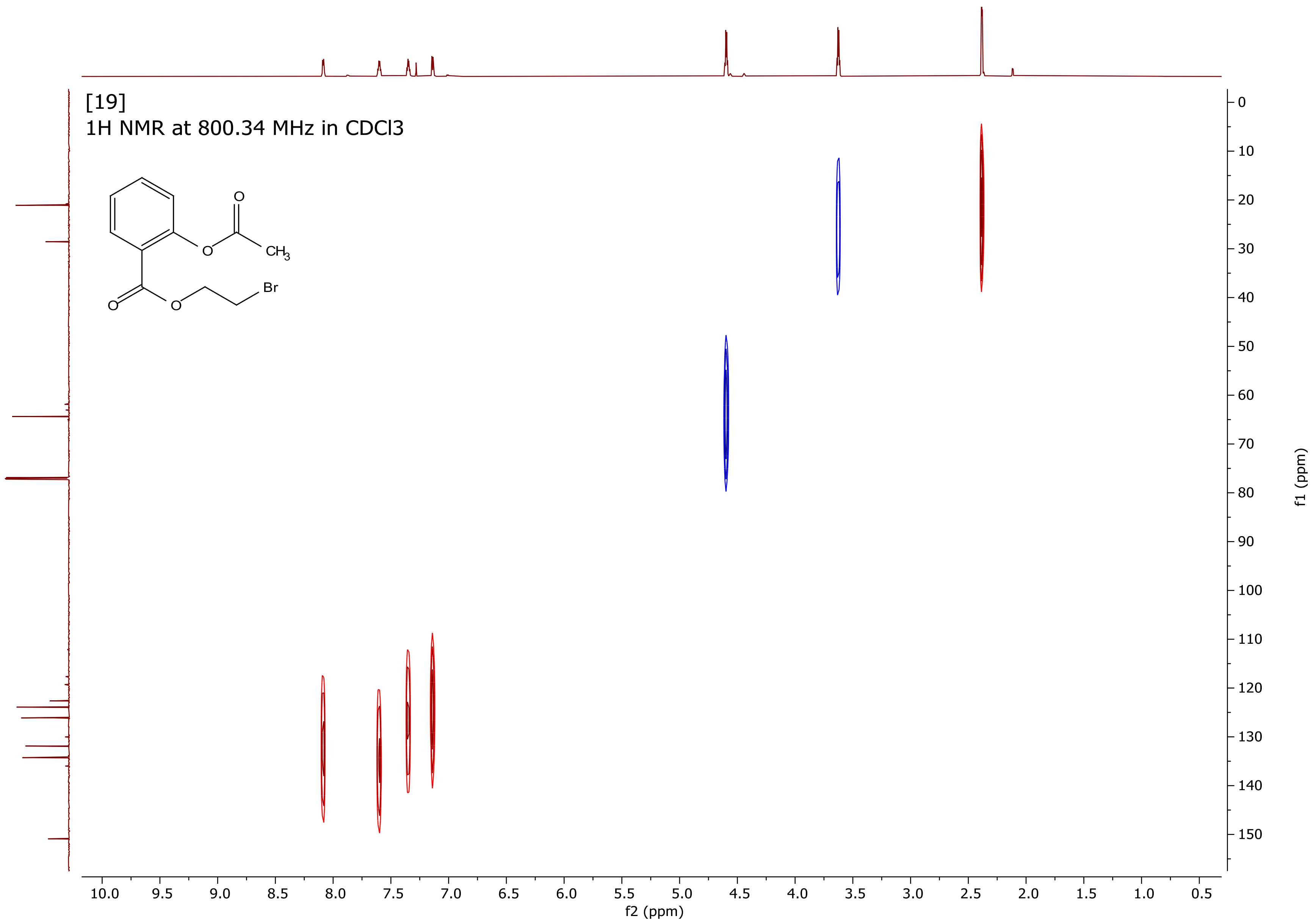
[19]
1H NMR at 800.34 MHz in CDCl3
O
CH3
O
Br
O
O
f2 (ppm)
f1 (ppm)

[36]
1H NMR at 400.15 MHz in CDCl3

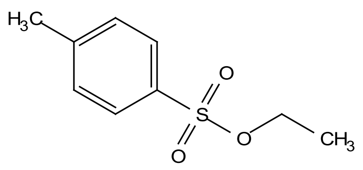

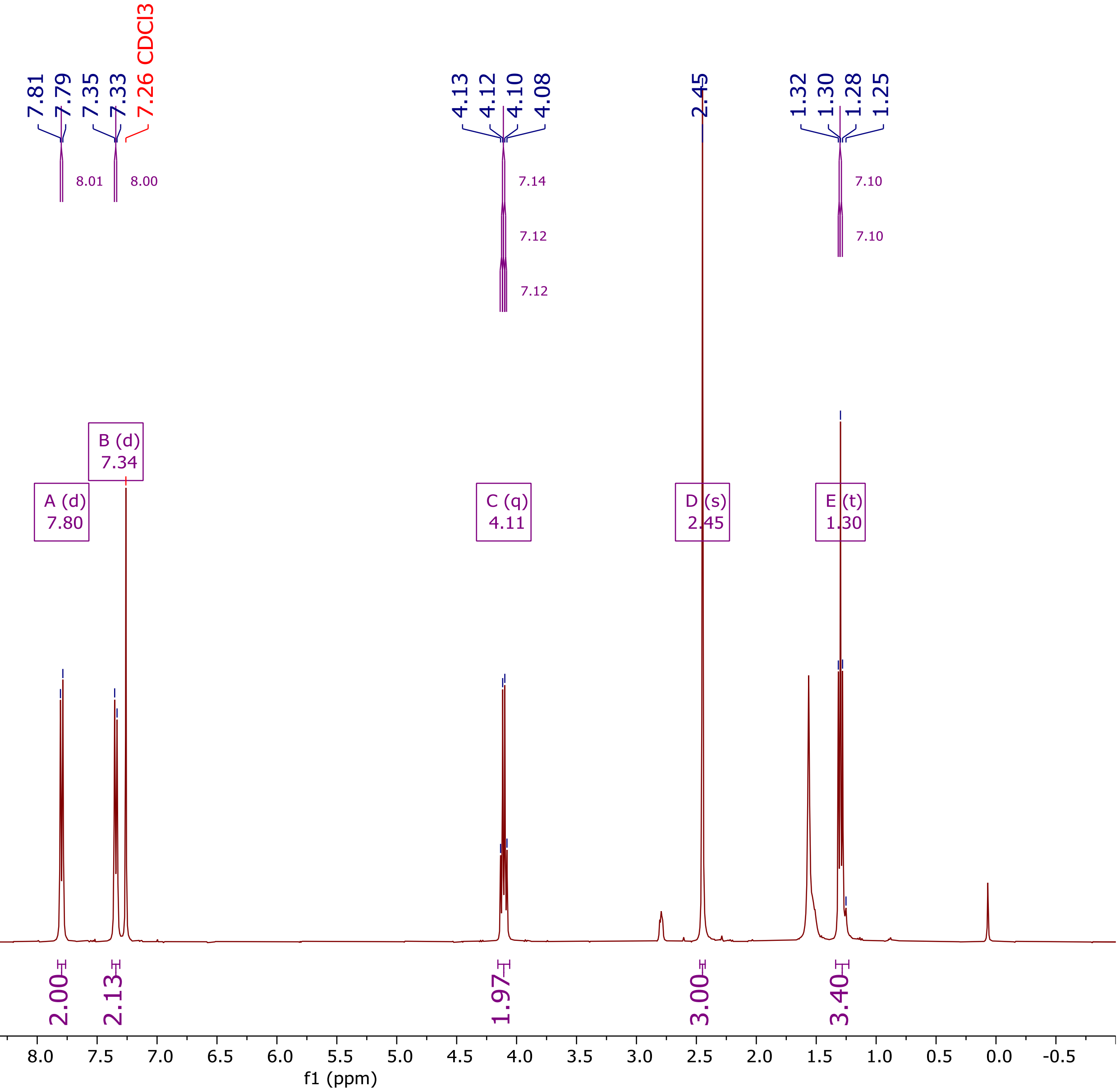

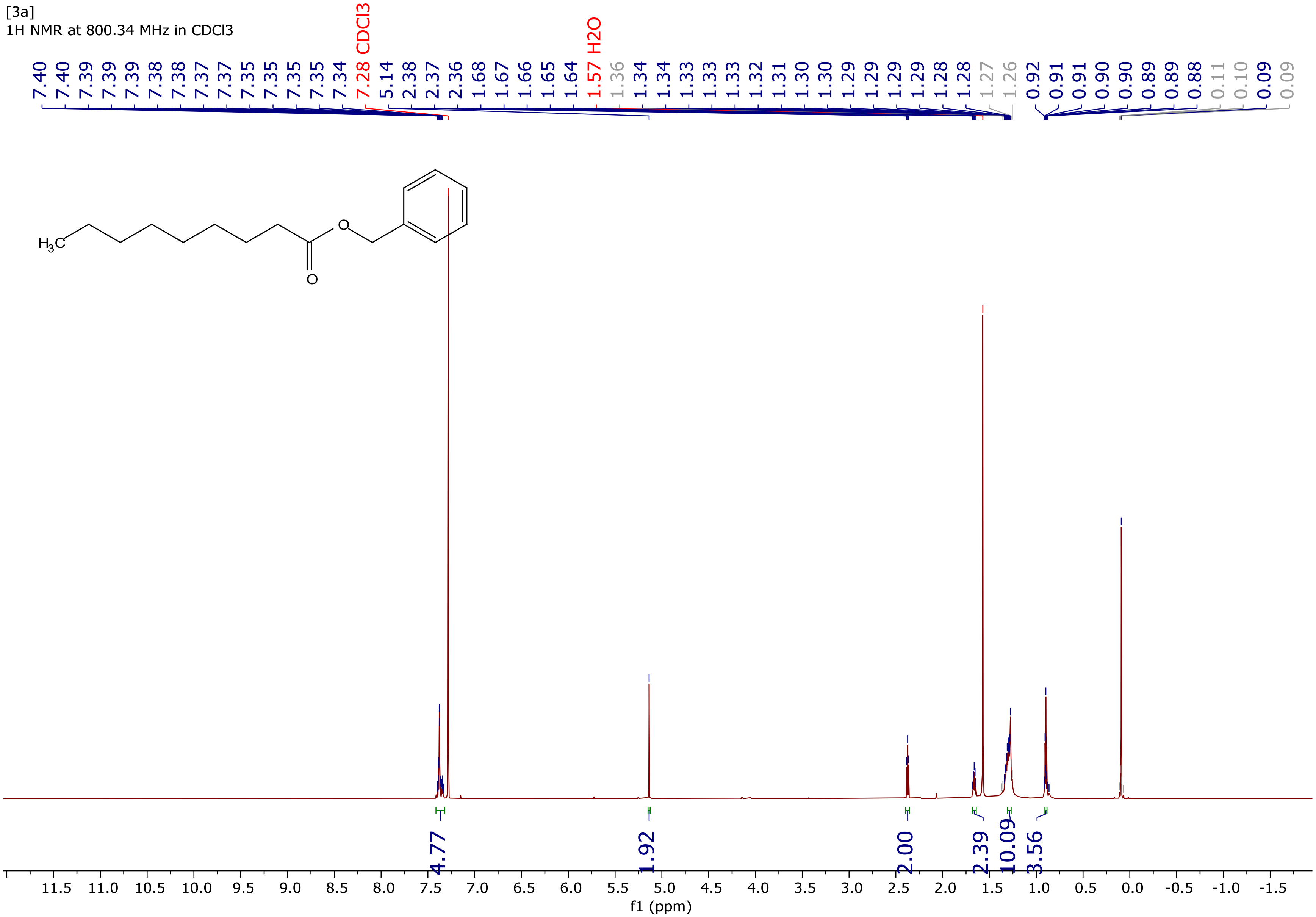
[3a]
1H NMR at 800.34 MHz in CDCl3
7.40 7.40 7.39 7.39 7.39 7.38 7.38 7.37 7.37 7.35 7.35 7.35 7.35 7.34 7.28 CDCl3 5.14 2.38 2.37 2.36 1.68 1.67 1.66 1.65 1.64 1.57 H2O 1.36 1.34 1.34 1.33 1.33 1.33 1.32 1.31 1.30 1.30 1.29 1.29 1.29 1.29 1.28 1.28 1.27 1.26 0.92 0.91 0.91 0.90 0.90 0.89 0.89 0.88 0.11 0.10 0.09 0.09
H3C
O
O
4.77
1.92
2.00
2.39
10.09
3.56
11.5 11.0 10.5 10.0 9.5 9.0 8.5 8.0 7.5 7.0 6.5 6.0 5.5 5.0 4.5 4.0 3.5 3.0 2.5 2.0 1.5 1.0 0.5 0.0 -0.5 -1.0 -1.5
f1 (ppm)

[3a]
13C NMR at 201.27 MHz in CDCl3

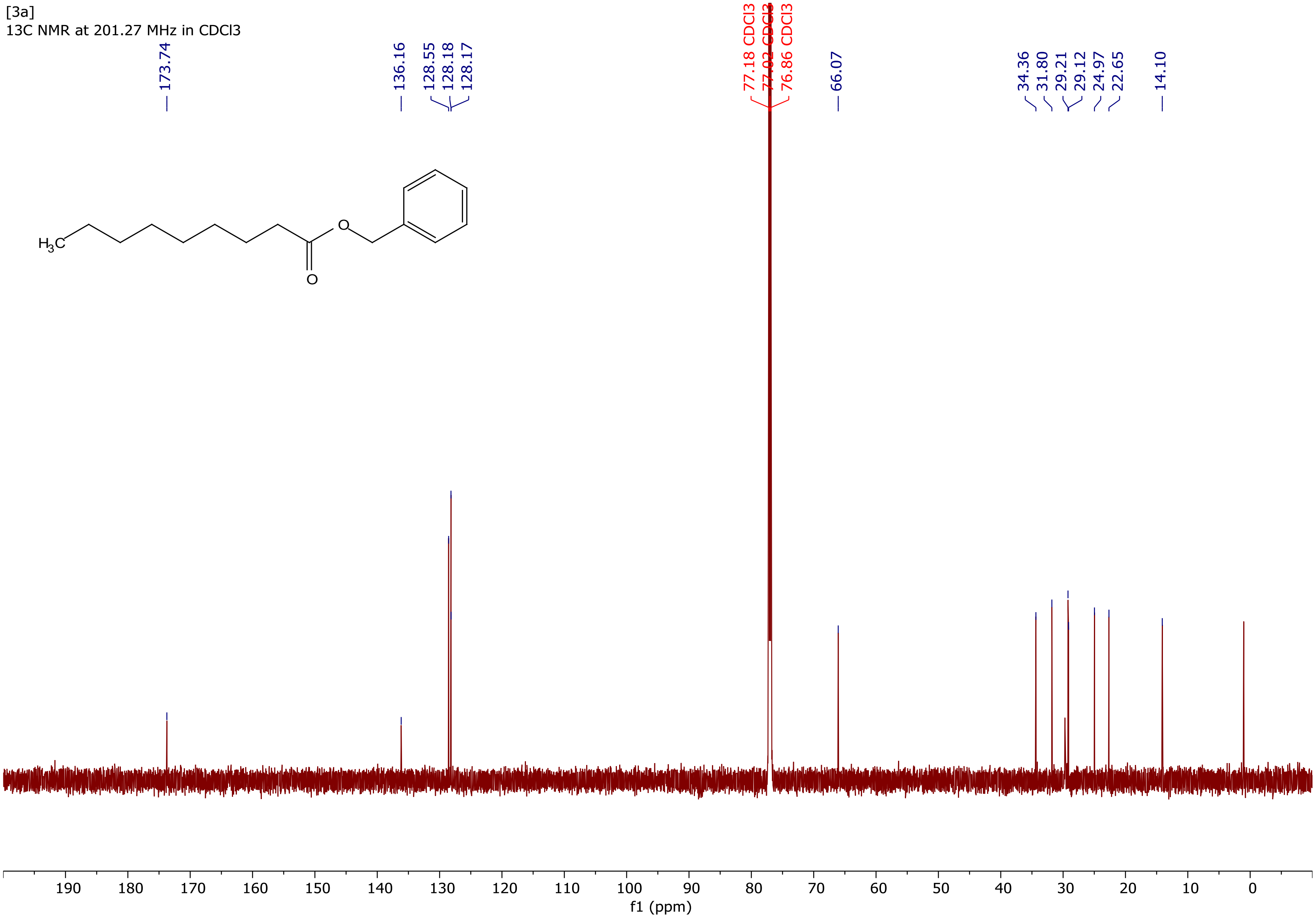

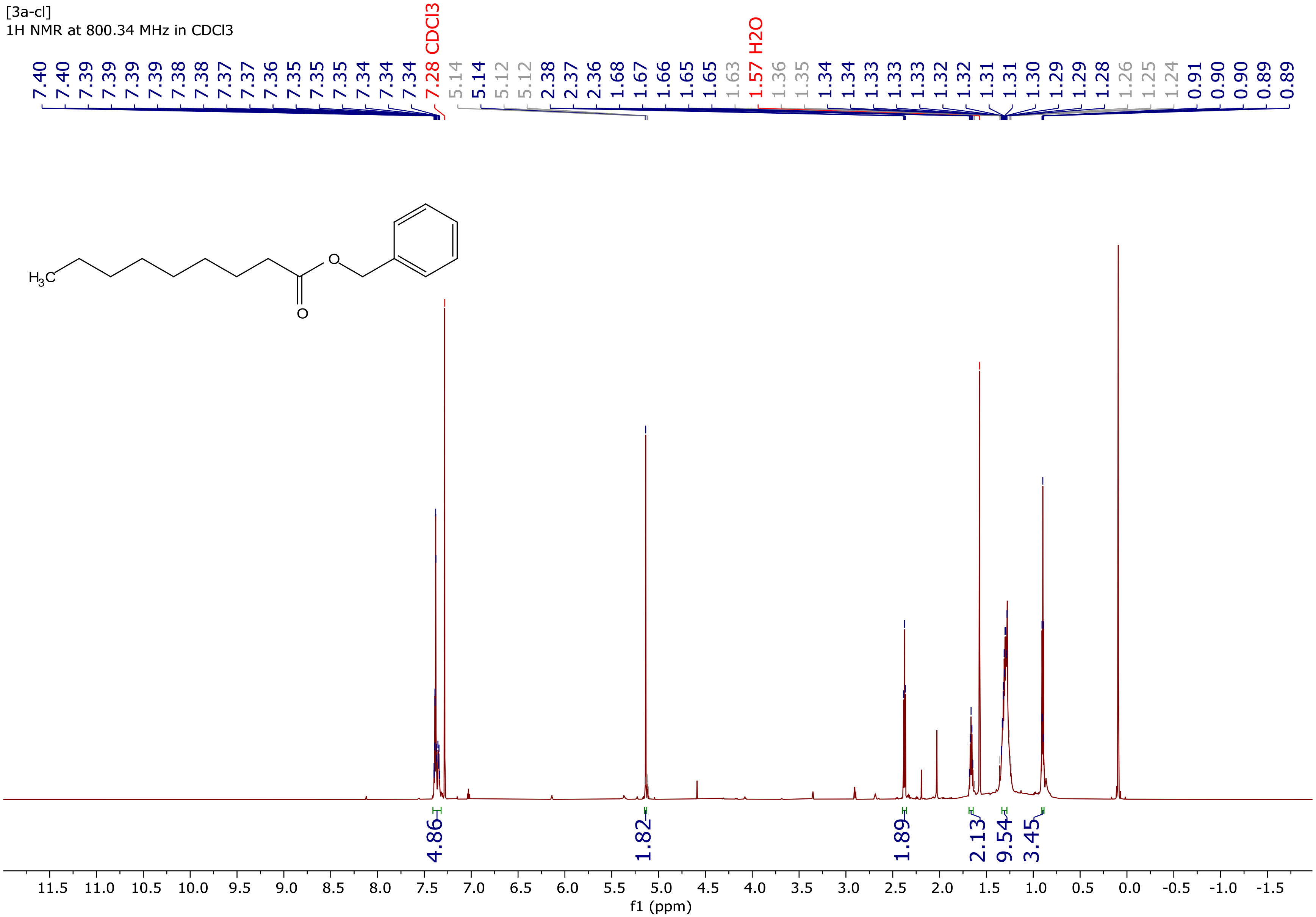
[3a-cl]
1H NMR at 800.34 MHz in CDCl3
7.40 7.40 7.39 7.39 7.39 7.39 7.38 7.38 7.37 7.37 7.36 7.35 7.35 7.35 7.34 7.34 7.34 7.28 CDCl3 5.14 5.14 5.12 5.12 2.38 2.37 2.36 1.68 1.67 1.66 1.65 1.65 1.63 1.57 H2O 1.36 1.35 1.34 1.34 1.33 1.33 1.33 1.32 1.32 1.31 1.31 1.30 1.29 1.29 1.28 1.26 1.25 1.24 0.91 0.90 0.90 0.89 0.89
H3C
O
O
4.86
1.82
1.89
2.13
9.54
3.45
11.5 11.0 10.5 10.0 9.5 9.0 8.5 8.0 7.5 7.0 6.5 6.0 5.5 5.0 4.5 4.0 3.5 3.0 2.5 2.0 1.5 1.0 0.5 0.0 -0.5 -1.0 -1.5
f1 (ppm)

[3a-cl]
13C NMR at 201.27 MHz in CDCl3

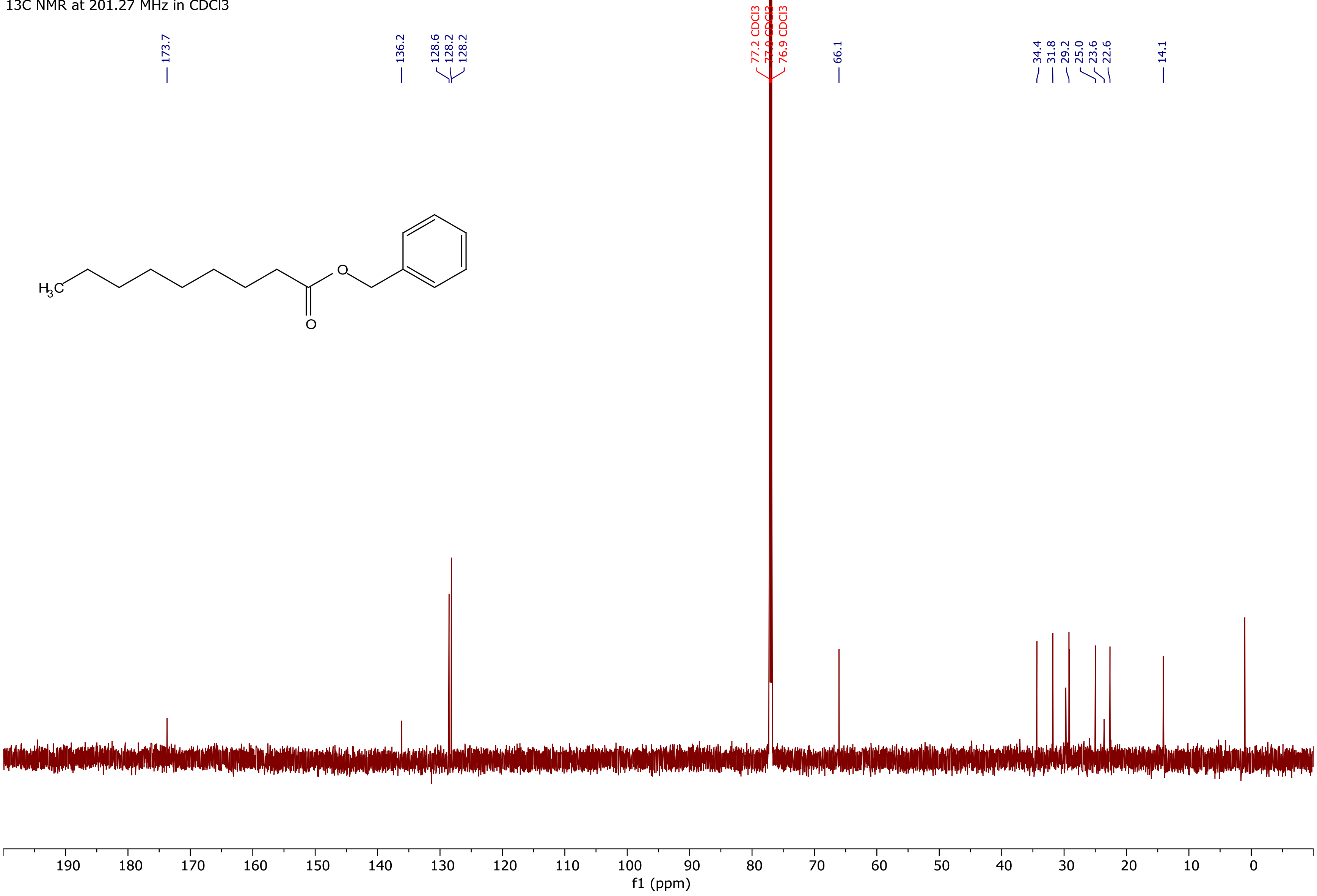

[5a]
1H NMR at 800.34 MHz in CDCl3

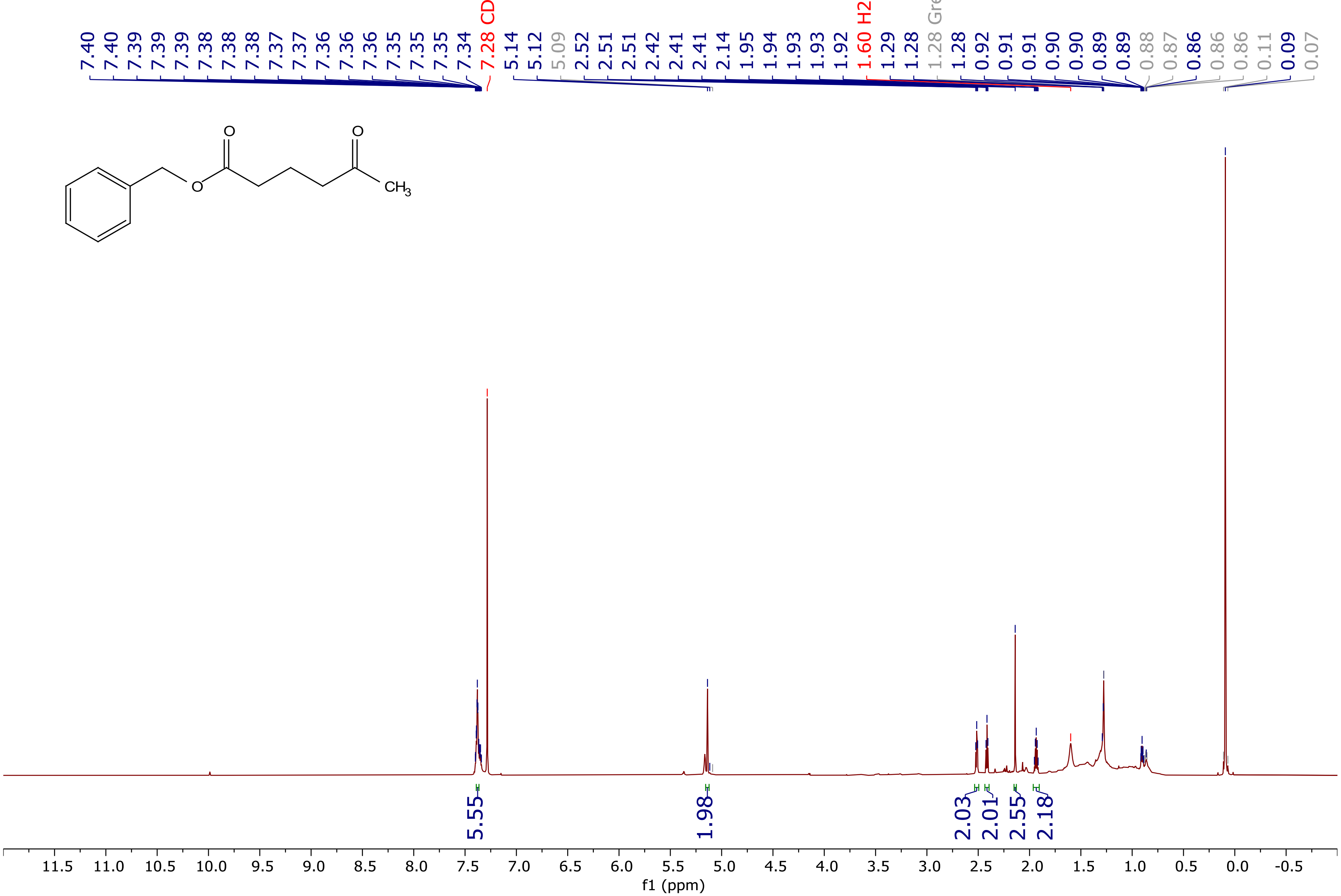

[5a]
13C NMR at 201.27 MHz in CDCl3

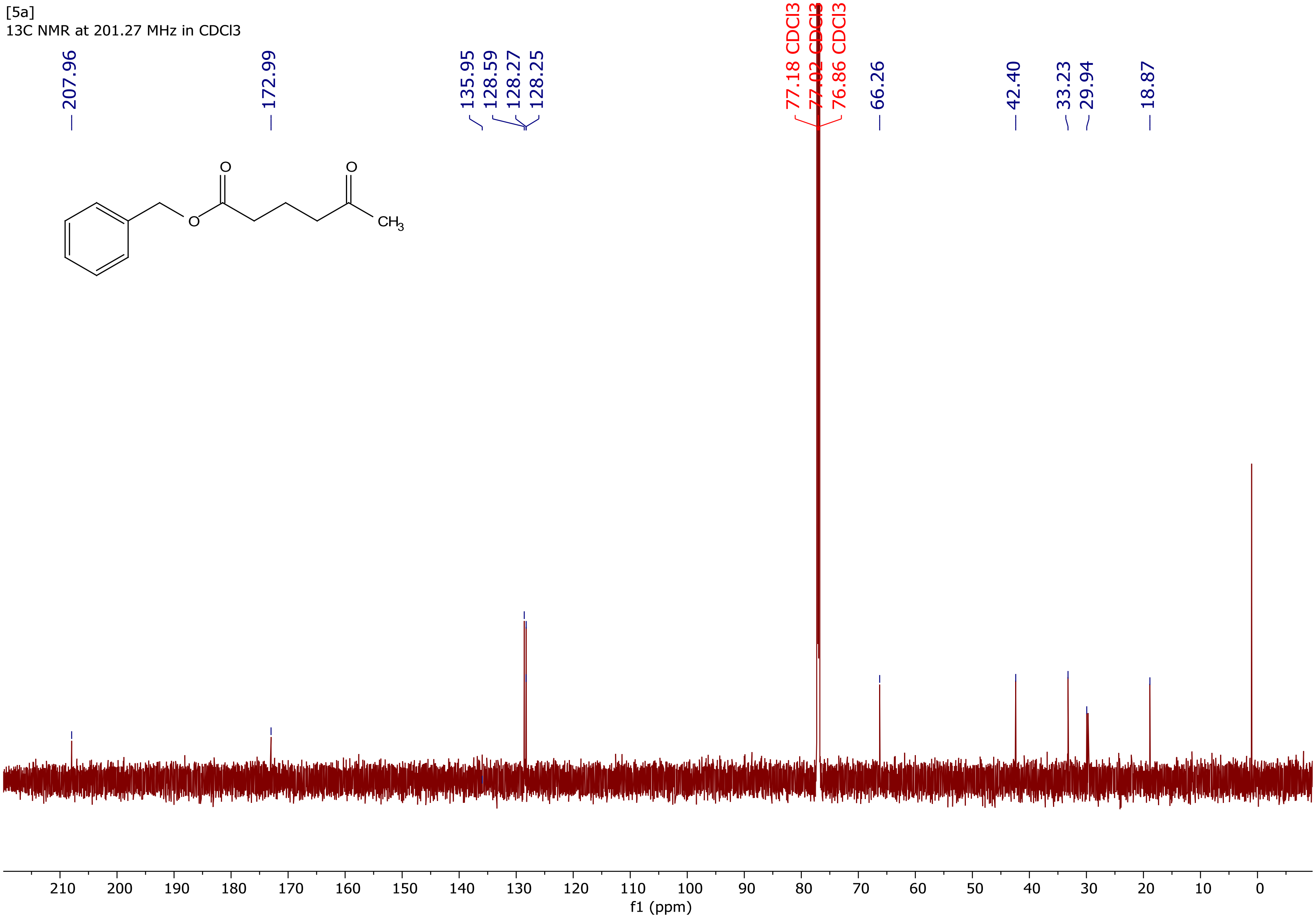

[6a]
1H NMR at 400.15 MHz in CDCl3

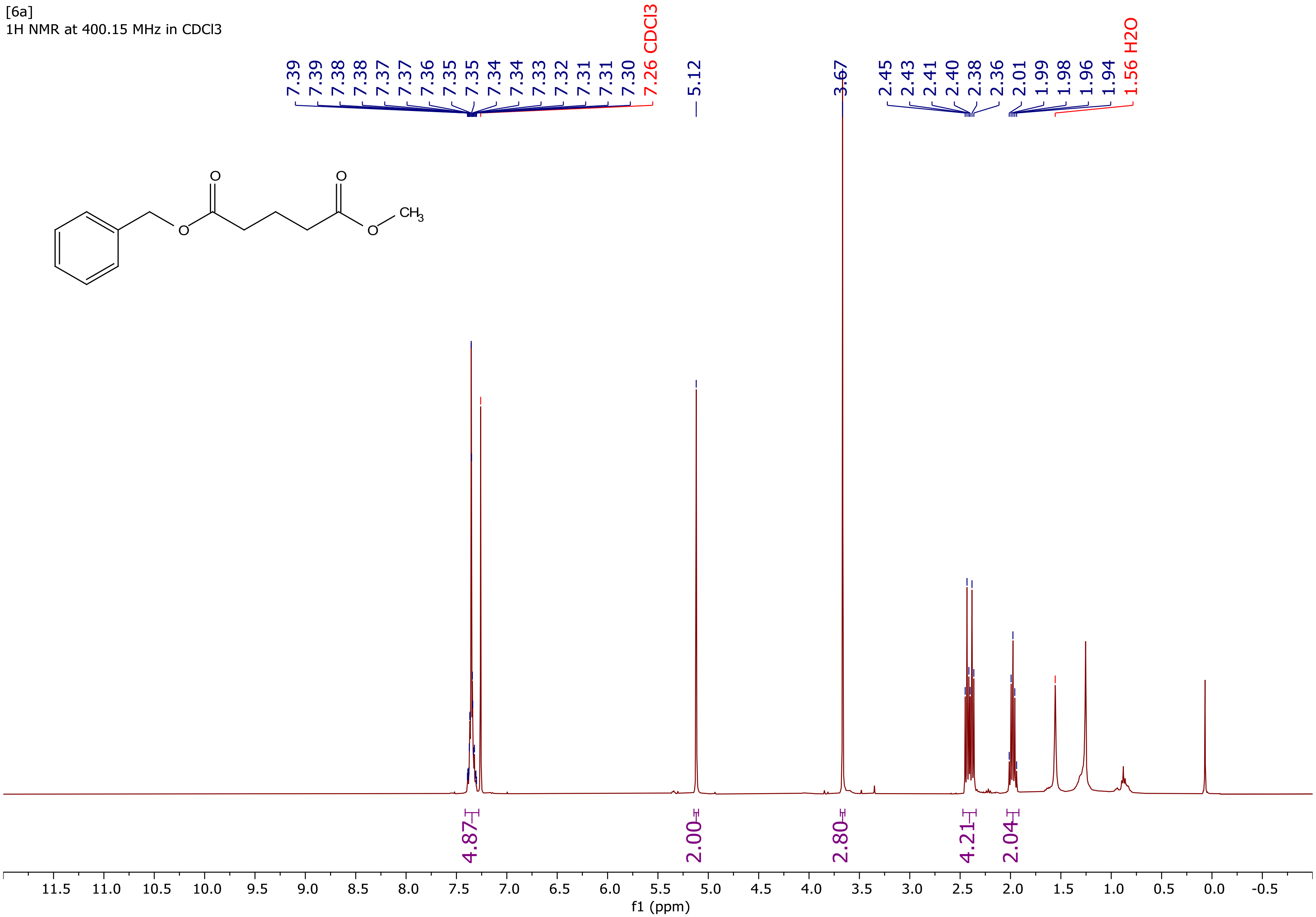

[6a]
13C NMR at 100.63 MHz in CDCl3

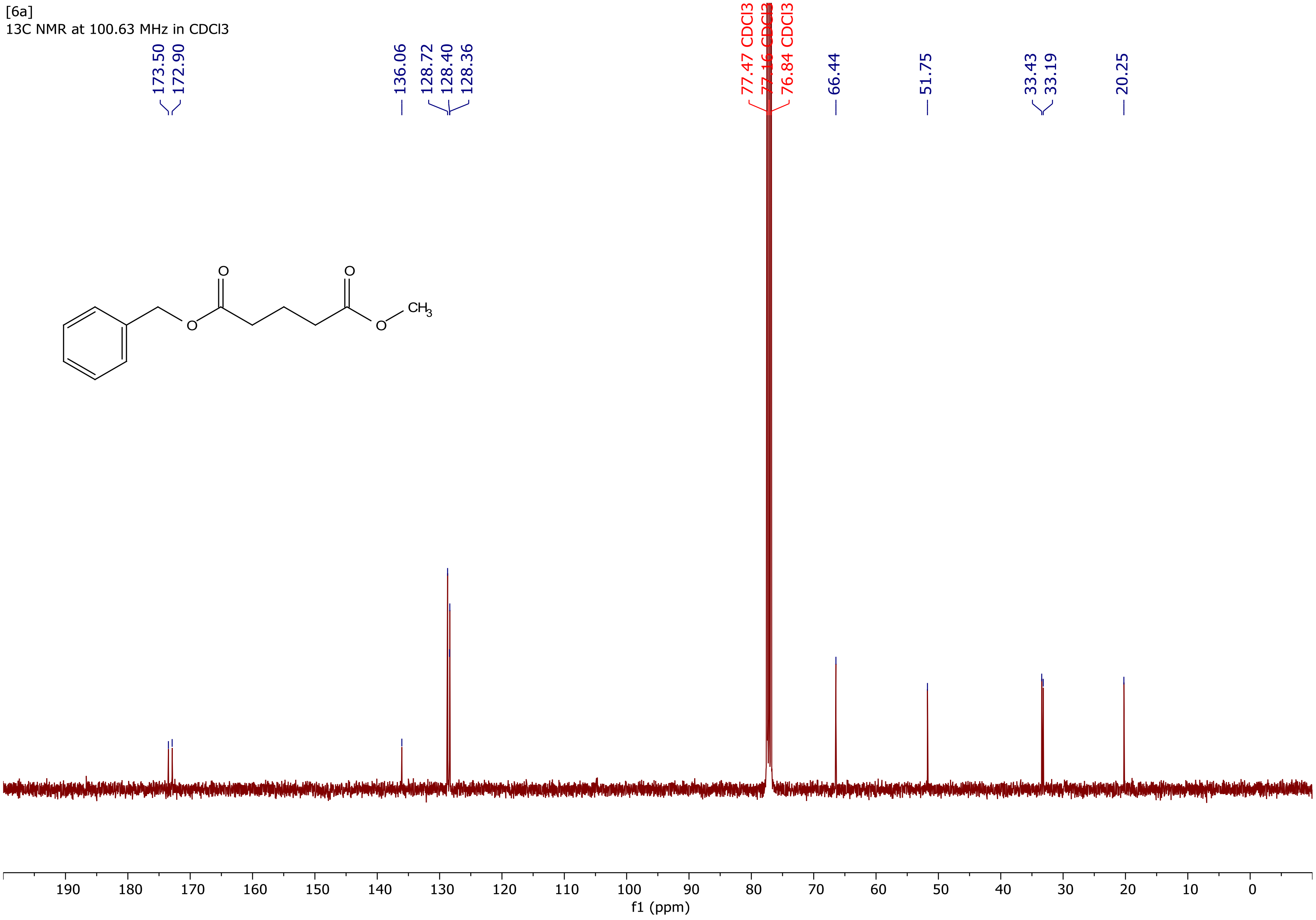

[7a-Cl]
1H NMR at 400.15 MHz in CDCl3

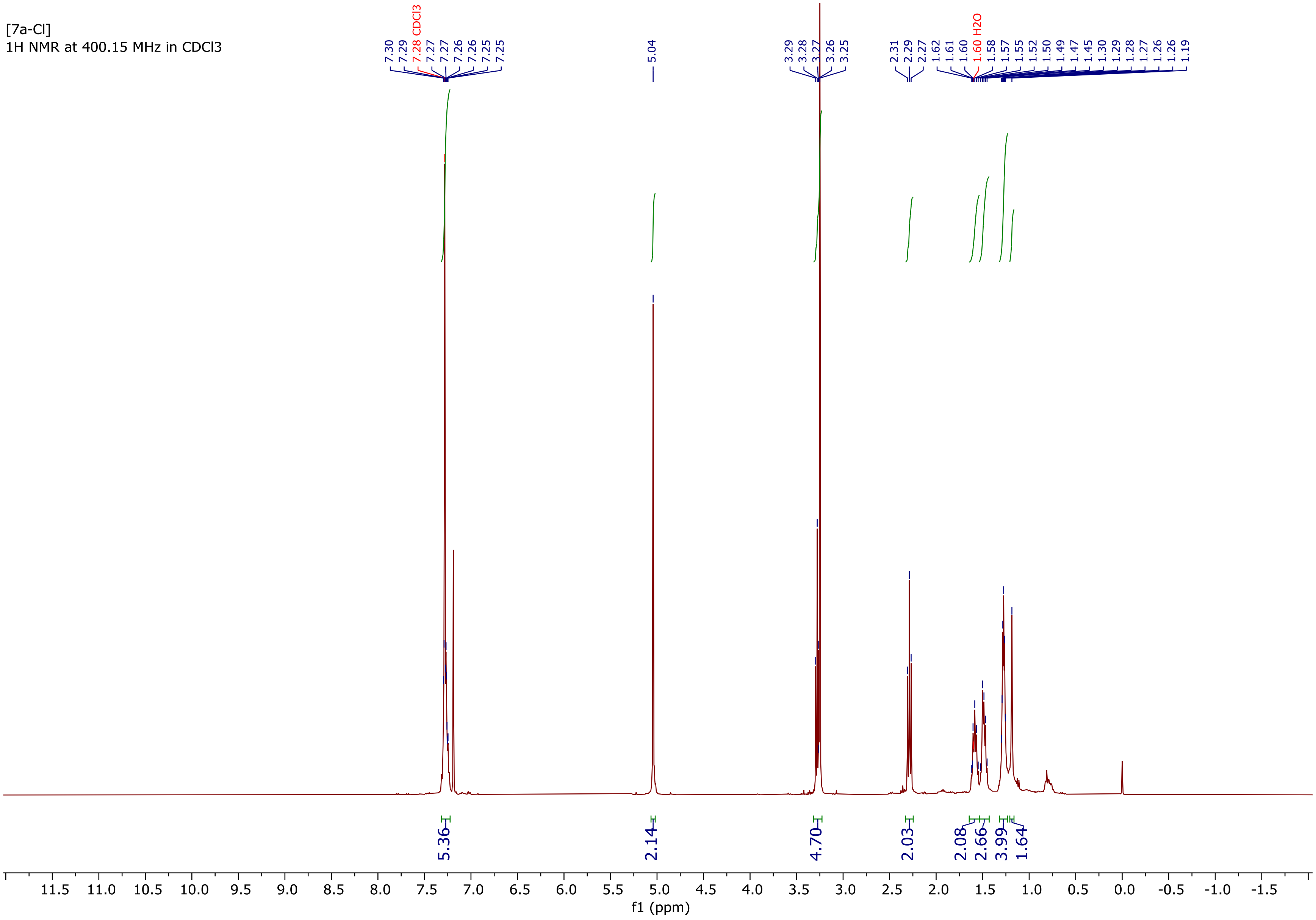

[7a-Br]
1H NMR at 400.15 MHz in CDCl3

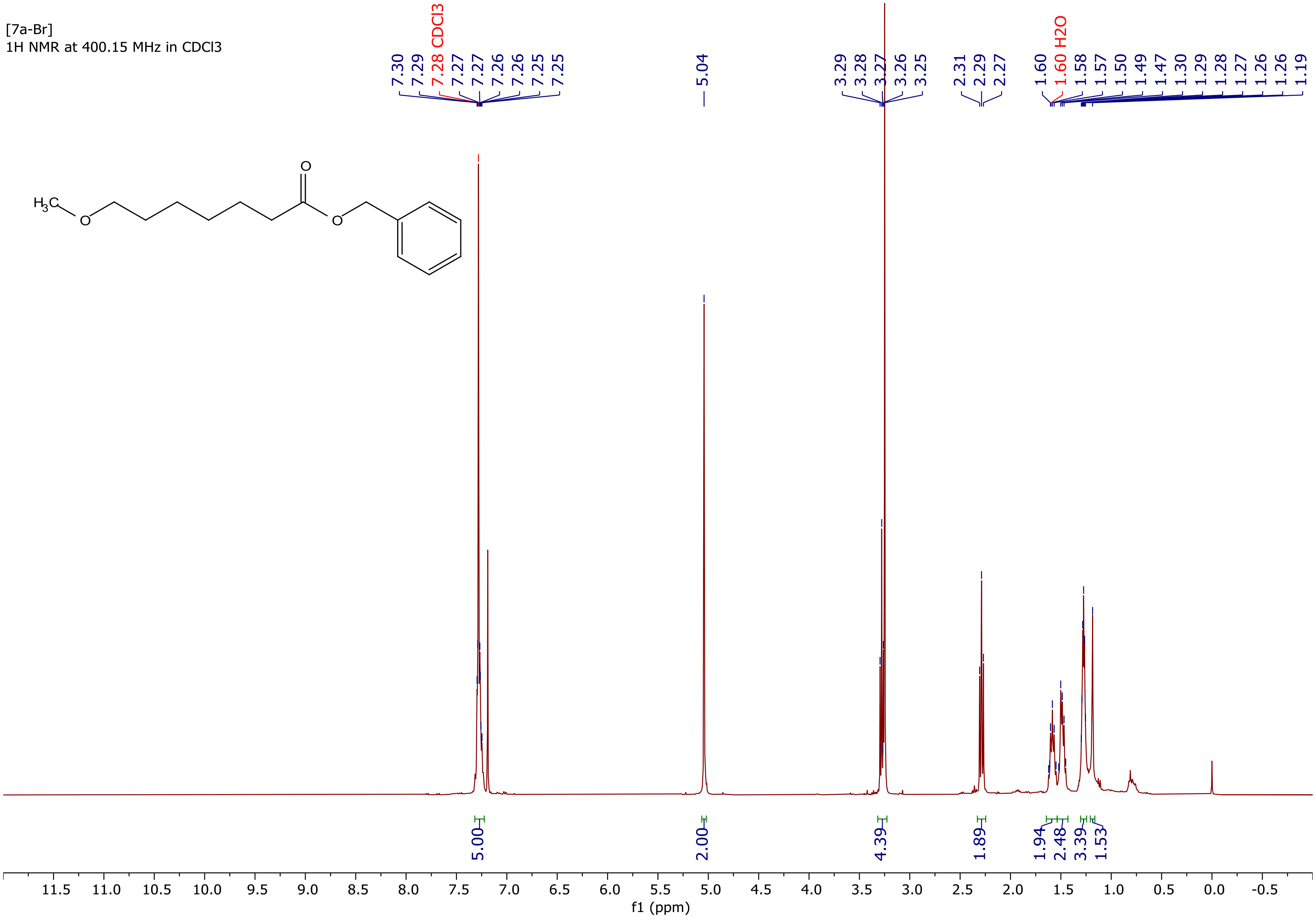

[7a-Br]
13C NMR at 201.27 MHz in CDCl3

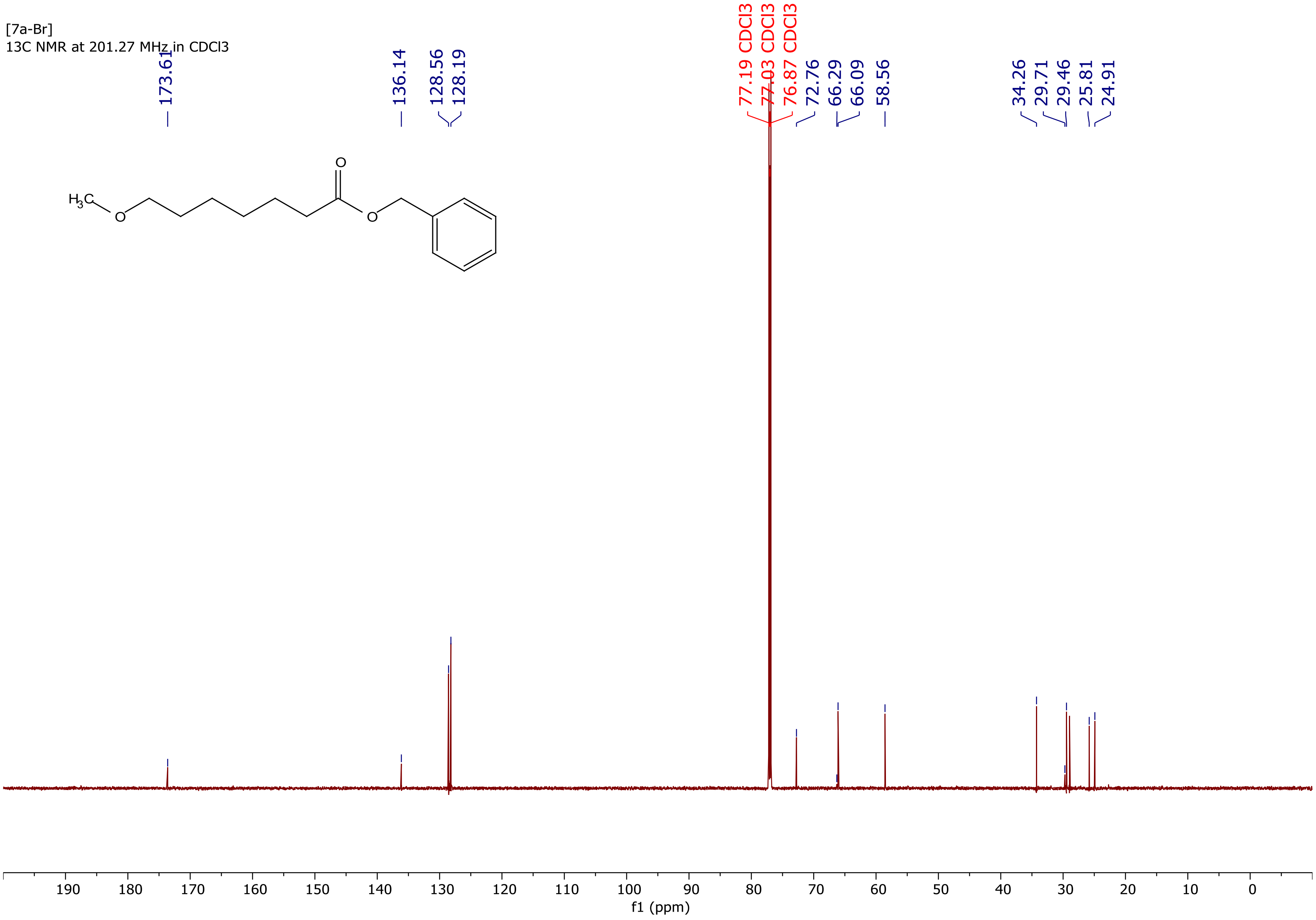

[8a]
1H NMR at 400.15 MHz in CDCl3

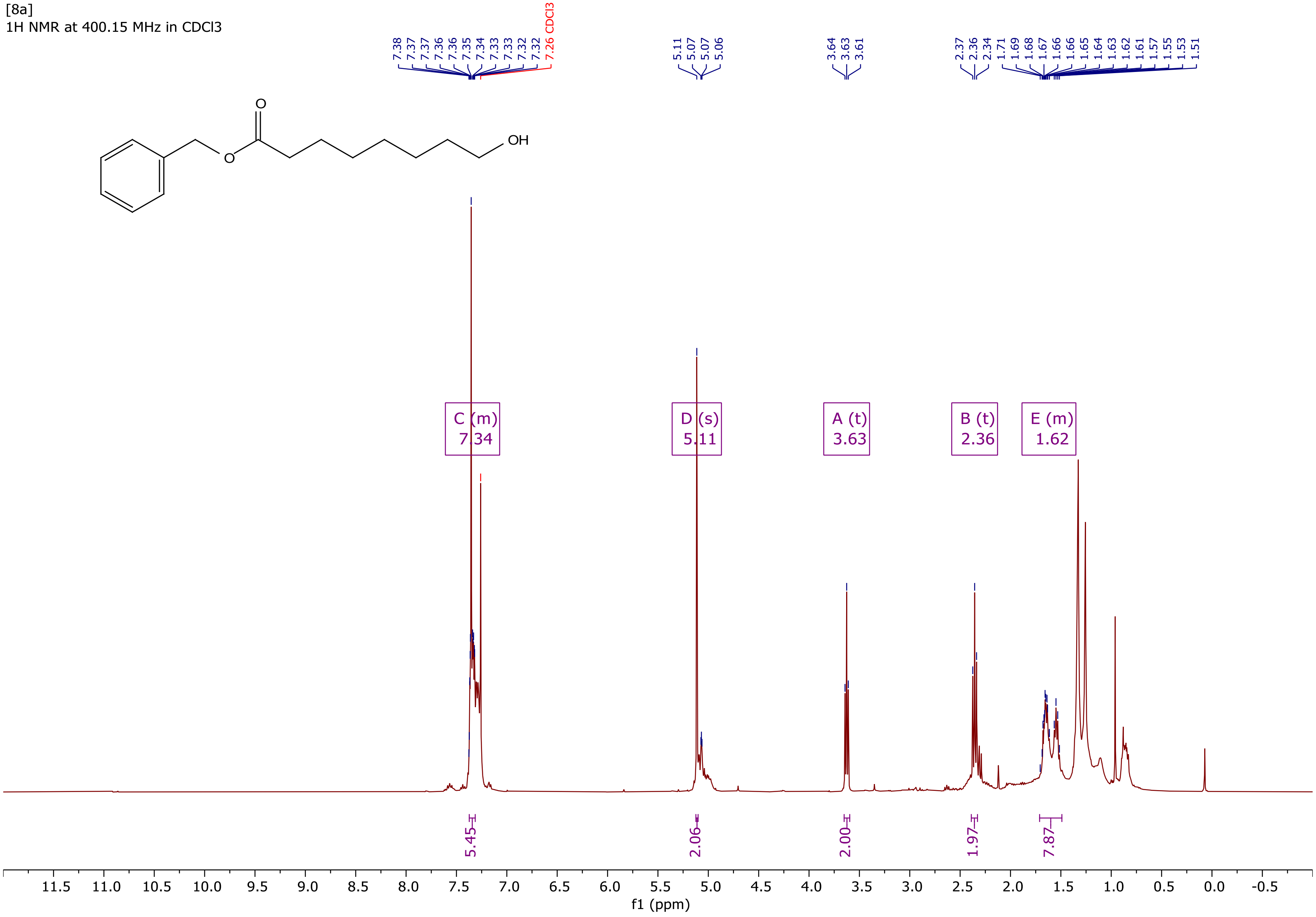

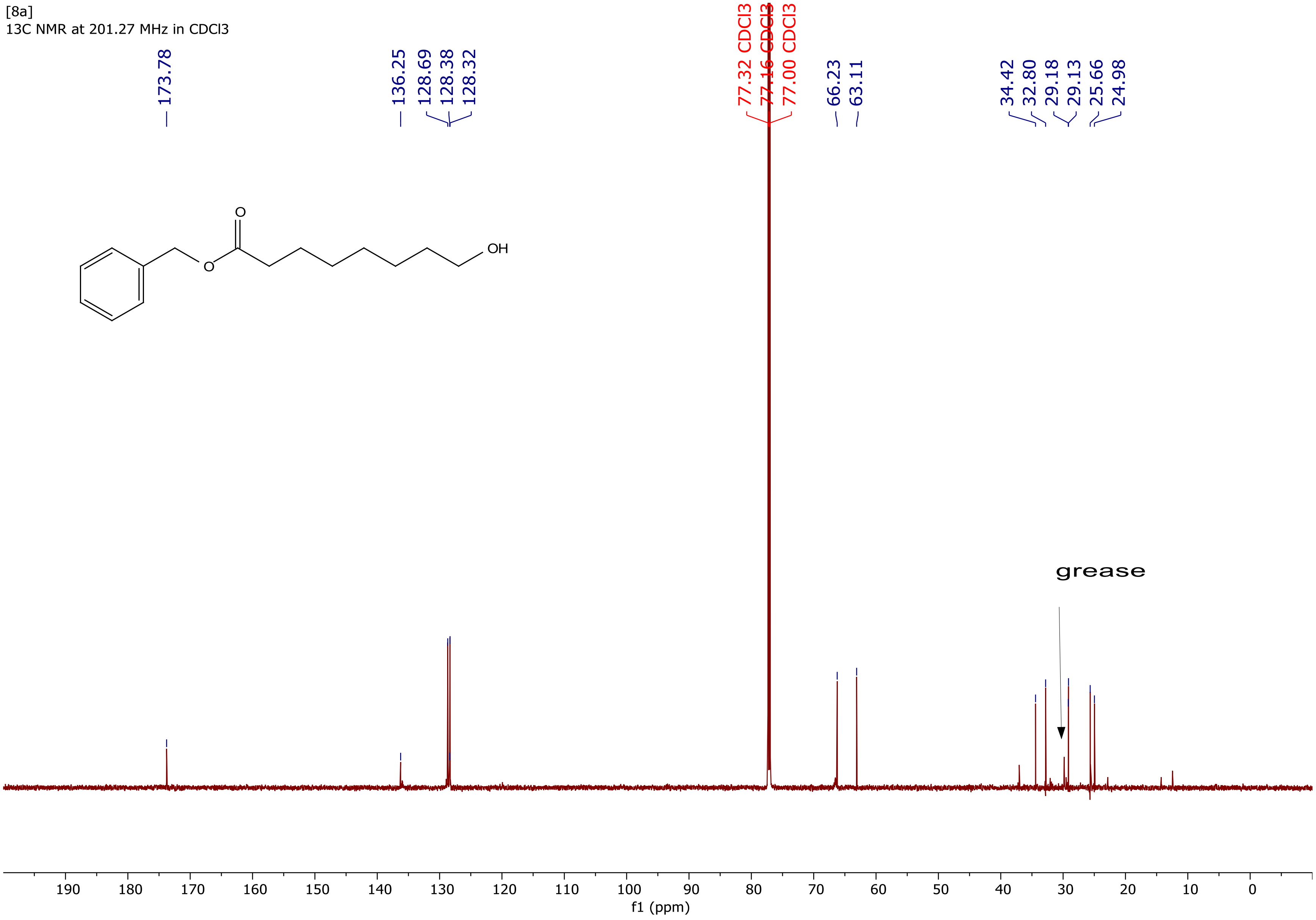
[8a]
13C NMR at 201.27 MHz in CDCl3
173.78
136.25
128.69
128.38
128.32
77.32 CDCl3
77.16 CDCl3
77.00 CDCl3
66.23
63.11
34.42
32.80
29.18
29.13
25.66
24.98
O
O
OH
grease
190 180 170 160 150 140 130 120 110 100 90 80 70 60 50 40 30 20 10 0
f1 (ppm)

[9a]
1H NMR at 400.15 MHz in CDCl3

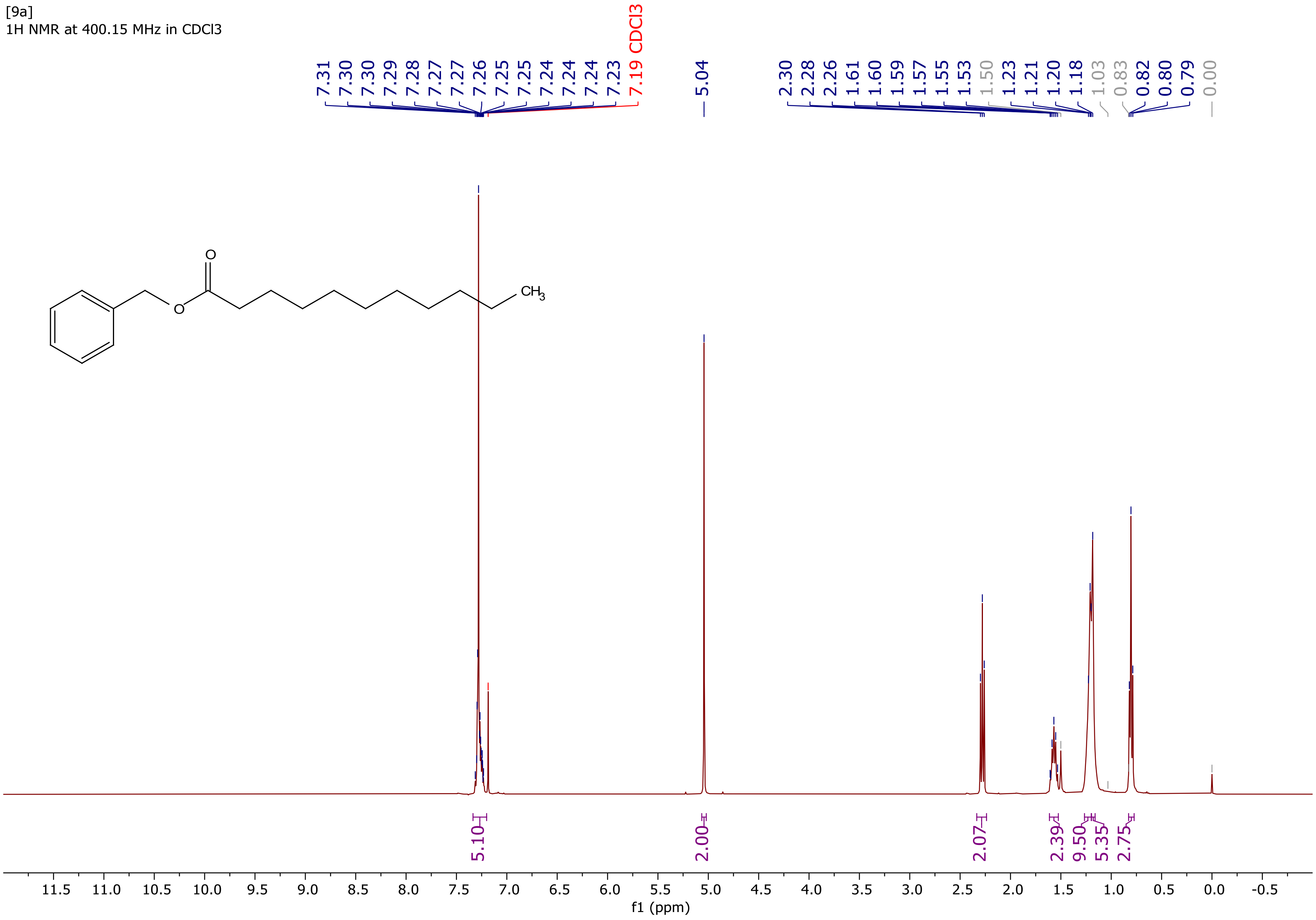

[9a]
13C NMR at 201.27 MHz in CDCl3

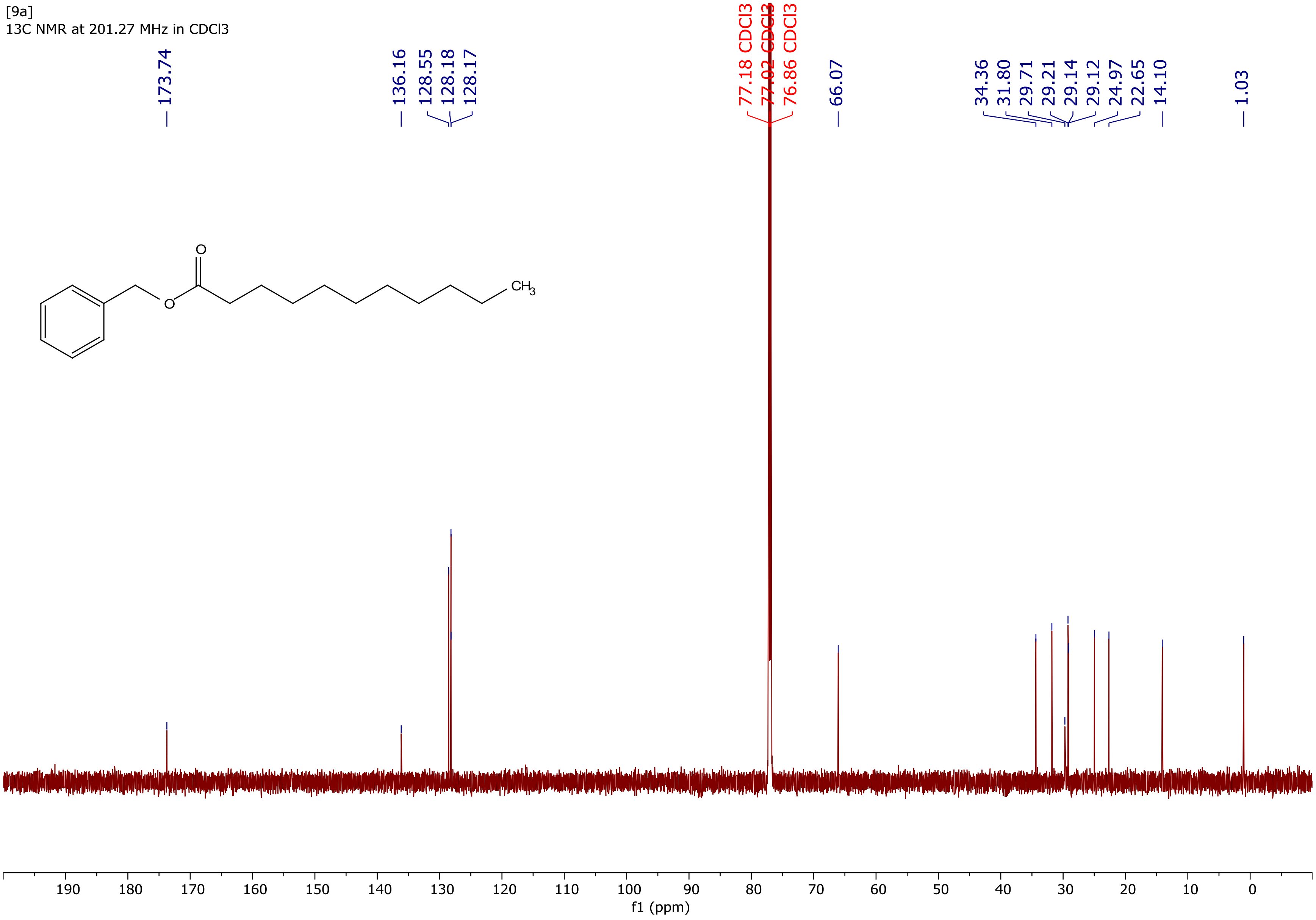

[10a]
1H NMR at 400.15 MHz in CDCl3

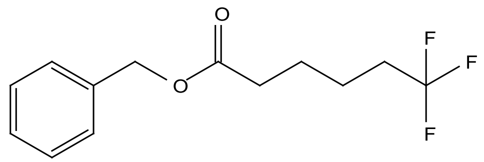

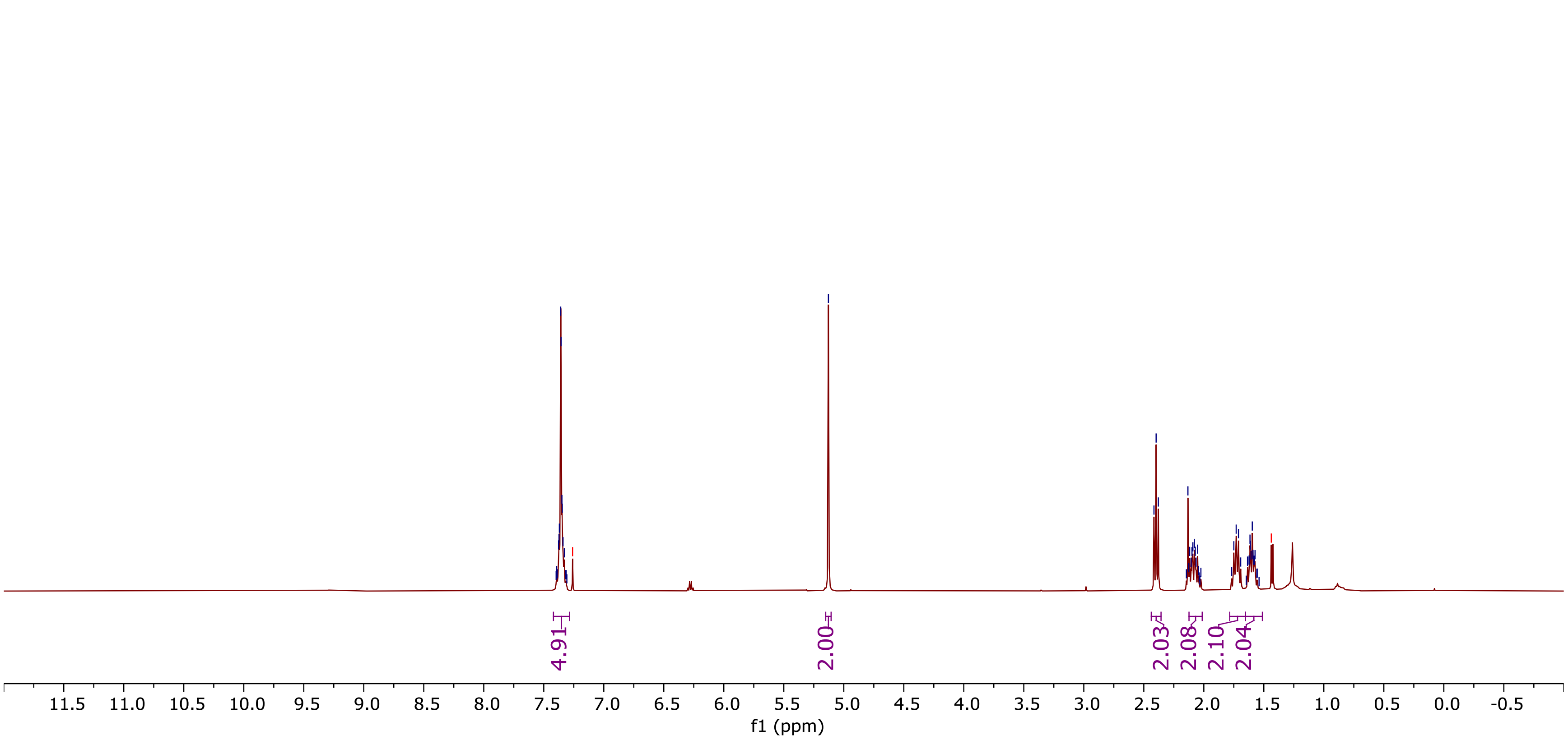

[10a]
13C NMR at 201.27 MHz in CDCl3

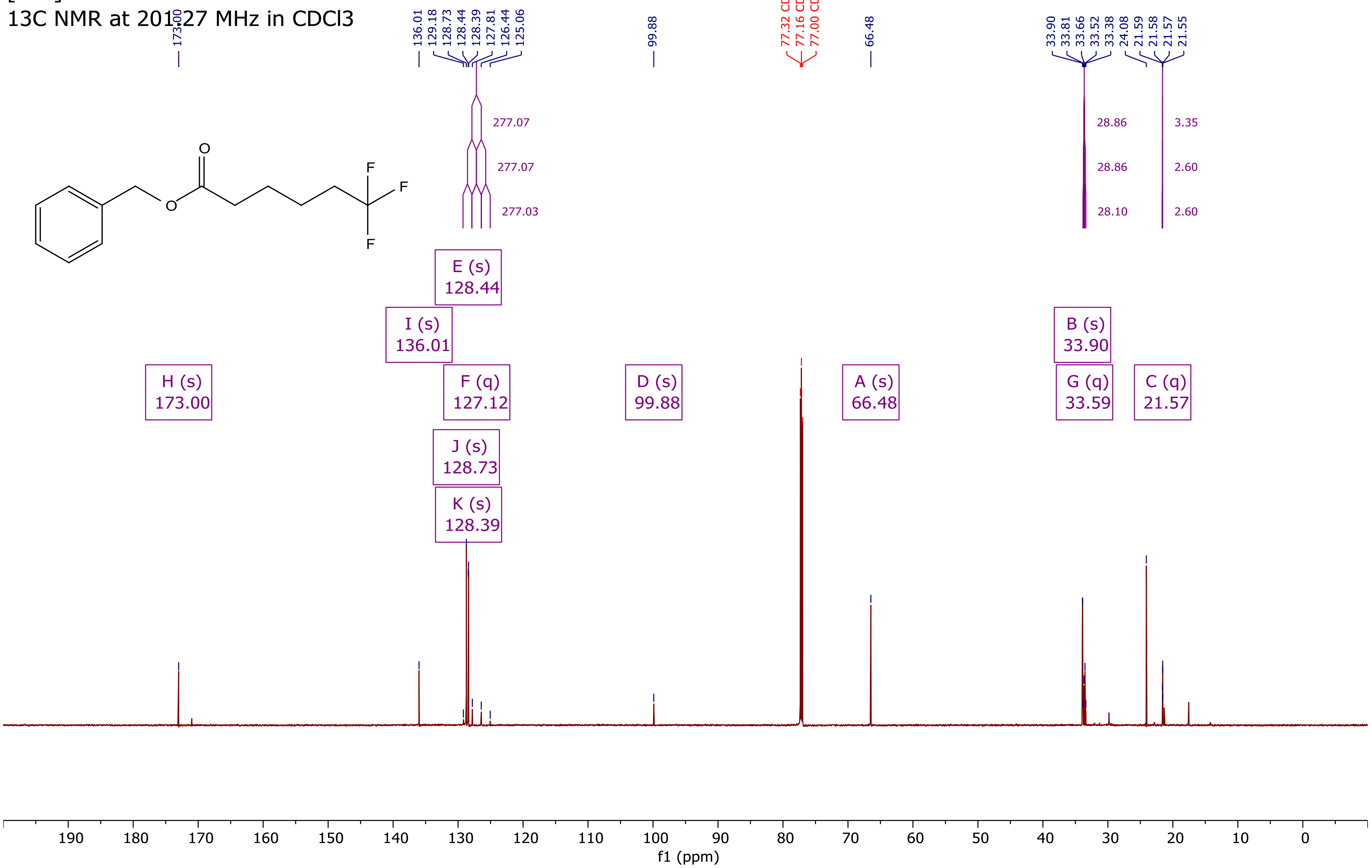

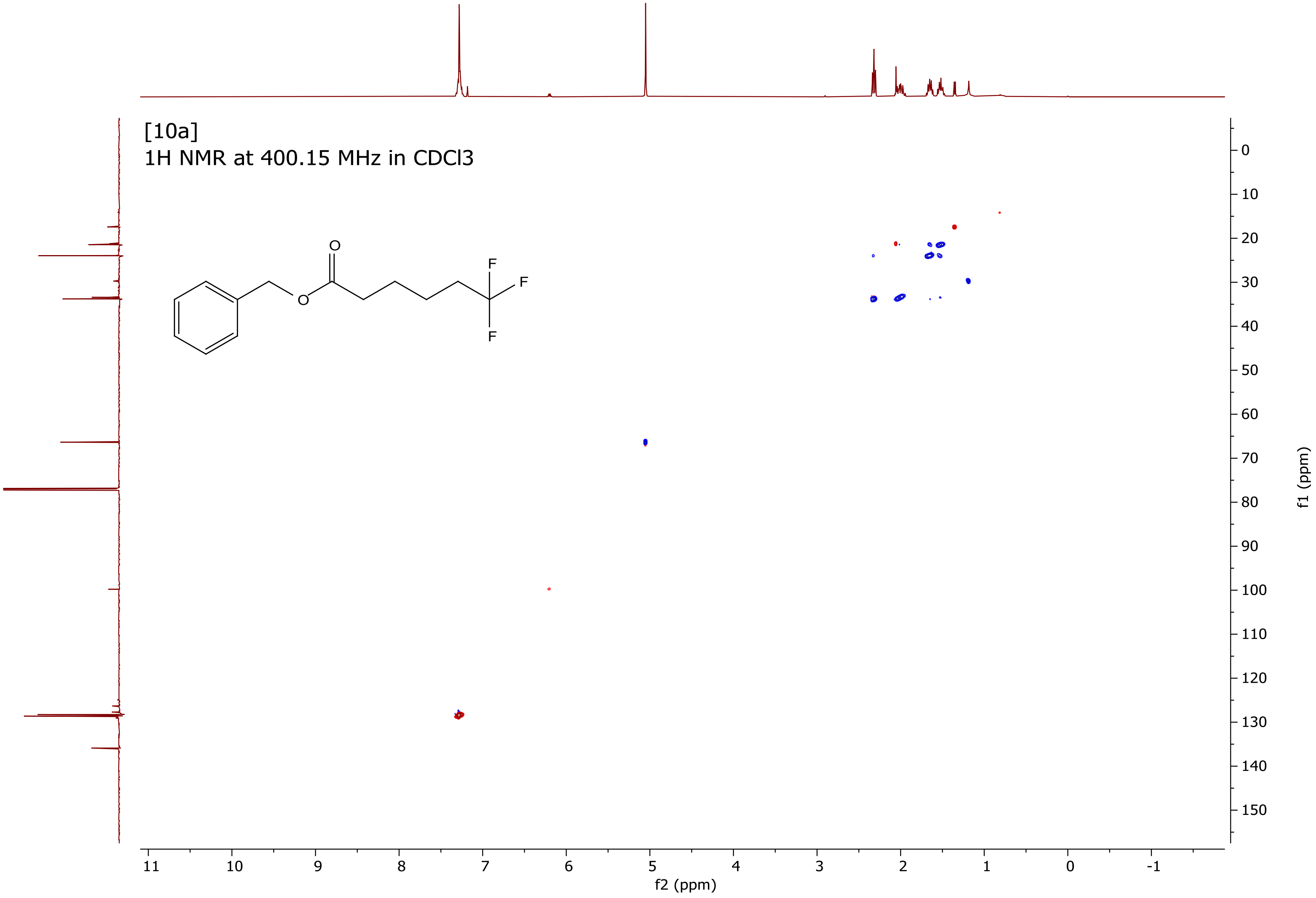
[10a]
1H NMR at 400.15 MHz in CDCl3
O
O
F
F
F
f2 (ppm)
f1 (ppm)

[10a]
19F NMR at 376.48 MHz in CDCl3

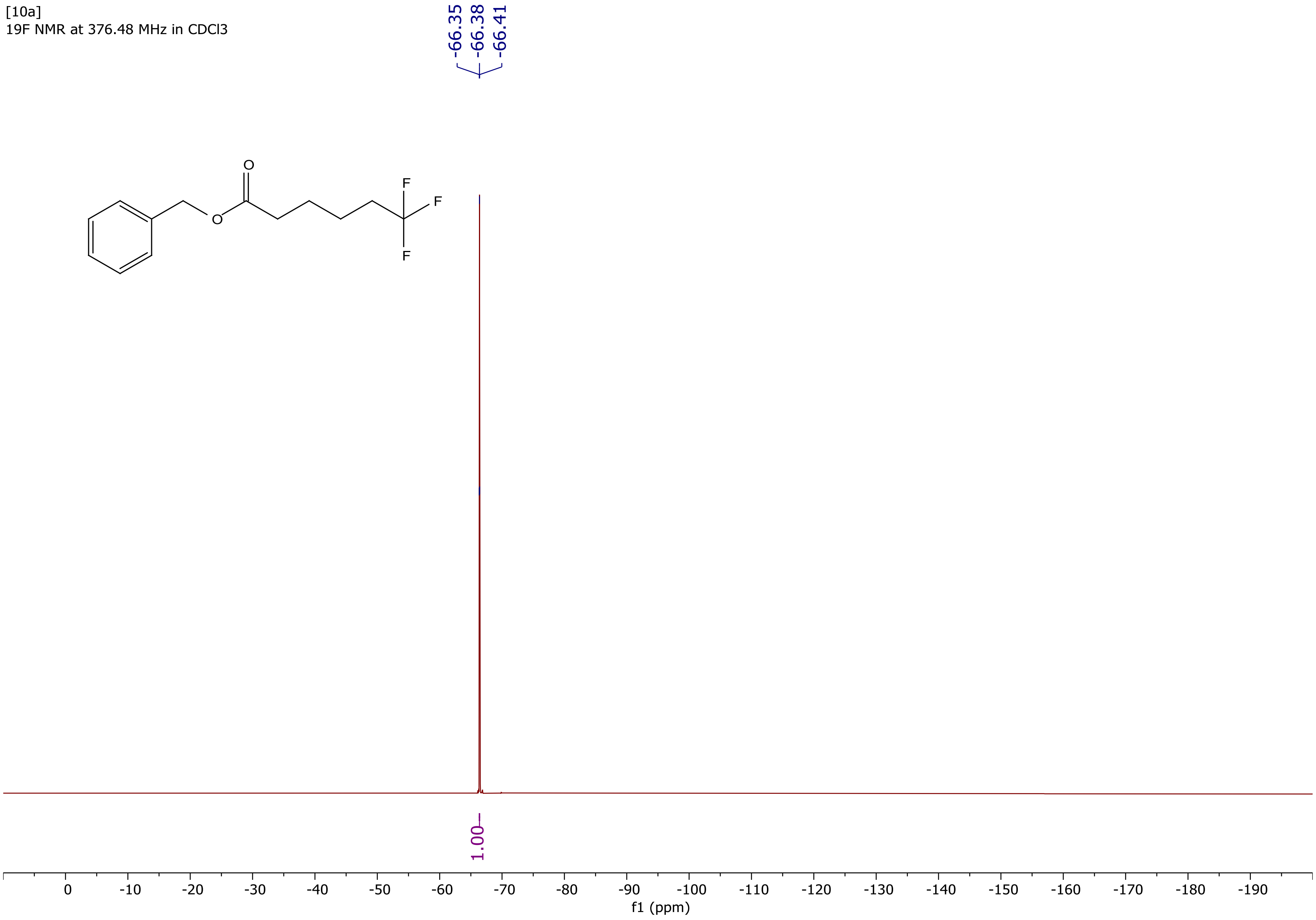

[11a]
1H NMR at 400.15 MHz in CDCl3

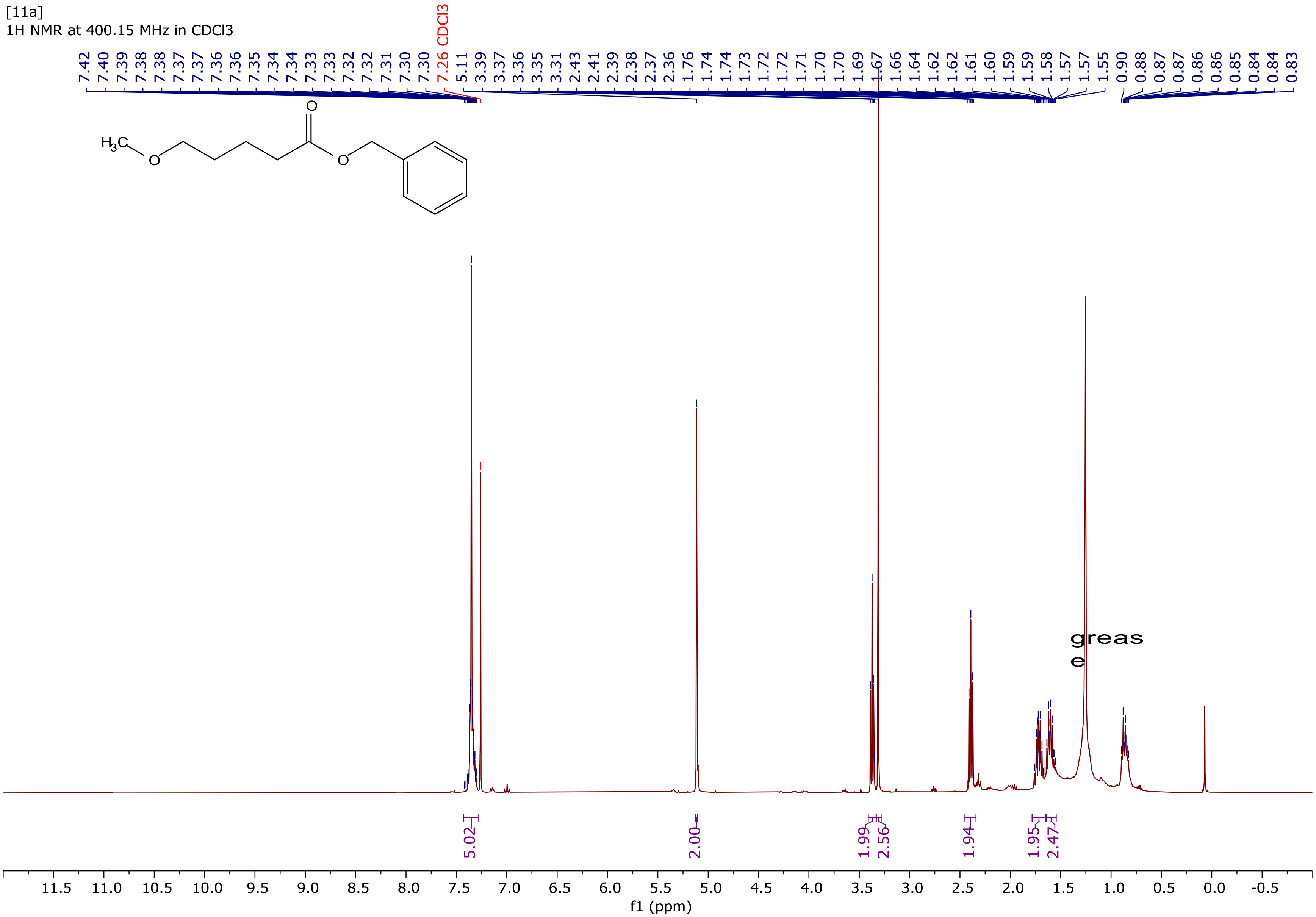

[11a]
13C NMR at 100.63 MHz in CDCl3

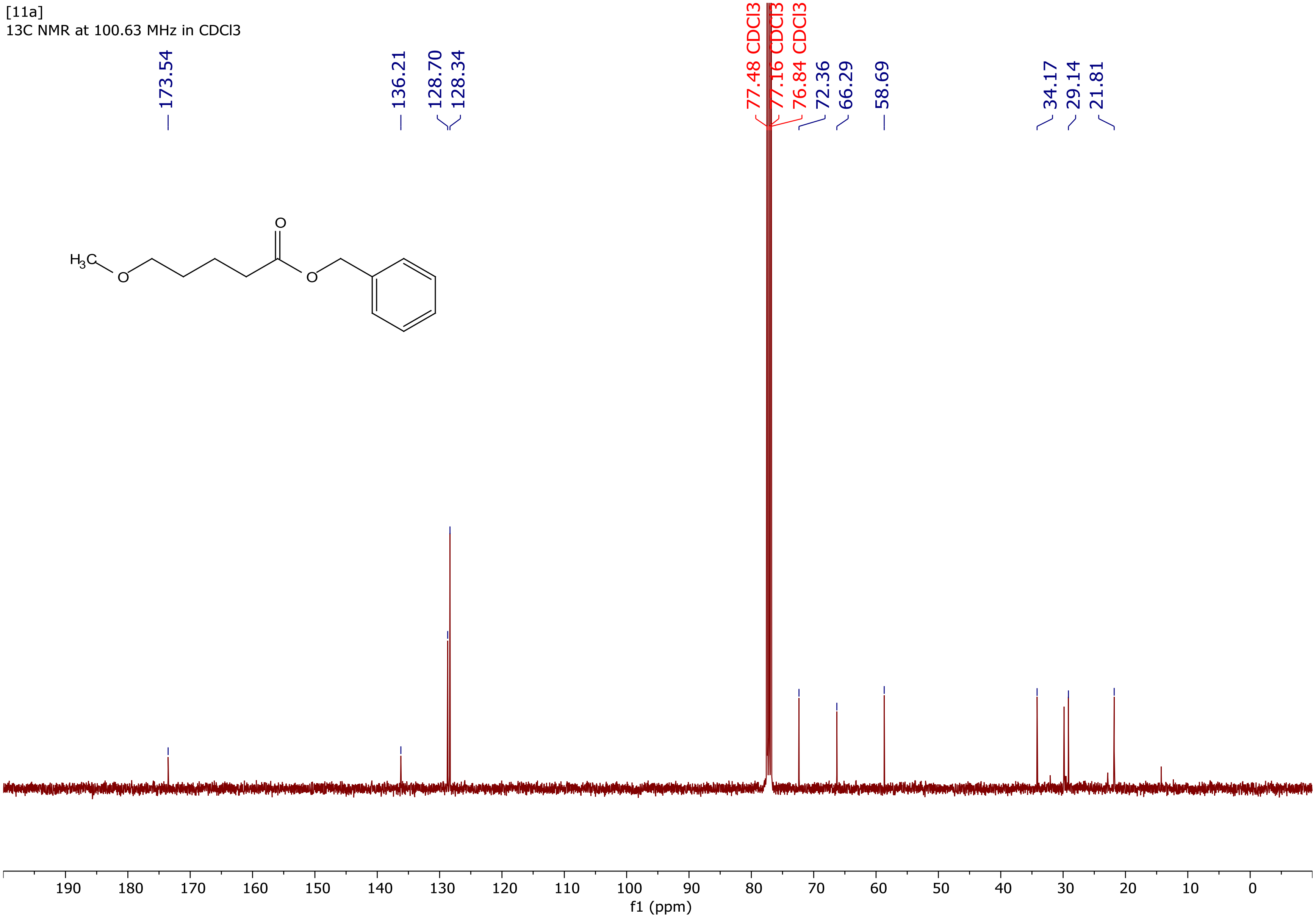

[12a]
1H NMR at 400.15 MHz in CDCl3

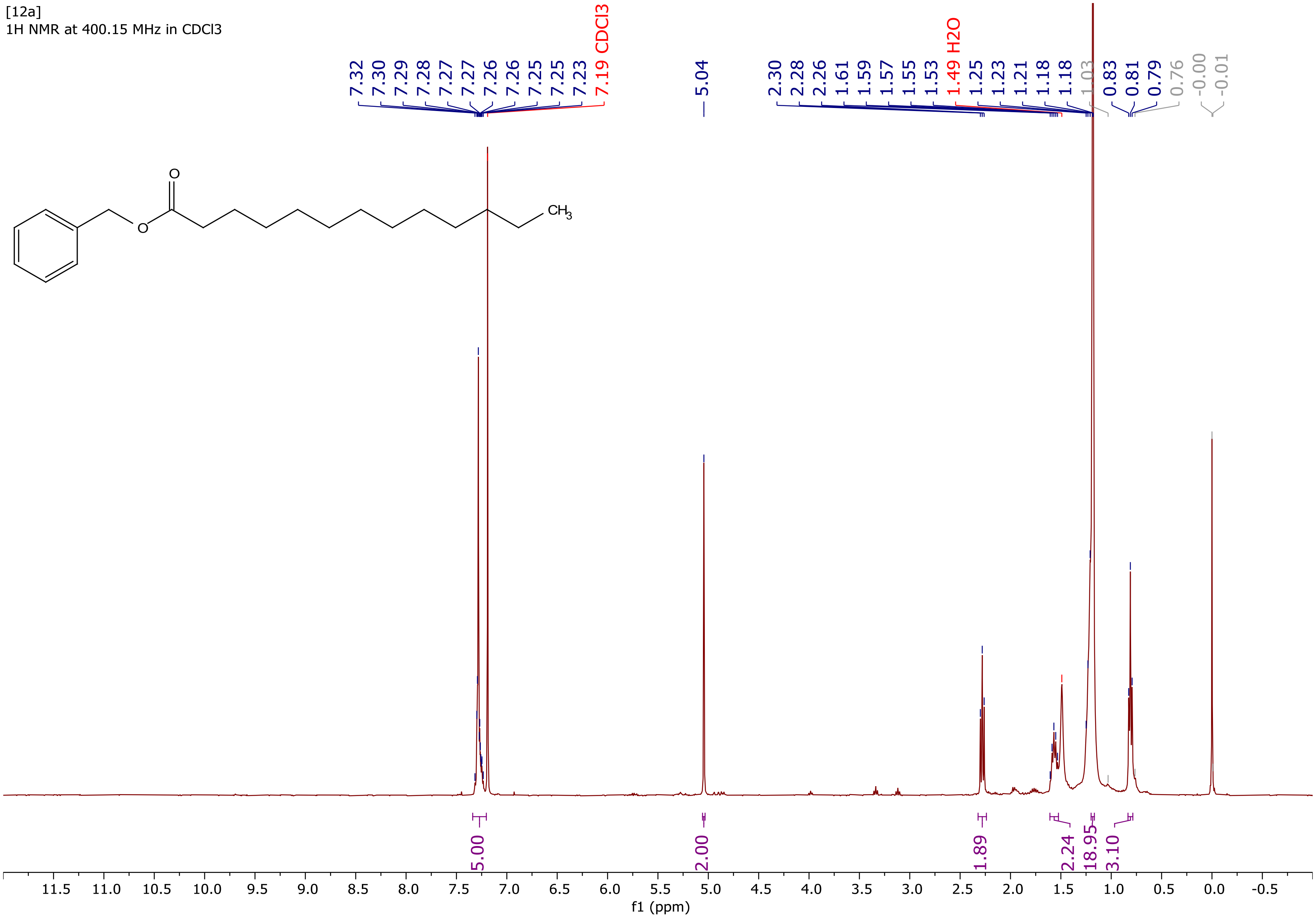

[12a]
13C NMR at 201.27 MHz in CDCl3

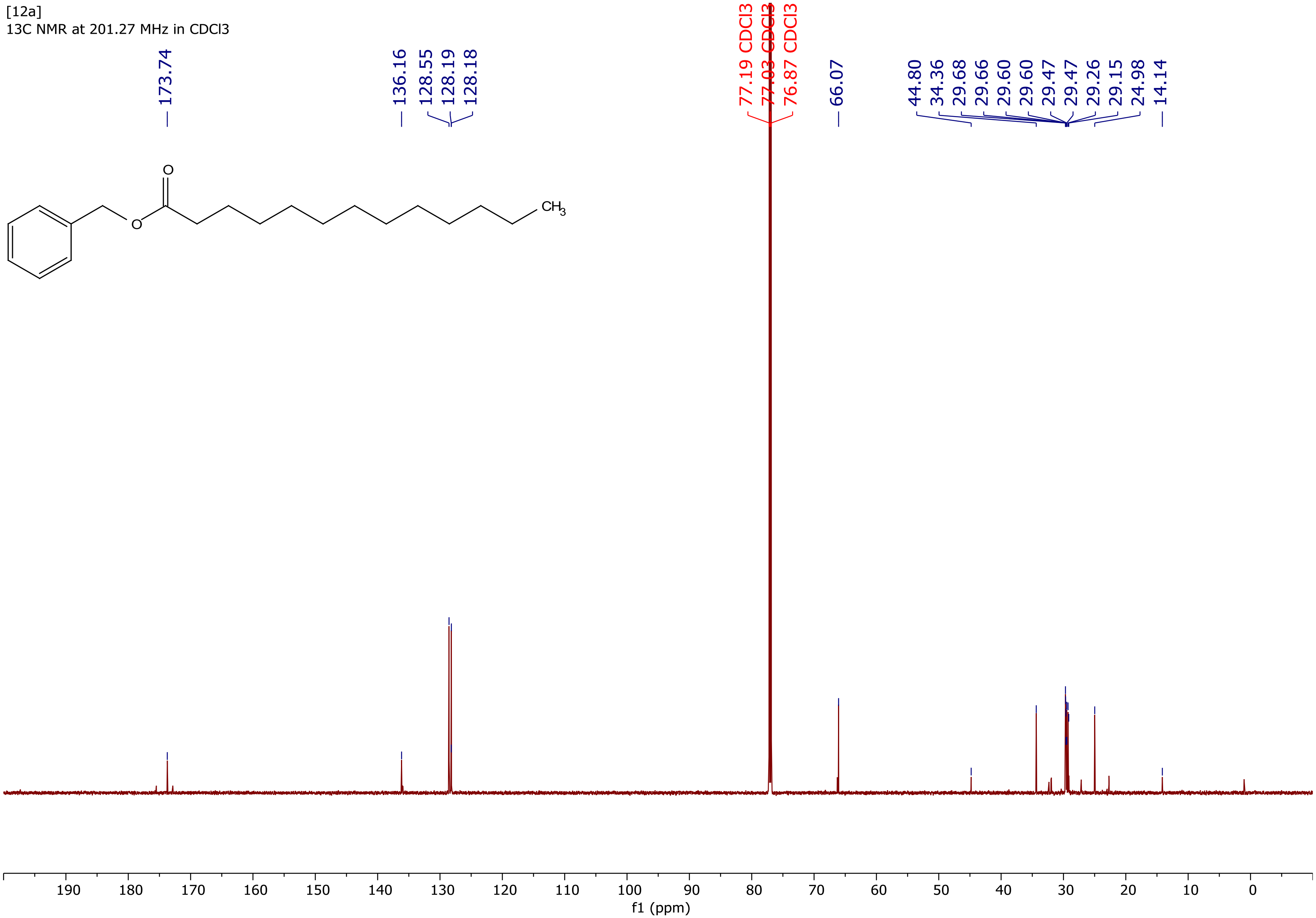

[13a]
1H NMR at 800.34 MHz in CDCl3

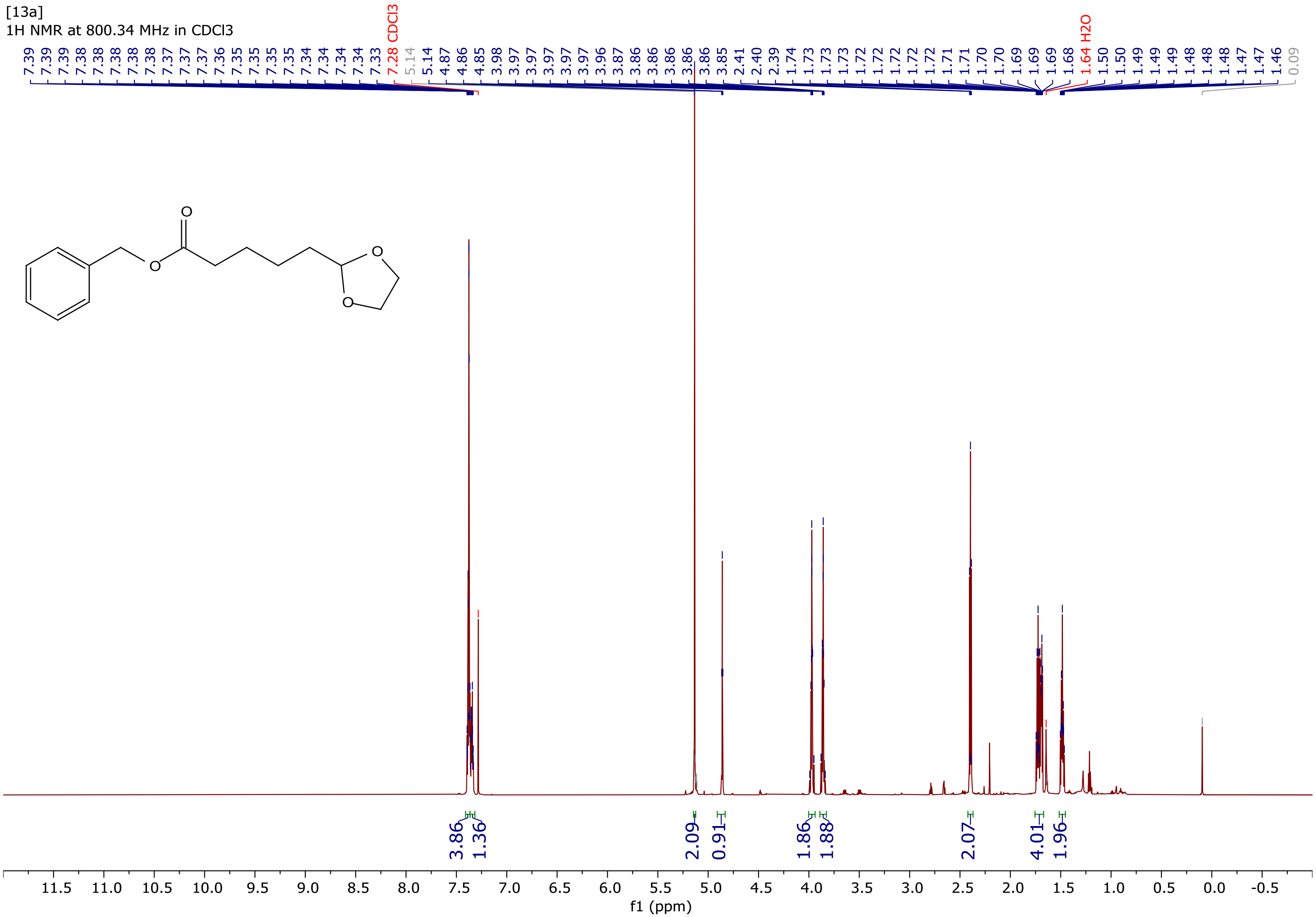

[13a]
13C NMR at 201.27 MHz in CDCl3

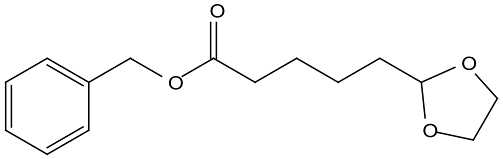

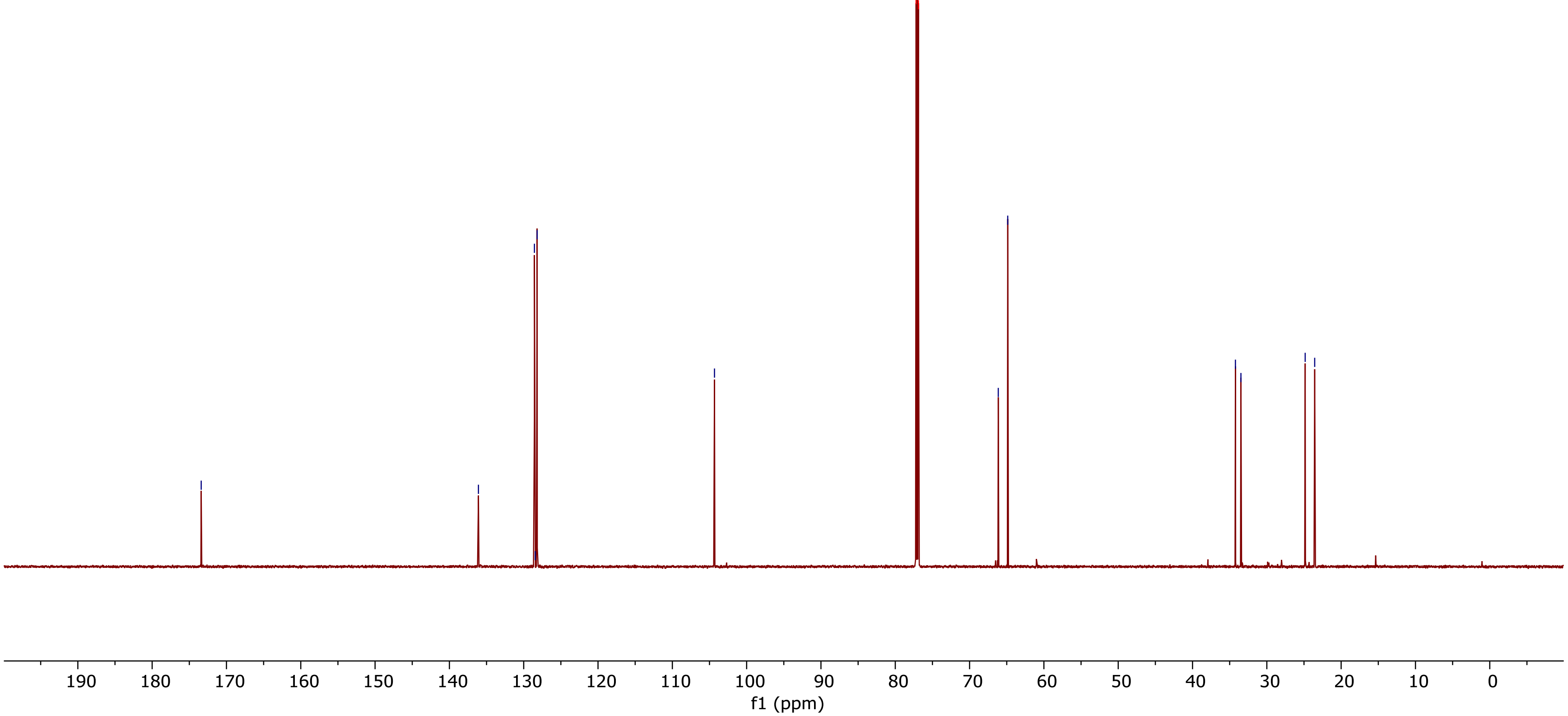

[14a]
1H NMR at 400.15 MHz in CDCl3

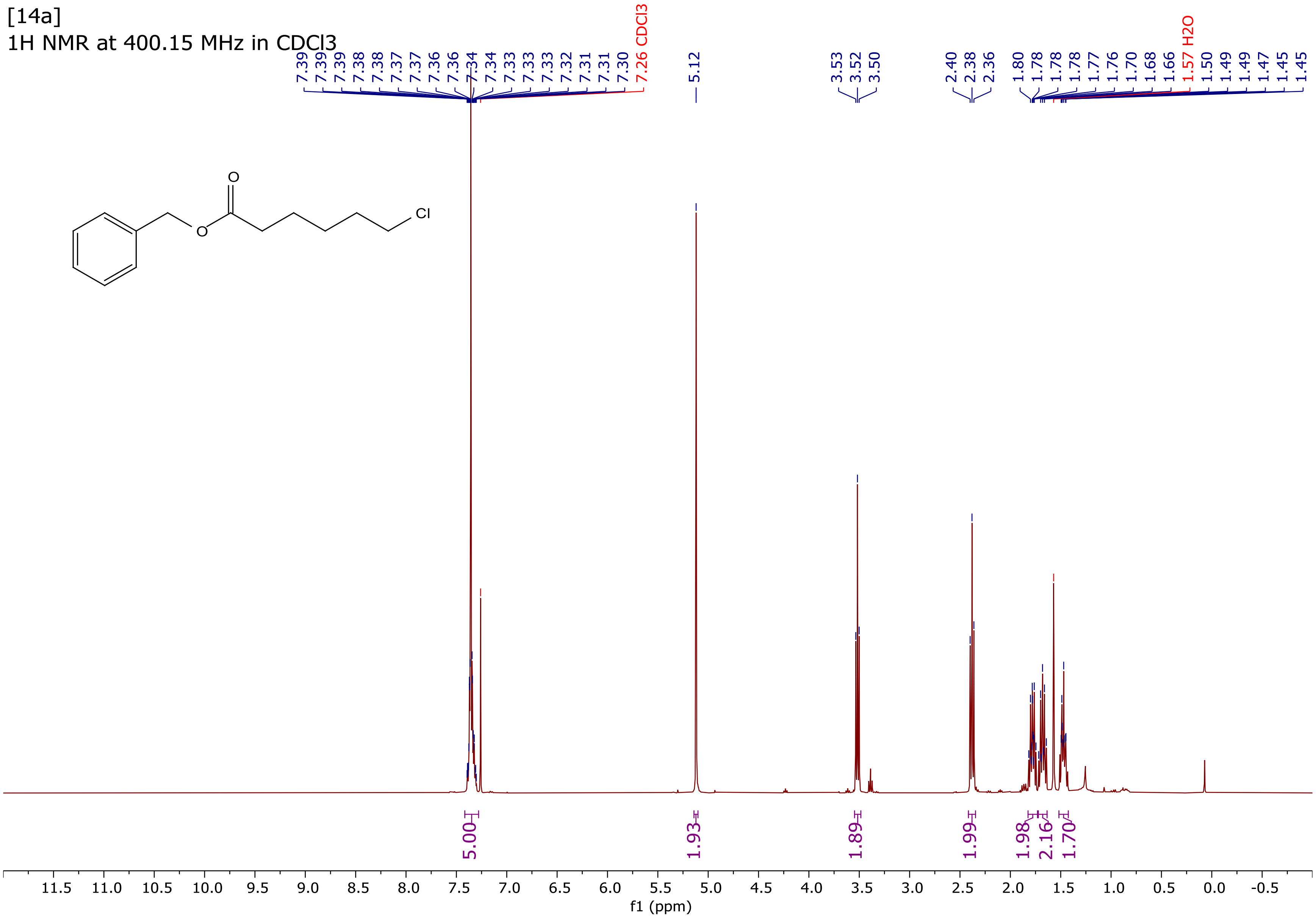

[14a]
13C NMR at 201.27 MHz in CDCl3

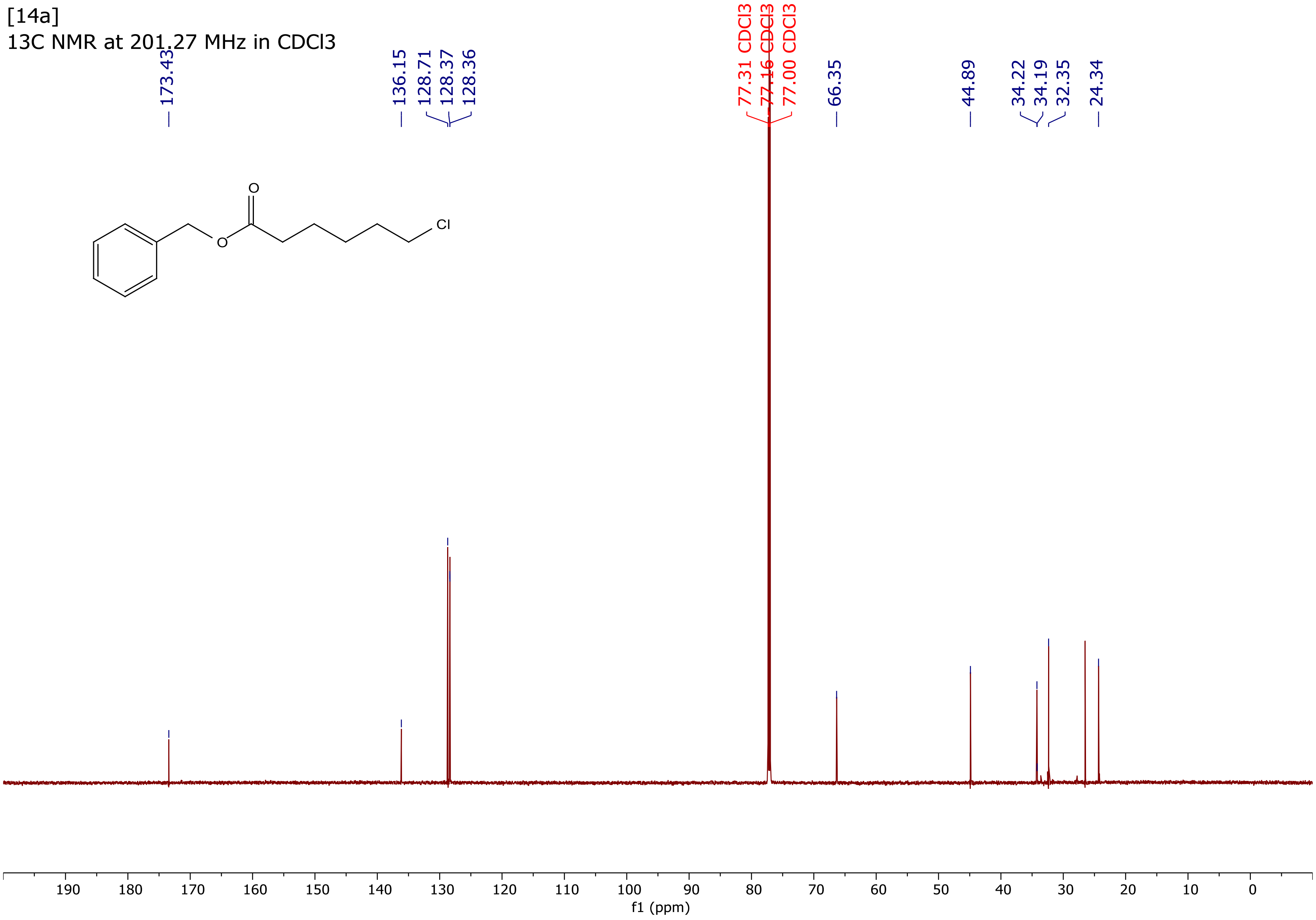

[15a]
1H NMR at 400.15 MHz in CDCl3

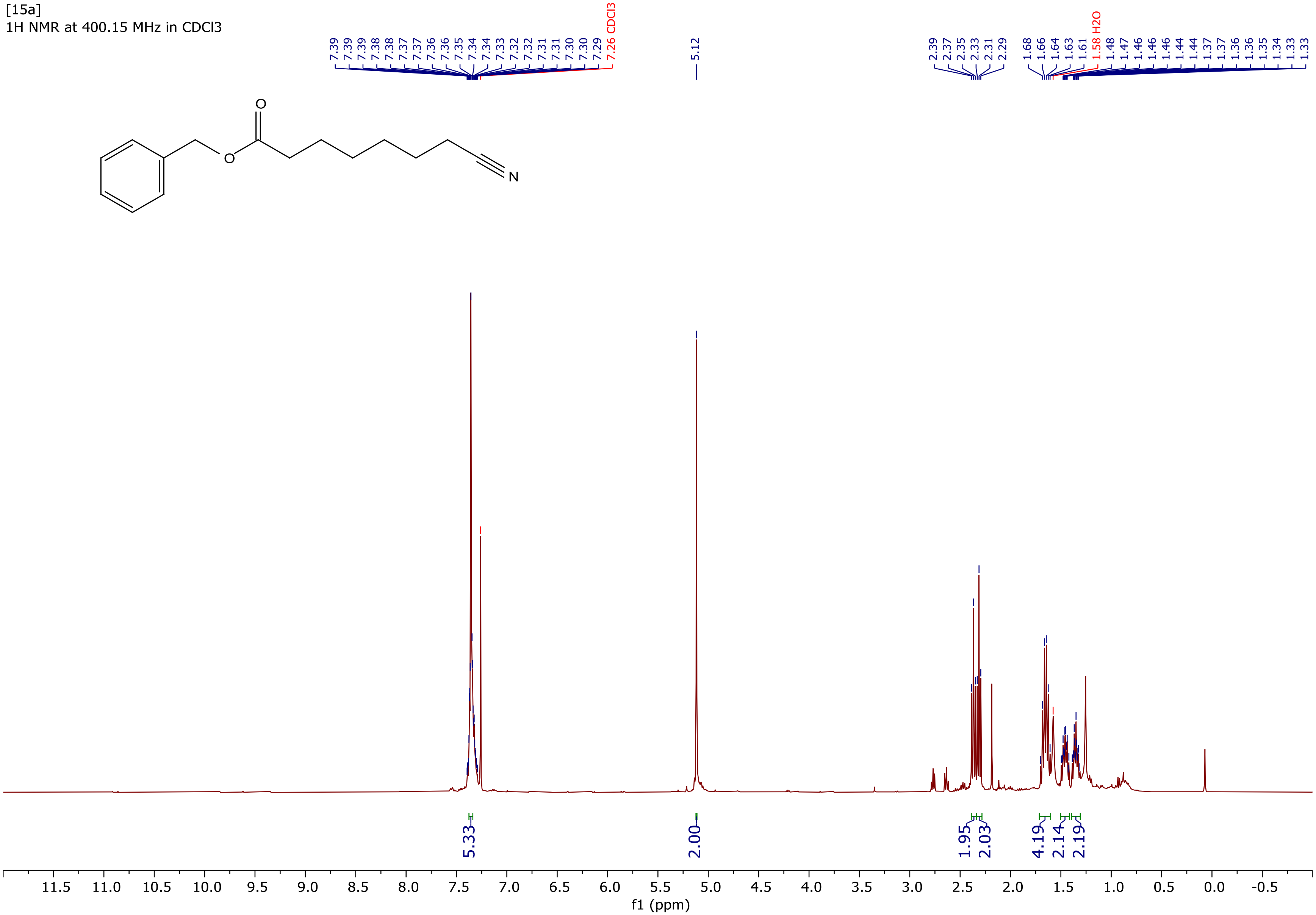

[15a]
13C NMR at 201.27 MHz in CDCl3

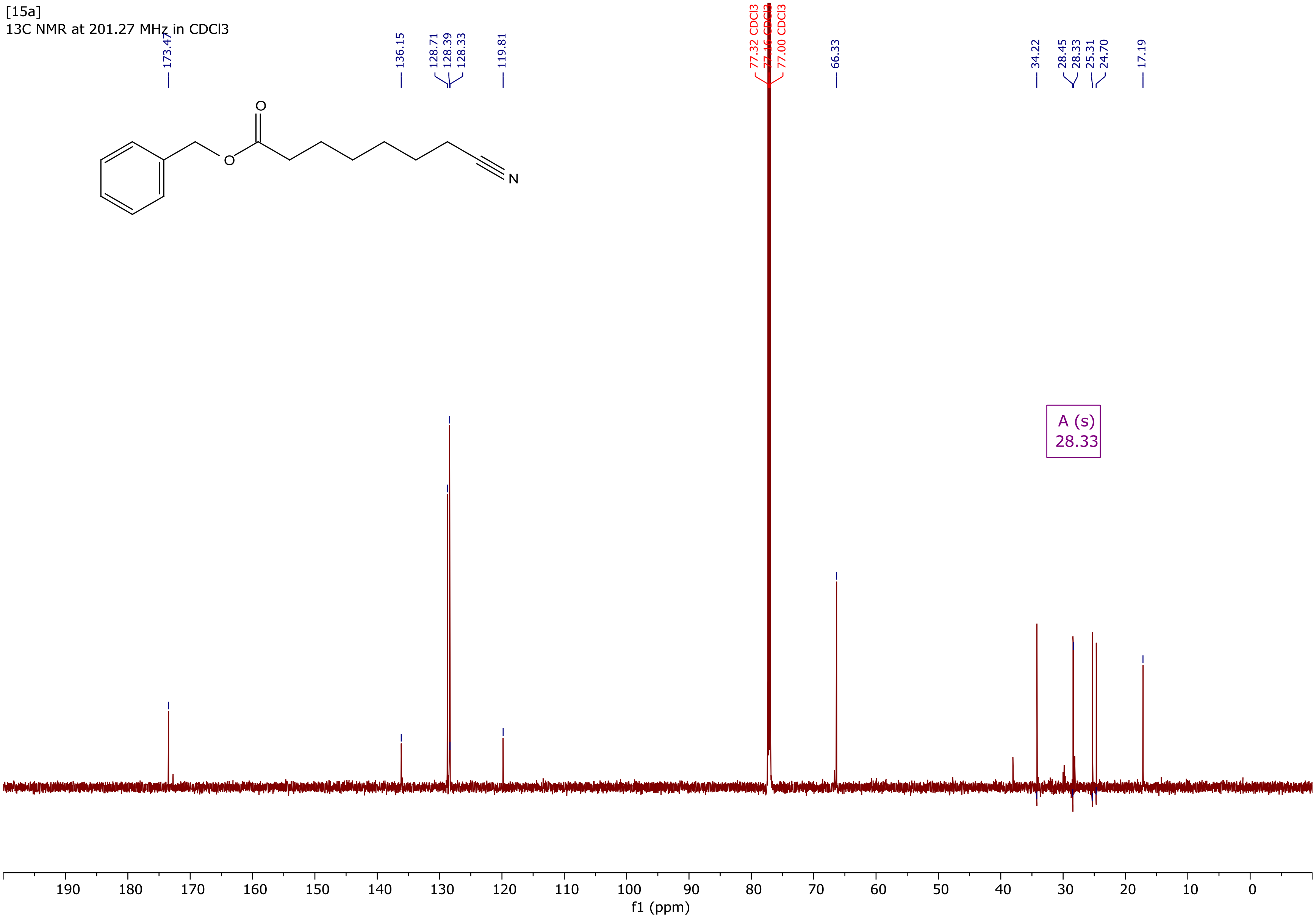

[16a]
1H NMR at 800.34 MHz in CDCl3

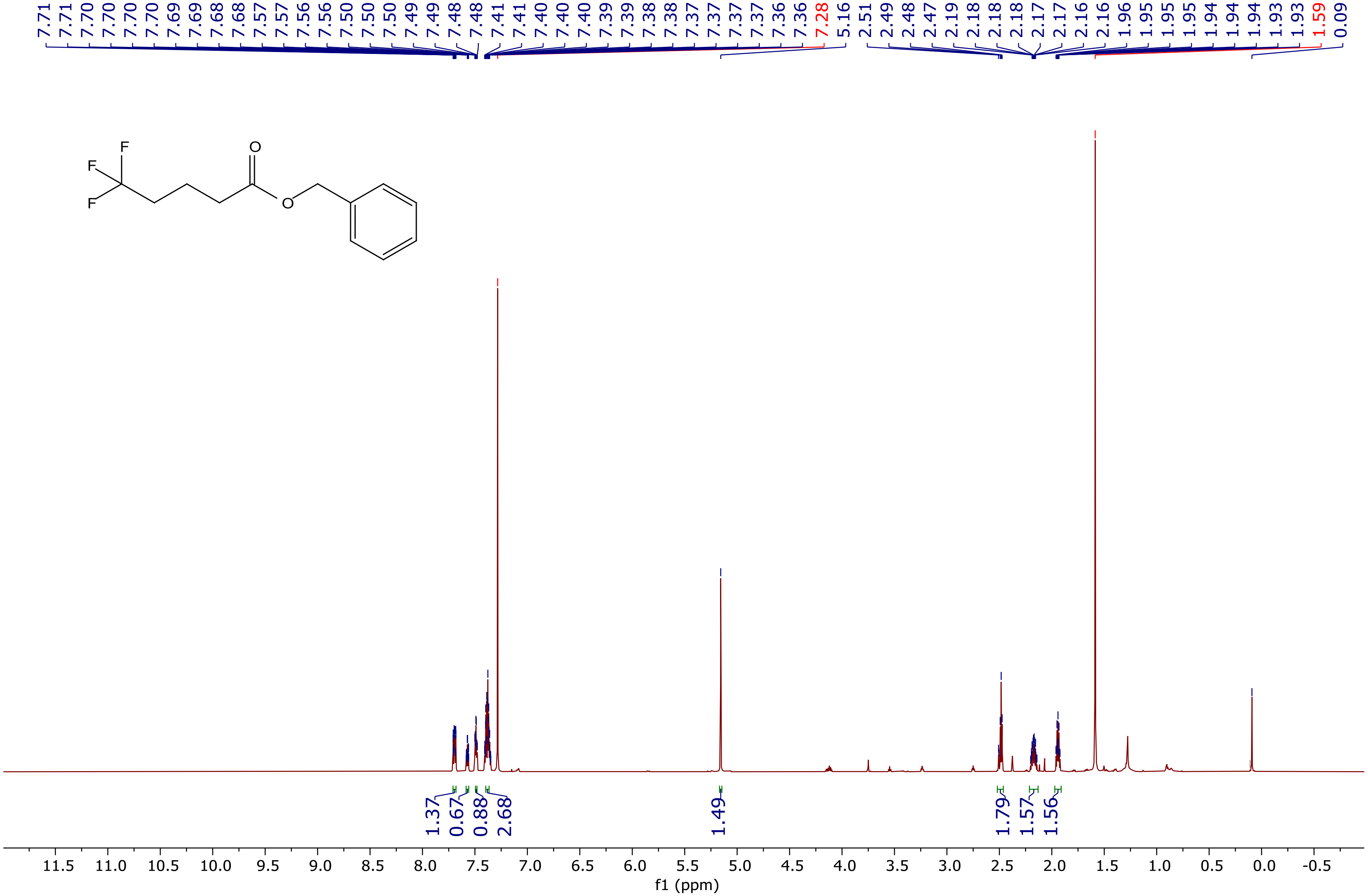

[16a]
13C NMR at 201.27 MHz in CDCl3

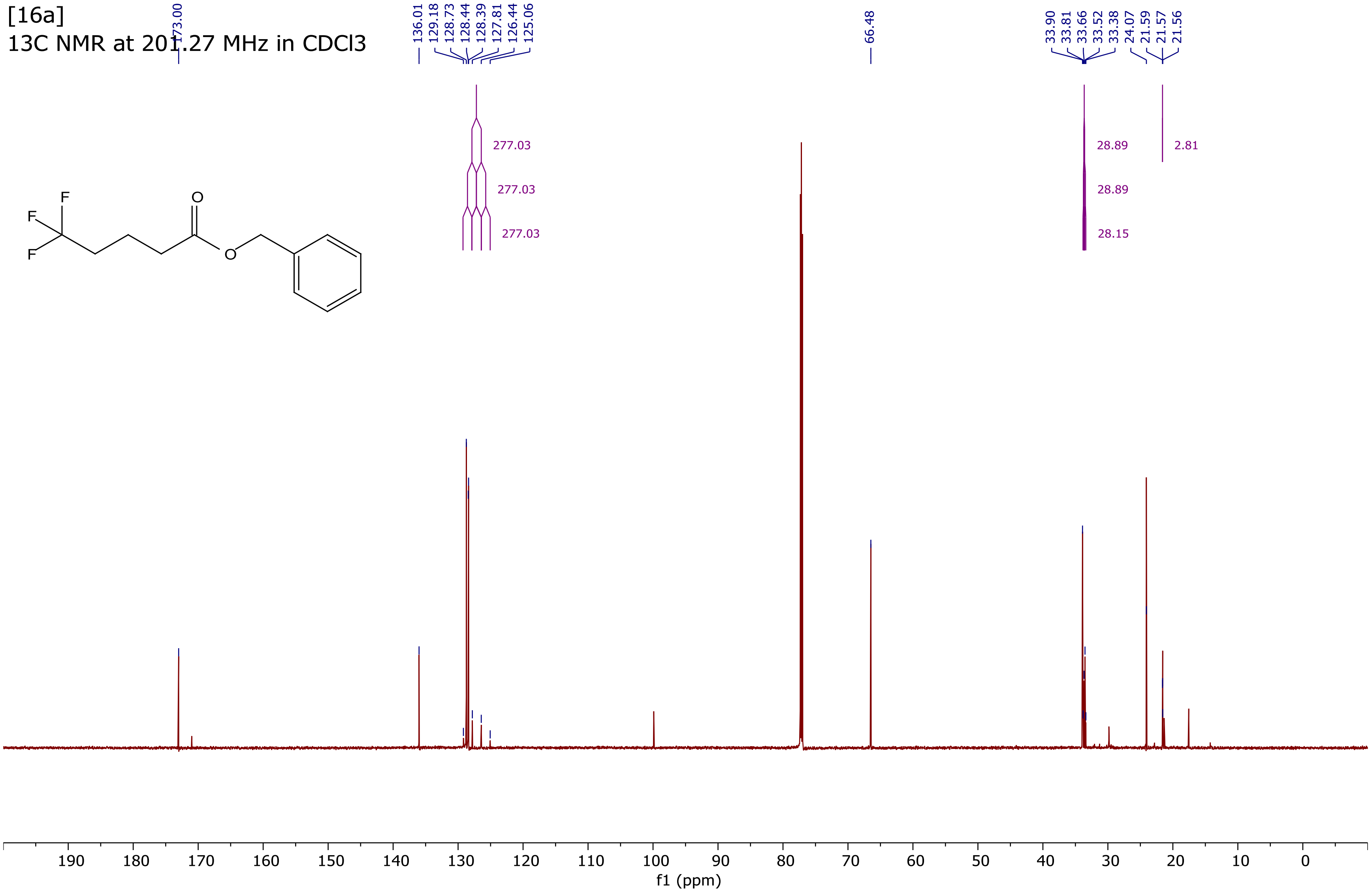

[16a]
19F NMR at 376.48 MHz in CDCl3

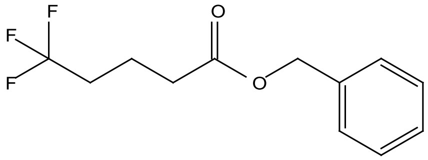

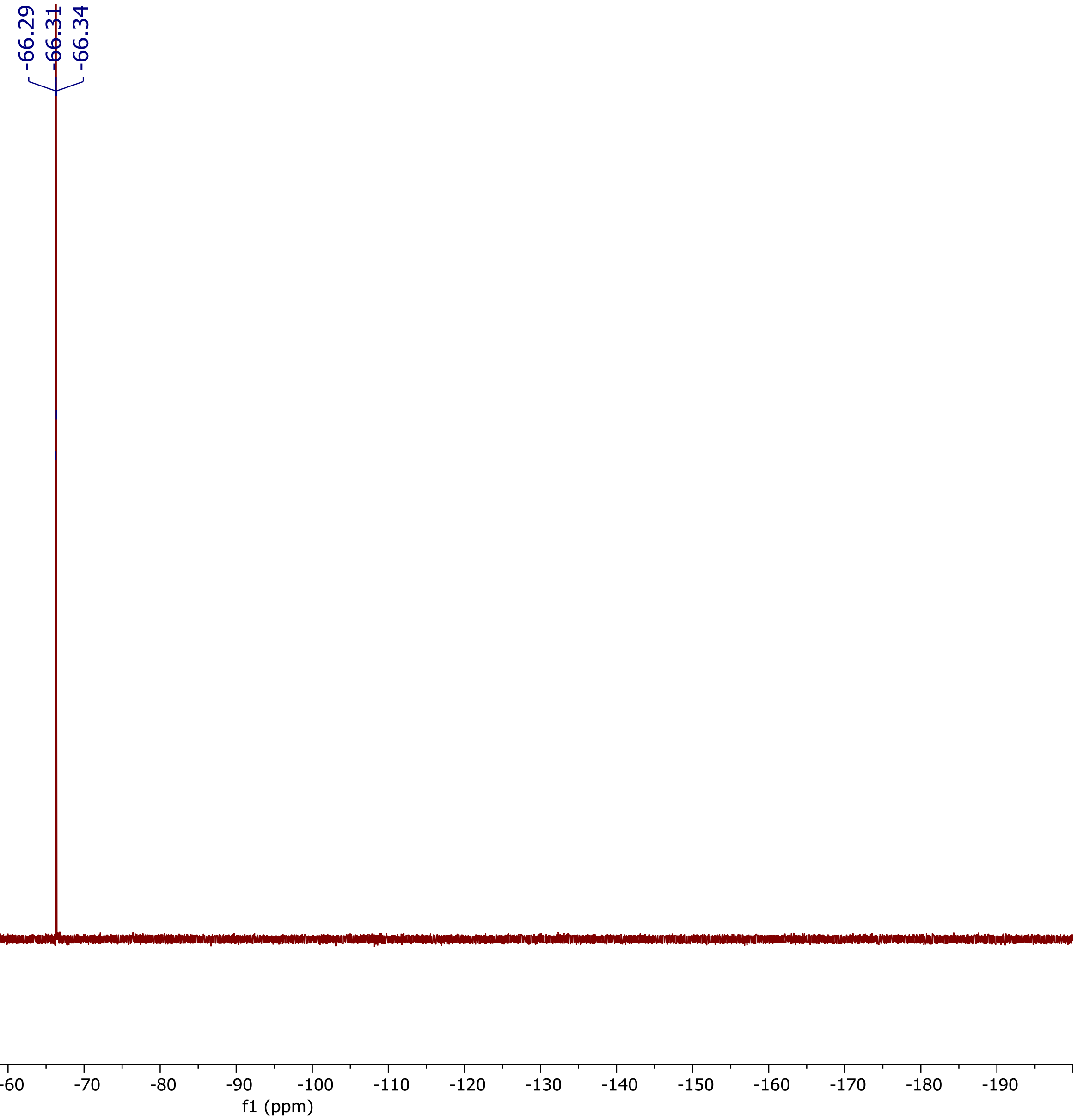

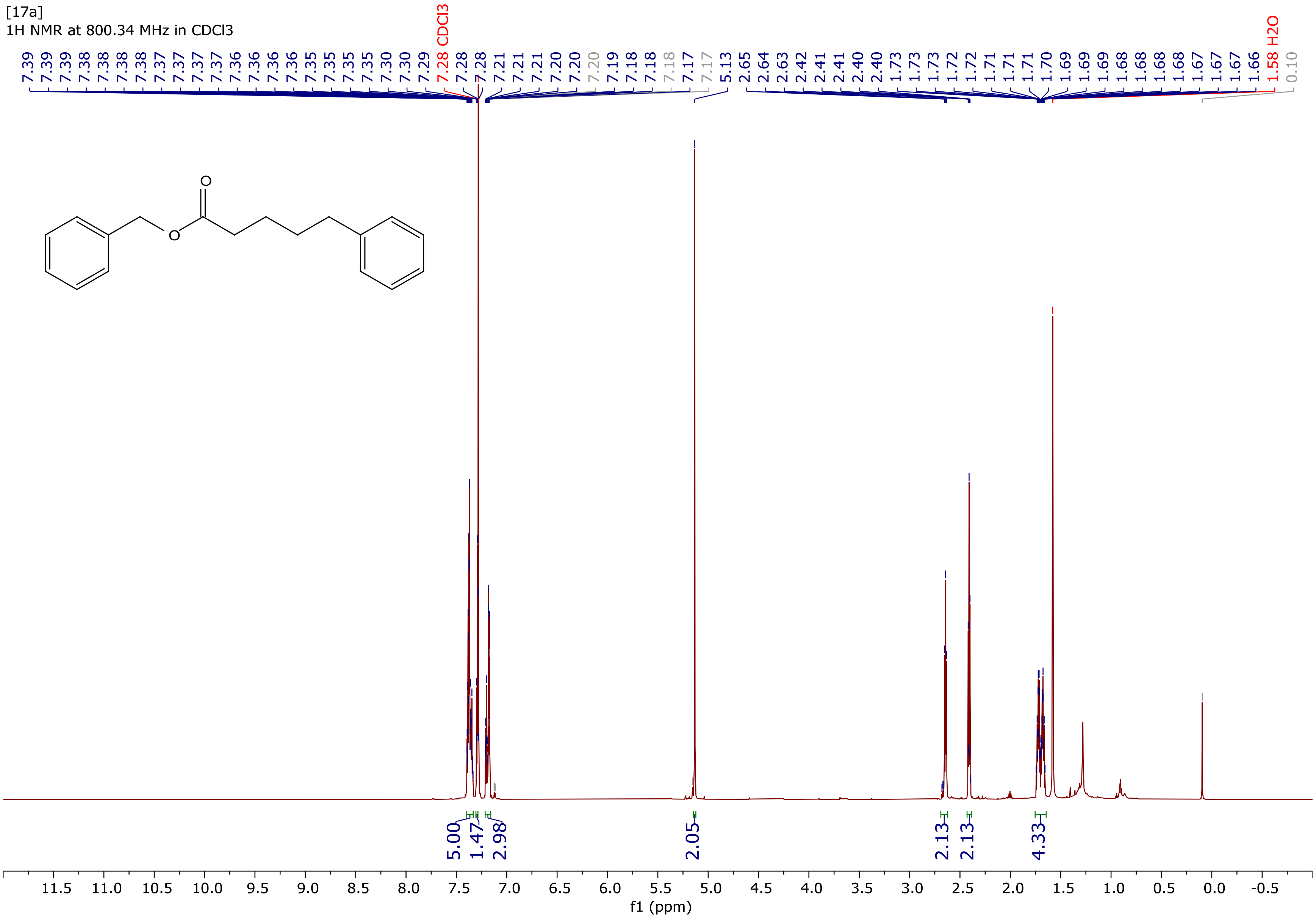
[17a]
1H NMR at 800.34 MHz in CDCl3
7.39 7.39 7.39 7.38 7.38 7.38 7.38 7.37 7.37 7.37 7.36 7.36 7.36 7.35 7.35 7.35 7.35 7.30 7.30 7.29 7.28 CDCl3 7.28 7.28 7.21 7.21 7.21 7.20 7.20 7.20 7.19 7.18 7.18 7.18 7.17 7.17 5.13 2.65 2.64 2.63 2.42 2.41 2.41 2.40 2.40 1.73 1.73 1.73 1.72 1.72 1.71 1.71 1.71 1.70 1.69 1.69 1.69 1.68 1.68 1.68 1.68 1.67 1.67 1.67 1.66 1.58 H2O 0.10
O
O
5.00 1.47 2.98 2.05 2.13 2.13 4.33
11.5 11.0 10.5 10.0 9.5 9.0 8.5 8.0 7.5 7.0 6.5 6.0 5.5 5.0 4.5 4.0 3.5 3.0 2.5 2.0 1.5 1.0 0.5 0.0 -0.5
f1 (ppm)

[17a]
13C NMR at 201.27 MHz in CDCl3

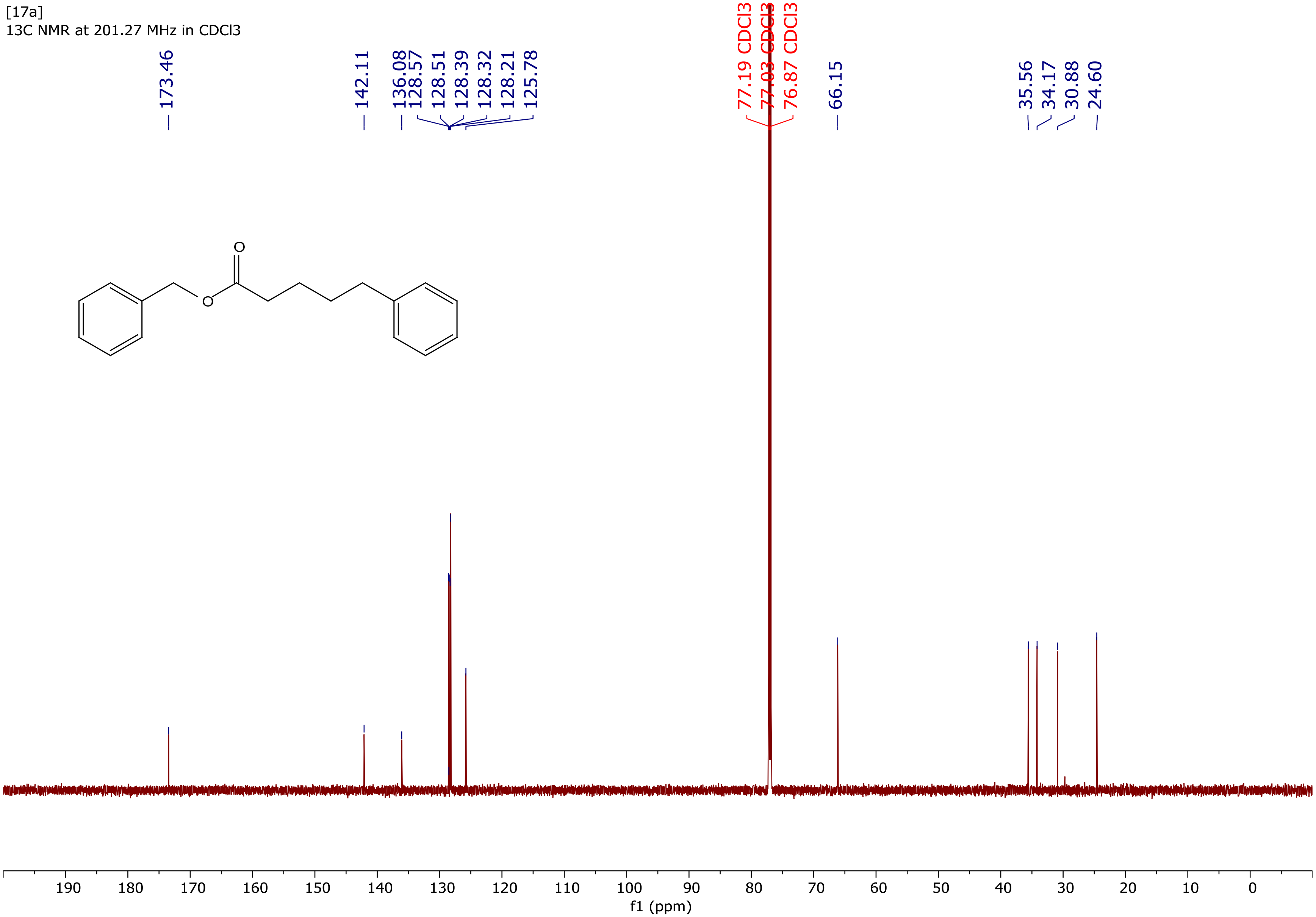

[18a]
1H NMR at 800.34 MHz in CDCl3

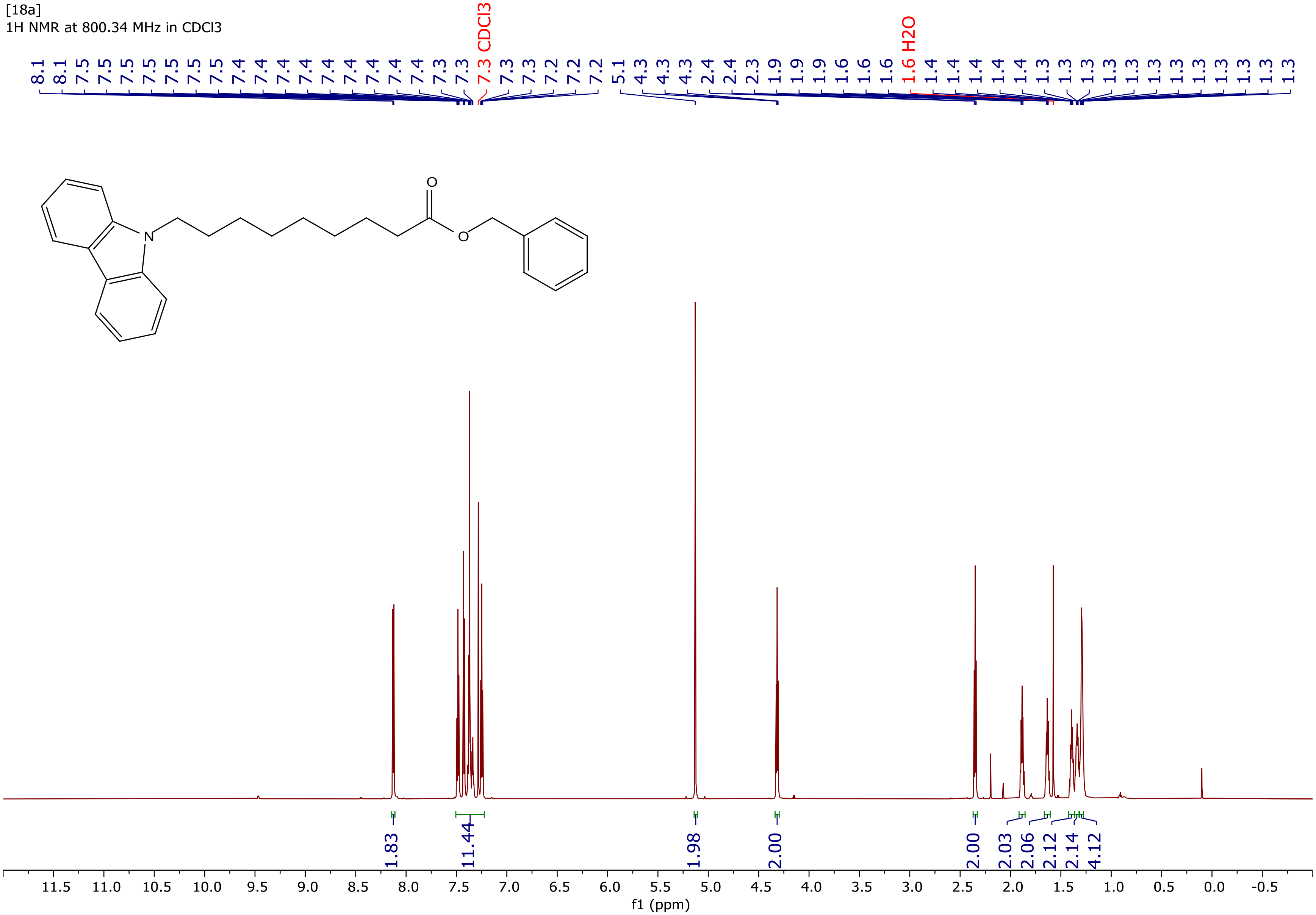

[18a]
13C NMR at 201.27 MHz in CDCl3

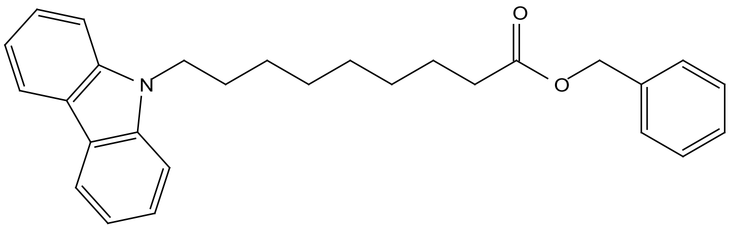

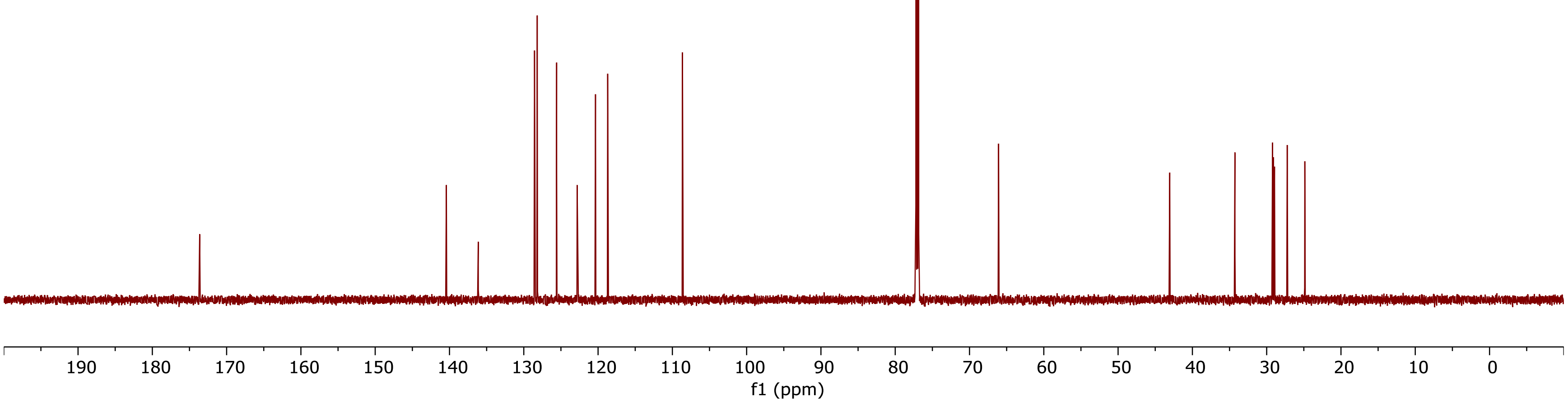

[19a]
1H NMR at 400.15 MHz in CDCl3

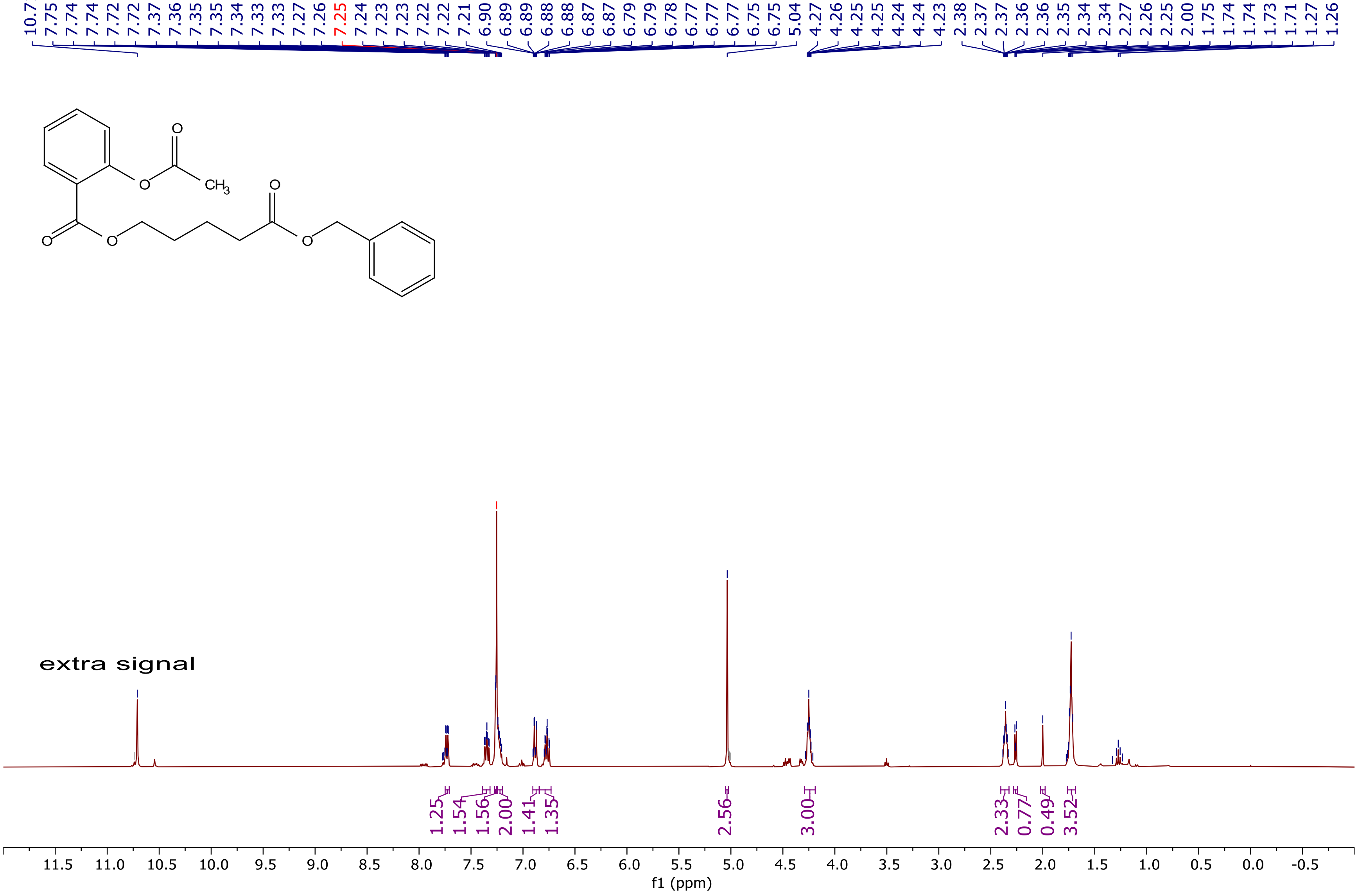

[19a]
13C NMR at 201.27 MHz in CDCl3

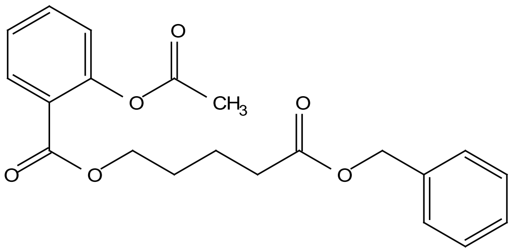

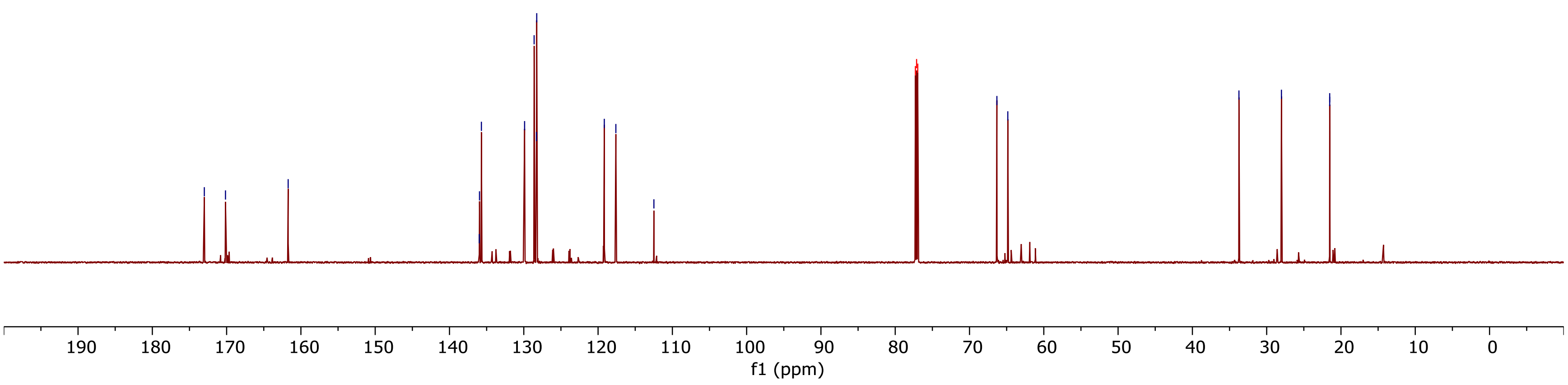

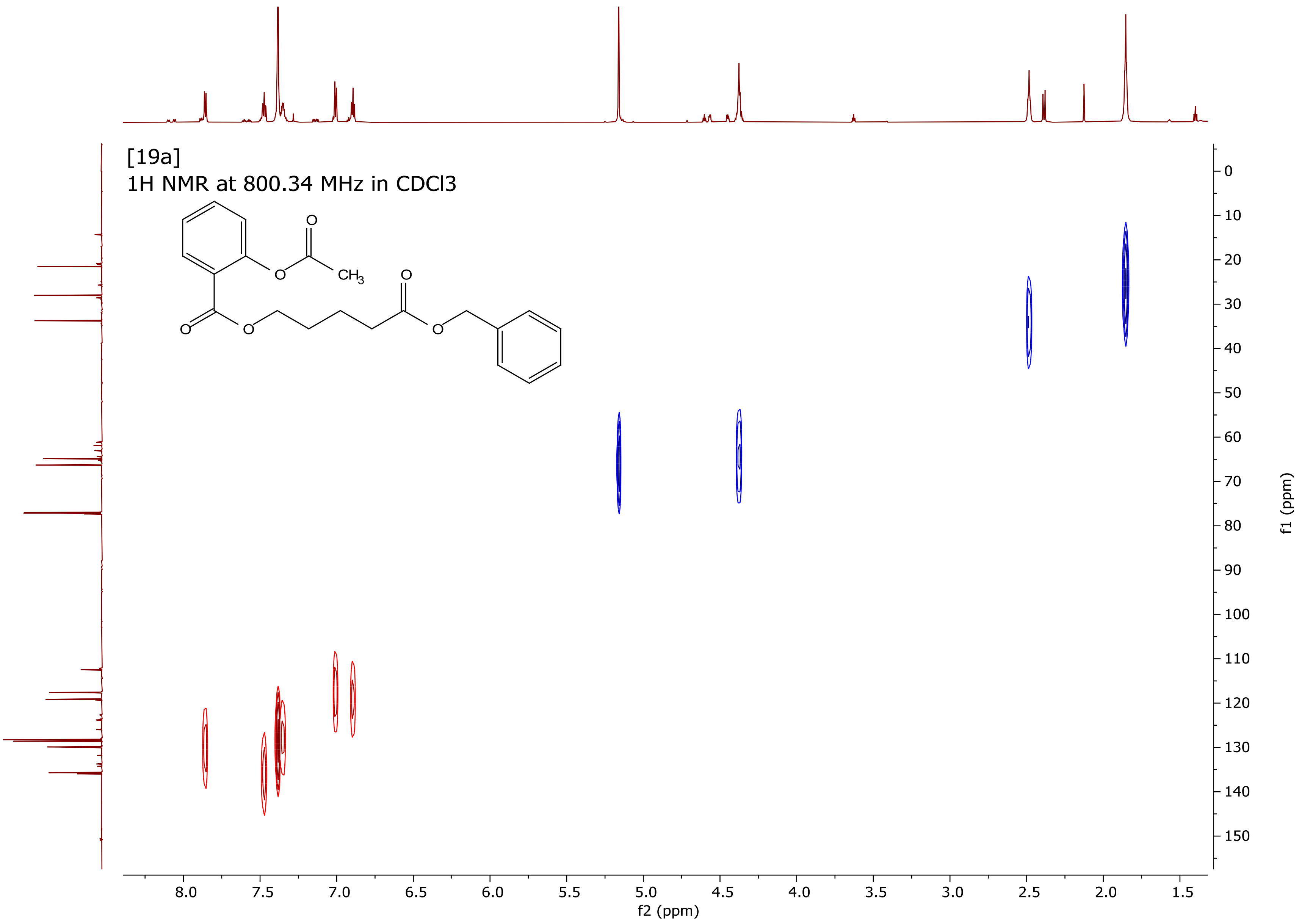

[19a]
1H NMR at 800.34 MHz in CDCl3
O
O
CH3
O
O
O
O
f2 (ppm)
f1 (ppm)
8.0
7.5
7.0
6.5
6.0
5.5
5.0
4.5
4.0
3.5
3.0
2.5
2.0
1.5
0
10
20
30
40
50
60
70
80
90
100
110
120
130
140
150

[20a]
1H NMR at 800.34 MHz in CDCl3

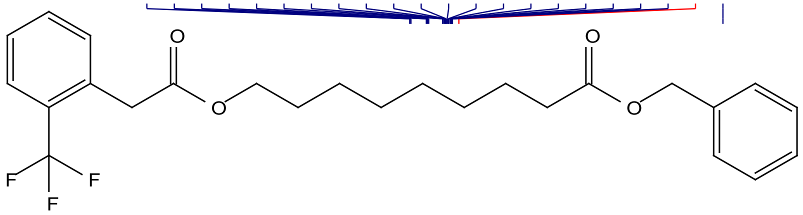

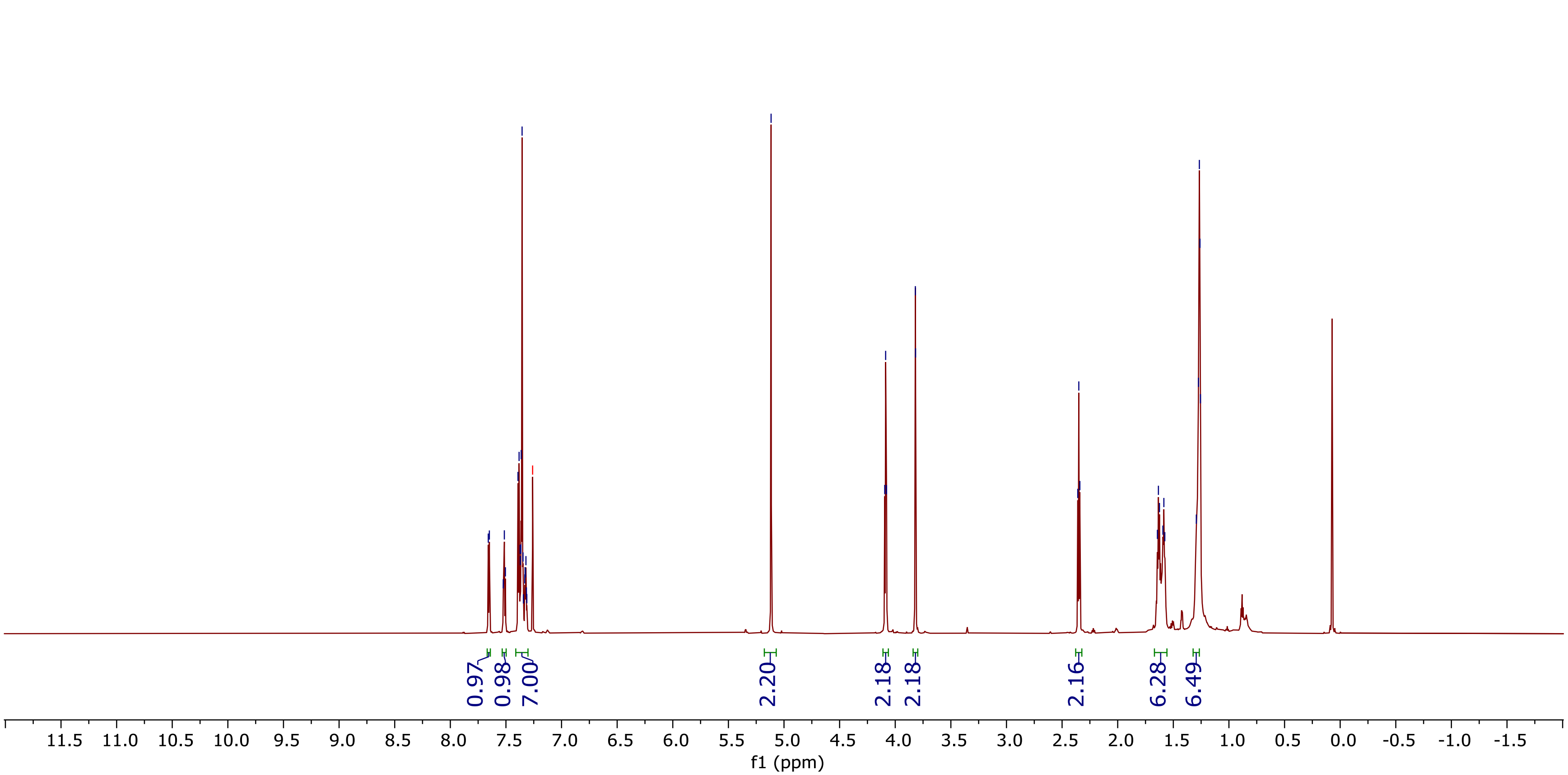

[20a]
13C NMR at 201.27 MHz in CDCl3

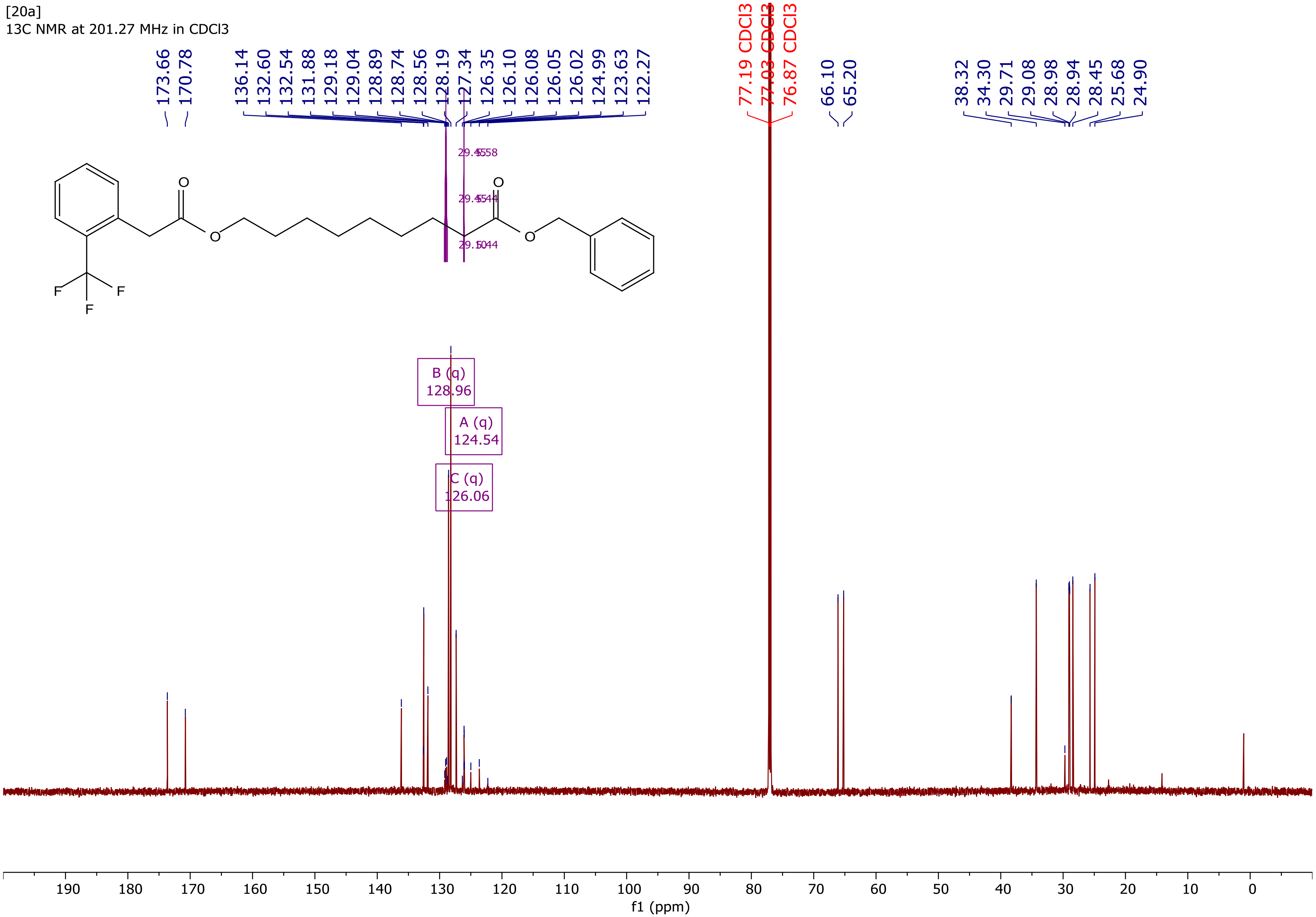

[20a]
19F NMR at 753.00 MHz in CDCl3

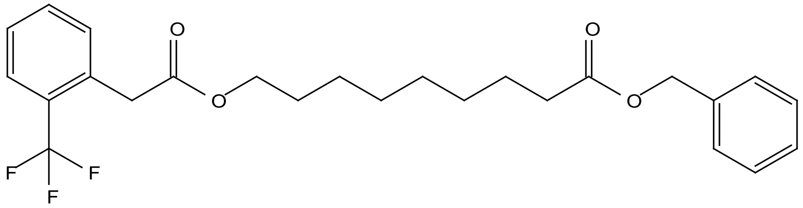

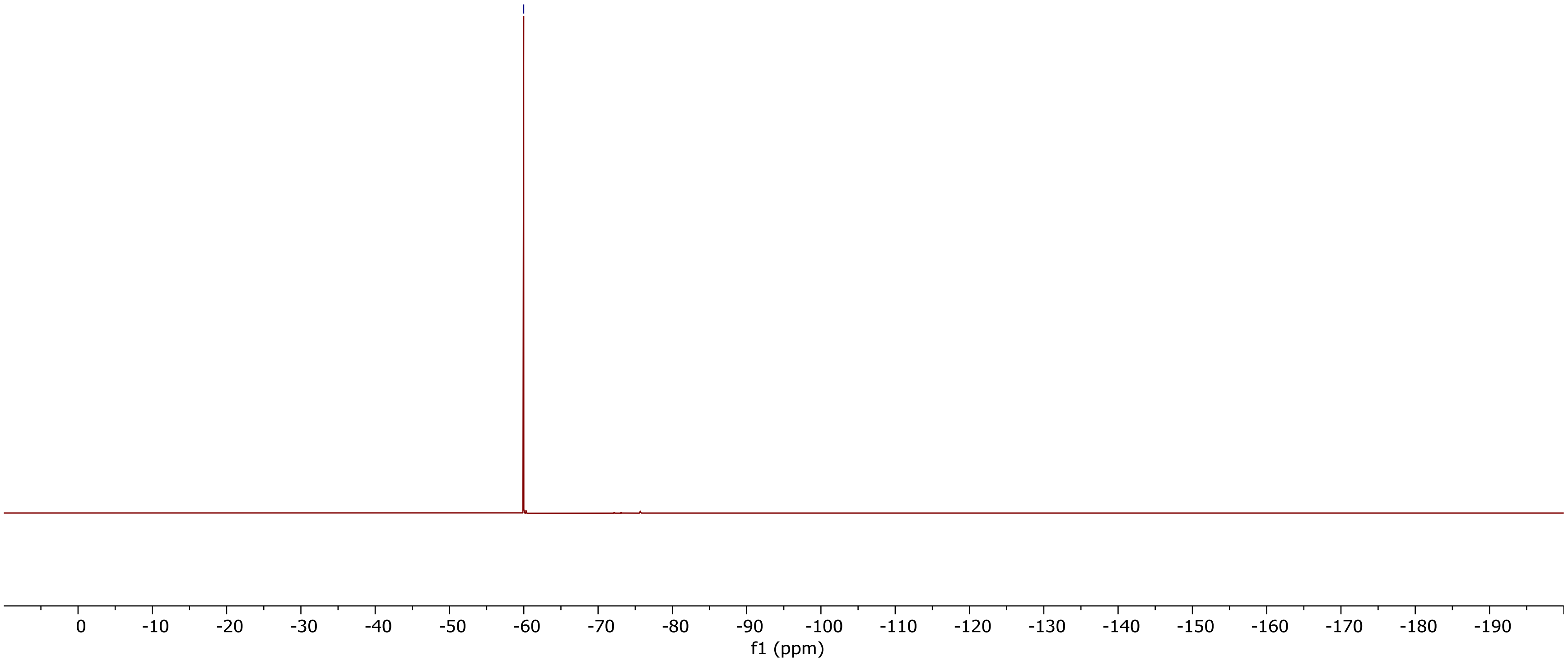

[21a]

1H NMR at 400.15 MHz in CDCl3

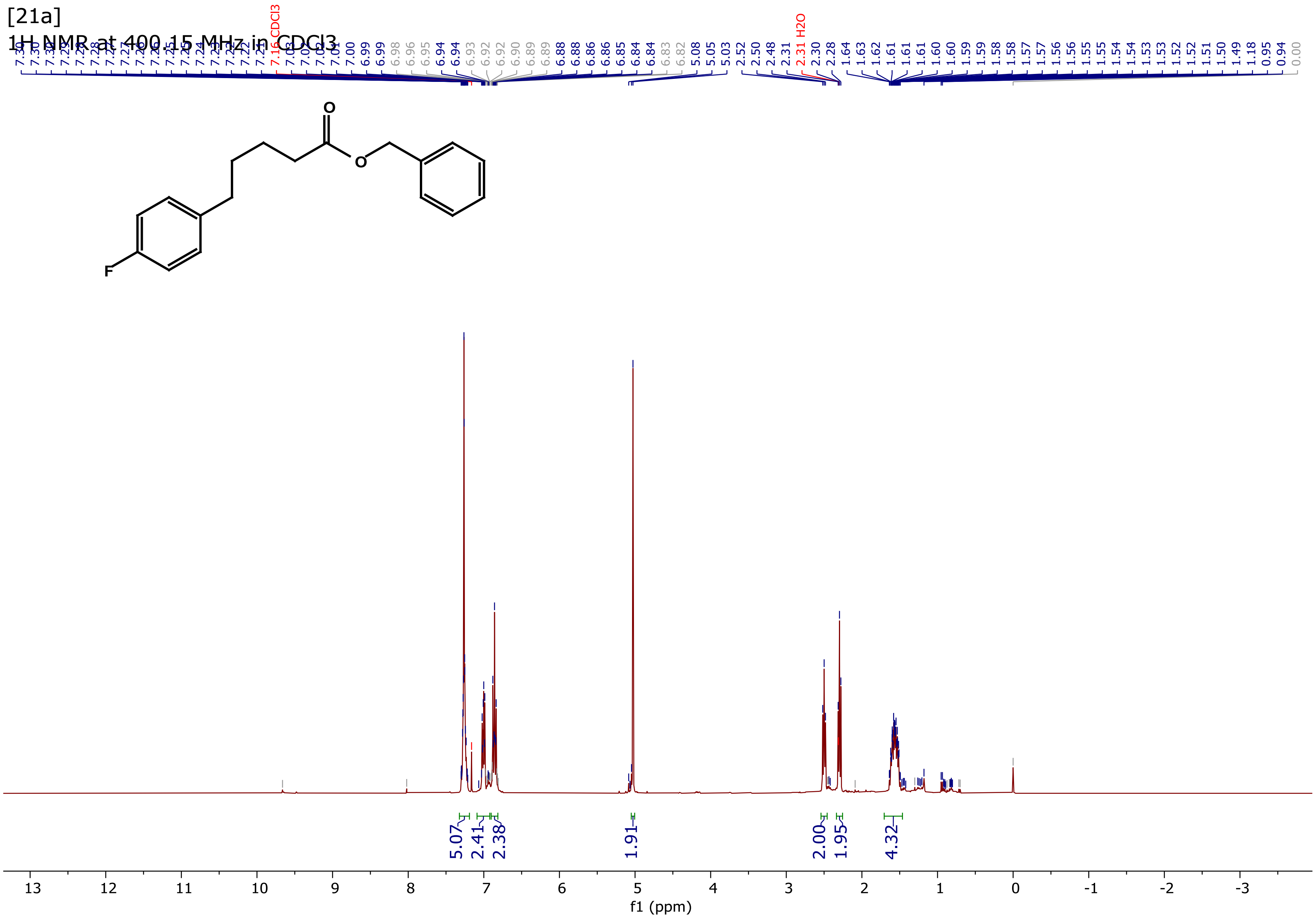

[21a]
19F NMR at 376.48 MHz in CDCl3

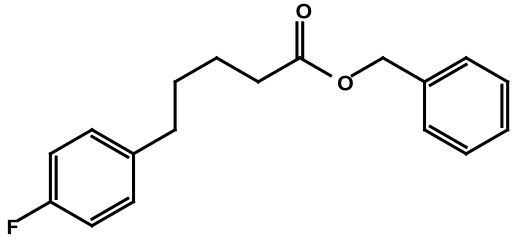

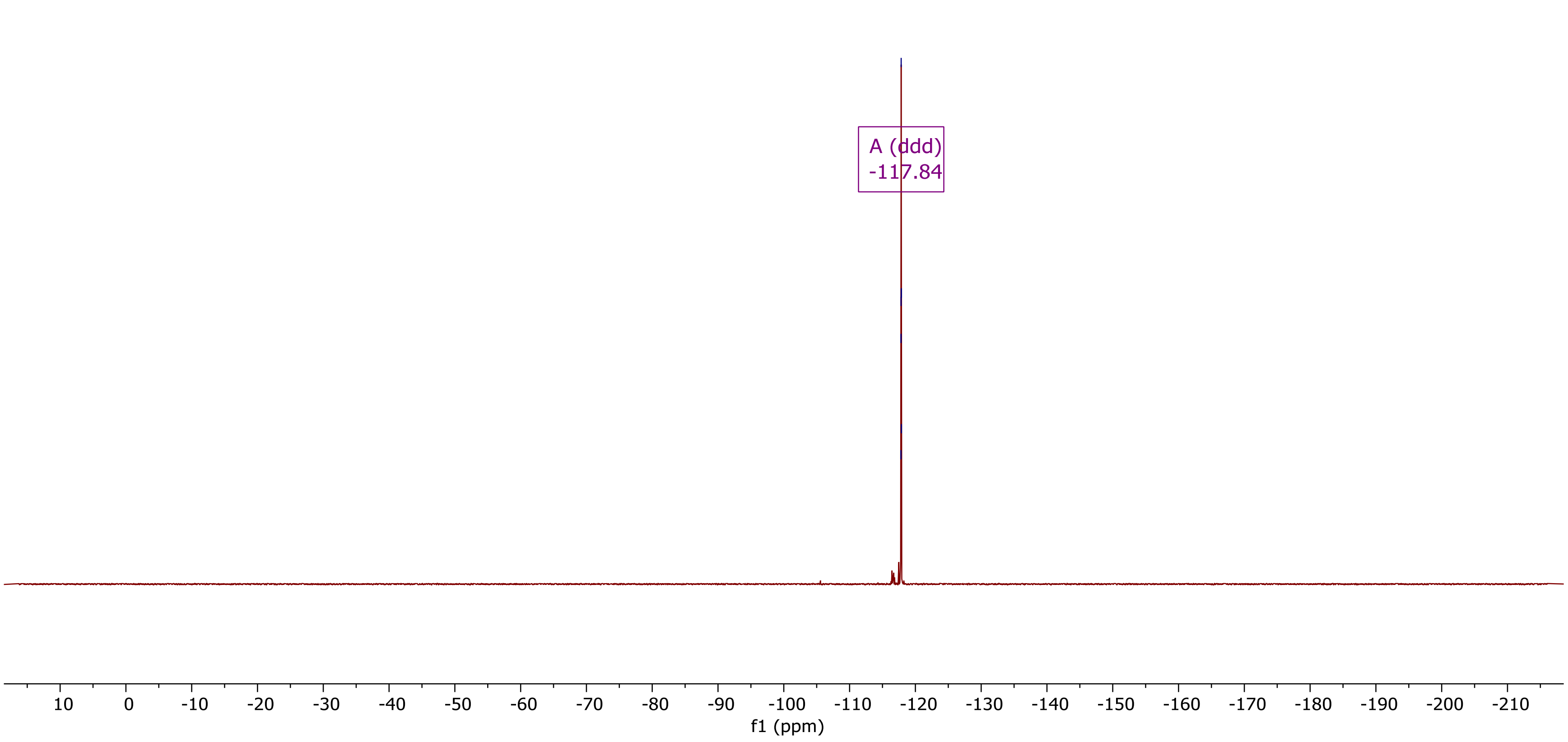

[21a]
13C NMR at 100.63 MHz in CDCl3

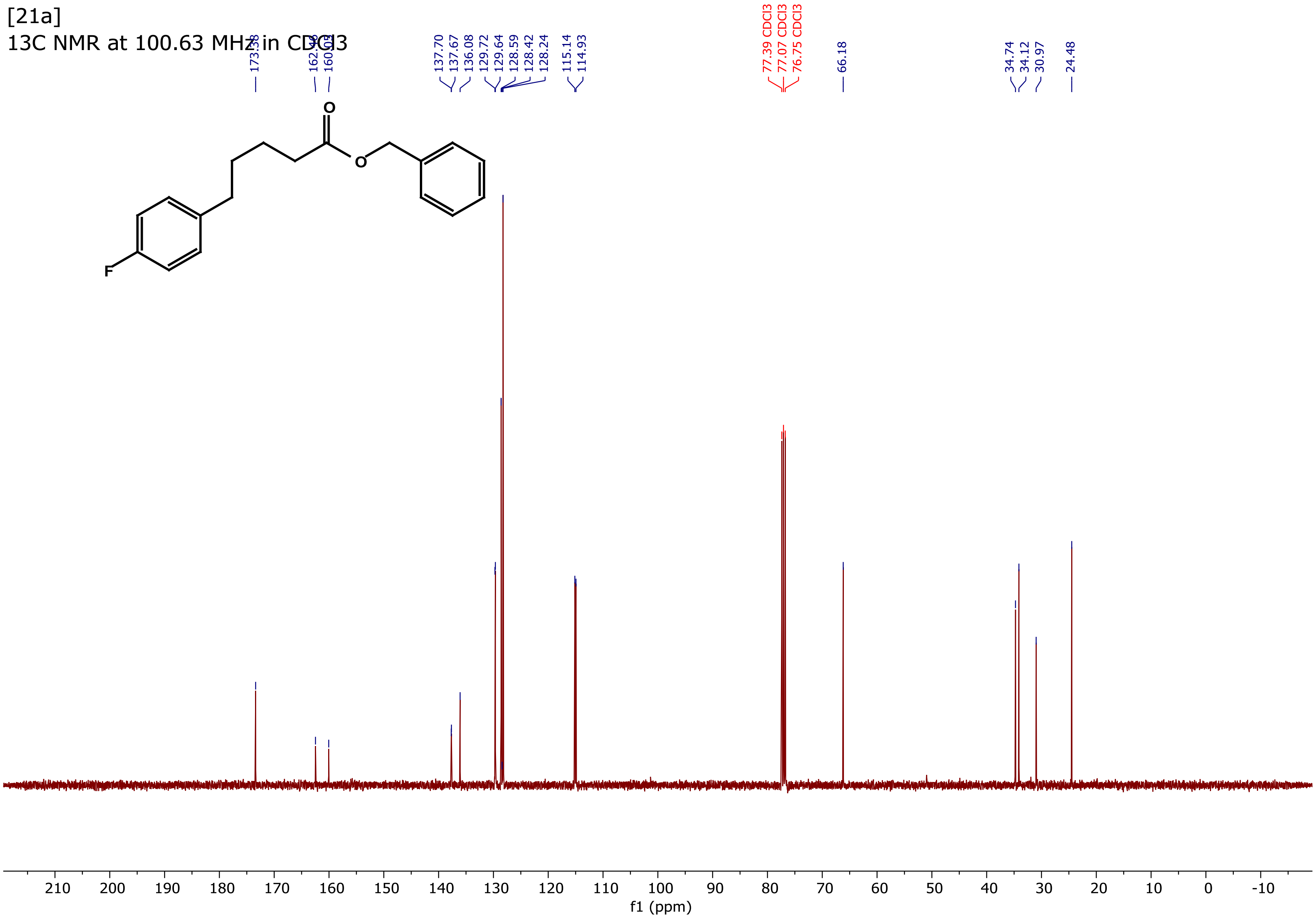

GCMS of benzyl 5-(3-fluorophenyl)pentanoate

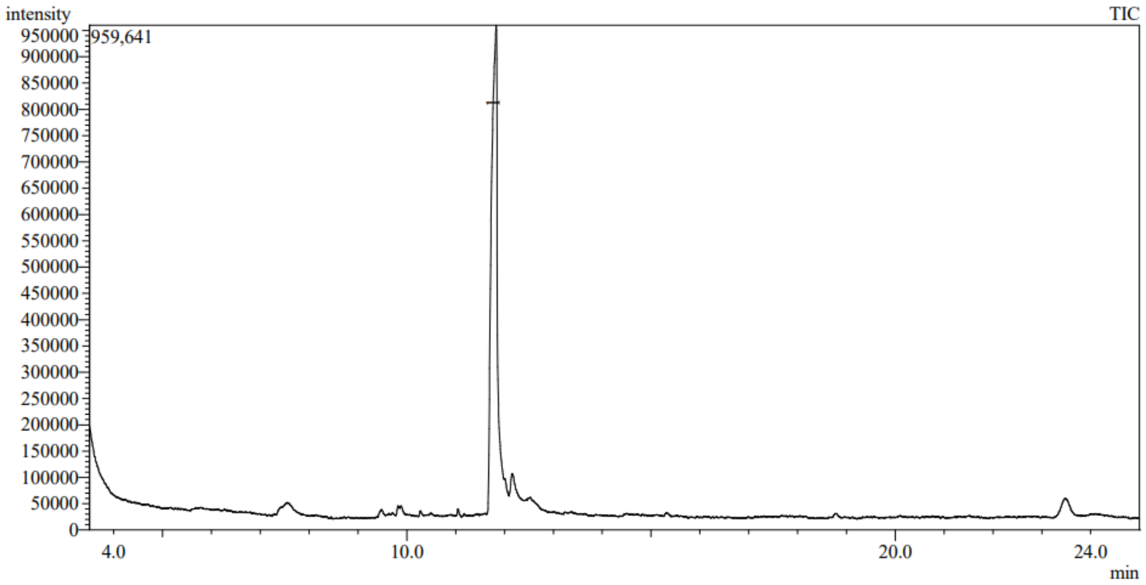

Peak Report TIC

Library
<< Target >>
Line#:1 R.Time:11.765(Scan#:1654) MassPeaks:341
RawMode:Single 11.765(1654) BasePeak:91.10(410596)
BG Mode:None Group 1 - Event 1 Scan

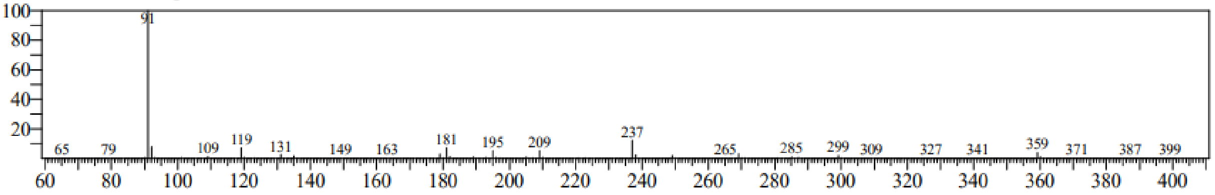

[22a]
1H NMR at 400.15 MHz in CDCl3

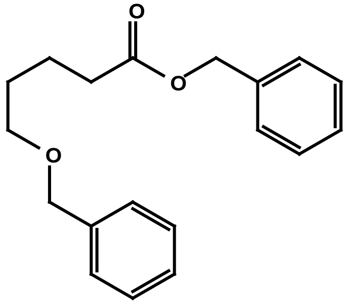

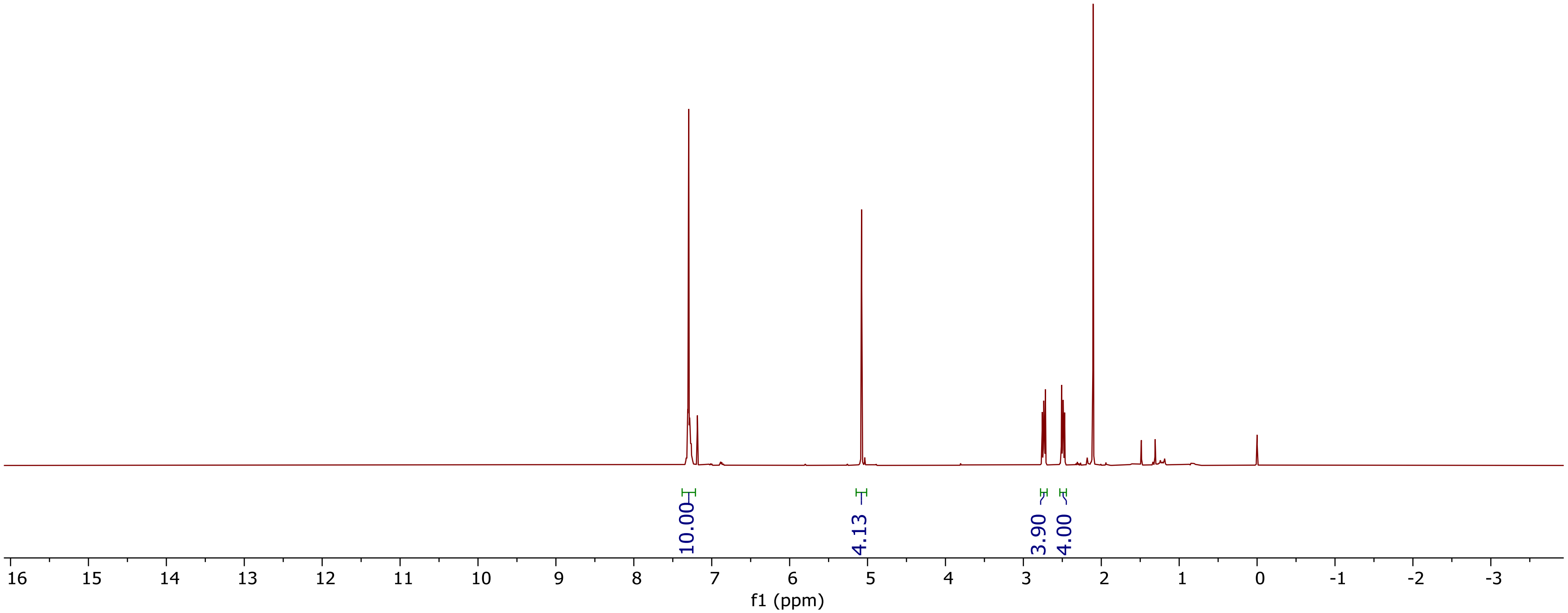

[22a]
13C NMR at 100.63 MHz in CDCl3

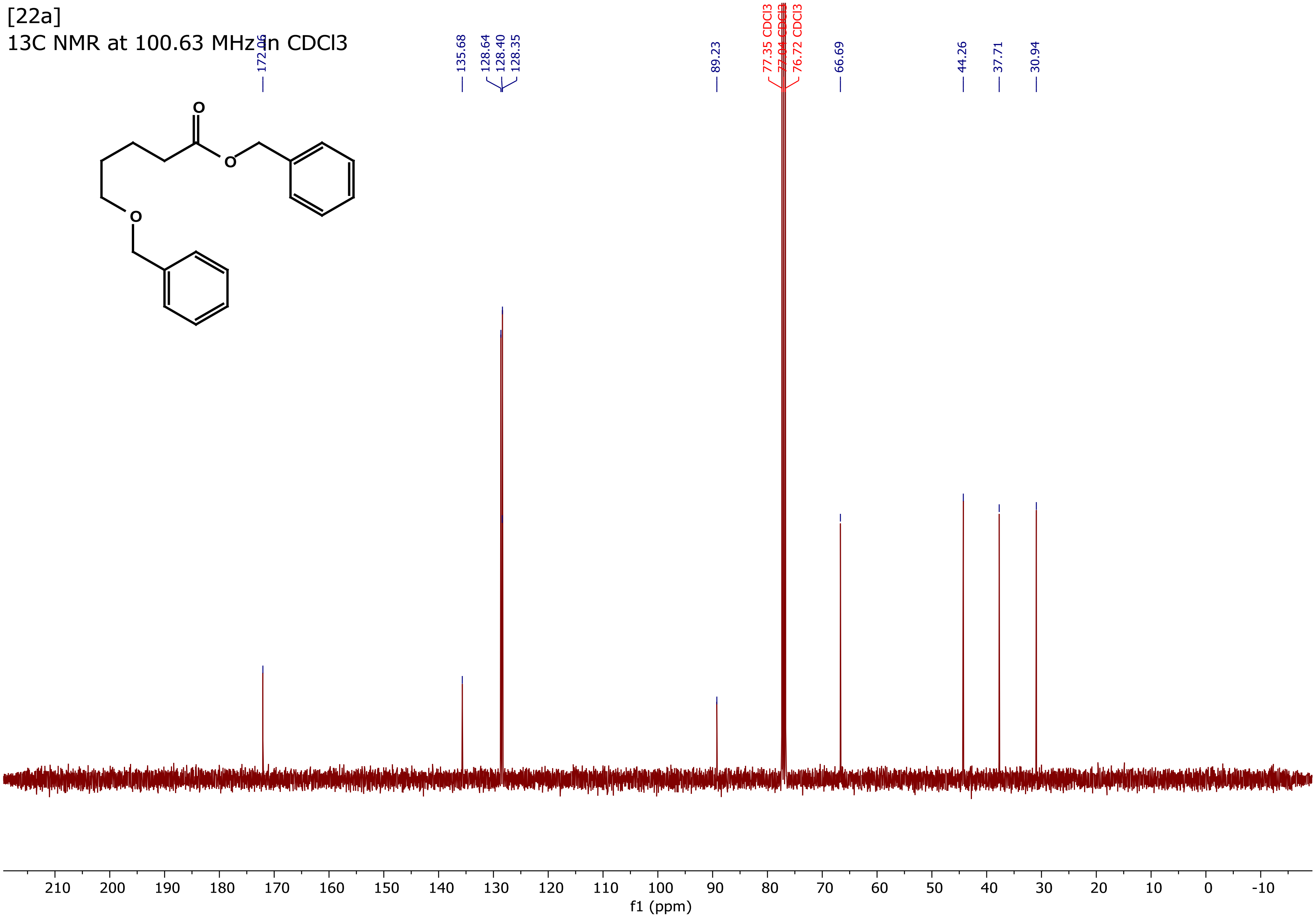

GCMS of benzyl 5-phenoxypentanoate

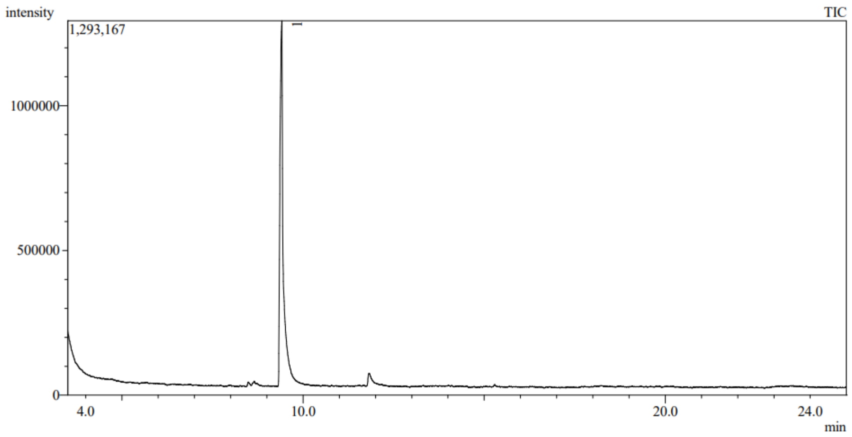

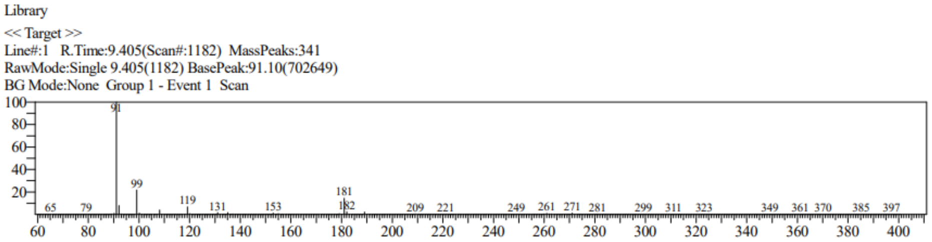

[23a]
1H NMR at 800.34 MHz in CDCl3

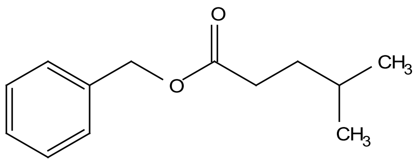

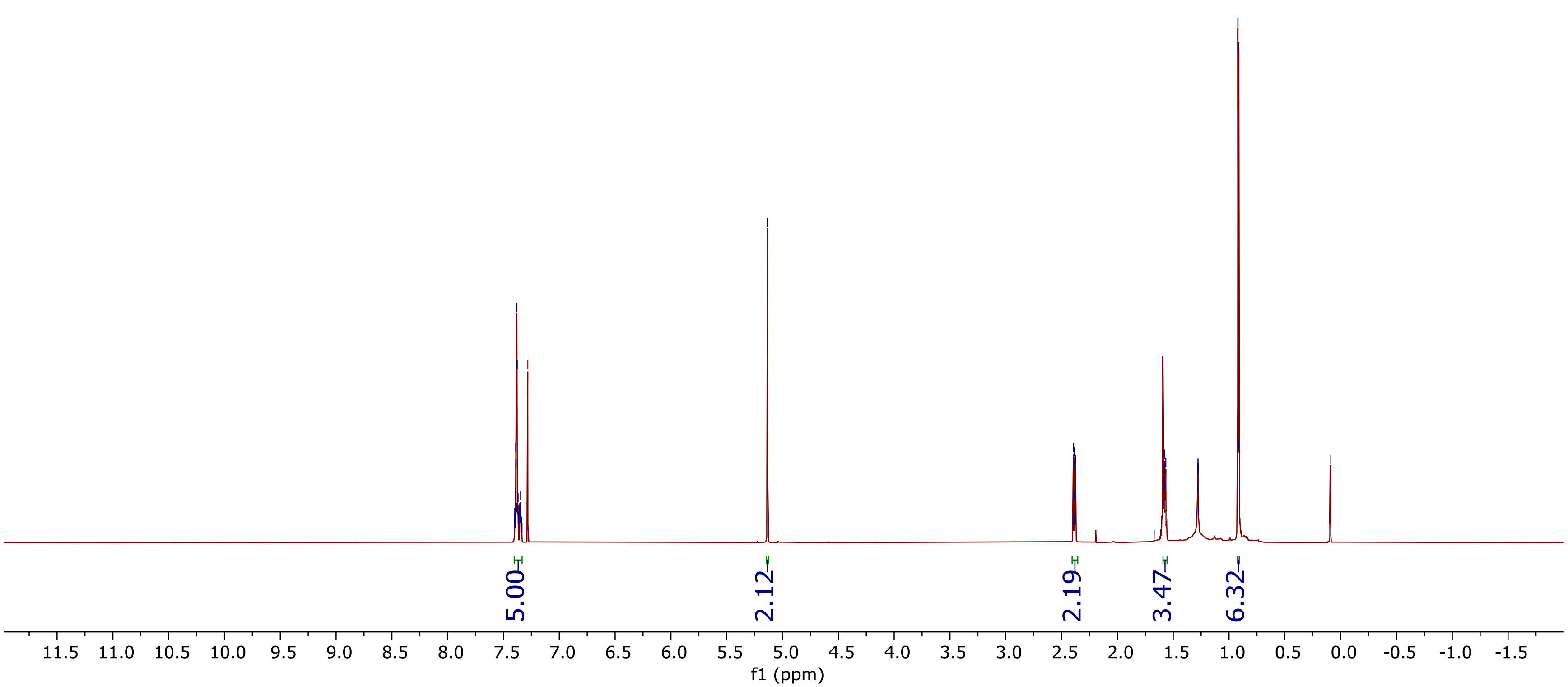

[23a]
13C NMR at 201.27 MHz in CDCl3

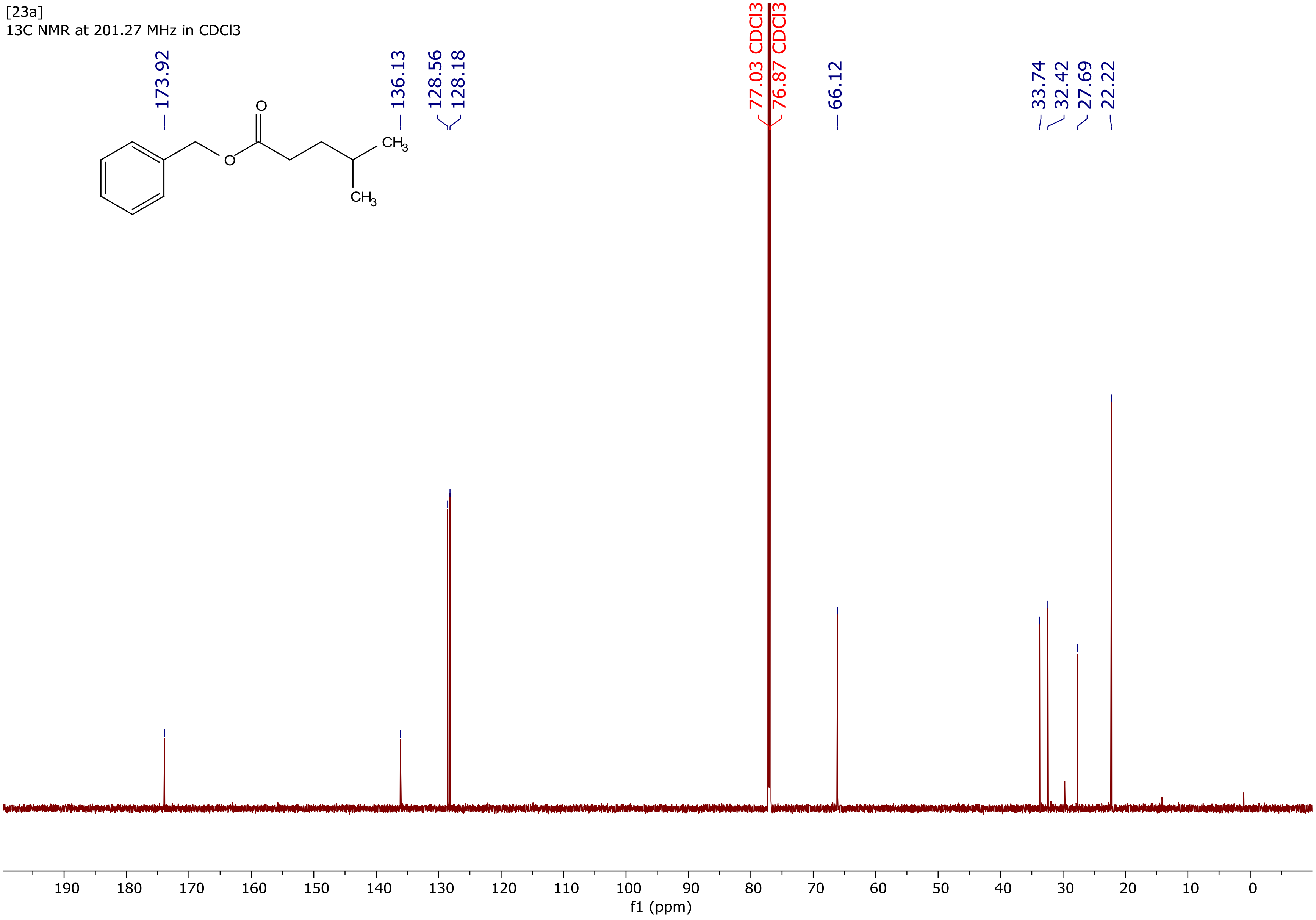

[24a-Br]
1H NMR at 400.15 MHz in CDCl3

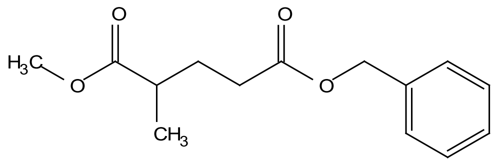

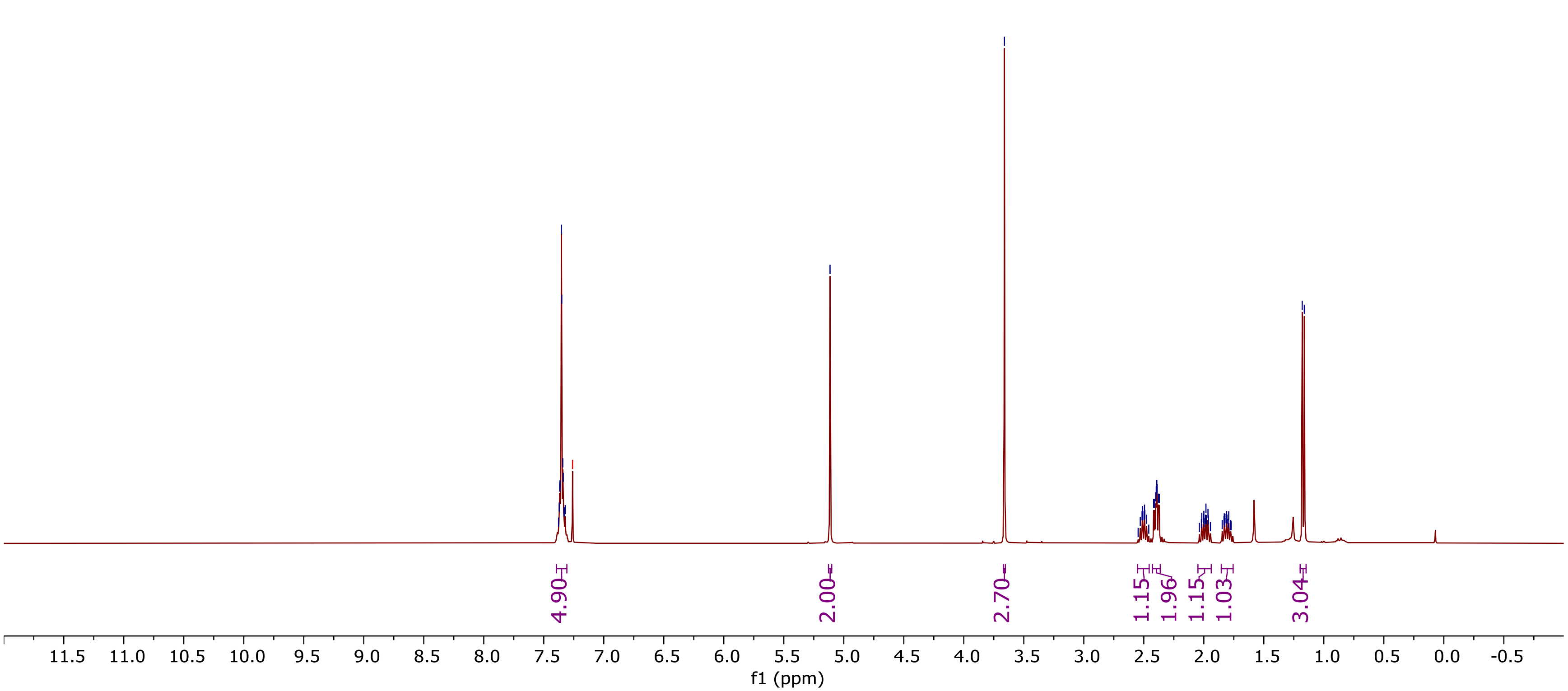

[24a-Cl]
1H NMR at 800.34 MHz in CDCl3

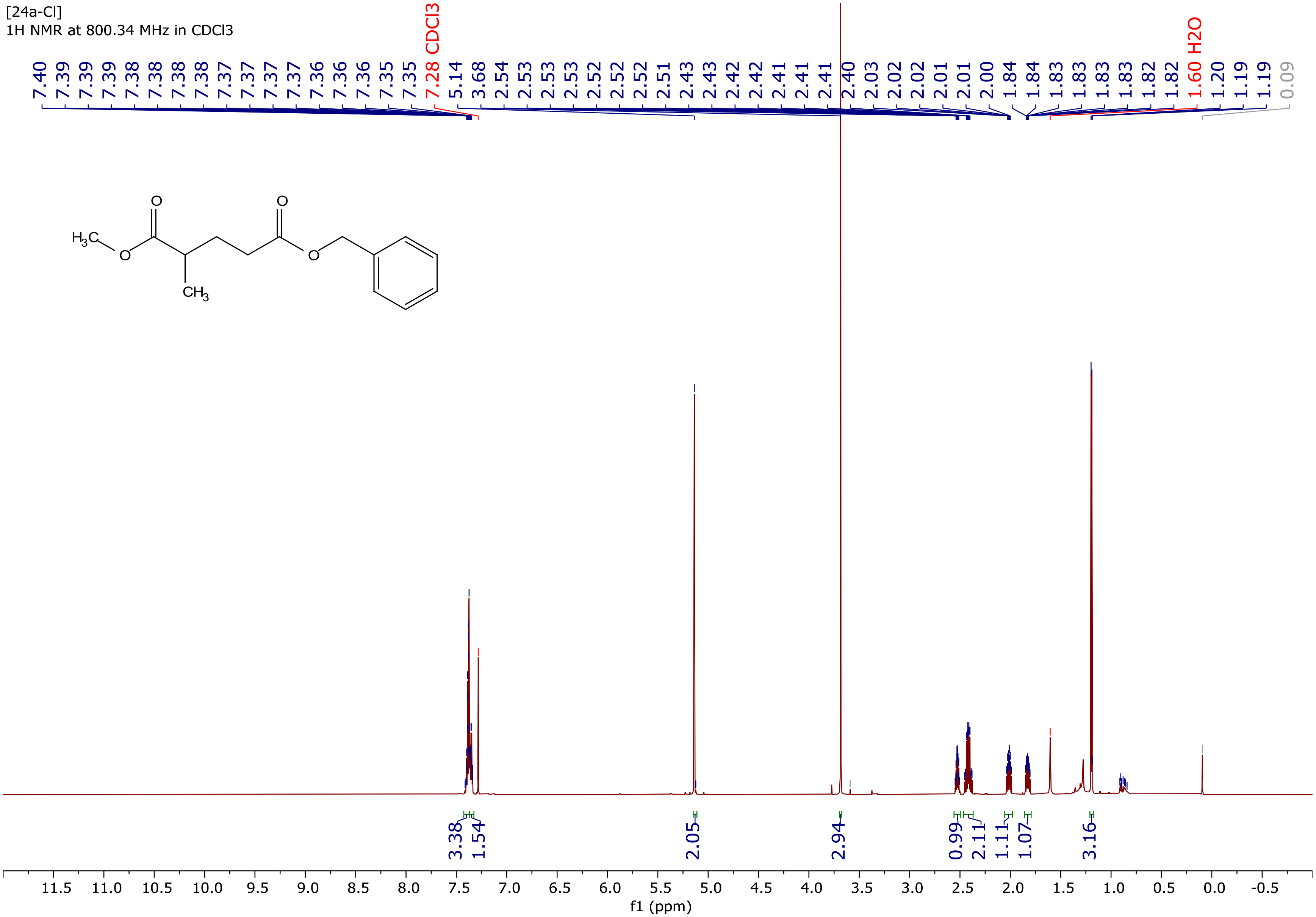

[24a-Cl]
13C NMR at 201.27 MHz in CDCl3

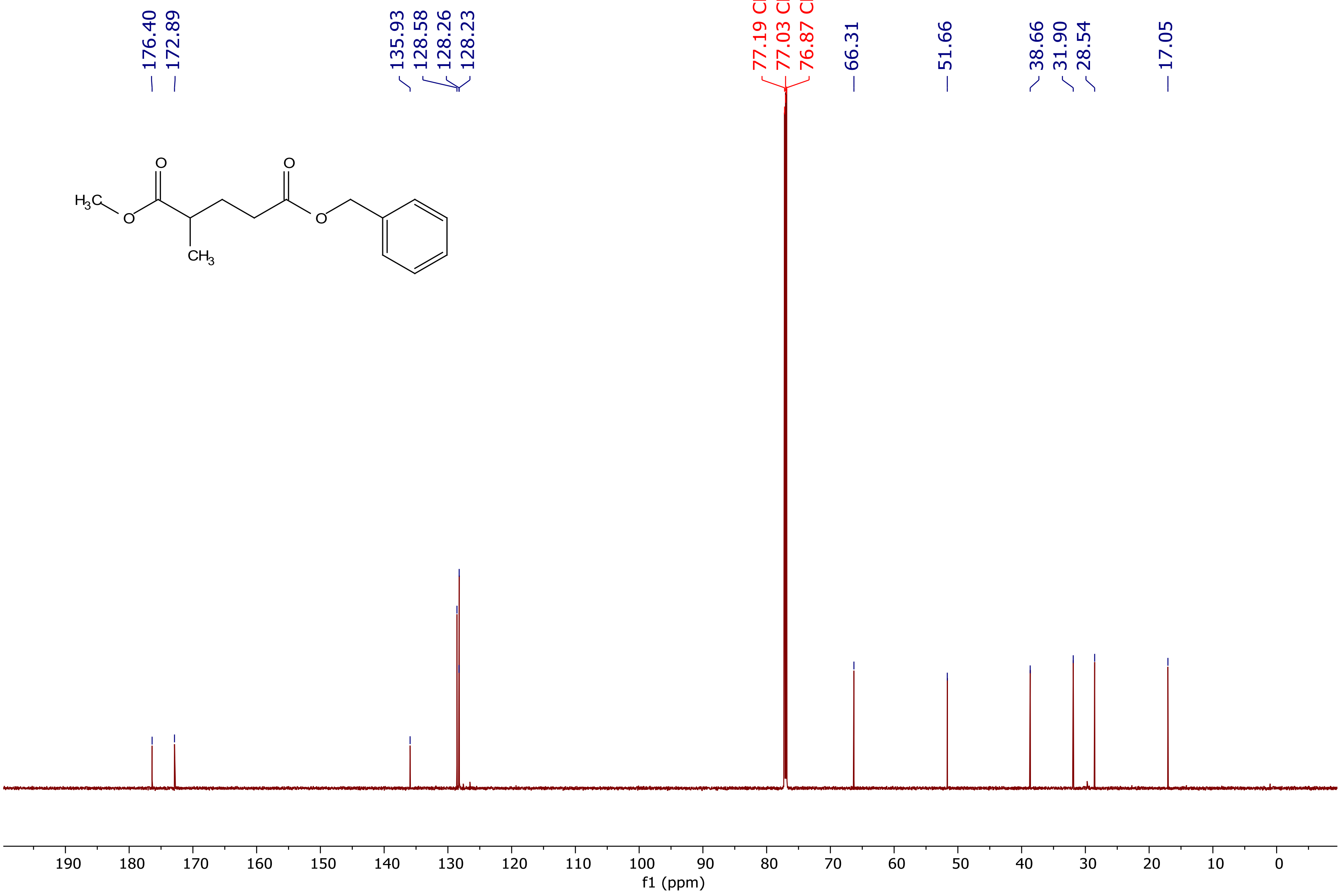

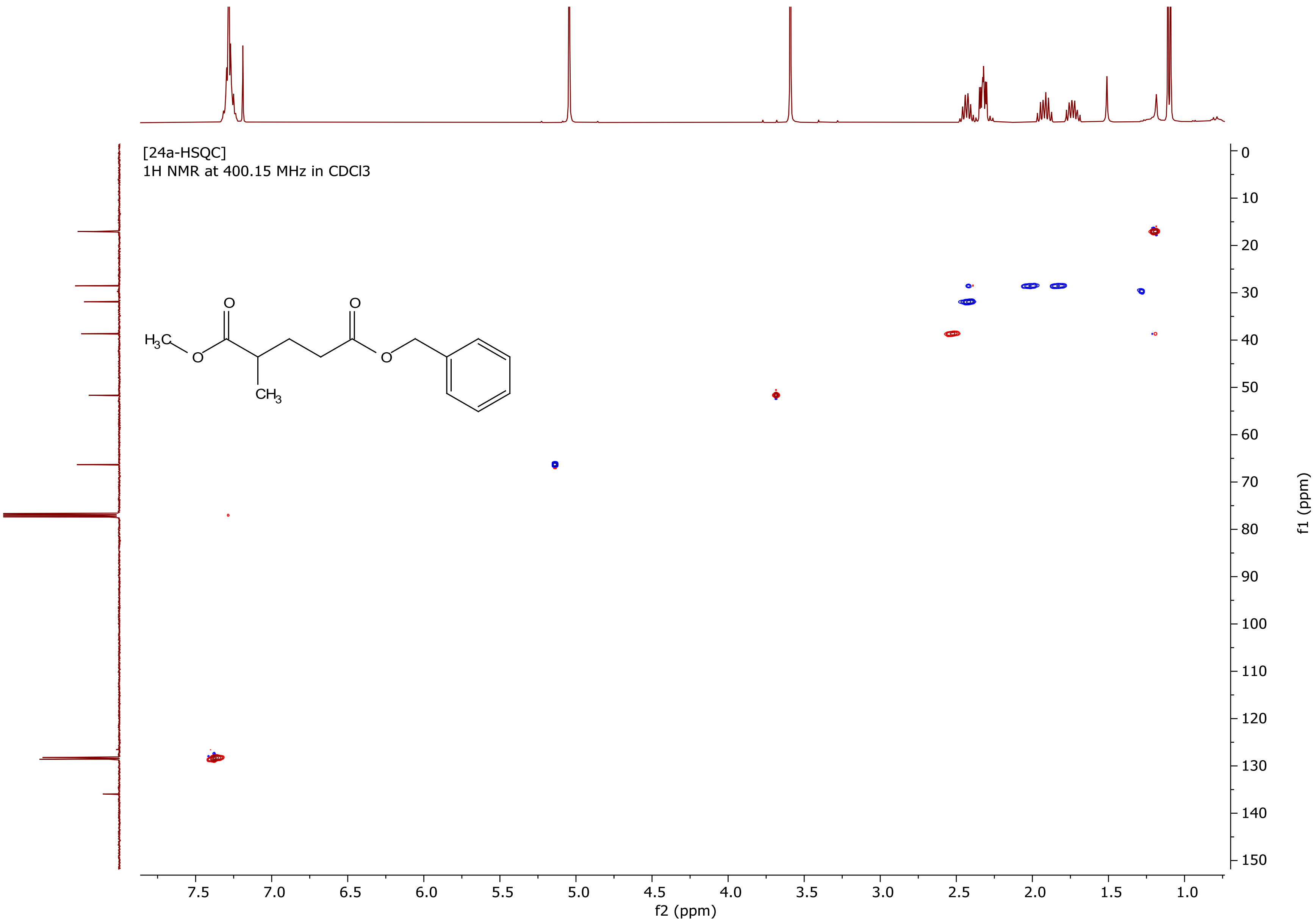
[24a-HSQC]
1H NMR at 400.15 MHz in CDCl3
H3C
O
O
O
O
CH3
f2 (ppm)
f1 (ppm)
7.5
7.0
6.5
6.0
5.5
5.0
4.5
4.0
3.5
3.0
2.5
2.0
1.5
1.0
0
10
20
30
40
50
60
70
80
90
100
110
120
130
140
150

[25a]
1H NMR at 400.15 MHz in CDCl3

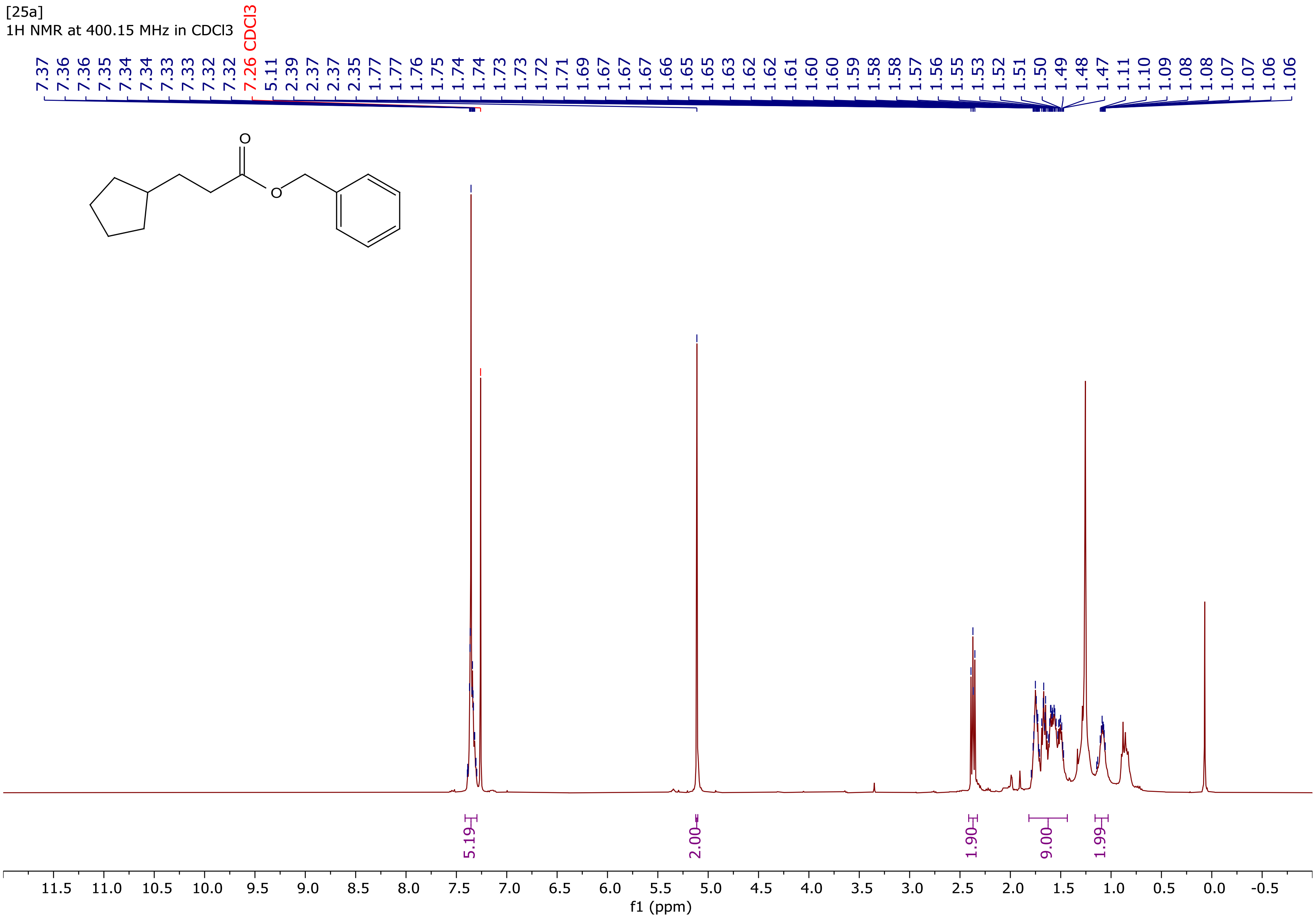

[25a]
13C NMR at 100.63 MHz in CDCl3

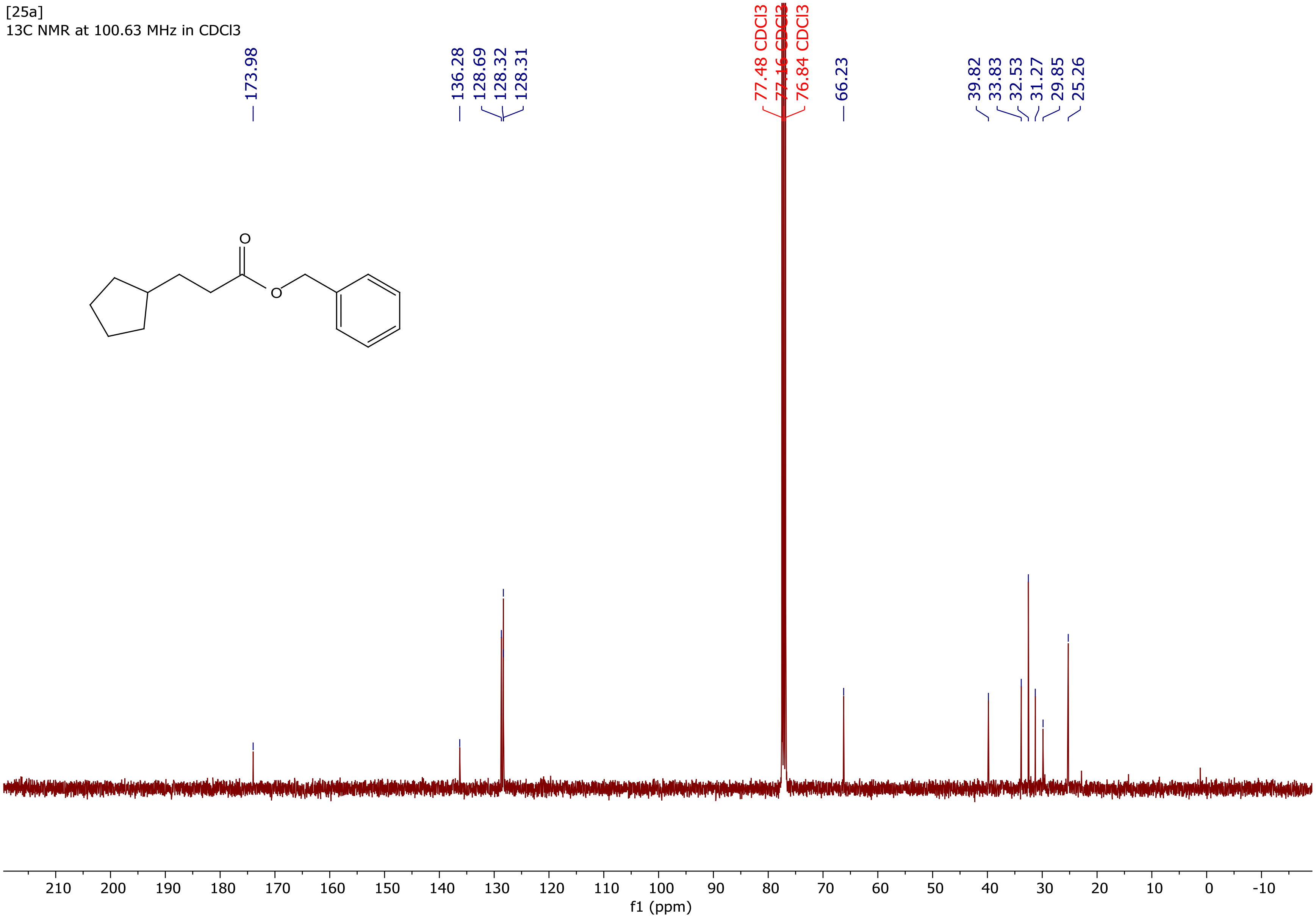

[26a]
1H NMR at 400.15 MHz in CDCl3

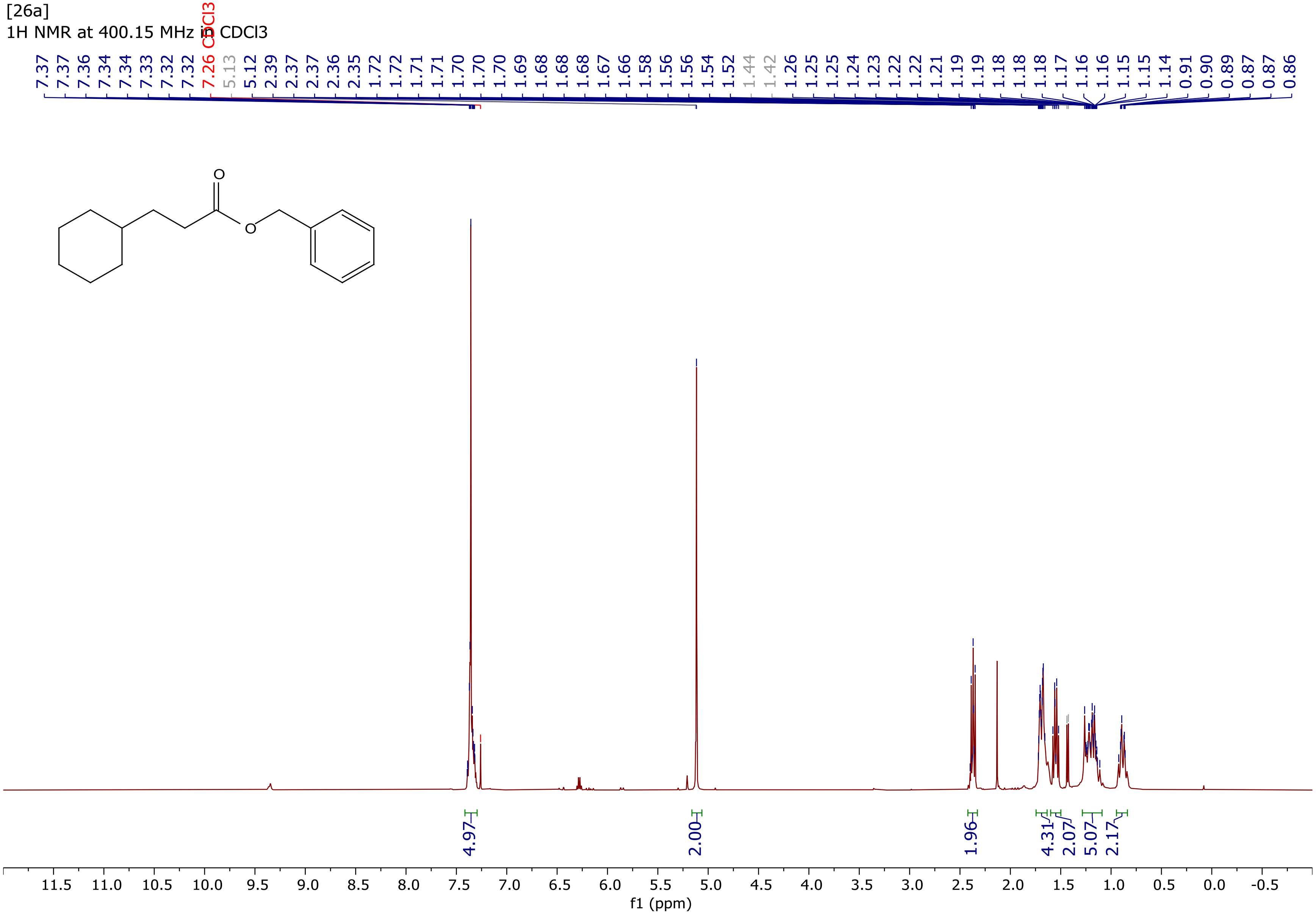

[26a]
13C NMR at 201.27 MHz in CDCl3

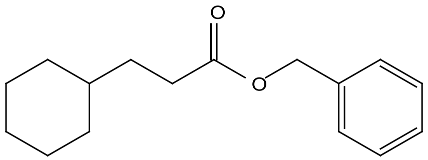

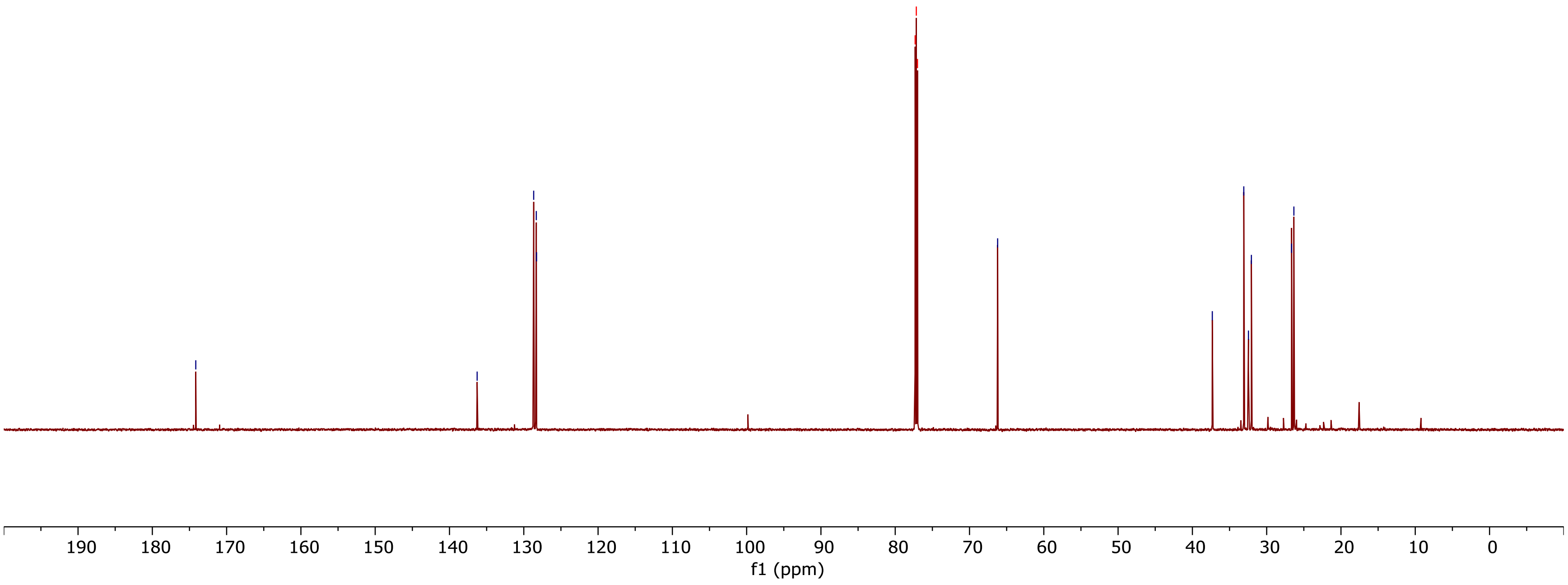

[27a]
1H NMR at 400.15 MHz in CDCl3

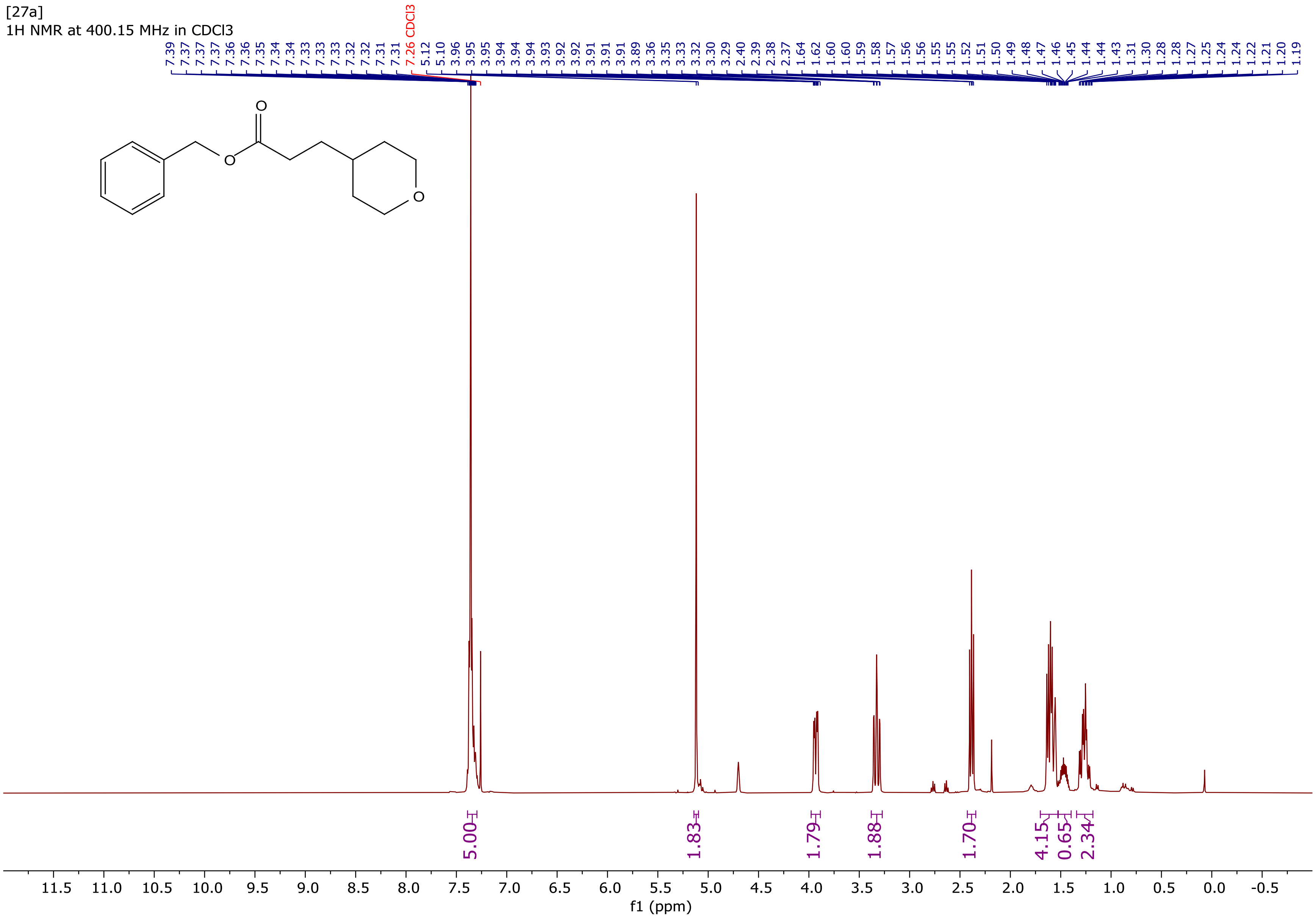

[27a]
13C NMR at 100.63 MHz in CDCl3

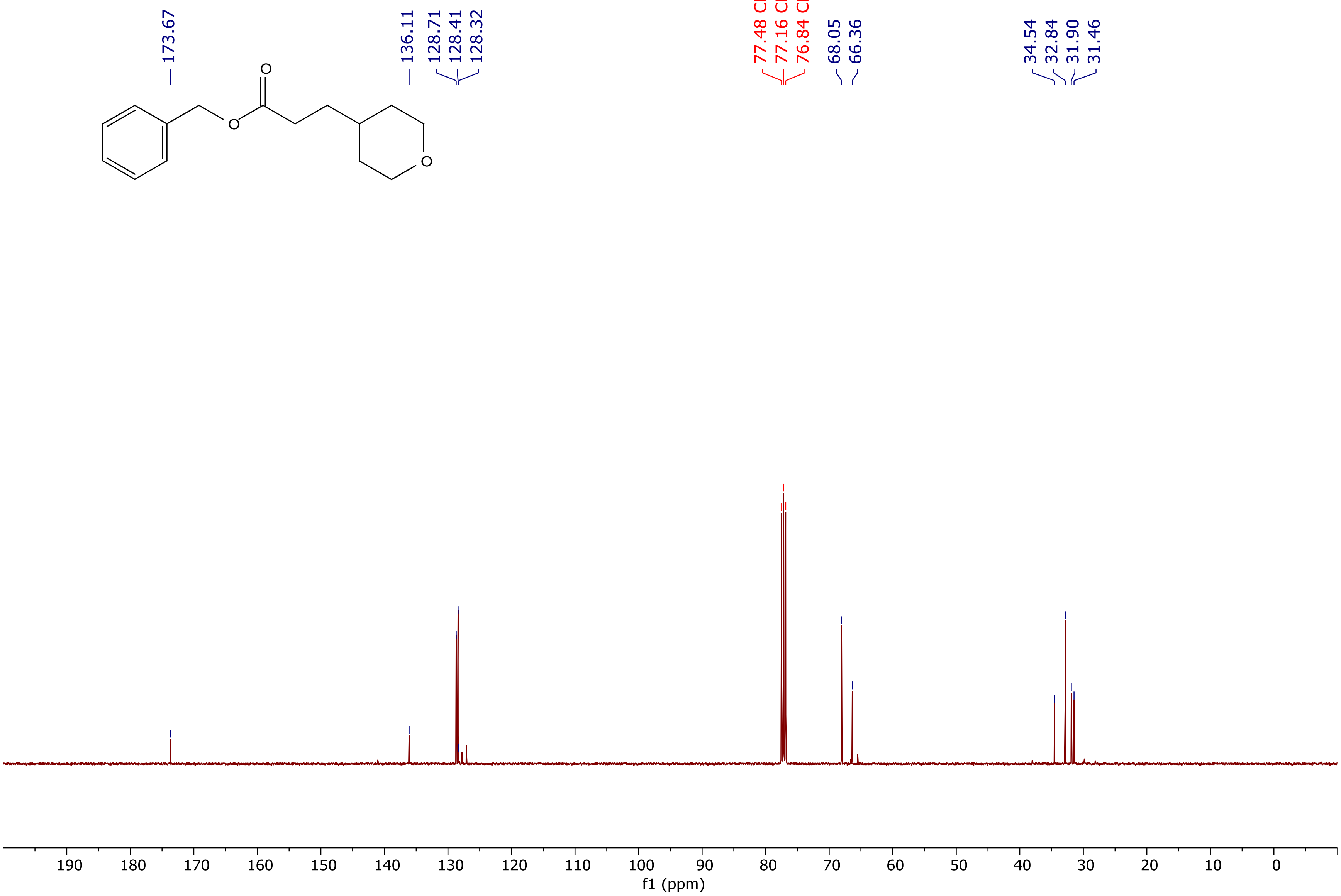

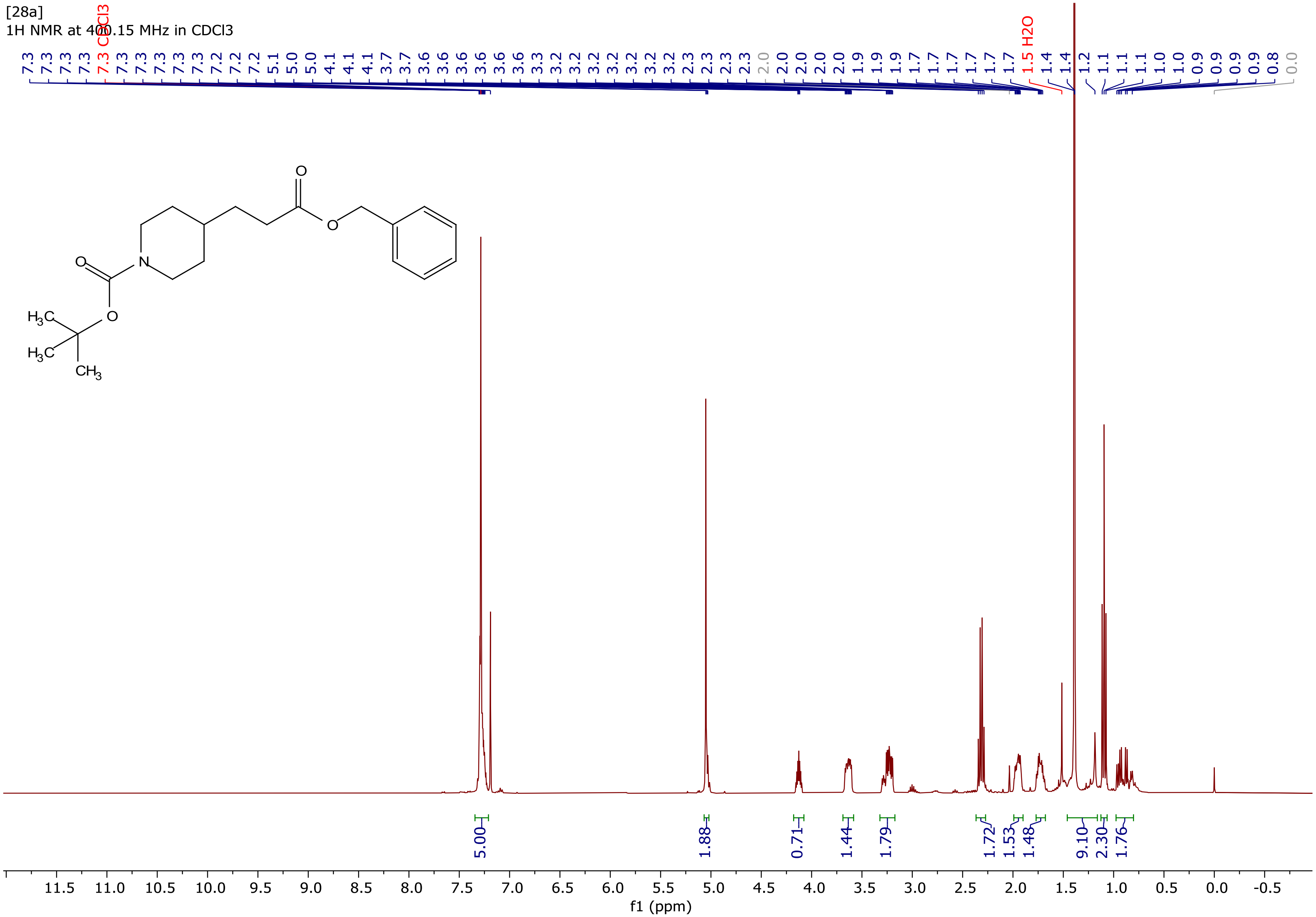
[28a]
1H NMR at 400.15 MHz in CDCl3
7.3 CDCl3
1.5 H2O
5.00
1.88
0.71
1.44
1.79
1.72
1.53
1.48
9.10
2.30
1.76
f1 (ppm)

[28a]
13C NMR at 201.27 MHz in CDCl3

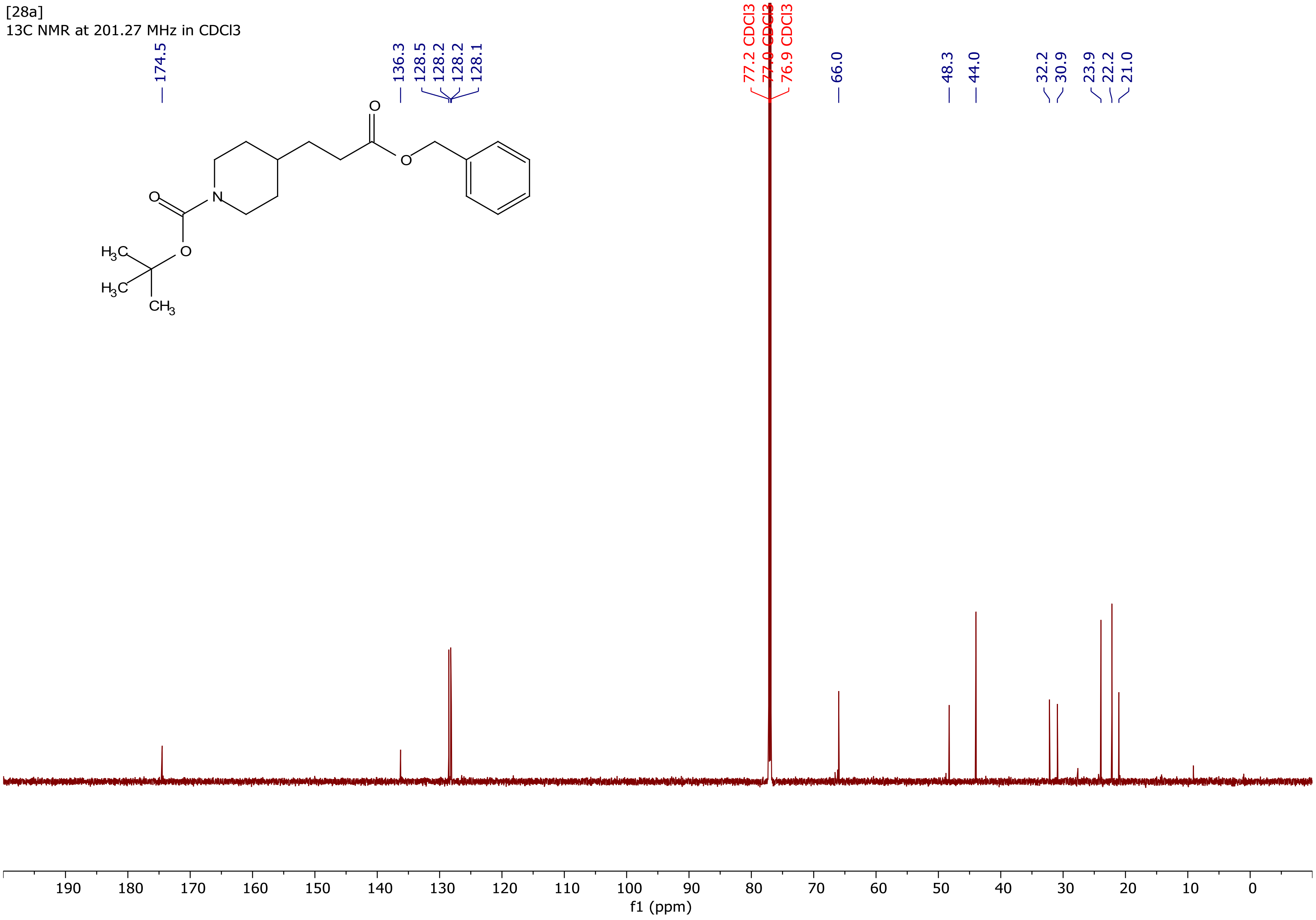

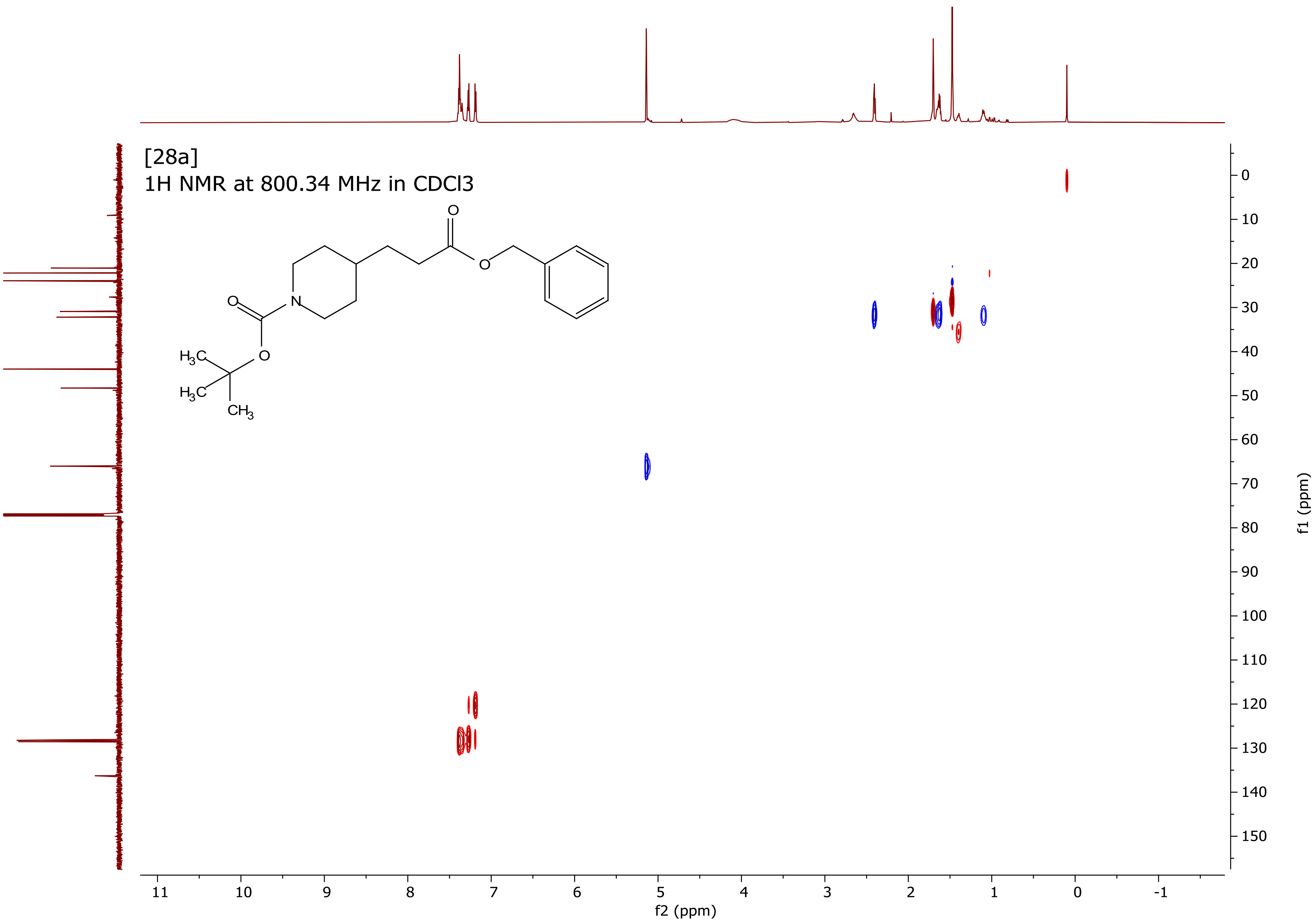
[28a]
1H NMR at 800.34 MHz in CDCl3
O
N
O
O
O
H3C
H3C
CH3
f2 (ppm)
f1 (ppm)

[29f]

1H NMR at 800.34 MHz in CDCl3

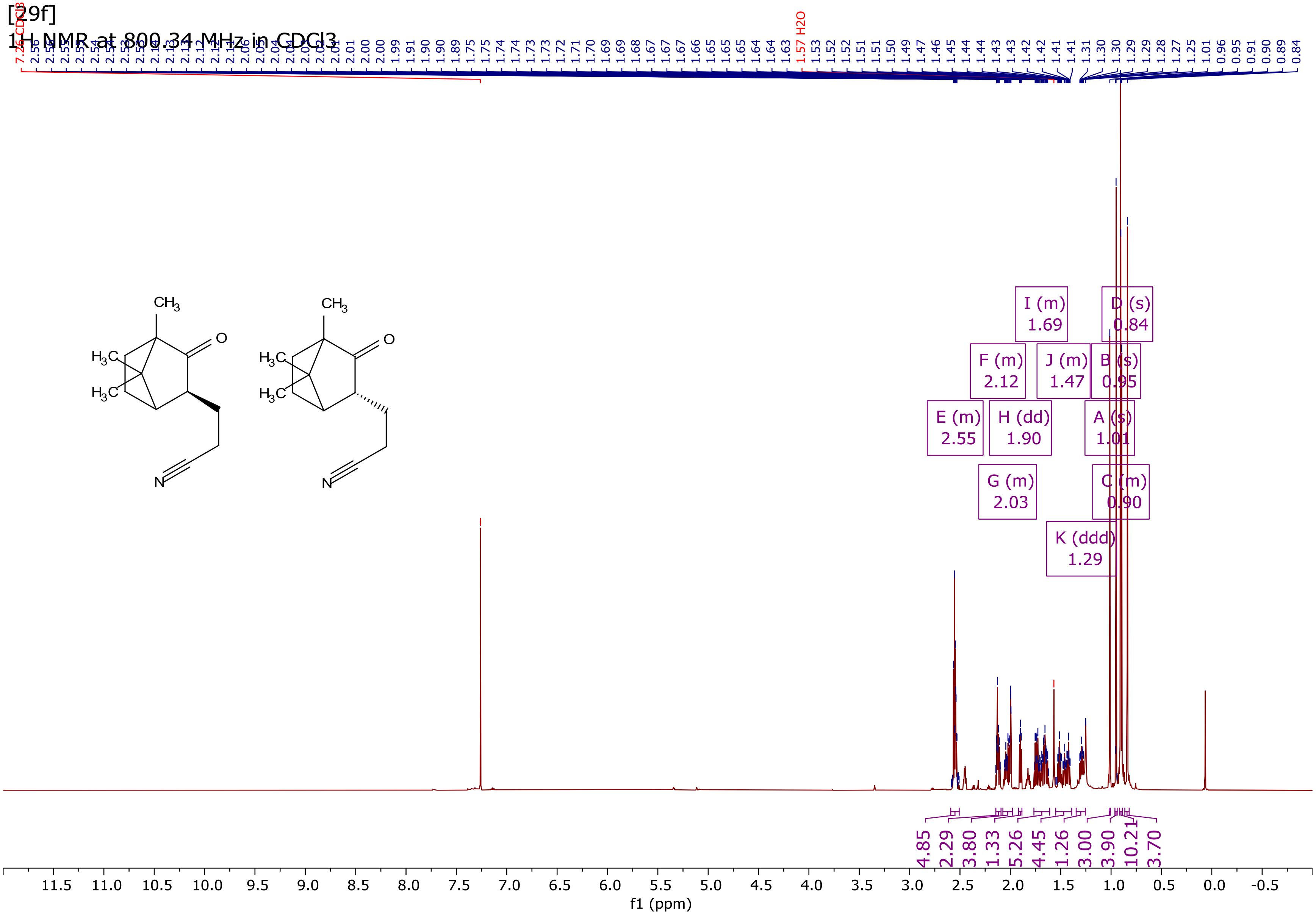

[29f]
13C NMR at 201.27 MHz in CDCl3

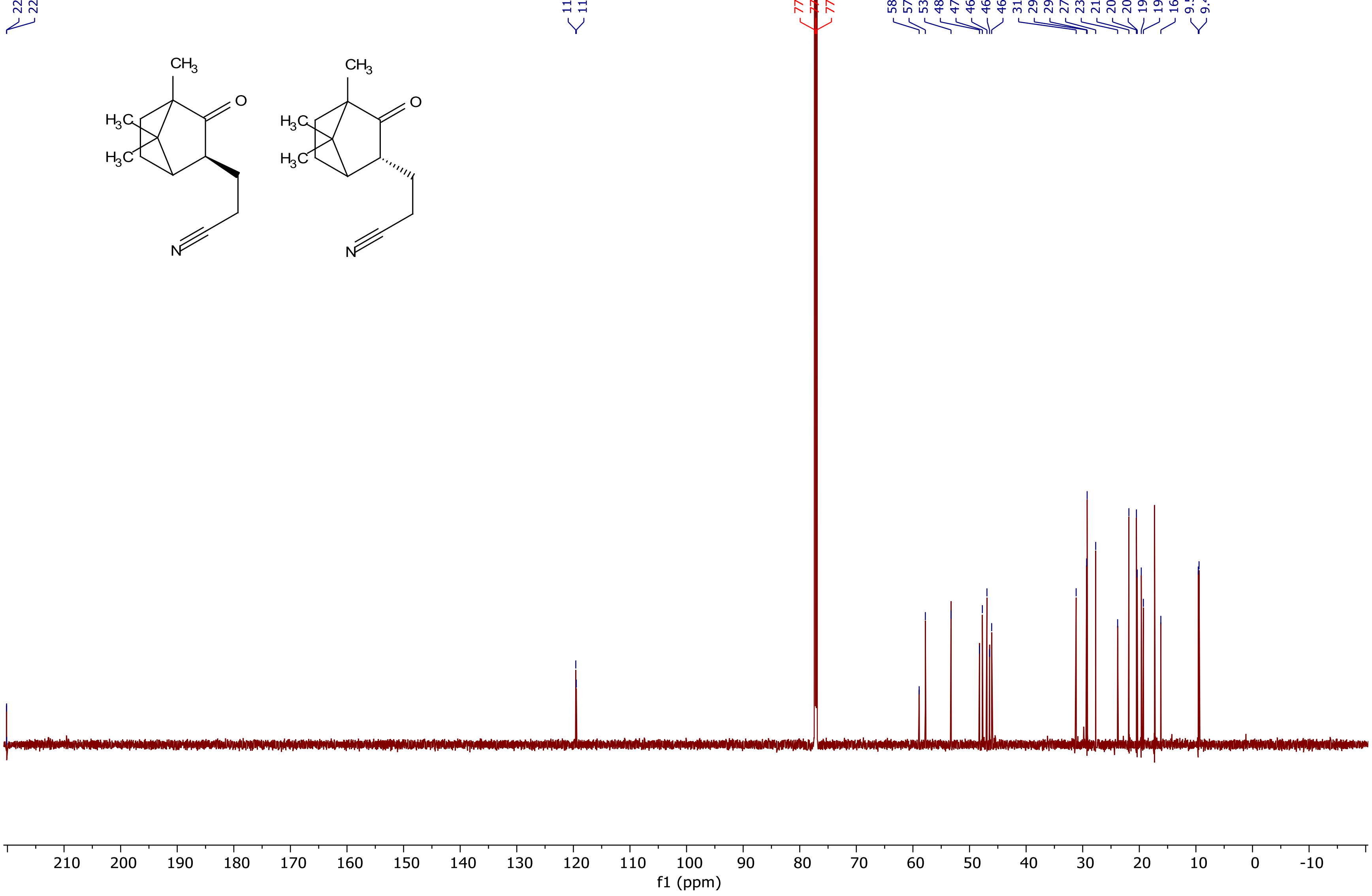

[30a]
1H NMR at 800.34 MHz in CDCl3

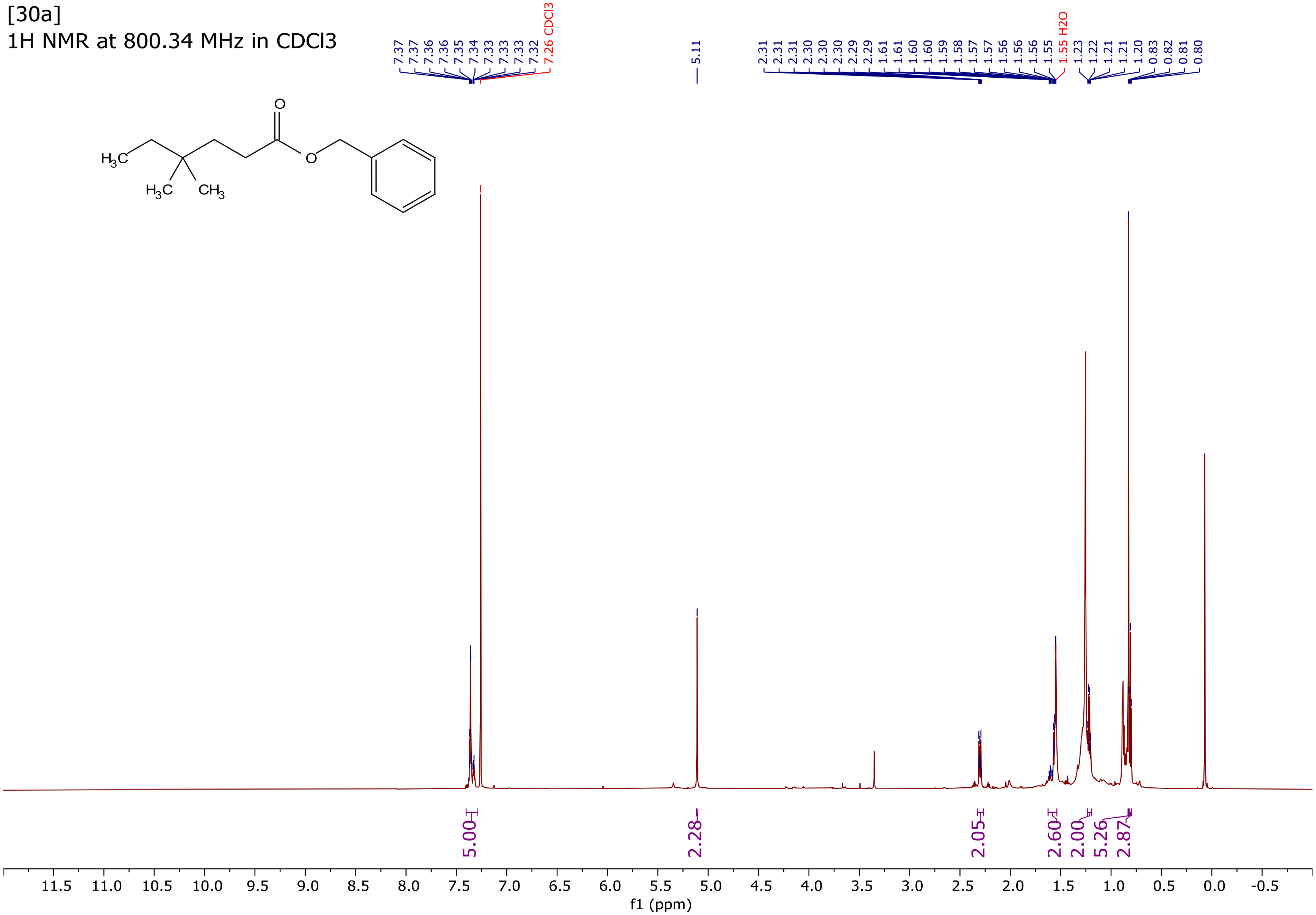

[30a]
13C NMR at 201.27 MHz in CDCl3

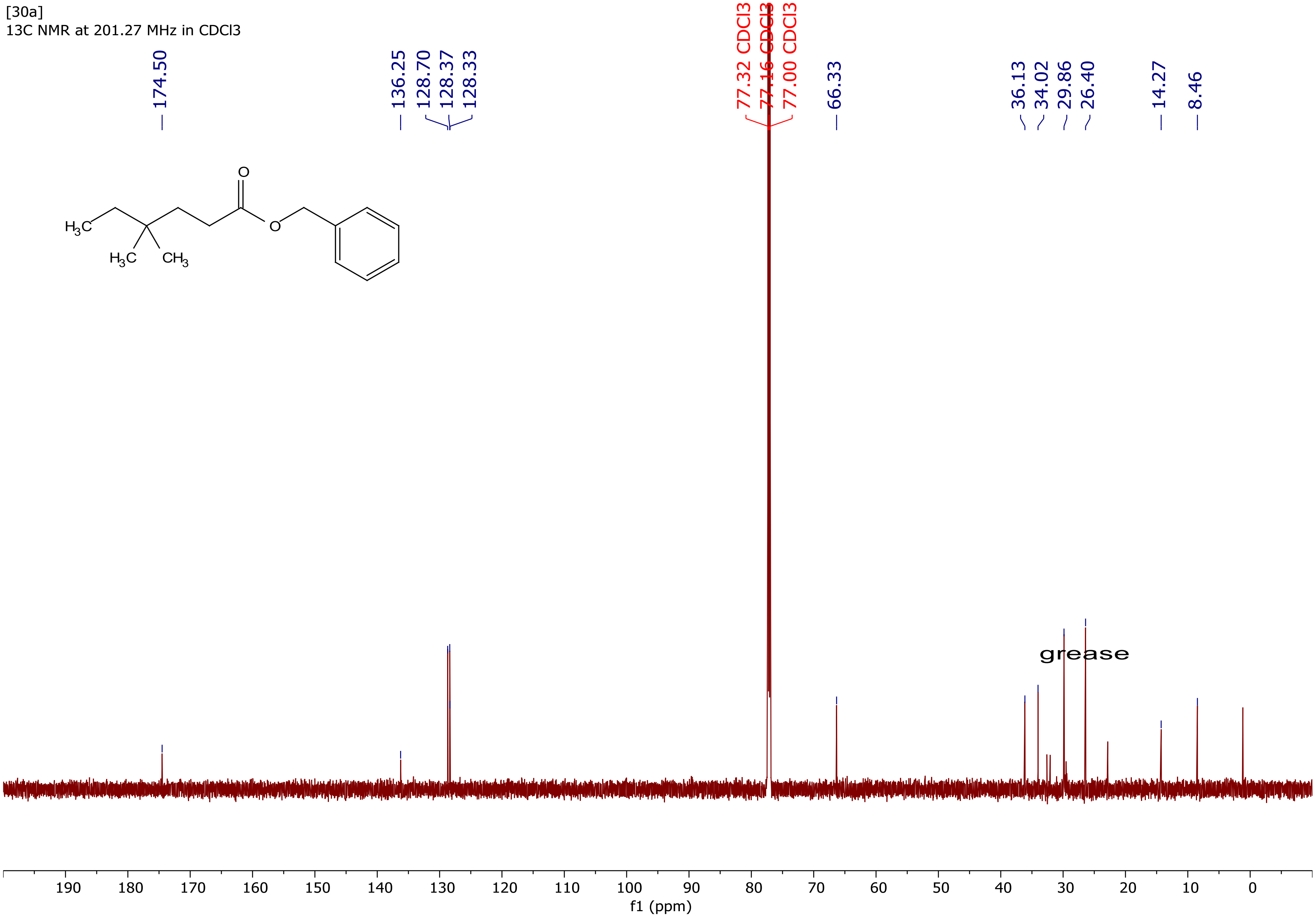

[31a]
1H NMR at 400.15 MHz in CDCl3

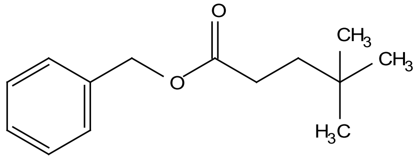

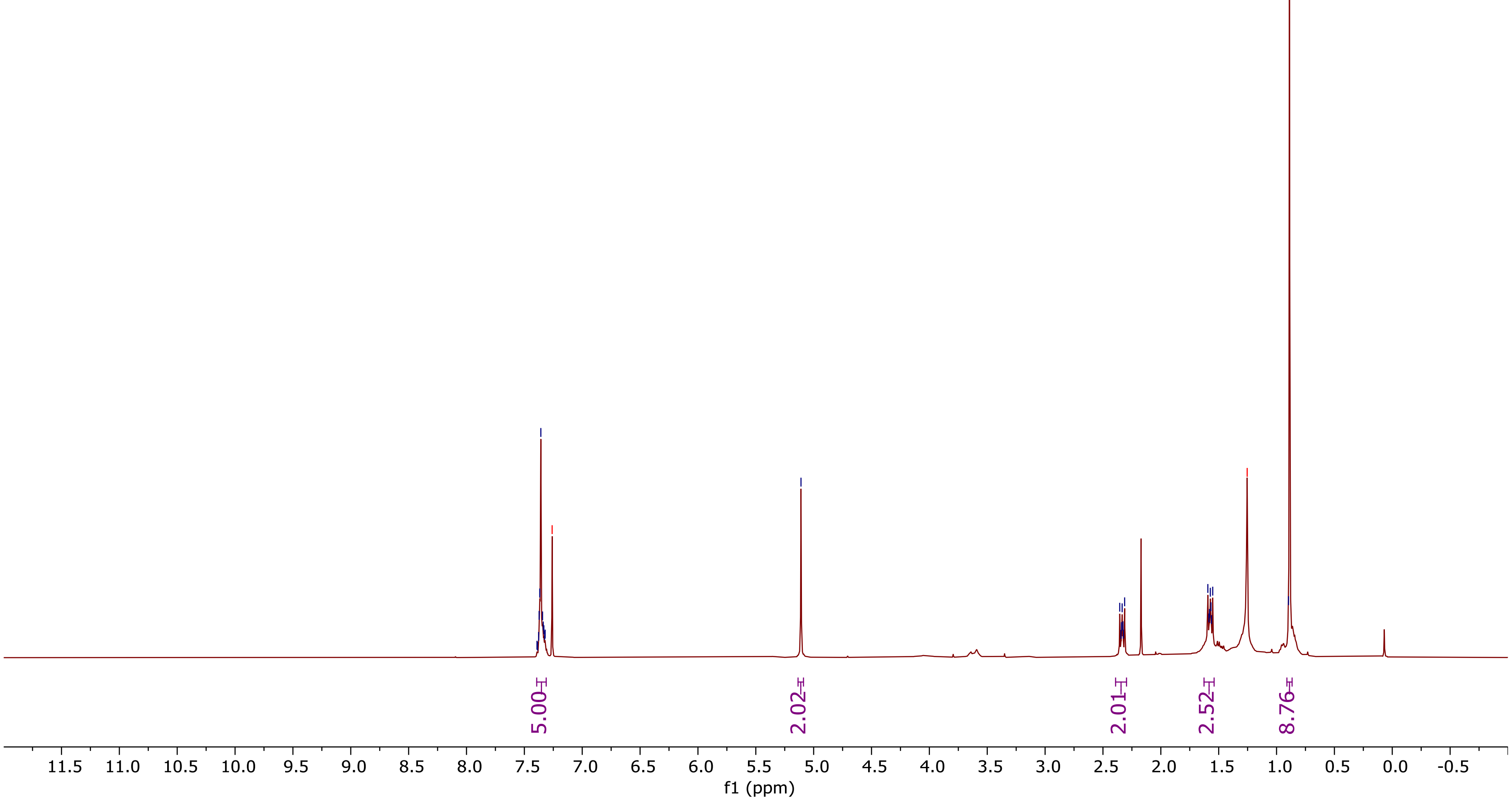

[31a]
13C NMR at 201.27 MHz in CDCl3

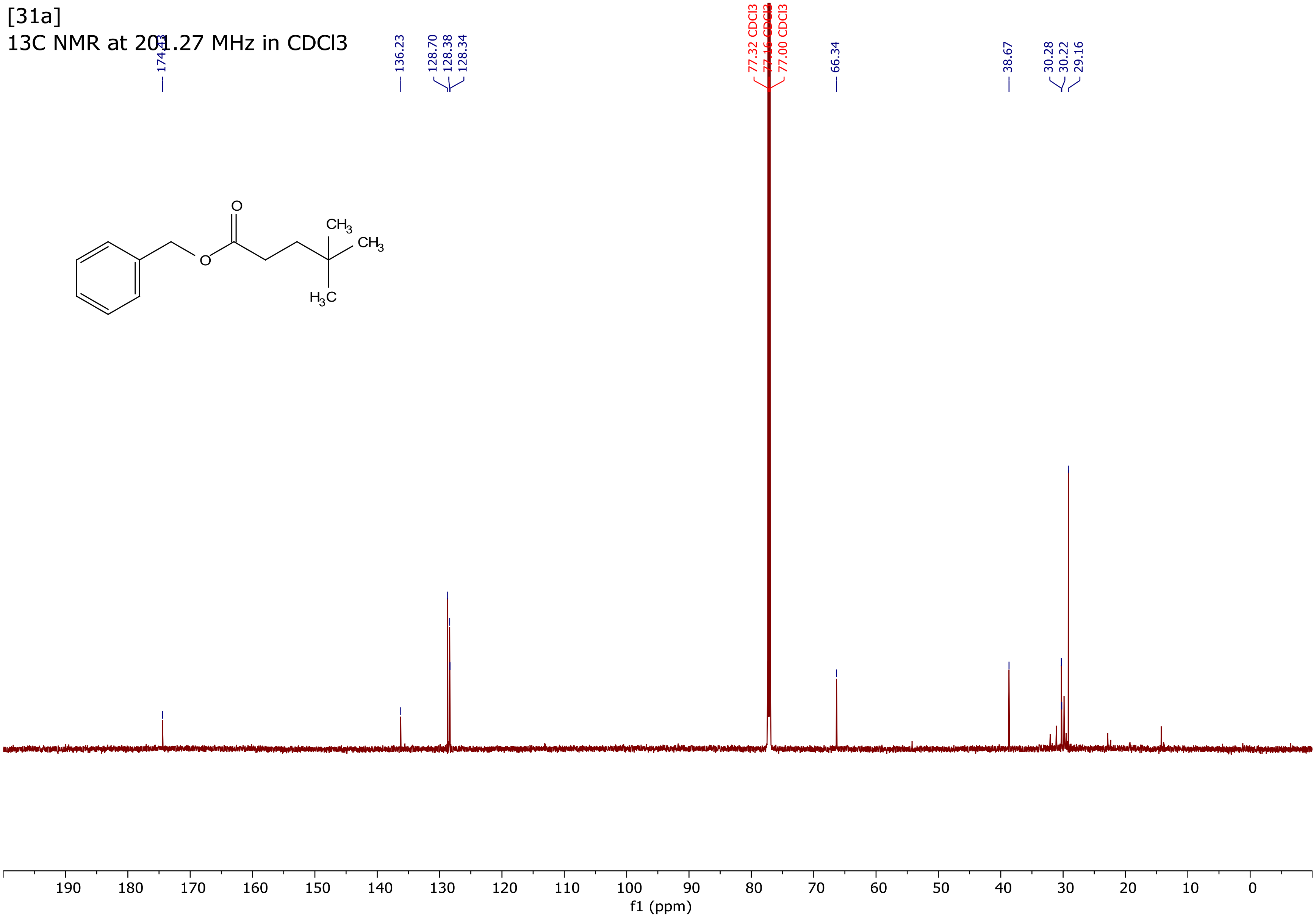

[32a]
1H NMR at 400.15 MHz in CDCl3

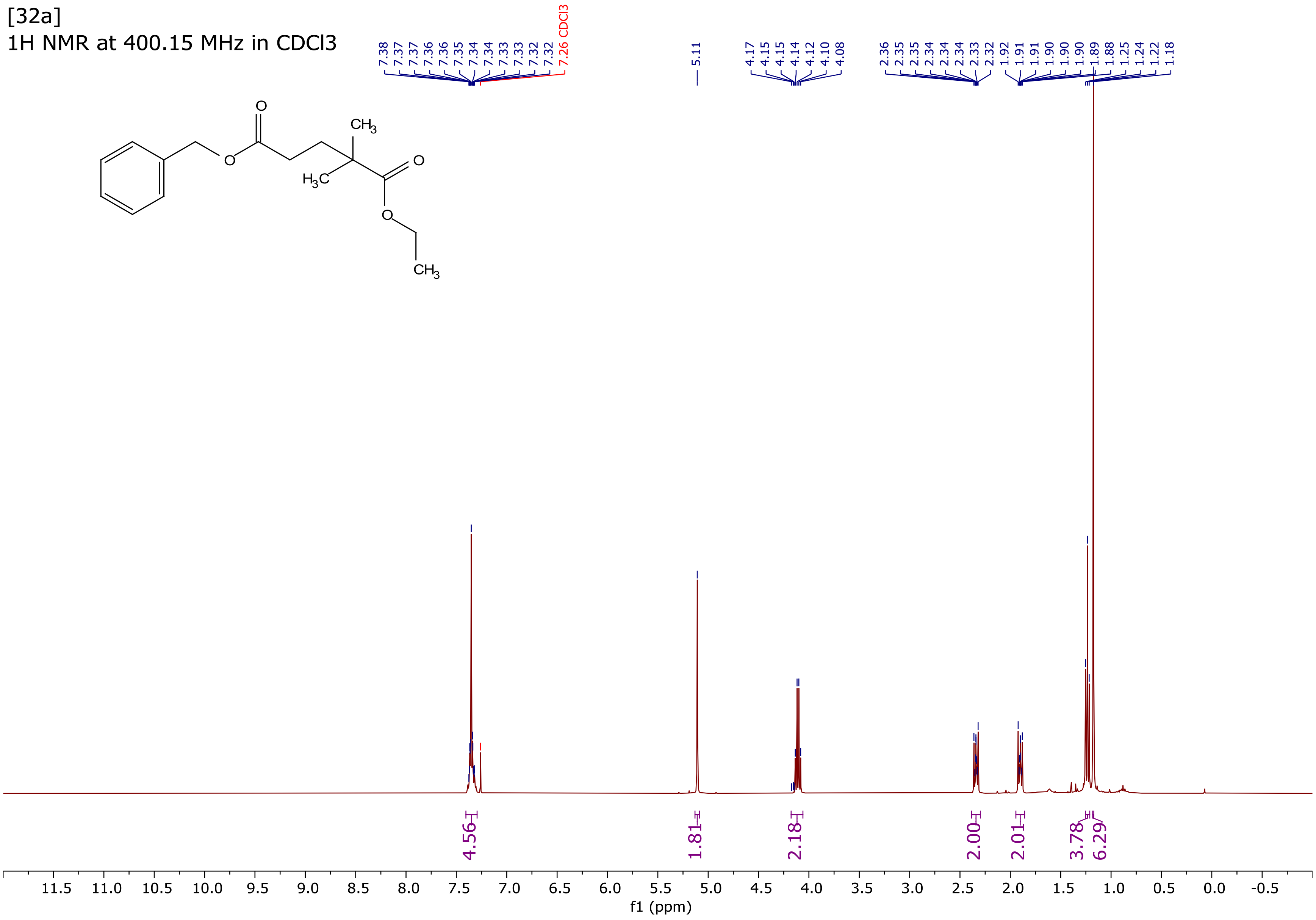

[32a]
13C NMR at 100.63 MHz in CDCl3

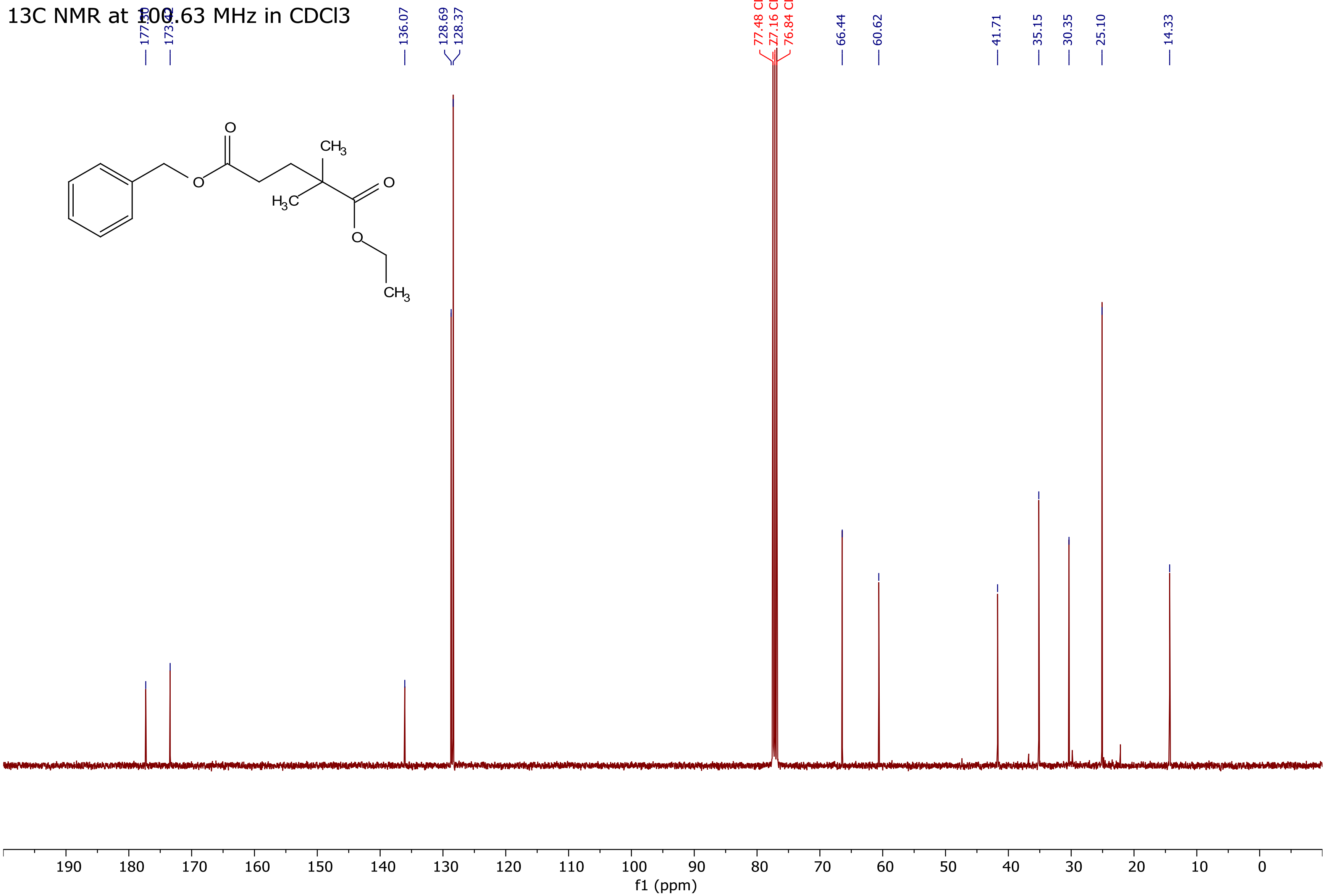

[33f]
1H NMR at 800.34 MHz in CDCl3

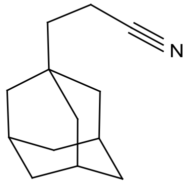

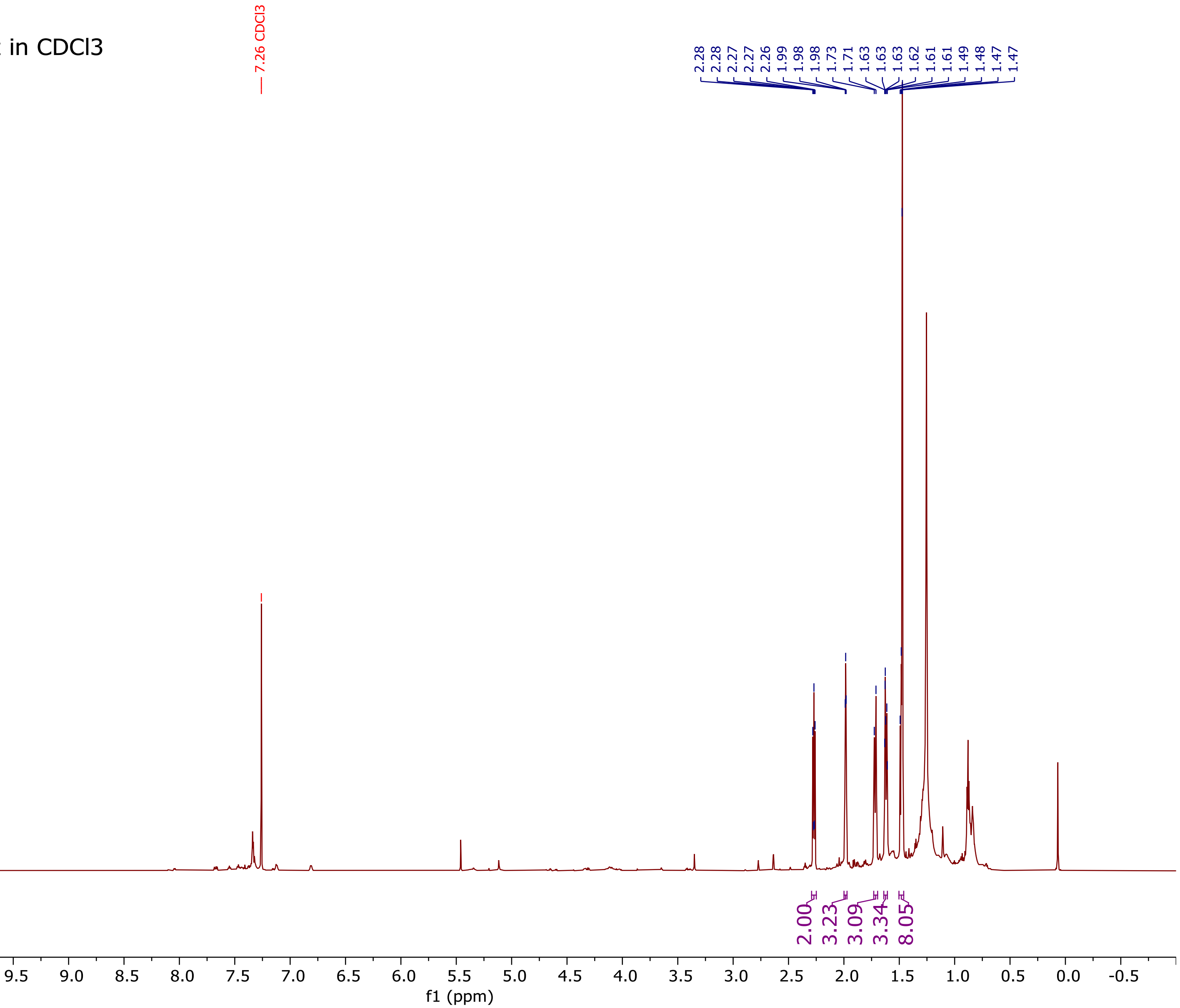

[33f]
13C NMR at 201.27 MHz in CDCl3

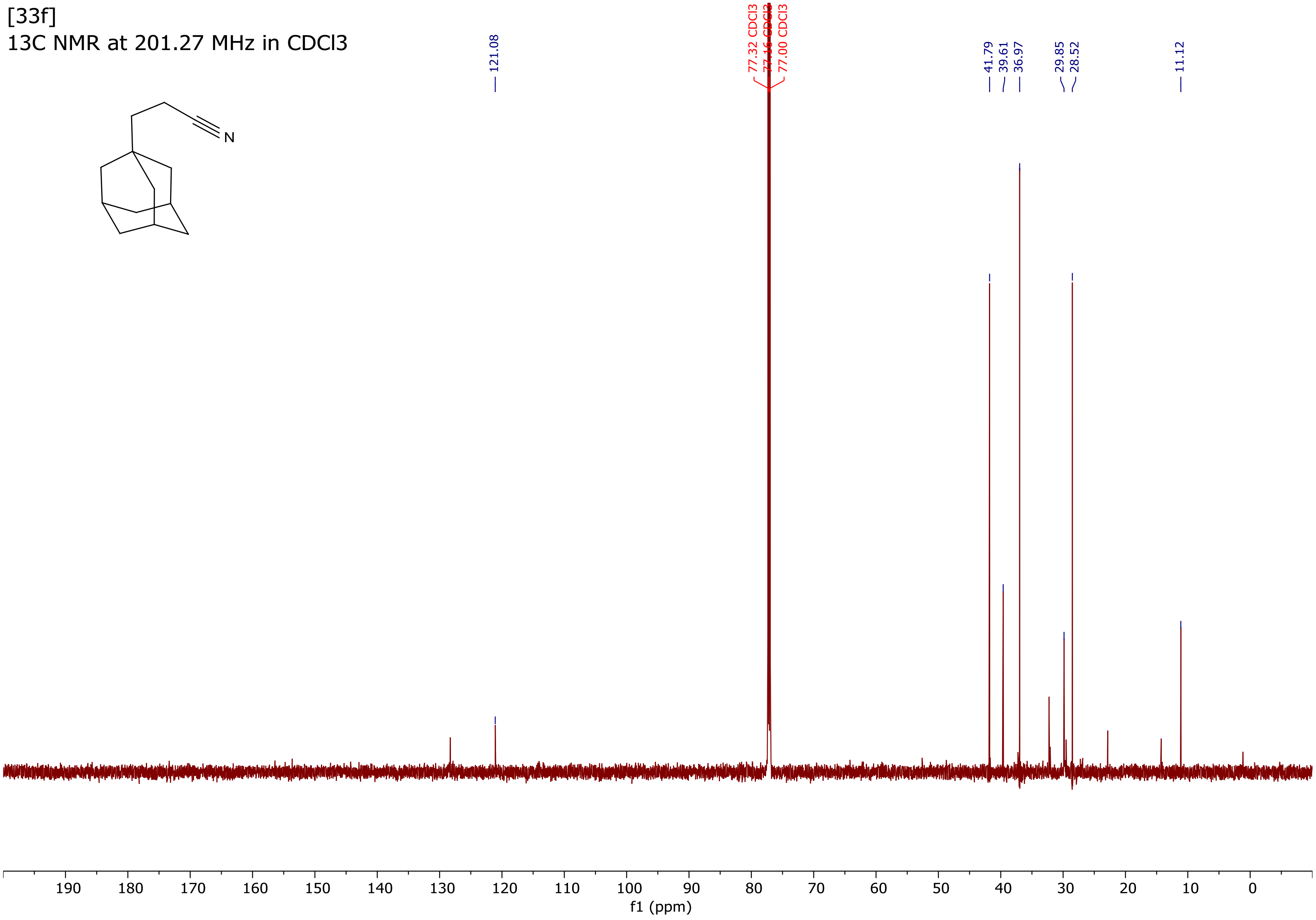

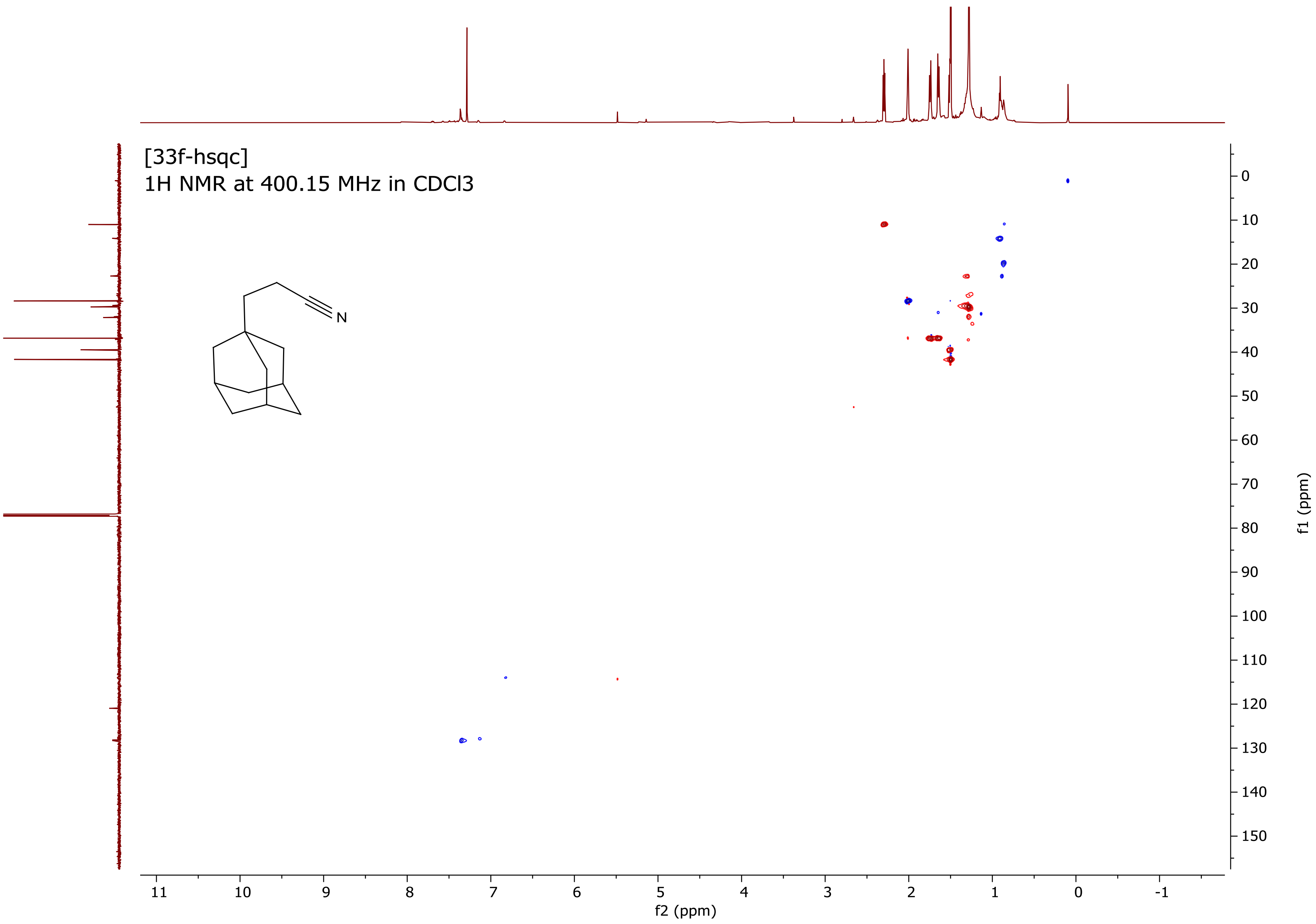

[33f-hsqc]
1H NMR at 400.15 MHz in CDCl3
N
f2 (ppm)
f1 (ppm)

[34a]
1H NMR at 400.15 MHz in CDCl3

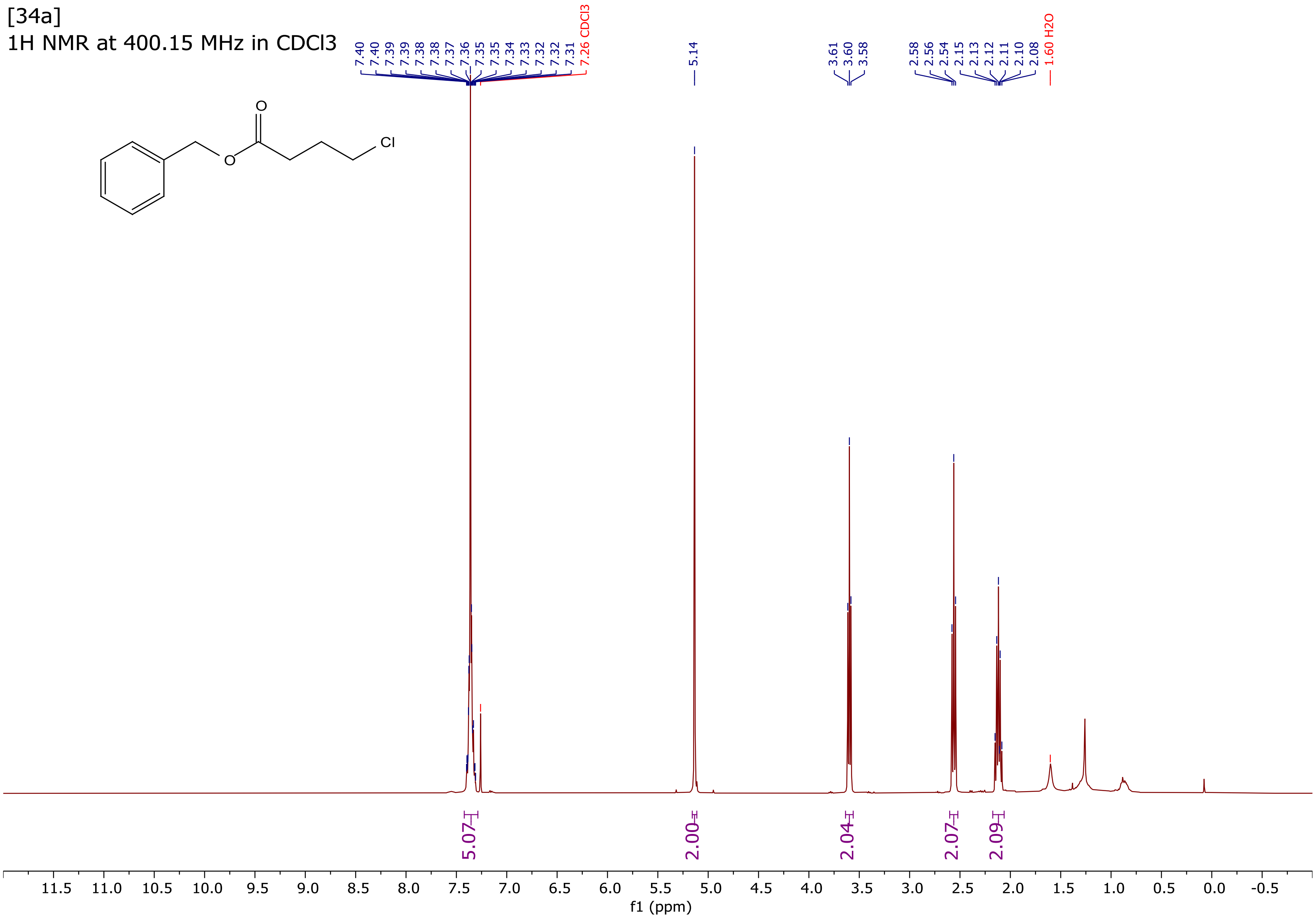

[34a]
13C NMR at 201.27 MHz in CDCl3

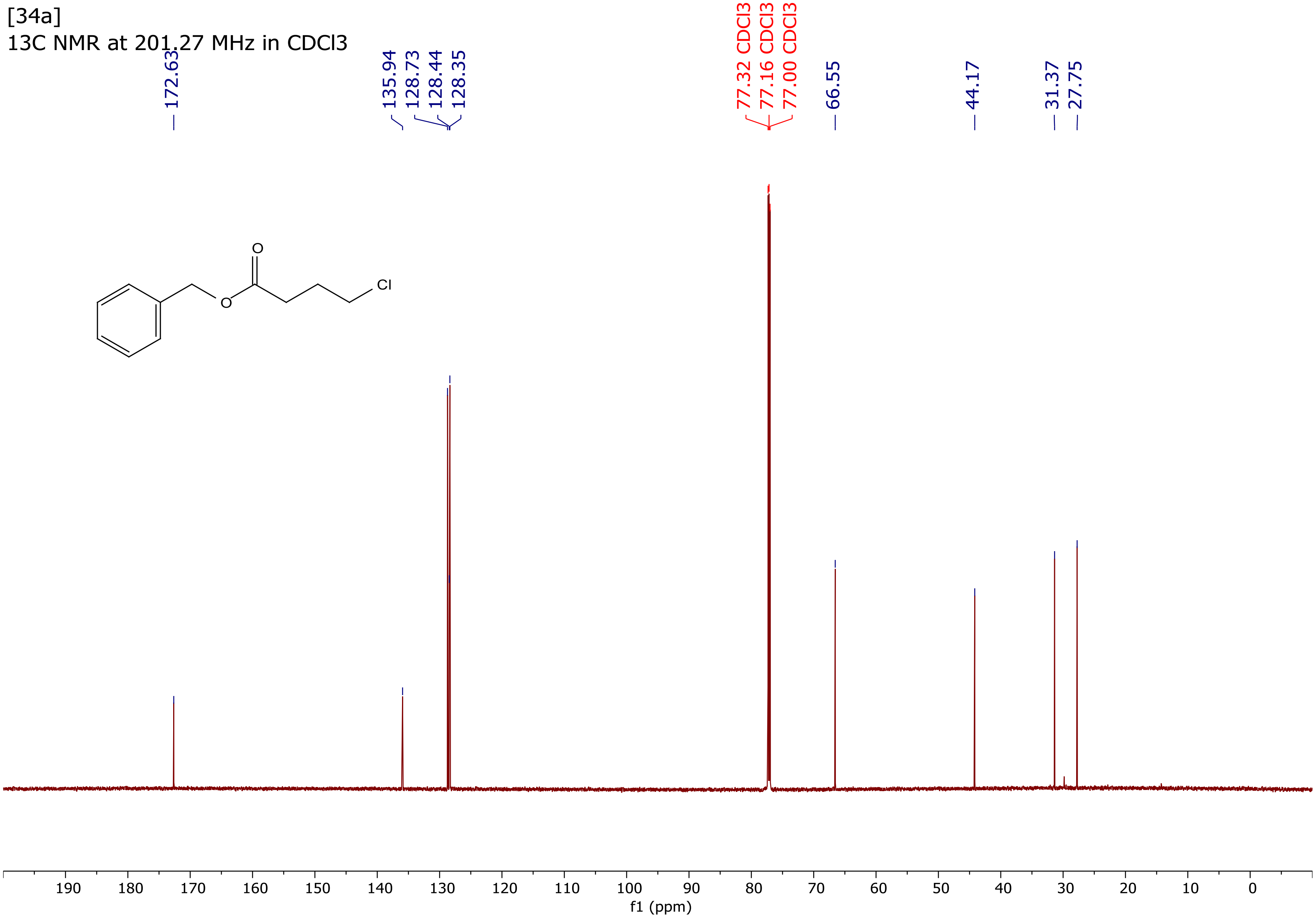

[35a]
1H NMR at 800.34 MHz in CDCl3

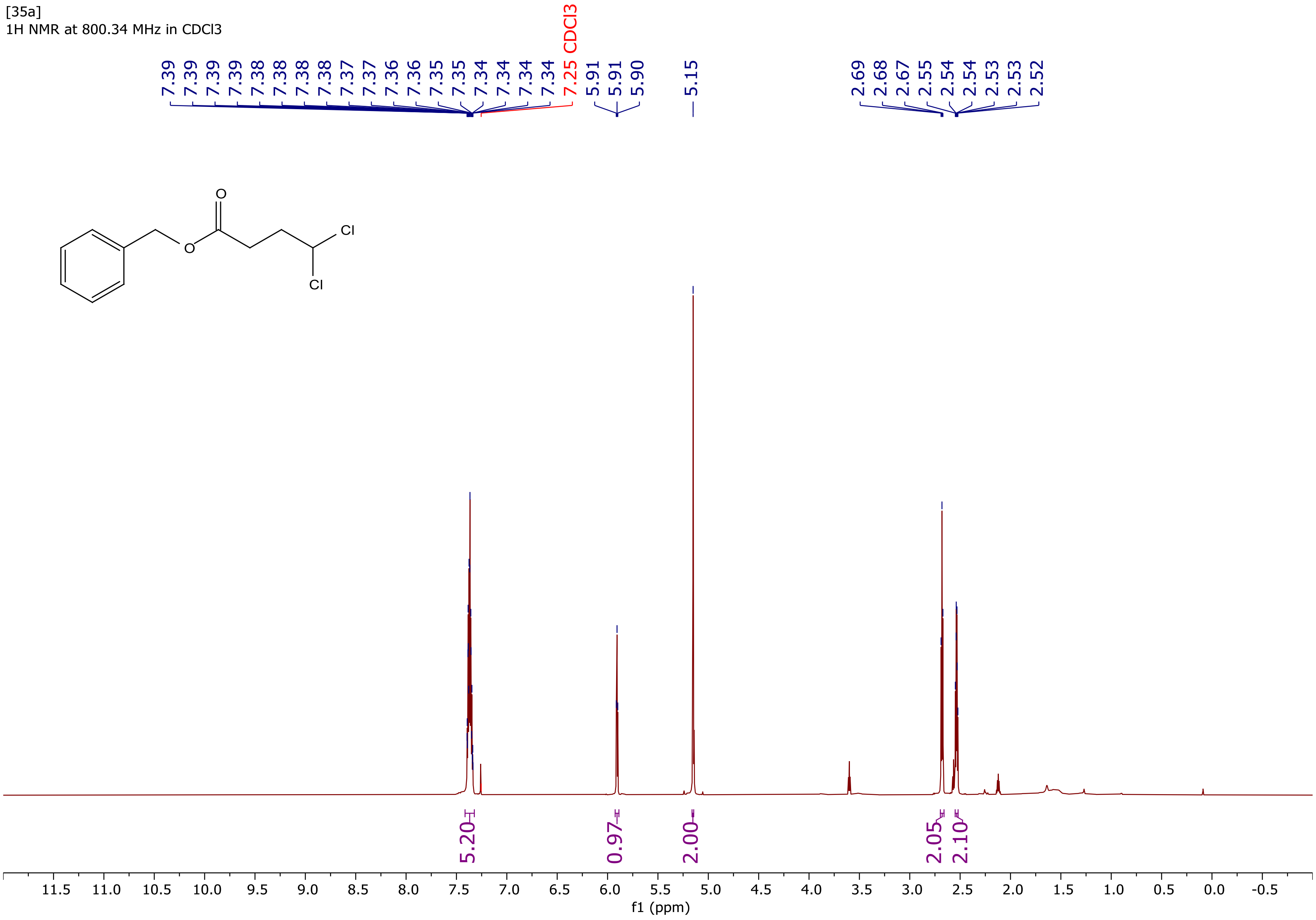

[35a]
13C NMR at 201.27 MHz in CDCl3

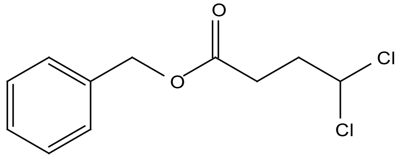

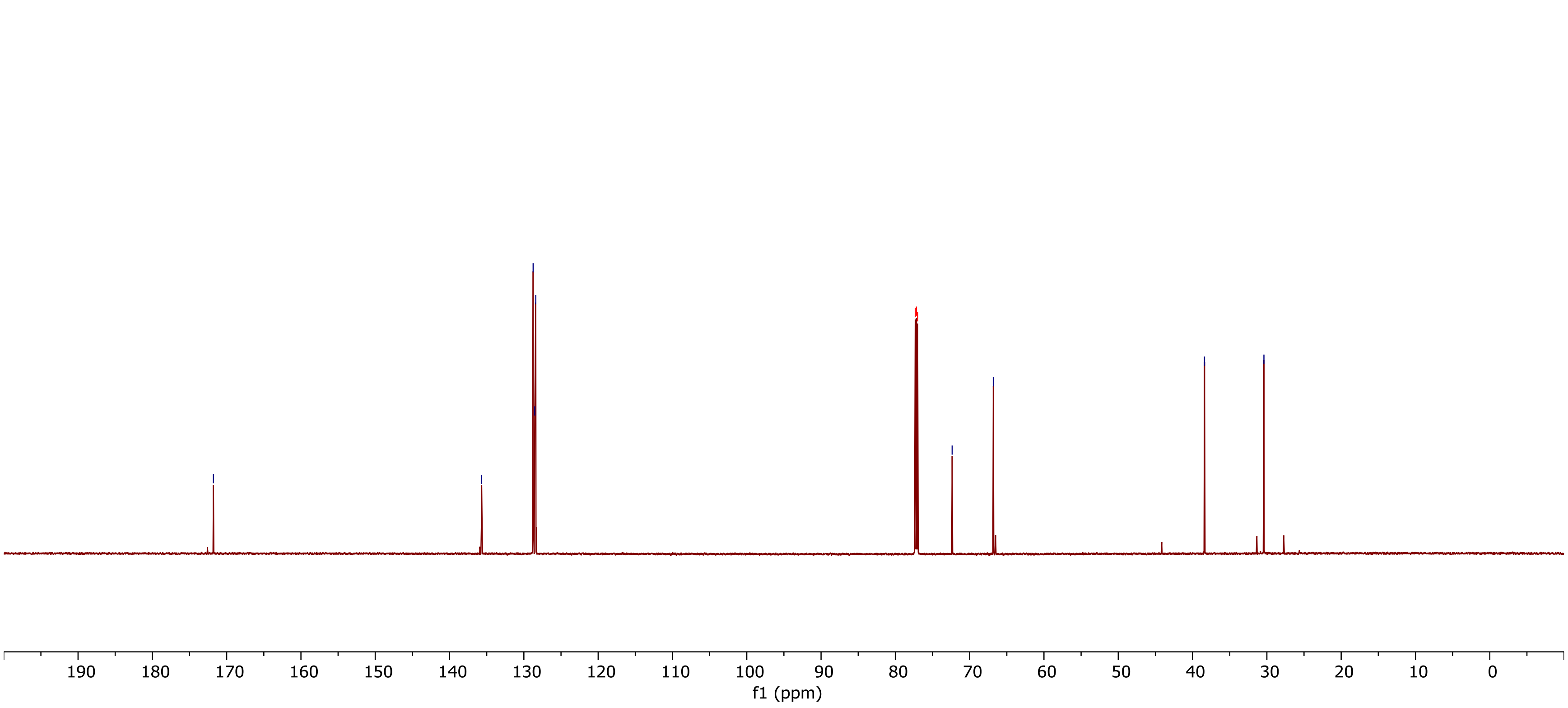

GCMS of benzyl 4,4-dichlorobutanoate

Chromatogram rh-3271-col C:\GCMSsolution\Data\Project1\Weaver\Roshini\rh-3271-col.qgd

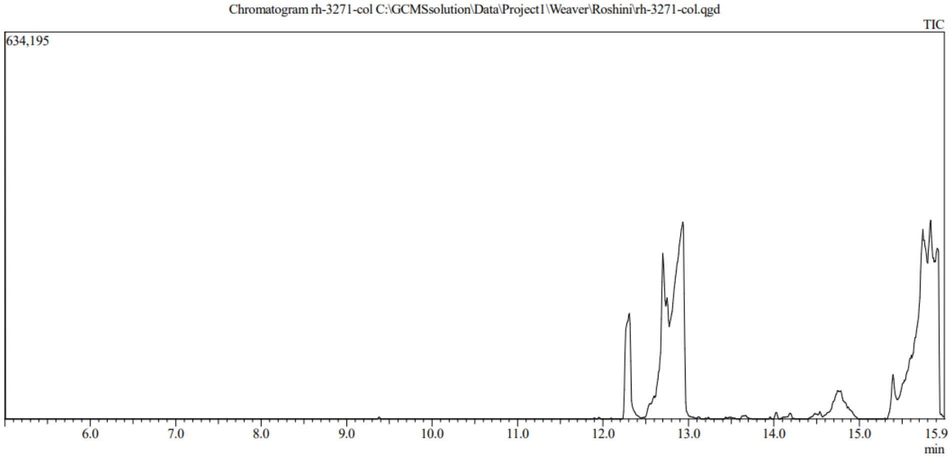

Peak Report TIC

Peak#	R.Time	I.Time	F.Time	Area	Area%	Height	Height%	A/H	Mark	Name
				0	0.00	0	0.00			

Line#:2 R.Time:12.725(Scan#:928)
MassPeaks:12
RawMode:Single 12.725(928) BasePeak:213(72023)
BG Mode:None Group 1 - Event 1 Scan

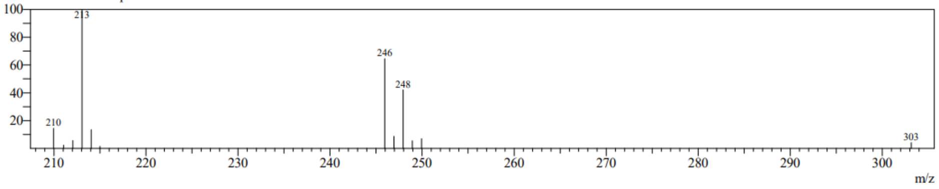

[36a]
1H NMR at 800.34 MHz in CDCl3

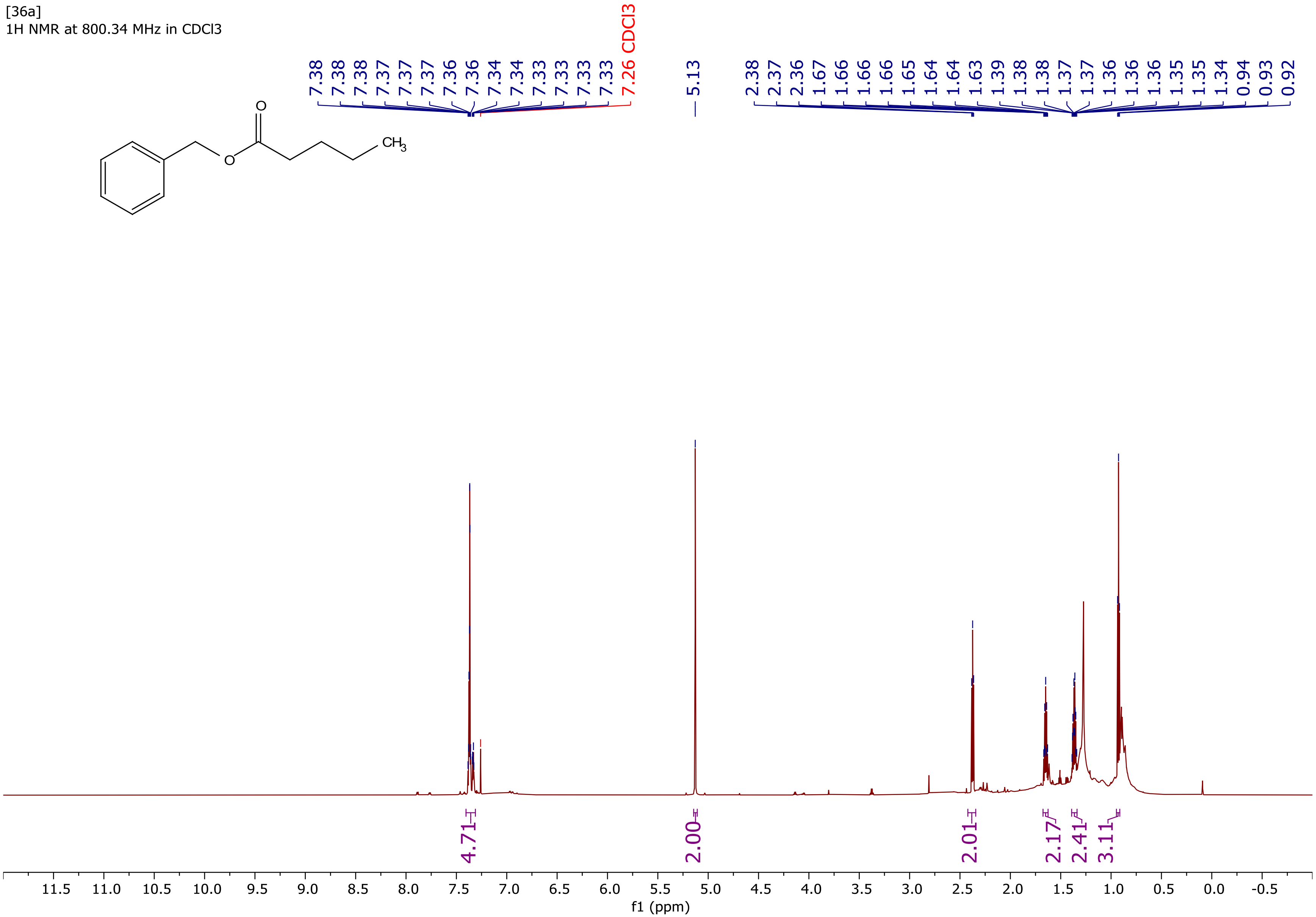

[36a]
13C NMR at 201.27 MHz in CDCl3

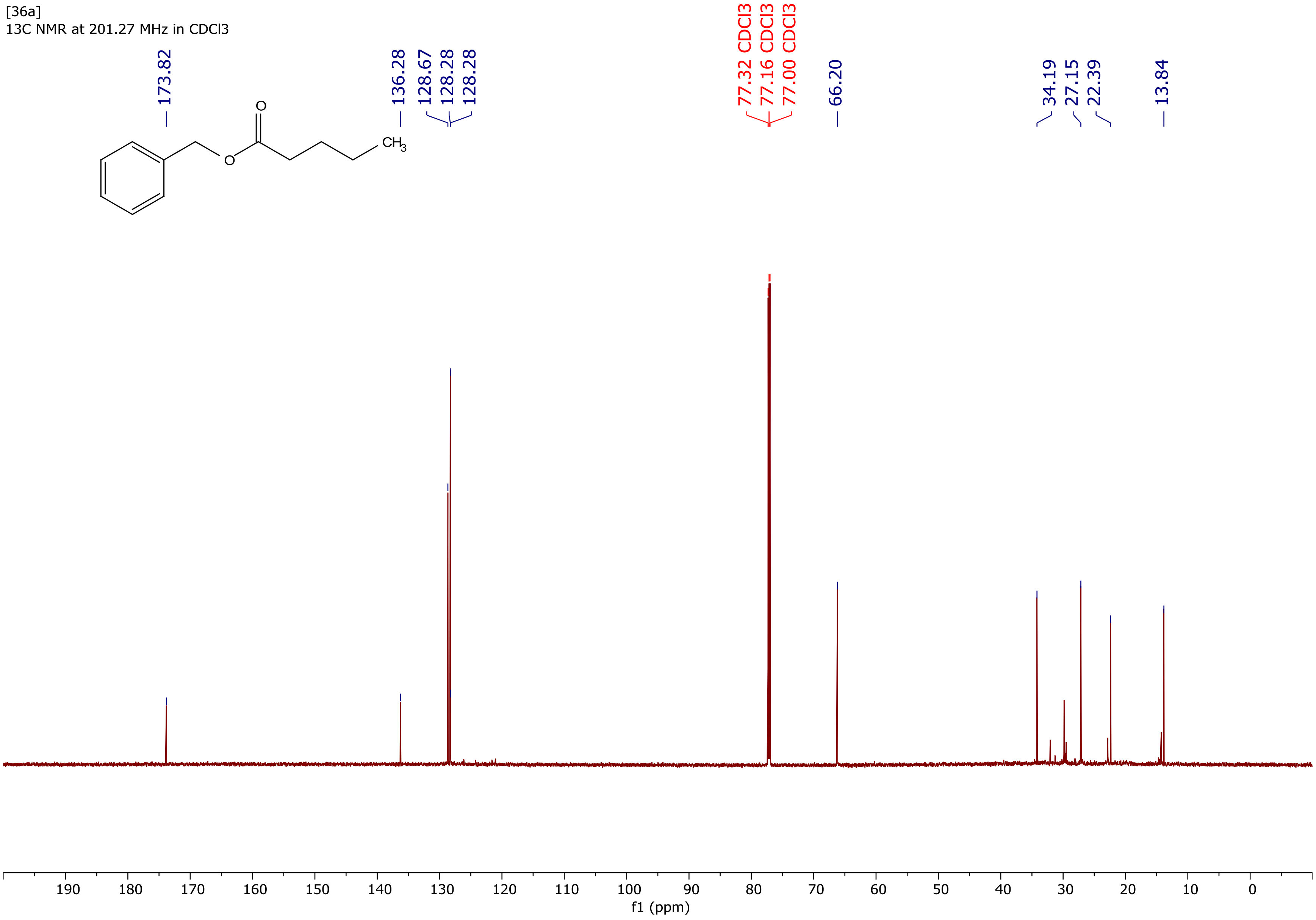

[36a-trifluoroacetate]
1H NMR at 400.15 MHz in CDCl3

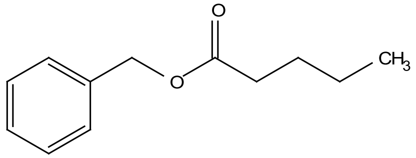

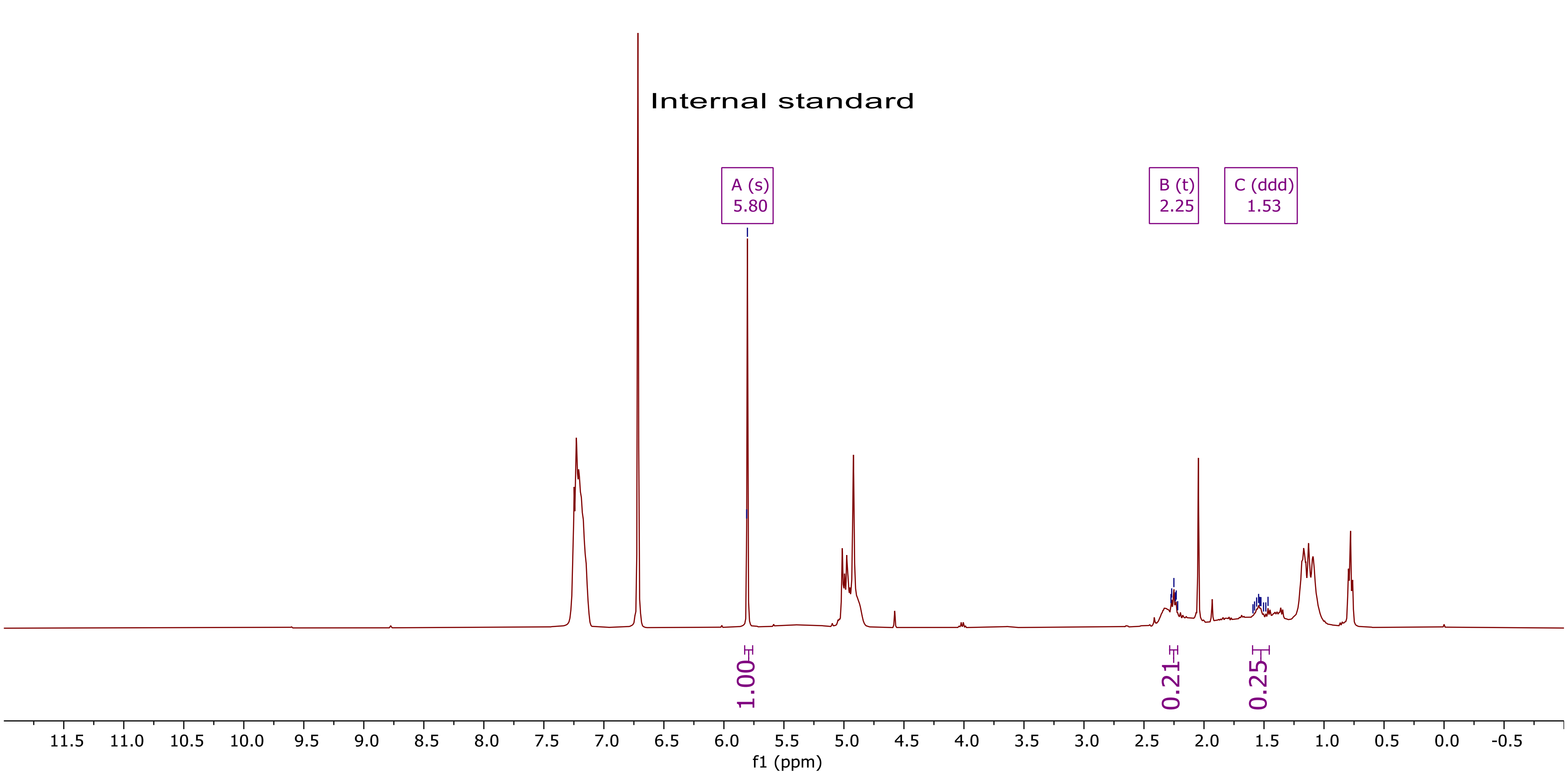

[17b]
1H NMR at 800.34 MHz in CDCl3

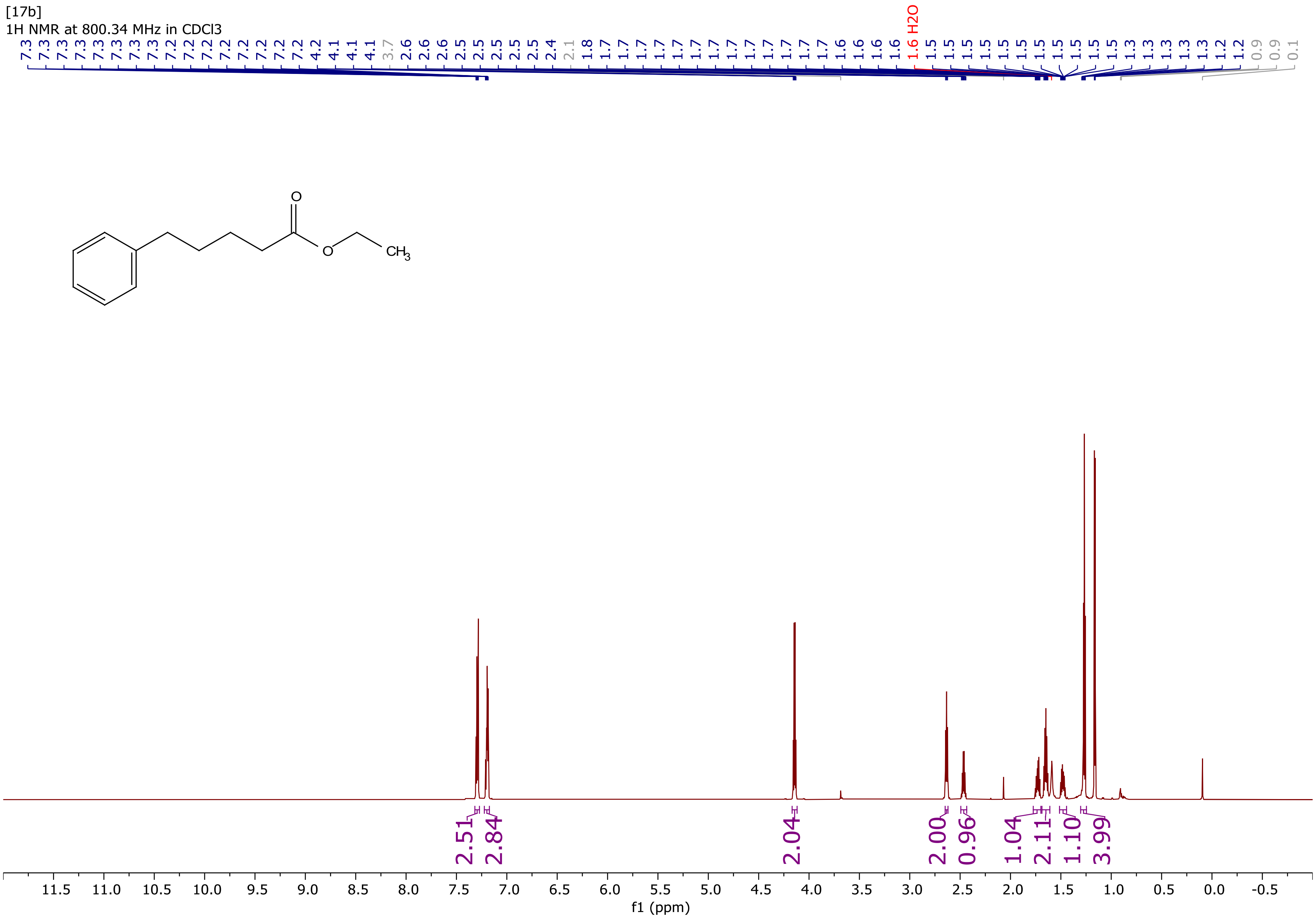

[17b]
13C NMR at 201.27 MHz in CDCl3

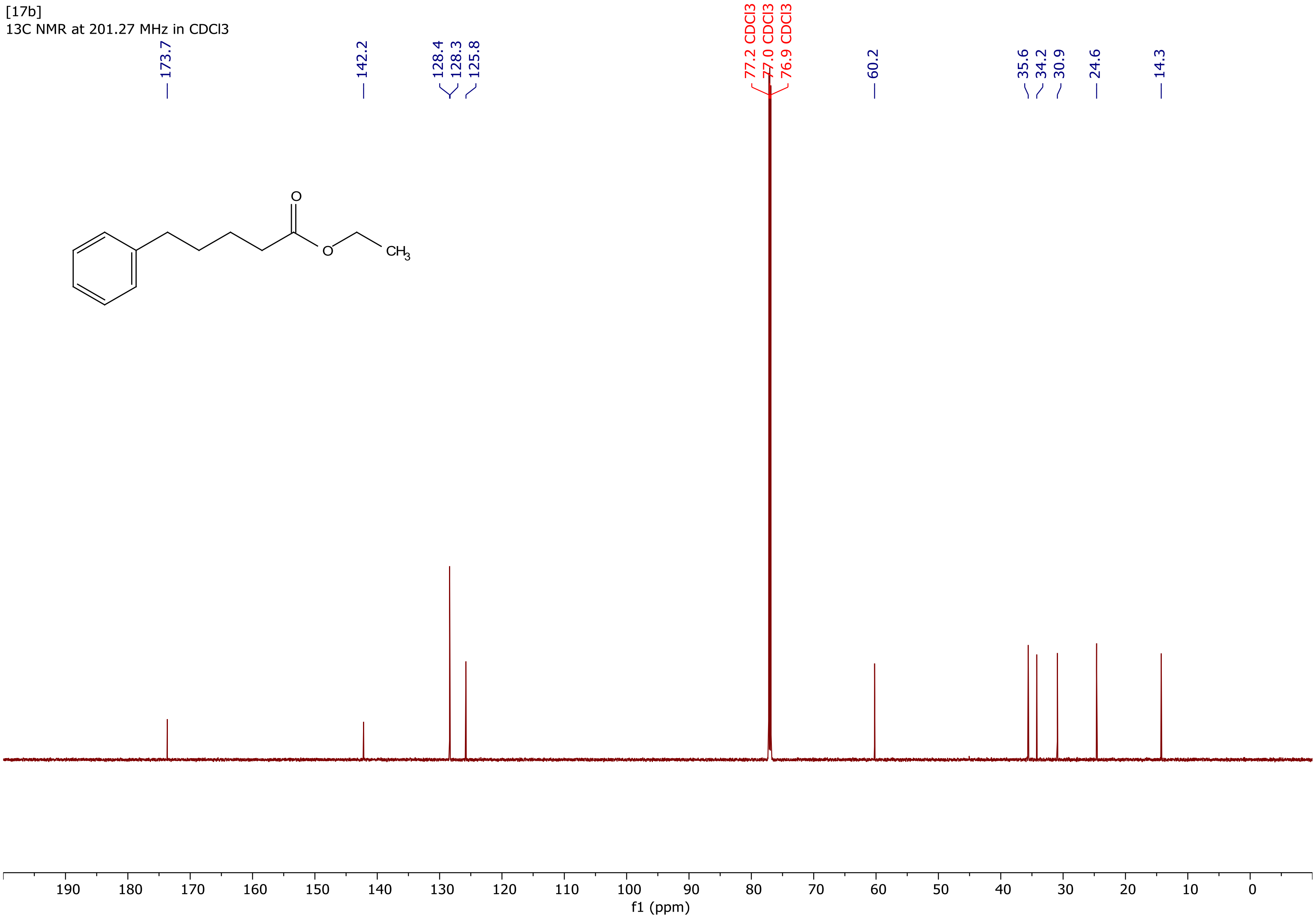

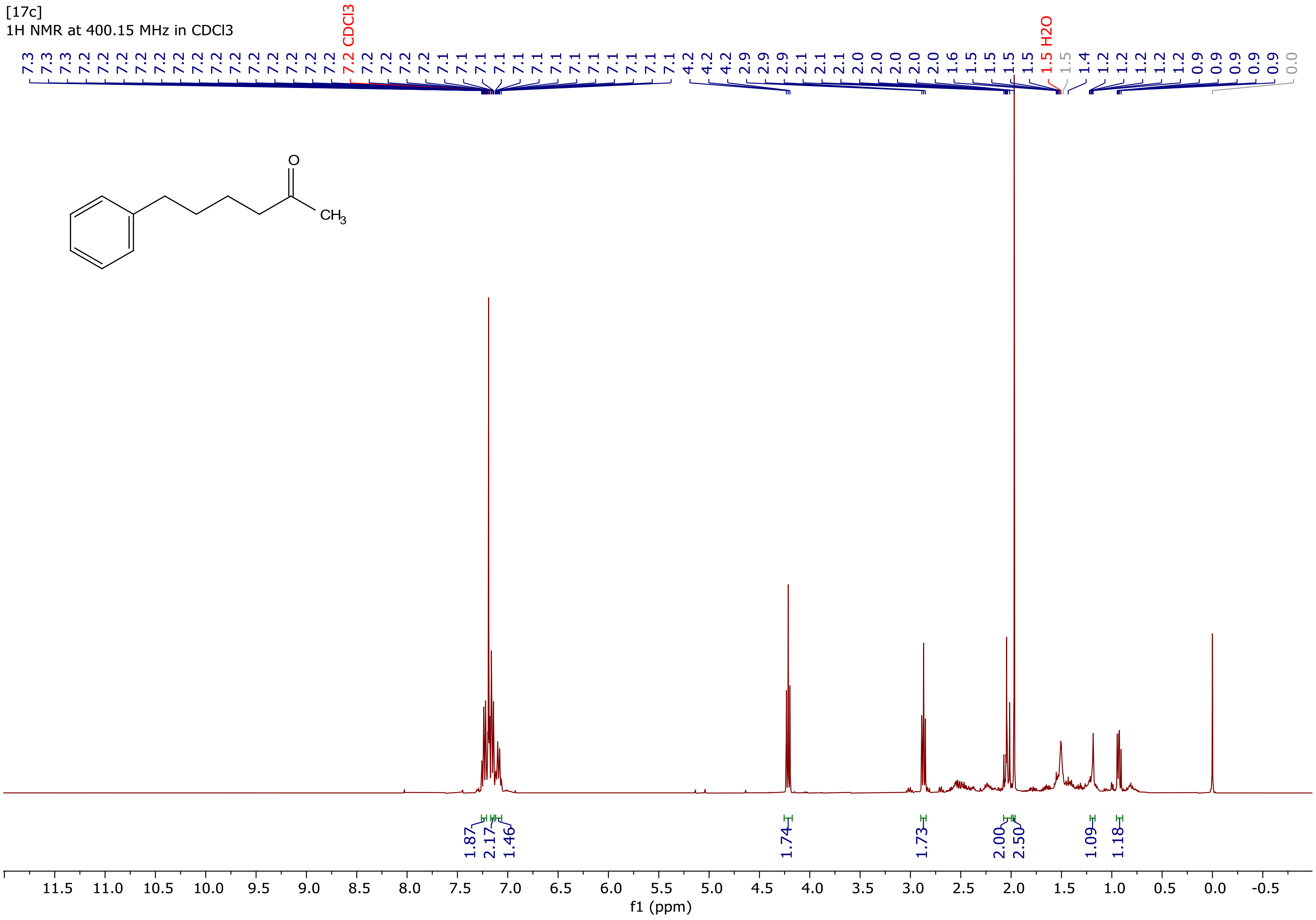
[17c]
1H NMR at 400.15 MHz in CDCl3
7.3 7.3 7.3 7.2 7.2 7.2 7.2 7.2 7.2 7.2 7.2 7.2 7.2 7.2 7.2 7.2 7.2 CDCl3 7.2 7.2 7.2 7.2 7.1 7.1 7.1 7.1 7.1 7.1 7.1 7.1 7.1 7.1 7.1 7.1 7.1 4.2 4.2 4.2 2.9 2.9 2.9 2.1 2.1 2.1 2.0 2.0 2.0 2.0 2.0 1.6 1.5 1.5 1.5 1.5 1.5 H2O 1.5 1.4 1.2 1.2 1.2 1.2 1.2 0.9 0.9 0.9 0.9 0.9 0.0
O
CH3
1.87
2.17
1.46
1.74
1.73
2.00
2.50
1.09
1.18
11.5 11.0 10.5 10.0 9.5 9.0 8.5 8.0 7.5 7.0 6.5 6.0 5.5 5.0 4.5 4.0 3.5 3.0 2.5 2.0 1.5 1.0 0.5 0.0 -0.5
f1 (ppm)

[17c]
13C NMR at 201.27 MHz in CDCl3

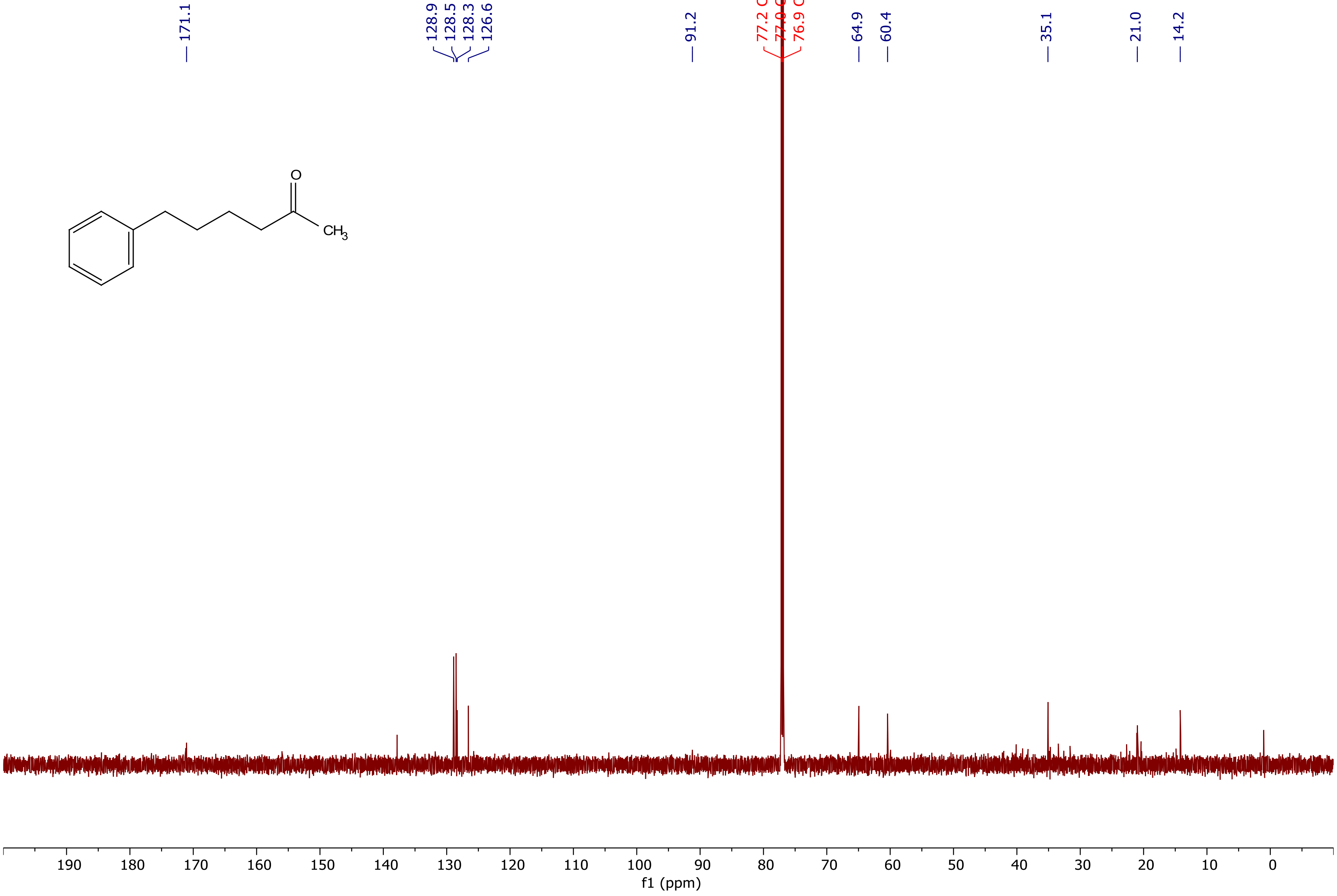

[17d]
1H NMR at 800.34 MHz in CDCl3

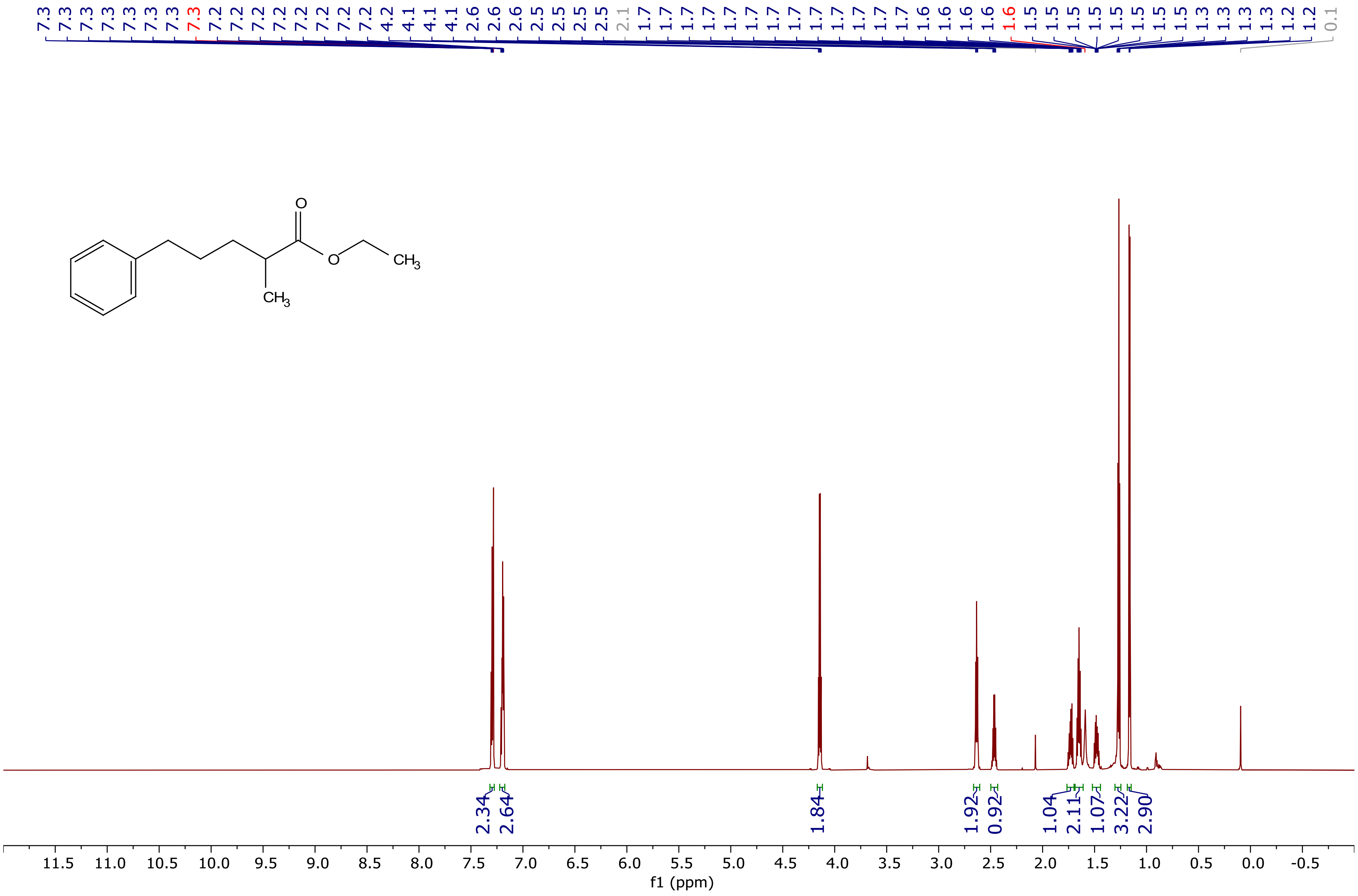

[17d]
13C NMR at 201.27 MHz in CDCl3

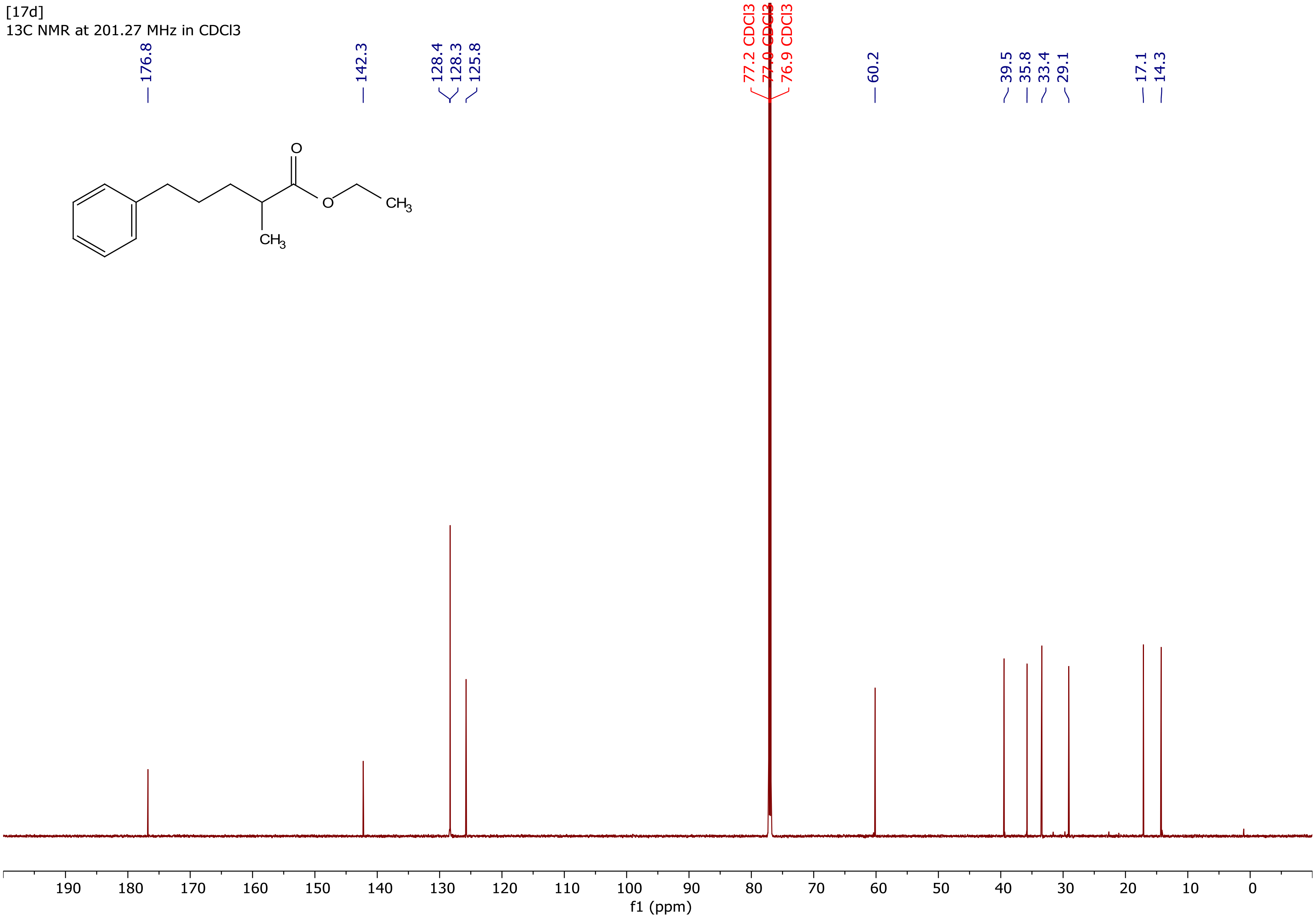

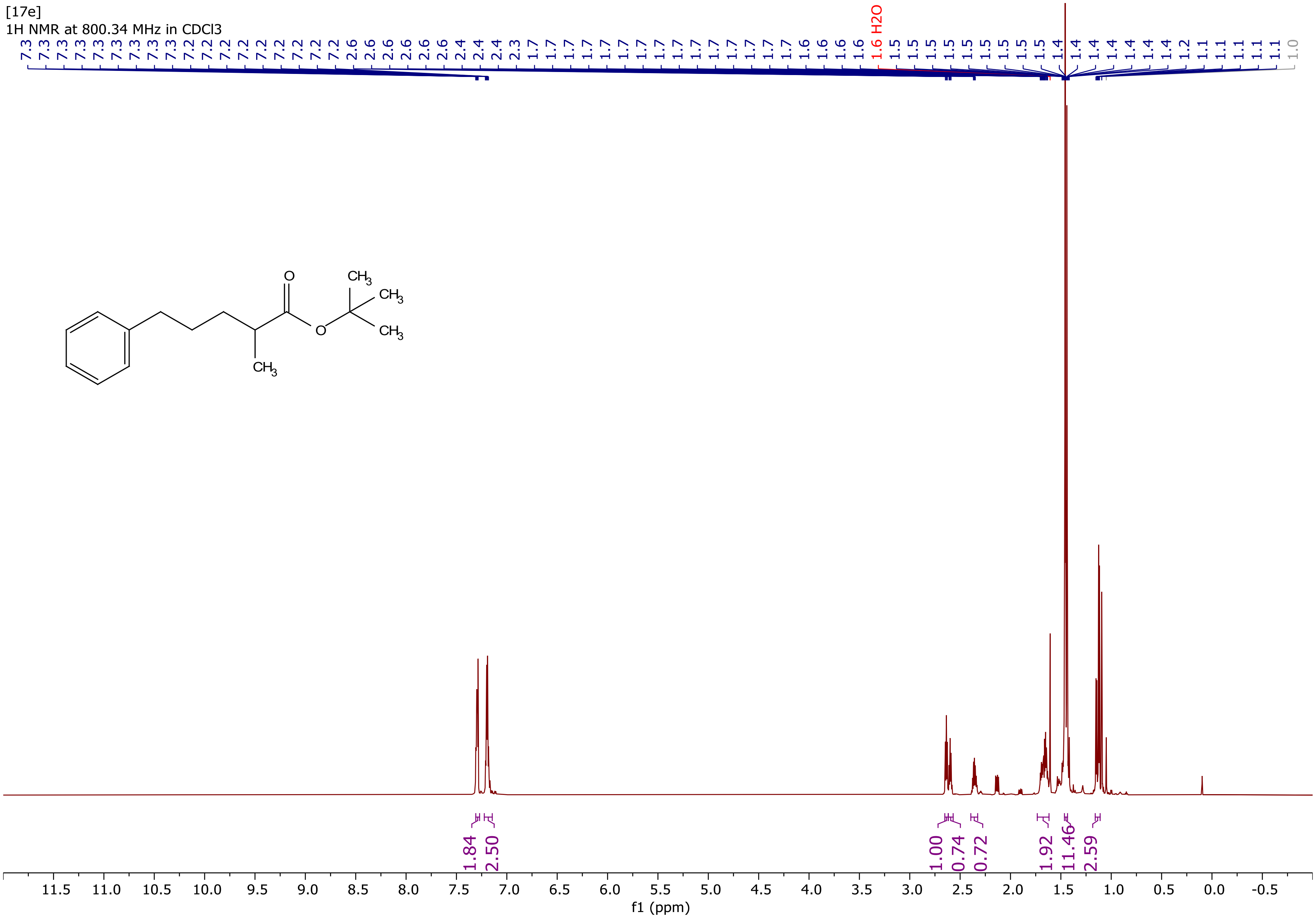
[17e]
1H NMR at 800.34 MHz in CDCl3
1.6 H2O
O
O
CH3
CH3
CH3
CH3
1.84
2.50
1.00
0.74
0.72
1.92
11.46
2.59
f1 (ppm)

[17e]
13C NMR at 201.27 MHz in CDCl3

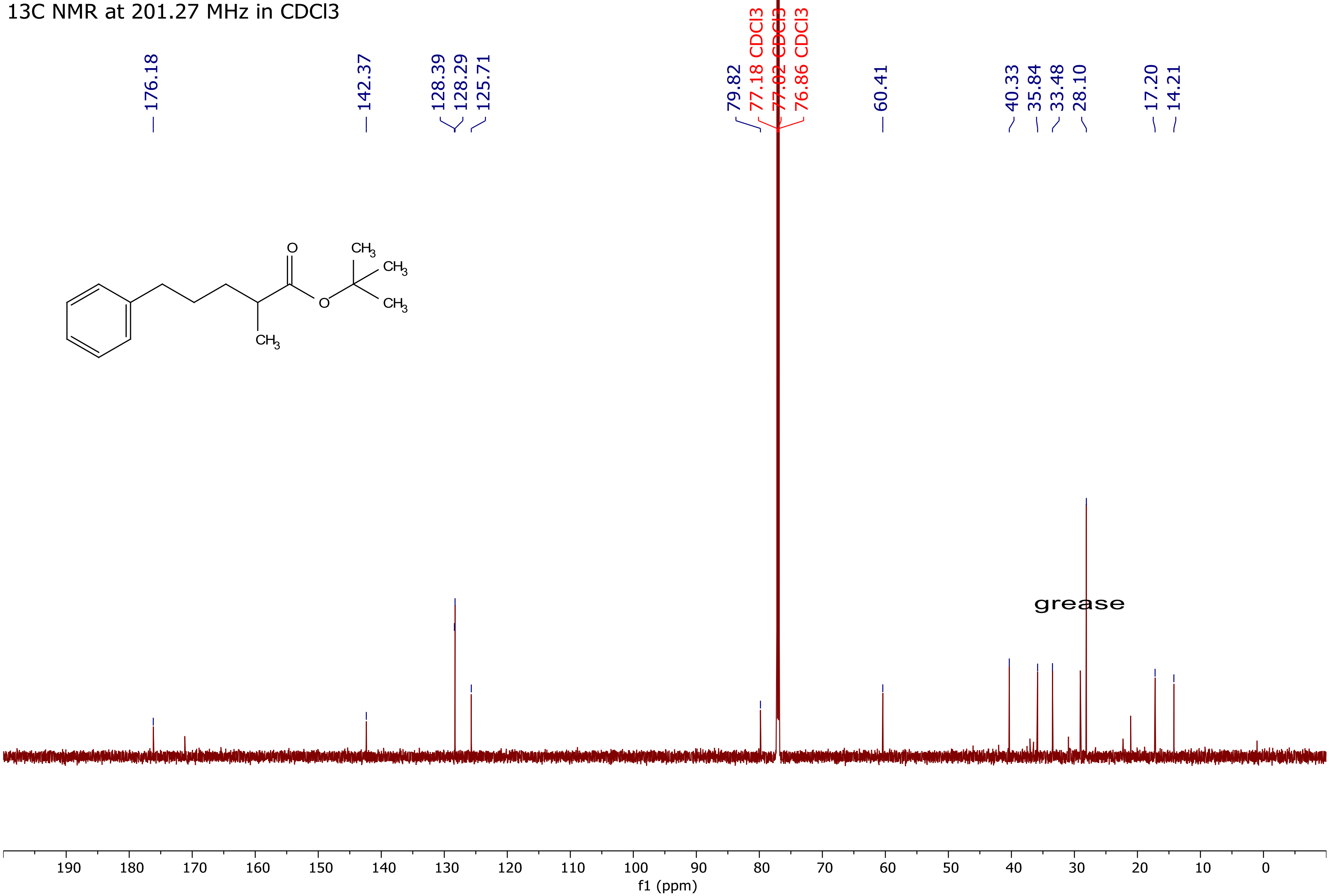

[37f]
1H NMR at 400.15 MHz in CDCl3

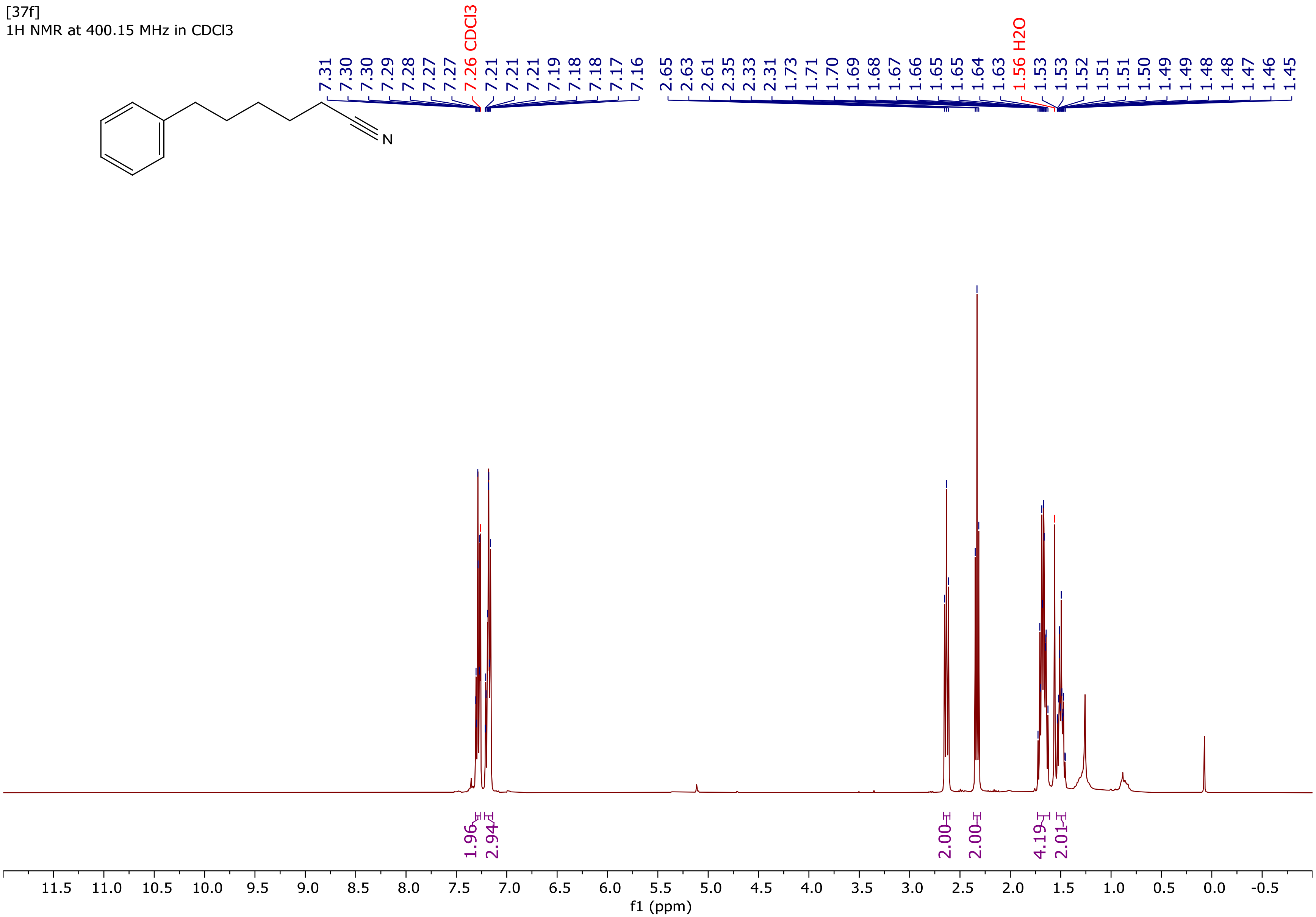

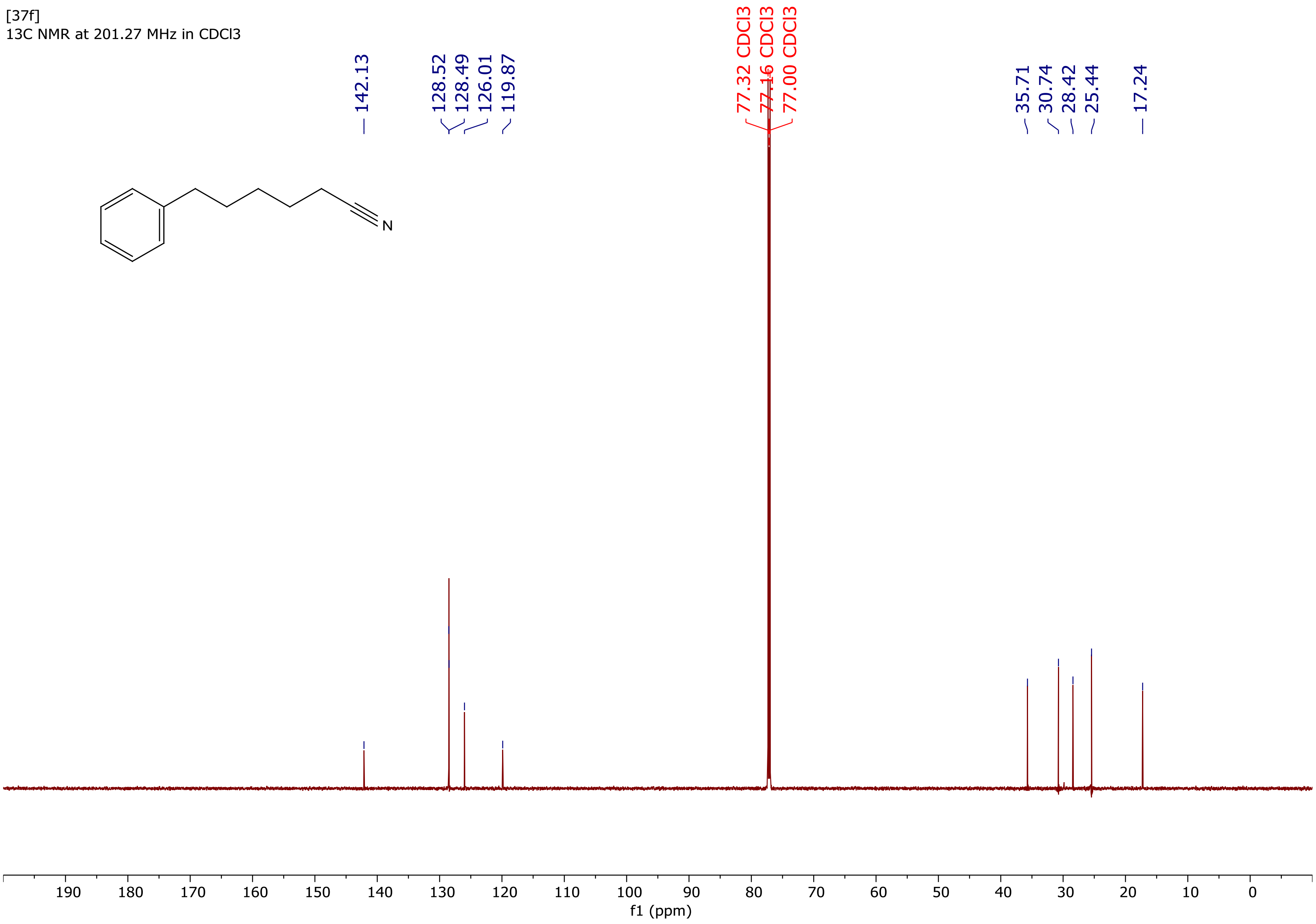
[37f]
13C NMR at 201.27 MHz in CDCl3
142.13
128.52
128.49
126.01
119.87
77.32 CDCl3
77.16 CDCl3
77.00 CDCl3
35.71
30.74
28.42
25.44
17.24
N
190 180 170 160 150 140 130 120 110 100 90 80 70 60 50 40 30 20 10 0
f1 (ppm)

[17g]
1H NMR at 800.34 MHz in CDCl3

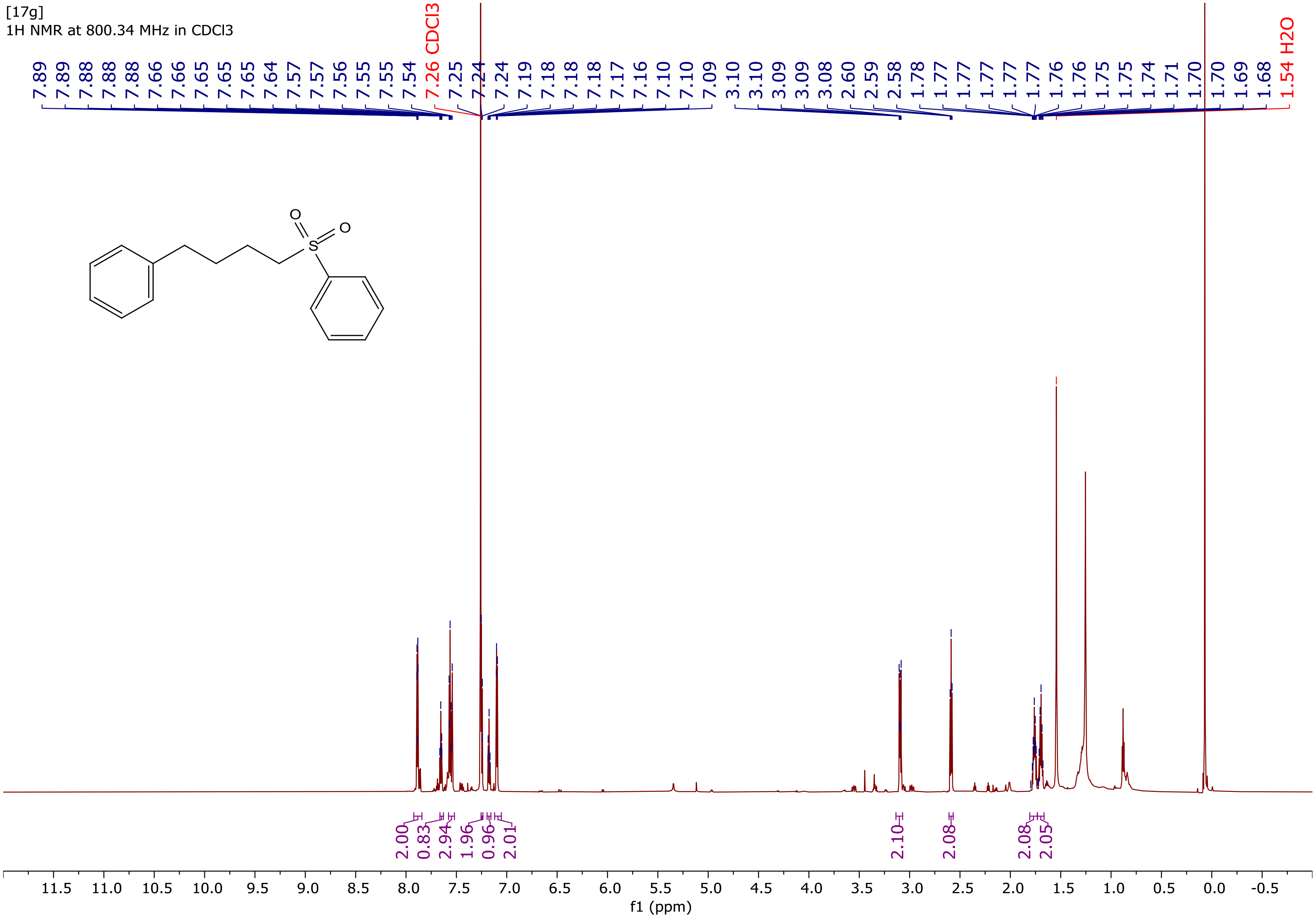

[17g]
13C NMR at 201.27 MHz in CDCl3

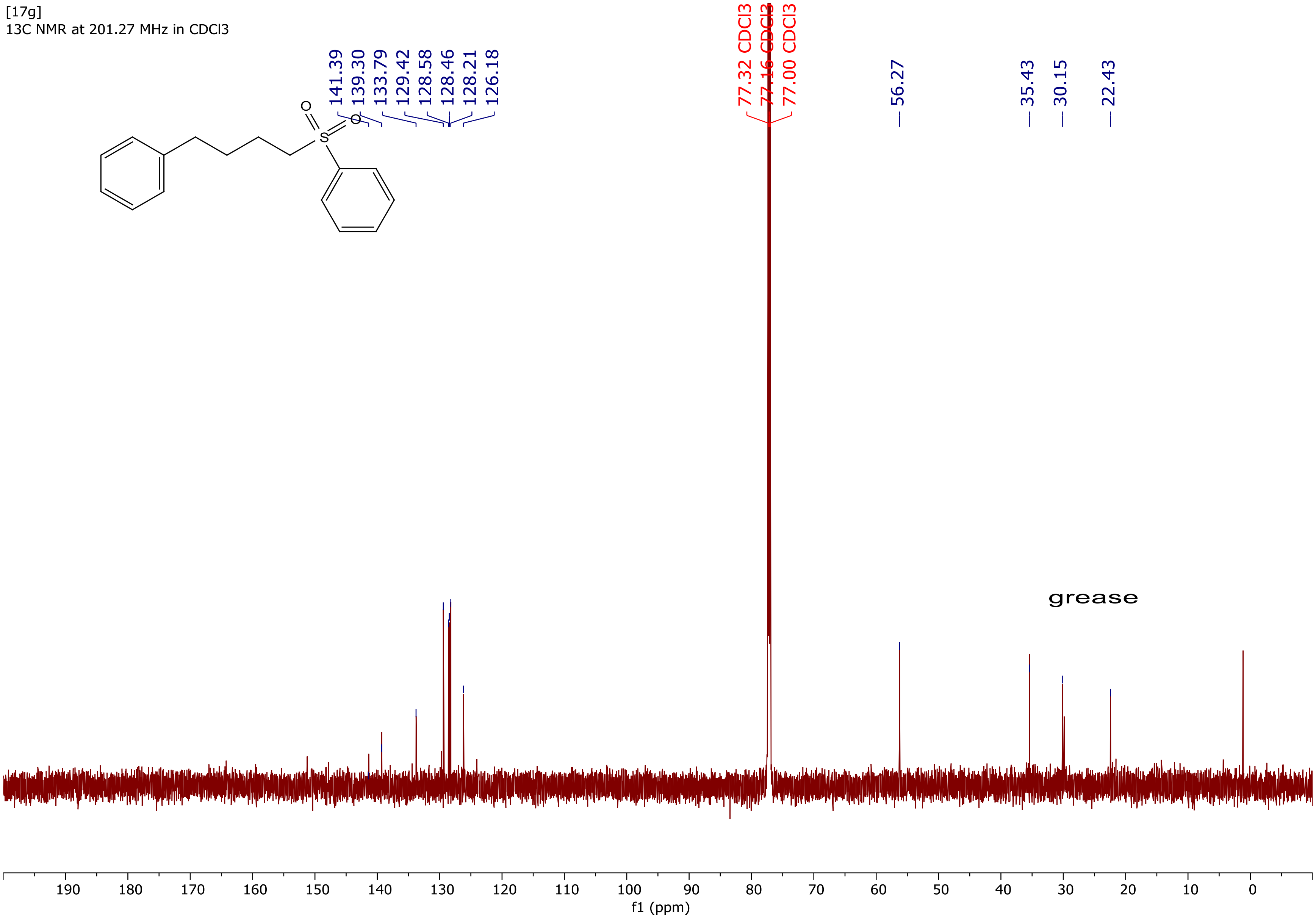

[17h]
1H NMR at 800.34 MHz in CDCl3

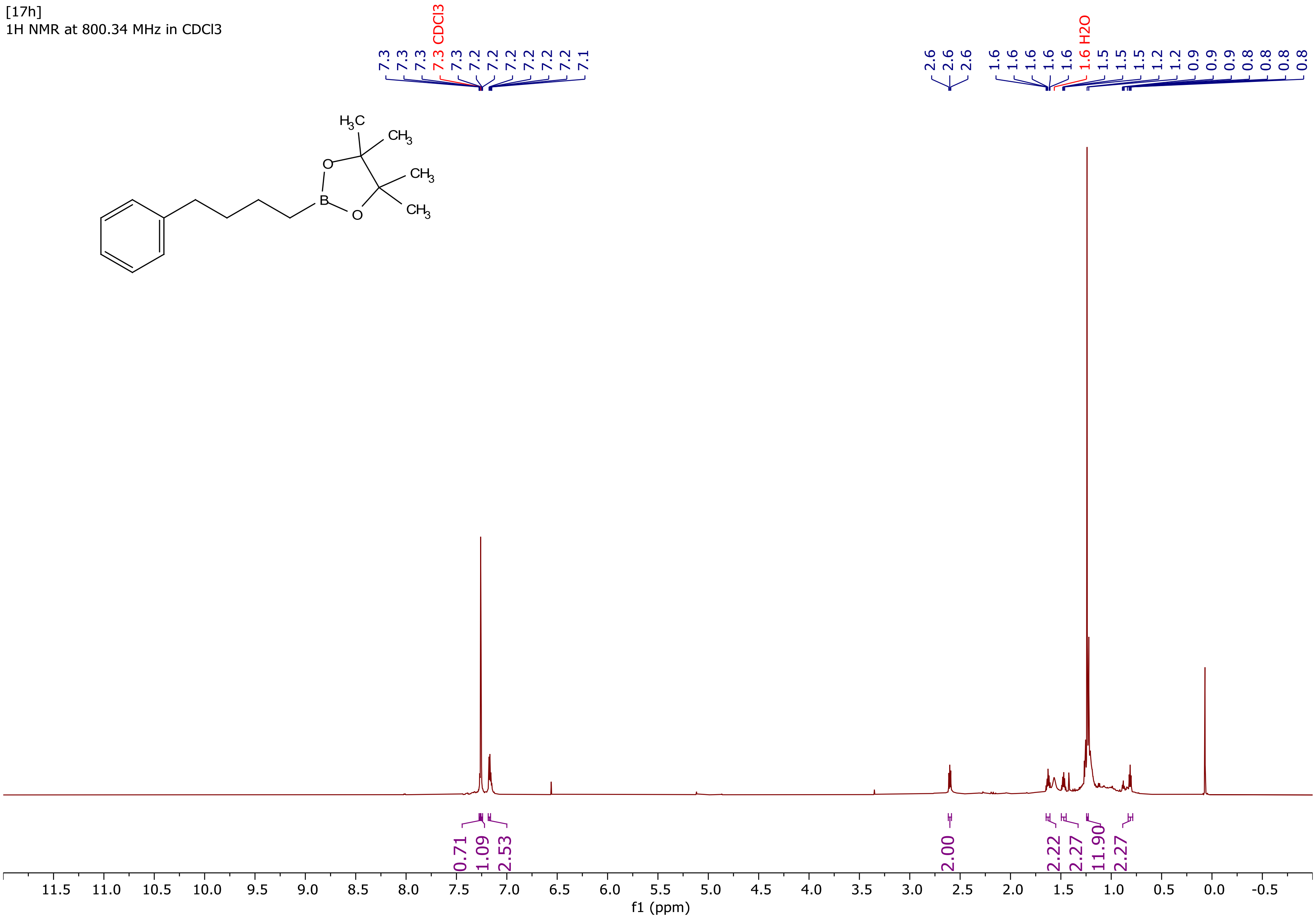

[17h]
13C NMR at 201.27 MHz in CDCl3

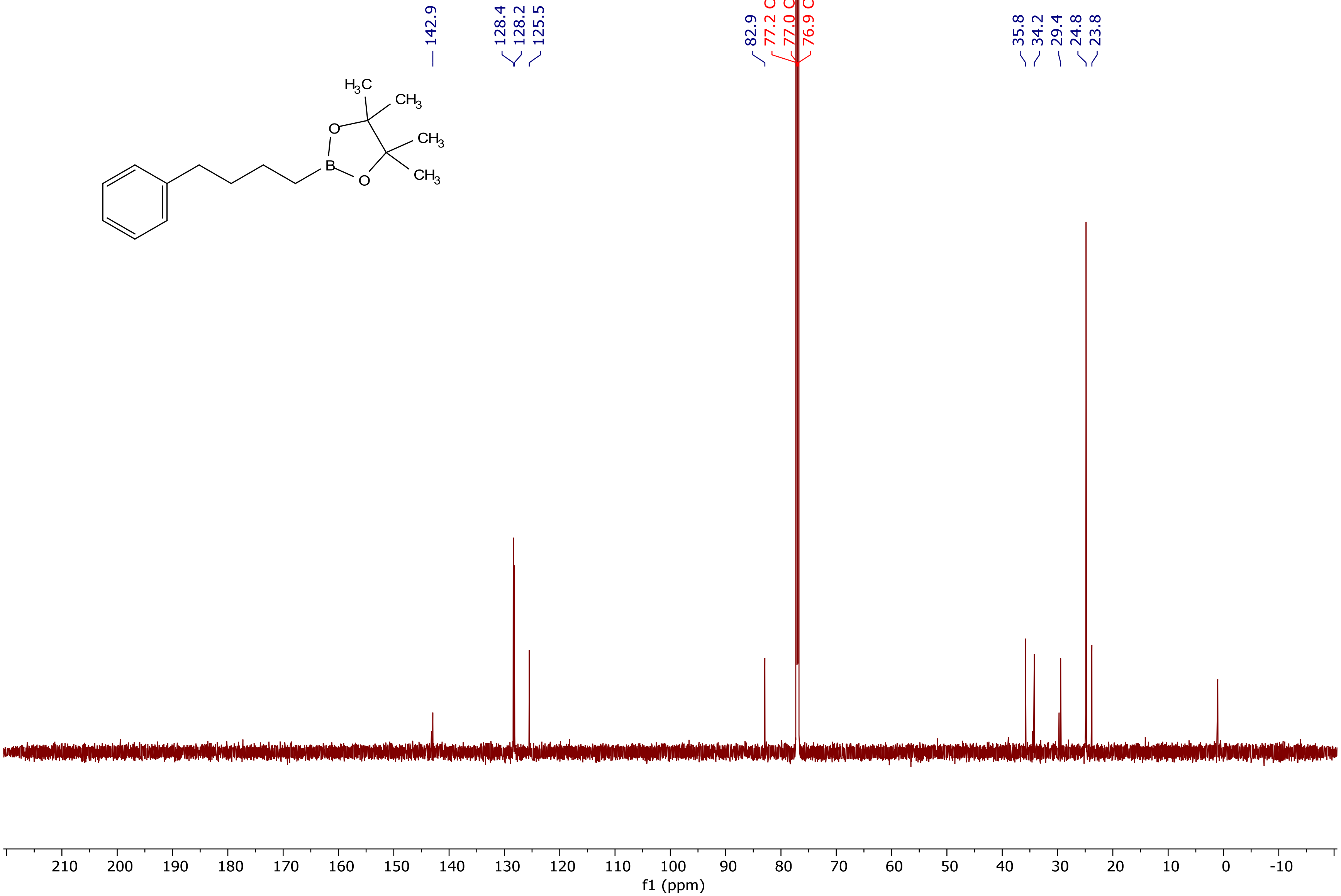

[17i]
1H NMR at 400.15 MHz in CDCl3

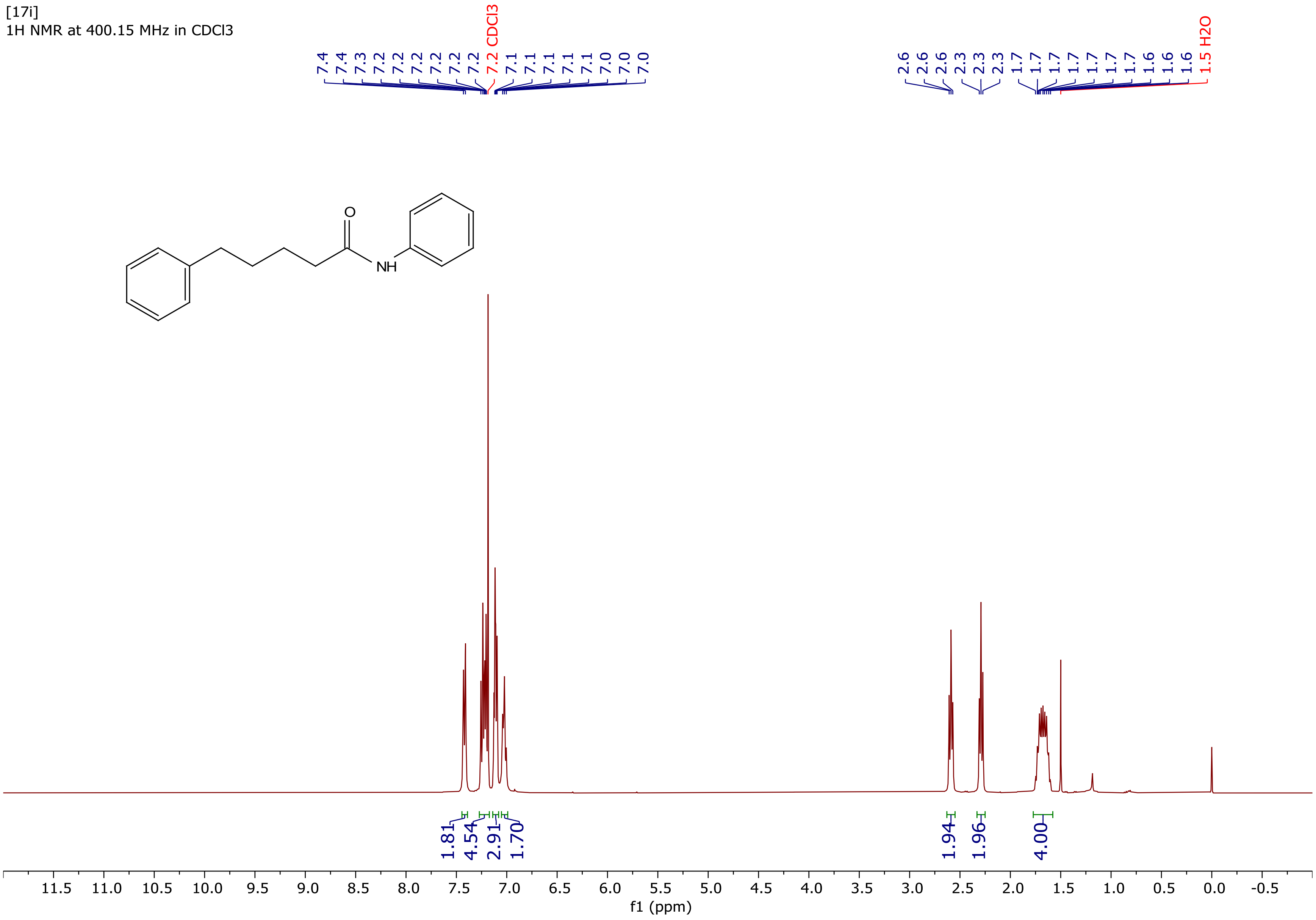

[17i]
13C NMR at 201.27 MHz in CDCl3

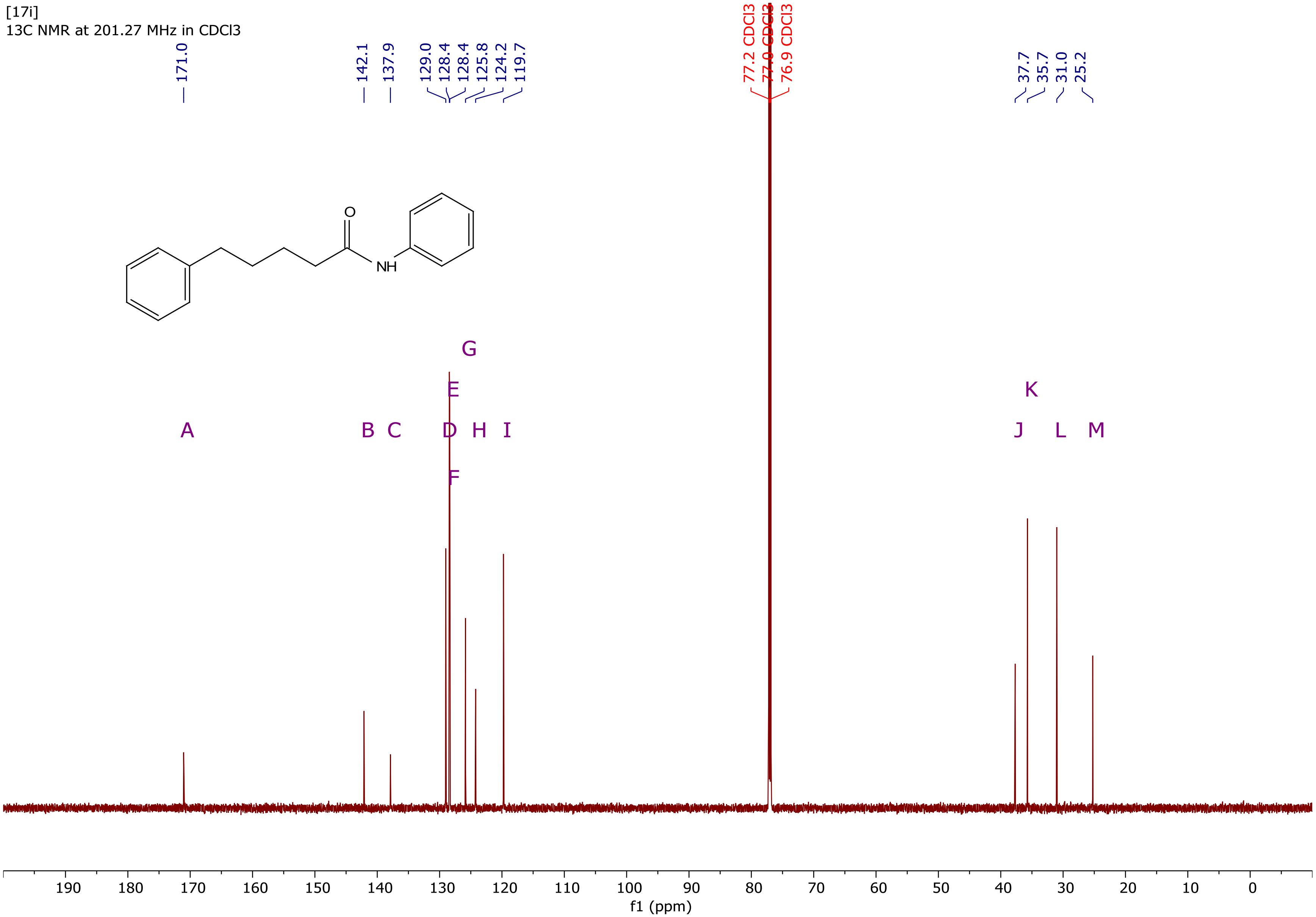

[17j]
1H NMR at 800.34 MHz in CDCl3

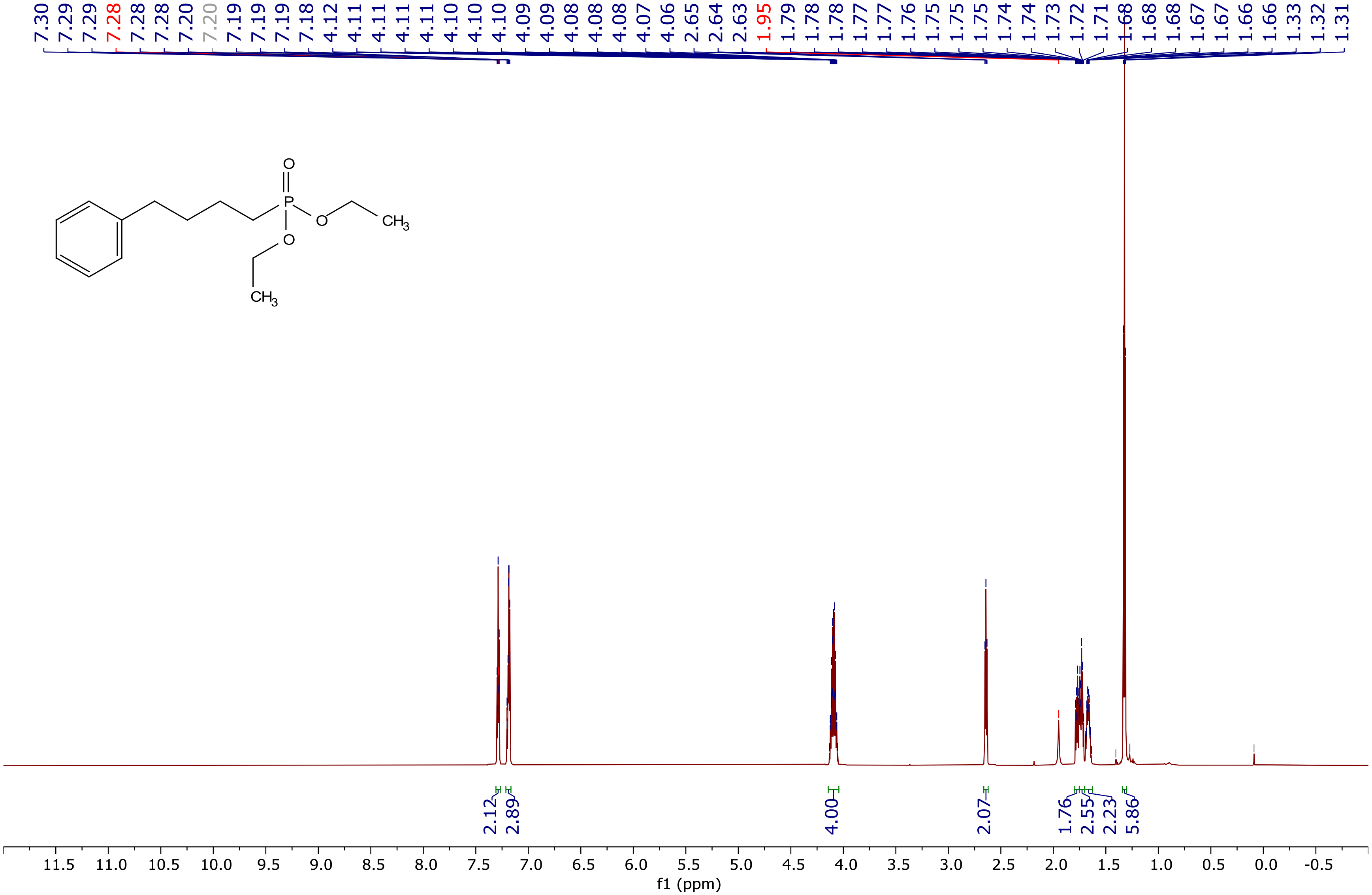

[17j]

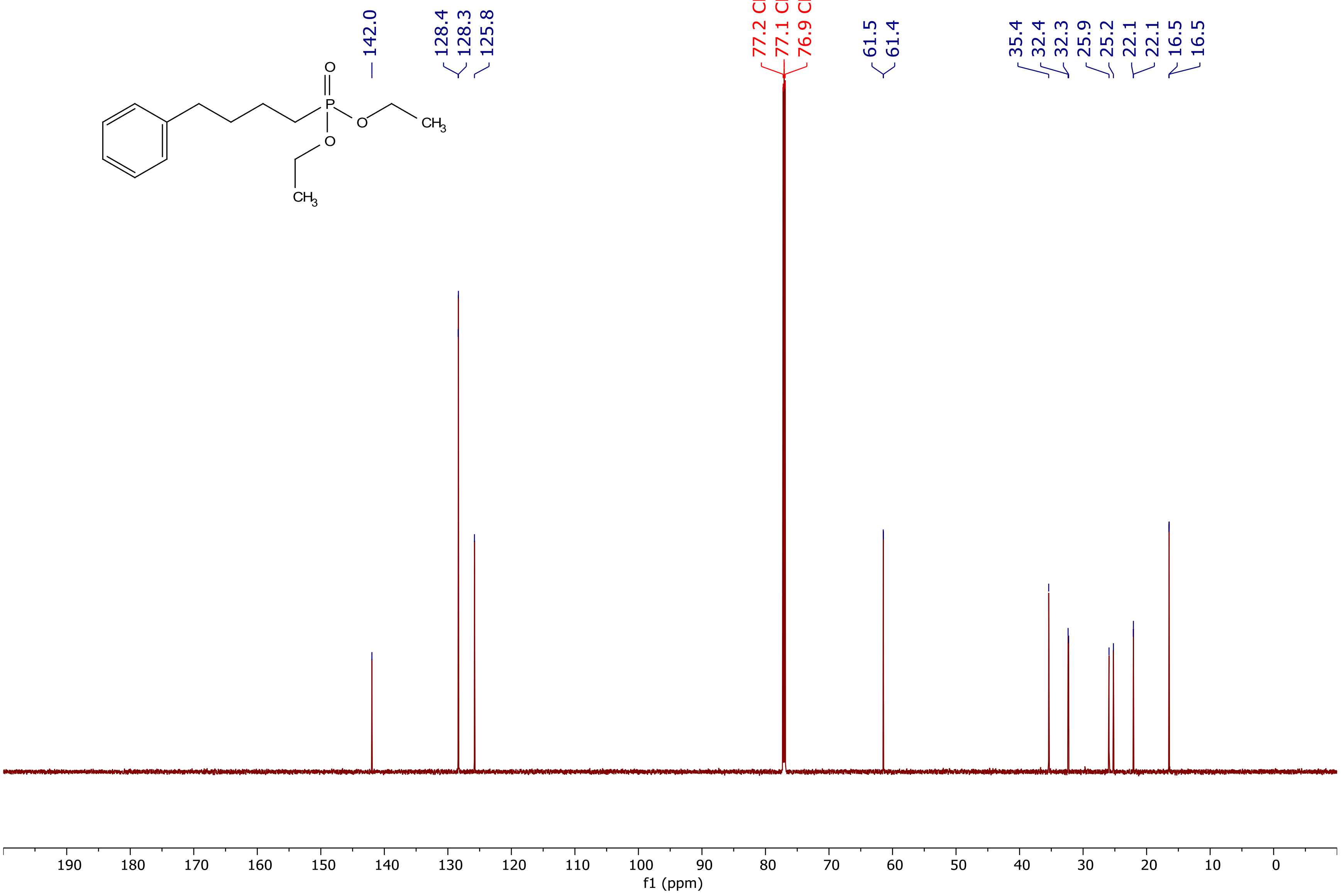

O
P
O
CH3
O
CH3
142.0
128.4
128.3
125.8
77.2 CDCl3
77.1 CDCl3
76.9 CDCl3
61.5
61.4
35.4
32.4
32.3
25.9
25.2
22.1
22.1
16.5
16.5
190
180
170
160
150
140
130
120
110
100
90
80
70
60
50
40
30
20
10
0
f1 (ppm)

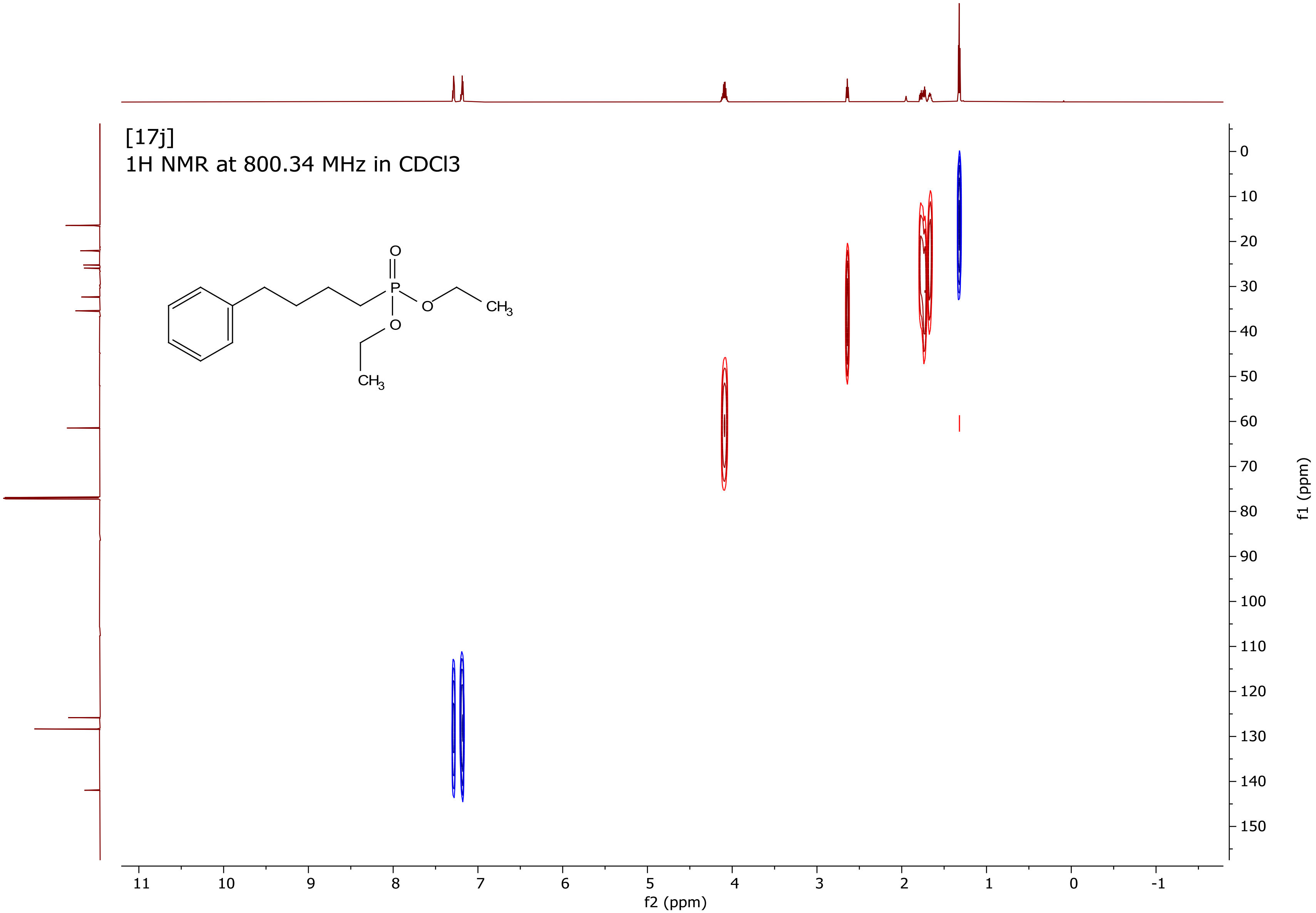
[17j]
1H NMR at 800.34 MHz in CDCl3
O
P
O
CH3
O
CH3
f2 (ppm)
f1 (ppm)

[13k]

1H NMR at 800.34 MHz in CDCl3

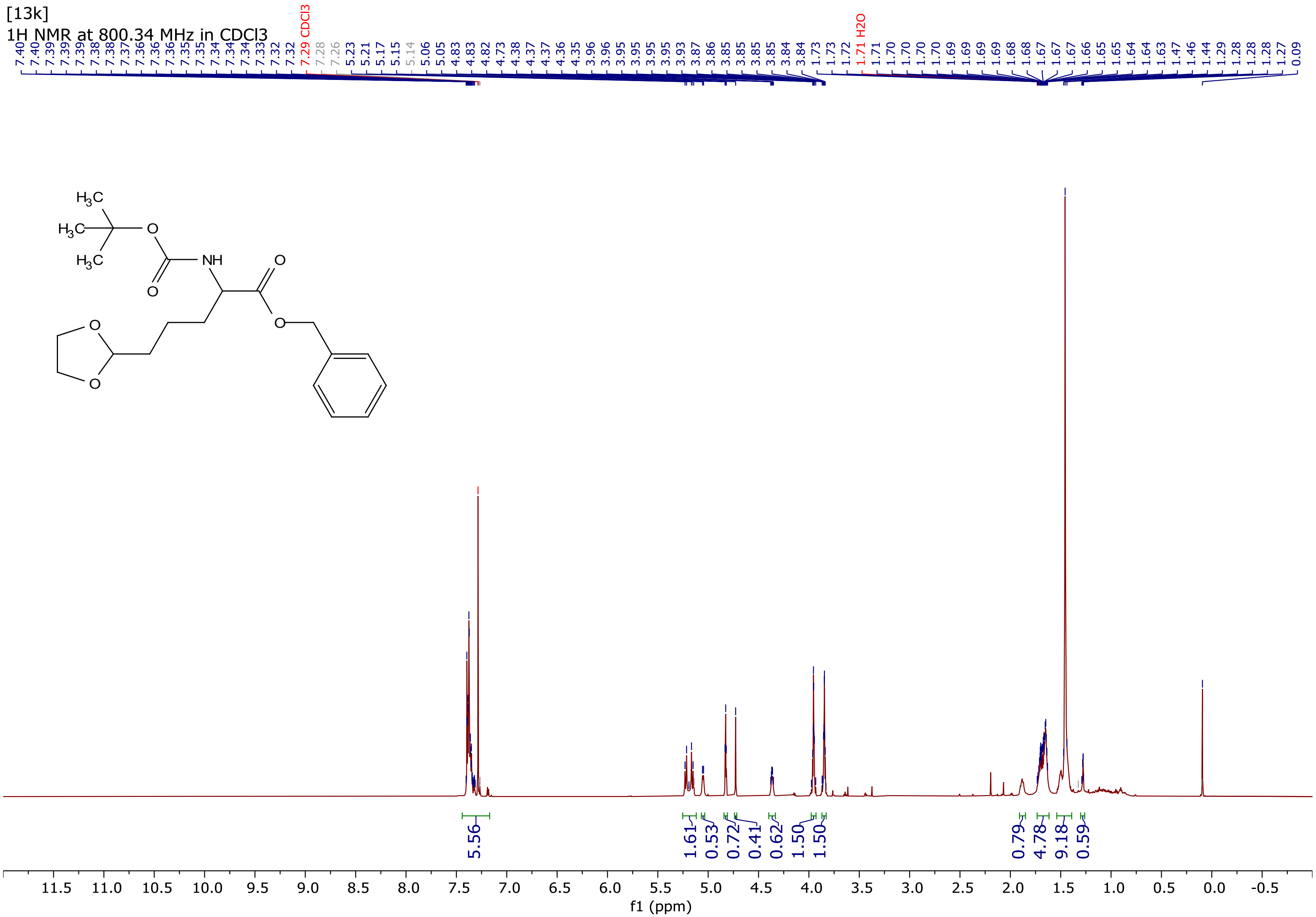

[13k]
13C NMR at 201.27 MHz in CDCl3

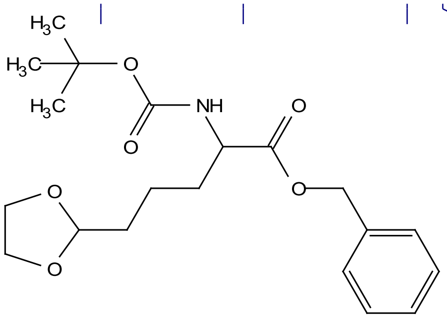

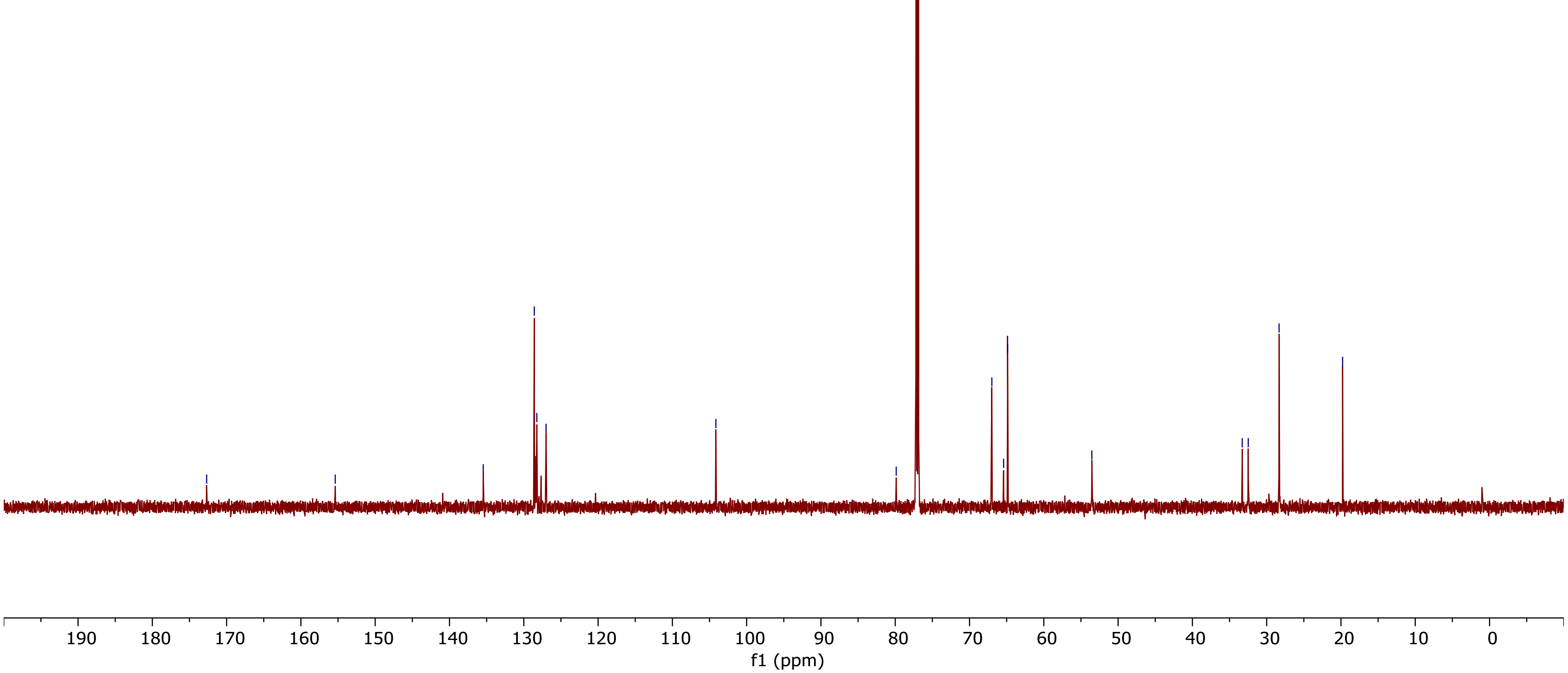

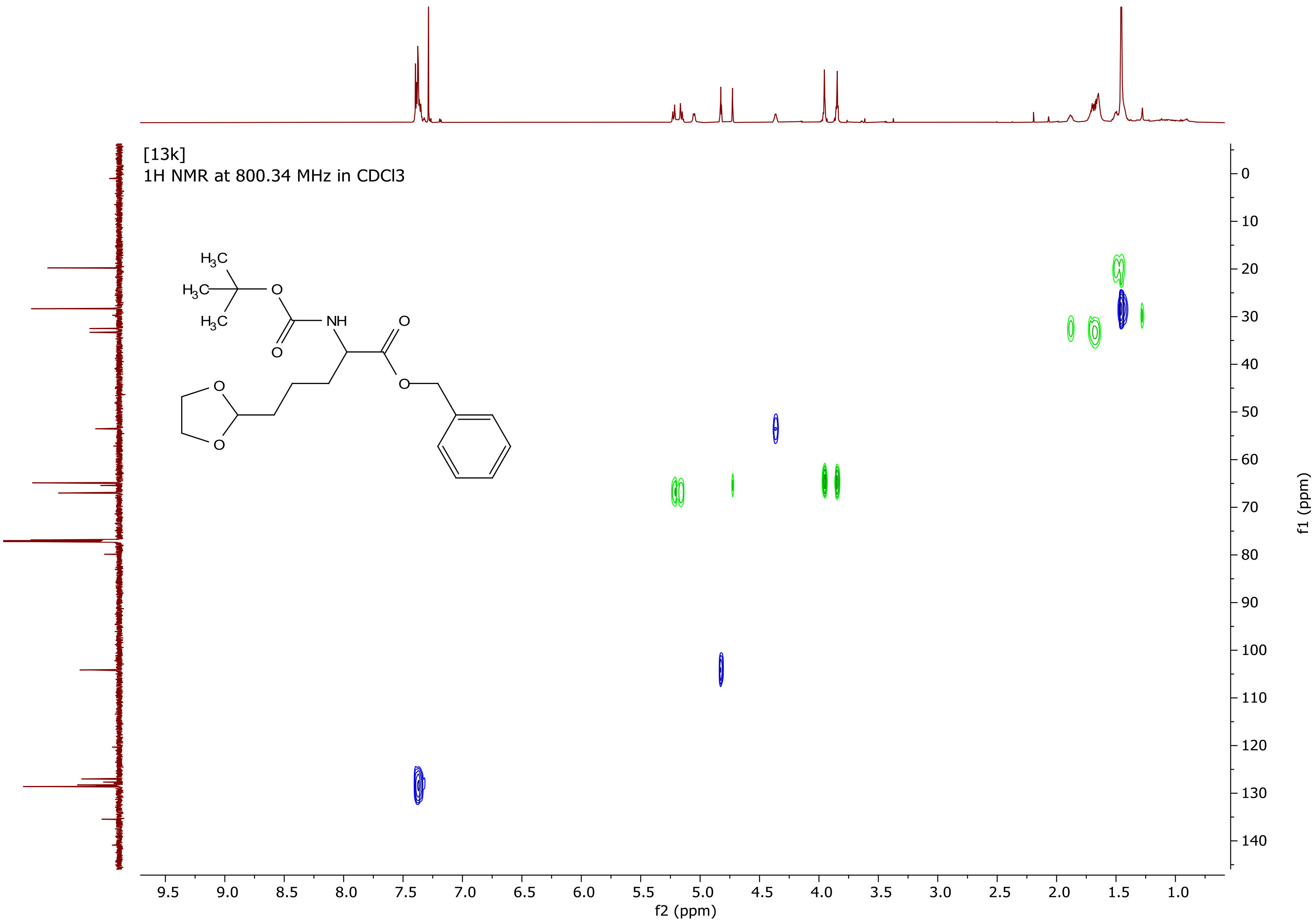
[13k]
1H NMR at 800.34 MHz in CDCl3
f2 (ppm)
f1 (ppm)

[37l]
1H NMR at 400.15 MHz in CDCl3

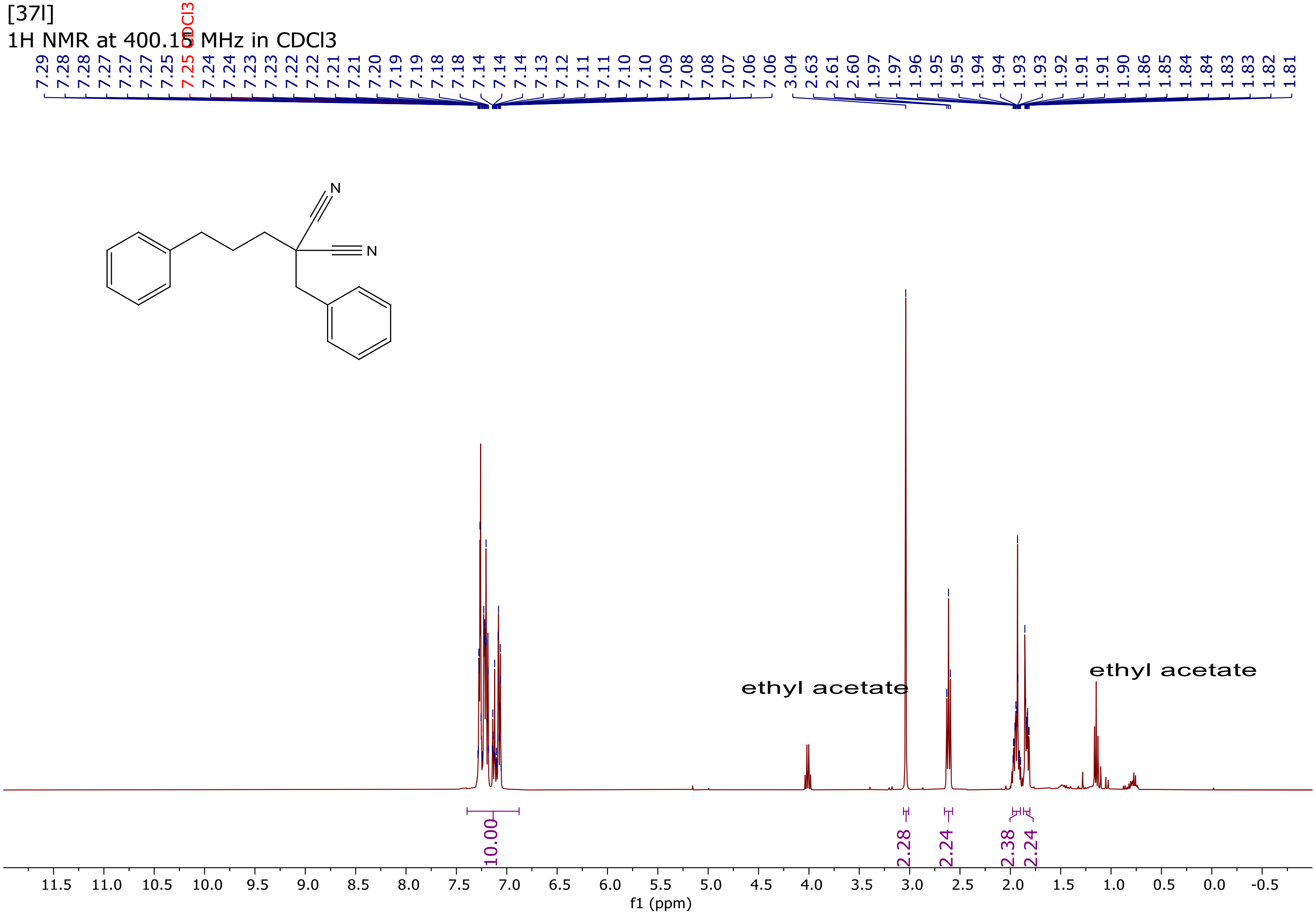

[37l]
13C NMR at 100.63 MHz in CDCl3

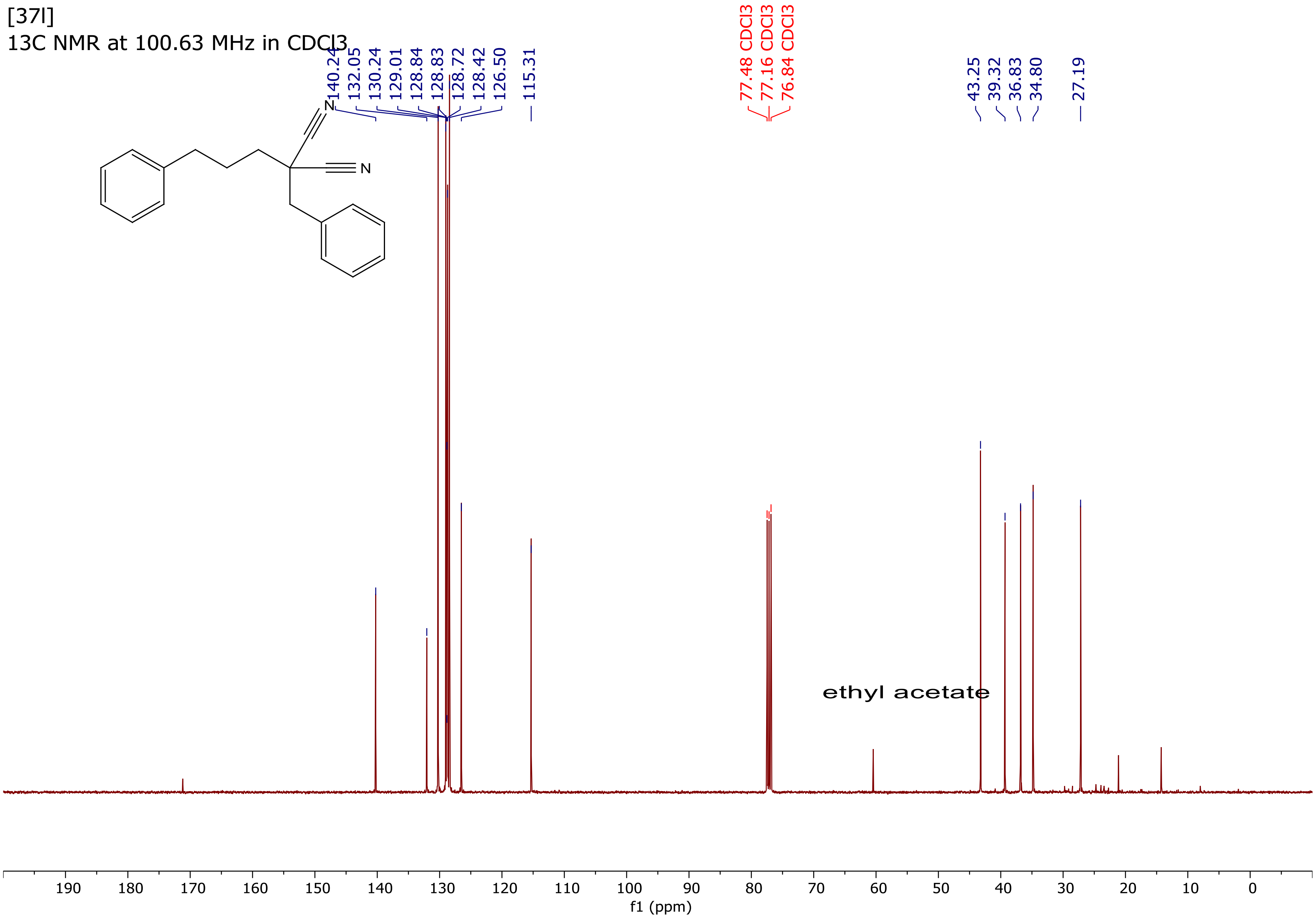

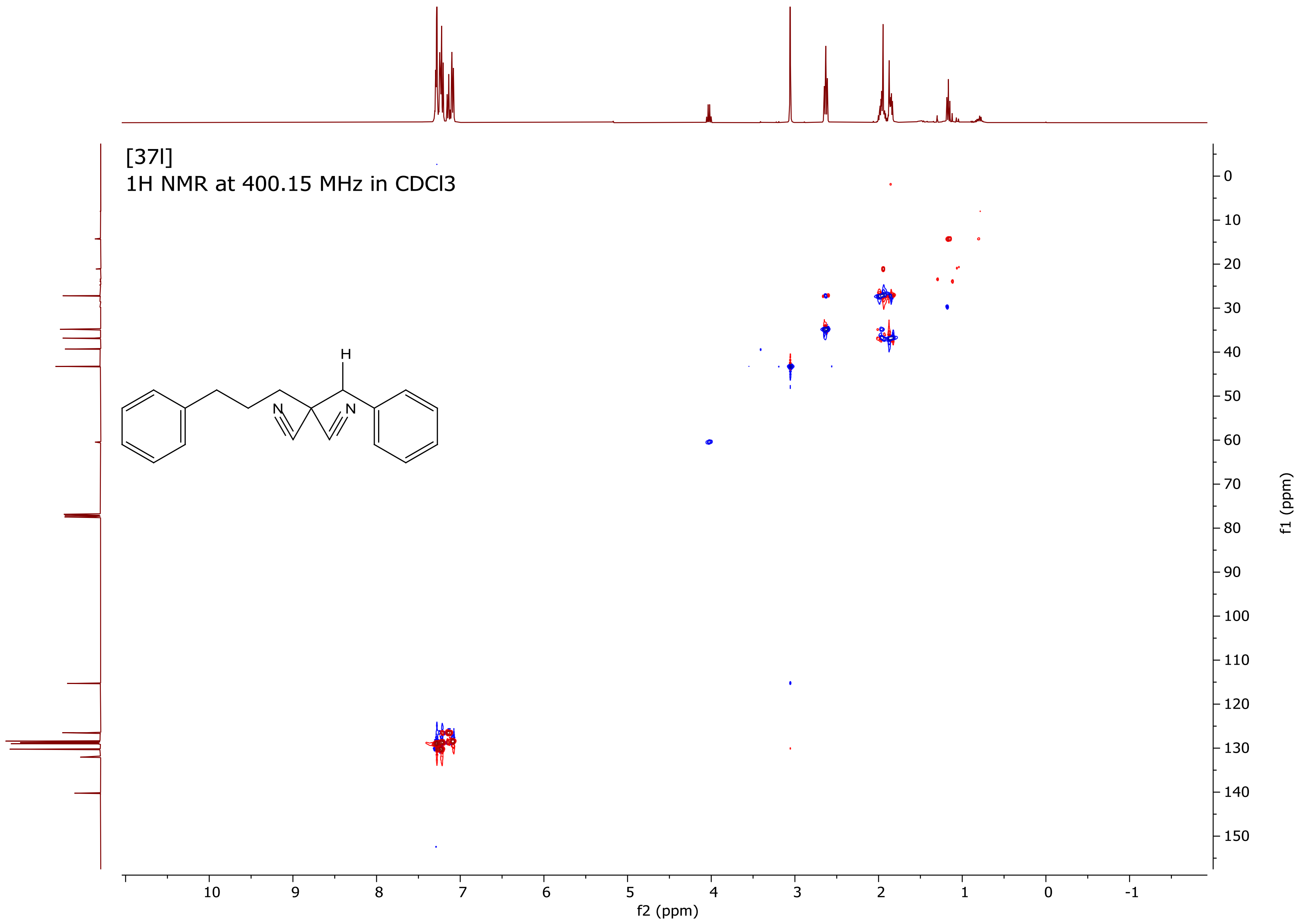
[37l]
1H NMR at 400.15 MHz in CDCl3
H
N
N
f2 (ppm)
f1 (ppm)

[17m]

1H NMR at 800.34 MHz in CDCl3

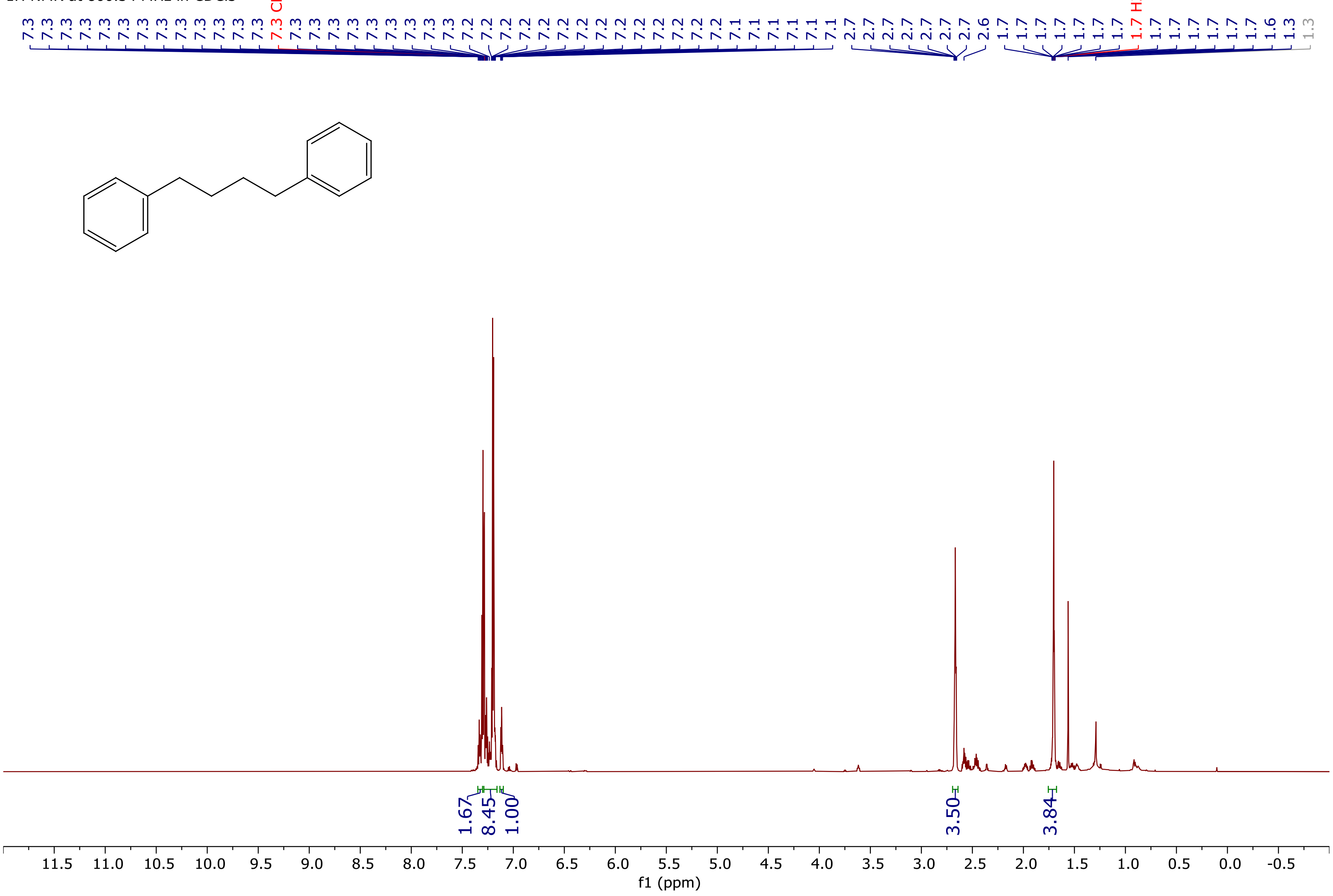

[17m]
13C NMR at 201.27 MHz in CDCl3

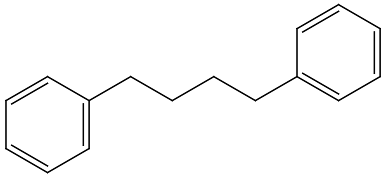

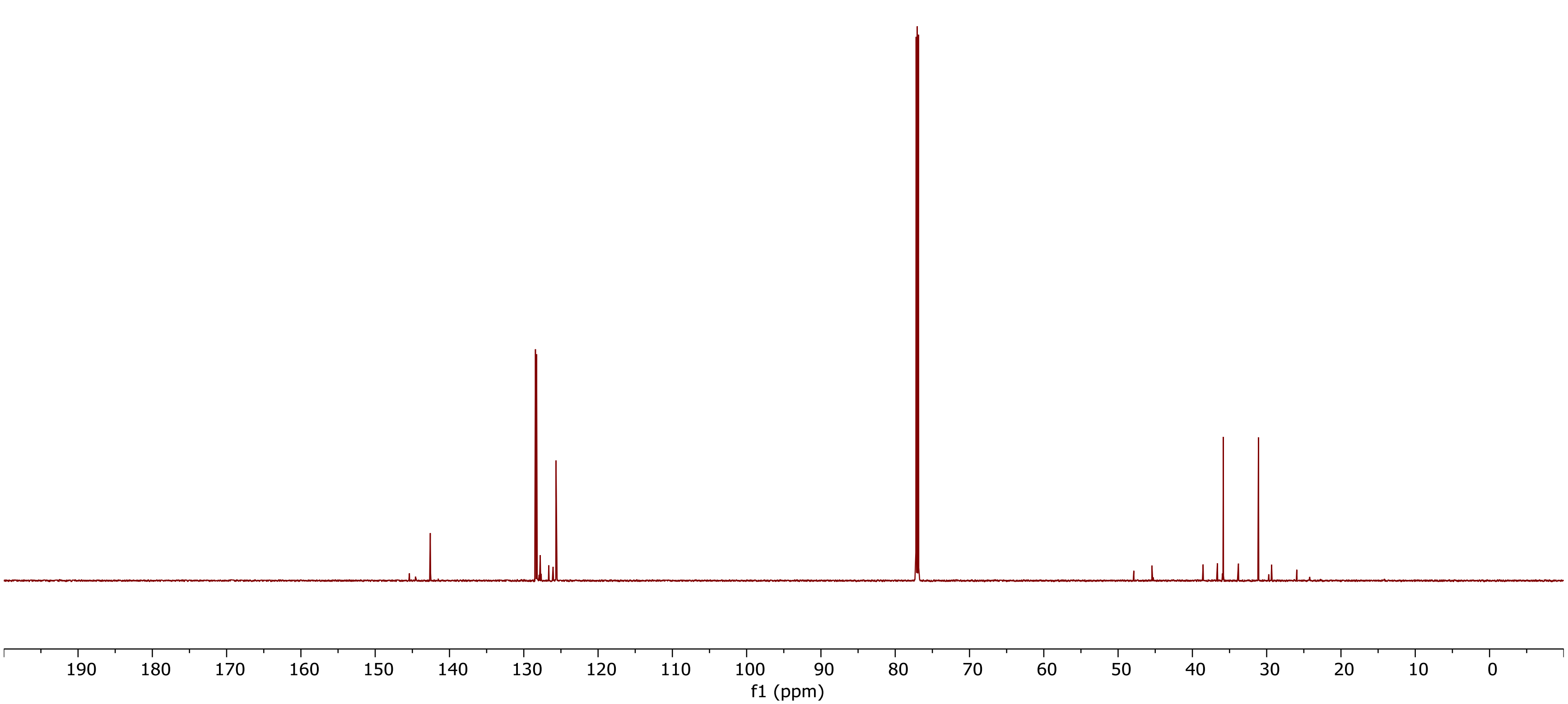

[13n]
1H NMR at 800.34 MHz in CDCl3

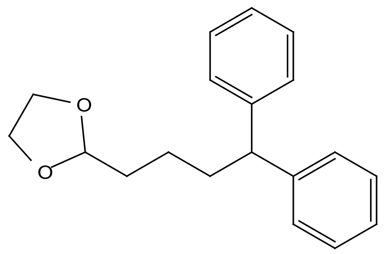

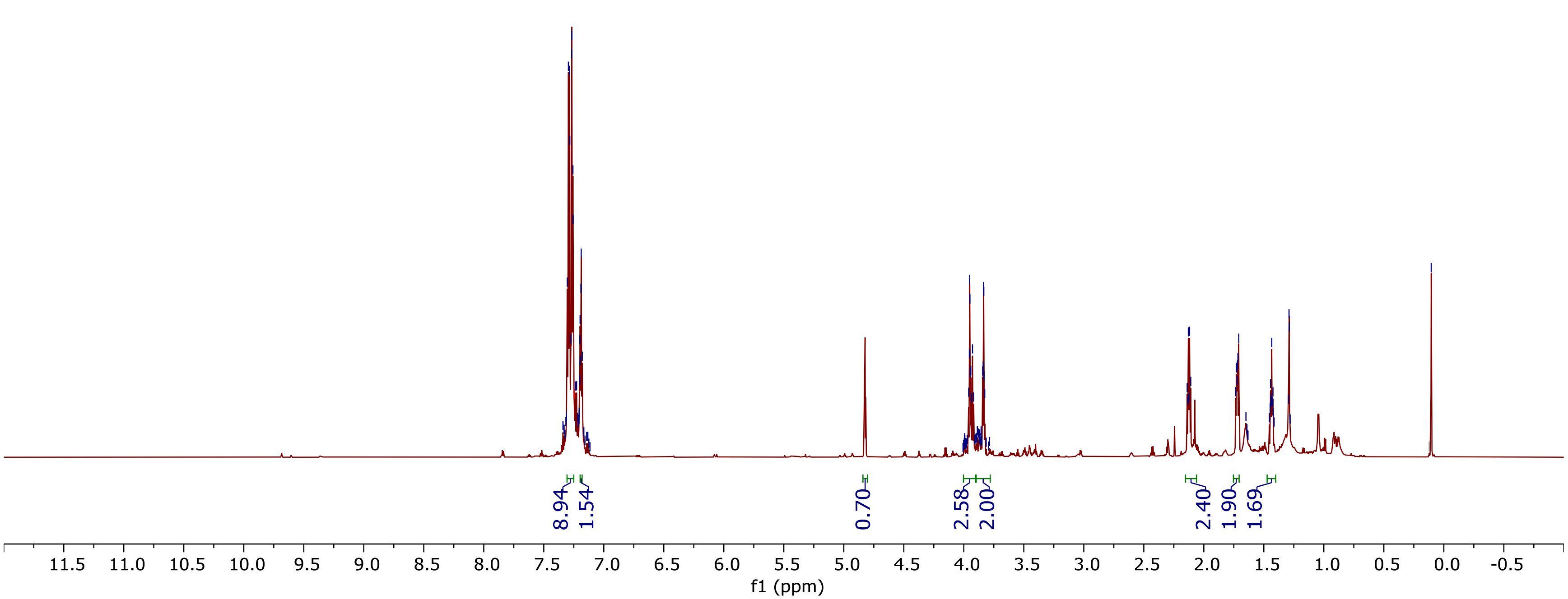

[13n]
13C NMR at 201.27 MHz in CDCl3

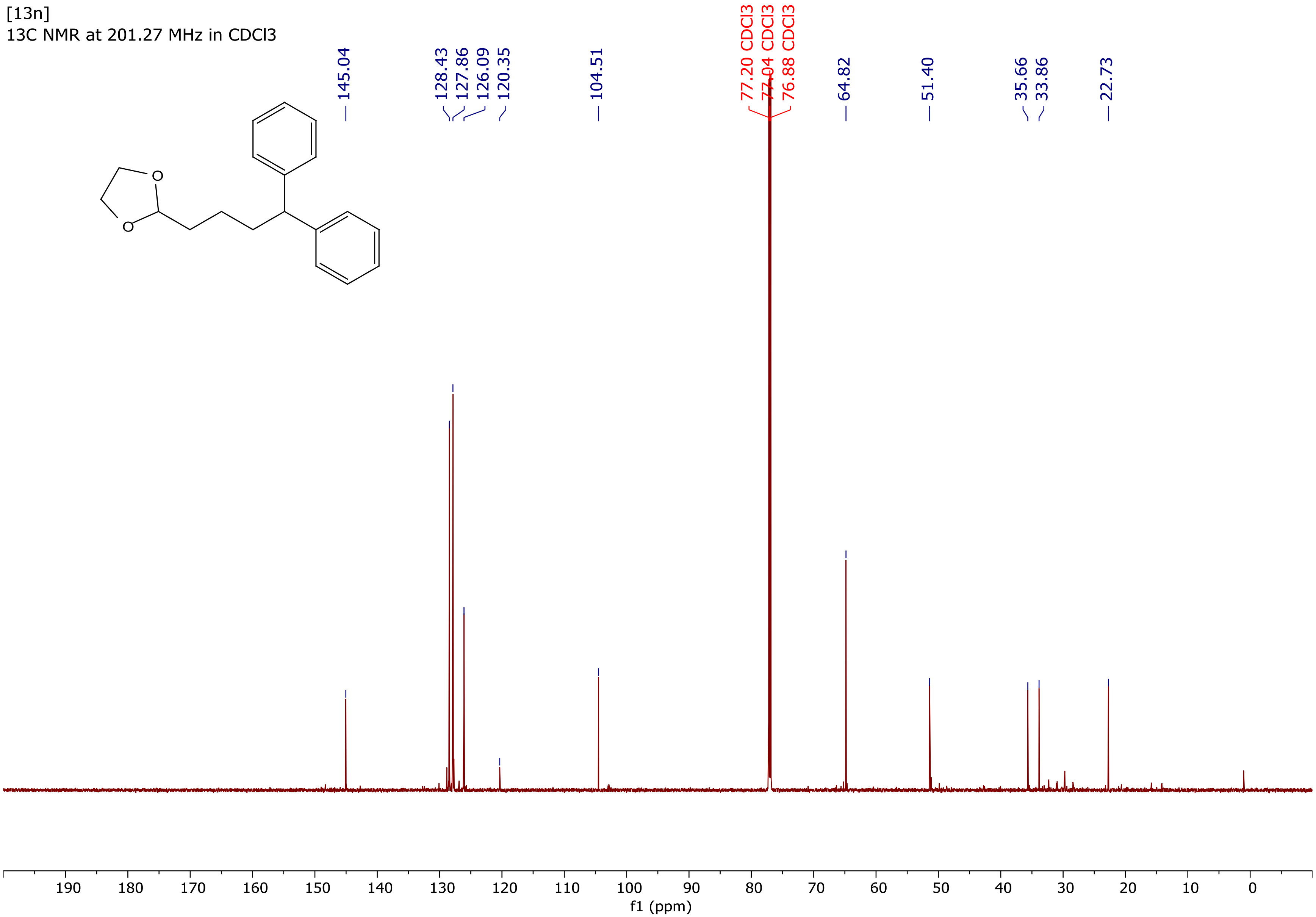

[13o]
1H NMR at 800.34 MHz in CDCl3

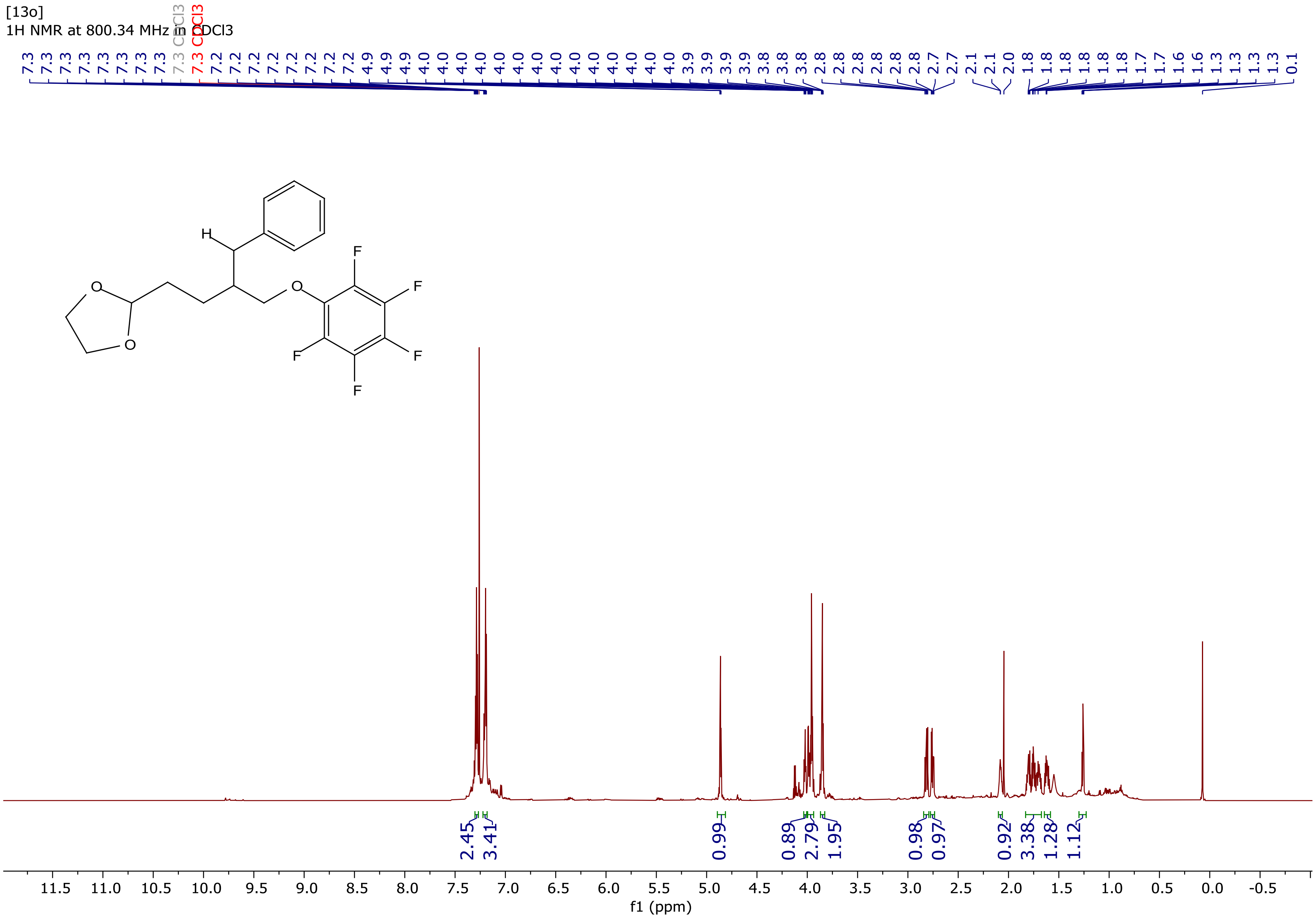

[13o]
13C NMR at 201.27 MHz in CDCl3

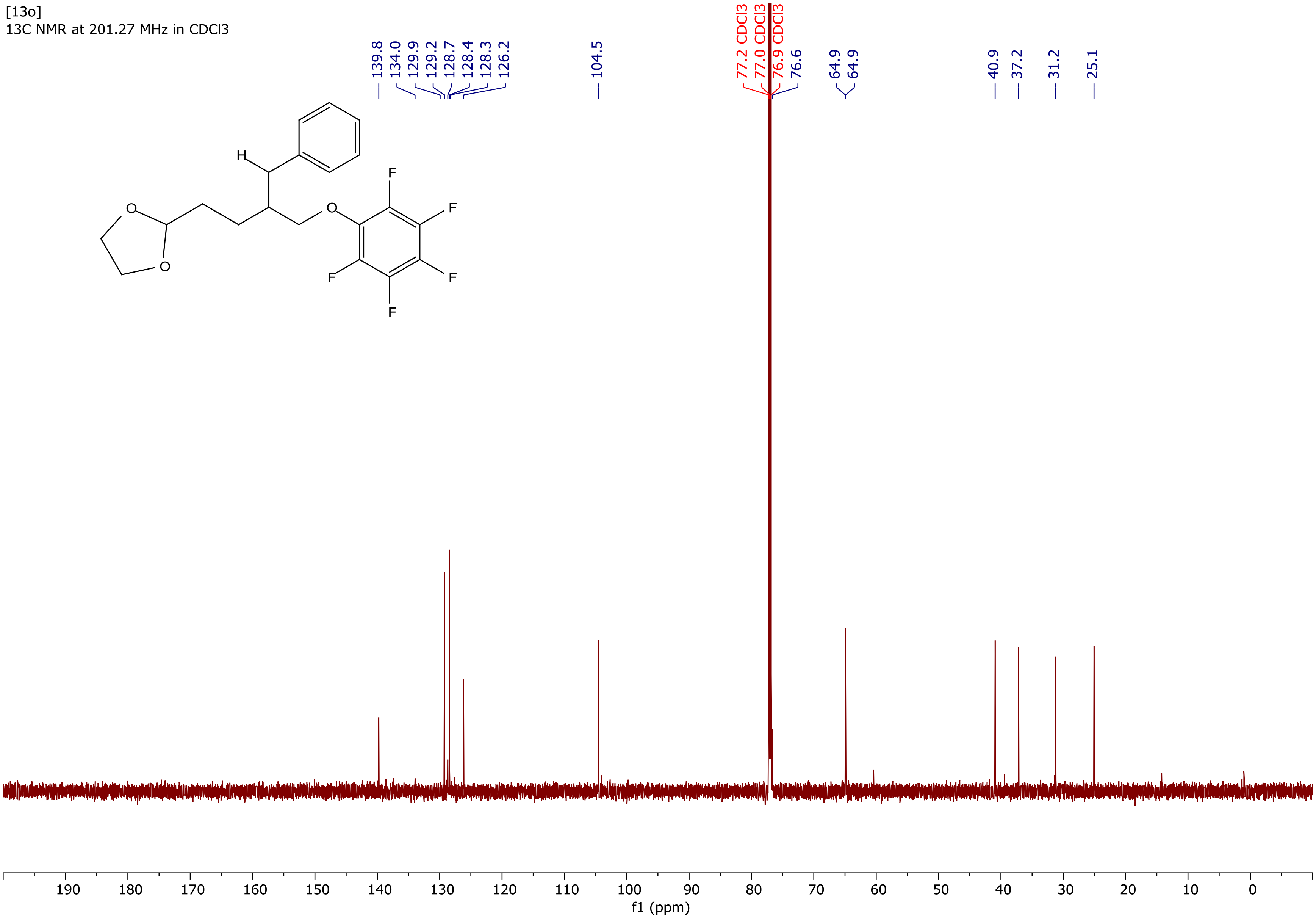

[13o]
19F NMR at 753.00 MHz in CDCl3

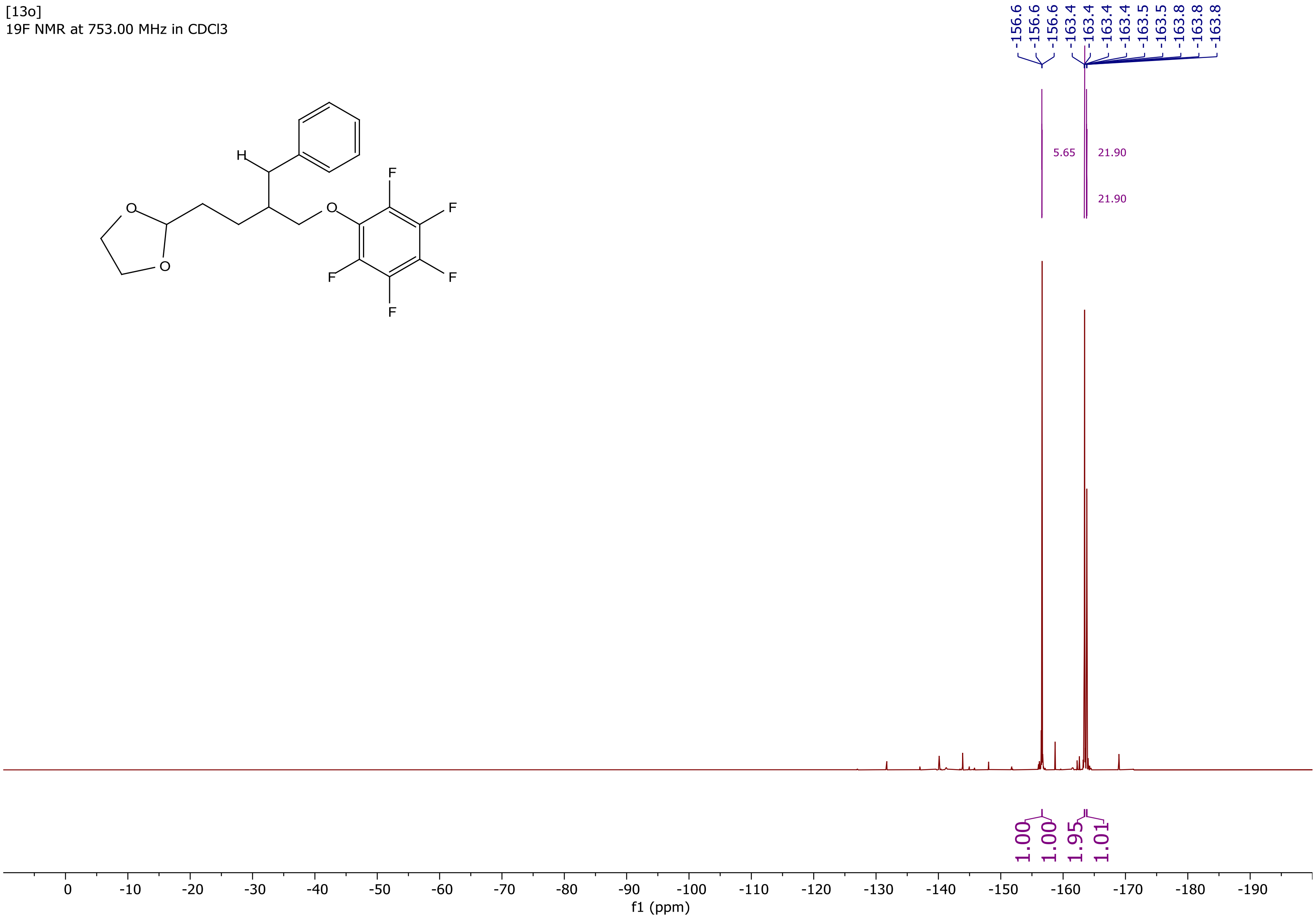

[18p]
1H NMR at 800.34 MHz in CDCl3

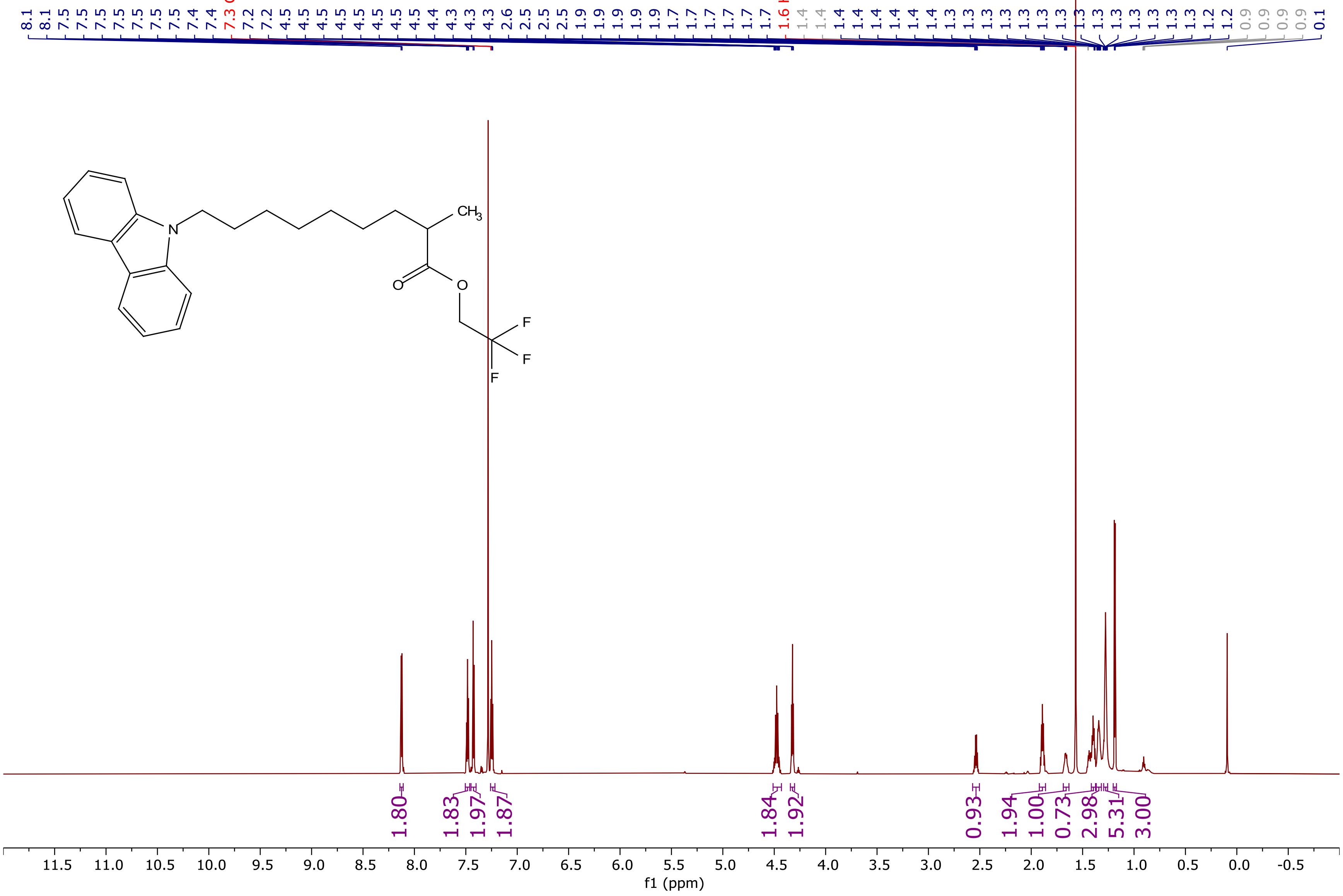

[18p]
13C NMR at 201.27 MHz in CDCl3

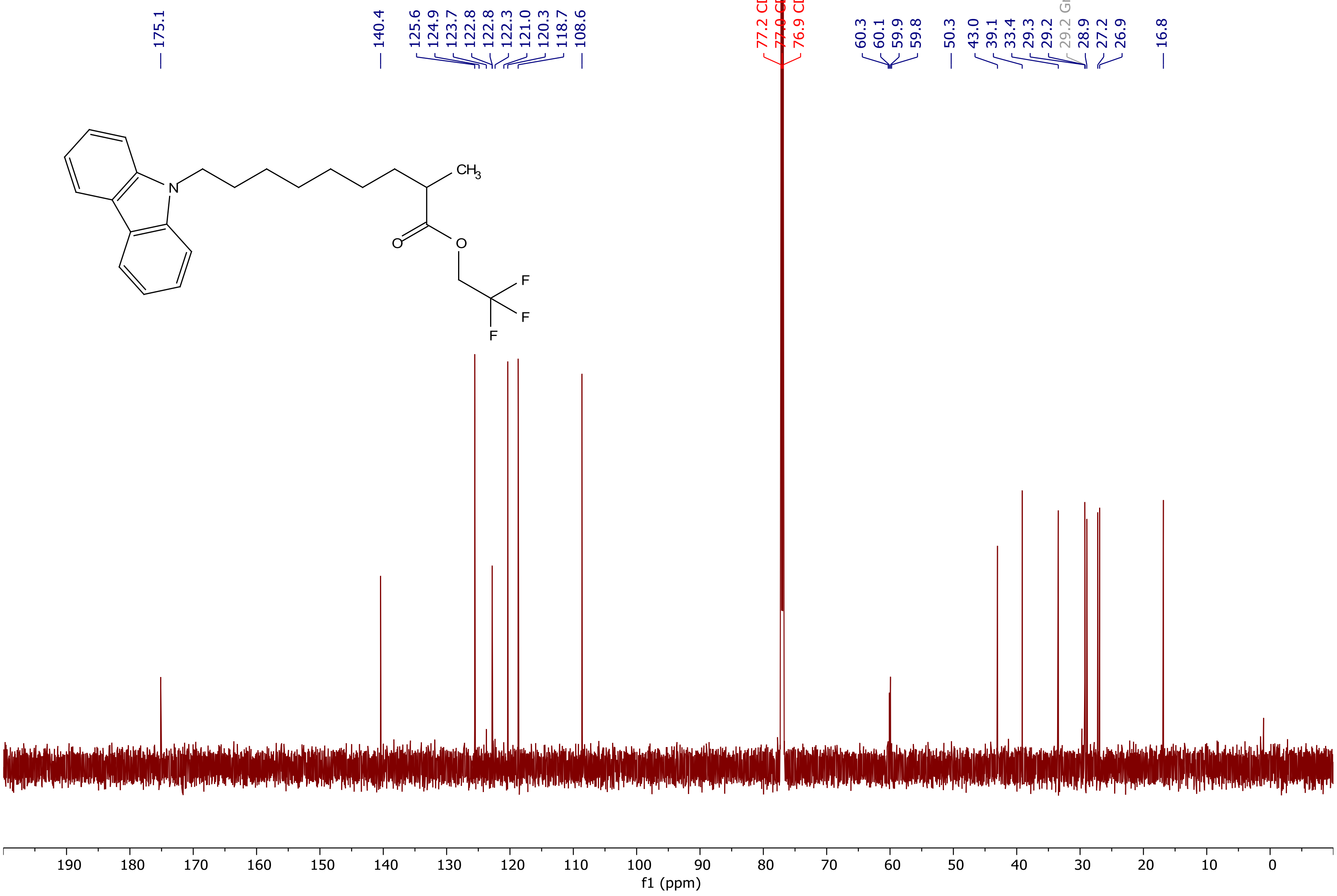

[18p]
19F NMR at 753.00 MHz in CDCl3

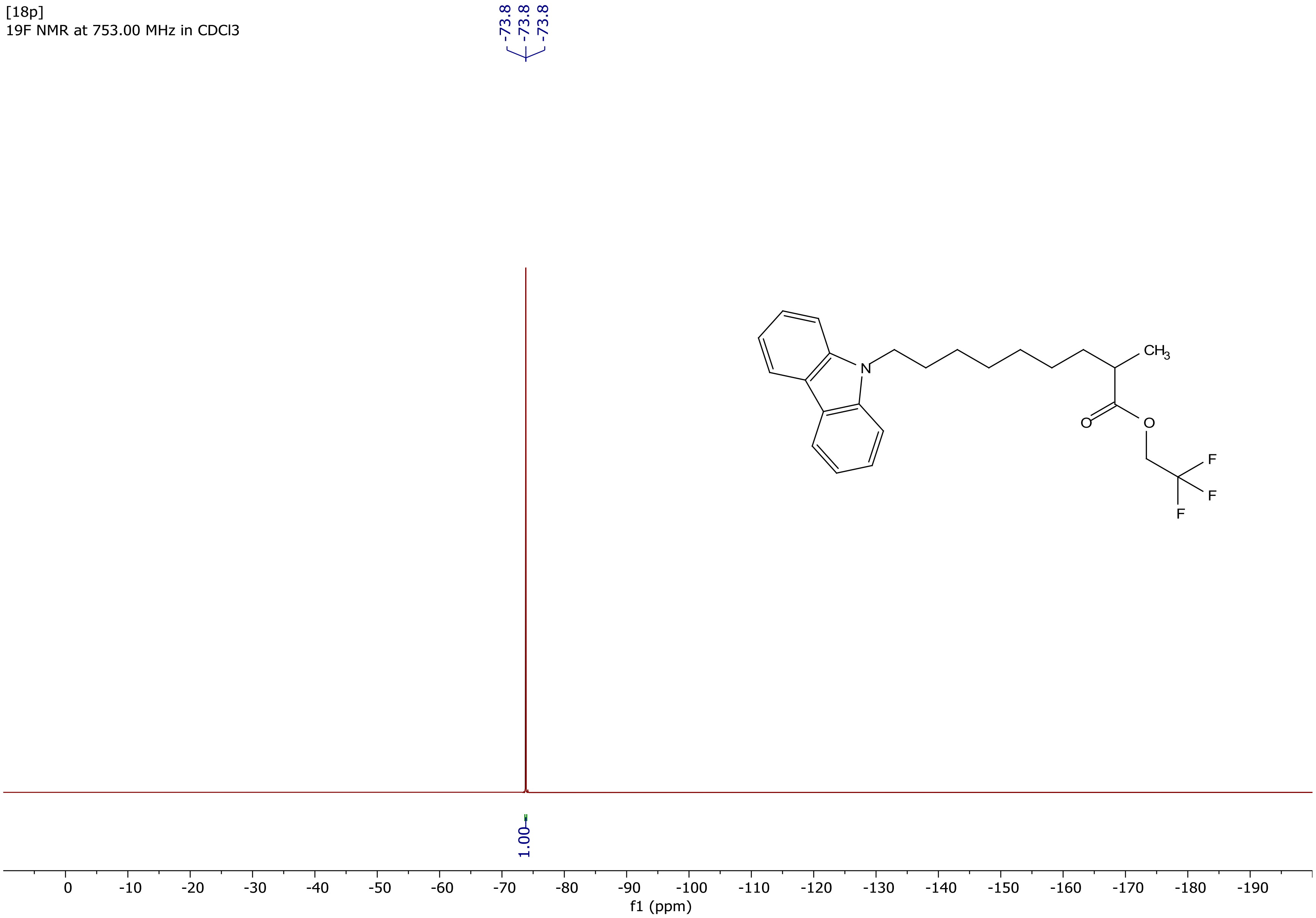

[17q]
1H NMR at 400.15 MHz in CDCl3

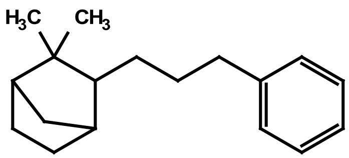

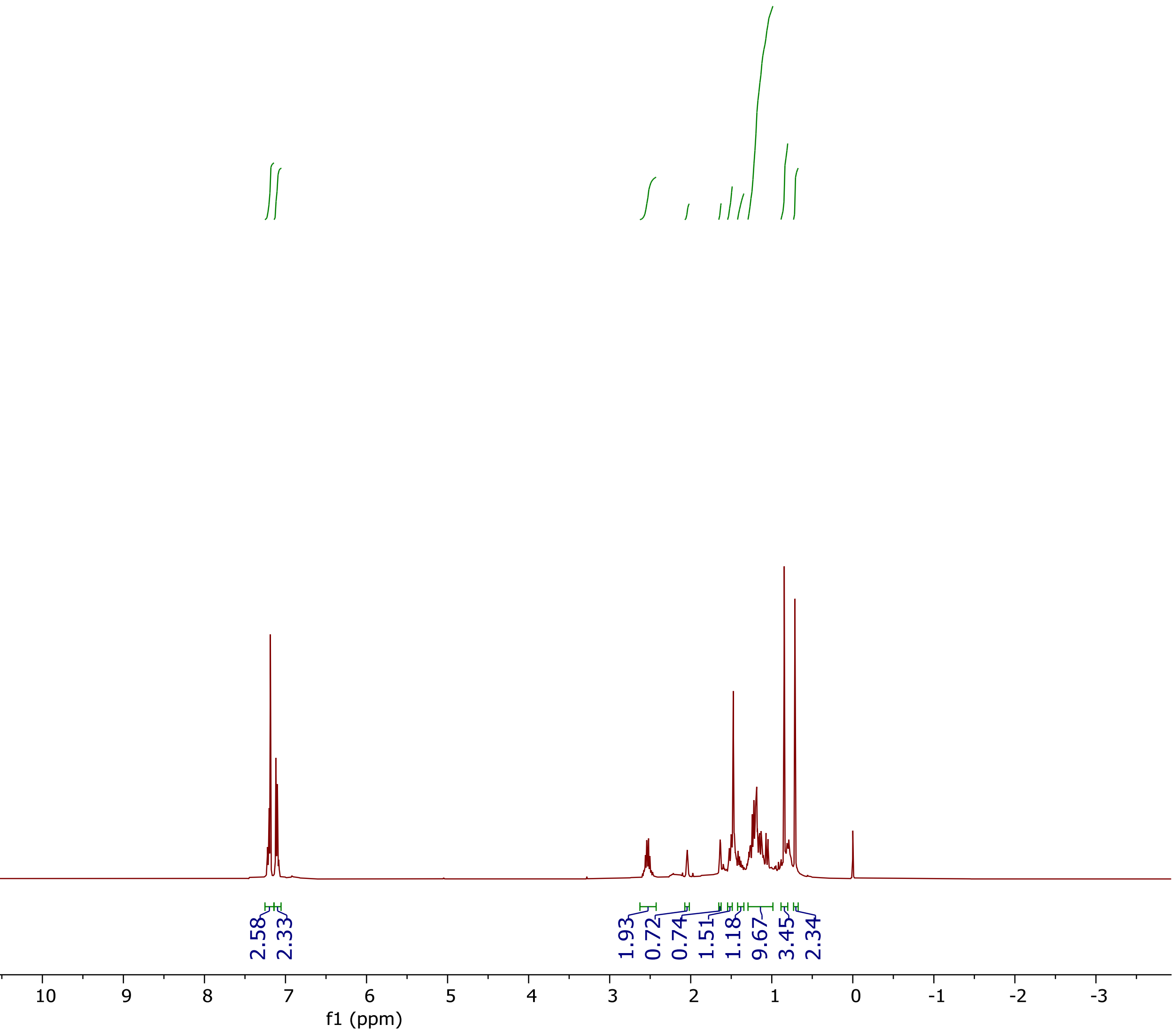

[17q]
13C NMR at 201.27 MHz in CDCl3

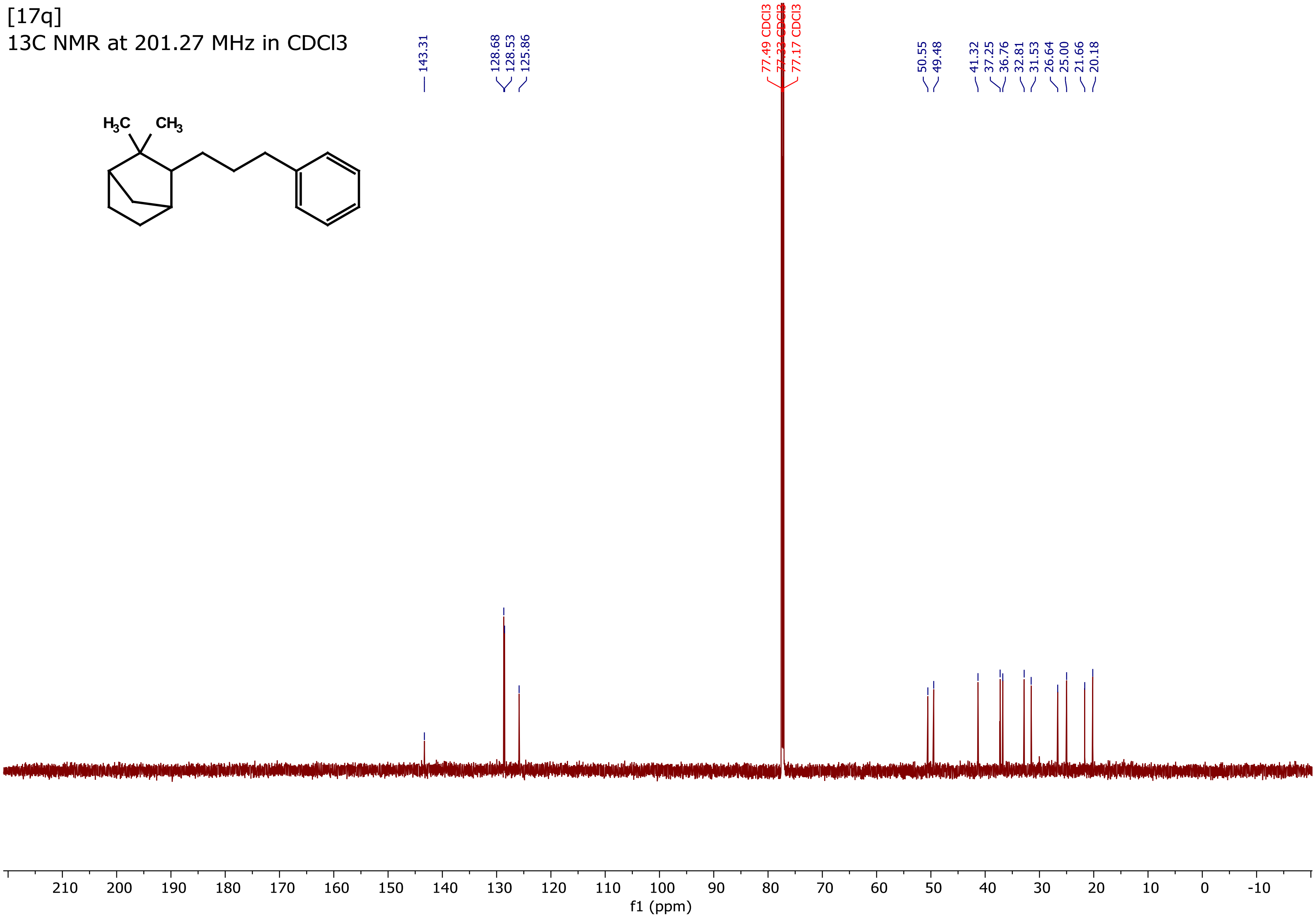

GCMS of 2,2-dimethyl-3-(3-phenylpropyl)bicyclo[2.2.1]heptane

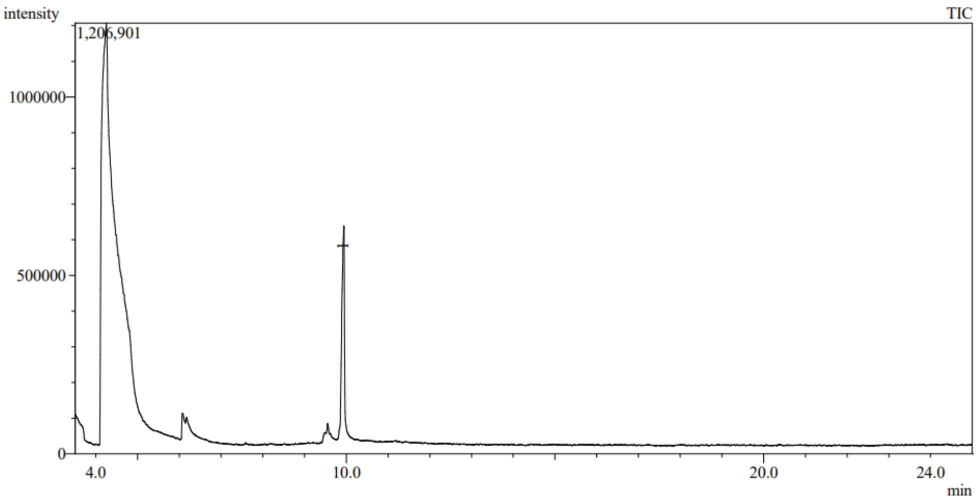

Library
<< Target >>
Line#:1 R.Time:9.920(Scan#:1285) MassPeaks:341
RawMode:Single 9.920(1285) BasePeak:109.15(63766)
BG Mode:None Group 1 - Event 1 Scan

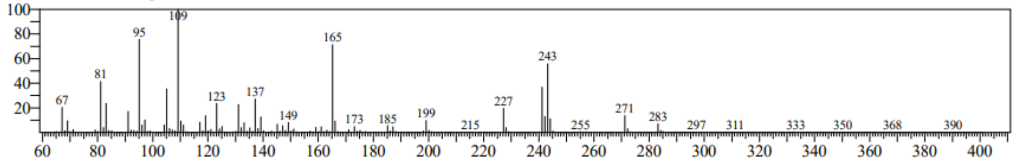

[17r]
1H NMR at 800.34 MHz in CDCl3

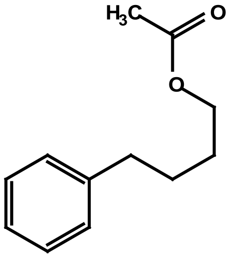

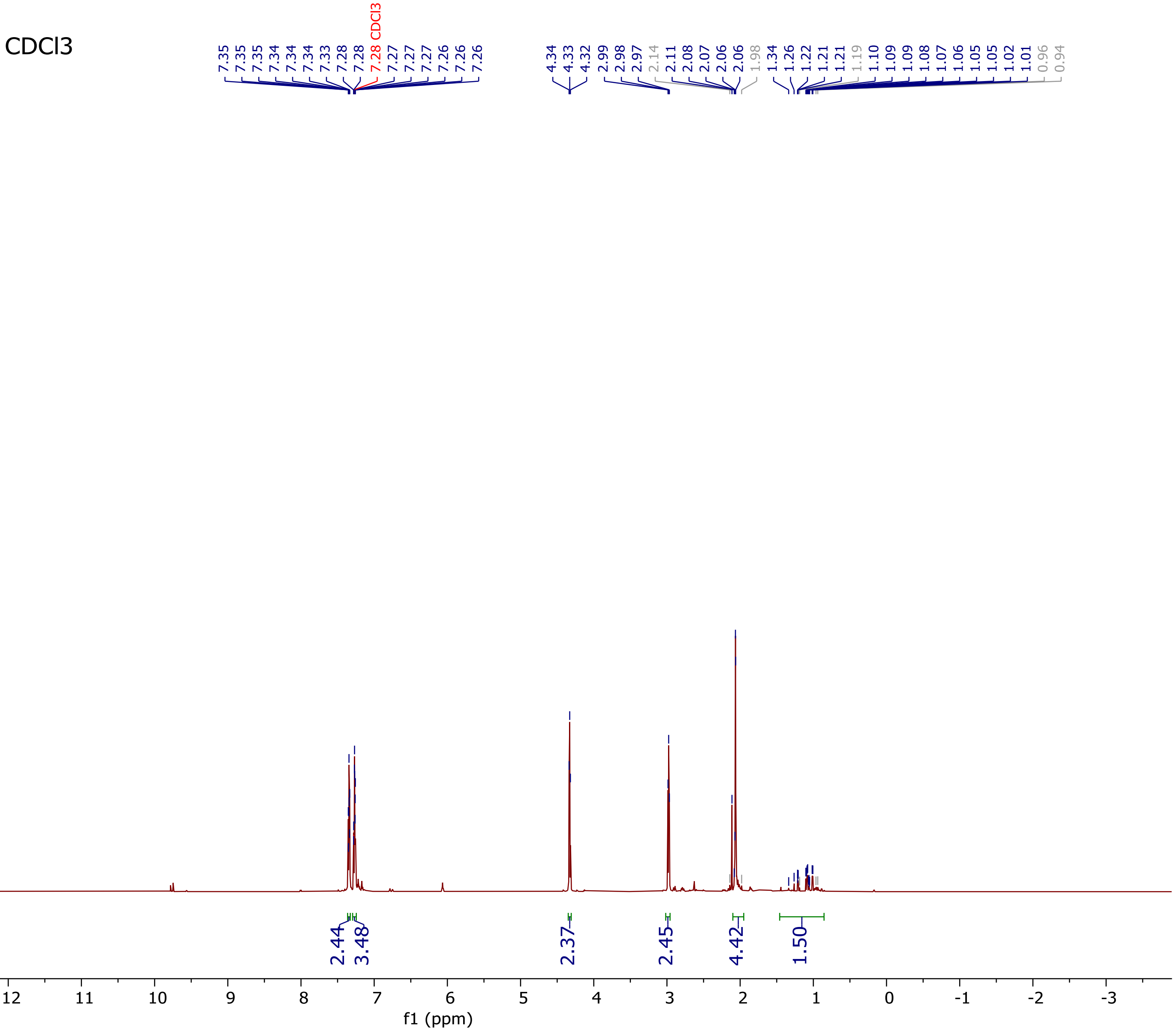

[17r]
13C NMR at 100.63 MHz in CDCl3

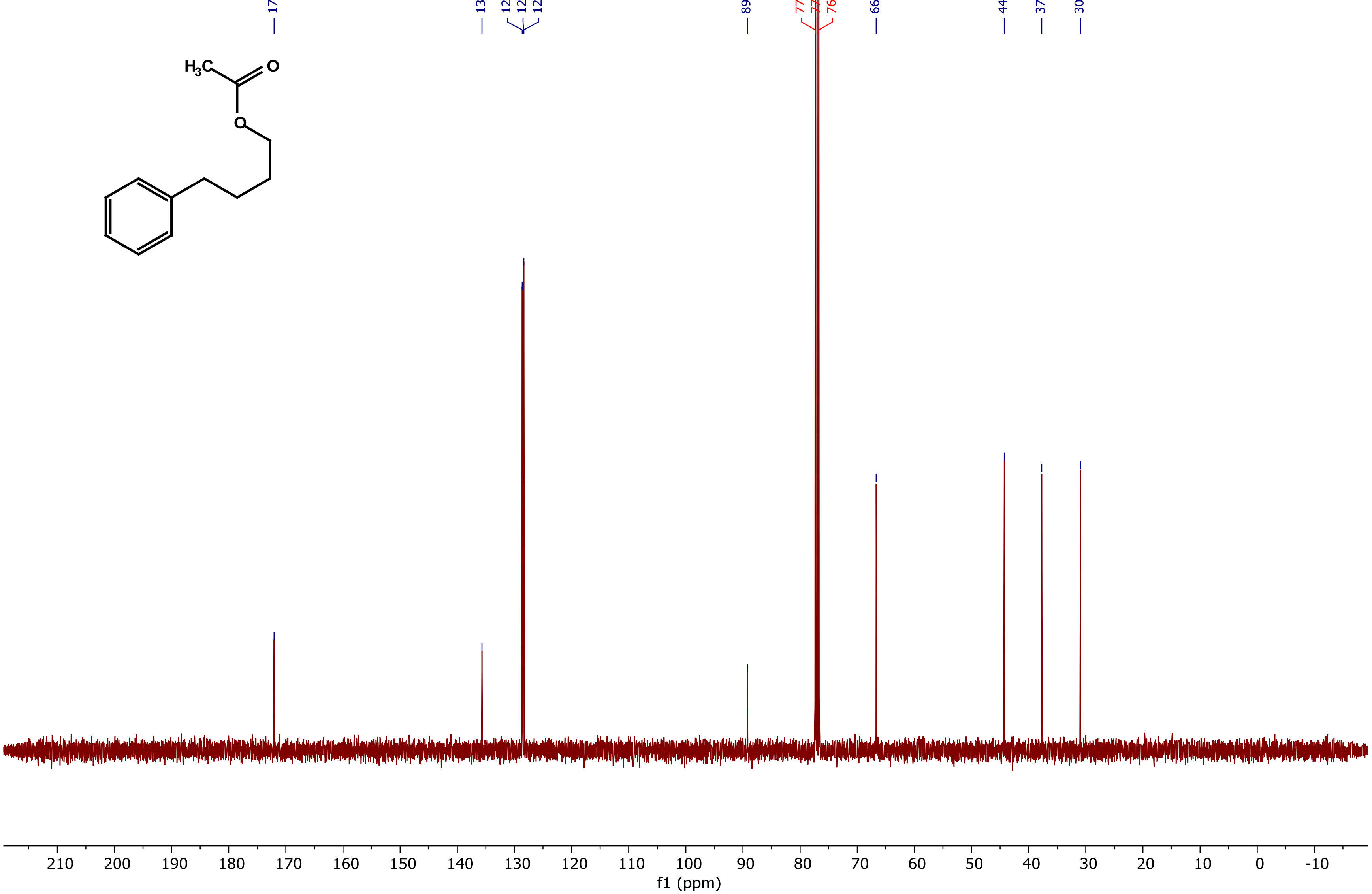

Supplement: Supplementary file 1 — ja4c15812_si_001.pdf [file ja4c15812_si_001.pdf]
