# Supplementary material for: Co-Catalytic Coupling of Alkyl Halides and Alkenes: the Curious Role of Lutidine
Source: J Am Chem Soc. 2025 Feb 3;147(6):5238–46. doi: 10.1021/jacs.4c15812 (PMC11827002; doi:10.1021/jacs.4c15812)
Supplement: Supplementary file 2 — ja4c15812_si_002.zip [file ja4c15812_si_002.zip › 21a/Primary_NMR_Data_files/21a-F19.zip!/PS-2171-C19-F/10/pdata/1/email_PS-2171-C19-F_10_1.pdf]

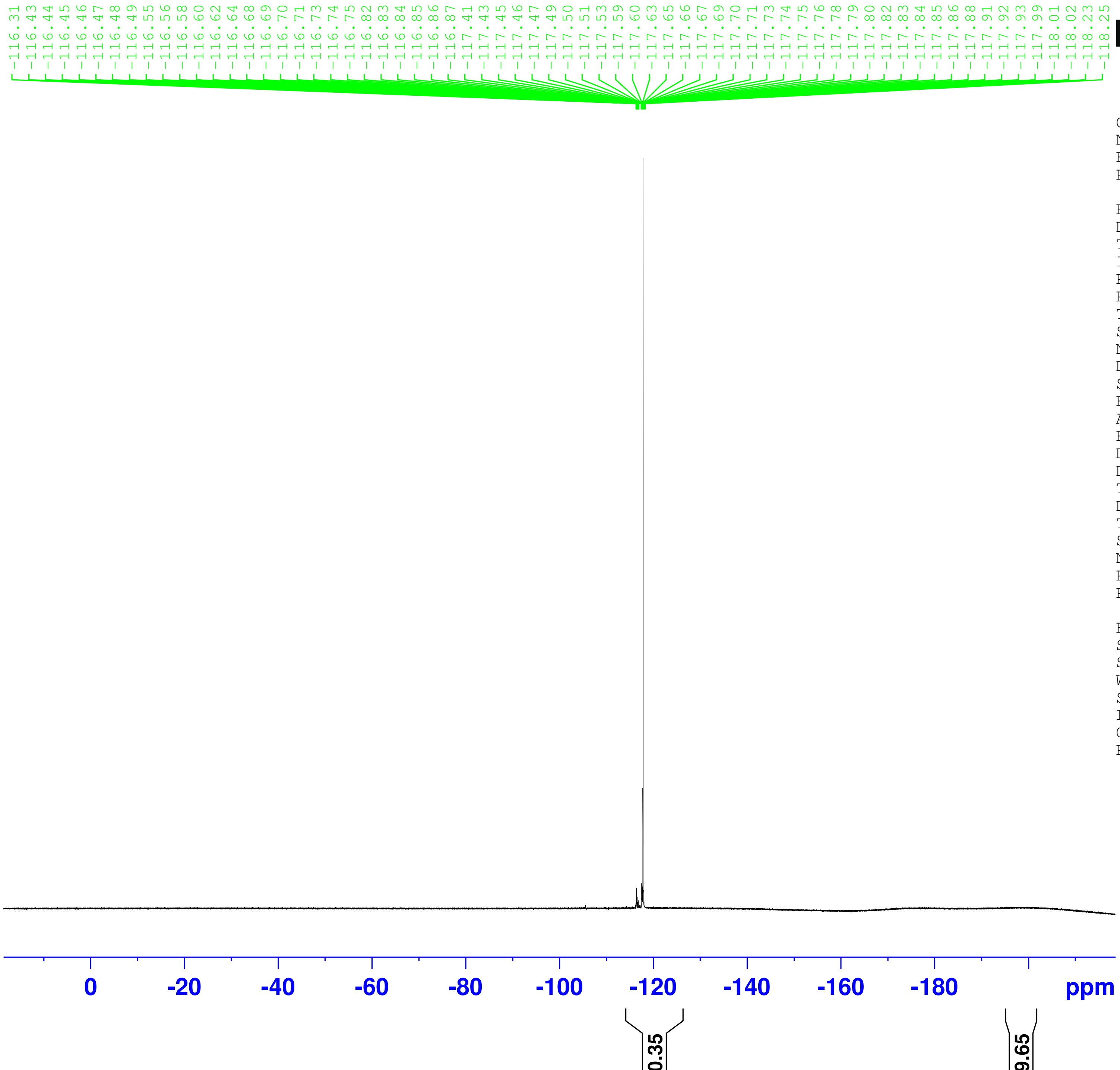

Current Data Parameters
NAME PS-2171-C19-F
EXPNO 10
PROCNO 1

F2 - Acquisition Parameters
Date_ 20241205
Time 15.47 h
INSTRUM spect
PROBHD Z116098_0222 (
PULPROG zgflqn
TD 131072
SOLVENT CDCl3
NS 16
DS 4
SWH 89285.711 Hz
FIDRES 1.362392 Hz
AQ 0.7340032 sec
RG 206.31
DW 5.600 usec
DE 6.50 usec
TE 298.0 K
D1 1.00000000 sec
TD0 1
SFO1 376.4795333 MHz
NUC1 19F
P1 14.00 usec
PLW1 22.00000000 W

F2 - Processing parameters
SI 65536
SF 376.5171850 MHz
WDW EM
SSB 0
LB 0.30 Hz
GB 0
PC 1.00
