# Supplementary material for: Co-Catalytic Coupling of Alkyl Halides and Alkenes: the Curious Role of Lutidine
Source: J Am Chem Soc. 2025 Feb 3;147(6):5238–46. doi: 10.1021/jacs.4c15812 (PMC11827002; doi:10.1021/jacs.4c15812)
Supplement: Supplementary file 2 — ja4c15812_si_002.zip [file ja4c15812_si_002.zip › 24a/Primary_NMR_Data_files/24a-Br-HSQC.zip!/RH-2251-col/21/pdata/1/email_RH-2251-col_21_1.pdf]

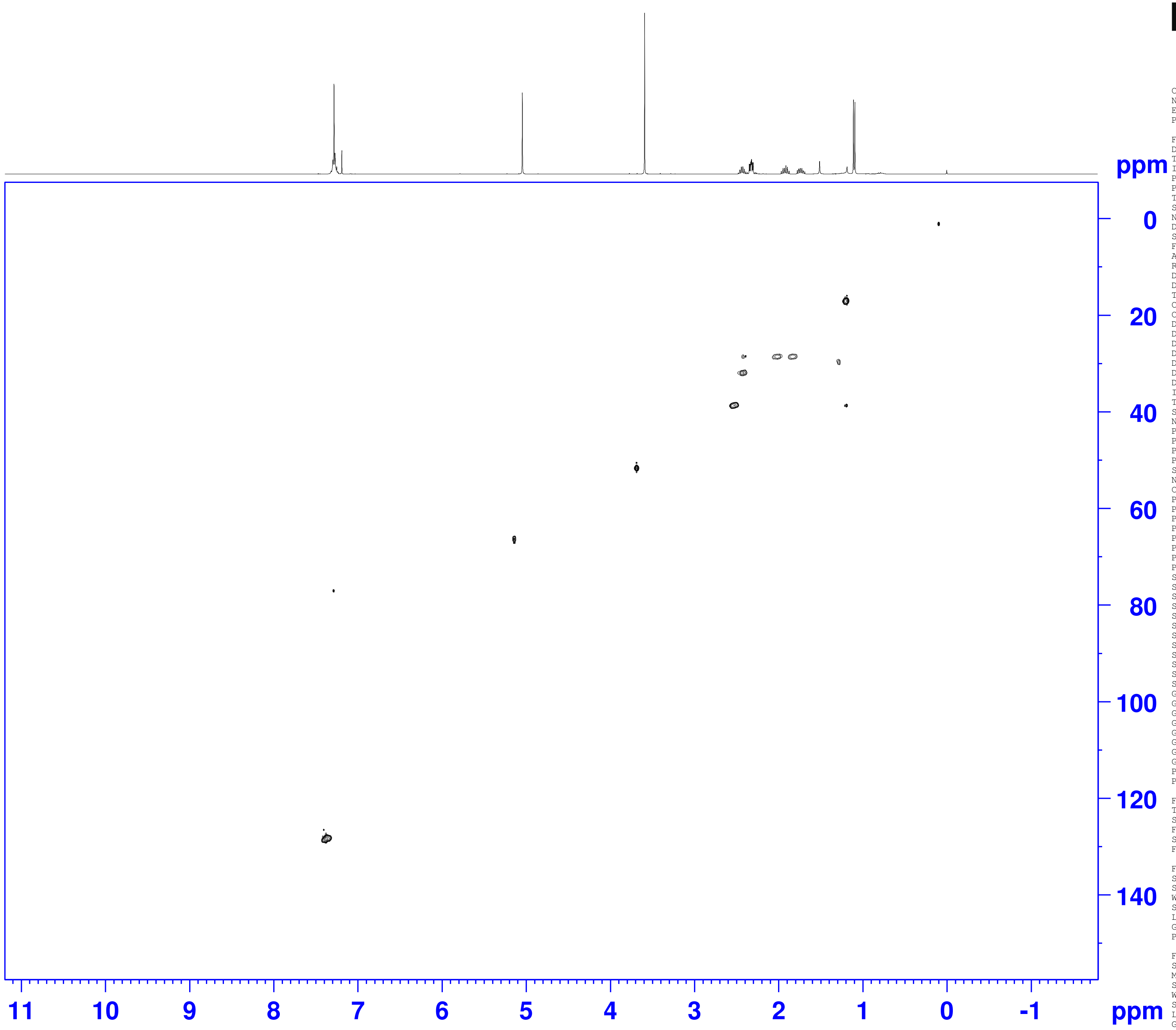


```
Current Data Parameters
NAME          RH-2251-col
EXPNO                  21
PROCNO                  1

F2 - Acquisition Parameters
Date_            20230907
Time                19.43 h
INSTRUM             spect
PROBHD   Z116098_0222 (
PULPROG  hsqcedetgpsisp2.3
TD                   2048
SOLVENT             CDCl3
NS                      4
DS                     32
SWH              5197.505 Hz
FIDRES           5.075689 Hz
AQ              0.1970176 sec
RG                 206.31
DW                 96.200 usec
DE                   6.50 usec
TE                  298.0 K
CNST2         145.0000000
CNST17         -0.5000000
D0             0.00000300 sec
D1             1.50000000 sec
D4             0.00172414 sec
D11            0.03000000 sec
D16            0.00020000 sec
D21            0.00344800 sec
D24            0.00086200 sec
IN0            0.00003010 sec
TDav                    1
SFO1          400.1518807 MHz
NUC1                   1H
P1                  10.00 usec
P2                  20.00 usec
P28               1000.00 usec
PLW1          13.89000034 W
SFO2          100.6253439 MHz
NUC2                  13C
CPDPRG[2            garp4
P3                  10.00 usec
P14                500.00 usec
P24               2000.00 usec
P31               2119.00 usec
PCPD2               80.00 usec
PLW0      0 W
PLW2          70.00000000 W
PLW12          1.09379995 W
SPNAM[3] Crp60,0.5,20.1
SPOAL3              0.500
SPOFFS3   0 Hz
SPW3          10.69499969 W
SPNAM[7]     Crp60comp.4
SPOAL7              0.500
SPOFFS7   0 Hz
SPW7          10.69499969 W
SPNAM[18   Crp60_xfilt.2
SPOAL18             0.500
SPOFFS18  0 Hz
SPW18          2.06040001 W
GPNAM[1]       SMSQ10.100
GPZ1                80.00 %
GPNAM[2]       SMSQ10.100
GPZ2                20.10 %
GPNAM[3]       SMSQ10.100
GPZ3                11.00 %
GPNAM[4]       SMSQ10.100
GPZ4                -5.00 %
P16               1000.00 usec
P19                600.00 usec

F1 - Acquisition parameters
TD                    256
SFO1             100.6253 MHz
FIDRES         129.775742 Hz
SW                165.081 ppm
FnMODE       Echo-Antiecho

F2 - Processing parameters
SI                   1024
SF            400.1500000 MHz
WDW                 QSINE
SSB                     2
LB        0 Hz
GB        0
PC                   1.40

F1 - Processing parameters
SI                   1024
MC2          echo-antiecho
SF            100.6177975 MHz
WDW                 QSINE
SSB                     2
LB        0 Hz
GB        0
```
