# Supplementary material for: Co-Catalytic Coupling of Alkyl Halides and Alkenes: the Curious Role of Lutidine
Source: J Am Chem Soc. 2025 Feb 3;147(6):5238–46. doi: 10.1021/jacs.4c15812 (PMC11827002; doi:10.1021/jacs.4c15812)
Supplement: Supplementary file 2 — ja4c15812_si_002.zip [file ja4c15812_si_002.zip › 25a/Primary_NMR_Data_files/25a-H1.zip!/RH-2213-col/10/pdata/1/email_RH-2213-col_10_1.pdf]

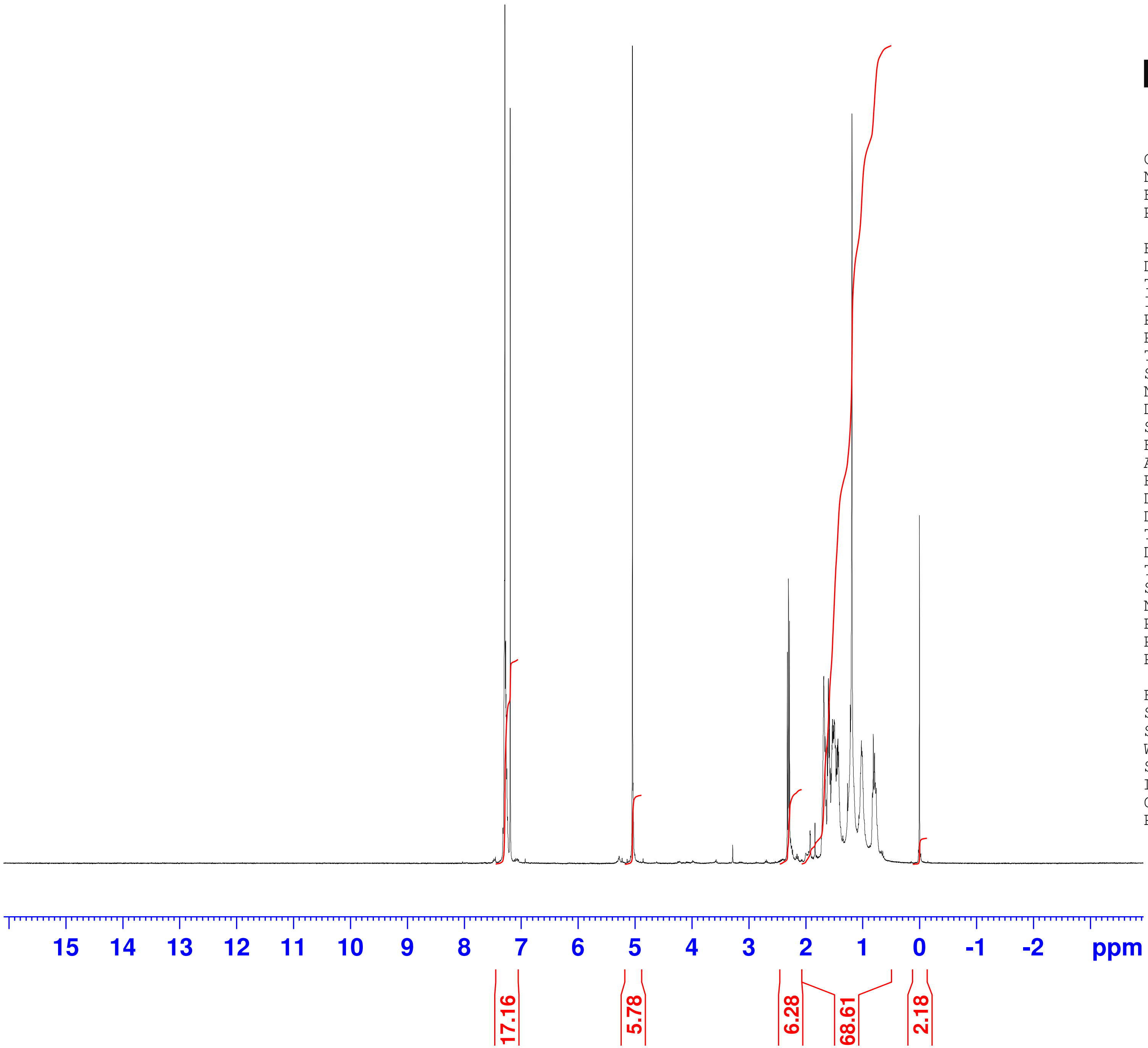


Current Data Parameters

| | |
|---|---|
| NAME | RH-2213-col |
| EXPNO | 10 |
| PROCNO | 1 |

F2 - Acquisition Parameters

| | |
|---|---|
| Date_ | 20230818 |
| Time | 21.02 h |
| INSTRUM | spect |
| PROBHD | Z116098_0222 ( |
| PULPROG | zg30 |
| TD | 65536 |
| SOLVENT | CDCl3 |
| NS | 16 |
| DS | 2 |
| SWH | 8012.820 Hz |
| FIDRES | 0.244532 Hz |
| AQ | 4.0894465 sec |
| RG | 61.86 |
| DW | 62.400 usec |
| DE | 6.50 usec |
| TE | 298.0 K |
| D1 | 1.00000000 sec |
| TD0 | 1 |
| SFO1 | 400.1524709 MHz |
| NUC1 | 1H |
| P0 | 3.33 usec |
| P1 | 10.00 usec |
| PLW1 | 13.89000034 W |

F2 - Processing parameters

| | | |
|---|---|---|
| SI | | 65536 |
| SF | | 400.1500378 MHz |
| WDW | | EM |
| SSB | 0 | |
| LB | | 0.30 Hz |
| GB | 0 | |
| PC | | 1.00 |
