# Supplementary material for: Co-Catalytic Coupling of Alkyl Halides and Alkenes: the Curious Role of Lutidine
Source: J Am Chem Soc. 2025 Feb 3;147(6):5238–46. doi: 10.1021/jacs.4c15812 (PMC11827002; doi:10.1021/jacs.4c15812)
Supplement: Supplementary file 2 — ja4c15812_si_002.zip [file ja4c15812_si_002.zip › 27a/Primary_NMR_Data_files/27a-C13.zip!/RH-2234-col/11/pdata/1/email_RH-2234-col_11_1.pdf]

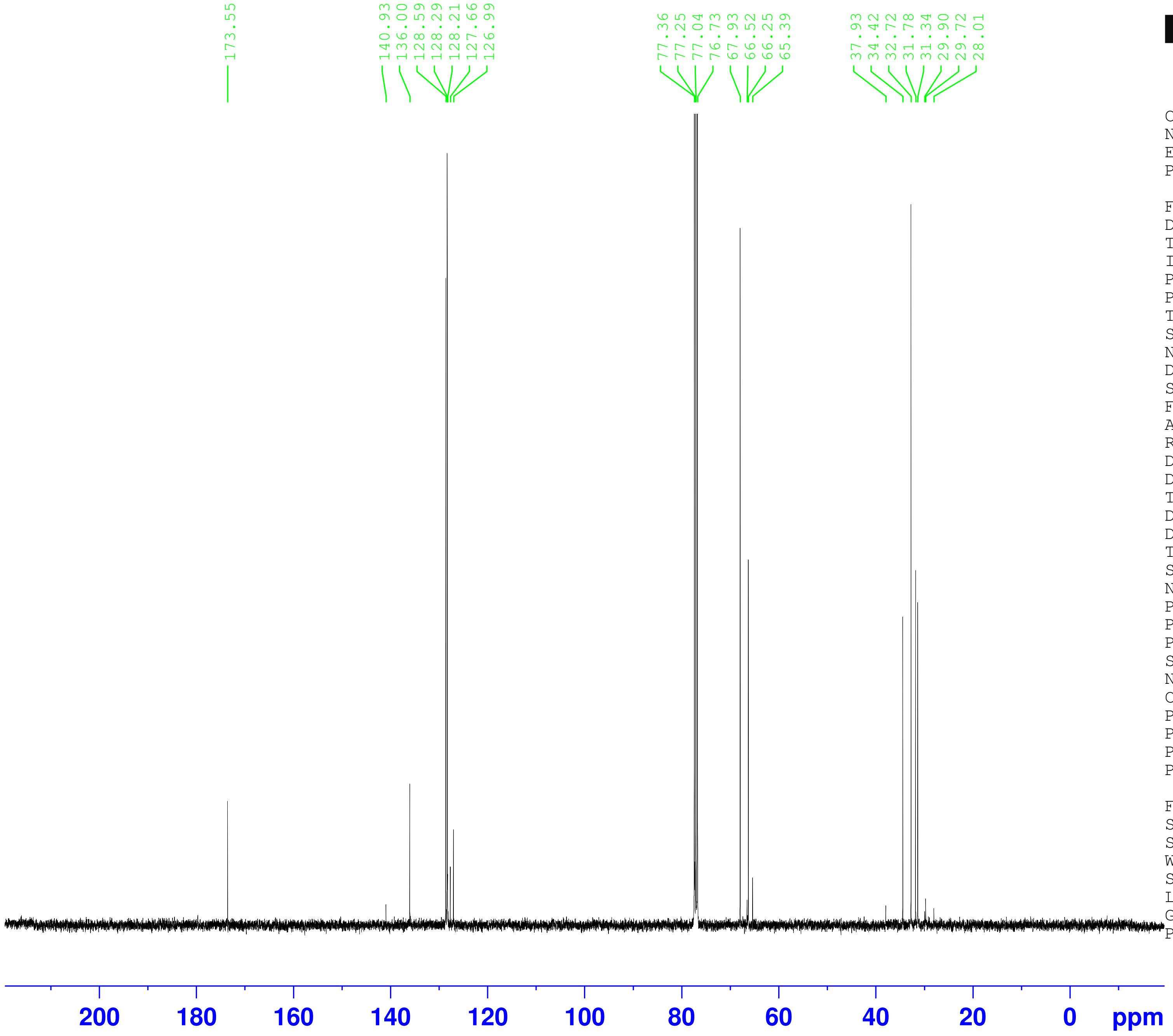


```
Current Data Parameters
NAME            RH-2234-col
EXPNO                    11
PROCNO                    1

F2 - Acquisition Parameters
Date_              20230905
Time                   4.49 h
INSTRUM               spect
PROBHD     Z116098_0222 (
PULPROG             zgpg30
TD                    65536
SOLVENT               CDCl3
NS                     1024
DS                        4
SWH               24038.461 Hz
FIDRES             0.733596 Hz
AQ                1.3631488 sec
RG                   206.31
DW                   20.800 usec
DE                     6.50 usec
TE                    296.4 K
D1               2.00000000 sec
D11              0.03000000 sec
TD0                       1
SFO1            100.6278593 MHz
NUC1                    13C
P0                     3.33 usec
P1                    10.00 usec
PLW1            70.00000000 W
SFO2            400.1516006 MHz
NUC2                     1H
CPDPRG[2            waltz65
PCPD2                 90.00 usec
PLW2            13.89000034 W
PLW12            0.17148000 W
PLW13            0.08625400 W

F2 - Processing parameters
SI                    32768
SF              100.6177975 MHz
WDW                      EM
SSB       0
LB                     1.00 Hz
GB        0
PC                     1.40
```
