# Supplementary material for: Co-Catalytic Coupling of Alkyl Halides and Alkenes: the Curious Role of Lutidine
Source: J Am Chem Soc. 2025 Feb 3;147(6):5238–46. doi: 10.1021/jacs.4c15812 (PMC11827002; doi:10.1021/jacs.4c15812)
Supplement: Supplementary file 2 — ja4c15812_si_002.zip [file ja4c15812_si_002.zip › 27a/Primary_NMR_Data_files/27a-H1.zip!/RH-2234-col/10/pdata/1/email_RH-2234-col_10_1.pdf]

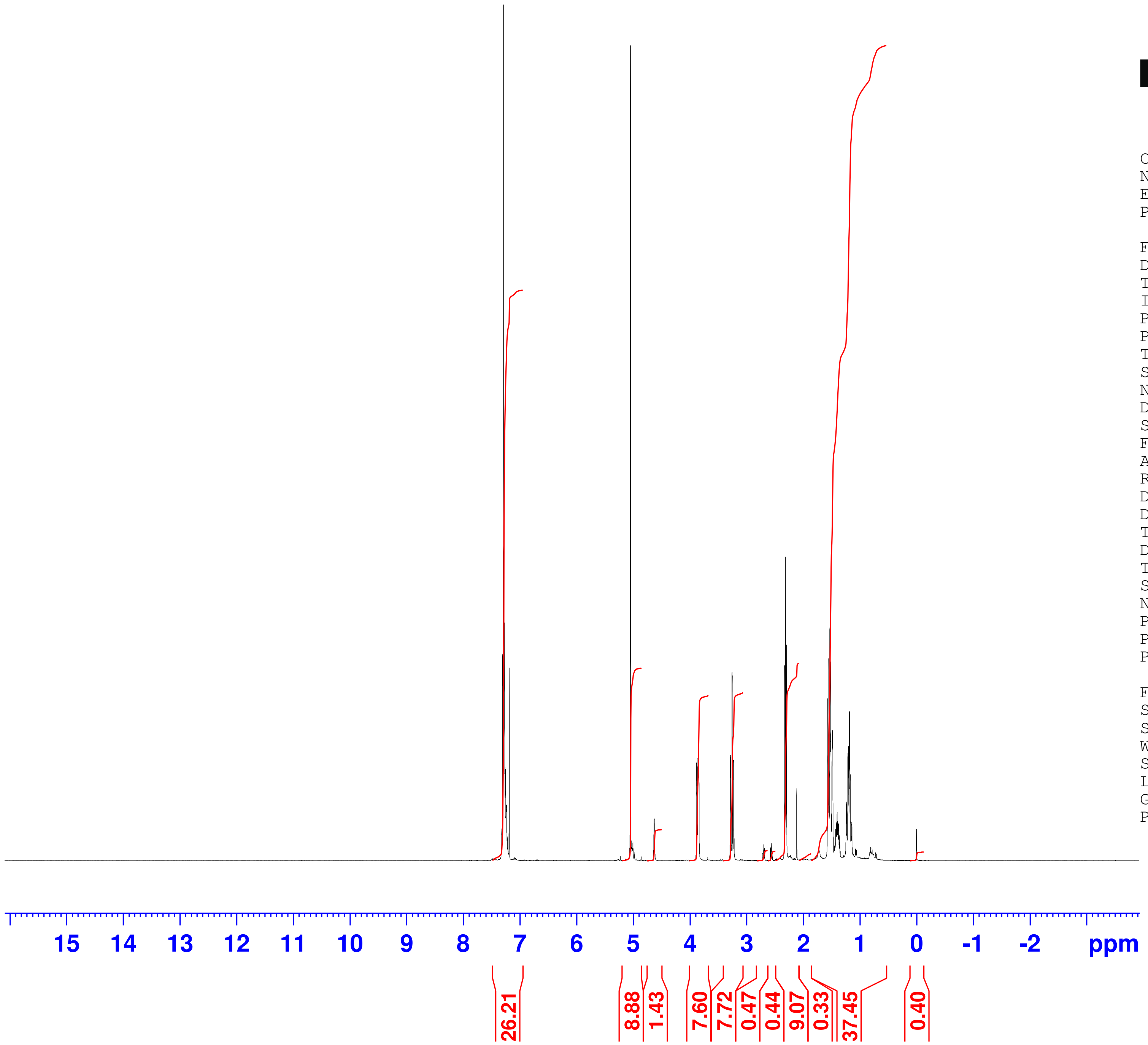

BRUKER

Current Data Parameters
NAME RH-2234-col
EXPNO 10
PROCNO 1

F2 - Acquisition Parameters

Date_	20230904	
Time	12.12	h
INSTRUM	spect	
PROBHD	Z116098_0222 (	
PULPROG	zg30	
TD	65536	
SOLVENT	CDCl3	
NS	16	
DS	2	
SWH	8012.820	Hz
FIDRES	0.244532	Hz
AQ	4.0894465	sec
RG	81.62	
DW	62.400	usec
DE	6.50	usec
TE	295.9	K
D1	1.00000000	sec
TD0	1	
SFO1	400.1524709	MHz
NUC1	1H	
P0	3.33	usec
P1	10.00	usec
PLW1	13.89000034	W

F2 - Processing parameters

SI	65536	
SF	400.1500383	MHz
WDW	EM	
SSB	0	
LB	0.30	Hz
GB	0	
PC	1.00	
