# Supplementary material for: Co-Catalytic Coupling of Alkyl Halides and Alkenes: the Curious Role of Lutidine
Source: J Am Chem Soc. 2025 Feb 3;147(6):5238–46. doi: 10.1021/jacs.4c15812 (PMC11827002; doi:10.1021/jacs.4c15812)
Supplement: Supplementary file 2 — ja4c15812_si_002.zip [file ja4c15812_si_002.zip › 37l/Primary_NMR_Data_files/37l-HSQC.zip!/RH-3126-hsqc/10/pdata/1/email_RH-3126-hsqc_10_1.pdf]

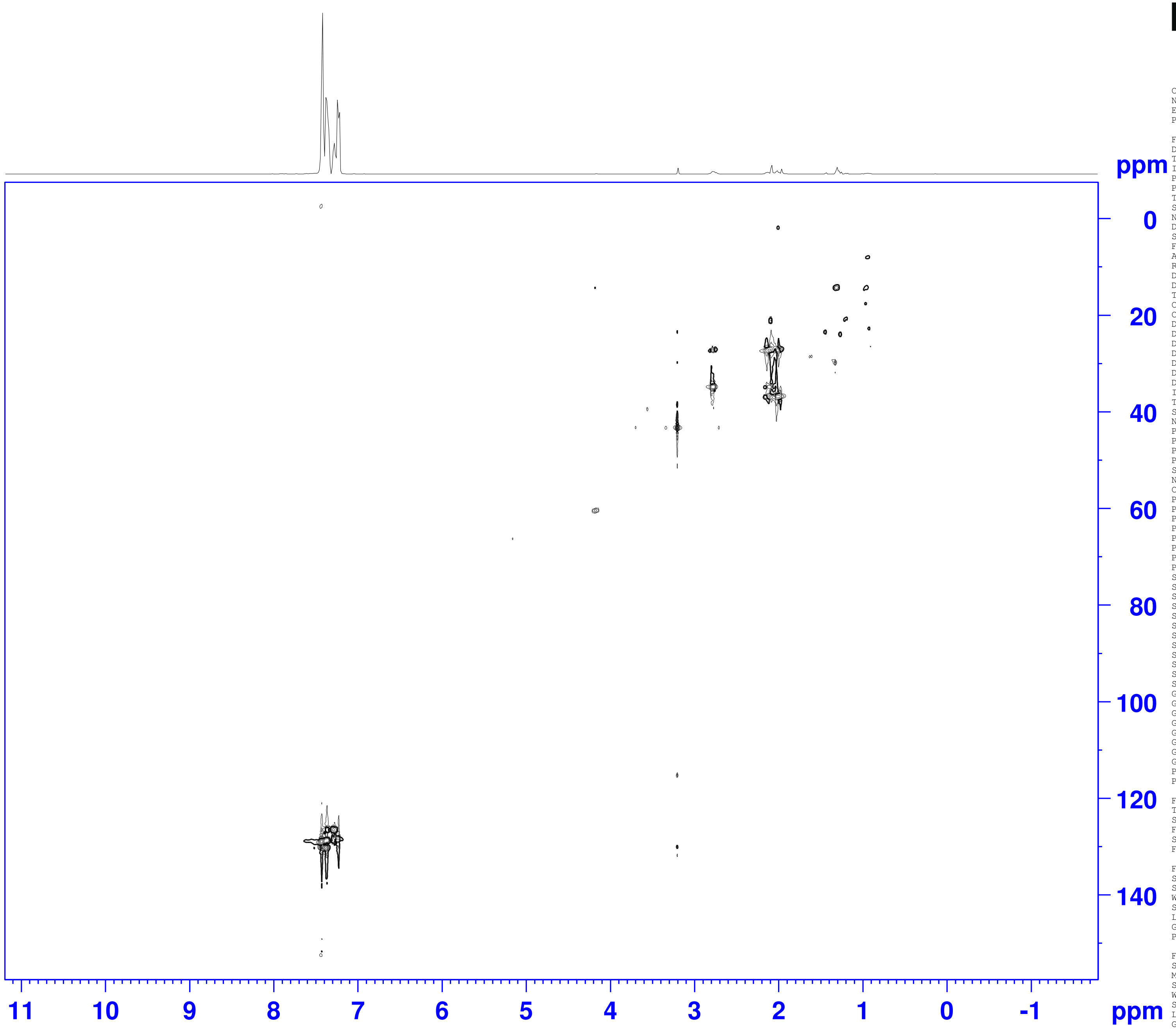

Current Data Parameters
NAME RH-3126-hsqc
EXPNO 10
PROCNO 1

F2 - Acquisition Parameters
Date_ 20240424
Time 14.21 h
INSTRUM spect
PROBHD Z116098_0222 (
PULPROG hsqcedetgpsisp2.3
TD 2048
SOLVENT CDCl3
NS 4
DS 32
SWH 5197.505 Hz
FIDRES 5.075689 Hz
AQ 0.1970176 sec
RG 206.31
DW 96.200 usec
DE 6.50 usec
TE 298.0 K
CNST2 145.0000000
CNST17 -0.5000000
D0 0.00000300 sec
D1 1.50000000 sec
D4 0.00172414 sec
D11 0.03000000 sec
D16 0.00020000 sec
D21 0.00344800 sec
D24 0.00086200 sec
IN0 0.00003010 sec
TDav 1
SFO1 400.1518807 MHz
NUC1 1H
P1 10.00 usec
P2 20.00 usec
P28 1000.00 usec
PLW1 13.89000034 W
SFO2 100.6253439 MHz
NUC2 13C
CPDPRG[2 garp4
P3 10.00 usec
P14 500.00 usec
P24 2000.00 usec
P31 2119.00 usec
PCPD2 80.00 usec
PLW0 0 W
PLW2 70.00000000 W
PLW12 1.09379995 W
SPNAM[3] Crp60,0.5,20.1
SPOAL3 0.500
SPOFFS3 0 Hz
SPW3 10.69499969 W
SPNAM[7] Crp60comp.4
SPOAL7 0.500
SPOFFS7 0 Hz
SPW7 10.69499969 W
SPNAM[18 Crp60_xfilt.2
SPOAL18 0.500
SPOFFS18 0 Hz
SPW18 2.06040001 W
GPNAM[1] SMSQ10.100
GPZ1 80.00 %
GPNAM[2] SMSQ10.100
GPZ2 20.10 %
GPNAM[3] SMSQ10.100
GPZ3 11.00 %
GPNAM[4] SMSQ10.100
GPZ4 -5.00 %
P16 1000.00 usec
P19 600.00 usec

F1 - Acquisition parameters
TD 256
SFO1 100.6253 MHz
FIDRES 129.775742 Hz
SW 165.081 ppm
FnMODE Echo-Antiecho

F2 - Processing parameters
SI 1024
SF 400.1500000 MHz
WDW QSINE
SSB 2
LB 0 Hz
GB 0
PC 1.40

F1 - Processing parameters
SI 1024
MC2 echo-antiecho
SF 100.6177975 MHz
WDW QSINE
SSB 2
LB 0 Hz
GB 0
