# Supplementary material for: Co-Catalytic Coupling of Alkyl Halides and Alkenes: the Curious Role of Lutidine
Source: J Am Chem Soc. 2025 Feb 3;147(6):5238–46. doi: 10.1021/jacs.4c15812 (PMC11827002; doi:10.1021/jacs.4c15812)
Supplement: Supplementary file 2 — ja4c15812_si_002.zip [file ja4c15812_si_002.zip › 4i/Primary_NMR_Data_files/4i-C13.zip!/RH-3062/10/pdata/1/email_RH-3062_10_1.pdf]

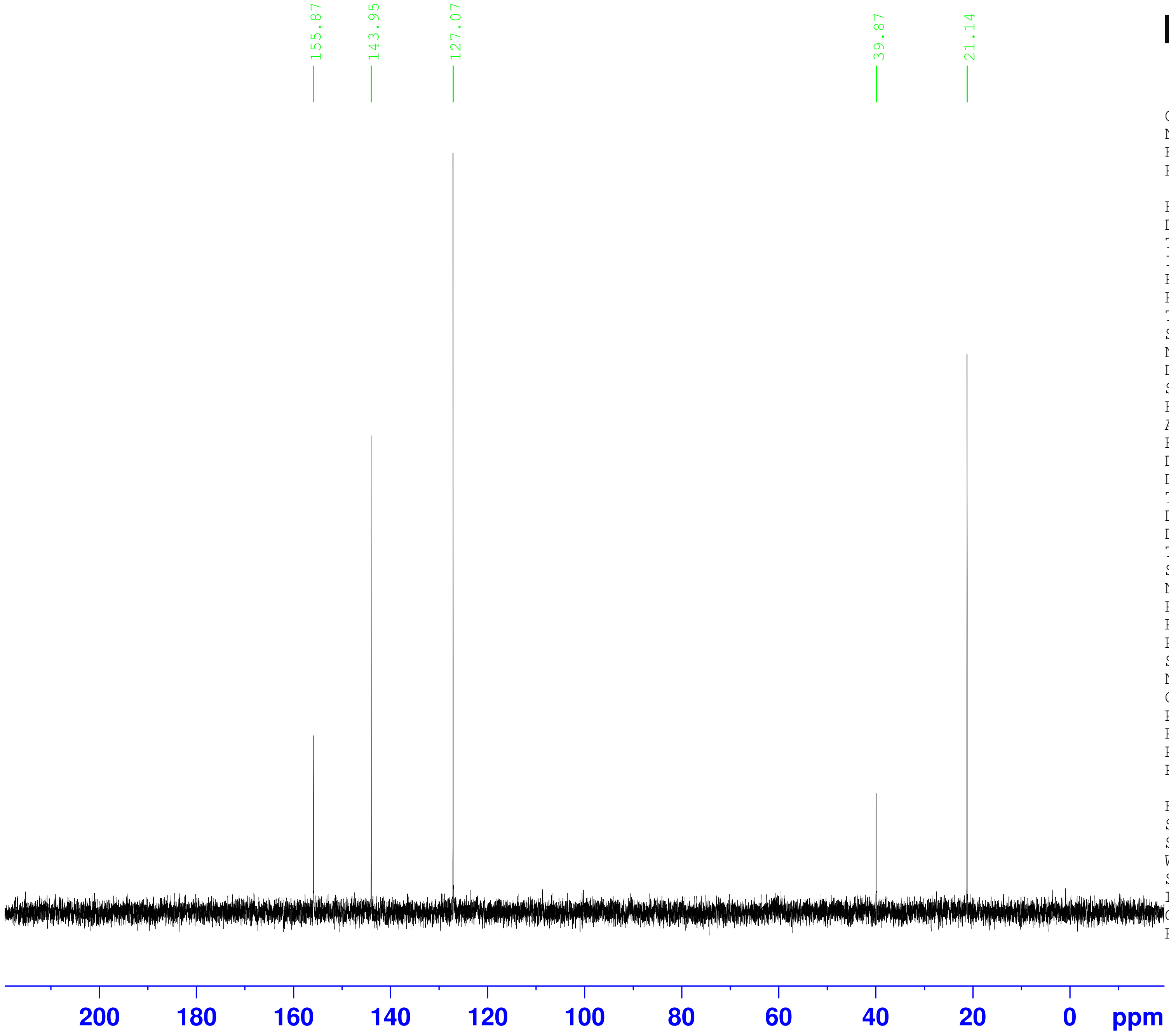

BRUKER

Current Data Parameters

NAME	RH-3062	
EXPNO	10	
PROCNO	1	

F2 - Acquisition Parameters

Date_	20240125	
Time	3.05	h
INSTRUM	spect	
PROBHD	Z116098_0222 (	
PULPROG	zgpg30	
TD	65536	
SOLVENT	D2O	
NS	1024	
DS	4	
SWH	24038.461	Hz
FIDRES	0.733596	Hz
AQ	1.3631488	sec
RG	206.31	
DW	20.800	usec
DE	6.50	usec
TE	298.0	K
D1	2.00000000	sec
D11	0.03000000	sec
TD0	1	
SFO1	100.6278593	MHz
NUC1	13C	
P0	3.33	usec
P1	10.00	usec
PLW1	70.00000000	W
SFO2	400.1516006	MHz
NUC2	1H	
CPDPRG[2	waltz65	
PCPD2	90.00	usec
PLW2	13.89000034	W
PLW12	0.17148000	W
PLW13	0.08625400	W

F2 - Processing parameters

SI	32768	
SF	100.6177975	MHz
WDW	EM	
SSB	0	
LB	1.00	Hz
GB	0	
PC	1.40	
