# Supplementary material for: Co-Catalytic Coupling of Alkyl Halides and Alkenes: the Curious Role of Lutidine
Source: J Am Chem Soc. 2025 Feb 3;147(6):5238–46. doi: 10.1021/jacs.4c15812 (PMC11827002; doi:10.1021/jacs.4c15812)
Supplement: Supplementary file 2 — ja4c15812_si_002.zip [file ja4c15812_si_002.zip › 4i/Primary_NMR_Data_files/4i-H1.zip!/RH-3062-salt/10/pdata/1/email_RH-3062-salt_10_1.pdf]

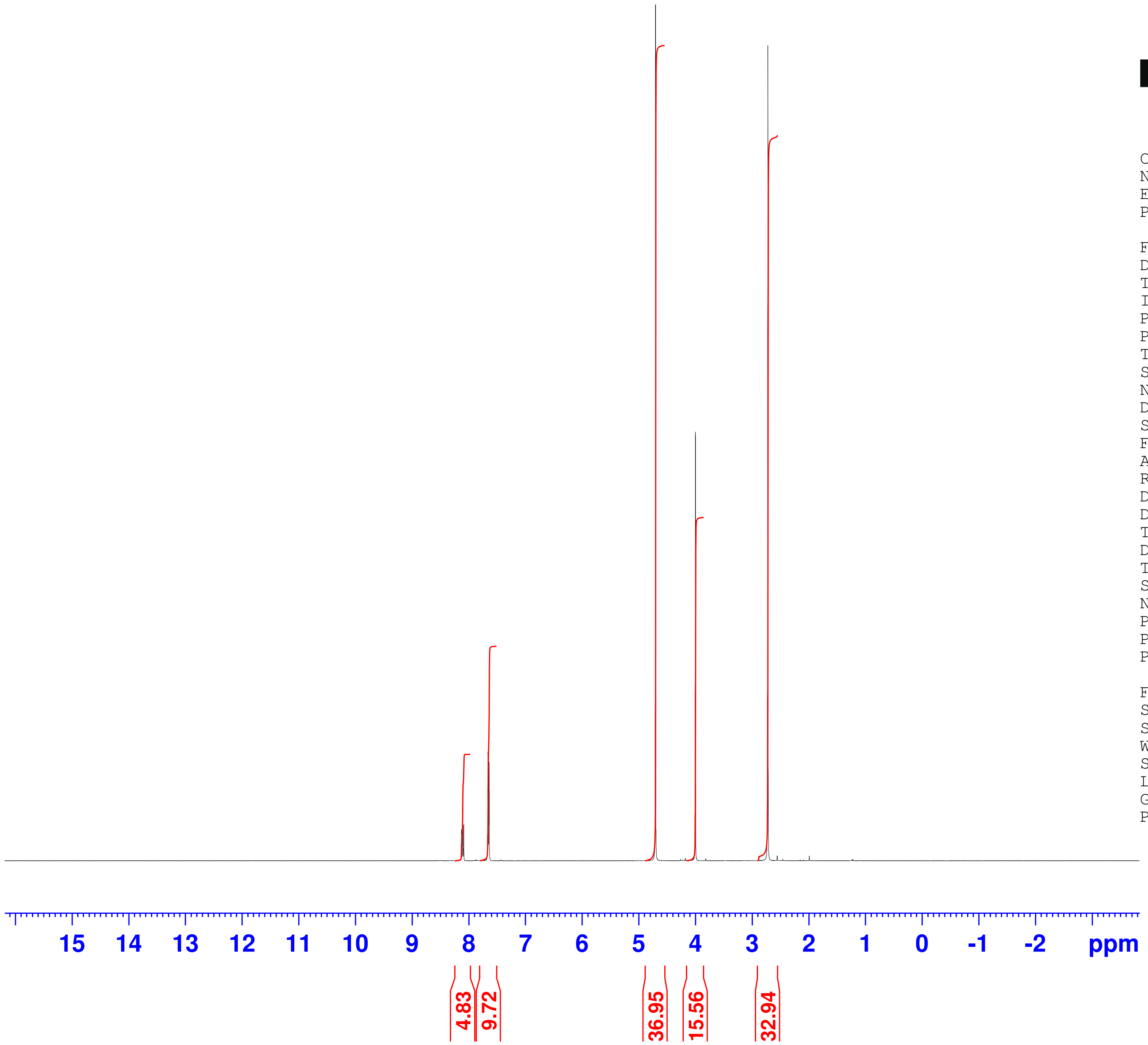

Current Data Parameters

NAME	RH-3062-salt
EXPNO	10
PROCNO	1

F2 - Acquisition Parameters

Date_	20240124
Time	12.19 h
INSTRUM	spect
PROBHD	Z116098_0222 (
PULPROG	zg30
TD	65536
SOLVENT	D2O
NS	16
DS	2
SWH	8012.820 Hz
FIDRES	0.244532 Hz
AQ	4.0894465 sec
RG	102.12
DW	62.400 usec
DE	6.50 usec
TE	298.0 K
D1	1.00000000 sec
TD0	1
SFO1	400.1524709 MHz
NUC1	1H
P0	3.33 usec
P1	10.00 usec
PLW1	13.89000034 W

F2 - Processing parameters

SI		65536
SF		400.1500000 MHz
WDW		EM
SSB	0	
LB		0.30 Hz
GB	0	
PC		1.00
