# Supplementary material for: Co-Catalytic Coupling of Alkyl Halides and Alkenes: the Curious Role of Lutidine
Source: J Am Chem Soc. 2025 Feb 3;147(6):5238–46. doi: 10.1021/jacs.4c15812 (PMC11827002; doi:10.1021/jacs.4c15812)
Supplement: Supplementary file 2 — ja4c15812_si_002.zip [file ja4c15812_si_002.zip › 4k/Primary_NMR_Data_files/4k-H1.zip!/PS-1107-H1/10/pdata/1/email_PS-1107-H1_10_1.pdf]

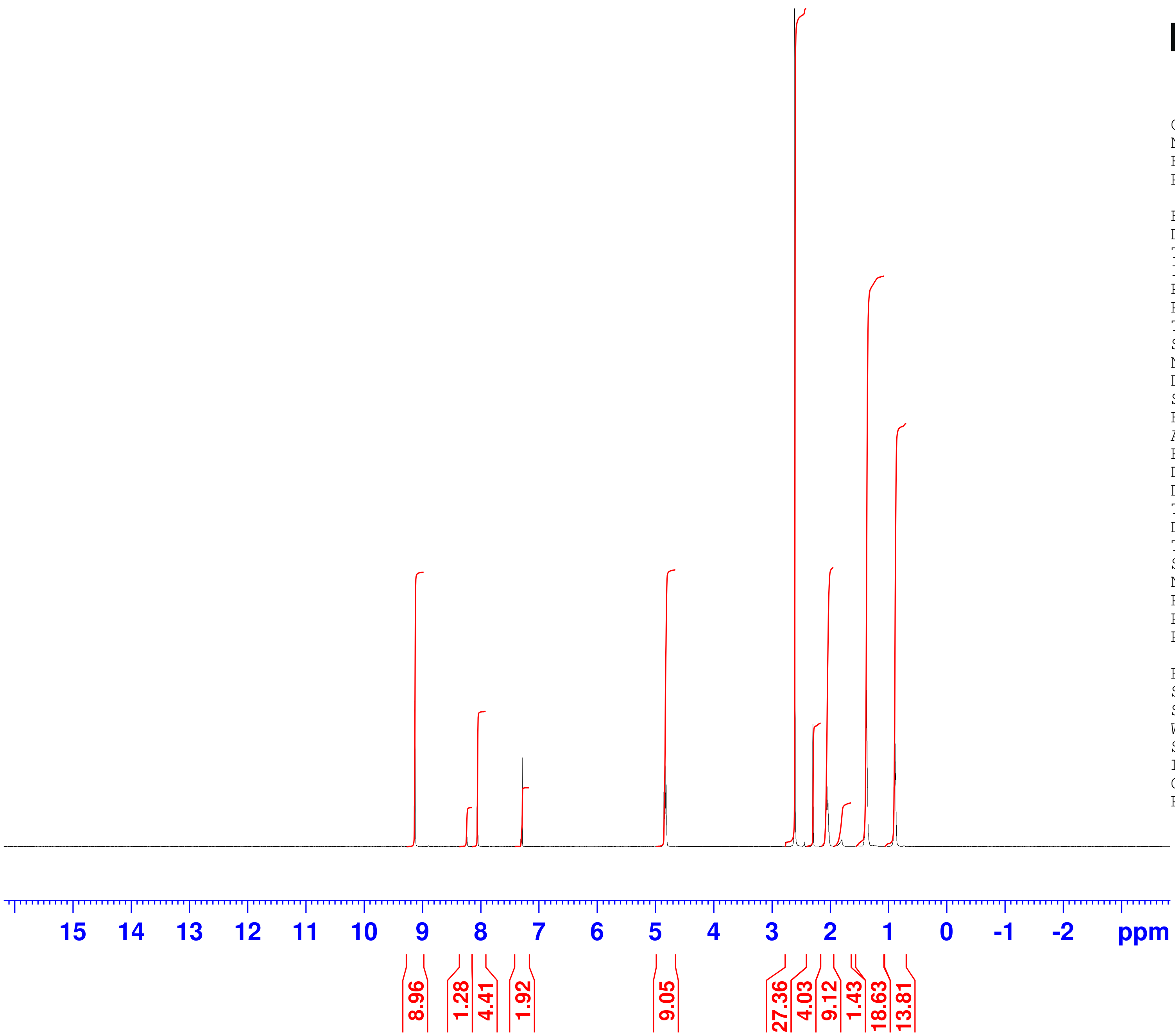


```
Current Data Parameters
NAME          PS-1107-H1
EXPNO                 10
PROCNO                 1

F2 - Acquisition Parameters
Date_           20230803
Time                7.37 h
INSTRUM            spect
PROBHD   Z116098_0222 (
PULPROG             zg30
TD                 65536
SOLVENT            CDCl3
NS                    16
DS                     2
SWH             8012.820 Hz
FIDRES          0.244532 Hz
AQ             4.0894465 sec
RG                 91.67
DW                62.400 usec
DE                  6.50 usec
TE                 298.0 K
D1            1.00000000 sec
TD0                    1
SFO1         400.1524709 MHz
NUC1                  1H
P0                  3.33 usec
P1                 10.00 usec
PLW1         13.89000034 W

F2 - Processing parameters
SI                 65536
SF           400.1500000 MHz
WDW                   EM
SSB      0
LB                  0.30 Hz
GB       0
PC                  1.00
```
