# Supplementary material for: Co-Catalytic Coupling of Alkyl Halides and Alkenes: the Curious Role of Lutidine
Source: J Am Chem Soc. 2025 Feb 3;147(6):5238–46. doi: 10.1021/jacs.4c15812 (PMC11827002; doi:10.1021/jacs.4c15812)
Supplement: Supplementary file 2 — ja4c15812_si_002.zip [file ja4c15812_si_002.zip › 4v/Primary_NMR_Data_files/4v-C13.zip!/PS-1256-A-H1/11/pdata/1/email_PS-1256-A-H1_11_1.pdf]

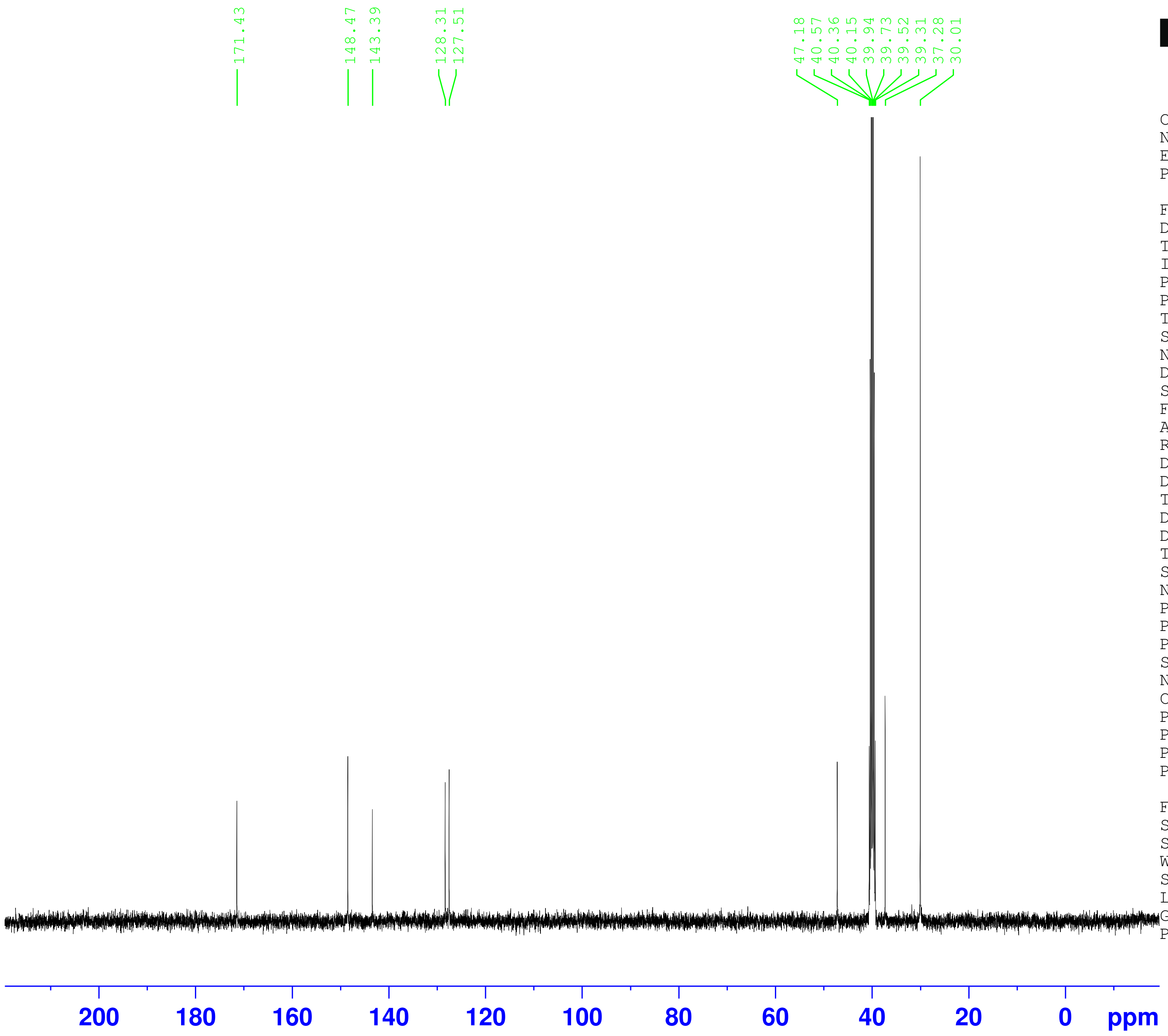

BRUKER

Current Data Parameters

NAME	PS-1256-A-H1
EXPNO	11
PROCNO	1

F2 - Acquisition Parameters

Date_	20240328
Time	12.34 h
INSTRUM	spect
PROBHD	Z116098_0222 (
PULPROG	zgpg30
TD	65536
SOLVENT	DMSO
NS	182
DS	4
SWH	24038.461 Hz
FIDRES	0.733596 Hz
AQ	1.3631488 sec
RG	206.31
DW	20.800 usec
DE	6.50 usec
TE	298.0 K
D1	2.00000000 sec
D11	0.03000000 sec
TD0	1
SFO1	100.6278593 MHz
NUC1	13C
P0	3.33 usec
P1	10.00 usec
PLW1	70.00000000 W
SFO2	400.1516006 MHz
NUC2	1H
CPDPRG[2	waltz65
PCPD2	90.00 usec
PLW2	13.89000034 W
PLW12	0.17148000 W
PLW13	0.08625400 W

F2 - Processing parameters

SI	32768
SF	100.6177975 MHz
WDW	EM
SSB	0
LB	1.00 Hz
GB	0
PC	1.40
