# Supplementary material for: Co-Catalytic Coupling of Alkyl Halides and Alkenes: the Curious Role of Lutidine
Source: J Am Chem Soc. 2025 Feb 3;147(6):5238–46. doi: 10.1021/jacs.4c15812 (PMC11827002; doi:10.1021/jacs.4c15812)
Supplement: Supplementary file 2 — ja4c15812_si_002.zip [file ja4c15812_si_002.zip › 7a/Primary_NMR_Data_files/7a-Cl.zip!/RH-3035/10/pdata/1/email_RH-3035_10_1.pdf]

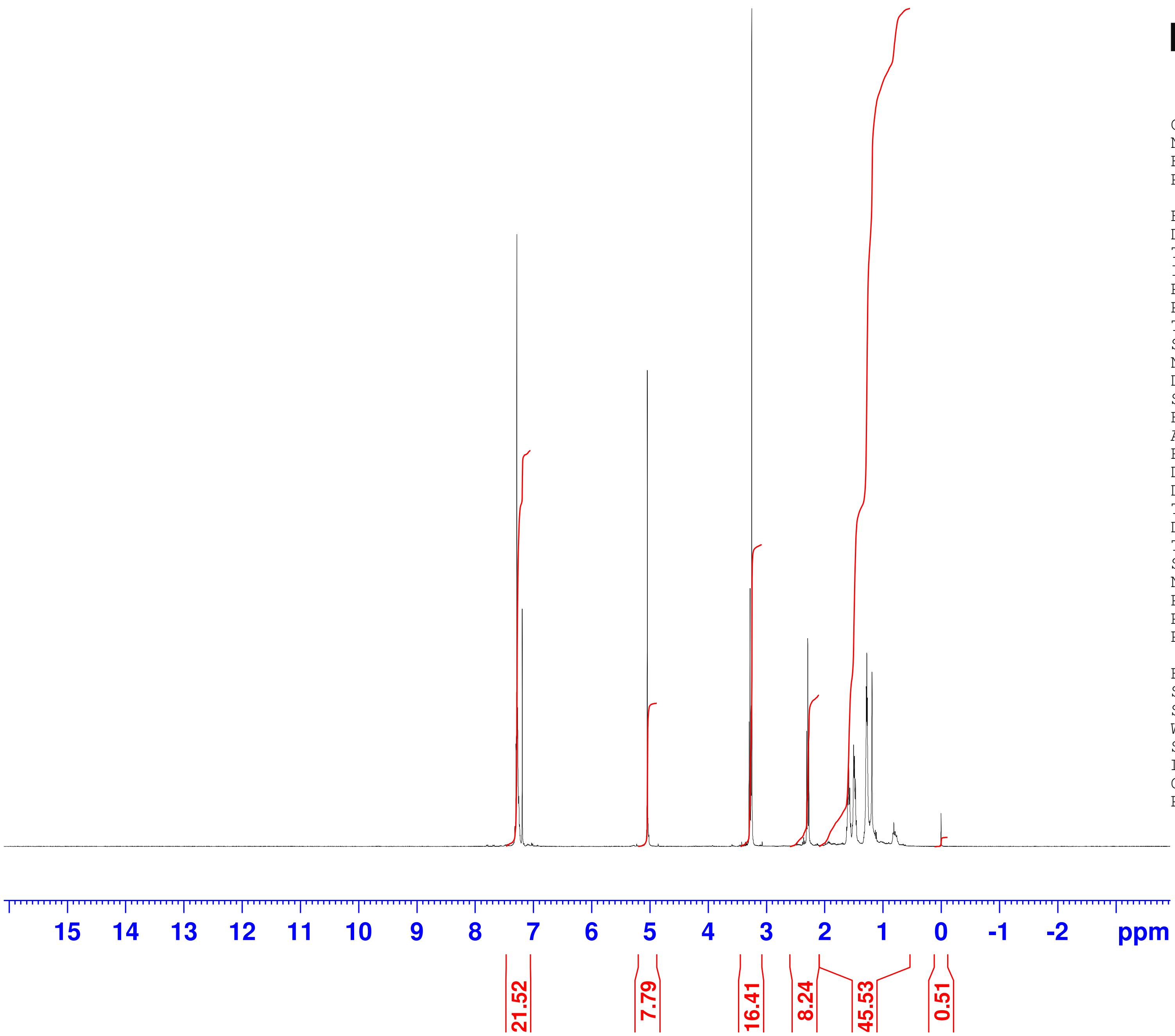

BRUKER

```
Current Data Parameters
NAME              RH-3035
EXPNO                  10
PROCNO                  1

F2 - Acquisition Parameters
Date_            20231127
Time                19.17 h
INSTRUM             spect
PROBHD   Z116098_0222 (
PULPROG              zg30
TD                  65536
SOLVENT             CDCl3
NS                     16
DS                      2
SWH              8012.820 Hz
FIDRES           0.244532 Hz
AQ              4.0894465 sec
RG                  57.93
DW                 62.400 usec
DE                   6.50 usec
TE                  298.0 K
D1             1.00000000 sec
TD0                     1
SFO1          400.1524709 MHz
NUC1                   1H
P0                   3.33 usec
P1                  10.00 usec
PLW1          13.89000034 W

F2 - Processing parameters
SI                  65536
SF            400.1500380 MHz
WDW                    EM
SSB      0
LB                   0.30 Hz
GB       0
PC                   1.00
```
