# Supplementary material for: Co-Catalytic Coupling of Alkyl Halides and Alkenes: the Curious Role of Lutidine
Source: J Am Chem Soc. 2025 Feb 3;147(6):5238–46. doi: 10.1021/jacs.4c15812 (PMC11827002; doi:10.1021/jacs.4c15812)
Supplement: Supplementary file 2 — ja4c15812_si_002.zip [file ja4c15812_si_002.zip › 8a/Primary_NMR_Data_files/8a-H1.zip!/RH-2273-col/10/pdata/1/email_RH-2273-col_10_1.pdf]

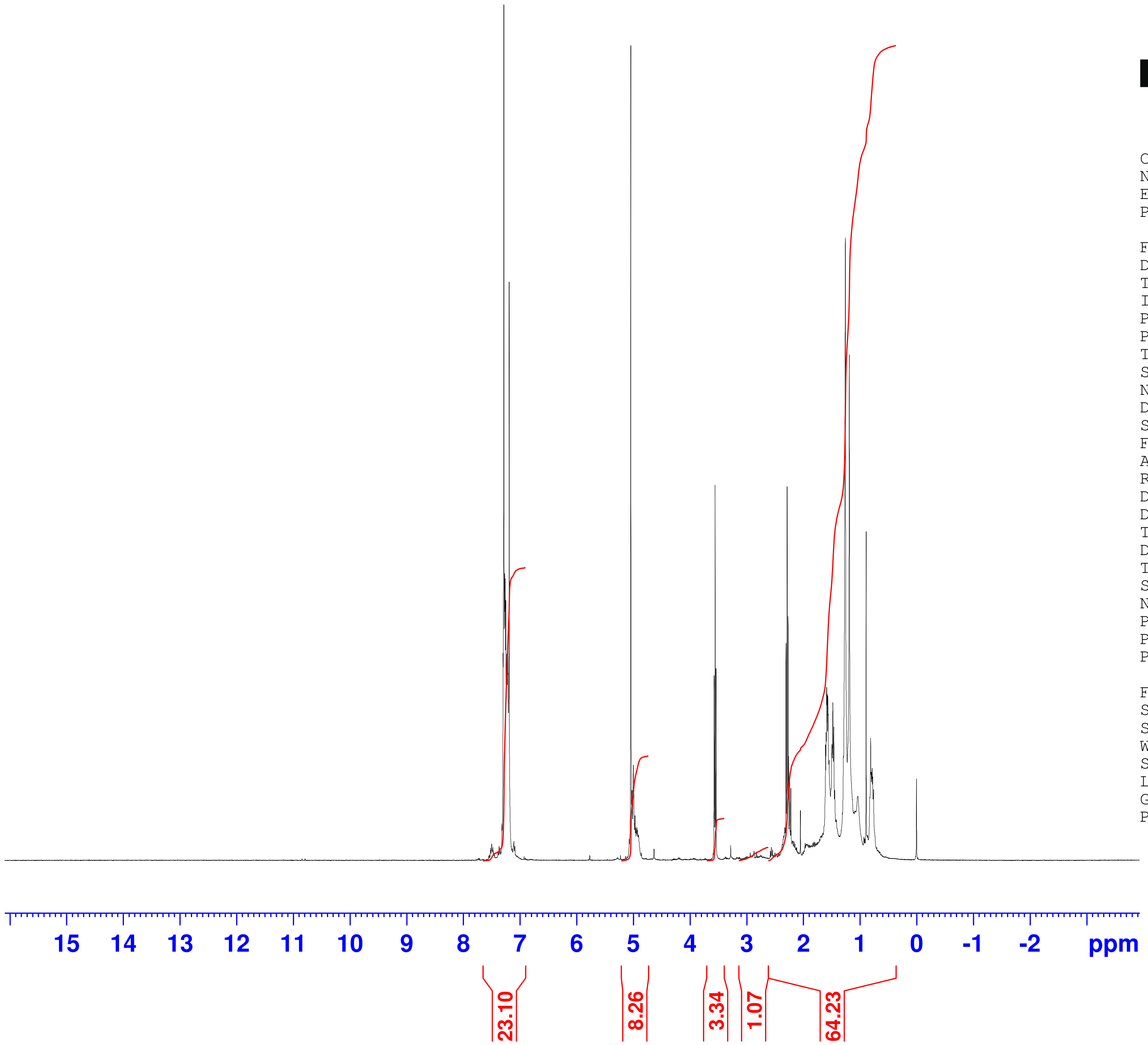

Current Data Parameters
NAME RH-2273-col
EXPNO 10
PROCNO 1

F2 - Acquisition Parameters
Date_ 20230927
Time 12.07 h
INSTRUM spect
PROBHD Z116098_0222 (
PULPROG zg30
TD 65536
SOLVENT CDCl3
NS 16
DS 2
SWH 8012.820 Hz
FIDRES 0.244532 Hz
AQ 4.0894465 sec
RG 49.53
DW 62.400 usec
DE 6.50 usec
TE 298.0 K
D1 1.00000000 sec
TD0 1
SFO1 400.1524709 MHz
NUC1 1H
P0 3.33 usec
P1 10.00 usec
PLW1 13.89000034 W

F2 - Processing parameters
SI 65536
SF 400.1500382 MHz
WDW EM
SSB 0
LB 0.30 Hz
GB 0
PC 1.00
